# Divergent functionalization of aldehydes photocatalyzed by neutral eosin Y with sulfone reagents

**DOI:** 10.1038/s41467-021-27550-8

**Published:** 2021-12-10

**Authors:** Jianming Yan, Haidi Tang, Eugene Jun Rong Kuek, Xiangcheng Shi, Chenguang Liu, Muliang Zhang, Jared L. Piper, Shengquan Duan, Jie Wu

**Affiliations:** 1 Department of Chemistry, National University of Singapore, 3 Science Drive 3, Singapore, 117543 Republic of Singapore; 2 Department of Medicinal Chemistry, College of Pharmacy, Chongqing Medical University, Chongqing, 400016 China; 3 National University of Singapore (Suzhou) Research Institute, 377 Lin Quan Street, Suzhou Industrial Park, Suzhou, Jiangsu 215123 China; 4 Pfizer Worldwide Research and Development, Eastern Point Rd, Groton, CT 06340 USA

**Keywords:** Synthetic chemistry methodology, Photocatalysis

## Abstract

While aldehydes represent a classic class of electrophilic synthons, the corresponding acyl radicals are inherently nucleophilic, which exhibits umpolung reactivity. Generation of acyl radicals typically requires noble metal catalysts or excess oxidants to be added. Herein, we report a convenient and green approach to access acyl radicals, capitalizing on neutral eosin Y-enabled hydrogen atom transfer (HAT) photocatalysis with aldehydes. The generated acyl radicals underwent SOMOphilic substitutions with various functionalized sulfones (X–SO_2_R’) to deliver value-added acyl products. The merger of eosin Y photocatalysis and sulfone-based SOMOphiles provides a versatile platform for a wide array of aldehydic C–H functionalizations, including fluoromethylthiolation, arylthiolation, alkynylation, alkenylation and azidation. The present protocol features green characteristics, such as being free of metals, harmful oxidants and additives; step-economic; redox-neutral; and amenable to scale-up assisted by continuous-flow technology.

## Abstract (web summary)

Acyl radicals represent a reactive species that allow for aldehyde subunits to be nucleophilic instead of their typical electrophilic behavior; however, these species are difficult to access in mild conditions. Here the authors show a method to generate acyl radicals using only an organic photocatalyst and light, and these species are shown as competent nucleophiles in a variety of couplings.

## Introduction

Acyl radicals are versatile synthetic intermediates in C–C bond-forming reactions, such as Giese addition^1,2^ and Minisci acylation^3^, as well as transition metal-mediated cross-coupling reactions^4–6^. The exploration of acyl radical chemistry greatly expanded the scope of accessible carbonyl-containing functional molecules^7^. However, conventional approaches to accessing acyl radicals normally require harsh conditions such as high temperature, ultraviolet irradiation, or the use of hazardous reagents. Emerging and rapidly expanding photocatalysis has offered enormous opportunities to access acyl radicals in a green and sustainable fashion from a variety of precursors, including aldehydes, carboxylic acids, acid derivatives, and acyl silanes^8–13^. Among them, the use of aldehydes for acyl radical generation represents the most straightforward and atom- and step-economical pathway.

**Fig. 1:**
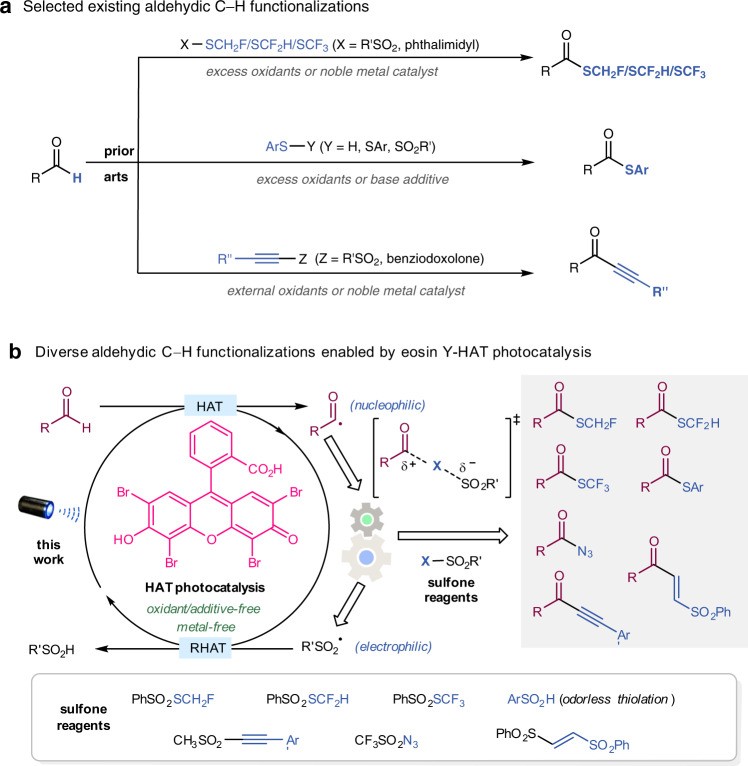
Diverse aldehydic C–H functionalizations. **a** Selected existing aldehydic C–H functionalizations. **b** This work: diverse aldehydic C–H functionalizations by HAT photocatalysis using sulfone regents.

By taking advantage of aldehyde feedstocks as acyl radical precursors^14–16^, a plethora of acyl–C bond-forming reactions have been developed. This bond formation is normally realized by acyl radical addition to unsaturated alkenes or (hetero)aromatics, leading to acyl–C(*sp*^3^) or acyl–C(*sp*^2^) bond formation. In stark contrast, the construction of acyl–X (X = S, N, D) and acyl–C(*sp*) bonds from aldehydes is largely underexplored (Fig. 1a). Excess oxidants^17,18^, additives^19,20^, or noble metal catalysts^21–24^ are usually required to achieve such transformations.

Direct photocatalyzed hydrogen atom transfer (HAT) has enabled a remarkable breakthrough in C–H functionalizations^25,26^. Capitalizing on HAT activity of C–O biradical of excited ketones or quinones, a series of HAT photocatalysts were developed^27–31^. Although anionic eosin Y was commonly used as a photoredox catalyst^32–34^, a previous study by our group established neutral eosin Y as an excellent direct HAT photocatalyst that can activate a wide range of C–H bonds to access the corresponding carbon radicals under simple and mild conditions^35^. Eosin Y was capable of providing access to acyl radicals from aldehyde feedstocks, which have been employed to react with alkenes and alkynes for asymmetric 1,4-addition^36^ and a radical Smiles rearrangement^37^, respectively. With the cumulative insights gained from the eosin Y-HAT photocatalytic system^38^, we envisioned that its merger with sulfone SOMOphiles^39,40^ can provide a general platform for aldehydic C–H functionalization to access various types of functional acyl compounds. Acyl radicals generated by photoinduced HAT undergo nucleophilic substitution with X–SO_2_R′, which will benefit from polarity matching^41–43^, delivering diverse functionalized acyl compounds accompanied by electrophilic sulfonyl radical species. The sulfonyl radical then participates in a reversed HAT (RHAT) process with eosin Y-H to complete the catalytic cycle.

In this work, by using different SOMOphilic sulfone reagents as acyl radical traps in HAT photocatalysis, we achieve aldehydic C–H fluoromethylthiolation, arylthiolation, alkynylation, alkenylation, and azidation (Fig. 1b). Notably, arylsulfinic acid is utilized as an odorless thiolation reagent for thioester generation. Compared to existing protocols for aldehydic C–H functionalizations, eosin Y-HAT photocatalysis features operationally simple, inexpensive, and metal-, oxidant-, and additive-free green attributes.

## Results

### Development of aldehydic C–H fluoromethylthiolation

The monofluoromethylthio moiety (SCH_2_F) widely exists in a variety of biologically active compounds (Supplementary Fig. 1)^44,45^. Previous reports to achieve aldehydic C–H monofluoromethylthiolation relied on the use of stoichiometric oxidants such as 2,2′-azodi(2-methylbutyronitrile) (AMBN)^46^ or PhI(O_2_CCF_3_)_2_/NaN_3_^17^. We envisioned that eosin Y-based HAT photocatalysis may enable this transformation in a redox-neutral fashion. After extensive condition optimization using benzaldehyde **1a** and *S*-(fluoromethyl) benzenesulfonothioate (PhSO_2_–SCH_2_F) **2a** as the model substrates (Supplementary Table 1), we found that neutral eosin Y (4 mol%) in *tert*-butanol (*t*BuOH) under blue light (18 W, 470 nm LED) irradiation at ambient temperature afforded desired product **3a** in optimal yield (88%). Notably, no product was generated using anionic eosin Y as the photocatalyst, while other photocatalytic systems for HAT^4,22,24,27,47^ gave inferior product yields (Supplementary Table 4), highlighting the effectiveness of eosin Y catalysis. Light irradiation was essential, as no product was detected when the reaction was performed in darkness.

**Fig. 2:**
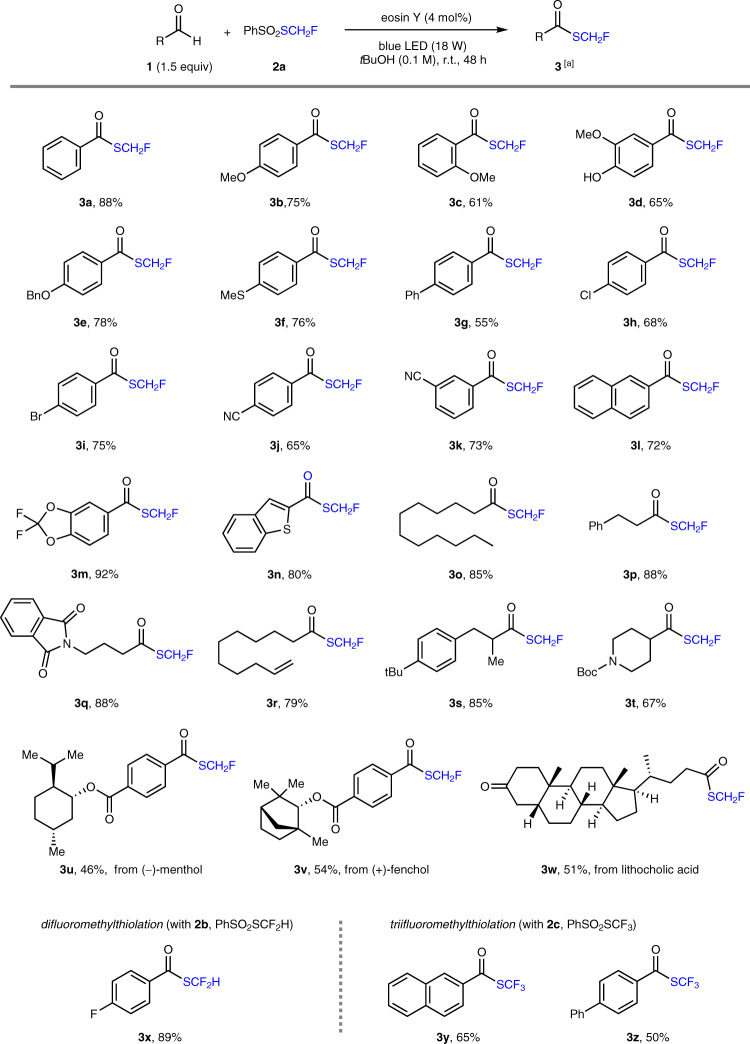
Aldehydes scope for C–H fluoromethylthiolation. [a] Reaction conditions unless otherwise noted: **1** (0.3 mmol), **2** (0.2 mmol), eosin Y (4 mol%), and *tert*-butanol (2.0 mL) in an argon-filled Schlenk tube (20 mL) at room temperature (~27 °C) under 470 nm light (18 W LED) irradiation.

With the optimized conditions, the scope of aldehydes amenable to monofluoromethylthiolation was investigated (Fig. 2). Electron-rich (**3b**–**f**) and electron-deficient (**3g**–**k**) arene derivatives possessing ortho-, meta-, or para-substituents all provided the corresponding monofluoromethyl thioester products in 55–78% yields. A wide range of functionalities, including ether (**3b**–**e**), phenol (**3d**), thioether (**3f**), halide (**3h**, **3i**), and cyanide (**3j**), were well tolerated. Naphthalene- or heterocycle (such as benzodioxole and benzothiophene)-substituted aldehydes smoothly participated in the transformation to afford products **3l**–**n** in good yields (72–92%). The scope with respect to aliphatic aldehydes was evaluated next. Both linear (**3o**–**r**) and branched (**3s**, **3t**) alkyl aldehydes afforded the desired products in good yields (67–88%). The incorporation of amide (**3q**), terminal alkene (**3r**) and piperidine (**3t**) substrates was compatible with our conditions. However, tertiary aldehydes such as pivalaldehyde failed to give the corresponding product (not shown), probably due to the facile decarbonylation of the unstable *tert*-alkyl acyl radical^48^. Moreover, the protocol can be applied to late-stage functionalization of natural product derivatives. Useful yields (46–54%) of monofluoromethylthiolation products were obtained with complex molecules derived from (−)-menthol (**3u**), (+)-fenchol (**3v**), and lithocholic acid (**3w**). Importantly, by simply changing fluoromethylthio-sulfone reagents **2a** to **2b** (PhSO_2_–SCF_2_H) and **2c** (PhSO_2_–SCF_3_), this protocol could be successfully extended to aldehydic C–H difluoromethylthiolation (**3x**) and trifluoromethylthiolation (**3y**, **3z**), respectively, representing a general method to access diverse fluoromethylthioesters in a simple and green manner.

### Development of aldehydic C–H thiolation using arylsulfinic acid as an odorless sulfur reagent

**Fig. 3:**
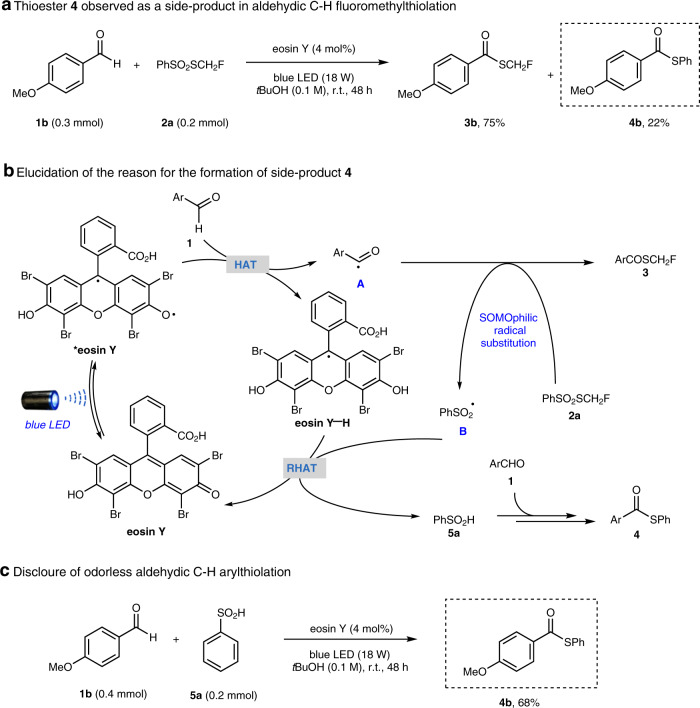
Discovery of aldehydic C–H thiolation using arylsulfinic acid as an odorless sulfur reagent. **a** Thioester **4** observed as a side-product in aldehydic C–H fluoromethylthiolation. **b** Possible rationale for the formation of side-product **4**. **c** Development of odorless aldehydic C–H thiolation.

During the study of aldehydic C–H fluoromethylthiolation, we found that the eosin Y-photocatalyzed reaction between 4-methoxybenzaldehyde **1b** and **2a** gave major product **3b** accompanied by *S*-phenyl thioester **4b** in >20% yield (Fig. 3a). In light of our previous study on HAT photocatalysis^34^ and related reports on photocascade catalysis^49–51^, we speculated that *S*-phenyl thioester **4b** might be derived from benzenesulfinic acid **5a** generated in situ (Fig. 3b). The photo-generated acyl radical **A** underwent radical substitution with **2a** to deliver monofluorothiolation product **3** and benzenesulfonyl radical **B** simultaneously. RHAT with eosin Y-H would convert benzenesulfonyl radical **B** to benzenesulfinic acid **5a**, which accumulated in the reaction mixture and served as the sulfur reagent for thiolation. This hypothesis was verified by treatment of **1b** with **5a** under eosin Y photocatalysis conditions, which delivered thioester **4b** in 68% yield (Fig. 3c).

**Fig. 4:**
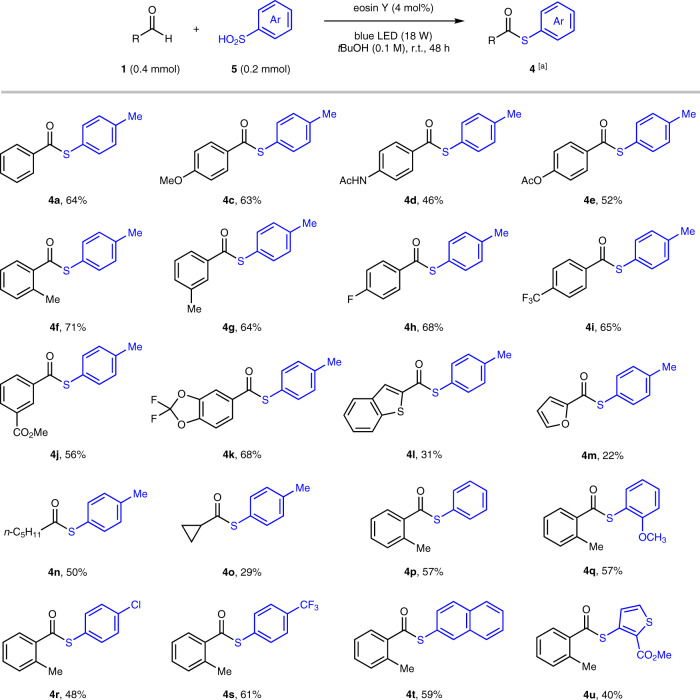
Scope of the odorless aldehydic C–H thiolation. [a] Reaction conditions unless otherwise noted: **1** (0.4 mmol), **2** (0.2 mmol), eosin Y (4 mol%), and *tert-*butanol (2.0 mL) in an argon-filled Schlenk tube (20 mL) at room temperature (~27 °C) under 470 nm light (18 W LED) irradiation.

Given the synthetic value of thioesters^52^ and the appealing property of arylsulfinic acid as an odorless and readily available sulfur reagent, we attempted to examine the arylthiolation scope of this operationally simple method (Fig. 4). A diverse set of aromatic aldehydes took part in this transformation to give moderate to good yields (46–71%) of thioester products (**4a**–**k**), tolerating a variety of functional moieties such as amide (**4d**), ester (**4e**, **4j**), halide (**4** **h**), trifluoromethyl (**4i**), and 2,2-difluorobenzodioxole (**4k**) groups. Aromatic aldehydes with para-, meta-, or ortho-substituents smoothly participated in the transformation. Heteroaryl (**4l**, **4m**) and alkyl (**4n**, **4o**) aldehydes were also amenable to thiolation, but with lower yields (22–50%), where a substantial amount of sulfonic acids was observed as the side-products. Furthermore, variation with respect to arylsulfinic acids was evaluated, illustrating that a broad scope of (hetero)arylsulfinic acids afforded the corresponding thioesters (**4p**–**u**) in useful yields (40–61%).

**Fig. 5:**
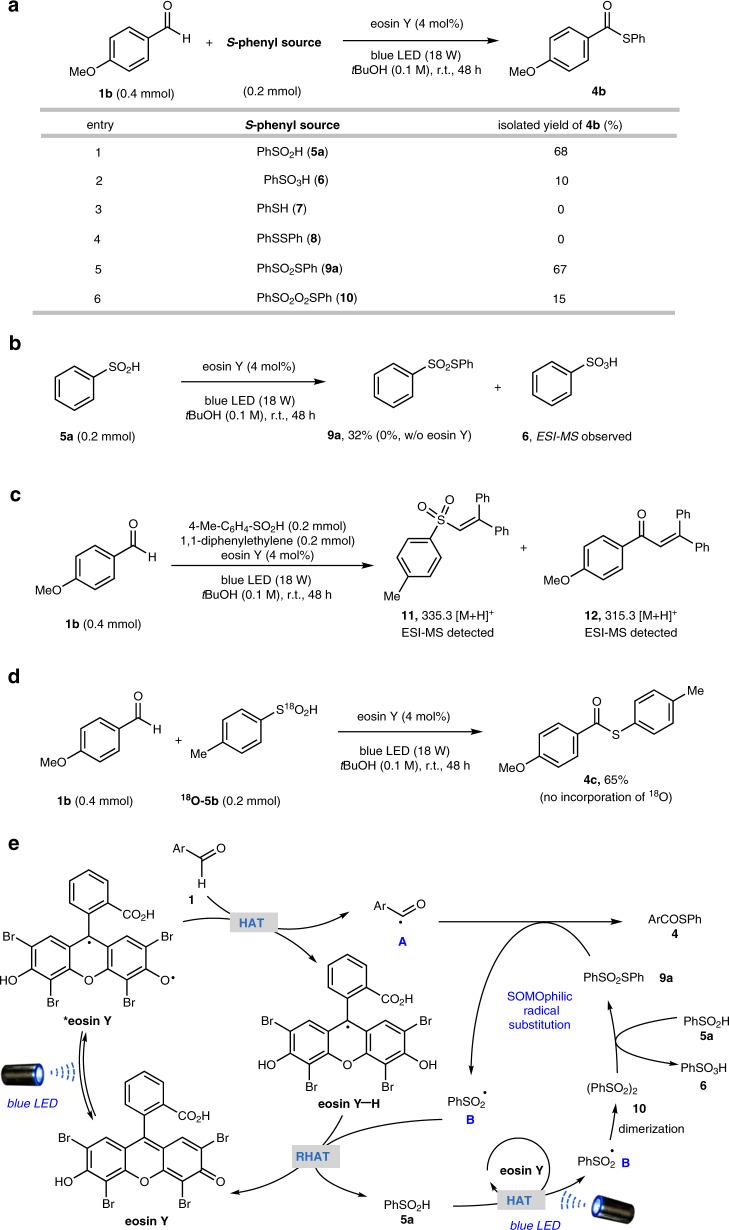
Proposed mechanism of aldehydic C–H arylthiolation with supporting evidence. **a** Evaluation of different *S*-phenyl sources. **b** Eosin Y photocatalytic formation of *S*-phenyl benzenethiosulfonate **9a** from benzenesulfinic acid **5a**. **c** Radical trapping experiments. **d** ^18^O-Labeling experiments. **e** Proposed plausible mechanisms.

To gain a mechanistic understanding of the aldehydic C–H arylthiolation, a range of control experiments were performed to elucidate the reaction intermediates. Various *S*-phenyl sources were examined (Fig. 5a). Both benzenesulfinic acid **5a** and *S*-phenyl benzenesulfonothioate **9a** could afford thioester **4b** in good yields, while other *S*-aryl sources such as benzenesulfonic acid **6**, thiol **7**, disulfide **8**, and disulfone **10** were not effective at all or delivered **4b** in very low yields. These results indicated that arylthiosulfonate **9** may act as a key intermediate in the reaction. This was further supported by the fact that benzenesulfinic acid **5a** could be converted to *S*-phenyl benzenethiosulfonate **9a** under eosin Y photocatalytic conditions, but **9a** could not be generated in the absence of eosin Y (Fig. 5b). The reaction became sluggish with the addition of radical scavengers such as 2,2,6,6-tetramethylpiperidin-1-yl)oxyl (TEMPO) and butylated hydroxytoluene (BHT), supporting a radical-based mechanism. When 1,1-diphenylethylene was added as an additive, adducts **11** and **12** were detected by electrospray ionization mass spectrometry (ESI-MS), indicating the presence of arylsulfonyl radicals and acyl radicals, respectively (Fig. 5c). Moreover, ^18^O incorporation was not observed when ^18^O-labeled 4-methylbenzenesulfinic acid ^18^O-**5b**^53^ was employed, which suggested that the carbonyl oxygen in the thioester product was derived from the aldehyde (Fig. 5d).

In light of all the experimental data and related literature, a tentative mechanistic pathway for aldehydic C–H arylthiolation is proposed (Fig. 5e). Photo-excited *eosin Y undergoes HAT with benzenesulfinic acid **5a** to generate benzenesulfonyl radical **B**^54^, which dimerizes to give disulfone species **10**. **10** is reduced by benzenesulfinic acid **5a** to give thiosulfonate **9a**^55,56^. **9a** then participates in radical substitution with acyl radical **A**, which is formed via eosin Y-HAT photocatalysis with aldehyde **1**. Arythioester product **4** is obtained together with arylsulfonyl radical **B**, which closes the photocatalytic cycle and regenerates benzenesulfinic acid **5a** (see Supplementary Fig. 12 for more discussion**)**.

### Development of Aldehydic C–H Alkynylation

**Fig. 6:**
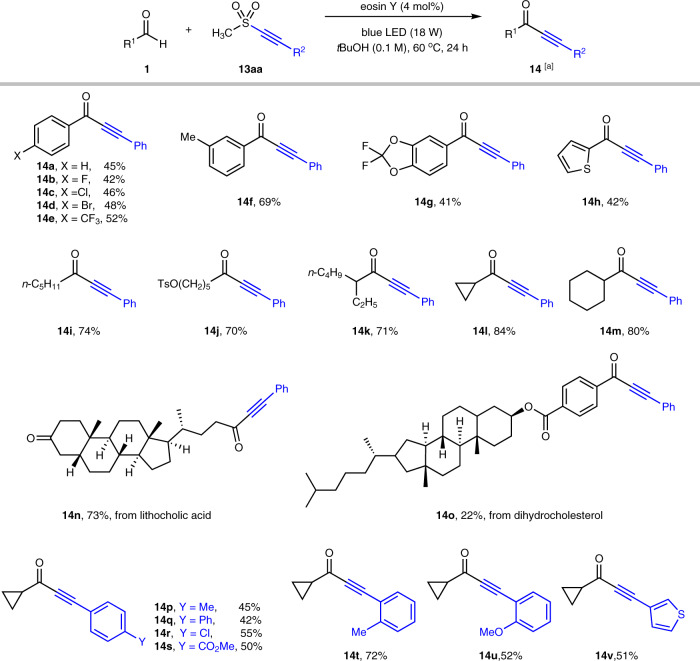
Substrate scope of aldehydic C–H alkynylation. [a] Reaction conditions: **1** (0.4 mmol), **13** (0.2 mmol), eosin Y (4 mol%), and *tert*-butanol (2.0 mL) in an argon-filled Schlenk tube (20 mL) at 60 °C under 470 nm light (18 W LED) irradiation.

To further demonstrate HAT photocatalysis by eosin Y with sulfone reagents as a versatile platform for aldehydic C–H functionalizations, we found that methanesulfonyl alkyne **13aa** was a suitable alkynylation reagent to deliver synthetically valuable ynone compounds (Fig. 6). A series of alkynyl sulfones bearing different R′SO_2_ group were examined and methanesulfonyl alkyne **13aa** was identified as the optimal reagent, probably due to the small steric hinderance (see Supplementary Tables 2 and 3 for optimization study). Diverse substituents (e.g., F, Cl, Br, CF_3_) could be incorporated in arylaldehydes to produce ynones **14a**–**g** in moderate yields (41–69%). Aldehydes possessing heteroaromatics such as thiophene (**14h**) were suitable substrates as well. Aliphatic aldehydes including both linear (**14i**, **14j**) and branched (**14k**–**m**) alkyl substituents participated in the alkynylation more efficiently to afford ynones in good yields (70–84%). Moreover, aldehydes derived from lithocholic acid and dihydrocholesterol underwent the transformations smoothly, leading to the facile formation of **14n** (73%) and **14o** (22%), respectively. Next, the scope regarding alkynyl sulfone **13** was explored. The protocol was feasible for both arylalkynyl sulfones (**14p**–**u**) bearing a variety of functionalities (e.g., Cl, CO_2_Me, OMe) and heteroarylalkynyl sulfones (**14v**) to produce the corresponding ynones with moderate to good yields (42–72%).

### Preliminary study on aldehydic C–H azidation and alkenylation and scale-up in continuous-flow reactors

**Fig. 7:**
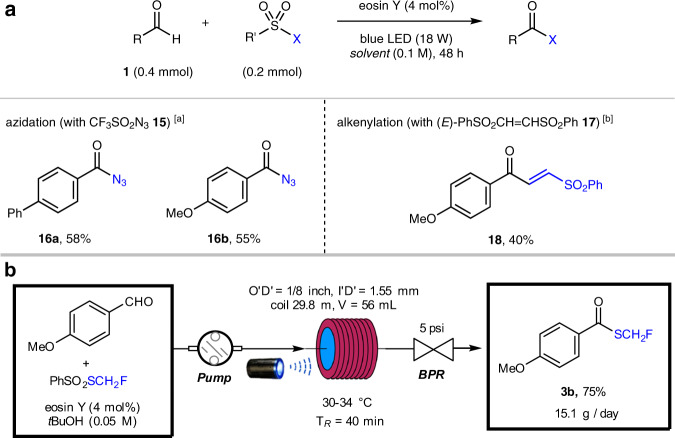
Aldehydic C–H diversification and scale-up synthesis in flow. **a** Aldehydic C–H azidation and vinylation. Reaction conditions unless otherwise noted: **1** (0.4 mmol), sulfone (0.2 mmol), eosin Y (4 mol%), and solvent (2.0 mL) in an argon-filled Schlenk tube (20 mL) under 470 nm light (18 W LED) irradiation. [a] Reaction was performed in CH_3_CN at room temperature. [b] Reaction was performed in ethyl acetate at 80 °C. **b** Reaction scale-up in continuous-flow microtubing reactors. Psi pound per square inch.

To further showcase the versatility of eosin Y-HAT photocatalysis with sulfone reagents, aldehydic C–H azidation and alkenylation were investigated (Fig. 7a). Our preliminary study illustrated that triflic azide (CF_3_SO_2_N_3_) **15** could be applied to forge C–N bonds, delivering acyl azides (**16a**, **16b**) in moderate yields (55–58%). This protocol provides a mild alternative to previous aldehydic C–H azidation reactions, which require either excess oxidants^57,58^ or inconvenient reagents^59^. Aldehydic C–H alkenylation was also feasible using (*E*)-1,2-bis(phenylsulfonyl)ethene **17** as the alkenylation reagent, delivering enone **18** in 40% yield. Finally, the aldehydic C–H monofluoromethylation reaction was smoothly transferred to an operationally simple continuous-flow reactor to achieve >15 g per day production, indicating the potential of our strategy for large-scale synthesis (Fig. 7b).

## Discussion

In summary, we have demonstrated a versatile platform for aldehydic C–H functionalization by merging neutral eosin Y-HAT photocatalysis with a variety of sulfone-based SOMOphiles to directly construct acyl–S, acyl–C and acyl–N bonds. The eosin Y-sulfone system will serve as a more green and sustainable method with easier handling for aldehydic functionalization compared to existing catalytic/stoichiometric systems and has several practically or mechanistically notable features. First, PhSO_2_–SCH_*x*_F_*y*_ **2** proved particularly effective for metal-, additive- and oxidant-free fluoromethythiolation with diverse aldehydes, including complex natural product derivatives. The fluoromethythiolation reaction was performed under continuous-flow conditions to achieve a productivity of 15 g per day. Second, arylsulfinic acid (ArSO_2_H), as an odorless and easily accessible reagent, was employed for arylthiolation of aldehydes. A preliminary mechanistic study supported that an in situ-generated arylthiosulfonate (ArSO_2_–SAr) species participated in the subsequent radical substitution step. Third, the acyl–C(*sp*) bond was successfully forged via acyl radical addition-sulfonyl radical elimination with a methanesulfonyl alkyne. Finally, exploiting the versatility of radical addition-elimination, the present strategy was further extended to aldehydic C–H azidation and alkenylation. Extension of the C–H substrate scope to abundant alkanes and expanding the chemical space of sulfone SOMOphilic reagents to new reaction patterns are currently under investigation in our laboratory.

## Methods

### General procedure of neutral-eosin Y-photocatalyzed aldehydic C–H fluoromethylthiolation

A 20 mL Schlenk tube equipped with a magnetic stir bar was charged with eosin Y (0.008 mmol, 5.2 mg), aldehyde **1** (0.3 mmol), and fluoromethylthiolation reagents **2** (0.2 mmol). Then, 2.0 mL of anhydrous *tert*-butanol was added. The Schlenk tube was connected to Schlenk line and freeze–pump–thaw was performed for three times to completely remove air inside the reaction mixture. Eventually the Schlenk tube was refilled with an atmosphere of argon at room temperature and sealed. The reaction vessel was surrounded by a coil of blue LED strip (2 m, 18 W). Then the reaction was running at ambient temperature (~27 ^o^C) using a fan to cool down the reaction mixture and stopped after 48 h. The solvent was removed under reduced pressure and the crude mixture was purified by silica gel column chromatography or prepared TLC (eluent: hexane/diethyl ether or hexane/ethyl acetate; 10/1–3/1) to give the corresponding product **3**. Note that the workup procedure was performed under weak vacuum (~50 mbar) and low temperature (~30 ^o^C) due to volatility of the corresponding product **3**.

### General procedure of neutral-eosin Y-photocatalyzed aldehydic C–H arylthiolation

A 20 mL Schlenk tube equipped with a magnetic stir bar was charged with eosin Y (0.008 mmol, 5.2 mg), aldehyde **1** (0.4 mmol), and arylsulfinic acid **5** (0.2 mmol). Then, 2.0 mL of anhydrous *tert*-butanol was added. The Schlenk tube was connected to Schlenk line and freeze–pump–thaw was performed for three times to completely remove air inside the reaction mixture. Eventually the Schlenk tube was refilled with an atmosphere of argon at room temperature and sealed. The reaction vessel was surrounded by a coil of blue LED strip (2 m, 18 W). Then the reaction was running at ambient temperature (~27 ^o^C) using a fan to cool down the reaction mixture and stopped after 48 h. The solvent was removed under reduced pressure and the crude mixture was purified by silica gel column chromatography or prepared TLC (eluent: hexane/diethyl ether or hexane/ethyl acetate; 10/1–3/1) to give the corresponding product **4**.

### General procedure of neutral-eosin Y-photocatalyzed aldehydic C–H alkynylation

A 20 mL Schlenk tube equipped with a magnetic stir bar was charged with eosin Y (0.008 mmol, 5.2 mg), aldehyde **1** (0.4 mmol), acetylenic sulfone reagents **13** (0.2 mmol). Then, 2.0 mL of anhydrous *tert*-butanol was added. The Schlenk tube was connected to Schlenk line and freeze–pump–thaw was performed for three times to completely remove air inside the reaction mixture. Eventually the Schlenk tube was refilled with an atmosphere of argon at room temperature and sealed. The reaction vessel was surrounded by a coil of blue LED strip (2 m, 18 W). Then the reaction tubes were placed in a water bath covered by top oil layer (to prevent evaporation of water bath). The reaction was running at 60 ^o^C and stopped after 24 h. The solvent was removed under reduced pressure and the crude mixture was purified by silica gel column chromatography or prepared TLC (eluent: hexane/diethyl ether or hexane/ethyl acetate; 10/1–3/1) to give the corresponding product **14**.

#### Computational details

Density functional theory calculations were performed to shed light on the mechanism of eosin Y regeneration (Supplementary Fig. 17). RHAT (red line) is the favored pathway, which features a barrier 2.1 kcal/mol lower than an alternative single electron transfer (SET, black line). The geometries optimization in this study was performed at the uB3LYP density functional with a standard def2-SVP basis set. The nature of the stationary points (minima with no imaginary frequency or transition states with one imaginary frequency) were confirmed. The free energies of the optimized geometries were calculated at the same level of theory, considering the solvent effect of acetone using an SMD continuum solvation model. Unless specified otherwise, the Gibbs free energy was used throughout. For transition state, intrinsic reaction coordinate calculations were performed to verify whether it connected with correct reactants and products or intermediates. All calculations were performed using the Gaussian 16 Rev. A.03 software suite^60^.

## Supplementary information

Supplementary Information

## Data Availability

The authors declare that all other data supporting the findings of this study are available within the article and Supplementary Information files, and also are available from the corresponding author upon request. Source data are provided with this paper.

## Source data

Source Data
