# Supplementary material for: Divergent functionalization of aldehydes photocatalyzed by neutral eosin Y with sulfone reagents
Source: Nat Commun. 2021 Dec 10;12:7214. doi: 10.1038/s41467-021-27550-8 (PMC8664905; doi:10.1038/s41467-021-27550-8)
Supplement: Supplementary file 1 — Supplementary Information [file 41467_2021_27550_MOESM1_ESM.pdf]

Supplementary Information

Divergent Functionalization of Aldehydes Photocatalyzed by Neutral Eosin Y with Sulfone Reagents

Jianming Yan[1,2,*], Haidi Tang[1,3,*], Eugene Jun Rong Kuek[1], Xiangcheng Shi[1], Chenguang Liu[1], Muliang Zhang[1], Jared L. Piper[4], Shengquan Duan[4] & Jie Wu[1,3]

[1]Department of Chemistry, National University of Singapore, 3 Science Drive 3, Singapore 117543, Republic of Singapore

[2]Department of Medicinal Chemistry, College of Pharmacy, Chongqing Medical University, Chongqing 400016, China

[3]National University of Singapore (Suzhou) Research Institute, 377 Lin Quan Street, Suzhou Industrial Park, Suzhou, Jiangsu, 215123, China

[4]Pfizer Worldwide Research and Development, Eastern Point Rd, Groton, CT 06340, USA

*These authors contributed equally to this work.

Correspondence and requests for materials should be addressed to J.W. (email: chmjie@nus.edu.sg), M.Z. (email: muliang0206@foxmail.com), S.D. (email: shengquan.duan@pfizer.com).

Table of contents

Supplementary Methods S2
I. General information S2
II. Bioactive molecules containing fluoromethylthio (SCH_2F) moiety S2
III. Preparation of starting materials S3
IV. Optimization for aldehydic C–H fluoromethylthiolation and alkynylation S7
V. General procedures for eosin Y-photocatalyzed aldehydic C–H functionalization with sulfone reagents S10
VI. Mechanistic studies S31
- Evaluation of different photo HAT catalysis systems S31
- Study on site-selectivity of aldehydic C–H fluoromethylthiolation S32
- Control experiments to elucidate the reaction intermediates in arylthiolation S33
- Evidence for the involvement of arylsulfonyl radical and acyl radical in arylthiolation S36
- ^{18}O-labelling study S38
- Discussion on mechanism of eosin Y-photocatalytic aldehydic C–H arylthiolation S39
- Study on electrochemical potentials of sulfone derivatives S42
- Determination of the quantum yield by standard ferrioxalate actinometry S44
- Light on/off experiments over time S45
- DFT calculation on SET and RHAT pathways for eosin Y regeneration S46

VII. Scale up in flow reactors S47
- Flow reactor setup S48
- Aldehydic C–H fluoromethylthiolation in flow S48

VIII. NMR spectra S49
Supplementary References S159

Supplementary Methods

I. General information

Unless otherwise noted, all chemicals and anhydrous solvents were purchased from commercial suppliers (Sigma Aldrich, TCI, Oakwood, BLDpharm) and used as received. Commercial unavailable substrates were synthesized according to literature. ^{1}H NMR, ^{13}C NMR spectra were recorded on a Bruker AV-III400 (400 MHZ) or AMX500 (500 MHz) spectrometer. Chemical shifts were calibrated using residual undeuterated solvent as an internal reference ($CDCl_3$: 7.26 ppm ^{1}H NMR, 77.16 ppm ^{13}C NMR). Multiplicity was indicated as follows: s (singlet), d (doublet), t (triplet), q (quartet), m (multiplet), dd (doublet of doublet), brs (broad singlet). High-resolution mass spectra (HRMS) were obtained on a Finnigan/MAT 95XL-T spectrometer. GC analysis was performed on Aglilent 7820A & 5977E GC-MS. HPLC analysis was performed using the corresponding commercial chiral columns (chiralcel IA, IC, ID, IE IF and OJ-H columns) as stated in the experimental procedures at 30 °C with the UV detector at 254 nm. Cyclic voltammograms (CV) were collected using a VersaSTAT 3 Potentiostat Galvanostat from Princeton Applied Research. Blue LED strips (2 meters, 18 W) were purchased from Inwares Pte Ltd (Singapore). The Kessil PR160 series (λ_{max} = 440 nm, 40 W) were used as the blue LED light source for the continuous-flow reactions. All catalytic reactions were carried out in Schlenk tube (20 mL) under an argon atmosphere with magnetic stirring after repeated freeze-pump-thaw. The isolated yield was the purified state by flash chromatography over silica gel.

II. Bioactive molecules containing fluoromethylthio (SCH_2F) moiety

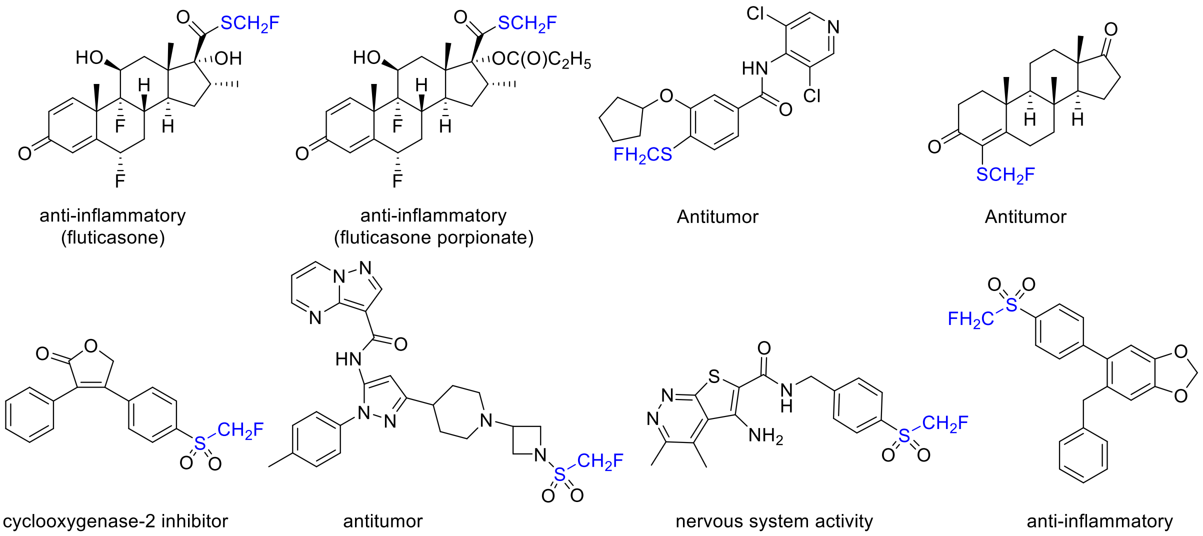

Supplementary Figure 1. Bioactive compounds featuring a fluoromethylthio (SCH_2F) moiety.

III. Preparation of starting materials

Commercially unavailable aldehydes (**S-3q**,[1] **S-3u**,[2] **S-3v,**[2] **S-3w**,[3] **S-14j**,[4] **S-14o**[2]), sulfinic acids **5**,[5,6] *S*-trifluoromethyl benzenesulfonothioate **2c**[7] ($PhSO_2$–SCF_3), disulfone **10** [8] ($PhSO_2$–SO_2Ph) and triflic azide **15**[9] were prepared according to reported procedures. A range of alkynyl sulfones **13** were prepared according to the literature.[10] Unless newly characterized herein, the spectra data of these substrates are in accordance with the corresponding reported data in the literature.

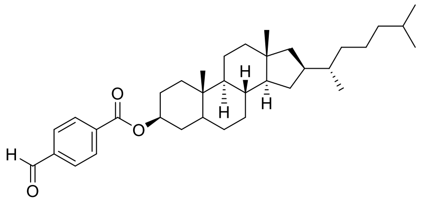

(3*S*,8*S*,9*S*,10*S*,13*R*,14*S*,16*S*)-10,13-Dimethyl-16-((*S*)-6-methylheptan-2-yl)hexadecahydro-1*H*-cyclopenta[a]phenanthren-3-yl 4-formylbenzoate (S-14o): Following the literature procedure,[2] the title compound **S-14o** (3656 mg) was obtained in 78% yield starting from dihydrocholesterol (3498 mg, 9.0 mmol) and 4-formyl benzoic acid (1351 mg, 9.0 mmol).^{1}H NMR (400 MHz, $CDCl_3$) δ 10.10 (s, 1H), 8.19 (d, *J* = 8.3 Hz, 2H), 7.94 (d, *J* = 8.3 Hz, 2H), 4.97 (tt, *J* = 11.4, 4.9 Hz, 1H), 2.00 – 1.94 (m, 2H), 1.82 – 1.67 (m, 5H), 1.59 – 1.52 (m, 6H), 1.35 – 1.22 (m, 9H), 1.16 – 0.97 (m, 9H), 0.91 (d, *J* = 6.5 Hz, 3H), 0.88 (s, 3H), 0.87 (d, *J* = 2.3 Hz, 3H), 0.86 (d, *J* = 2.3 Hz, 3H), 0.66 (s, 3H); ^{13}C NMR (126 MHz, $CDCl_3$) δ 191.74, 165.10, 138.99, 135.99, 130.14, 129.45, 75.23, 56.43, 56.29, 54.24, 44.72, 42.62, 40.00, 39.53, 36.78, 36.18, 35.82, 35.53, 35.51, 34.08, 32.01, 28.65, 28.26, 28.03, 27.55, 24.23, 23.85, 22.83, 22.58, 21.25, 18.59, 12.31, 12.09. HRMS ESI $[M+H]^+$ calculated for $C_{35}H_{53}O_3$ 521.3989, found 521.3981.

Di-aldehydic substrate 4-(3-oxopropyl)benzaldehyde **S1** was prepared according to the following procedure:

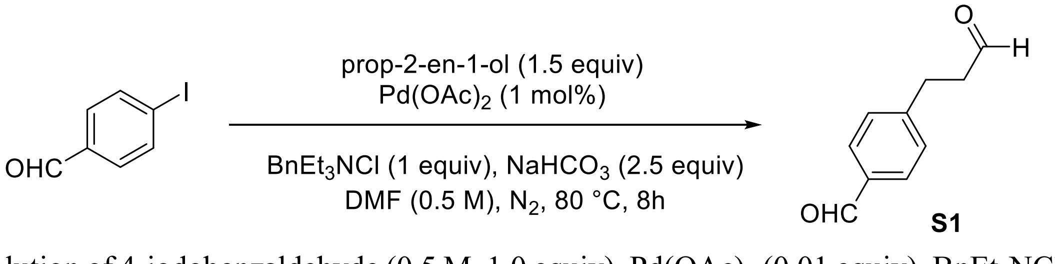

To a solution of 4-iodobenzaldehyde (0.5 M, 1.0 equiv), $Pd(OAc)_2$ (0.01 equiv), $BnEt_3NCl$ (1.0 equiv) and $NaHCO_3$ (2.5 equiv) in DMF, prop-2-en-1-ol (1.5 equiv.) was added under N_2 atmosphere. The reaction was stirred at 80 °C for 8 h. The reaction mixture was then cooled to room temperature, diluted with EtOAc, washed with sat. aq. NH_4Cl and brine, dried with Na_2SO_4 and concentrated under reduced pressure, purified by column chromatography on silica gel to deliver 4-(3-oxopropyl)benzaldehyde **S1**. The spectra data of **S1** is in accordance the literature.[11]

Sulfinic acids **5** were synthesized according to the following known procedure. [5,6]

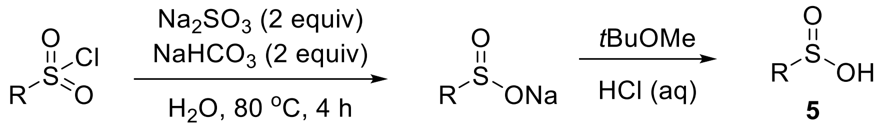

Step 1: Sodium sulfite (20 mmol, 2 eq.), sodium bicarbonate (20 mmol, 2 eq.) and the corresponding aryl sulfonyl chloride (10 mmol, 1 eq.) were dissolved in distilled water (10 mL). The reaction mixture was stirred for 4 h at 80 °C. After cooling down to room temperature, water was removed by rotary evaporator. 25 mL of ethanol was then added to this white residue and the resulting heterogeneous solution was filtered. The filtrate was concentrated under reduced pressure and the desired sodium aryl sulfinates were obtained as white crystalline powders.

Step 2: Corresponding sodium sulfonate (5 mmol) was dissolved in 3 mL of water, then tert-butyl methyl ether (1.5 mL) was added followed by the slow addition of 0.5 mL of concentrated aqueous hydrochloric acid (HCl) over 2 min. The mixture was stirred for an additional 10 min, transferred to a separatory funnel, and the aqueous layer was removed. The organic layer was concentrated on a rotary evaporator, and sulfinic acids **5** were obtained.
NMR spectra of the known compounds (**5a**, **5b**, **5q**, **5r**, **5t**, **5u)** are in line with literature. [5,6]
Sulfinic acid **5s** is newly characterized.

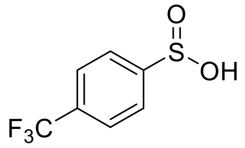

4-(Trifluoromethyl)benzenesulfinic acid (5s): ^{1}H NMR (500 MHz, DMSO-d_6) δ 7.95 (d, J = 8.2 Hz, 2H), 7.89 (d, J = 8.2 Hz, 2H); ^{13}C NMR (126 MHz, DMSO-d_6) δ 153.53, 131.85 (q, $^{2}J_{C\text{-}F}$ = 8.2 Hz), 126.55 (q, $^{3}J_{C\text{-}F}$ = 3.8 Hz), 126.15, 124.25 (q, $^{1}J_{C\text{-}F}$ = 272.2 Hz). ^{19}F NMR (377 MHz, $CDCl_3$) δ: -61.31 (s).

Acetylenic sulfones **13** were synthesized according to the reported protocols.[10]

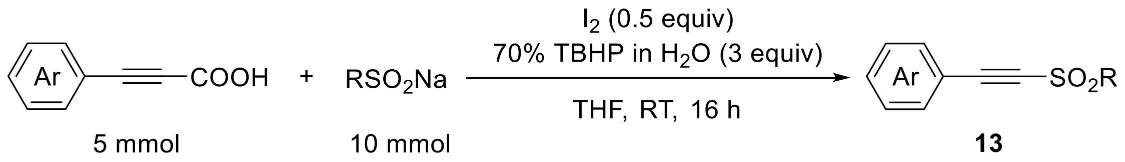

Procedure: To a solution of arylacetylenic acid (5 mmol) with sodium sulfinate (10 mmol) and iodine (2.5 mmol) in THF (20 mL) was added TBHP (70% in water, 15 mmol). The resulting mixture was stirred at room temperature for 16 h. The reaction mixture was quenched by the addition of saturated aqueous $Na_2S_2O_3$ (20 mL). Further stirring was followed by extraction with EtOAc (2 × 30 mL). The combined organic extracts were washed with H_2O (30 mL) and brine (30 mL), dried over $MgSO_4$, filtered, and concentrated (aspirator). The residue was purified by column chromatography using EtOAc/hexanes as eluent to afford the

corresponding product.
The spectra of the known compound **13aa-13af, 13p-13r and 13u** match the previous report.[10] Acetylenic sulfones **13s**, **13t** and **13v** are newly synthesized.

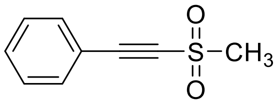

((Methylsulfonyl)ethynyl)benzene (13aa): ^{1}H NMR (500 MHz, $CDCl_3$) δ 7.60 – 7.58 (m, 2H), 7.54 – 7.50 (m, 1H), 7.44 – 7.40 (m, 2H), 3.30 (s, 3H); ^{13}C NMR (100 MHz, $CDCl_3$) δ 132.89, 131.81, 128.85, 117.52, 91.57, 84.46, 46.85.

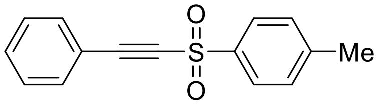

1-Methyl-4-((phenylethynyl)sulfonyl)benzene (13ab): ^{1}H NMR (500 MHz, $CDCl_3$) δ 7.97-7.95 (m, 2H), 7.53 - 7.51 (m, 2H), 7.49 - 7.45 (m, 1H), 7.40 - 7.35 (m, 4H), 2.47 (s, 3H); ^{13}C NMR (126 MHz, $CDCl_3$) δ 145.38, 138.97, 132.74, 131.46, 130.01, 128.67, 127.53, 118.05, 92.97, 85.62, 21.76.

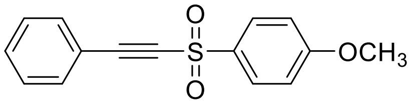

1-Methoxy-4-((phenylethynyl)sulfonyl)benzene (13ac): ^{1}H NMR (500 MHz, $CDCl_3$) δ 8.01 – 7.99 (m, 2H), 7.52 - 7.50 (m, 2H), 7.47 - 7.44 (m, 1H), 7.38 - 7.34 (m, 2H), 7.05 – 7.03 (m, 2H), 3.90 (s, 3H); ^{13}C NMR (126 MHz, $CDCl_3$) δ 164.22, 133.44, 132.69, 131.39, 129.86, 128.66, 118.12, 114.61, 92.52, 85.88, 55.79.

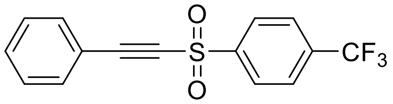

1-((Phenylethynyl)sulfonyl)-4-(trifluoromethyl)benzene (13ae): ^{1}H NMR (500 MHz, $CDCl_3$) δ 8.22 (d, J = 8.2 Hz, 2H), 7.87 (d, J = 8.2 Hz, 2H), 7.56 - 7.49 (m, 3H), 7.41 - 7.38 (m, 2H); ^{13}C NMR (126 MHz, $CDCl_3$) δ 145.17, 135.76 (q, $^{2}J_{C–F}$ = 33.2 Hz), 132.90, 131.98, 128.81, 128.04, 126.60 (q, $^{3}J_{C–F}$ = 3.7 Hz), 123.07 (q, $^{1}J_{C–F}$ = 273.5 Hz), 117.43, 94.98, 84.64; ^{19}F NMR (377 MHz, $CDCl_3$) δ -63.24 (s).

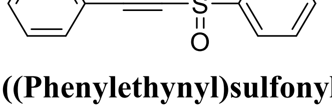

((Phenylethynyl)sulfonyl)benzene (13af): ^{1}H NMR (400 MHz, $CDCl_3$) δ 8.10 - 8.07 (m, 2H), 7.72 - 7.67 (m, 1H), 7.63 - 7.58 (m, 2H), 7.55 - 7.52 (m, 2H), 7.50 - 7.46 (m, 1H), 7.40 - 7.35 (m, 2H); ^{13}C NMR (126 MHz, $CDCl_3$) δ 141.84, 134.19, 132.78, 131.61, 129.41, 128.72, 127.42, 117.89, 93.53, 85.36.

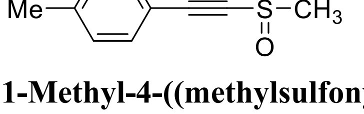

1-Methyl-4-((methylsulfonyl)ethynyl)benzene (13p): ^{1}H NMR (500 MHz, $CDCl_3$) δ 7.48 (d, J = 8.2 Hz, 2H), 7.22 (d, J = 7.9 Hz, 2H), 3.29 (s, 3H), 2.40 (s, 3H); ^{13}C NMR (126 MHz,

CDCl3) δ 142.69, 132.85, 129.61, 114.38, 92.28, 84.10, 46.87, 21.83.

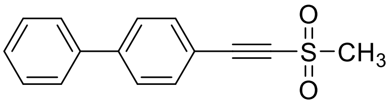

4-((Methylsulfonyl)ethynyl)-1,1'-biphenyl (13q): ^{1}H NMR (500 MHz, $CDCl_3$) δ 7.67 – 7.63 (m, 4H), 7.61 - 7.59 (m, 2H), 7.49 - 7.46 (m, 2H), 7.43 - 7.39 (m, 1H), 3.32 (s, 3H); ^{13}C NMR (126 MHz, $CDCl_3$) δ 144.60, 139.42, 133.39, 129.09, 128.50, 127.46, 127.19, 116.12, 91.74, 85.02, 46.89.

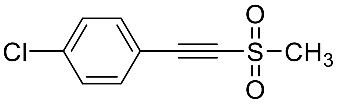

1-Chloro-4-((methylsulfonyl)ethynyl)benzene (13r): ^{1}H NMR (400 MHz, $CDCl_3$) δ 7.54 (d, *J* = 8.8 Hz, 2H), 7.41 (d, *J* = 8.8 Hz, 2H), 3.30 (s, 3H); ^{13}C NMR (126 MHz, $CDCl_3$) δ 138.41, 134.07, 129.38, 115.98, 90.19, 85.29, 46.78.

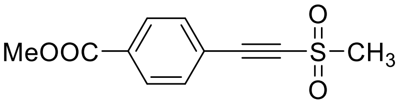

Methyl 4-((methylsulfonyl)ethynyl)benzoate (13s): ^{1}H NMR (500 MHz, $CDCl_3$) δ 8.10 – 8.08 (m, 2H), 7.68 – 7.66 (m, 2H), 3.95 (s, 3H), 3.32 (s, 3H); ^{13}C NMR (126 MHz, $CDCl_3$) δ 165.78, 132.83, 132.79, 129.82, 121.87, 89.86, 86.34, 52.61, 46.78.

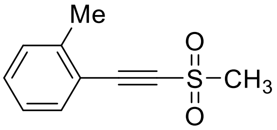

1-Methyl-2-((methylsulfonyl)ethynyl)benzene (13t): ^{1}H NMR (400 MHz, $CDCl_3$) δ 7.54 (dd, *J*= 7.7 Hz, 1.4 Hz, 1H), 7.40 (td, *J*= 7.6, 1.4 Hz, 1H), 7.29 – 7.20 (m, 2H), 3.27 (s, 3H), 2.36 (s, 3H); ^{13}C NMR (126 MHz, $CDCl_3$) δ 142.52, 133.30, 131.79, 130.05, 126.05, 117.38, 91.07, 88.07, 47.01, 20.49. HRMS (EI) calculated for $C_{10}H_{10}O_2S$ $[M]^+$ 194.0396, found 194.0392.

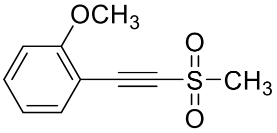

1-Methoxy-2-((methylsulfonyl)ethynyl)benzene (13u): ^{1}H NMR (400 MHz, $CDCl_3$) δ 7.50 – 7.44 (m, 2H), 6.98 – 6.91 (m, 2H), 3.88 (s, 3H), 3.30 (s, 3H); ^{13}C NMR (126 MHz, $CDCl_3$) δ 161.86, 134.64, 133.55, 120.67, 111.02, 106.79, 89.50, 88.05, 55.89, 47.01.

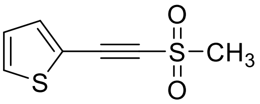

2-((Methylsulfonyl)ethynyl)thiophene (13v): ^{1}H NMR (500 MHz, $CDCl_3$) δ 7.82 – 7.81 (m, 1H), 7.36 – 7.35 (m, 1H), 7.22 – 7.20 (m, 1H), 3.27 (s, 3H); ^{13}C NMR (126 MHz, $CDCl_3$) δ 135.17, 129.79, 126.87, 116.66, 87.32 84.46, 46.88. HRMS (EI) calculated for $C_7H_6O_2S_2$ $[M]^+$ 185.9804, found 185.9797.

IV. Optimization for aldehydic C–H fluoromethylthiolation and alkynylation

Typical reaction setup

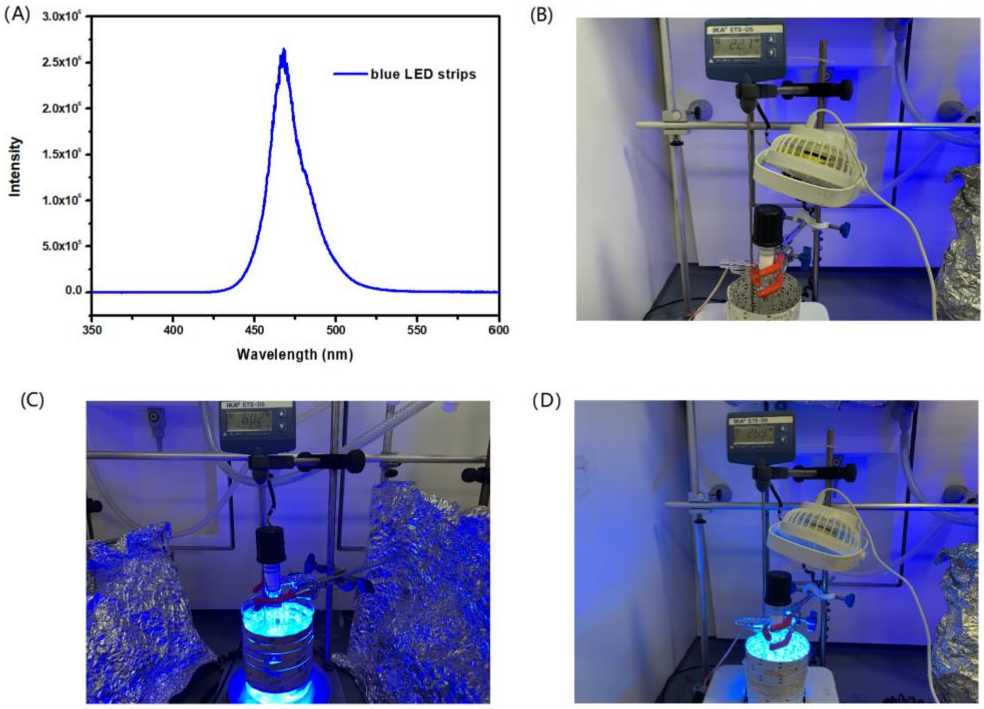

Supplementary Figure 2. The setup for photo-mediated reactions.
(A) Emission spectra of the blue LED light source.
(B) Reaction setup using a fan for cooling.
(C) Reaction setup under 60 ^{o}C.
(D) Reaction setup at room temperature.

Supplementary Table 1. Selected optimization results for aldehydic C–H fluoromethylthiolation.

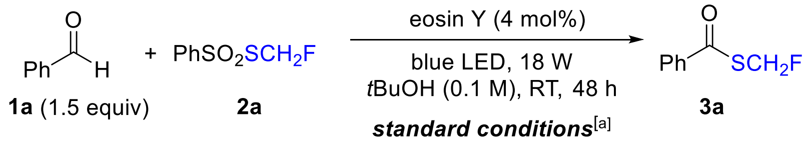

entry	deviation from standard conditions	conv. of **2a** (%)[b]	yield of **3a** (%)[b]
1	none	100	91(88)
2	shorter reaction time of 24 h	78	70
3	Na_2eosin Y (4 mol%) as the catalyst	10	0
4	Mes-$Arc^+ClO_4^-$ (2 mol%)+HCl (5 mol%)	60	17
5	$Ir(dF(CF_3)ppy)_2(dtbbpy)]PF_6$ (2 mol%) + $PhCO_2Na$ (5 mol%)	100	60
6[c]	TBADT (4 mol%) as the catalyst	100	50
7	2.5 equiv of **1a**	100	91
8	*t*-Amyl acohol instead of *t*BuOH	100	85
9	acetone instead of *t*BuOH	100	53
10	CH_3CN instead of *t*BuOH	100	80
11	ethyl acetate instead of *t*BuOH	26	83
12	in darkness without blue LED	0	0

[a]Standard conditions: **1a** (0.3 mmol), **2a** (0.2 mmol), eosin Y (4 mol%), and *t*BuOH (2.0 mL) in a Schlenk tube (20 mL) at room temperature (~ 27 °C) under blue LED (18 W) irradiation. Freeze-pump-thaw was repeated 3 times to remove air and refill argon into the reaction vessel.

[b]Conversions were determined by analysis of the crude ^1H NMR using CH_2Br_2 as an internal standard; an isolated yield is shown in parentheses.

[c]The reaction was performed under 365 nm LED irradiation.

Supplementary Table 2. Selected optimization results for aldehydic C–H alkynylation.

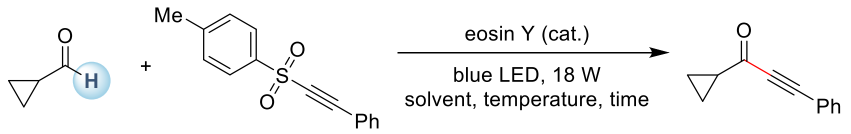

S-14l 13ab 14l

Entry	Solvent	T (°C)	Time (h)	Conv. (%) of **13ab**	yield (%)[a]
1	MeCN (0.1 M)	60	24	82	17
2	Acetone (0.1 M)	60	24	90	-
3	DCM (0.1 M)	60	24	63	34
4	DCE (0.1 M)	60	24	70	55
5	DMSO (0.1 M)	60	24	>99	10
6	Toluene (0.1 M)	60	24	53	16
7	DMF (0.1 M)	60	24	>99	24
8	PhF (0.1 M)	60	24	43	33
11	EtOAc (0.1 M)	60	24	74	65
**12**	***t*BuOH (0.1 M)**	**60**	**24**	**93**	**74**
13	*t*BuOH/EtOAc (1:1, 0.1 M)	60	24	94	58
14	*t*BuOH (0.1 M)	80	24	>99	56
15	*t*BuOH (0.1 M)	43	24	71	40
16	*t*BuOH (0.1 M)	43	36	86	53
17	*t*BuOH (0.05 M)	60	24	87	72
18	*t*BuOH (0.2 M)	60	24	81	64
19[b]	*t*BuOH (0.1 M)	60	24	82	60
20[c]	*t*BuOH (0.1 M)	60	24	90	72

General reaction conditions: **S-14l** (0.2 mmol), **13ab** (0.1 mmol), eosin Y (4.0 mol%) in solvent (0.1 M) under argon with 18 W blue LED irradiation, unless otherwise noted.
[a] Yield based analysis of crude ^{1}H NMR spectra using CH_2Br_2 as an internal standard.
[b] Eosin Y (2.0 mol%) was used.
[c] Eosin Y (8.0 mol%) was used..

Supplementary Table 3. Evaluation of different acetylenic sulfone **13** for aldehydic C–H alkynylation.

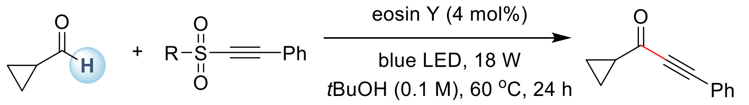

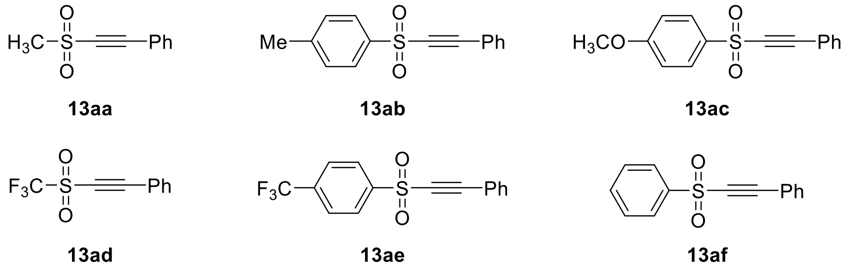

Entry	Sulfone	Conversion (%) of **13**	NMR Yield (%)[a]
**1**	**13aa**	**92**	**84**
2	**13ab**	99	63
3	**13ac**	94	44
4	**13ad**	99	32
5	**13ae**	74	41
6	**13af**	90	56

General reaction conditions:**S-14l** (0.4 mmol), **13** (0.2 mmol), eosin Y (4.0 mol%) in *t*BuOH (0.1 M) under argon with 18 W blue LED irradiation for 24 h.
[a] Yield was determined by the crude ^{1}H NMR spectra using CH_2Br_2 as an internal standard.

V. General procedures for eosin Y-photocatalyzed aldehydic C–H functionalization with sulfone reagents

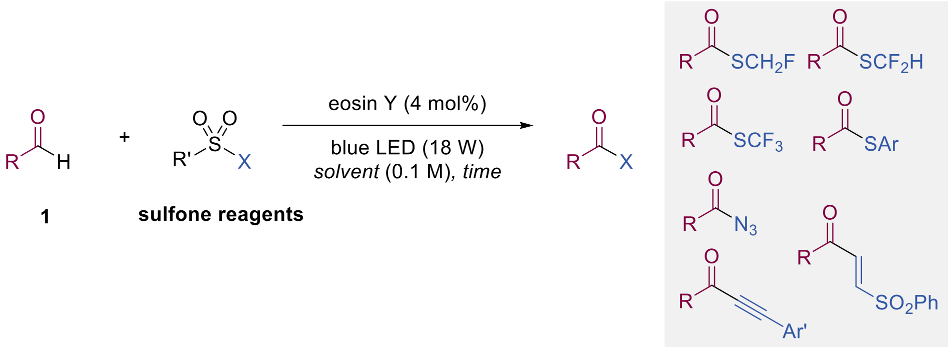

General procedure I: neutral-eosin Y-photocatalyzed aldehydic C–H fluoromethylthiolation

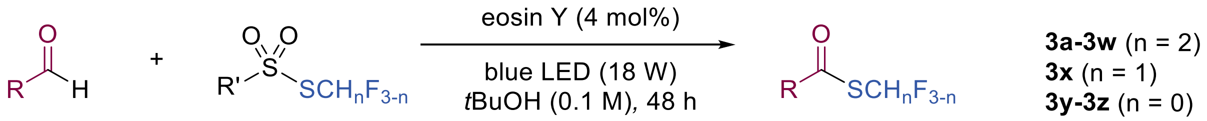

1 2 (n = 2, 1, 0) 3 (n = 2, 1, 0)

A 20 mL Schlenk tube equipped with a magnetic stir bar was charged with eosin Y (0.008 mmol, 5.2 mg), aldehyde **1** (0.3 mmol), and fluoromethylthiolation reagents **2** (0.2 mmol). Then, 2.0 mL of anhydrous *tert*-butanol was added. The Schlenk tube was connected to Schlenk line and freeze-pump-thaw was performed for three times to completely remove air inside the reaction mixture. Eventually the Schlenk tube was refilled with an atmosphere of argon at room temperature and sealed. The reaction vessel was surrounded by a coil of blue LED strip (2 meters, 18 W). Then the reaction was running at ambient temperature (~27 ^{o}C) using a fan to cool down the reaction mixture and stopped after 48 h. The solvent was removed under reduced pressure and the crude mixture was purified by silica gel column chromatography or prepared TLC (eluent: hexane/diethyl ether or hexane/ethyl acetate; 10/1 – 3/1) to give the corresponding product **3**. Note that the workup procedure was performed under weak vacuum (≥ 50 mbar) and low temperature (≤ 30 ^{o}C) due to volatility of the corresponding products **3**.

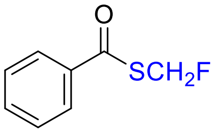

***S*-(fluoromethyl) benzothioate (3a)**.[12] Following the general procedure I, the title compound (30.0 mg) was obtained in 88% yield. Colorless oil. ^{1}H NMR (400 MHz, $CDCl_3$) δ 8.05 – 7.94 (m, 2H), 7.67 – 7.60 (m, 1H), 7.56 – 7.43 (m, 2H), 6.01 (d, *J* = 50.2 Hz, 2H). ^{13}C NMR (126 MHz, $CDCl_3$) δ 188.17, 136.08, 134.45, 129.04, 127.94, 80.82 (d, *J* = 215.5 Hz). ^{19}F NMR (377 MHz, $CDCl_3$) δ -192.10. HRMS ESI $[M+H]^+$ calculated for C_8H_8FOS 171.0275, found 171.0279.

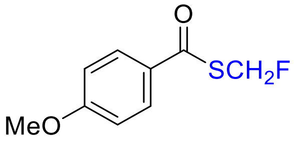

***S*-(fluoromethyl) 4-methoxybenzothioate (3b)**.[12] Following the general procedure I, the title compound (28.0 mg) was obtained in 70% yield. ^{1}H NMR (400 MHz, $CDCl_3$) δ 7.97 (d, *J* = 9.0 Hz, 2H), 6.96 (d, *J* = 9.0 Hz, 2H), 5.99 (d, *J* = 50.4 Hz, 2H), 3.88 (s, 3H). ^{13}C NMR (126 MHz, $CDCl_3$) δ 186.36, 164.63, 130.25, 128.85, 114.22, 80.90 (d, *J* = 214.2 Hz), 55.75. ^{19}F NMR (377 MHz, $CDCl_3$) δ -191.32. HRMS ESI $[M+H]^+$ calculated for $C_9H_{10}FO_2S$ 201.0380, found 201.0375.

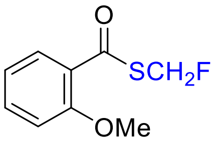

***S*-(fluoromethyl) 2-methoxybenzothioate (3c)**. Following the general procedure I, the title compound (24.4 mg) was obtained in 61% yield. ^{1}H NMR (400 MHz, $CDCl_3$) δ 7.87 (dd, *J* = 7.8, 1.8 Hz, 1H), 7.53 (ddd, *J* = 8.4, 7.4, 1.8 Hz, 1H), 7.09 – 6.97 (m, 2H), 5.95 (d, *J* = 50.3 Hz, 2H), 3.95 (s, 3H). ^{13}C NMR (126 MHz, $CDCl_3$) δ 186.91, 158.87, 134.98, 130.46, 125.45, 120.81, 112.30, 81.33 (d, *J* = 213.4 Hz), 55.95. ^{19}F NMR (377 MHz, $CDCl_3$) δ -192.93. HRMS ESI $[M+H]^+$ calculated for $C_9H_{10}FO_2S$ 201.0380, found 201.0373.

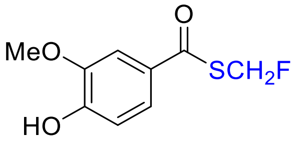

***S*-(fluoromethyl) 4-hydroxy-3-methoxybenzothioate (3d)**. Following the general procedure I, the title compound (28.1 mg) was obtained in 65% yield. ^{1}H NMR (400 MHz, $CDCl_3$) δ 7.63 (dd, *J* = 8.4, 2.0 Hz, 1H), 7.49 (d, *J* = 2.0 Hz, 1H), 6.98 (d, *J* = 8.4 Hz, 1H), 6.15 (s, 1H), 5.99 (d, *J* = 50.4 Hz, 2H), 3.96 (s, 3H). ^{13}C NMR (126 MHz, $CDCl_3$) δ 186.51, 151.52, 146.79, 128.67, 123.45, 114.46, 109.62, 80.95 (d, *J* = 215.5 Hz), 56.33. ^{19}F NMR (377 MHz, $CDCl_3$) δ -191.28. HRMS (EI) calculated for $C_9H_9FO_3S$ $[M]^+$ 216.0251, found 216.0255.

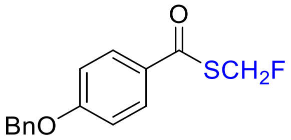

***S*-(fluoromethyl) 4-(benzyloxy)benzothioate (3e)**. Following the general procedure I, the title compound (43.1 mg) was obtained in 78% yield. ^{1}H NMR (400 MHz, $CDCl_3$) δ 7.97 (d, *J* = 9.0 Hz, 2H), 7.60 – 7.32 (m, 5H), 7.04 (d, *J* = 9.0 Hz, 2H), 5.99 (d, *J* = 50.3 Hz, 2H), 5.15 (s, 2H). ^{13}C NMR (126 MHz, $CDCl_3$) δ 186.37, 163.76, 136.03, 130.27, 129.04, 128.89, 128.51, 127.63, 115.06, 80.90 (d, *J* = 215.5 Hz), 70.44. ^{19}F NMR (377 MHz, $CDCl_3$) δ -191.35. HRMS (EI) calculated for $C_{15}H_{13}FO_2S$ $[M]^+$ 276.0615, found 276.0622.

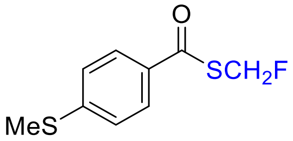

***S*-(fluoromethyl) 4-(methylthio)benzothioate (3f)**. Following the general procedure I, the title compound (32.8 mg) was obtained in 76% yield. ^{1}H NMR (400 MHz, $CDCl_3$) δ 7.89 (d, *J* = 8.7 Hz, 2H), 7.28 (d, *J* = 8.7 Hz, 2H), 5.99 (d, *J* = 50.3 Hz, 2H), 2.53 (s, 3H). ^{13}C NMR (126 MHz, $CDCl_3$) δ 186.95, 147.94, 132.14, 128.24, 125.17, 80.82 (d, *J* = 215.5 Hz), 14.84. ^{19}F NMR (377 MHz, $CDCl_3$) δ -191.70. HRMS (EI) calculated for $C_9H_9FOS_2$ $[M]^+$ 216.0073, found 216.0074

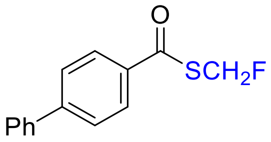

***S*-(fluoromethyl) [1,1'-biphenyl]-4-carbothioate (3g)**. Following the general procedure I, the title compound (27.1 mg) was obtained in 55% yield. ^{1}H NMR (400 MHz, $CDCl_3$) δ 7.99 (d, *J* = 8.7 Hz, 2H), 7.64 (d, *J* = 8.7 Hz, 2H), 7.59 – 7.52 (m, 2H), 7.45 – 7.39 (m, 2H), 7.38 – 7.31 (m, 1H), 5.96 (d, *J* = 50.2 Hz, 2H). ^{13}C NMR (126 MHz, $CDCl_3$) δ 187.95, 147.24, 139.66,

134.73, 129.19, 128.68, 128.52, 127.64, 127.46, 80.85 (d, J = 215.5 Hz). ^{19}F NMR (377 MHz, $CDCl_3$) δ -191.90. HRMS (EI) calculated for $C_{14}H_{11}FOS$ $[M]^+$ 246.0509, found 246.0511.

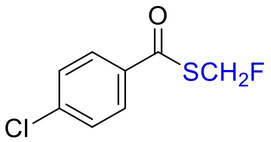

***S*-(fluoromethyl) 4-chlorobenzothioate (3h)**.[12] Following the general procedure I, the title compound (27.8 mg) was obtained in 68% yield. ^{1}H NMR (400 MHz, $CDCl_3$) δ 7.93 (d, J = 8.9 Hz, 2H), 7.48 (d, J = 8.8 Hz, 2H), 6.00 (d, J = 50.0 Hz, 2H). ^{13}C NMR (126 MHz, $CDCl_3$) δ 187.07, 140.99, 134.39, 129.40, 129.23, 80.74 (d, J = 216.7 Hz). ^{19}F NMR (377 MHz, $CDCl_3$) δ -192.27. HRMS ESI $[M+H]^+$ calculated for C_8H_7ClFOS 204.9885, found 204.9881.

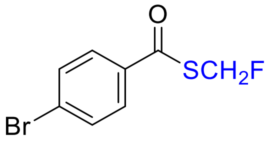

***S*-(fluoromethyl) 4-bromobenzothioate (3i)**.[12] Following the general procedure I, the title compound (37.4 mg) was obtained in 75% yield. ^{1}H NMR (400 MHz, $CDCl_3$) δ 7.85 (d, J = 8.7 Hz, 2H), 7.64 (d, J = 8.7 Hz, 2H), 5.99 (d, J = 50.1 Hz, 2H). ^{3}C NMR (126 MHz, $CDCl_3$) δ 187.29, 134.81, 132.39, 129.70, 129.29, 80.71 (d, J = 216.5 Hz). ^{19}F NMR (377 MHz, $CDCl_3$) δ -192.32. HRMS ESI $[M+H]^+$ calculated for C_8H_7BrFOS 248.9380, found 248.9378.

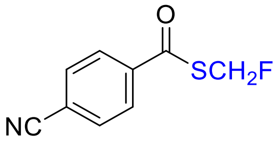

***S*-(fluoromethyl) 4-cyanobenzothioate (3j)**.[12] Following the general procedure I, the title compound (25.4 mg) was obtained in 65% yield. ^{1}H NMR (400 MHz, $CDCl_3$) δ 8.08 (d, J = 8.7 Hz, 2H), 7.81 (d, J = 8.7 Hz, 2H), 6.01 (d, J = 49.8 Hz, 2H). ^{13}C NMR (126 MHz, $CDCl_3$) δ 187.26, 139.10, 132.90 (2C), 128.30 (2C), 117.69 (2C), 80.60 (d, J = 217.9 Hz). ^{19}F NMR (377 MHz, $CDCl_3$) δ -192.93. HRMS ESI $[M+H]^+$ calculated for C_9H_7FNOS 196.0227, found 196.0225.

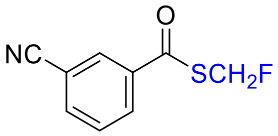

***S*-(fluoromethyl) 3-cyanobenzothioate (3k)**. Following the general procedure I, the title compound (28.5 mg) was obtained in 73% yield. ^{1}H NMR (400 MHz, $CDCl_3$) δ 8.26 (td, J = 1.7, 0.5 Hz, 1H), 8.20 (ddd, J = 7.9, 1.8, 1.2 Hz, 1H), 7.96 – 7.88 (m, 1H), 7.66 (td, J = 8.0, 0.5 Hz, 1H), 6.02 (d, J = 49.8 Hz, 2H). ^{13}C NMR (126 MHz, $CDCl_3$) δ 186.70, 137.18, 136.96, 131.72, 131.42, 130.14, 117.57, 113.77, 80.58 (d, J = 218.80 Hz). ^{19}F NMR (377 MHz, $CDCl_3$) δ -192.79. HRMS ESI $[M+H]^+$ calculated for C_9H_7FNOS 196.0227, found 196.0221.

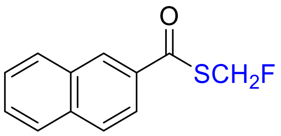

***S*-(fluoromethyl) naphthalene-2-carbothioate (3l)**.[12] Following the general procedure I, the title compound (31.7 mg) was obtained in 72% yield. ^{1}H NMR (400 MHz, $CDCl_3$) δ 8.55 (d, *J* = 1.3 Hz, 1H), 8.05 – 7.96 (m, 2H), 7.95 – 7.85 (m, 2H), 7.69 – 7.51 (m, 2H), 6.06 (d, *J* = 50.2 Hz, 2H). ^{13}C NMR (126 MHz, $CDCl_3$) δ 188.00, 136.26, 133.33, 132.48, 129.82 (2C), 129.17, 128.96, 128.01, 127.32, 123.24, 80.91 (d, *J* = 215.5 Hz). ^{19}F NMR (377 MHz, $CDCl_3$) δ -191.75. HRMS (EI) calculated for $C_{12}H_9FOS$ $[M]^+$ 220.0353, found 220.0351.

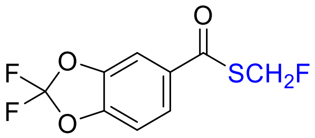

***S*-(fluoromethyl) 2,2-difluorobenzo[d][1,3]dioxole-5-carbothioate (3m)**. Following the general procedure I, the title compound (46.0 mg) was obtained in 92% yield. ^{1}H NMR (400 MHz, $CDCl_3$) δ 7.84 (dd, *J* = 8.4, 1.8 Hz, 1H), 7.70 (d, *J* = 1.7 Hz, 1H), 7.18 (d, *J* = 8.4 Hz, 1H), 5.99 (d, *J* = 50.0 Hz, 2H). ^{13}C NMR (126 MHz, $CDCl_3$) δ 186.19, 148.04, 144.35, 132.42, 131.82 (t, *J* = 258.5 Hz), 125.26, 109.74, 109.04, 80.81 (d, *J* = 216.7 Hz). ^{19}F NMR (377 MHz, $CDCl_3$) δ -49.62, -192.17. HRMS ESI $[M+H]^+$ calculated for $C_9H_6F_3O_3S$ 250.9984, found 250.9981.

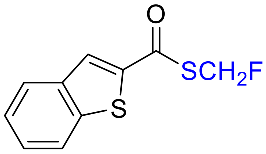

***S*-(fluoromethyl) benzo[b]thiophene-2-carbothioate (3n)**. Following the general procedure I, the title compound (36.2 mg) was obtained in 80% yield. ^{1}H NMR (400 MHz, $CDCl_3$) δ 8.13 (d, *J* = 0.5 Hz, 1H), 7.95 – 7.85 (m, 2H), 7.55 – 7.41 (m, 2H), 6.03 (d, *J* = 50.2 Hz, 2H). ^{13}C NMR (126 MHz, $CDCl_3$) δ 181.34, 142.54, 140.24, 135.18, 129.99, 128.10, 126.30, 125.55, 123.09, 80.94 (d, *J* = 218.0 Hz). ^{19}F NMR (377 MHz, $CDCl_3$) δ -191.17. HRMS (EI) calculated for $C_{10}H_7FOS_2$ $[M]^+$ 225.9917, found 225.9913.

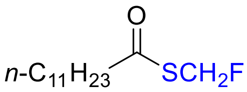

***S*-(fluoromethyl) dodecanethioate (3o)**.[12] Following the general procedure I, the title compound (42.2 mg) was obtained in 85% yield. ^{1}H NMR (400 MHz, $CDCl_3$) δ 5.80 (d, *J* = 50.3 Hz, 2H), 2.64 (td, *J* = 7.5, 1.2 Hz, 2H), 1.76 – 1.65 (m, 2H), 1.34 – 1.15 (m, 16H), 0.88 (d, *J* = 6.8 Hz, 3H). ^{13}C NMR (126 MHz, $CDCl_3$) δ 195.86, 80.99 (d, *J* = 212.8 Hz), 44.54, 32.04, 29.72, 29.70, 29.51, 29.46, 29.33, 29.00, 25.26, 22.83, 14.26. ^{19}F NMR (377 MHz, $CDCl_3$) δ -192.20. HRMS ESI $[M+H]^+$ calculated for $C_{13}H_{26}FOS$ 249.1683, found 249.1681.

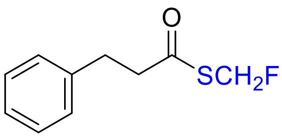

***S*-(fluoromethyl) 3-phenylpropanethioate (3p)**. Following the general procedure I, the title compound (34.9 mg) was obtained in 88% yield. ^{1}H NMR (400 MHz, $CDCl_3$) δ 7.40 – 7.26 (m, 2H), 7.23 – 7.15 (m, 3H), 5.81 (d, *J* = 50.2 Hz, 2H), 3.31 – 2.85 (m, 4H). ^{13}C NMR (126 MHz, $CDCl_3$) δ 194.92, 139.65, 128.78, 128.43, 126.69, 80.92 (d, *J* = 213.1 Hz), 45.95, 30.99.

^{19}F NMR (377 MHz, $CDCl_3$) δ -192.32. HRMS (EI) calculated for $C_{10}H_{11}FOS$ $[M]^+$ 198.0509, found 198.0503.

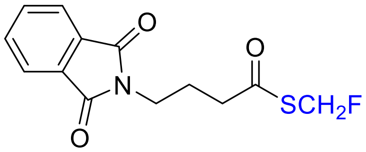

***S*-(fluoromethyl) 4-(1,3-dioxoisoindolin-2-yl)butanethioate (3q)**. Following the general procedure I, the title compound (49.5 mg) was obtained in 88% yield. ^{1}H NMR (400 MHz, $CDCl_3$) δ 7.84 (dd, *J* = 5.5, 3.0 Hz, 2H), 7.72 (dd, *J* = 5.5, 3.0 Hz, 2H), 5.76 (d, *J* = 50.1 Hz, 2H), 3.75 (t, *J* = 6.8 Hz, 2H), 2.73 (t, *J* = 7.4 Hz, 2H), 2.40 – 1.95 (m, 2H). ^{13}C NMR (126 MHz, $CDCl_3$) δ 194.67, 168.40, 134.19, 132.09, 123.46, 80.83 (d, *J* = 215.5 Hz), 41.65, 37.03, 23.99. ^{19}F NMR (377 MHz, $CDCl_3$) δ -192.30. HRMS (EI) calculated for $C_{13}H_{12}FNO_3S$ $[M]^+$ 281.0516, found 281.0522.

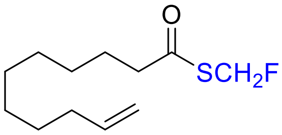

***S*-(fluoromethyl) undec-10-enethioate (3r)**. Following the general procedure I, the title compound (36.7 mg) was obtained in 79% yield. ^{1}H NMR (400 MHz, $CDCl_3$) δ 5.80 (d, *J* = 50.3 Hz, 2H), 5.85 – 5.75 (m, 1H), 5.29 – 4.63 (m, 2H), 2.64 (td, *J* = 7.5, 1.2 Hz, 2H), 2.14 – 1.90 (m, 2H), 1.69 (p, *J* = 7.5 Hz, 2H), 1.40 – 1.09 (m, 10H). ^{13}C NMR (126 MHz, $CDCl_3$) δ 195.80, 139.30, 114.33, 80.99 (d, *J* = 215.0 Hz), 44.53, 33.91, 29.33, 29.27, 29.15, 29.01, 28.97, 25.24. ^{19}F NMR (377 MHz, $CDCl_3$) δ -192.22. HRMS ESI $[M+H]^+$ calculated for $C_{12}H_{22}FOS$ 233.1370, found 233.1365.

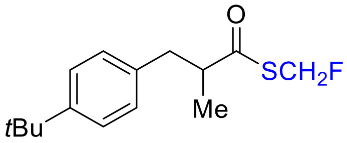

***S*-(fluoromethyl) 3-(4-(tert-butyl)phenyl)-2-methylpropanethioate (3s)**. Following the general procedure I, the title compound (45.6 mg) was obtained in 85% yield. ^{1}H NMR (400 MHz, $CDCl_3$) δ 7.31 (d, *J* = 8.3 Hz, 2H), 7.09 (d, *J* = 8.5 Hz, 2H), 5.82 (dd, *J* = 50.4, 9.2 Hz, 1H), 5.78 (dd, *J* = 50.4, 9.2 Hz, 1H), 3.10 (dd, *J* = 13.6, 6.1 Hz, 1H), 3.02 – 2.91 (m, 1H), 2.65 (dd, *J* = 13.6, 8.3 Hz, 1H), 1.30 (s, 9H), 1.21 (d, *J* = 6.9 Hz, 3H). ^{13}C NMR (126 MHz, $CDCl_3$) δ 199.49, 149.58, 135.42, 128.83, 125.52, 80.89 (d, *J* = 212.9 Hz), 50.77, 38.98, 34.55, 31.50, 16.92. ^{19}F NMR (377 MHz, $CDCl_3$) δ -192.52. HRMS (EI) calculated for $C_{15}H_{21}FOS$ $[M]^+$ 268.1292, found 268.1293.

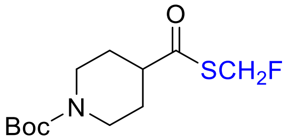

***tert*-Butyl 4-(((fluoromethyl)thio)carbonyl)piperidine-1-carboxylate (3t)**. Following the general procedure I, the title compound (37.2 mg) was obtained in 67% yield. ^{1}H NMR (400 MHz, $CDCl_3$) δ 5.80 (d, *J* = 50.1 Hz, 2H), 4.19 – 4.01 (m, 2H), 2.81 (t, *J* = 11.9 Hz, 2H), 2.75 – 2.63 (m, 1H), 2.03 – 1.86 (m, 2H), 1.76 – 1.60 (m, 3H), 1.45 (s, 9H); ^{13}C NMR (126 MHz,

$CDCl_3$) δ 197.83, 154.70, 80.64 (d, *J* = 215.5 Hz), 79.99, 50.69, 43.27, 28.54, 28.26. ^{19}F NMR (377 MHz, $CDCl_3$) δ -192.63. HRMS ESI $[M+H]^+$ calculated for $C_{12}H_{21}FNO_3S$ 278.1221, found 278.1214.

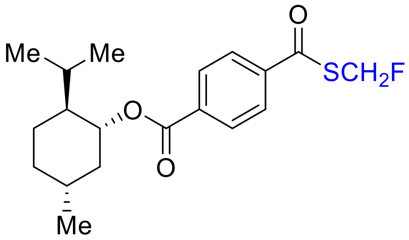

(*1R,2S,5R*)-2-Isopropyl-5-methylcyclohexyl 4-(((fluoromethyl)thio)carbonyl)benzoate (3u). Following the general procedure I, the title compound (32.4 mg) was obtained in 46% yield. ^{1}H NMR (400 MHz, $CDCl_3$) δ 8.15 (d, *J* = 8.7 Hz, 2H), 8.04 (d, *J* = 8.7 Hz, 2H), 6.01 (d, *J* = 50.0 Hz, 2H), 4.96 (td, *J* = 10.9, 4.4 Hz, 1H), 2.17 – 2.08 (m, 1H), 1.98 – 1.88 (m, 1H), 1.79 – 1.68 (m, 2H), 1.65 – 1.51 (m, 3H), 1.17 – 1.07 (m, 2H), 0.94 (d, *J* = 5.7 Hz, 3H), 0.92 (d, *J* = 6.2 Hz, 3H), 0.80 (d, *J* = 7.0 Hz, 3H). ^{13}C NMR (126 MHz, $CDCl_3$) δ 187.86, 165.03, 139.09, 135.88, 130.17, 127.77, 80.73 (d, *J* = 216.7 Hz), 75.87, 47.36, 41.01, 34.38, 31.60, 26.71, 23.76, 22.16, 20.88, 16.65. ^{19}F NMR (377 MHz, $CDCl_3$) δ -192.55. HRMS ESI $[M+H]^+$ calculated for $C_{16}H_{26}FO_3S$ 353.1581, found 353.1576.

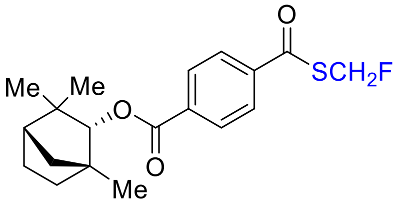

(*1R,2R,4S*)-1,3,3-Trimethylbicyclo[2.2.1]heptan-2-yl 4-(((fluoromethyl)thio)carbonyl)benzoate (3v). Following the general procedure I, the title compound (37.8 mg) was obtained in 54% yield. ^{1}H NMR (400 MHz, $CDCl_3$) δ 8.17 (d, *J* = 8.7 Hz, 2H), 8.06 (d, *J* = 8.6 Hz, 2H), 6.01 (d, *J* = 50.0 Hz, 2H), 4.65 (d, *J* = 1.9 Hz, 1H), 1.96 – 1.86 (m, 1H), 1.84 – 1.75 (m, 2H), 1.71 – 1.64 (m, 1H), 1.60 – 1.49 (m, 1H), 1.30 – 1.22 (m, 2H), 1.19 (s, 3H), 1.12 (s, 3H), 0.84 (s, 3H). ^{13}C NMR (126 MHz, $CDCl_3$) δ 187.86, 165.83, 139.17, 135.71, 130.13, 127.87, 87.66, 80.74 (d, *J* = 216.7 Hz), 48.80, 48.53, 41.59, 40.04, 29.89, 27.02, 26.03, 20.44, 19.63. ^{19}F NMR (377 MHz, $CDCl_3$) δ -192.57. HRMS ESI $[M+H]^+$ calculated for $C_{19}H_{24}FO_3S$ 351.1425, found 351.1417.

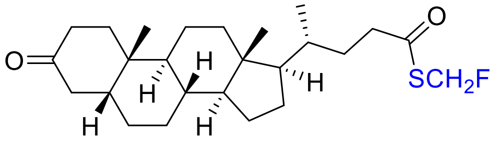

***S*-(fluoromethyl) (R)-4-((5R,8R,9S,10S,13R,14S,17R)-10,13-dimethyl-3-oxohexadecahydro-1H-cyclopenta[a]phenanthren-17-yl)pentanethioate (3w)**. Following the general procedure I, the title compound (43.1 mg) was obtained in 51% yield. ^{1}H NMR (400 MHz, $CDCl_3$) δ 5.80 (d, *J* = 50.2 Hz, 2H), 2.75 – 2.64 (m, 2H), 2.57 (ddd, *J* = 15.4, 8.6, 6.5 Hz, 1H), 2.33 (td, *J* = 14.5, 5.3 Hz, 1H), 2.21 – 2.11 (m, 1H), 2.07 – 1.97 (m, 3H), 1.93 – 1.77 (m, 4H), 1.66 – 1.57 (m, 1H), 1.52 – 1.35 (m, 7H), 1.30 – 1.17 (m, 4H), 1.15 – 1.06 (m, 4H), 1.01 (s, 3H), 0.93 (d, *J* = 6.4 Hz, 3H), 0.68 (s, 3H). ^{13}C NMR (126 MHz, $CDCl_3$) δ 213.55, 196.17, 81.02 (d, *J* = 214.2 Hz), 56.56, 55.99, 44.45, 42.96, 42.51, 41.60, 40.87, 40.18, 37.36,

37.15, 35.67, 35.35, 35.03, 31.23, 28.29, 26.75, 25.90, 24.28, 2c2.80, 21.33, 18.48, 12.23. ^{19}F NMR (377 MHz, $CDCl_3$) δ -192.12. HRMS ESI $[M+Na]^+$ calculated for $C_{25}H_{39}FO_2SNa$ 445.2547, found 445.2547.

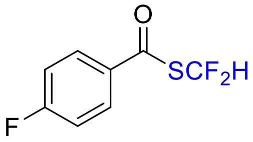

***S*-(difluoromethyl) 4-fluorobenzothioate (3x).**[13] Following the general procedure I, the title compound (36.7 mg) was obtained in 89% yield. ^{1}H NMR (400 MHz, $CDCl_3$) δ 7.94 (dd, *J* = 9.0, 5.2 Hz, 2H), 7.48 (t, *J* = 55.2 Hz, 1H), 7.23 – 7.14 (m, 2H). ^{13}C NMR (126 MHz, $CDCl_3$) δ 185.93, 166.83 (d, $^1J_{C-F}$ = 258.3 Hz), 132.08, 130.48 (d, $^3J_{C-F}$ = 10.1 Hz), 120.58 (t, $^1J_{C-F}$ = 270.9 Hz), 116.54 (d, $^2J_{C-F}$ = 22.7 Hz). ^{19}F NMR (377 MHz, $CDCl_3$) δ -99.25, -101.66. HRMS ESI $[M+H]^+$ calculated for $C_8H_6F_3OS$ 207.0086, found 207.0093.

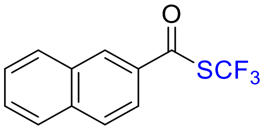

***S*-(trifluoromethyl) naphthalene-2-carbothioate (3y).**[14] Following the general procedure I, the title compound (33.3 mg) was obtained in 65% yield. ^{1}H NMR (400 MHz, $CDCl_3$) δ 8.41 (d, *J* = 1.4 Hz, 1H), 8.04 – 7.81 (m, 4H), 7.70 – 7.58 (m, 2H). ^{13}C NMR (126 MHz, $CDCl_3$) δ 183.33, 136.53, 132.59, 132.41, 130.03, 129.88, 129.73, 129.44, 128.25 (q, $^1J_{C-F}$ = 310.0 Hz), 128.13, 127.71, 122.74. ^{19}F NMR (377 MHz, $CDCl_3$) δ -39.51. HRMS (EI) $[M]^+$ calculated for $C_{12}H_7F_3OS$ 256.0170, found 256.0177.

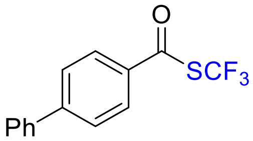

***S*-(trifluoromethyl) [1,1'-biphenyl]-4-carbothioate (3z).**[14] Following the general procedure I, the title compound (28.2 mg) was obtained in 50% yield. ^{1}H NMR (500 MHz, $CDCl_3$) δ 7.94 (d, *J* = 8.4 Hz, 2H), 7.73 (d, *J* = 8.4 Hz, 2H), 7.67 – 7.58 (m, 2H), 7.54 – 7.47 (m, 2H), 7.46 – 7.40 (m, 1H). ^{13}C NMR (126 MHz, $CDCl_3$) δ 182.92, 148.05, 139.30, 133.87, 129.26, 128.95, 128.41, 128.22 (q, $^1J_{C-F}$ = 310.0 Hz), 127.91, 127.47. ^{19}F NMR (377 MHz, $CDCl_3$) δ -39.56. HRMS (EI) $[M]^+$ calculated for $C_{14}H_9F_3OS$ 282.0326, found 282.0321.

General procedure II: neutral-eosin Y-photocatalyzed aldehydic C–H arylthiolation

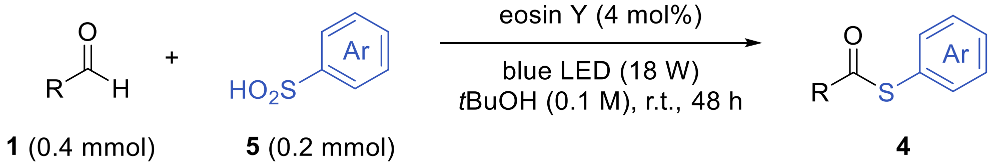

A 20 mL Schlenk tube equipped with a magnetic stir bar was charged with eosin Y (0.008 mmol, 5.2 mg), aldehyde **1** (0.4 mmol), and arylsulfinic acid **5** (0.2 mmol). Then, 2.0 mL of anhydrous *tert*-butanol was added. The Schlenk tube was connected to Schlenk line and freeze-pump-thaw was performed for three times to completely remove air inside the reaction mixture.

Eventually the Schlenk tube was refilled with an atmosphere of argon at room temperature and sealed. The reaction vessel was surrounded by a coil of blue LED strip (2 meters, 18 W). Then the reaction was running at ambient temperature (~27 ^{o}C) using a fan to cool down the reaction mixture and stopped after 48 h. The solvent was removed under reduced pressure and the crude mixture was purified by silica gel column chromatography or prepared TLC (eluent: hexane/diethyl ether or hexane/ethyl acetate; 10/1 – 3/1) to give the corresponding product **4**.

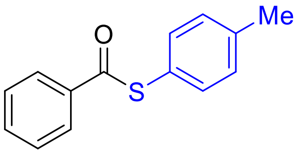

***S*-(p-tolyl) benzothioate (4a).** Following the general procedure II, the tittle compound **4a** (29.2 mg) was obtained in 64% yield. ^{1}H NMR (500 MHz, $CDCl_3$) δ 8.04 – 8.02 (m, 2H), 7.62 – 7.59 (m, 1H), 7.50 – 7.47 (m, 2H), 7.41 – 7.40 (m, 2H), 6.28 – 6.27 (m, 2H), 2.41 (s, 3H). ^{13}C NMR (126 MHz, $CDCl_3$) δ 190.62, 139.84, 136.74, 135.05, 133.59, 130.14, 128.74, 127.49, 123.79, 21.40. HRMS (EI) calculated for $C_{14}H_{12}OS$ $[M]^+$ 228.0603, found 228.0599.

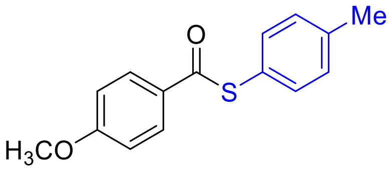

***S*-(p-tolyl) 4-methoxybenzothioate (4c).** Following the general procedure II, the tittle compound **4c** (32.5 mg) was obtained in 63% yield. ^{1}H NMR (500 MHz, $CDCl_3$) δ 8.02 – 8.00 (m, 2H), 7.40 – 7.39 (m, 2H), 7.26 – 7.25 (m, 2H), 6.97 – 6.95 (m, 2H), 3.88 (s, 3H), 2.40 (s, 3H). ^{13}C NMR (126 MHz, $CDCl_3$) δ 189.06, 163.95, 139.65, 135.15, 130.05, 129.70, 129.52, 124.08, 113.90, 55.56, 21.39. HRMS (EI) calculated for $C_{15}H_{14}O_2S$ $[M]^+$ 258.0709, found 258.0718.

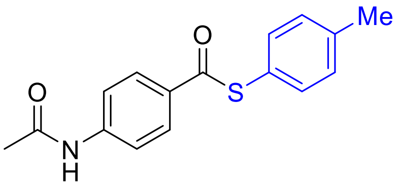

***S*-(p-tolyl) 4-acetamidobenzothioate (4d).** Following the general procedure II, the tittle compound **4d** (26.2 mg) was obtained in 46% yield. ^{1}H NMR (400 MHz, $CDCl_3$) δ 8.06 – 7.93 (m, 2H), 7.62 (d, J = 8.3 Hz, 2H), 7.51 (s, 1H), 7.39 – 7.37 (m, 2H), 7.25 (d, J = 8.2 Hz, 2H), 2.39 (s, 3H), 2.20 (s, 3H).^{13}C NMR (126 MHz, $CDCl_3$) δ 189.48, 168.50, 142.74, 139.83, 135.08, 132.18, 130.11, 128.86, 123.76, 118.91, 24.82, 21.38.

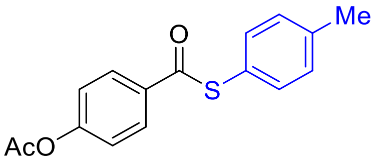

4-((p-tolylthio)carbonyl)phenyl acetate (4e). Following the general procedure II, the tittle

compound **4e** (29.7 mg) was obtained in 52% yield. ^{1}H NMR (500 MHz, $CDCl_3$) δ 8.06 (d, J = 8.6 Hz, 2H), 7.39 (d, J = 8.1 Hz, 2H), 7.27 (d, J = 8.3 Hz, 2H), 7.22 (d, J = 8.6 Hz, 2H), 2.41 (s, 3H), 2.33 (s, 3H). ^{13}C NMR (126 MHz, $CDCl_3$) δ 189.40, 168.69, 154.74, 139.89, 135.01, 134.26, 130.13, 129.04, 123.63, 121.92, 21.36, 21.13.

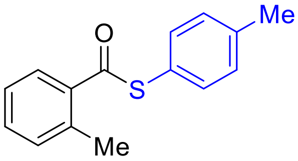

***S*-(p-tolyl) 2-methylbenzothioate (4f).** Following the general procedure II, the tittle compound **4f** (34.3 mg) was obtained in 71% yield. ^{1}H NMR (500 MHz, $CDCl_3$) δ 7.95 – 7.93 (m, 1H), 7.44 – 7.39 (m, 3H), 7.32 – 7.27 (m, 4H), 2.49 (s, 3H), 2.41 (s, 3H). ^{13}C NMR (126 MHz, $CDCl_3$) δ 192.69, 139.77, 137.40, 136.86, 134.88, 131.94, 131.73, 130.14, 128.63, 125.84, 124.68, 21.41, 20.78. HRMS (EI) calculated for $C_{15}H_{14}OS$ $[M]^+$ 242.076, found 242.0764.

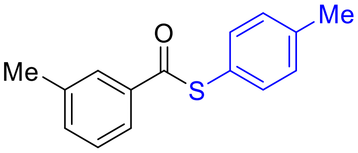

***S*-(p-tolyl) 3-methylbenzothioate (4g).** Following the general procedure II, the tittle compound **4g** (30.9 mg) was obtained in 64% yield. ^{1}H NMR (500 MHz, $CDCl_3$) δ 7.84 – 7.82 (m, 2H), 7.42 – 7.35 (m, 4H), 7.28 – 7.26 (m, 2H), 2.43 (s, 3H), 2.41 (s, 3H). ^{13}C NMR (126 MHz, $CDCl_3$) δ 190.72, 139.77, 138.63, 136.76, 135.03, 134.46, 130.11, 128.61, 127.94, 124.71, 123.94, 21.40, 21.36. HRMS (EI) calculated for $C_{15}H_{14}OS$ $[M]^+$ 242.076, found 242.0763.

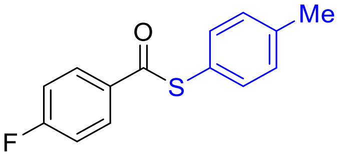

***S*-(p-tolyl) 4-fluorobenzothioate (4h).** Following the general procedure II, the tittle compound **4h** (33.4 mg) was obtained in 68% yield. ^{1}H NMR (500 MHz, $CDCl_3$) δ 8.08 – 8.04 (m, 2H), 7.40 – 7.38 (m, 2H), 7.28 – 7.27 (m, 2H), 7.18 – 7.14 (m, 2H), 2.41 (s, 3H). ^{13}C NMR (126 MHz, $CDCl_3$) δ 189.17, 166.05 (d, $^{1}J_{C\text{-}F}$ = 255.2 Hz), 139.99, 135.04, 133.06 (d, $^{4}J_{C\text{-}F}$ = 2.8 Hz), 130.18, 130.06 (d, $^{3}J_{C\text{-}F}$ = 9.3 Hz), 123.48, 115.90 (d, $^{2}J_{C\text{-}F}$ = 22.2 Hz), 21.4. ^{19}F NMR (377 MHz, $CDCl_3$) δ -104.34. HRMS (EI) calculated for $C_{14}H_{11}FOS$ $[M]^+$ 246.0509, found 246.0516.

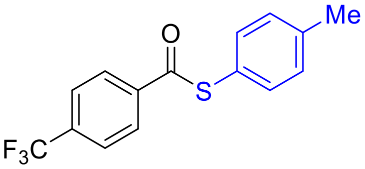

***S*-(p-tolyl) 4-(trifluoromethyl)benzothioate (4i).** Following the general procedure II, the tittle compound **4i** (38.5 mg) was obtained in 65% yield. ^{1}H NMR (500 MHz, $CDCl_3$) δ: 8.14 – 8.11 (m, 2H), 7.75 (d, J = 8.3 Hz, 2H), 7.40 (d, J = 8.2 Hz, 2H), 7.30 – 7.28 (m, 2H), 2.42 (s, 3H). ^{13}C NMR (126 MHz, $CDCl_3$) δ 189.87, 140.26, 139.53, 134.92, 134.87 (q, $^{2}J_{C\text{-}F}$ = 34.02),

130.28, 127.82, 125.83 (q, $^{3}J_{C\text{-}F}$ = 3.7), 123.53 (q, $^{1}J_{C\text{-}F}$ = 273.42), 123.00, 21.41. ^{19}F NMR (377 MHz, $CDCl_3$) δ -63.13. HRMS (EI) calculated for $C_{15}H_{11}F_3OS$ $[M]^+$ 296.0477, found 296.0489.

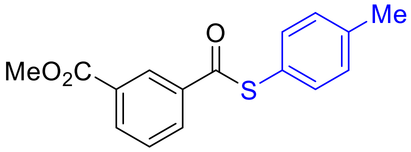

Methyl 3-((p-tolylthio)carbonyl)benzoate (4j). Following the general procedure II, the tittle compound **4j** (32.0 mg) was obtained in 56% yield. ^{1}H NMR (400 MHz, $CDCl_3$) δ 8.69 – 6.68 (m, 1H), 8.28 – 8.26 (m, 1H), 8.19 – 8.17 (m, 1H), 7.58 (t, *J* = 7.8 Hz, 1H), 7.41 – 7.39 (m, 2H), 7.29 – 7.27 (m, 2H), 3.97 (s, 3H), 2.41 (s, 3H). ^{13}C NMR (126 MHz, $CDCl_3$) δ 189.99, 166.07, 140.07, 137.06, 134.97, 134.32, 131.49, 130.91, 130.22, 129.00, 128.62, 123.32, 52.50, 21.41. HRMS (EI) calculated for $C_{16}H_{14}O_3S$ $[M]^+$ 286.0658, found 286.0657.

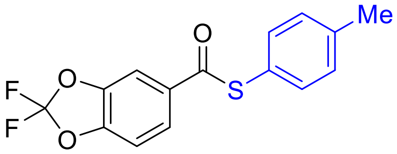

***S*-(p-tolyl) 2,2-difluorobenzo[d][1,3]dioxole-5-carbothioate (4k).** Following the general procedure II, the tittle compound **4k** (41.9 mg) was obtained in 68% yield. ^{1}H NMR (400 MHz, $CDCl_3$) δ 7.89(dd, *J* = 8.4 Hz, 1.8 Hz, 1H), 7.72 (d, *J* = 1.7 Hz, 1H), 7.40 – 7.37 (m, 2H), 7.29 – 7.27 (m, 2H), 7.16 (d, *J* = 8.4 Hz, 1H), 2.41 (s, 3H). ^{13}C NMR (126 MHz, $CDCl_3$) δ 188.67, 147.39, 144.07, 140.19, 135.01, 133.09, 131.70 (t, $^{1}J_{C\text{-}F}$ = 258.3 Hz), 126.19, 124.57, 123.15, 109.41, 108.73, 21.40. ^{19}F NMR (377 MHz, $CDCl_3$) δ -49.68. HRMS (EI) calculated for $C_{15}H_{10}F_2O_3S$ $[M]^+$ 308.0313, found 308.0323.

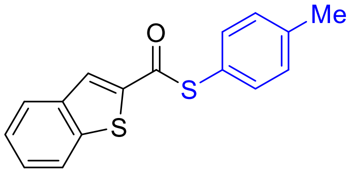

***S*-(p-tolyl) benzo[b]thiophene-2-carbothioate (4l).** Following the general procedure II, the tittle compound **4l** (17.6 mg) was obtained in 31% yield. ^{1}H NMR (500 MHz, $CDCl_3$) δ 8.17 (d, *J* = 0.8 Hz, 1H), 7.93 –7.87 (m, 2H), 7.50 – 7.42 (m, 4H), 7.29 – 7.27 (m, 2H), 2.41 (s, 3H). ^{13}C NMR (126 MHz, $CDCl_3$) δ 183.93, 142.04, 140.92, 140.16, 138.69, 134.99, 130.20, 128.57, 127.45, 125.92, 125.20, 123.13, 122.90, 21.42. HRMS (EI) calculated for $C_{16}H_{12}OS_2$ $[M]^+$ 284.0324, found 284.0333.

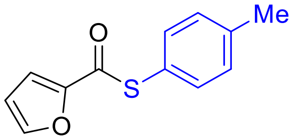

***S*-(p-tolyl) furan-2-carbothioate (4m).** Following the general procedure II, the tittle compound **4m** (9.6 mg) was obtained in 22% yield. ^{1}H NMR (500 MHz, $CDCl_3$) δ 7.62 (dd, *J* = 1.7 Hz, 0.8 Hz, 1H), 7.39 – 7.38 (m, 2H), 7.27 – 7.25 (m, 2H), 6.57 (dd = 3.6 Hz, 1.7 Hz, 1H), 2.40 (s, 3H). ^{13}C NMR (126 MHz, $CDCl_3$) δ 179.16, 150.47, 146.39, 140.00, 135.10, 130.14, 122.57, 116.14, 112.39, 21.39.

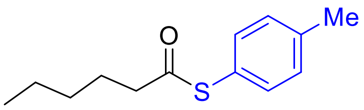

***S*-(p-tolyl) hexanethioate (4n).** Following the general procedure II, the tittle compound **4n** (17.7 mg) was obtained in 40% yield. ^{1}H NMR (500 MHz, $CDCl_3$) δ 7.28 (d, *J* = 8.1 Hz, 2H), 7.21 (d, *J* = 7.9 Hz, 2H), 2.63 (t, *J* = 7.5 Hz, 2H), 2.37 (s, 3H), 1.74- 1,68 (m, 2H), 1.36 – 1.33 (m, 5H), 0.92 – 0.89 (m, 3H). ^{13}C NMR (126 MHz, $CDCl_3$) δ 198.15, 139.55, 134.46, 129.99, 124.46, 43.60, 31.12, 25.32, 22.33, 21.34, 13.88. HRMS (EI) calculated for $C_{13}H_{18}OS$ $[M]^+$ 222.1073, found 222.107.

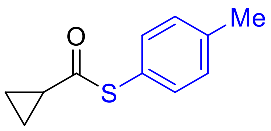

***S*-(p-tolyl) cyclopropanecarbothioate (4o).** Following the general procedure II, the tittle compound **4o** (11.1 mg) was obtained in 29% yield. ^{1}H NMR (400 MHz, $CDCl_3$) δ 7.32 – 7.29 (m, 2H), 7.22 – 7.19 (m, 2H), 2.37 (s, 3H), 2.09 (tt, *J* = 7.9 Hz, 4.6 Hz, 1H), 1.22- 1,18 (m, 2H), 1.01 – 0.97 (m, 2H). ^{13}C NMR (126 MHz, $CDCl_3$) δ 198.03, 139.58, 134.53, 129.97, 124.34, 22.07, 21.33, 11.09. HRMS (EI) calculated for $C_{11}H_{12}OS$ $[M]^+$ 192.0603, found 192.0602.

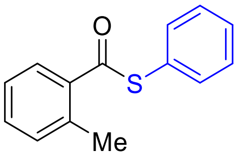

***S*-phenyl 2-methylbenzothioate (4p).** Following the general procedure II, the tittle compound **4p** (29.2 mg) was obtained in 57% yield. ^{1}H NMR (500 MHz, $CDCl_3$) δ 7.95 (dd, J_1 = 7.8 Hz, J_2 = 1.4 Hz, 1H), 7.55 – 7.52 (m, 2H), 7.49 – 7.42 (m, 4H), 7.33 – 7.27 (m, 2H), 2.50 (s, 3H). ^{13}C NMR (126 MHz, $CDCl_3$) δ 192.20, 137.45, 136.78, 134.93, 132.03, 131.77, 129.49, 129.29, 128.65, 128.24, 125.88, 20.79. HRMS (EI) calculated for $C_{14}H_{11}OS$ $[M]^+$ 227.0525 found 227.0523.

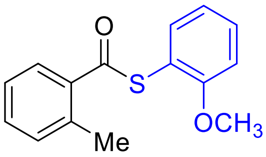

***S*-(2-methoxyphenyl) 2-methylbenzothioate (4q).** Following the general procedure II, the tittle compound **4q** (29.2 mg) was obtained in 57% yield. ^{1}H NMR (500 MHz, $CDCl_3$) δ 7.98 – 7.96 (m, 1H), 7.51 – 7.39 (m, 3H), 7.31 – 7.25 (m, 2H), 7.06 – 7.01 (m, 2H), 3.88 (s, 3H), 2.49 (s, 3H). ^{13}C NMR (126 MHz, $CDCl_3$) δ 191.58, 159.54, 137.24, 137.12, 137.00, 131.76, 131.74, 131.60, 128.66, 125.77, 121.18, 116.45, 111.66, 56.03, 20.63.

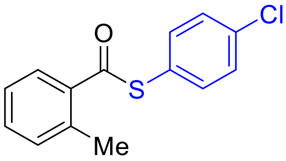

***S*-(4-chlorophenyl) 2-methylbenzothioate (4r).** Following the general procedure II, the tittle compound **4r** (25.9 mg) was obtained in 48% yield. ^{1}H NMR (500 MHz, $CDCl_3$) δ 7.94 (dd, *J* = 7.8 Hz, 1.4 Hz, 1H), 7.47 – 7.43 (m, 5H), 7.33 – 7.27 (m, 2H), 2.50 (s, 3H). ^{13}C NMR (126

MHz, $CDCl_3$) δ 191.56, 137.61, 136.39, 136.17, 135.93, 132.27, 131.87, 129.54, 128.69, 126.72, 125.95, 20.83. HRMS (EI) calculated for $C_{14}H_{10}ClOS$ $[M]^+$ 261.0135, found 261.0138.

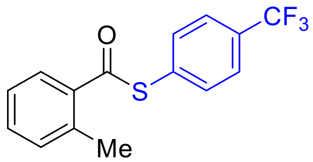

***S*-(4-(trifluoromethyl)phenyl) 2-methylbenzothioate (4s).** Following the general procedure II, the tittle compound **4s** (36.1 mg) was obtained in 61% yield. ^1H NMR (500 MHz, $CDCl_3$) δ 7.96 (dd, J = 7.8 Hz, 1.4 Hz, 1H), 7.82 – 7.63 (m, 4H), 7.46 (td, J = 7.5 Hz, 1.4 Hz, 1H), 7.39 – 7.28 (m, 2H), 2.51 (s, 3H). ^{13}C NMR (126 MHz, $CDCl_3$) δ 190.74, 137.77, 136.20, 135.05, 133.00, 132.46, 131.95, 131.38 (q, $^2J_{C\text{-}F}$ = 32.7 Hz), 128.74, 126.03 (q, $^3J_{C\text{-}F}$ = 3.8 Hz), 126.02, 123.87 (q, $^1J_{C\text{-}F}$ = 272.2 Hz), 20.85. ^{19}F NMR (377 MHz, $CDCl_3$) δ -62.83. HRMS (EI) calculated for $C_{15}H_{10}F_3OS$ $[M]^+$ 295.0399, found 295.0406.

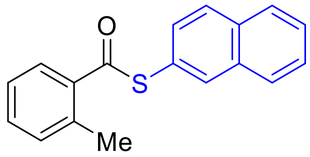

***S*-(naphthalen-2-yl) 2-methylbenzothioate (4t).** Following the general procedure II, the tittle compound **4t** (32.8 mg) was obtained in 59% yield. ^1H NMR (500 MHz, $CDCl_3$) δ 8.07 (d, J = 1.7 Hz, 1H), 8.01 (dd, J = 7.7 Hz, 1.4 Hz, 1H), 7.93 (d, J = 8.5 Hz, 1H), 7.88 (td, J = 7.7 Hz, 2.1 Hz, 2H), 7.59 – 7.52 (m, 3H), 7.45 (td, J = 7.5 Hz, 1.4 Hz, 1H), 7.35 – 7.29 (m, 2H), 2.52 (s, 3H). ^{13}C NMR (126 MHz, $CDCl_3$) δ 192.40, 137.51, 136.80, 134.79, 133.68, 133.45, 132.10, 131.82, 131.32, 128.87, 128.73, 128.06, 127.86, 127.19, 126.57, 125.93, 125.59, 20.82. HRMS (EI) calculated for $C_{18}H_{14}OS$ $[M]^+$ 278.076, found 278.077.

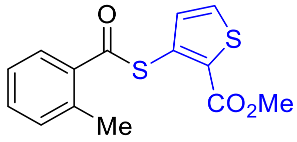

Methyl 3-((2-methylbenzoyl)thio)thiophene-2-carboxylate (4u). Following the general procedure II, the tittle compound **4u** (23.4 mg) was obtained in 40% yield. ^1H NMR (400 MHz, $CDCl_3$) δ 7.96 (dd, J = 7.7 Hz, 1.4 Hz, 1H), 7.61 (d, J = 5.2 Hz, 1H), 7.45 – 7.41 (m, 1H), 7.41 (d, J = 5.2 Hz, 1H), 7.33 – 7.26 (m, 2H), 3.88 (s, 3H), 2.49 (s, 3H). ^{13}C NMR (126 MHz, $CDCl_3$) δ 190.46, 161.45, 137.47, 136.73, 133.40, 132.33, 132.17, 131.70, 131.46, 130.31, 128.78, 125.93, 52.28, 20.56. HRMS (EI) calculated for $C_{14}H_{12}O_3S_2$ $[M]^+$ 292.0222, found 292.0224.

General procedure III: neutral-eosin Y-photocatalyzed aldehydic C–H alkynylation

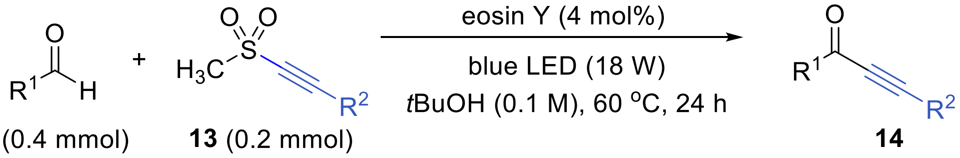

A 20 mL Schlenk tube equipped with a magnetic stir bar was charged with eosin Y (0.008 mmol, 5.2 mg), aldehyde **1** (0.4 mmol), acetylenic sulfone reagents **13** (0.2 mmol). Then, 2.0 mL of anhydrous *tert*-butanol was added. The Schlenk tube was connected to Schlenk line and freeze-pump-thaw was performed for three times to completely remove air inside the reaction

mixture. Eventually the Schlenk tube was refilled with an atmosphere of argon at room temperature and sealed. The reaction vessel was surrounded by a coil of blue LED strip (2 meters, 18 W). Then the reaction tubes were placed in a water bath covered by top oil layer (to prevent evaporation of water bath). The reaction was running at 60 °C and stopped after 24 h. The solvent was removed under reduced pressure and the crude mixture was purified by silica gel column chromatography or prepared TLC (eluent: hexane/diethyl ether or hexane/ethyl acetate; 10/1 – 3/1) to give the corresponding product **14**.

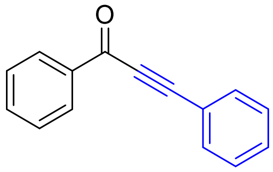

1,3-Diphenylprop-2-yn-1-one (14a). Following the general procedure III, the tittle compound **14a** (18.5 mg) was obtained in 45% yield. ^{1}H NMR (500 MHz, $CDCl_3$) δ 8.24 – 8.22 (m, 2H), 7.70 – 7.68 (m, 2H), 7.66 – 7.62(m, 1H), 7.54 – 7.48 (m, 3H), 7.45 – 7.42 (m, 2H). ^{13}C NMR (126 MHz, $CDCl_3$) δ 178.1, 136.9, 134.1, 133.1, 130.8, 129.6, 128.7, 128.6, 120.2, 93.1, 86.9. HRMS (EI) calculated for $C_{15}H_{10}O$ $[M]^+$ 206.0726, found 206.0722.

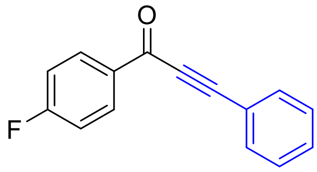

1-(4-fluorophenyl)-3-phenylprop-2-yn-1-one (14b). Following the general procedure III, the tittle compound **14b** (18.8 mg) was obtained in 42% yield. ^{1}H NMR (500 MHz, $CDCl_3$) δ 8.27 – 8.23 (m, 2H), 7.69 – 7.67 (m, 2H), 7.51 – 7.48 (m, 1H), 7.45 – 7.41(m, 2H), 7.21 – 7.17(m, 2H). ^{13}C NMR (126 MHz, $CDCl_3$) δ 176.42, 166.49 (d, $^{1}J_{C-F}$ = 256.5 Hz), 133.43 (d, $^{4}J_{C-F}$ = 2.6 Hz), 133.08, 132.26 (d, $^{3}J_{C-F}$ = 9.6 Hz), 130.93, 128.75, 119.99, 115.89 (d, $^{2}J_{C-F}$ = 22.1 Hz), 93.39, 86.62. ^{19}F NMR (377 MHz, $CDCl_3$) δ -103.18. HRMS (EI) calculated for $C_{15}H_9FO$ $[M]^+$ 224.0632, found 224.0633.

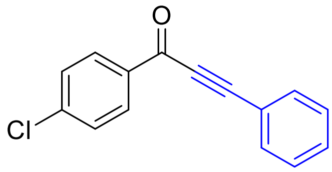

1-(4-Chlorophenyl)-3-phenylprop-2-yn-1-one (14c). Following the general procedure III, the tittle compound **14c** (22.1 mg) was obtained in 46% yield. ^{1}H NMR (500 MHz, $CDCl_3$) δ 8.17 – 8.15 (m, 2H), 7.70 – 7.67 (m, 2H), 7.52 – 7.49 (m, 3H), 7.45 – 7.42(m, 2H). ^{13}C NMR (126 MHz, $CDCl_3$) δ 176.68, 140.74, 135.34, 133.12, 130.99, 130.89, 129.03, 128.76, 119.93, 93.65, 86.60. HRMS (EI) calculated for $C_{15}H_9ClO$ $[M]^+$ 240.0336, found 240.0337.

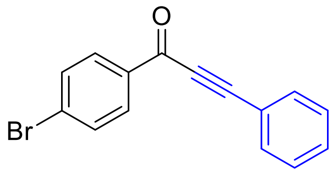

1-(4-Bromophenyl)-3-phenylprop-2-yn-1-one (14d). Following the general procedure III, the tittle compound **14d** (27.2 mg) was obtained in 48% yield. ^{1}H NMR (400 MHz, $CDCl_3$) δ 8.09 – 8.07 (m, 2H), 7.69 – 7.66 (m, 4H), 7.52 – 7.49 (m, 1H), 7.45 – 7.42(m, 2H). ^{13}C NMR (126 MHz, $CDCl_3$) δ 176.90, 135.72, 133.14, 132.03, 131.02, 130.97, 129.60, 128.77, 119.90, 93.72, 86.58. HRMS (EI) calculated for $C_{15}H_9BrO$ $[M]^+$ 283.9831, found 283.9838.

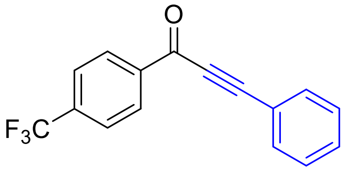

3-Phenyl-1-(4-(trifluoromethyl)phenyl)prop-2-yn-1-one (14e). Following the general procedure III, the tittle compound **14e** (28.5 mg) was obtained in 52% yield. ^{1}H NMR (500 MHz, $CDCl_3$) δ 8.34 – 8.32 (m, 2H), 7.81 – 7.78 (m, 2H), 7.71 – 7.69 (m, 2H), 7.55 – 7.50 (m, 1H), 7.47 – 7.43 (m, 2H). ^{13}C NMR (126 MHz, $CDCl_3$) δ 176.74, 139.42, 135.23 (q, $^{2}J_{C\text{-}F}$ = 32.76 Hz), 133.22, 131.22, 129.82, 128.82, 125.74 (q, $^{3}J_{C\text{-}F}$ = 3.7 Hz), 123.56 (q, $^{1}J_{C\text{-}F}$ = 274.68 Hz), 119.70, 94.50, 86.60. ^{19}F NMR (400 MHz, $CDCl_3$) δ -63.13. HRMS (EI) calculated for $C_{16}H_9F_3O$ $[M]^+$ 274.06, found 274.0611.

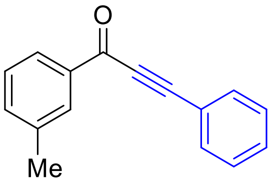

3-Phenyl-1-(m-tolyl)prop-2-yn-1-one (14f). Following the general procedure III, the tittle compound **14f** (30.4 mg) was obtained in 69% yield. ^{1}H NMR (400 MHz, $CDCl_3$) δ 8.05 – 8.01 (m, 2H), 7.70 – 7.68 (m, 2H), 7.51 – 7.39 (m, 5H), 2.45 (s, 3H). ^{13}C NMR (126 MHz, $CDCl_3$) δ 178.26, 138.53, 136.96, 135.00, 133.07, 130.75, 129.81, 128.70, 128.54, 127.15, 120.25, 92.90, 87.04, 21.36. HRMS (EI) calculated for $C_{16}H_{12}O$ $[M]^+$ 220.0883, found 220.0884.

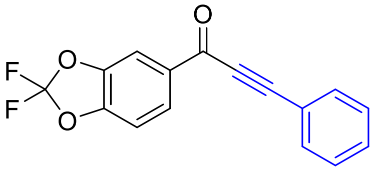

1-(2,2-Difluorobenzo[d][1,3]dioxol-5-yl)-3-phenylprop-2-yn-1-one (14g). Following the general procedure III, the tittle compound **14g** (23.5 mg) was obtained in 41% yield. ^{1}H NMR (500 MHz, $CDCl_3$) δ 8.10 (dd, J = 8.3 Hz, 1.7 Hz, 1H), 7.90 (d, J = 1.6 Hz, 1H), 7.70 – 7.68 (m, 2H), 7.53 – 7.49 (m, 1H), 7.46 – 7.42 (m, 2H), 7.20 (d, J = 8.3 Hz, 1H). ^{13}C NMR (126 MHz, $CDCl_3$) δ 175.62, 147.89, 144.15, 133.63, 131.70 (t, $^{1}J_{C\text{–}F}$ = 258.3 Hz), 131.10, 126.33, 128.80, 127.47, 119.77, 110.04, 109.36, 93.82, 86.33. ^{19}F NMR (400 MHz, $CDCl_3$) δ -49.69. HRMS (EI) calculated for $C_{16}H_8F_2O_3$ $[M]^+$ 286.0436, found 286.0441.

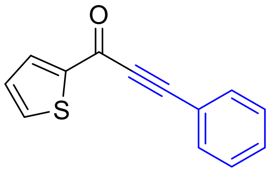

3-Phenyl-1-(thiophen-2-yl)prop-2-yn-1-one (14h). Following the general procedure III, the tittle compound **14h** (17.8 mg) was obtained in 42% yield. ^{1}H NMR (500 MHz, $CDCl_3$) δ 8.02 (dd, J = 3.8 Hz, 1.2 Hz, 1H), 7.73 (dd, J = 4.9 Hz, 1.2 Hz, 1H), 7.68 – 7.66 (m, 2H), 7.51 – 7.47 (m, 1H), 7.44 – 7.41 (m, 2H), 7.20 – 7.18 (m, 1H). ^{13}C NMR (126 MHz, $CDCl_3$) δ 169.83, 144.99, 135.25, 135.08, 133.07, 130.87, 128.72, 128.35, 119.99, 91.75, 86.50. HRMS (EI) calculated for $C_{13}H_8OS$ $[M]^+$ 212.029, found 212.0294.

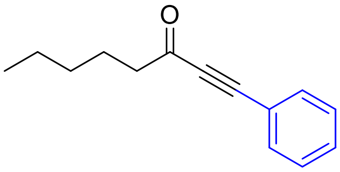

1-Phenyloct-1-yn-3-one (14i). Following the general procedure III, the tittle compound **14i** (29.6 mg) was obtained in 74% yield. ^{1}H NMR (500 MHz, $CDCl_3$) δ 7.57 (d, J = 7.6 Hz, 2H), 7.45 (t, J = 7.4Hz, 1H), 7.38 (t, J = 7.5Hz, 2H), 2.66 (t, J = 7.4Hz, 3H), 1.75(t, J = 7.4Hz, 2H), 1.37 – 1.35 (m, 4H), 0.92-0.90 (m, 2H). ^{13}C NMR (126 MHz, $CDCl_3$) δ 188.35, 133.04, 130.64, 128.62, 120.09, 90.56, 87.87, 45.53, 31.18, 23.88, 22.42, 13.91. HRMS (EI) calculated for $C_{14}H_{15}O$ $[M]^+$ 199.1117, found 199.1112.

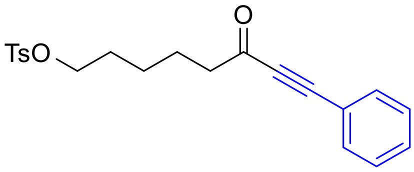

6-Oxo-8-phenyloct-7-yn-1-yl 4-methylbenzenesulfonate (14j). Following the general procedure III, the tittle compound **14j** (51.8 mg) was obtained in 70% yield. ^{1}H NMR (500 MHz, $CDCl_3$) δ 7.78 (d, J = 8.4 Hz, 2H), 7.58 – 7.56 (m, 2H), 7.48 – 7.45 (m, 1H), 7.40 – 7.37 (m, 2H), 7.34 (d, J = 8.1 Hz, 2H), 4.03 (t, J = 6.4 Hz, 2H), 2.63 (t, J = 7.3 Hz, 2H), 2.44 (s, 3H), 1.72 – 1.58 (m, 4H), 1.42 – 1.36 (m, 2H). ^{13}C NMR (126 MHz, $CDCl_3$) δ 187.52, 157.68, 144.87, 133.08, 130.79, 129.87, 128.67, 127.90, 119.90, 90.88, 87.74, 70.21, 45.15, 28.65, 24.83, 23.34, 21.66. HRMS ESI $[M+Na]^+$ calculated for $C_{21}H_{22}NaO_4S$ 393.1131, found 393.1131.

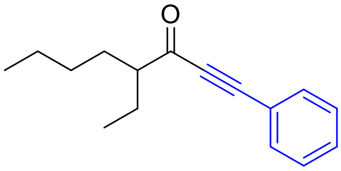

4-Ethyl-1-phenyloct-1-yn-3-one (14k). Following the general procedure III, the tittle compound **14k** (32.4 mg) was obtained in 71% yield. ^{1}H NMR (500 MHz, $CDCl_3$) δ 7.59 – 7.57 (m, 2H), 7.47 – 7.44 (m, 1H), 7.40 – 7.37 (m, 2H), 2.50 (tt, J = 8.4 Hz, 5.4 Hz, 1H), 1.85 – 1.76 (m, 2H), 1.66 – 1.54 (m, 2H), 1.37 – 1.29 (m, 4H), 0.95 (t, J = 7.5Hz, 3H), 0.90 (t, J = 7.0Hz, 3H). ^{13}C NMR (126 MHz, $CDCl_3$) δ 192.43, 133.09, 130.61, 128.62, 120.22, 91.06, 87.07, 56.24, 30.92, 29.43, 24.67, 22.75, 13.92, 11.72. HRMS (EI) calculated for $C_{16}H_{19}O$ $[M]^+$ 227.143, found 227.1433.

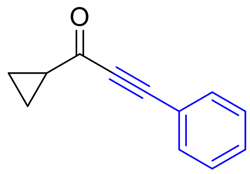

1-Cyclopropyl-3-phenylprop-2-yn-1-one (14l). Following the general procedure III, the tittle compound **14l** (28.6 mg) was obtained in 84% yield. ^{1}H NMR (400 MHz, $CDCl_3$) δ 7.57 – 7.55 (m, 2H), 7.47 – 7.43 (m, 1H), 7.40 – 7.36 (m, 2H), 2.16 (tt, *J* = 8.0 Hz, 4.6 Hz, 1H), 1.35 – 1.31 (m, 2H), 1.12 – 1.07 (m, 2H). ^{13}C NMR (126 MHz, $CDCl_3$) δ 188.38, 132.98, 130.62, 128.63, 120.04, 90.44, 86.19, 24.61, 11.15. HRMS (EI) calculated for $C_{12}H_{10}O$ $[M]^+$ 170.0726, found 170.0722.

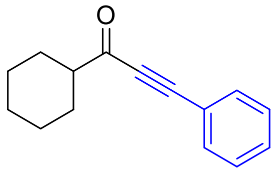

1-Cyclohexyl-3-phenylprop-2-yn-1-one (14m). Following the general procedure III, the tittle compound **14m** (33.9 mg) was obtained in 80% yield. ^{1}H NMR (500 MHz, $CDCl_3$) δ 7.59 – 7.57 (m, 2H), 7.47 – 7.43 (m, 1H), 7.40 – 7.36 (m, 2H), 2.51 (tt, *J* = 11.2 Hz, 3.6 Hz, 1H), 2.08 – 2.03 (m, 2H), 1.84 – 1.79 (m, 2H), 1.71 – 1.67 (m, 1H), 1.54 – 1.46 (m, 2H), 1.39 – 1.23 (m, 3H). ^{13}C NMR (126 MHz, $CDCl_3$) δ 191.54, 133.03, 130.57, 128.61, 120.22, 91.36, 87.24, 52.34, 28.34, 25.83, 25.44. HRMS (EI) calculated for $C_{15}H_{15}O$ $[M]^+$ 211.1117, found 211.1118.

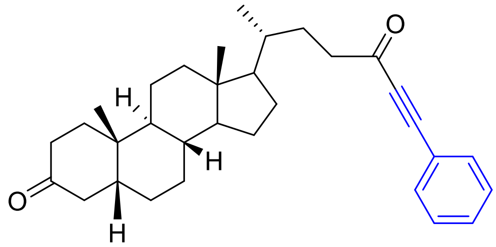

(5*R*,8*R*,9*S*,10*S*,13*R*,14*S*,17*R*)-10,13-Dimethyl-17-((*R*)-5-oxo-7-phenylhept-6-yn-2-yl)hexadecahydro-3H-cyclopenta[a]phenanthren-3-one (14n). Following the general procedure III, the tittle compound **14n** (66.8 mg) was obtained in 73% yield. ^{1}H NMR (500 MHz, $CDCl_3$) δ 7.58 – 7.56 (m, 2H), 7.47 – 7.44 (m, 1H), 7.40 – 7.37 (m, 2H), 2.73 – 2.66 (m, 2H), 2.62 – 2.56 (m, 1H), 2.36 – 2.30 (m, 1H), 2.18 – 2.14 (m, 1H), 2.05 – 2.01 (m, 3H), 1.95 – 1.70 (m, 4H), 1.63 – 1.61 (m, 1H), 1.52 – 1.33 (m, 12H), 1.13 – 1.07 (m, 3H), 1.01 (s, 3H), 0.96 (d, *J* = 6.2 Hz, 3H), 0.69 (s, 3H). ^{13}C NMR (126 MHz, $CDCl_3$) δ 212.45, 188.65, 133.03, 130.66, 128.64, 120.09, 90.62, 87.64, 56.46, 55.99, 44.33, 42.83, 42.60, 42.38, 40.75, 40.07, 37.24, 37.03, 35.55, 35.27, 24.90, 30.17, 29.72, 28.20, 26.63, 25.79, 24.18, 22.67, 21.21, 18.47, 12.12. HRMS ESI $[M+H]^+$ calculated for $C_{32}H_{43}O_2$ 459.3258, found 459.3258.

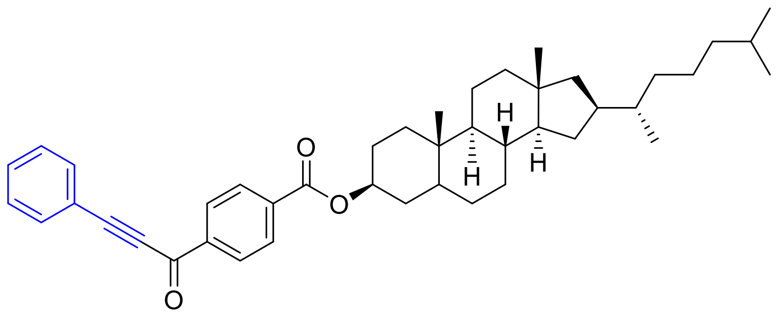

(3*S*,8*S*,9*S*,10*S*,13*R*,14*S*)-10,13-Dimethyl-16-((*S*)-6-methylheptan-2-yl)hexadecahydro-1H-cyclopenta[a]phenanthren-3-yl 4-(3-phenylpropioloyl)benzoate (14o). Following the general procedure III, the tittle compound **14o** (36.7 mg) was obtained in 32% yield. ^{1}H NMR (400 MHz, $CDCl_3$) δ 8.27 – 8.25 (m, 2H), 8.17 – 8.16 (m, 2H), 7.71 – 7.69 (m, 2H), 7.53 – 7.49 (m, 1H), 7.46 – 7.43 (m, 2H), 5.01 – 4.94 (m, 1H), 2.00 – 1.95 (m, 2H), 1.81 – 1.65 (m, 5H), 1.61 – 1.49 (m, 6H), 1.34 – 1.25 (m, 9H), 1.17 – 0.98 (m, 9H), 0.91 (d, J = 6.6 Hz, 3H), 0.88 (s, 3H), 0.87 (d, J = 2.3 Hz, 3H), 0.86 (d, J = 2.3 Hz, 3H), 0.66 (s, 3H). ^{13}C NMR (126 MHz, $CDCl_3$) δ 177.35, 165.20, 139.76, 135.58, 133.21, 129.78, 128.79, 119.88, 94.14, 86.87, 75.17, 56.44, 56.29, 54.25, 44.73, 42.62, 40.00, 39.53, 36.79, 36.18, 35.82, 35.54, 35.51, 34.09, 32.02, 28.65, 28.26, 28.03, 27.56, 24.23, 23.85, 22.84, 22.58, 21.25, 18.59, 12.32, 12.10. HRMS ESI $[M+H]^+$ calculated for $C_{43}H_{57}O_3$ 621.4302, found 621.43.

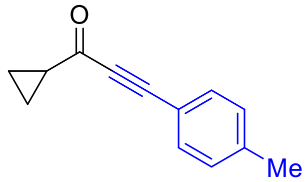

1-Cyclopropyl-3-(p-tolyl)prop-2-yn-1-one (14p). Following the general procedure III, the tittle compound **14p** (16.6 mg) was obtained in 45% yield. ^{1}H NMR (500 MHz, $CDCl_3$) δ 7.45 (d, J = 8.1 Hz, 2H), 7.18 (d, J = 8.0 Hz, 2H), 2.38 (s, 3H), 2.15 (tt, J = 7.9 Hz, 4.6 Hz, 1H), 1.33 – 1.30 (m, 2H), 1.10 – 1.06 (m, 2H). ^{13}C NMR (126 MHz, $CDCl_3$) δ 188.47, 141.31, 133.02, 129.42, 116.92, 91.11, 86.02, 24.56, 21.74, 11.08. HRMS (EI) calculated for $C_{13}H_{12}O$ $[M]^+$ 184.0883, found 184.0879.

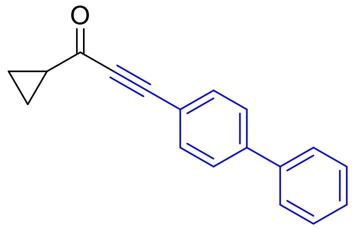

3-([1,1'-Biphenyl]-4-yl)-1-cyclopropylprop-2-yn-1-one (14q). Following the general procedure III, the tittle compound **14q** (20.7 mg) was obtained in 42% yield. ^{1}H NMR (500 MHz, $CDCl_3$) δ 7.65 – 7.59 (m, 6H), 7.48 – 7.45 (m, 1H), 7.41 – 7.37 (m, 2H), 2.19 (tt, J = 7.9 Hz, J_2 = 4.6 Hz, 1H), 1.36 – 1.33 (m, 2H), 1.13 – 1.09 (m, 2H). ^{13}C NMR (126 MHz, $CDCl_3$) δ 188.37, 143.44, 139.82, 133.50, 128.99, 128.18, 127.29, 127.14, 118.76, 90.51, 86.88, 24.62, 11.20. HRMS (EI) calculated for $C_{18}H_{14}O$ $[M]^+$ 246.1039, found 246.105.

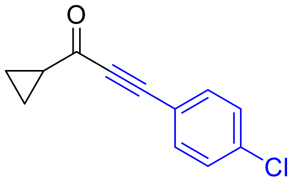

3-(4-Chlorophenyl)-1-cyclopropylprop-2-yn-1-one (14r). Following the general procedure III, the tittle compound **14r** (22.4 mg) was obtained in 55% yield. ^{1}H NMR (500 MHz, $CDCl_3$) δ 7.50 – 7.47 (m, 2H), 7.37 – 7.35 (m, 2H), 2.16 (tt, J = 7.9 Hz,4.6 Hz, 1H), 1.33 – 1.1.29 (m, 2H), 1.12 – 1.08 (m, 2H). ^{13}C NMR (126 MHz, $CDCl_3$) δ 188.11, 137.01, 134.15, 129.10, 118.51, 88.95, 86.99, 24.58, 11.29. HRMS (EI) calculated for $C_{12}H_9ClO$ [M]$^+$ 204.0336, found 204.0333.

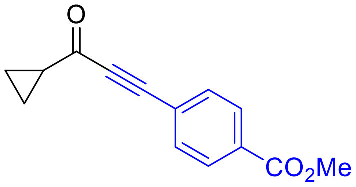

Methyl 4-(3-cyclopropyl-3-oxoprop-1-yn-1-yl)benzoate (14s). Following the general procedure III, the tittle compound **14s** (22.8 mg) was obtained in 50% yield. ^{1}H NMR (500 MHz, $CDCl_3$) δ 8.05 – 8.03 (m, 2H), 7.62 – 7.61 (m, 2H), 3.94 (s, 3H), 2.18 (tt, J = 8.0 Hz,4.6 Hz, 1H), 1.34 – 1.32 (m, 2H), 1.13 – 1.11 (m, 2H). ^{13}C NMR (126 MHz, $CDCl_3$) δ 188.02, 166.11, 132.75, 131.62, 129.66, 124.55, 88.63, 87.99, 52.46, 24.66, 11.38. HRMS (EI) calculated for $C_{14}H_{12}O_3$ [M]$^+$ 228.0781, found 228.0784.

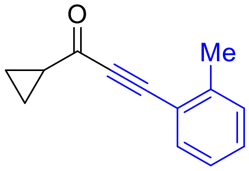

1-Cyclopropyl-3-(o-tolyl)prop-2-yn-1-one (14t). Following the general procedure III, the tittle compound **14t** (26.5 mg) was obtained in 72% yield. ^{1}H NMR (400 MHz, $CDCl_3$) δ 7.53 – 7.51 (m, 1H), 7.36 – 7.32 (m, 1H), 7.26 – 7.17 (m, 2H), 2.49 (s, 3H), 2.16 (tt, J = 8.0 Hz, 4.6 Hz, 1H), 1.36 – 1.32 (m, 2H), 1.13 – 1.08 (m, 2H). ^{13}C NMR (126 MHz, $CDCl_3$) δ 188.46, 141.95, 133.56, 130.66, 129.80, 125.89, 119.87, 89.84, 89.49, 24.71, 20.68, 11.04. HRMS (EI) calculated for $C_{13}H_{12}O$ [M]$^+$ 184.0883, found 184.0877.

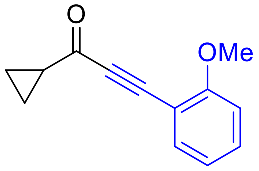

1-Cyclopropyl-3-(2-methoxyphenyl)prop-2-yn-1-one (14u). Following the general procedure III, the tittle compound **14u** (20.8 mg) was obtained in 52% yield. ^{1}H NMR (500 MHz, $CDCl_3$) δ 7.49 (dd, J = 7.6 Hz, 1.8 Hz, 1H), 7.42 – 7.39 (m, 1H), 6.95 – 6.89 (m, 2H), 3.90 (s, 3H), 2.13 (tt, J = 7.9 Hz,4.6 Hz, 1H), 1.40 – 1.36 (m, 2H), 1.10 – 1.06 (m, 2H). ^{13}C NMR (126 MHz, $CDCl_3$) δ 188.74, 161.52, 134.85, 132.36, 120.59, 110.87, 109.30, 89.93, 87.30, 55.81, 24.78, 10.92. HRMS (EI) calculated for $C_{13}H_{12}O_2$ [M]$^+$ 200.0832, found

200.0827.

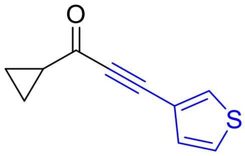

1-Cyclopropyl-3-(thiophen-3-yl)prop-2-yn-1-one (14v). Following the general procedure III, the tittle compound **14v** (29.2 mg) was obtained in 51% yield. ^{1}H NMR (500 MHz, $CDCl_3$) δ 7.73 – 7.72 (m, 1H), 7.34 – 7.32 (m, 1H), 7.21 – 7.20 (m, 1H), 2.15 (tt, *J* = 7.9 Hz, 4.6 Hz, 1H), 1.32 – 1.29 (m, 2H), 1.10 – 1.06 (m, 2H). ^{13}C NMR (126 MHz, $CDCl_3$) δ 188.32, 133.59, 130.24, 126.16, 119.30, 86.51, 85.78, 24.47, 11.17. HRMS (EI) calculated for $C_{10}H_8OS$ $[M]^+$ 176.029, found 176.0287.

Procedure IV: neutral-eosin Y-photocatalyzed aldehydic C–H azidation

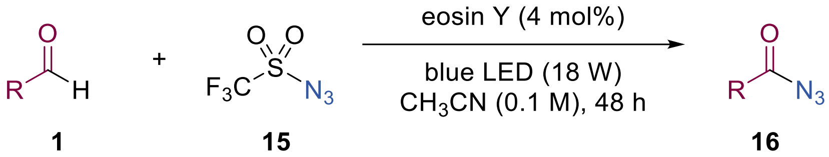

A 20 mL Schlenk tube equipped with a magnetic stir bar was charged with eosin Y (0.008 mmol, 5.2 mg), aldehyde **1** (0.4 mmol), and triflic azide **15** (0.2 mmol). Then, 2.0 mL of anhydrous acetonitrile was added. The Schlenk tube was connected to Schlenk line and freeze-pump-thaw was performed for three times to completely remove air inside the reaction mixture. Eventually the Schlenk tube was refilled with an atmosphere of argon at room temperature and sealed. The reaction vessel was surrounded by a coil of blue LED strip (2 meters, 18 W). Then the reaction was running at ambient temperature (~27 °C) using a fan to cool down the reaction mixture and stopped after 48 h. The solvent was removed under reduced pressure and the crude mixture was purified by silica gel column chromatography (eluent: hexane/diethyl ether 10/1 – 3/1) to give the corresponding product **16**.

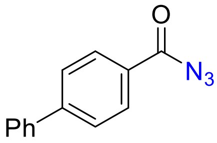

[1,1'-Biphenyl]-4-carbonyl azide (16a). Following the procedure IV, the title compound (25.9 mg) was obtained in 58% yield. ^{1}H NMR (400 MHz, $CDCl_3$) δ 8.10 (d, *J* = 8.6 Hz, 2H), 7.68 (d, *J* = 8.6 Hz, 2H), 7.65 – 7.60 (m, 2H), 7.51 – 7.45 (m, 2H), 7.42 (dt, *J* = 9.6, 4.3 Hz, 1H). ^{13}C NMR (126 MHz, $CDCl_3$) δ 172.30, 147.12, 139.65, 130.05, 129.40, 129.02, 128.49, 127.33 (2C). HRMS ESI $[M+H]^+$ calculated for $C_{13}H_{10}N_3O$ 224.0818, found 224.0812.

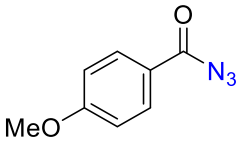

4-Methoxybenzoyl azide (16b).[15] Following the procedure IV, the title compound (12.9 mg) was obtained in 55% yield. ^{1}H NMR (400 MHz, $CDCl_3$) δ 7.99 (d, *J* = 9.0 Hz, 2H), 6.93 (d, *J* = 9.0 Hz, 2H), 3.88 (s, 3H). ^{13}C NMR (126 MHz, $CDCl_3$) δ 171.84, 164.76, 131.89, 123.37,

114.09, 55.71. HRMS ESI $[M+H]^+$ calculated for $C_8H_8N_3O_2$ 178.0611, found 178.0607. HRMS (ESI) $[M+Na]^+$ calculated for $C_8H_7N_3O_2Na$ 200.0430. found 200.0432. HRMS (EI) calculated for $C_8H_7N_3O_2$ $[M]^+$ 177.0533, found 177.0534.

Procedure V: neutral-eosin Y-photocatalyzed aldehydic C–H alkenylation

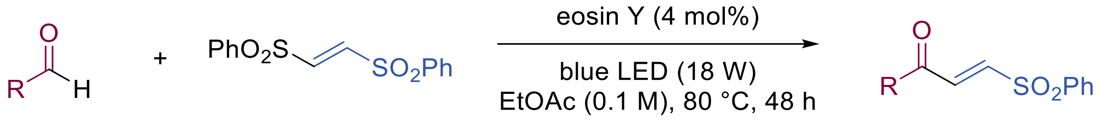

1 **17** **18**

A 20 mL Schlenk tube equipped with a magnetic stir bar was charged with eosin Y (0.008 mmol, 5.2 mg), aldehyde **1** (0.4 mmol), and (*E*)-1,2-bis(phenylsulfonyl)ethene **17** (0.2 mmol). Then, 2.0 mL of anhydrous ethyl acetate (EtOAc) was added. The Schlenk tube was connected to Schlenk line and freeze-pump-thaw was performed for three times to completely remove air inside the reaction mixture. Eventually the Schlenk tube was refilled with an atmosphere of argon at room temperature and sealed. The reaction vessel was surrounded by a coil of blue LED strip (2 meters, 18 W). Then the reaction tubes were placed in a water bath covered by top oil layer (to prevent evaporation of water bath). The reaction was running at 80 °C and stopped after 48 h. The solvent was removed under reduced pressure and the crude mixture was purified by silica gel column chromatography (eluent: hexane/diethyl ether 5/1 – 3/1) to give the corresponding product **18**.

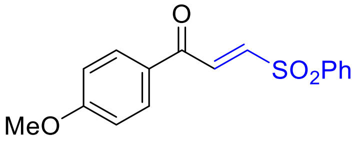

(*E*)-1-(4-Methoxyphenyl)-3-(phenylsulfonyl)prop-2-en-1-one (18). Following the procedure V, the title compound (24.2 mg) was obtained in 40% yield. ^1H NMR (400 MHz, $CDCl_3$) δ 8.02 – 7.90 (m, 5H), 7.71 – 7.65 (m, 1H), 7.59 (dd, *J* = 8.2, 6.9 Hz, 2H), 7.33 (d, *J* = 14.8 Hz, 1H), 6.99 (d, *J* = 9.0 Hz, 2H), 3.90 (s, 3H). ^{13}C NMR (126 MHz, $CDCl_3$) δ 185.85, 164.87, 141.41, 138.98, 134.40, 133.51, 131.67, 129.76, 129.29, 128.39, 114.50, 55.82. HRMS ESI $[M+H]^+$ calculated for $C_{16}H_{15}O_4S$ 303.0686, found 303.0681. HRMS (EI) calculated for $C_{16}H_{14}O_4S$ $[M]^+$ 302.0607, found 302.0618.

VI. Mechanistic studies

Evaluation of different photo HAT catalysis systems

The examination on the effectiveness of different hydrogen atom transfer (HAT)-based photocatalytic systems was shown in Supplementary Table 4. Neutral eosin Y as a direct HAT photocatalyst promote the current reaction most effectively (entry 1). Anionic form of eosin Y (Na_2 eosin Y) resulted in negligible conversion of **2a** (entry 2). Catalytic systems consisting of a photoredox catalyst and a HAT agent were less effective (entries 3-9). Other direct HAT photocatalysts gave lower yields (entries 10-16) or no product (entries 17-18). These results highlight the photo HAT catalytic efficiency of neutral eosin Y.

Activation of benzophenones and quinones normally requires ultraviolet (UV) light, while eosin Y is inexpensive and commercial-available, with maximum absorption at visible-light region, which features a long lifetime of triplet excited state owing to heavy atom (bromine) effect. Furthermore, the formed eosinY-H species is a relatively stable radical due to both captodative and steric effects, which avoids side reactions and enables an effective RHAT process.

Supplementary Table 4. Control experiments using other photo HAT catalytic systems.

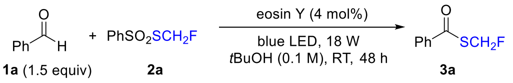

entry	catalyst (mol%) and condition deviation	conv. of **2a** (%)	yield of **3a** (%)[a]
1	**eosin Y (4)**	**100**	**91(88)**[b]
2	Na_2eosin Y (4)	10	0
3	Mes-Arc$^+$$ClO_4$ (2)+HCl (5)	60	17
4	[Ir(dF(CF_3)ppy)$_2$(dtbbpy)]PF_6 (2) + 2-I-PhCO$_2$Na(10)	86	43
5	4-CzIPN (2) + ***HAT cat. 1*** (10)	73	41
6	[Ir(dF(CF_3)ppy)$_2$(dtbbpy)]PF_6 (2) + ***HAT cat. 2*** (10)	93	50
7	[Ir(dF(CF_3)ppy)$_2$(dtbbpy)]PF_6 (2) + *i*Pr$_3$SiSH (10)	95	62
8	Ir(dF(CF_3)ppy)$_2$(dtbbpy)]PF_6 (2) + PhCO$_2$Na (5)	100	60
9	[Ir(ppy)$_2$(dtbpy)]PF_6 (2) + HSCH$_2$CO$_2$Et (20) +LiOAc (10)	100	30
10	TBADT (4); under 365 nm LED	100	50
11	***HAT cat. 3*** (10); under 370 nm LED	100	40
12	***HAT cat. 4*** (10); under 370 nm LED	100	48
13	***HAT cat. 5*** (10); under 370 nm LED	100	60
14	***HAT cat. 6*** (10); under 370 nm LED	100	62
15	***HAT cat. 7*** (10); under blue LED	100	80
16	***HAT cat. 8*** (10); under white LED	100	77
17	PhCOCOOH (10); under CFL	53	0
18	*N*-hydroxyphthalimide (10); under CFL	65	0

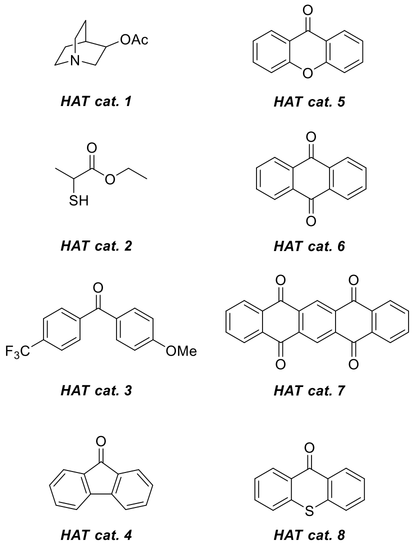

[a]Yields based on analysis of the crude ^{1}H NMR spectra using dibromomethane as an internal standard.
[b]Isolated yields.

Study on site-selectivity of aldehydic C–H fluoromethylthiolation

To evaluate the site-selectivity of aldehydic C-H functionalization, the substrate **S1** containing one aliphatic and one aromatic aldehyde moieties has been synthesized. **S1** was subjected to the standard conditions, fluoromethylthiolation of both aliphatic and aromatic aldehydic C-H bonds occurred to give a major product **S2** in 32% yield and a minor product **S3** in 16% yield (Supplementary Figure 3A). Under standard conditions, competitive fluoromethylthiolation of benzaldehyde and 3-phenylpropanal was also performed (Supplement Figure 3B). The reaction gave both *S*-(fluoromethyl) benzothioate **3a** (26% yield) and *S*-(fluoromethyl) 3-phenylpropanethioate **3p** (66% yield).

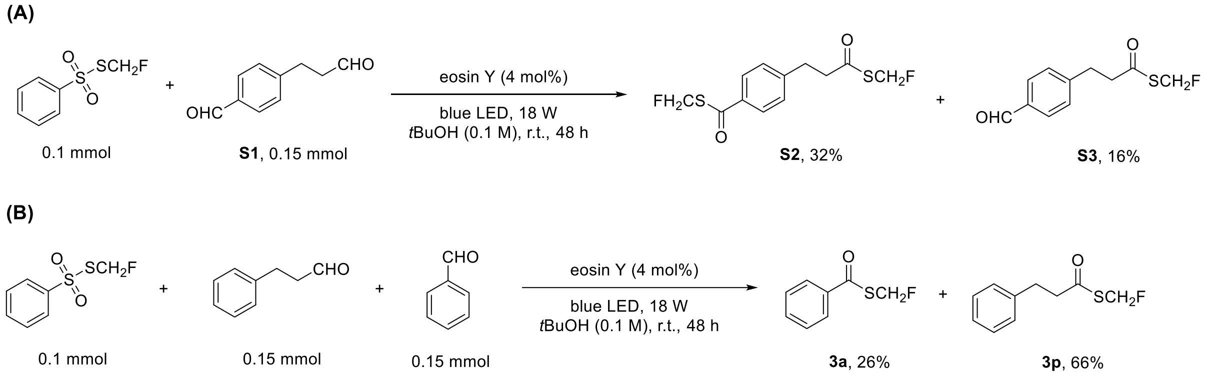

Supplementary Figure 3. Competition of aliphatic- and aromatic aldehydic C–H bonds in the fluoromethylthiolation reaction.

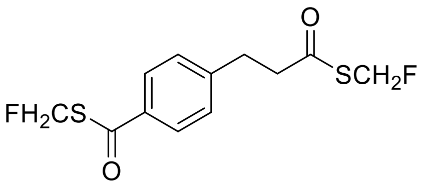

S-(fluoromethyl) 4-(3-((fluoromethyl)thio)-3-oxopropyl)benzothioate **(S2)**. ^{1}H NMR (400 MHz, $CDCl_3$) δ 7.93 (d, *J* = 8.4 Hz, 2H), 7.32 (d, *J* = 8.4 Hz, 2H), 5.99 (d, *J* = 50.2 Hz, 2H), 5.80 (d, *J* = 50.1 Hz, 2H), 3.12 – 3.07 (m, 2H), 3.02 – 2.98 (m, 2H). ^{13}C NMR (126 MHz, $CDCl_3$) δ 194.30, 187.46, 146.58, 134.41, 128.91, 128.23, 81.55 (d, *J* = 4.7 Hz), 79.84 (d, *J* = 5.0 Hz), 44.96, 30.75. ^{19}F NMR (377 MHz, $CDCl_3$) δ -191.98, -192.39. HRMS (EI) calculated for $C_{11}H_{10}FO_2S_2$ $[M-CH_2F]^+$ 257.0101, found 257.0099.

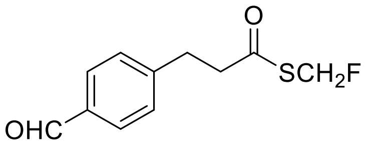

S-(fluoromethyl) 3-(4-formylphenyl)propanethioate **(S3)**. ^{1}H NMR (500 MHz, $CDCl_3$) δ 9.97 (s, 1H), 7.82 (d, *J* = 8.2 Hz, 2H), 7.36 (d, *J* = 8.1 Hz, 2H), 5.80 (d, *J* = 50.0 Hz, 2H), 3.12 – 3.08 (m, 2H), 3.02 – 2.99 (m, 2H). ^{13}C NMR (126 MHz, $CDCl_3$) δ 194.35, 191.83, 146.72, 135.06, 130.17, 129.07, 80.72 (d, *J* = 215.1 Hz), 45.01, 30.88. ^{19}F NMR (377 MHz, $CDCl_3$) δ -192.36. HRMS (APCI) calculated for $C_{11}H_{12}FO_2S$ $[M]^+$ 227.0537, found 227.0545.

Control experiments to elucidate the reaction intermediates in arylthiolation

To gain mechanistic understanding of the aldehydic C–H arylthiolation, control experiments were performed to elucidate possible intermediates (Supplementary Figure 4) besides those described in Fig. 5. Under otherwise same conditions described in general procedure II, the reaction of 4-methyl-benzenesulfinic acid **5b** and 4-methoxylbenzaldehye **1b** was stopped earlier (reaction time was decreased from 48 h to 24 h) and substantial amount of 4-methylphenylthiosulfonate **9b** was obtained in 30% yield (Supplement Figure 4A). 4-Methyl-benzenesulfinic acid **5b** can produce 4-methylphenylthiosulfonate **9b** under the general eosin Y photocatalytic conditions (Supplement Figure 4B). Crude ^{1}H NMR analysis of this reaction also indicates the presence of disulfone **10b** and 4-methyl-benzenesulfinic acid **6b** (Supplementary Figure 5 and Supplementary Figure 6).

(A)

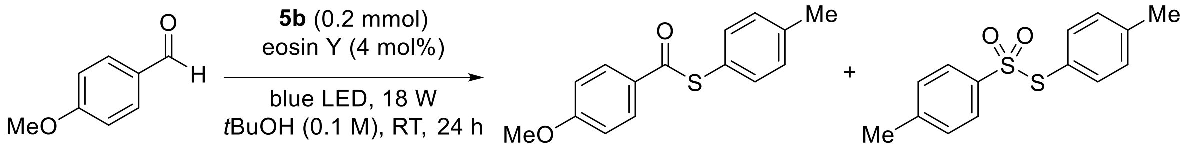

1b (0.4 mmol) **4c**, 43% **9b**, 30%

(B)

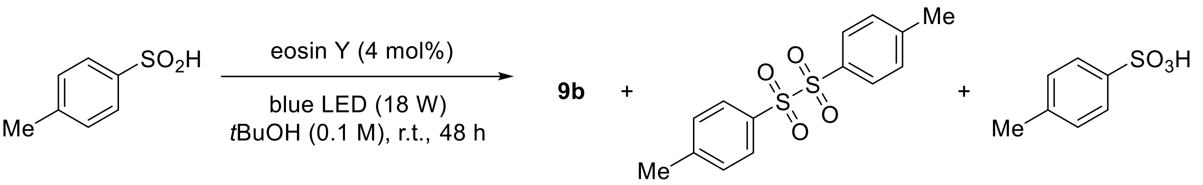

5b (0.2 mmol) 19% **10b**, 4% **6b**, 24%

Supplementary Figure 4. Elucidation of possible intermediates during aldehydic C–H arylthiolation.

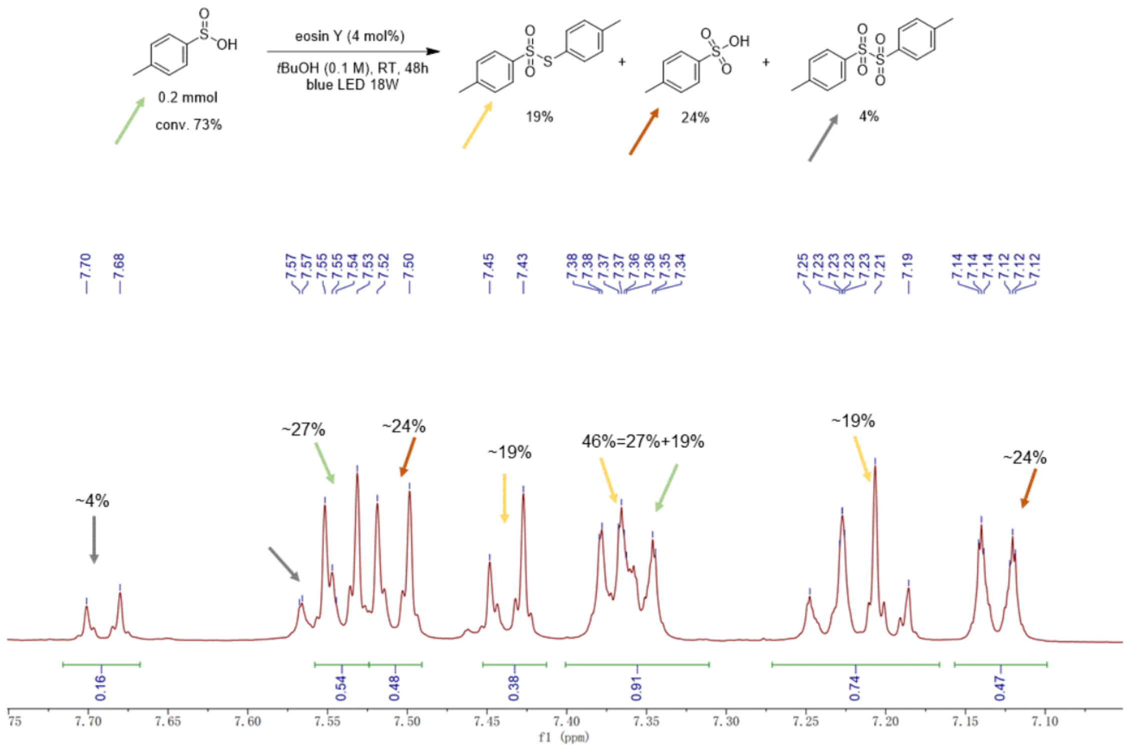

Supplementary Figure 5. Crude ^{1}H NMR analysis of reaction in Supplementary Figure 4B.

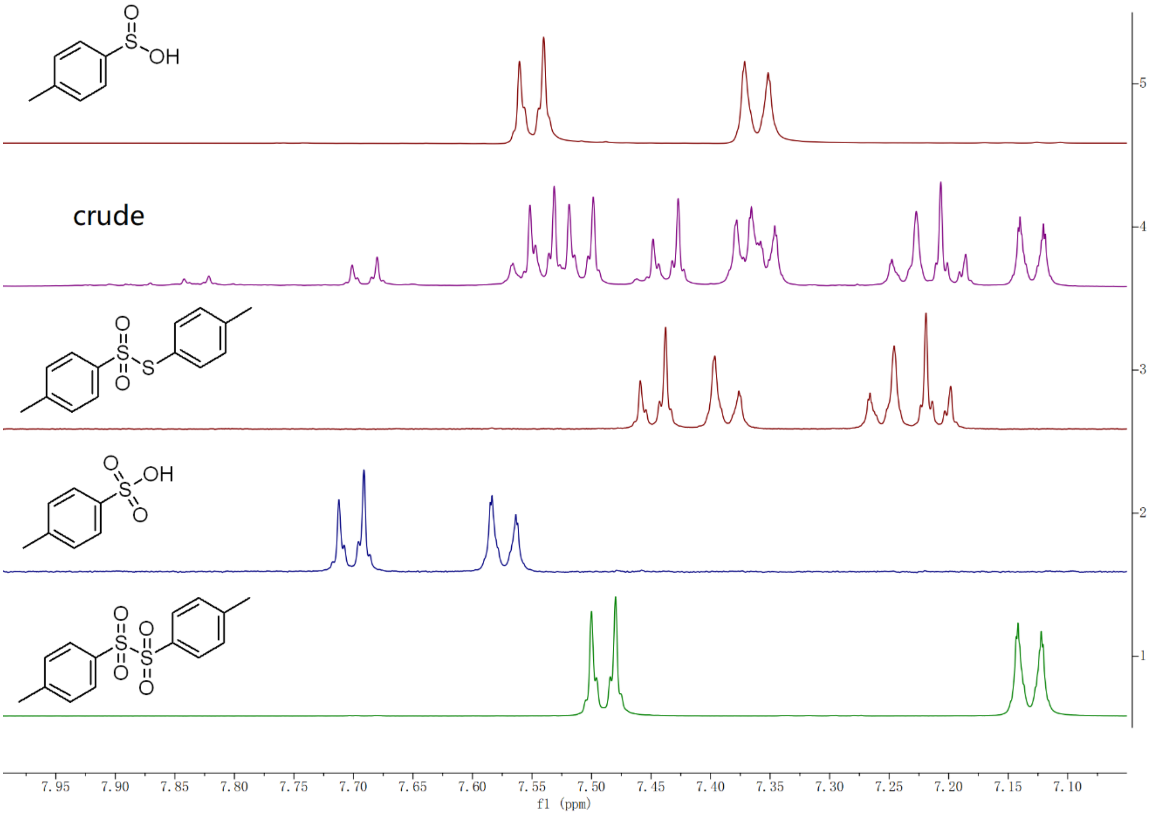

Supplementary Figure 6. ^{1}H NMR spectra of crude product mixture (for reaction in Supplementary Figure 4B) and authentic samples.

Following the general procedure II, the aldehydic C–H thiolation reaction with decreased reaction time (48 h to 24 h, Supplementary Figure 4A) afforded **4c** (21.0 mg) in 43% yield, together with intermediate **9b** (16.7 mg) in 30% yield. The spectra data of **4c**[16] (as described *vide supra*) and **9b**[17] are in line with literature.

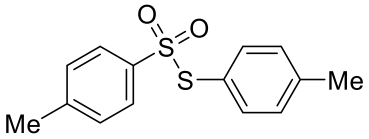

***S*-(*p*-tolyl) 4-methylbenzenesulfonothioate (9b)**. ^{1}H NMR (400 MHz, $CDCl_3$) δ 7.46 (d, *J* = 8.4 Hz, 2H), 7.25 – 7.19 (m, 4H), 7.14 (d, *J* = 7.9 Hz, 2H), 2.42 (s, 3H), 2.38 (s, 3H). ^{13}C NMR (126 MHz, $CDCl_3$) δ 144.71, 142.18, 140.63, 136.65, 130.35, 129.51, 127.76, 124.75, 21.81, 21.63. HRMS ESI $[M+H]^+$ calculated for $C_{14}H_{15}O_2S_2$ 279.0508, found 279.0503.

Following the general procedure II, the aldehydic C–H thiolation reactions with $PhSO_2H$ **5a** and $PhSO_2SPh$ **9a** (entries 1 and 5, Fig. 5a) afforded **4b** (33.2 mg and 32.7 mg, 68% and 67%, respectively). The spectra data of **4b**[18] is in line with literature.

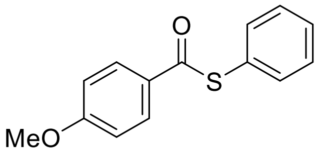

***S*-phenyl 4-methoxybenzothioate (4b)**. ^{1}H NMR (400 MHz, $CDCl_3$) δ 8.02 (d, *J* = 9.0 Hz, 2H), 7.55 – 7.50 (m, 2H), 7.49 – 7.41 (m, 3H), 6.97 (d, *J* = 9.0 Hz, 2H), 3.88 (s, 3H). ^{13}C NMR (126 MHz, $CDCl_3$) δ 188.76, 164.14, 135.34, 130.54, 129.86, 129.52, 129.32, 127.79, 114.06, 55.70. HRMS ESI $[M+H]^+$ calculated for $C_{14}H_{13}O_2S$ 245.0631, found 245.0627.

Following the general procedure II (in the absence of aldehyde substrate, Fig. 5b), the reaction of benzenesulfinic acid **5a** (28.4 mg, 0.2 mmol) and eosin Y (5.2 mg, 0.008 mmol) gave benzenesulfonothioate **9a** (8.0 mg) in 32% yield. Benzenesulfonic acid **6** was detected in ESI-MS of the reaction mixture (Supplementary Figure 7). By contrast, **9a** was not observed in the absence of eosin Y as the photocatalyst. The spectra data of **9a**[17] is in line with literature.

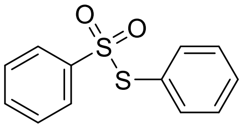

***S*-Phenyl benzenesulfonothioate (9a)**. Solid, m.p. 38–40 °C, (lit.14 41–42 °C). ^{1}H NMR (500 MHz, $CDCl_3$) δ 7.60 – 7.55 (m, 3H), 7.50 – 7.45 (m, 1H), 7.44 – 7.40 (m, 2H), 7.39 – 7.31 (m, 4H). ^{13}C NMR (126 MHz, $CDCl_3$) δ 143.12, 136.75, 133.76, 131.55, 129.58, 128.94, 128.01, 127.72. HRMS ESI $[M+H]^+$ calculated for $C_{12}H_{11}O_2S_2$ 251.0195, found 251.0191.

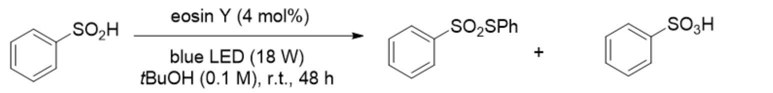

5a (0.2 mmol) **9a**, 32% (0%, w/o eosin Y) **6**, *ESI-MS* observed

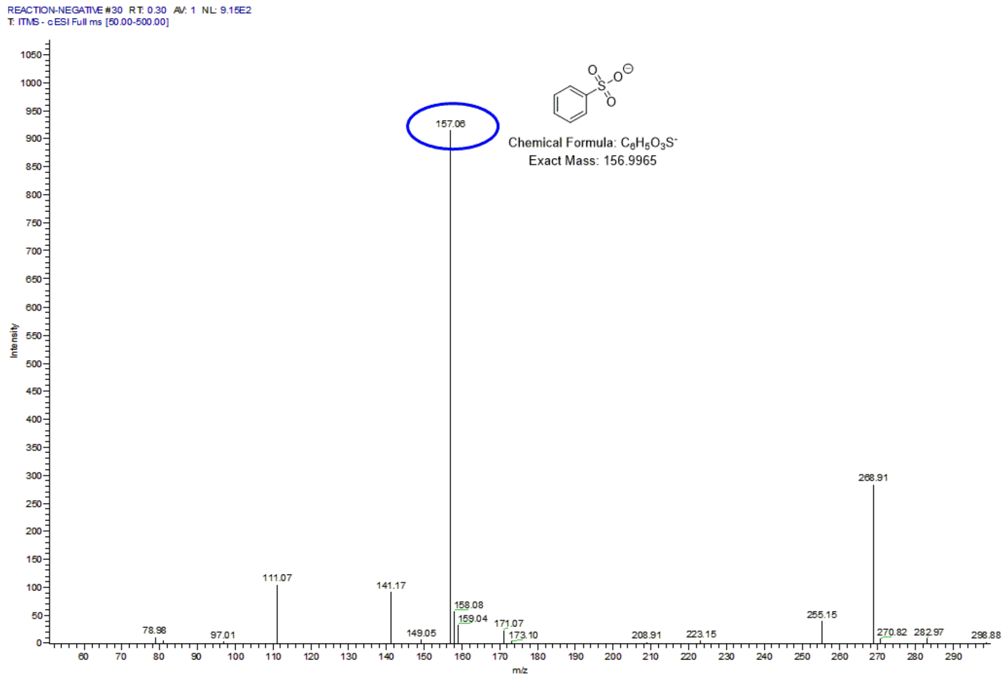

Supplementary Figure 7. ESI-MS indicating the presence of benzenesulfonic acid **6**.

Evidence for the involvement of arylsulfonyl radical and acyl radical in arylthiolation

The involvement of acyl radical and arylsulfonyl radical in arylthiolation was indicated in Supplementary Figure 8. The reaction of 4-methoxybenzaldehyde **1b** and 4-Me-C_6H_4-SO_2H **5b**, under otherwise standard conditions, was diminished in the presence of radical traps such as 2,2,6,6-tetramethylpiperidin-1-yl)oxyl (TEMPO) and butylated hydroxytoluene (BHT). Addition of radical trap 1,1-diphenylethylenen (DPE) to the reaction mixture of 4-methoxybenzaldehyde **1b** and 4-Me-C_6H_4-SO_2H **5b** suppressed the reaction and decreased the yield of **4c** to 13%, while adducts **11** and **12** were detected by electrospray ionization mass (ESI-MS) spectrometric analysis (Supplementary Figure 9), indicating the presence of arylsulfonyl radical[19]and acyl radical respectively. The presence of acyl radical is further supported by the isolation of a decarbonylated alkynylation product **14w** under the general procedure (Supplementary Figure 8C). Facile decarbonylation of pivaloyl radical to the more stable *tert*-butyl radical reasonably accounts for this observation.

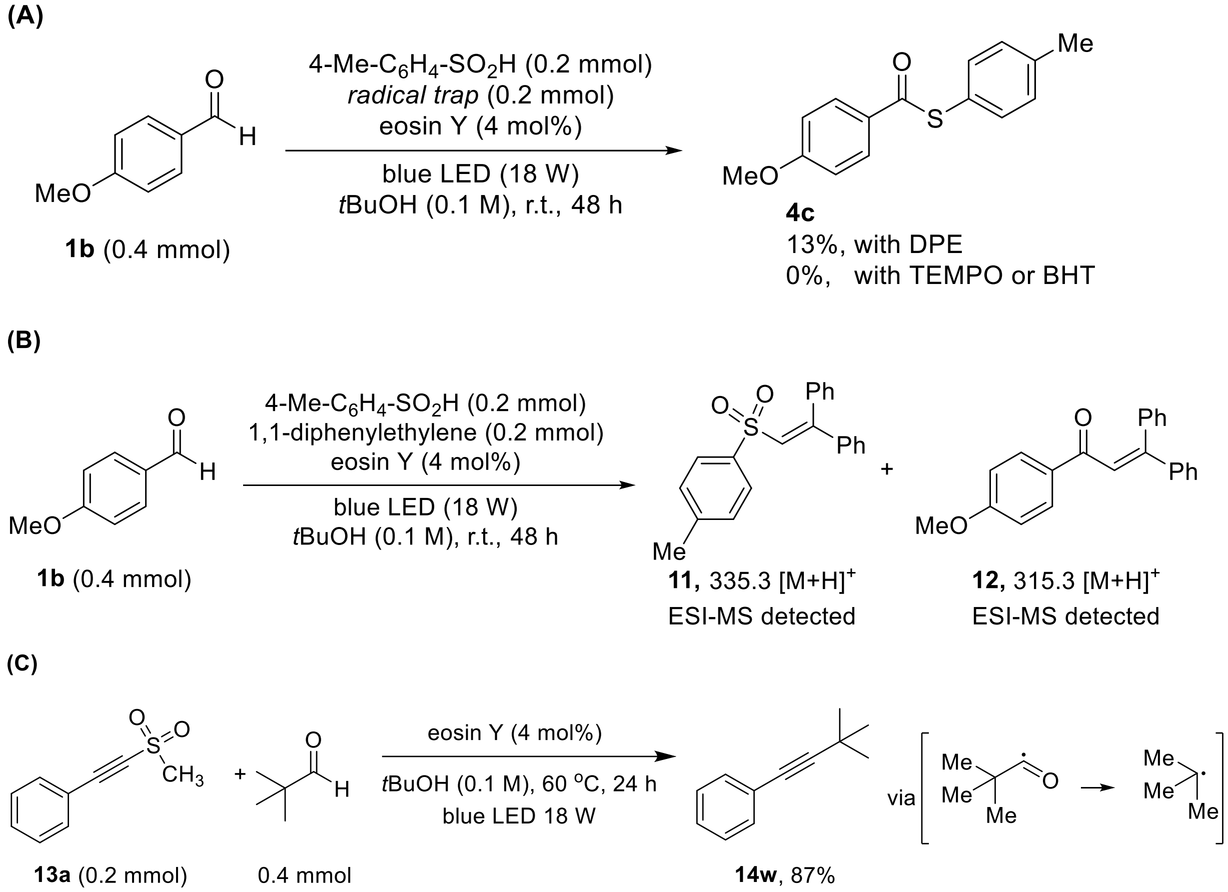

Supplementary Figure 8. Involvement of arylsulfonyl radical and acyl radical.

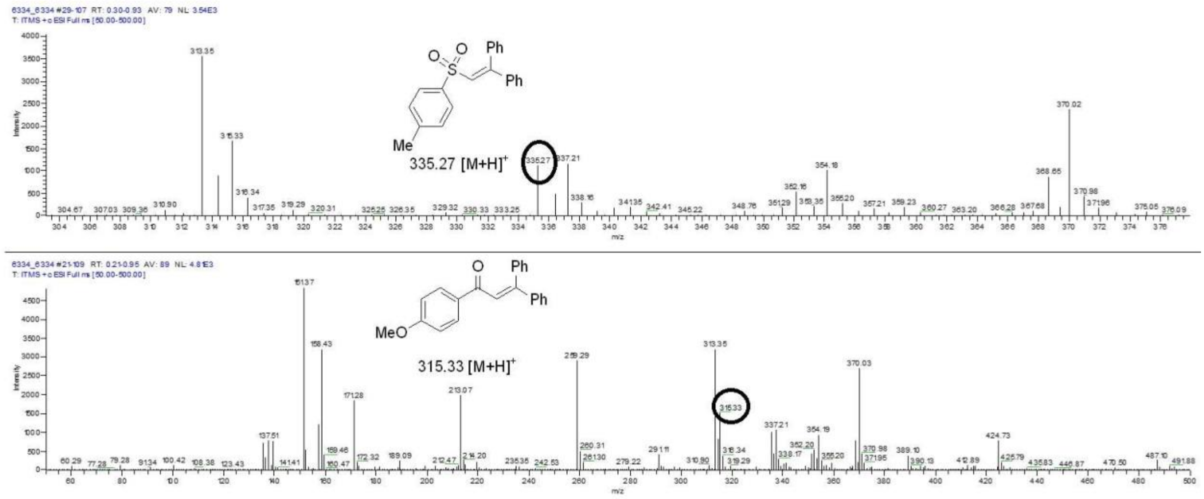

Supplementary Figure 9. Trapping arylsulfonyl radical and acyl radical by DPE.

^{18}O-labelling study

^{18}O-labeled 4-methylbenzenesulfinic acid **^{18}O-5b** was synthesized following reported literature,[20] with its structure confirmed by ESI-MS spectrometric analysis (Supplementary Figure 10). ^{18}O incorporation was not observed in product **4c** when ^{18}O-labeled 4-methylbenzenesulfinic acid **^{18}O-5b** was employed for aldehydic C–H thiolation (as indicated by $[M+H]^+$ 259.4 in ESI-MS analysis), which suggested that the carbonyl oxygen in thioester **4c** was derived from aldehyde **1b** (Supplementary Figure 11).

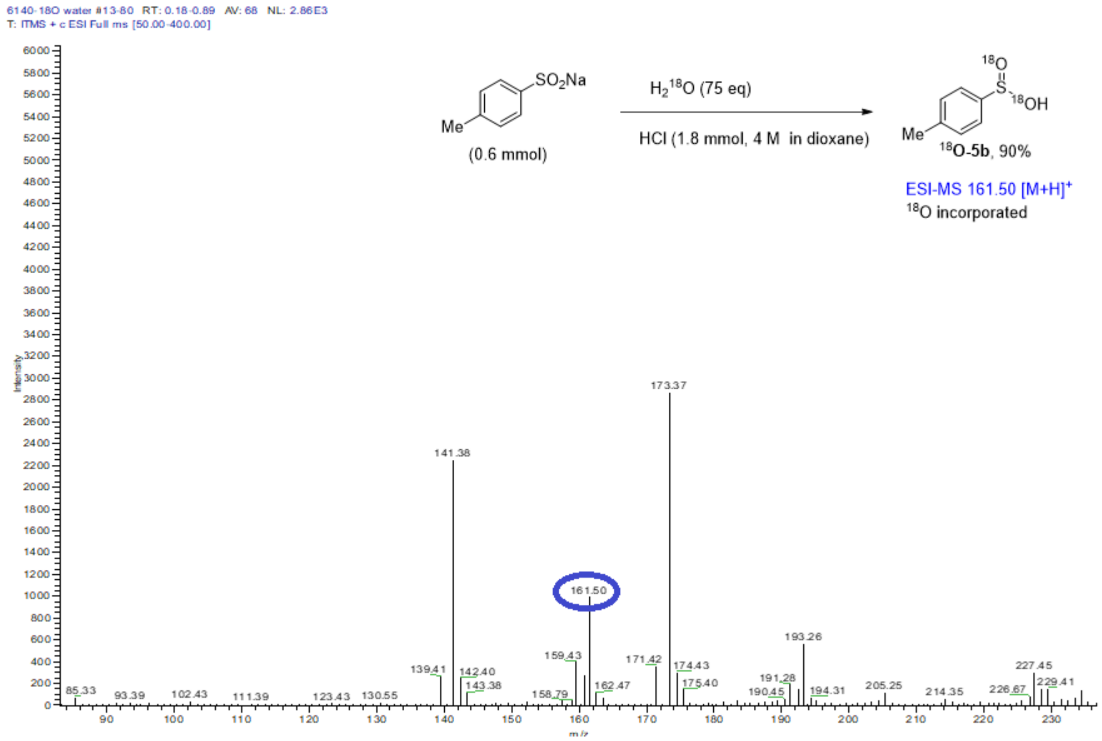

Supplementary Figure 10. Preparation of ^{18}O-labeled 4-methylbenzenesulfinic acid.

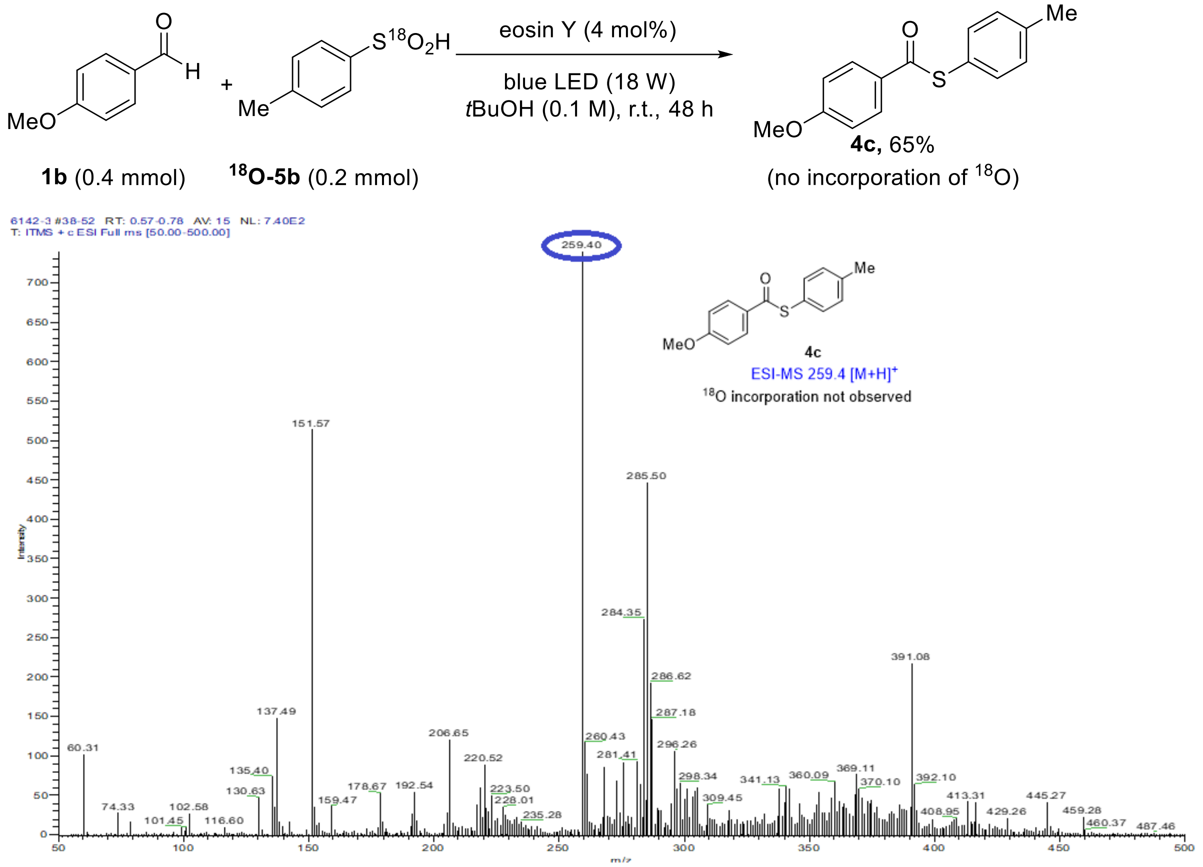

Supplementary Figure 11. Aldehydic C–H thiolation using ^{18}O-labeled 4-methylbenzenesulfinic acid.

Discussion on mechanism of eosin Y-photocatalytic aldehydic C–H arylthiolation

In light of related literature reports and our experimental data, a tentative mechanism for the aldehydic C–H arylthiolation reaction was proposed in Supplementary Figure 12. Photo-excited *eosin Y undergoes HAT with benzenesulfinic acid **5a** to generate benzenesulfonyl radical **B**[21], which dimerizes to give disulfone species **10**. **10** is reduced by benzenesulfinic acid **5a** to give thiosulfonate **9**, delivering benzenesulfonic acid **6** simultaneously.[22],[23] Next, a single electron transfer (SET) process between **6** and eosin Y-H species may complete the eosin Y-photocatalytic cycle to regenerate eosin Y photocatalyst, accompanied by the formation of disulfone species **10**. In addition, **6** could also be reduced by excess amount of aldehyde **1** with the assistance of eosin Y (the corresponding carboxylic acid as an oxidized by-product was isolated, Supplementary Figure 13A, *vide infra*) to regenerate benzenesulfinic acid **5a**.

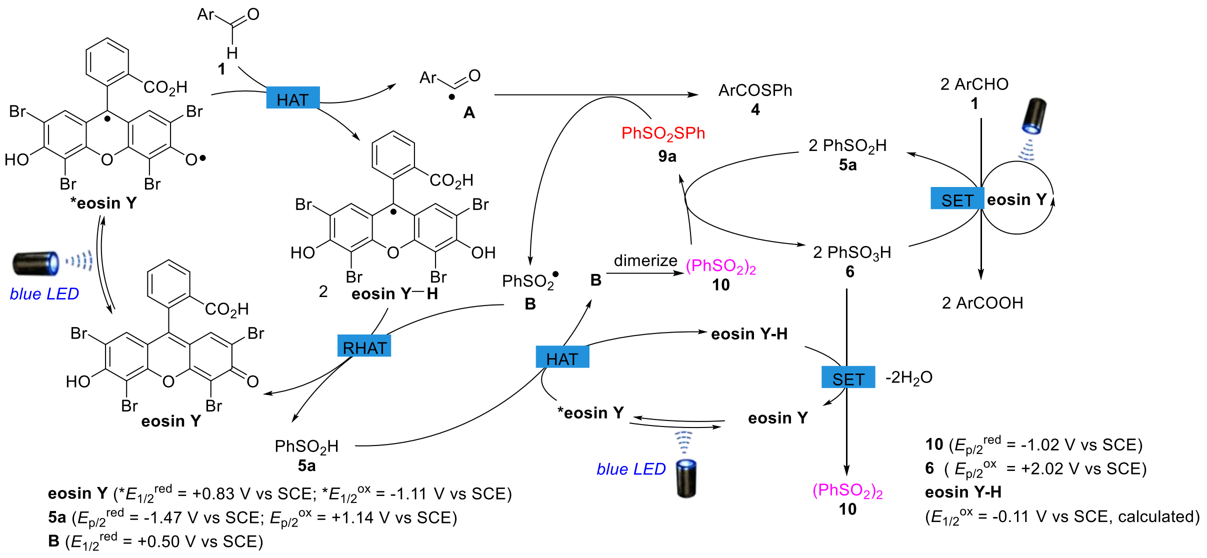

Supplementary Figure 12. Tentative mechanism for aldehydic C–H arylthiolation.

Under the standard aldehydic C–H thioarylation conditions, arylcarboxylic acid (ArCOOH) is commonly observed as a by-product. For instance, the reaction of 4-methoxybenzaldehyde **1b** and 4-methylbenzenesulfonic acid **5b,** under standard photocatalytic conditions, affords **4c** (63% yield) and 4-methoxybenzoic acid in 30% yield (Supplementary Figure 13A). We reasoned that the carboxylic originates from aldehyde, which serves as an alternative reductant to reduce sulfonic **6** (Supplementary Figure 13B, eq S3).

(A) arylcarboxylic acid was observed as the by-product in aldehydic C-H thioarylation

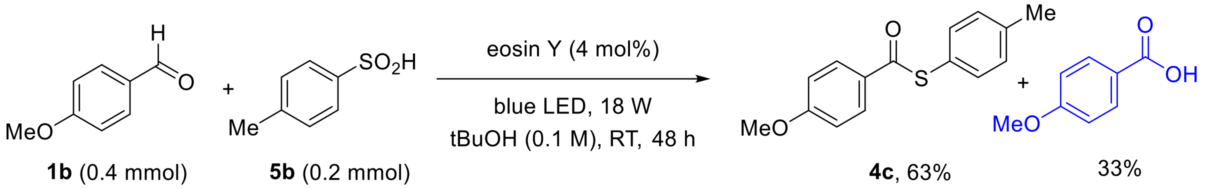

(B) Overall consideration on stoichiometric analysis

balanced equation (based on catalytic cycles in Supplementary Figure 12)

eq S1 $3\ PhSO_2H \xrightarrow{\text{eosin Y}} PhSO_2\text{-}SPh + PhSO_3H + H_2O$

ArCHO also serves as a reductant, as evidenced by the detection of ArCOOH (Supplementary Figure 13A)

eq S2 $PhSO_2\text{-}SPh + ArCHO \xrightarrow{\text{eosin Y}} ArCOSPh + PhSO_2H$

eq S3 $ArCHO + PhSO_3H \xrightarrow{\text{eosin Y}} ArCOOH + PhSO_2H$

overall equation: **eq S1 + eq S2 + eq S3**

eq S4 $2\ ArCHO + PhSO_2H \xrightarrow{\text{eosin Y}} ArCOSPh + ArCOOH + H_2O$

Supplementary Figure 13. Consideration of aldehyde as an inherent reductant.

Control experiments were performed regarding the formation of thiosulfonate **9a** from a potential intermediate **10** (Supplementary Figure 14). Under standard condition described in general procedure II, the reaction of diphenyl disulfone **10** and benzenesulfinic acid **5a** delivered thiosulfonate **9a** (26% yield) and benzenesulfonic acid **6** (36% yield).

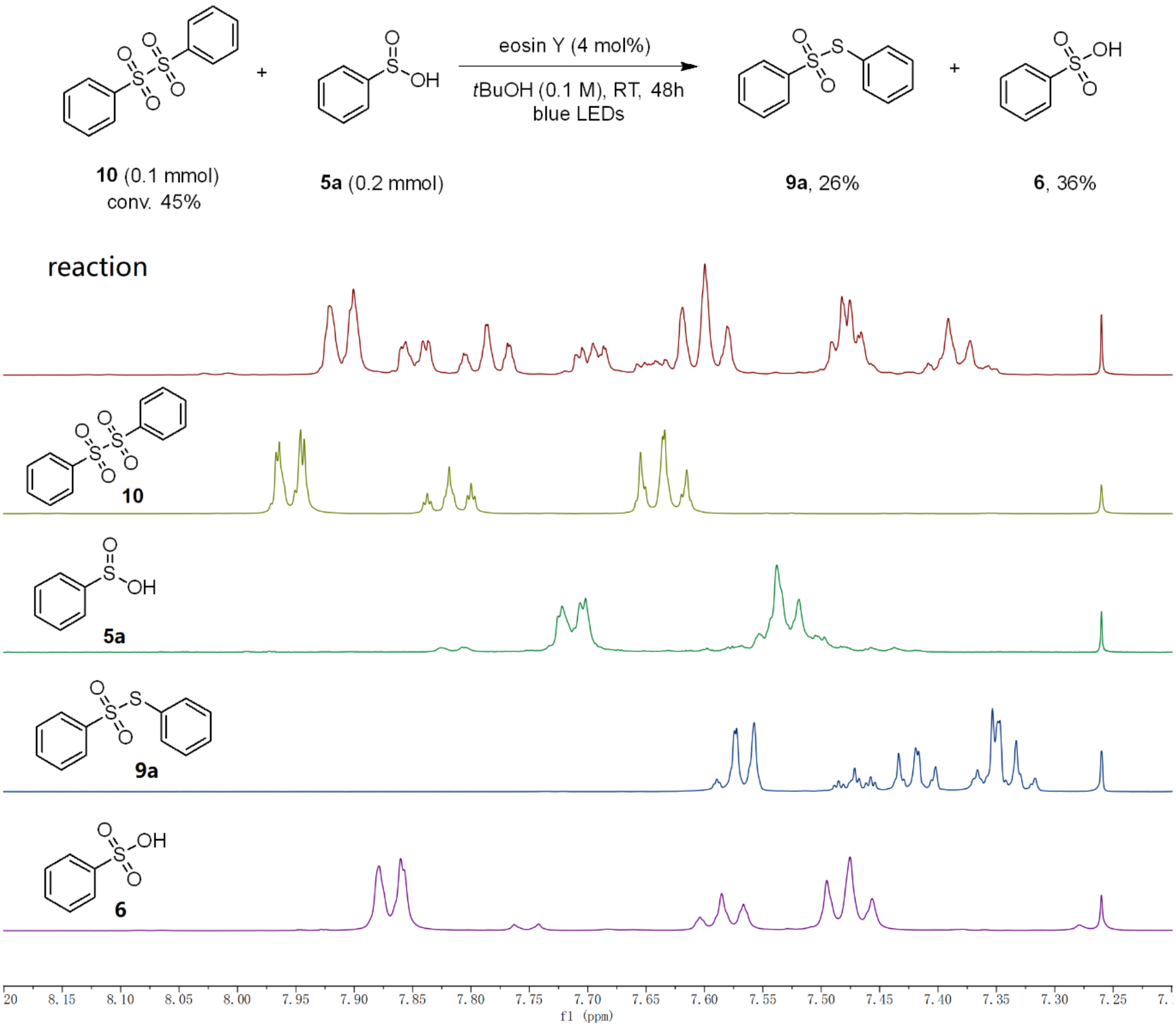

Supplementary Figure 14. Formation of thiosulfonate **9a** from possible intermediate **10**.

Study on electrochemical potentials of sulfone derivatives

To gain evidence for the mechanism proposed in Supplementary Figure 12, we measured electrochemical potentials of **5a**, **6** and **10** using cyclic voltammetry (CV) experiments (Supplementary Figure 15). We also calculated electrochemical redox potentials of eosin Y-H species..

Excited neutral eosin Y[24] ($^*E_{1/2}^{red}$ = +0.83 V vs SCE; $^*E_{1/2}^{ox}$ = -1.11 V vs SCE) is unlikely to perform SET with an aldehyde[25] to generate acyl radical **A**. Thus a photo-induced HAT step is proposed to generate acyl radical **A** from aldehydes.

Although the regeneration of eosin Y by a SET process between $PhSO_2^{\bullet}$ ($E_{1/2}^{red}$ = +0.50 V vs SCE) [26] and eosin Y-H ($E_{1/2}^{ox}$ = -0.11 V vs SCE, calculated) cannot be ruled out, DFT calculation suggested that the RHAT process is a favored pathway (Supplementary Figure 17). HAT process is proposed to generate $PhSO_2^{\bullet}$ from $PhSO_2H$ **5a,** as reported in previous reports.[21] Our CV results also indicate that the SET process between eosin Y ($^*E_{1/2}^{red}$ = +0.83 V vs SCE; $^*E_{1/2}^{ox}$ = -1.11 V vs SCE) and $PhSO_2H$ **5a** ($E_{p/2}^{ox}$ = +1.14 V vs SCE, $E_{p/2}^{red}$ = -1.47

V vs SCE) is unfavored.

The generation of thiosulfonate **9a** from **10** and **5a** is reported.[23] Our CV measurement ($E_{1/2}^{red}$ (**10**) = -1.02 V vs SCE, $E_{1/2}^{red}$ (**5a**) = -1.47 V) also supports this step.
Cyclic voltammograms (CV) were collected using a VersaSTAT 3 Potentiostat Galvanostat from Princeton Applied Research. Samples were prepared with 0.03 mmol of substrate in 3 mL of 0.1 M tetra-*n*-butylammonium hexafluorophosphate in dry acetonitrile. The samples were bubbled with argon for 10 min to avoid trace amount of O_2. Measurements were conducted using glassy carbon working electrode, platinum wire counter electrode, and 3.5 M NaCl silver-silver chloride reference electrode in a scan rate of 0.1 V/s. Ferrocene (Fc) was used as internal standard. Reductions were measured by scanning potentials in the negative direction and oxidations in the positive direction.

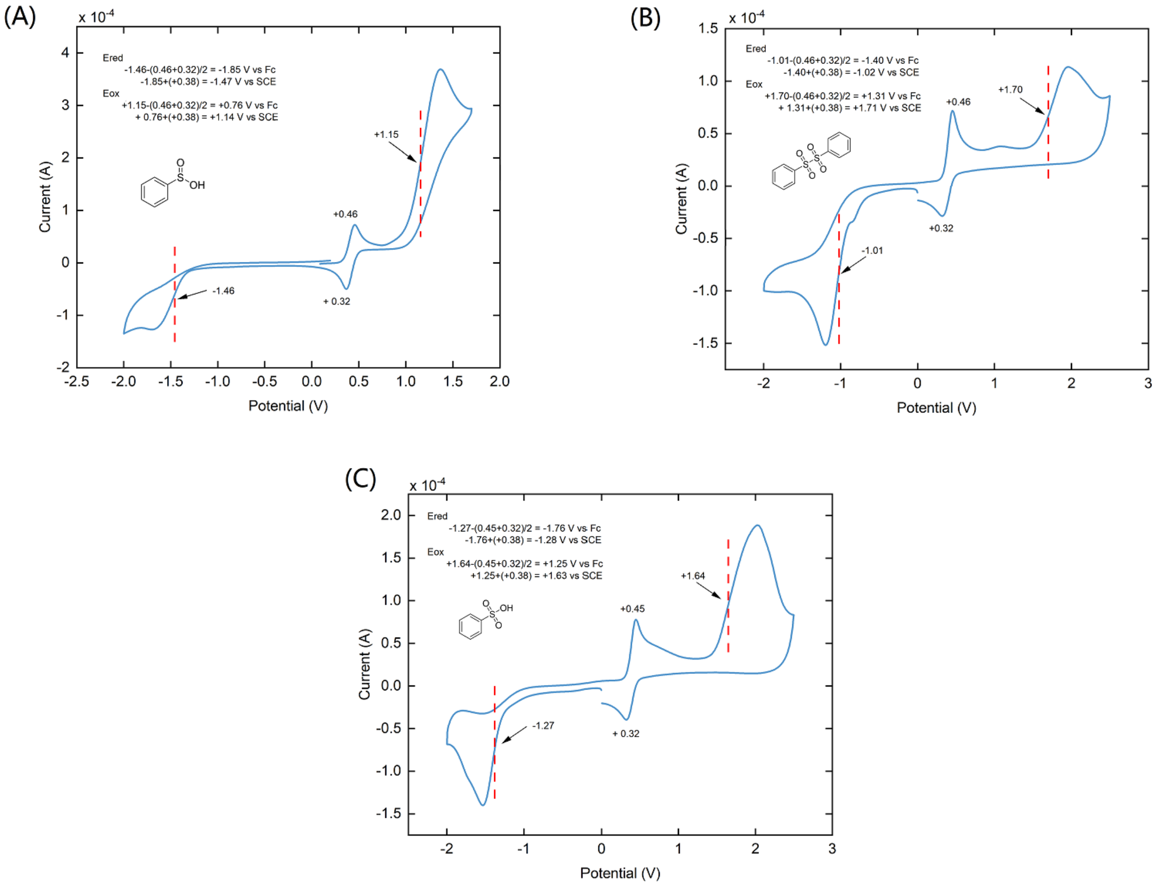

Supplementary Figure 15. Cyclic voltammograms. (A) benzenesulfinic acid **5a**. (B) diphenyl disulfone **10**. (C) benzenesulfonic acid **6**.

Calculation of electrochemical potential of eosin Y-H species

Density functional theory (DFT) calculations were performed to study the electrochemical potential of eosin Y-H. The calculation method was adapted from Reference.[27] The redox potential $E_{1/2}^{o,calc}$ (vs SCE) was calculated according to the following equation:

$$E_{1/2}^{o,calc}(\text{vs SCE}) = -\frac{(G_{298}[reduced] - G_{298}[oxidized])}{n_e\mathcal{F}} - E_{1/2}^{o,SHE} - E_{1/2}^{o,SCE}$$

$E_{1/2}^{ox}$ (eosin Y-H) = -0.11 V vs SCE

Where n_e is the number of electrons transferred (n_e=1 in our calculation), $\mathcal{F}$ is the Faraday constant (23.061 kcal mol^{-1} V^{-1}). $E_{1/2}^{o,SHE}$ is the absolute value for the standard hydrogen electrode (SHE, value = 4.281 V), $E_{1/2}^{o,SCE}$ is the potential of the saturated calomel electrode (SCE) relative to SHE in acetonitrile (value = 0.141 V). $G_{298}[reduced]$ and $G_{298}[oxidized]$ (1 Hartrees = 630 kcal/mol) are the calculated Gibbs free energies with the solvation model.

Computational Details

The geometries optimization in this study was performed at the uB3LYP/Def2SVP level of theory. The free energies of the optimized geometries were calculated at the same level of theory, considering the solvent effect using the Solvation Model Density (SMD) solvation model. All calculations were performed using the Gaussian 16 Rev. A.03 software suite. [28] See Supplementary Data for calculation details.

Determination of the quantum yield by standard ferrioxalate actinometry

Determination of the light intensity at 470 nm: Following Yoon's procedure,[29] the photon flux of the spectrophotometer was determined by standard ferrioxalate actinometry. A 0.15 M solution of ferrioxalate was prepared by dissolving 2.21 g of potassium ferrioxalate hydrate in 30 mL of 0.05 M H_2SO_4. A buffered solution of phenanthroline was prepared by dissolving 50 mg of phenanthroline and 11.25 g of sodium acetate in 50 mL of 0.5 M H_2SO_4. Both solutions were stored in the dark. To determine the photon flux of the spectrophotometer, 2.0 mL of the ferrioxalate solution was placed in a cuvette and irradiated for 90.0 seconds at λ = 470 nm with an emission slit width at 10.0 nm. After irradiation, 0.35 mL of the phenanthroline solution was added to the cuvette. The solution was then allowed to rest for 1 h to allow the ferrous ions to completely coordinate to the phenanthroline. The absorbance of the solution was measured at 510 nm. A non-irradiated sample was also prepared and the absorbance at 510 nm measured. Conversion was calculated using eq S5.

eq S5

$$\text{mol } Fe^{2+} = \frac{V \bullet \Delta A}{l \bullet \varepsilon}$$

V: Total volume (0.00235 L)
ΔA: difference in absorbance at 510 nm betwween irradiated and non-irradiated solution (0.874)
l: path length (1.000 cm)
ε: molar absorptivity at 510 nm (11100 L/mol/cm)

$$\text{mol } Fe^{2+} = \frac{0.00235 \text{ L} \bullet 0.874}{1 \text{ cm} \bullet 11100 \text{ L/mol/cm}} = 1.85 \times 10^{-7} \text{ mol}$$

Where V is the total volume (0.00235 L) of the solution after addition of phenanthroline, ΔA is the difference in absorbance at 510 nm between the irradiated and non-irradiated solutions, l is the path length (1.000 cm), and ε is the molar absorptivity at 510 nm (11,100 L mol^{-1} cm^{-1}).

The photon flux can be calculated using eq S6.

eq S6

$$\text{Photon flux} = \frac{\text{mol } Fe^{2+}}{\Phi \bullet t \bullet f}$$

Φ: Quantum yield for the ferrioxalate actinometer (0.92 for 0.15 M solution at λ = 468 nm)
t: irradiation time at 470 nm (90.0 s)
f: fraction of light at λ = 470 nm (0.735, $f = 1\text{-}10^{-A}$)

$$\text{Photon flux} = \frac{1.85 \times 10^{-7} \text{ mol}}{0.92 \bullet 90.0 \bullet 0.735} = 3.04 \times 10^{-9} \text{ einstein s}^{-1}$$

Where Φ is the quantum yield for the ferrioxalate actinometer (0.92 for a 0.15 M solution at λ = 468 nm),[30] t is the time (90.0 s), and f is the fraction of light absorbed at λ = 470 nm (0.725). The photon flux was calculated (average of three experiments) to be 3.04×10^{-9} einstein s^{-1}.

Determination of quantum yield of fluoromethylthiolation:

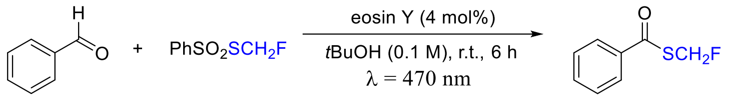

1a (0.3 mmol) **2a** (0.2 mmol) **3a**, 14 %

A cuvette was charged with *S*-(fluoromethyl) benzenesulfonothioate ($PhSO_2$–SCH_2F) **2a** (41.3 mg, 0.2 mmol), benzaldehyde **1a** (31 uL, 0.3 mmol), eosin Y (5.2 mg, 0.008 mmol), in anhydrous *tert*-butanol (2 mL). The cuvette was then capped with a PTFE stopper. The sample was stirred and irradiated (λ = 470 nm, slit width = 10.0 nm) for 21600 s (6 h). After irradiation, the solvent was removed. The yield of product formed was determined as 14% by crude ^{1}H NMR based on a dibromomethane standard. The quantum yield was determined using eq S7. Essentially all incident light (f > 0.999) is absorbed by the eosin Y at the reaction conditions described above.

eq S7

$$\Phi = \frac{\text{mol product}}{\text{flux} \bullet t \bullet f} = \frac{1.4 \times 10^{-5} \text{ mol}}{3.04 \times 10^{-9} \text{ einstein s}^{-1} \bullet 21600 \text{ s} \bullet 1.00} = 0.21$$

Φ (14%) = 0.21, radical chain mechanism is not suggested.

Light on/off experiments over time

To examine the impact of light, we conducted experiments under alternating periods of irradiation and darkness (Supplementary Figure 16). These resulted in a total interruption of the reaction progress in the absence of light and recuperation of reactivity on further illumination, which allows precise temporal control over the entire reaction period. These results demonstrate that light is a necessary component of the reaction. Even though they do not definitively rule out a radical-chain process, the data show that any chain-propagation process must be short-lived.

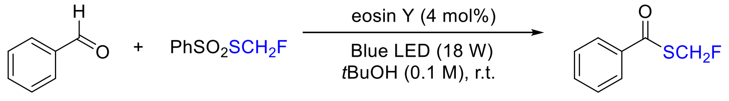

1a (0.3 mmol) **2a** (0.2 mmol) **3a**

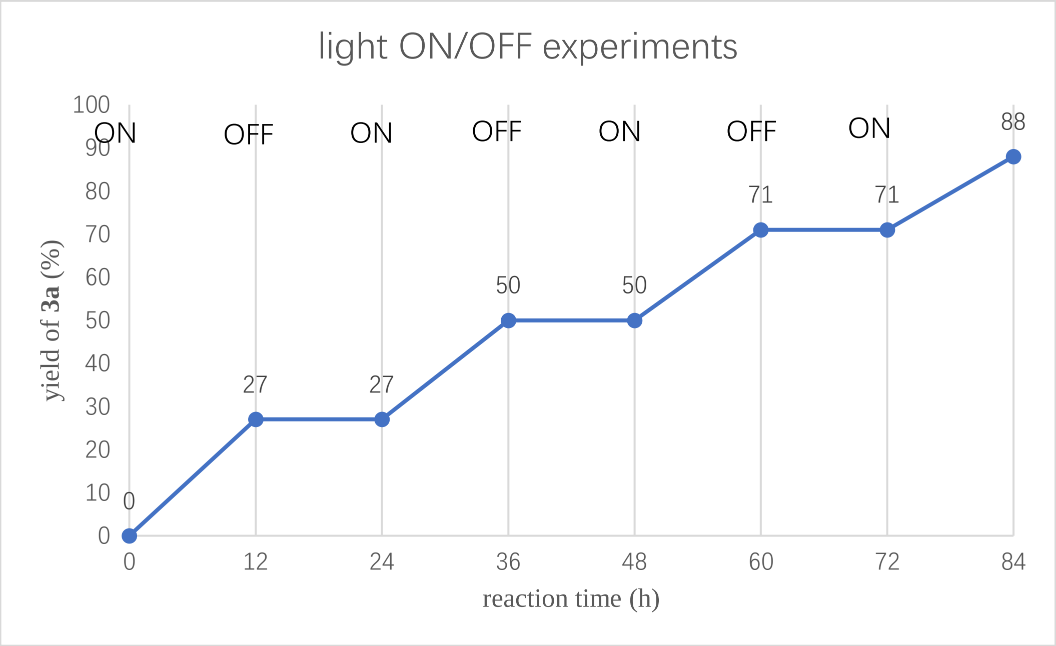

Supplementary Figure 16. Time profile of the transformation with the light ON/OFF over time.

DFT calculation on SET and RHAT pathways for eosin Y regeneration

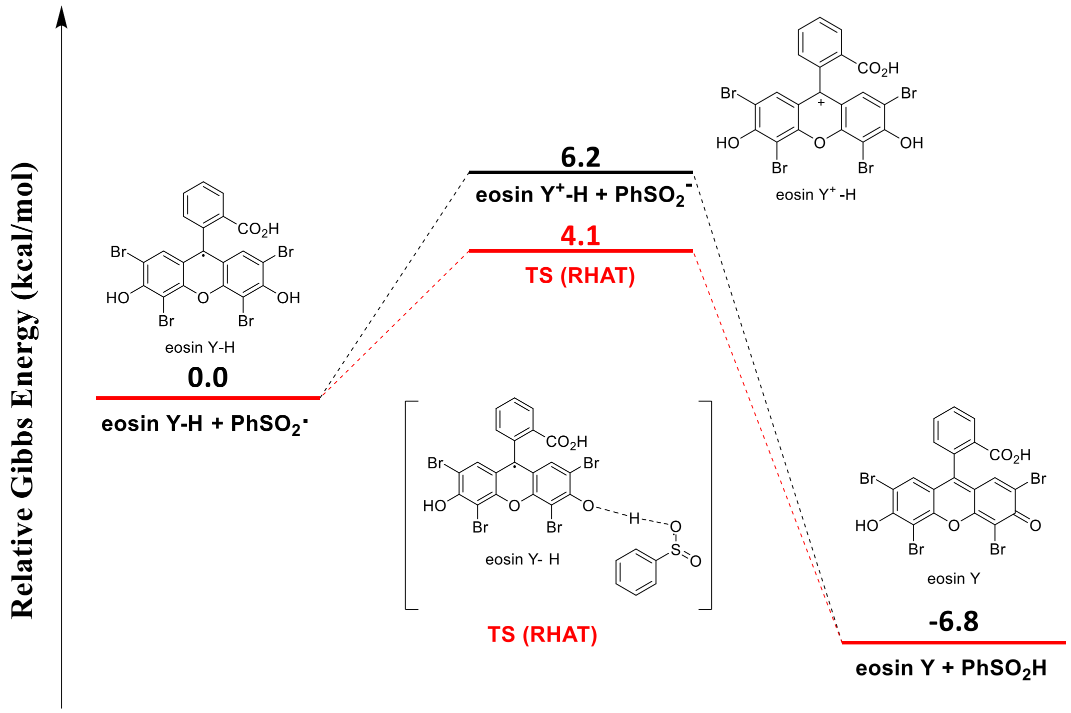

Supplementary Figure 17. Density functional theory (DFT) calculations on eosin Y regeneration.

Computational details

Density functional theory (DFT) calculations were performed to shed light on the mechanism

of eosin Y regeneration (Supplementary Figure 17). Reverse hydrogen atom transfer (RHAT, red line) is the favored pathway, which features a barrier 2.1 kcal/mol lower than an alternative single electron transfer (SET, black line). The geometries optimization in this study was performed at the uB3LYP density functional with a standard def2-SVP basis set. The nature of the stationary points (minima with no imaginary frequency or transition states with one imaginary frequency) were confirmed. The free energies of the optimized geometries were calculated at the same level of theory, considering the solvent effect of acetone using an SMD continuum solvation model. Unless specified otherwise, the Gibbs free energy was used throughout. For transition state, intrinsic reaction coordinate (IRC) calculations were performed to verify whether it connected with correct reactants and products or intermediates. All calculations were performed using the Gaussian 16 Rev. A.03 software suite.[28] See Supplementary Data for calculation details.

VII. Scale up in flow reactors

Images of flow reactors are shown in Supplementary Figure 18. Back pressure regulators (BPRs), high-purity perfluoroalkoxy polymer (HPFA) tubing, fittings were purchased from IDEX Health & Science Technologies. The HPLC pump (Model No. LS226100) was purchased from Chemikalie Pte Ltd. Selection valves were purchased from Valco Instruments Co. Inc.

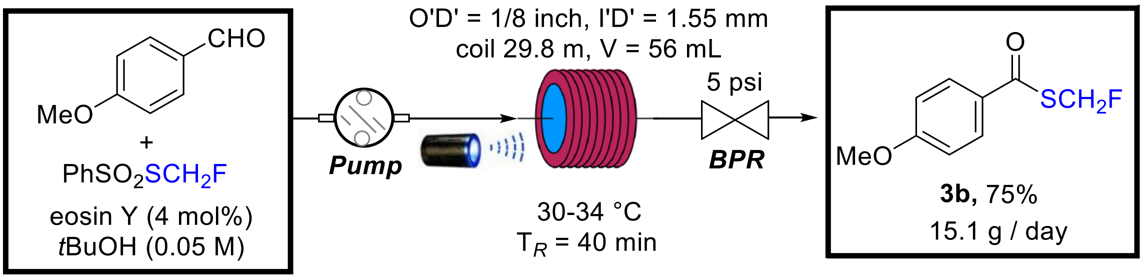

Flow reactor setup

A 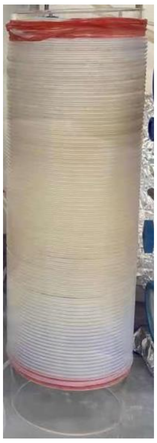 B 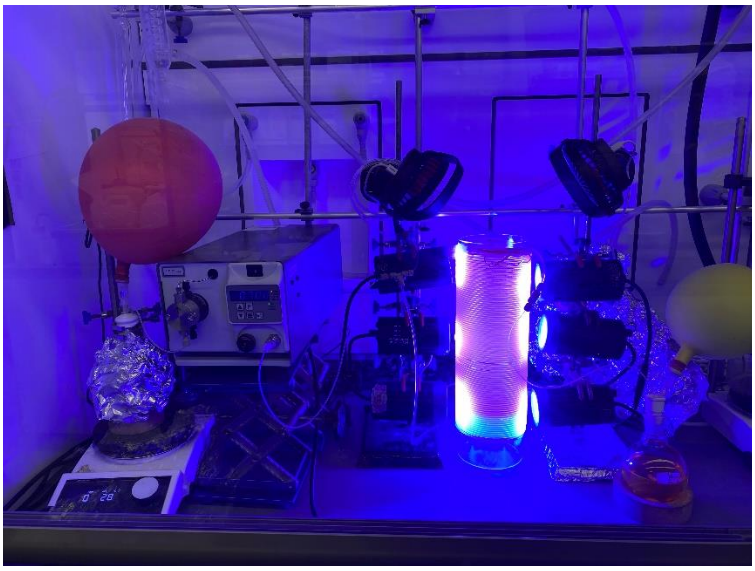

Supplementary Figure 18. The flow apparatus. **A**. The flow tubing on a glass cylinder. **B**. The flow set-up with light irradiation.

Aldehydic C–H fluoromethylthiolation in flow

Inside a glove-box, 4-methoxybenzaldehyde **1b** (2.78 mL, 22.4 mmol) and *S*-(fluoromethyl) benzenesulfonothioate **2a** (2.31 g, 11.2 mmol) were added to a 250 mL round bottom flask. The reagents were dissolved in degassed *tert*-butanol (*t*BuOH) and the total volume of the solution was adjusted to 224 mL. The reaction solution was introduced to the flow apparatus (Supplementary Figure 18). The flow apparatus was purged with degassed *t*BuOH to remove the air first. An HPLC pump (Model No. LS226100) was then connected to the reaction mixture and the tubing with 5 psi back-pressure regulator (BPR). The HPFA (high purity perfluoroalkoxyalkane) tubing (O.D. = 1/8 inch, I.D. = 1.55 mm, length = 29.8 m, volume = 56 mL) was rounded on a glass cylinder (I.D. = 10 cm). The tubing was then irradiated by six sets of 440 nm Kessil lamps (the light intensity for each was set 40 W). The flow apparatus was cooled by two fans, keeping the ambient temperature around 30-34 °C. The flow apparatus itself was set up with residence time (T_R) = 40 min, flow rate = 1.4 mL/min. After 60 min of equilibration, the product mixture was collected for 80 min. A crude sample (4 mL) was taken from the collected solution and analyzed by ^{1}H-NMR spectroscopy using CH_2Br_2 (7 µL, 0.1 mmol) as an internal standard. Full conversion of *S*-(fluoromethyl) benzenesulfonothioate (**2a**) was observed and the ^{1}H-NMR yield of product **3b** was 82%. The crude NMR sample was recovered and combined with the reaction mixture. The combined crude was concentrated and purified by column chromatography to give product **3b** (841 mg) in 75% yield (the productivity was 15.1 g/day).

VIII. NMR spectra

Supplementary Figure 19a | ^{1}H NMR (400 MHz, $CDCl_3$) of (3*S*,8*S*,9*S*,10*S*,13*R*,14*S*,16*S*)-10,13-Dimethyl-16-((*S*)-6-methylheptan-2yl)hexadecahydro-1*H* cyclopenta[a]phenanthren-3-yl 4-formylbenzoate (**14o**)

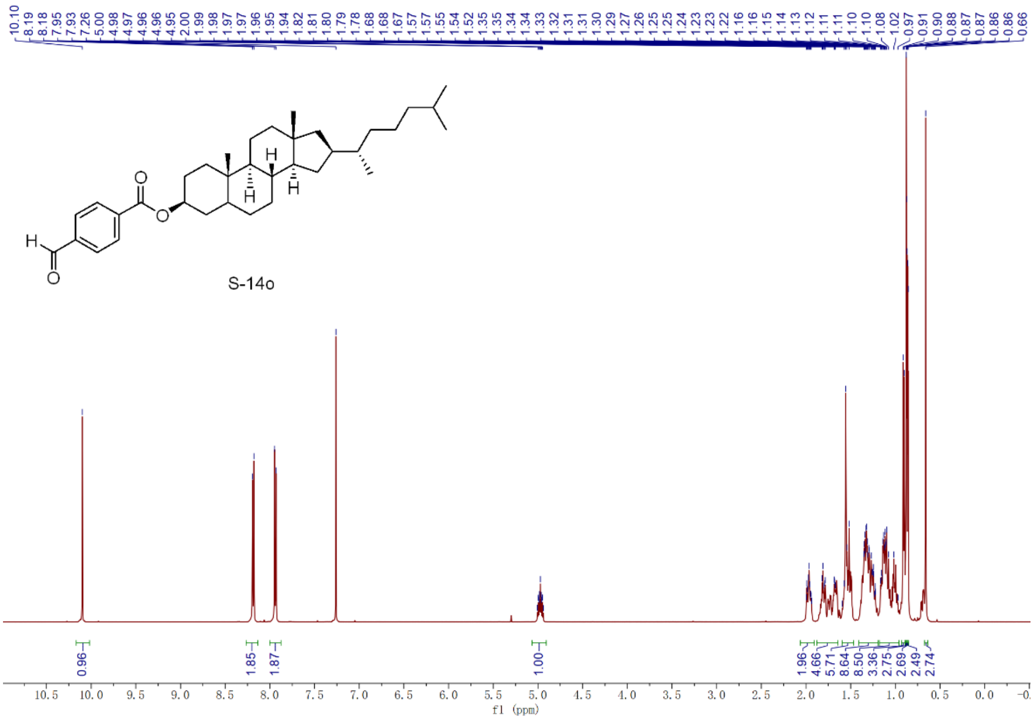

Supplementary Figure 19b | ^{13}C NMR (126 MHz, $CDCl_3$) of (3*S*,8*S*,9*S*,10*S*,13*R*,14*S*,16*S*)-10,13-Dimethyl-16-((*S*)-6-methylheptan-2yl)hexadecahydro-1*H* cyclopenta[a]phenanthren-3-yl 4-formylbenzoate (**14o**)

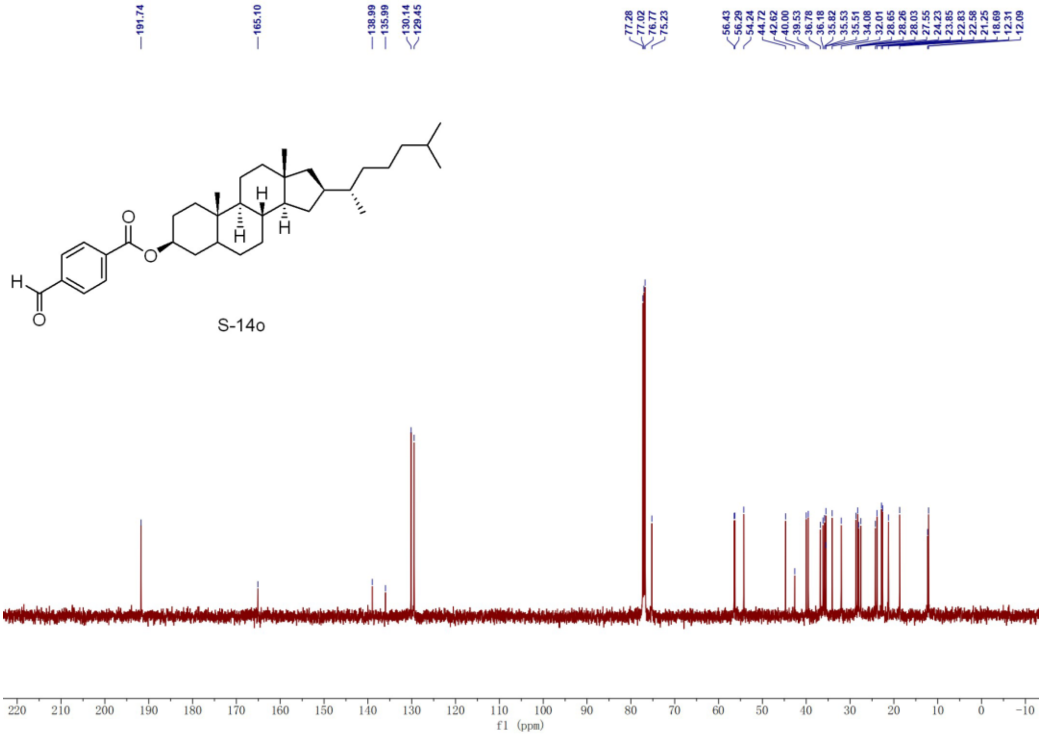

Supplementary Figure 20a | ^{1}H NMR (500 MHz, $CDCl_3$) of 4-(Trifluoromethyl)benzenesulfinic acid (**5s**)

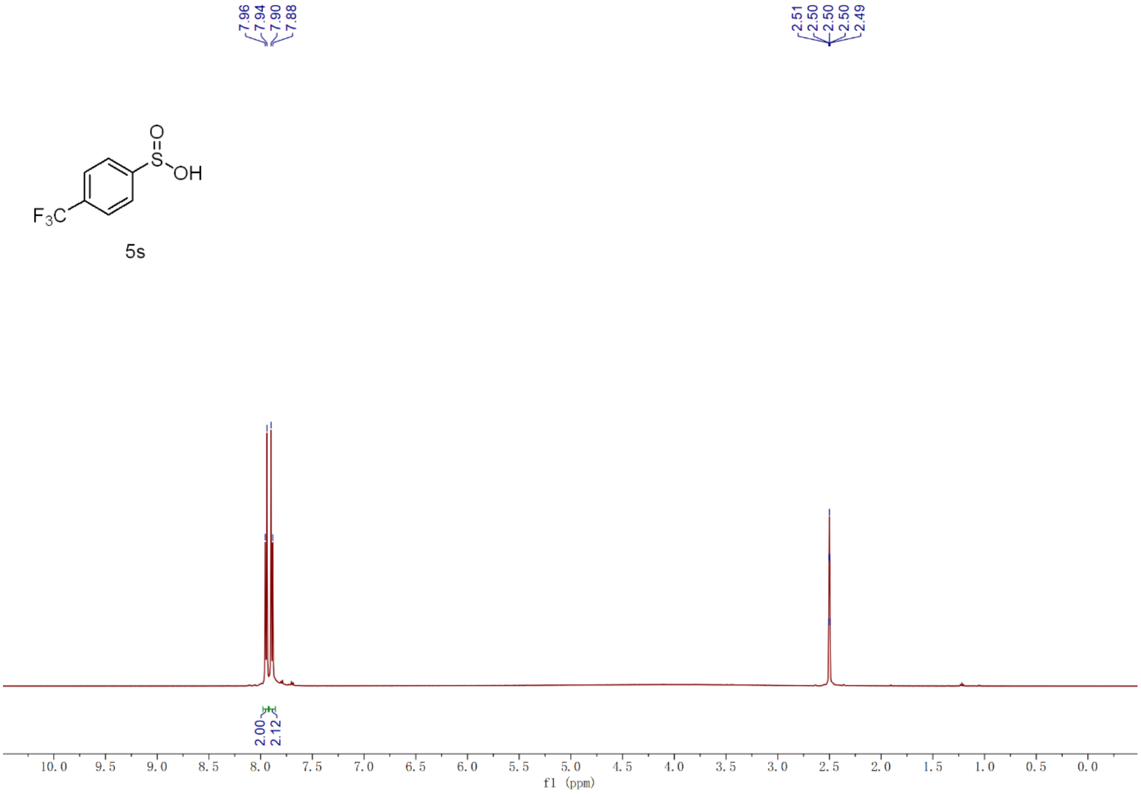

Supplementary Figure 20b | ^{13}C NMR (126 MHz, $CDCl_3$) of 4-(Trifluoromethyl)benzenesulfinic acid (**5s**)

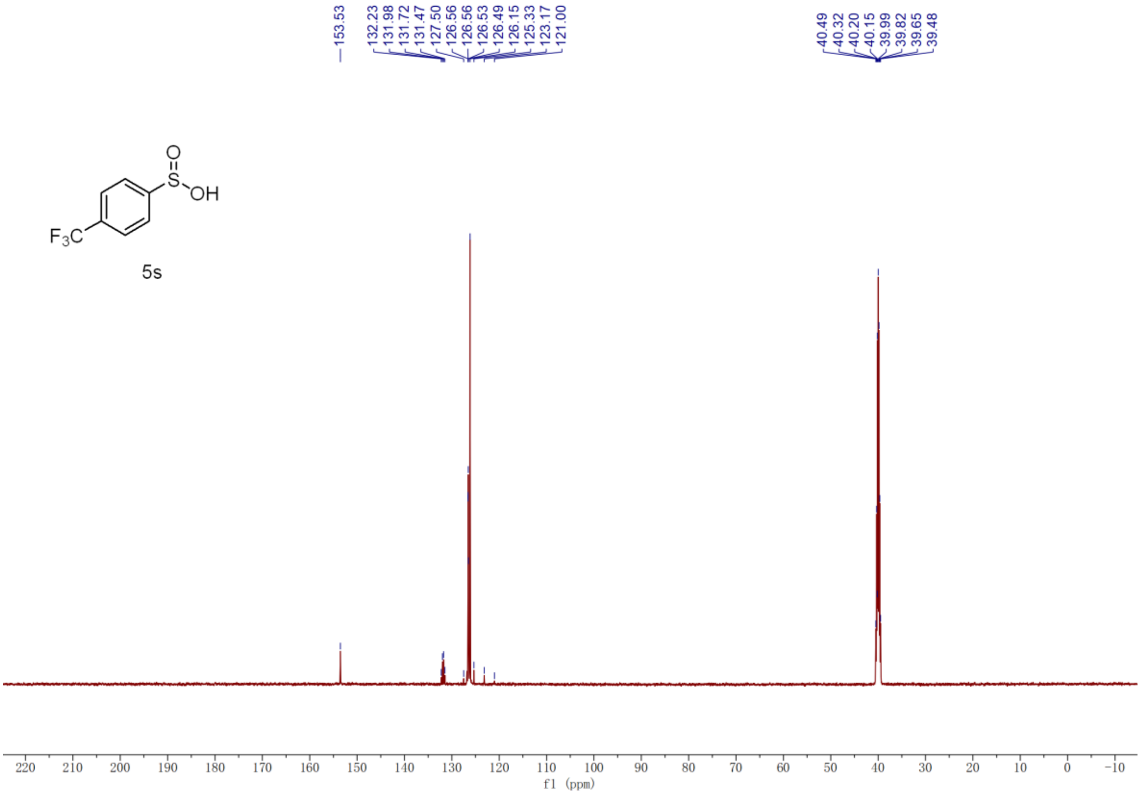

Supplementary Figure 20c | ^{19}F NMR (377 MHz, $CDCl_3$) of 4-(Trifluoromethyl)benzenesulfinic acid (**5s**)

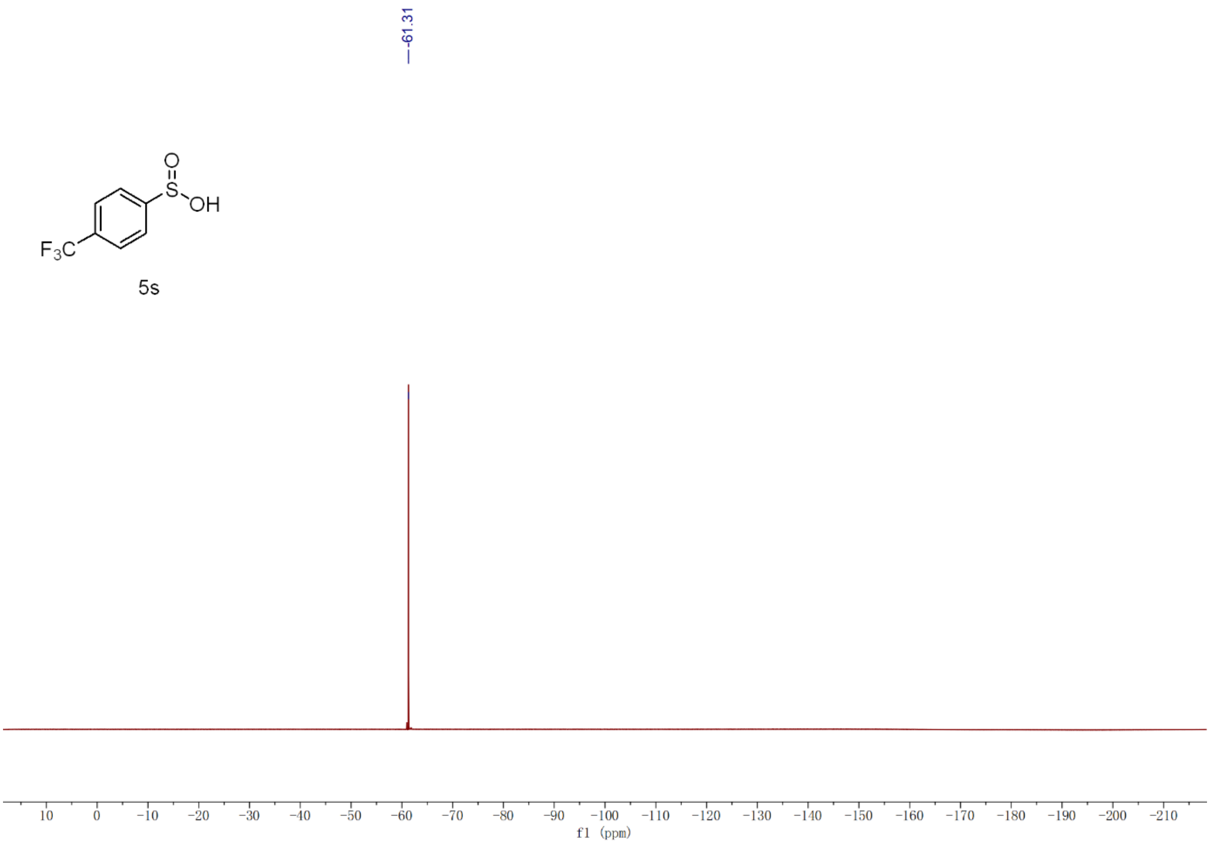

Supplementary Figure 21a | ^{1}H NMR (500 MHz, $CDCl_3$) of ((Methylsulfonyl)ethynyl)benzene (**13aa**)

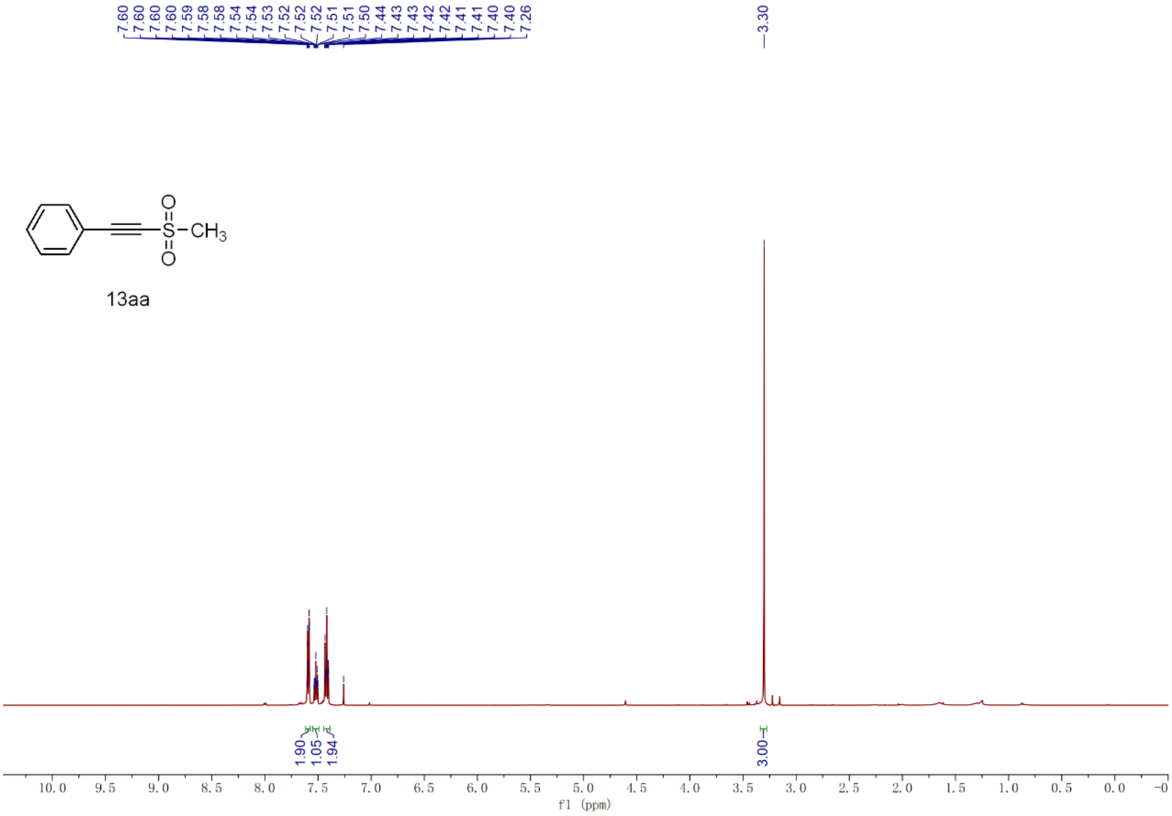

Supplementary Figure 21b | ^{13}C NMR (126 MHz, $CDCl_3$) of ((Methylsulfonyl)ethynyl)benzene (**13aa**)

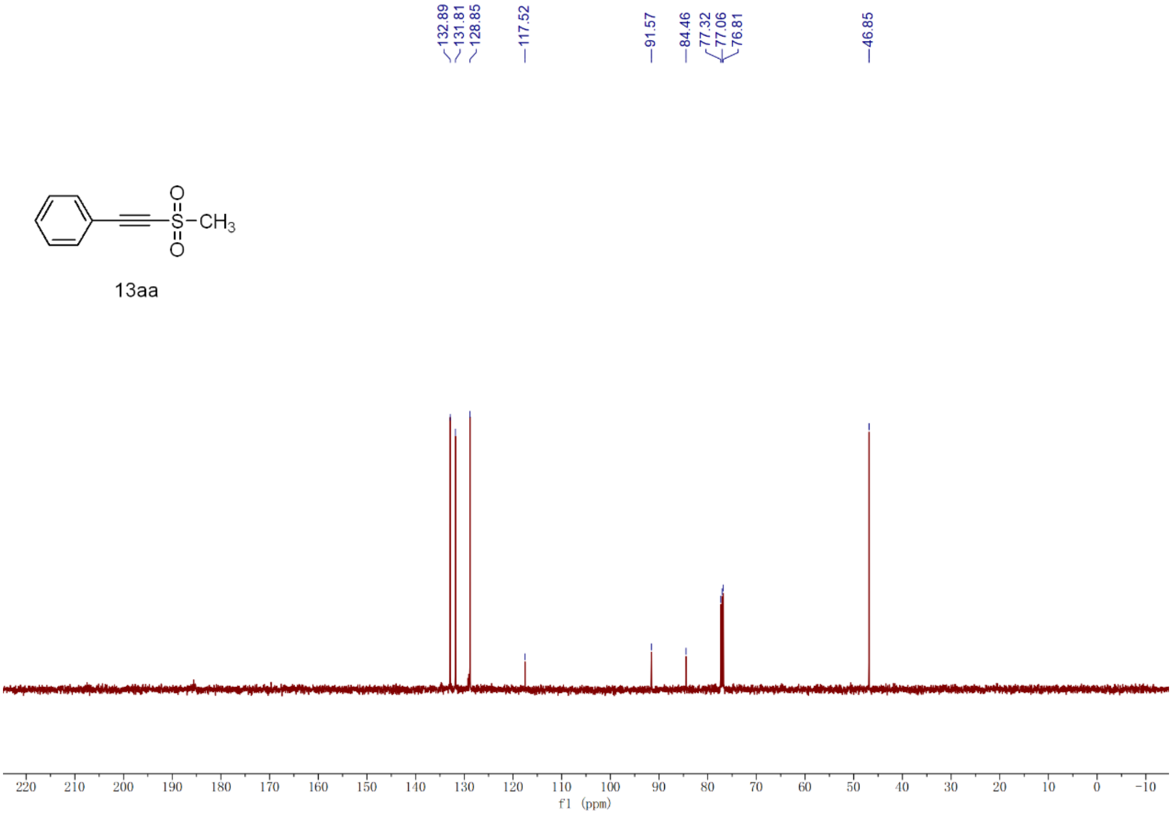

Supplementary Figure 22a | ^{1}H NMR (500 MHz, $CDCl_3$) of 1-Methyl-4-((phenylethynyl)sulfonyl)benzene (**13ab**)

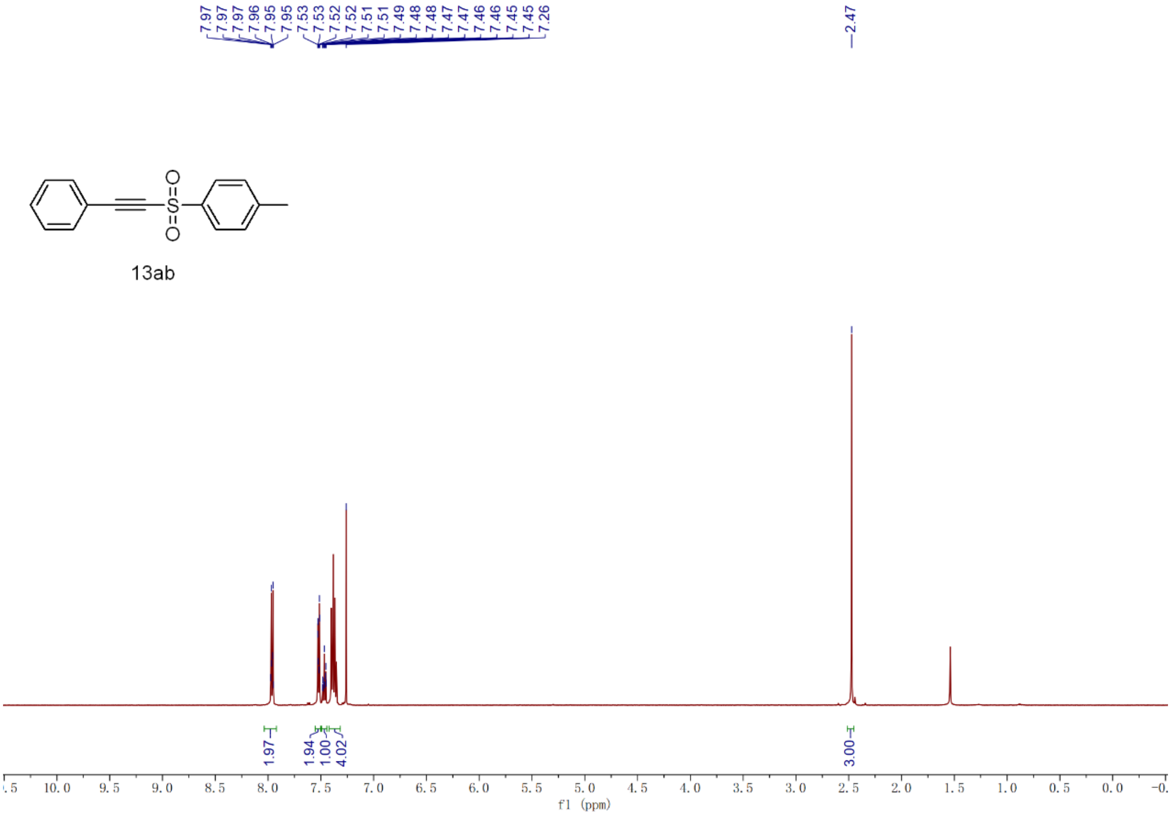

Supplementary Figure 22b | ^{13}C NMR (126 MHz, $CDCl_3$) of 1-Methyl-4-((phenylethynyl)sulfonyl)benzene (**13ab**)

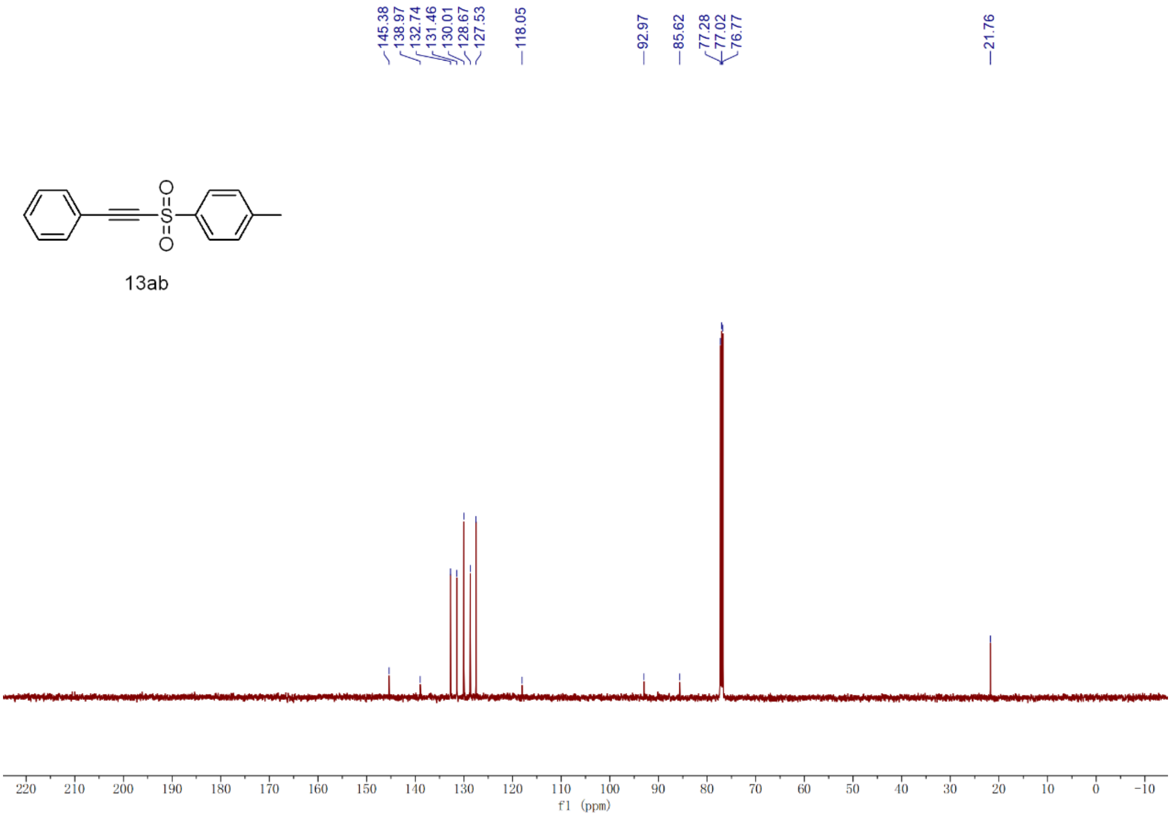

Supplementary Figure 23a | ^{1}H NMR (500 MHz, $CDCl_3$) of 1-Methoxy-4-((phenylethynyl)sulfonyl)benzene (**13ac**)

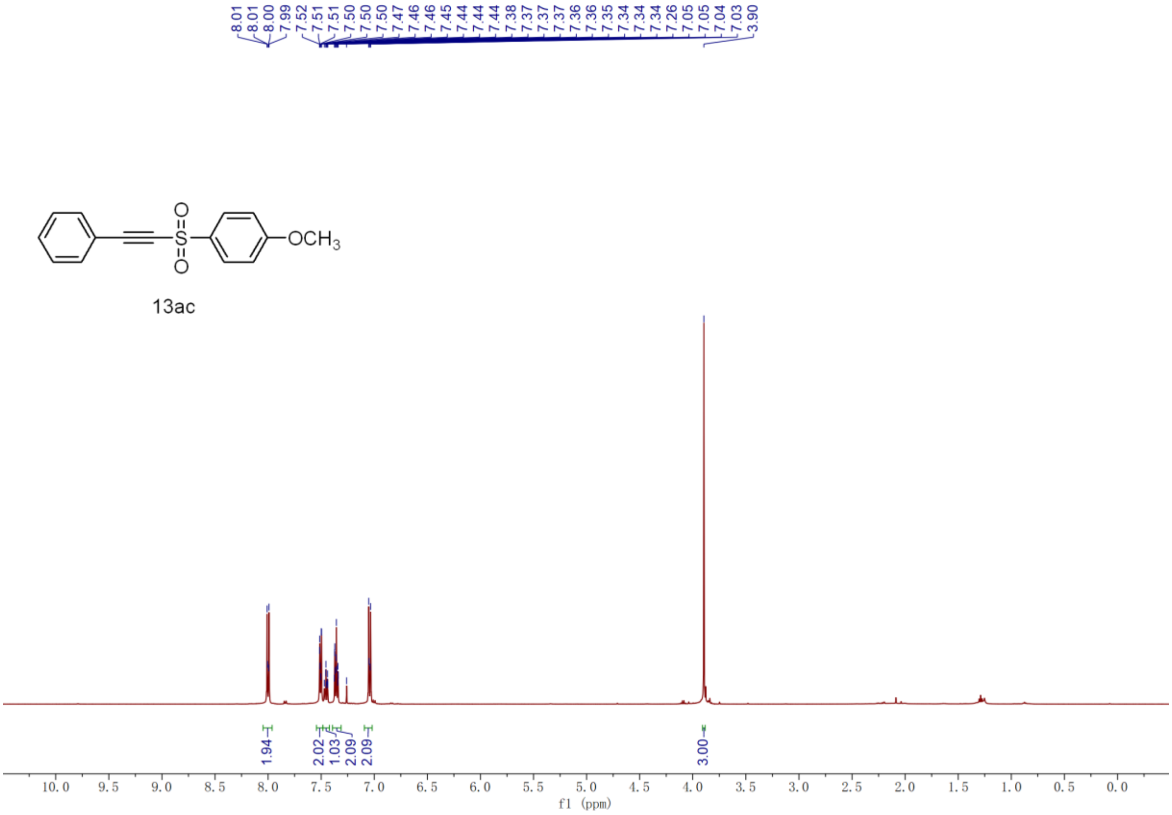

Supplementary Figure 23b | ^{13}C NMR (126 MHz, $CDCl_3$) of 1-Methoxy-4-((phenylethynyl)sulfonyl)benzene (**13ac**)

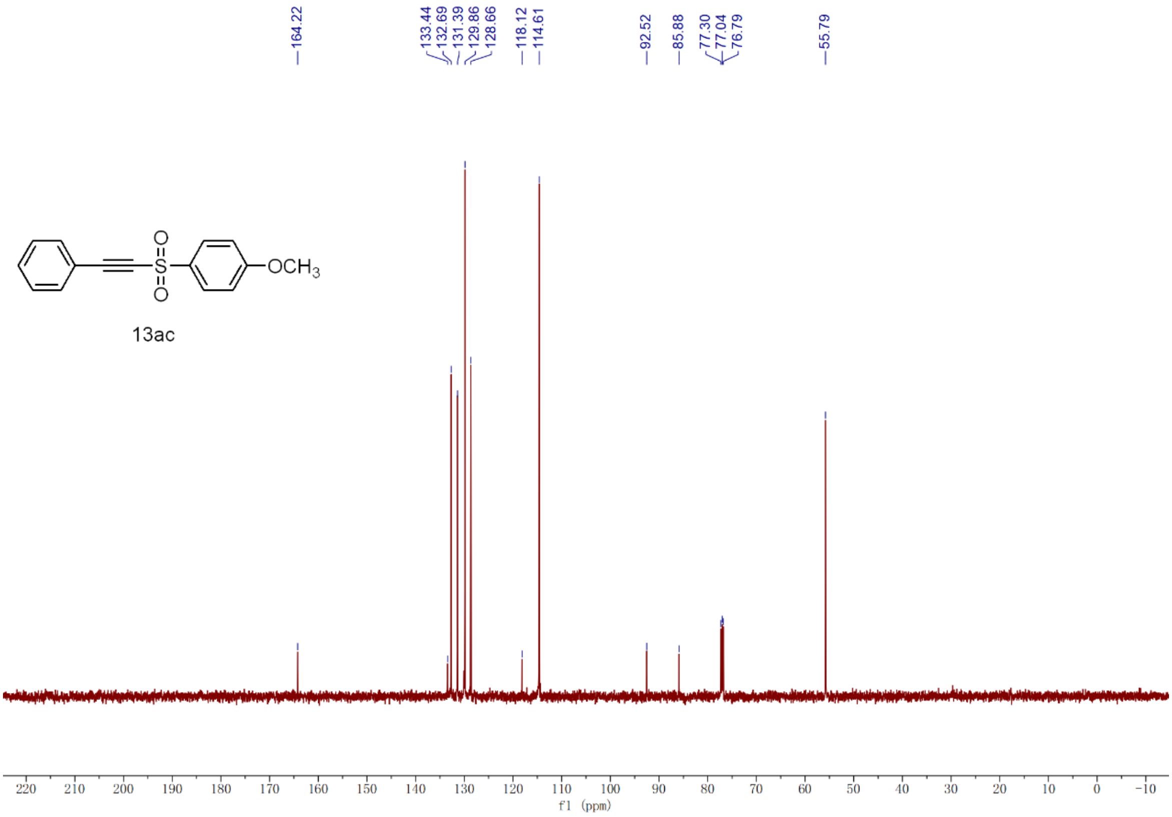

Supplementary Figure 24a | ^{1}H NMR (500 MHz, $CDCl_3$) of 1-((Phenylethynyl)sulfonyl)-4-(trifluoromethyl)benzene (**13ae**)

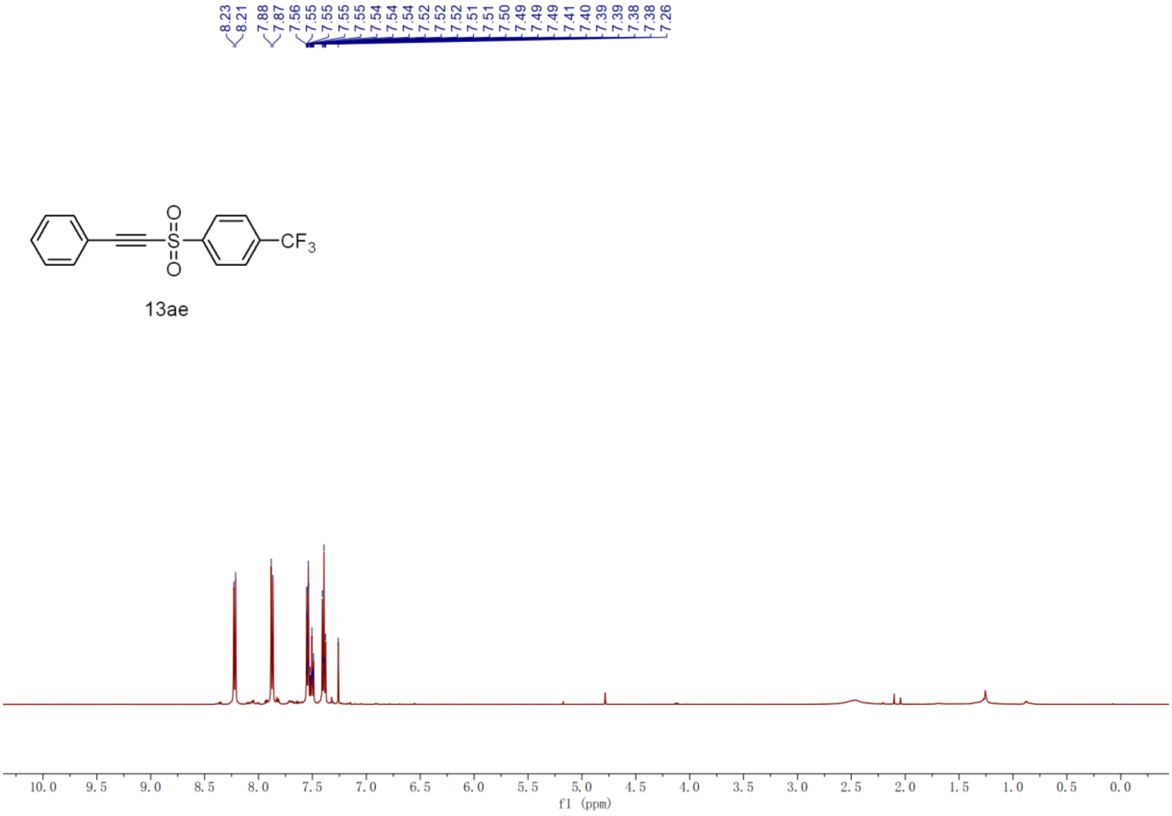

Supplementary Figure 24b | ^{13}C NMR (126 MHz, $CDCl_3$) of 1-((Phenylethynyl)sulfonyl)-4-(trifluoromethyl)benzene (**13ae**)

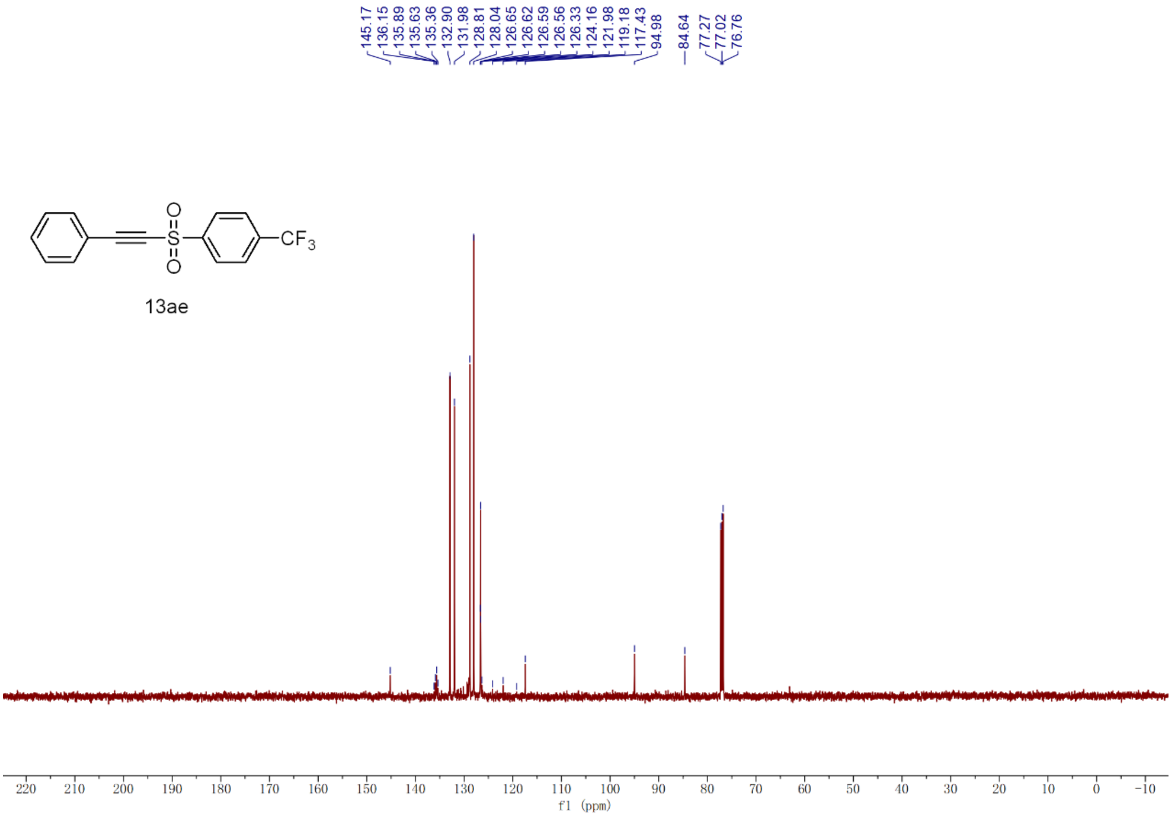

Supplementary Figure 24c | ^{19}F NMR (377 MHz, $CDCl_3$) of 1-((Phenylethynyl)sulfonyl)-4-(trifluoromethyl)benzene (**13ae**)

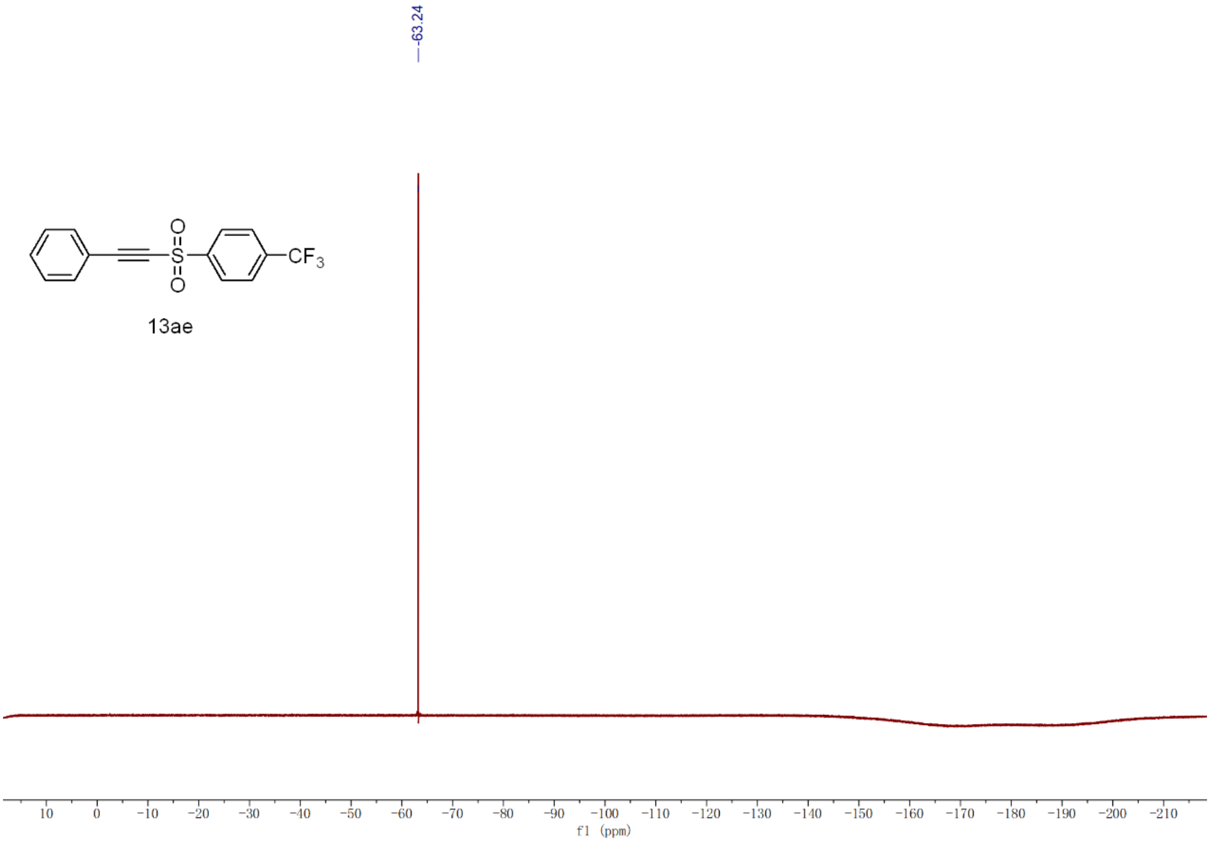

Supplementary Figure 25a | ^{1}H NMR (400 MHz, $CDCl_3$) of ((Phenylethynyl)sulfonyl)benzene (**13af**)

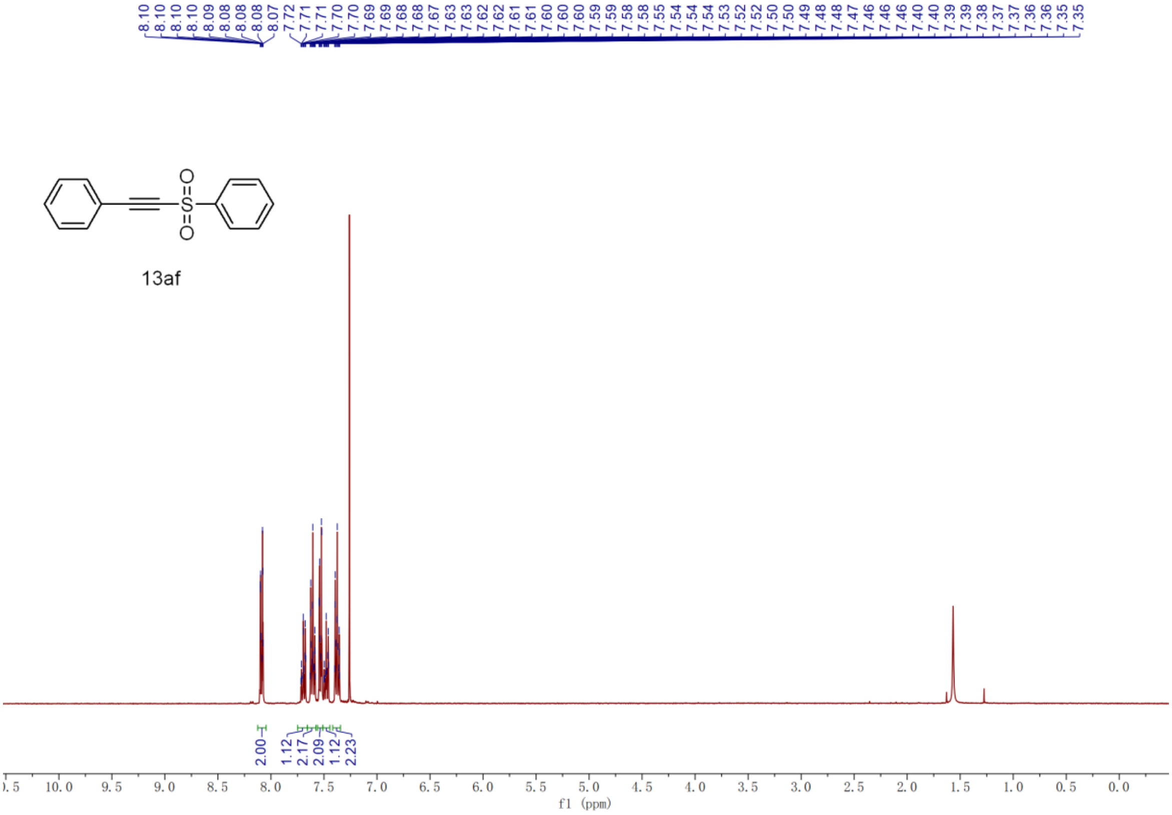

Supplementary Figure 25b | ^{13}C NMR (126 MHz, $CDCl_3$) of ((Phenylethynyl)sulfonyl)benzene (**13af**)

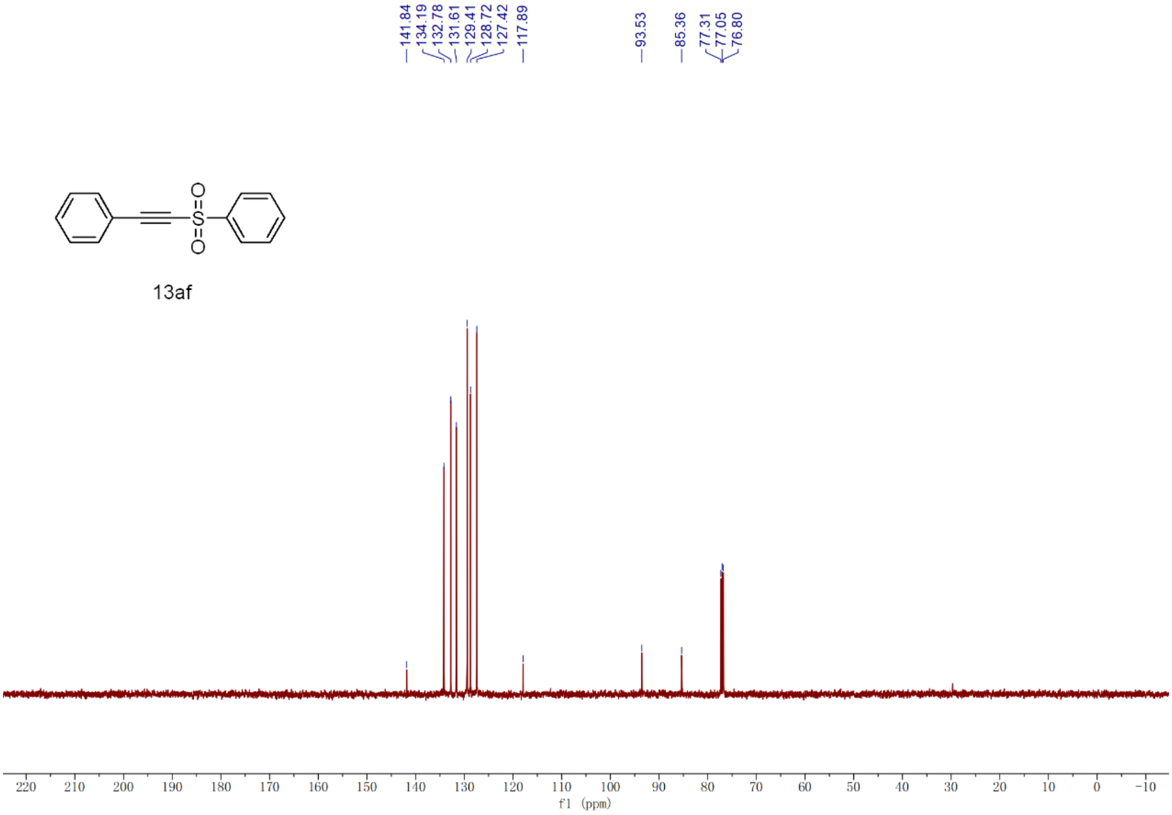

Supplementary Figure 26a | ^{1}H NMR (500 MHz, $CDCl_3$) of 1-Methyl-4-((methylsulfonyl)ethynyl)benzene (**13p**)

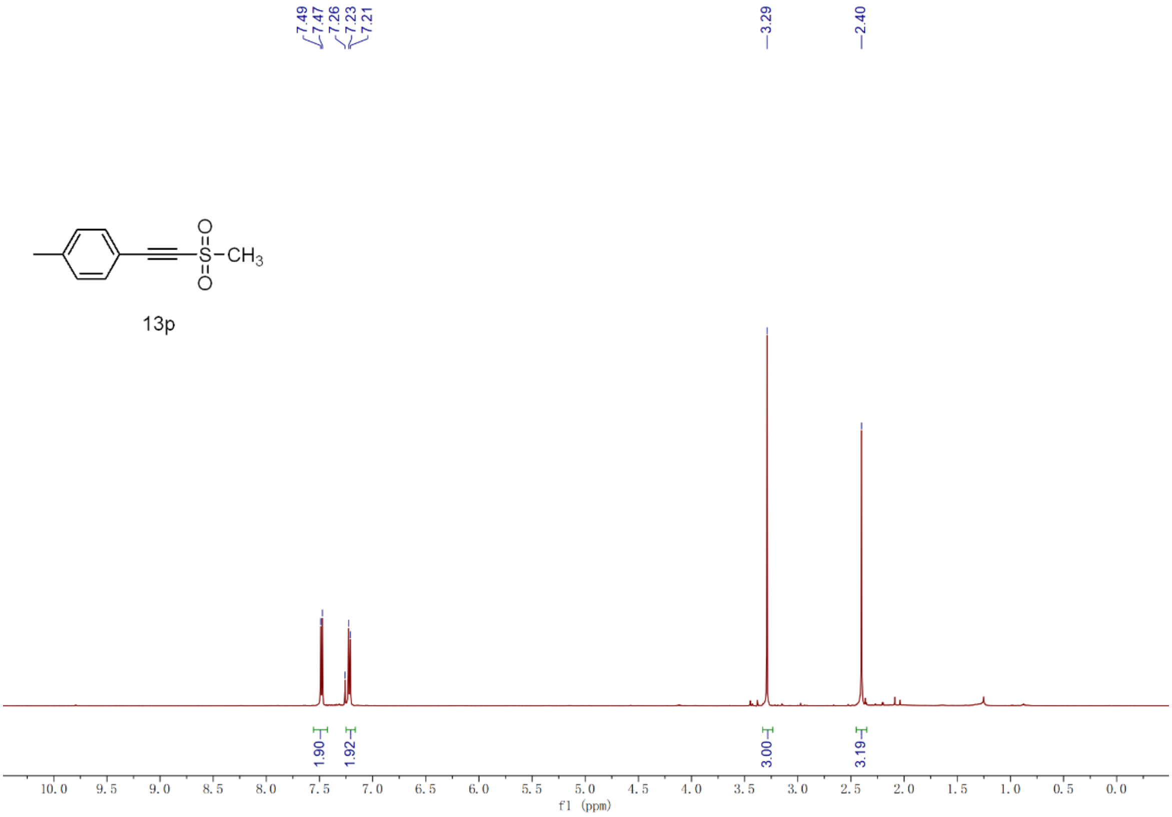

Supplementary Figure 26b | ^{13}C NMR (126 MHz, $CDCl_3$) of 1-Methyl-4-((methylsulfonyl)ethynyl)benzene (**13p**)

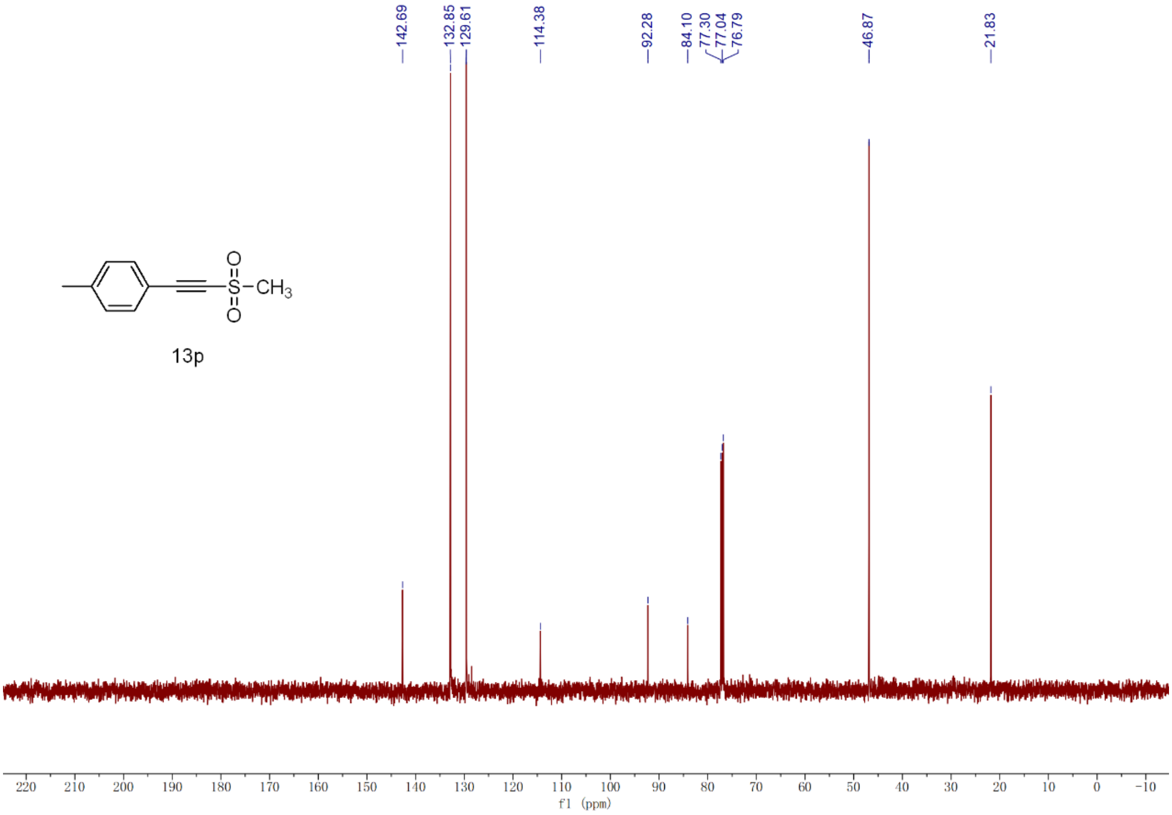

Supplementary Figure 27a | ^{1}H NMR (500 MHz, $CDCl_3$) of 4-((Methylsulfonyl)ethynyl)-1,1'-biphenyl (**13q**)

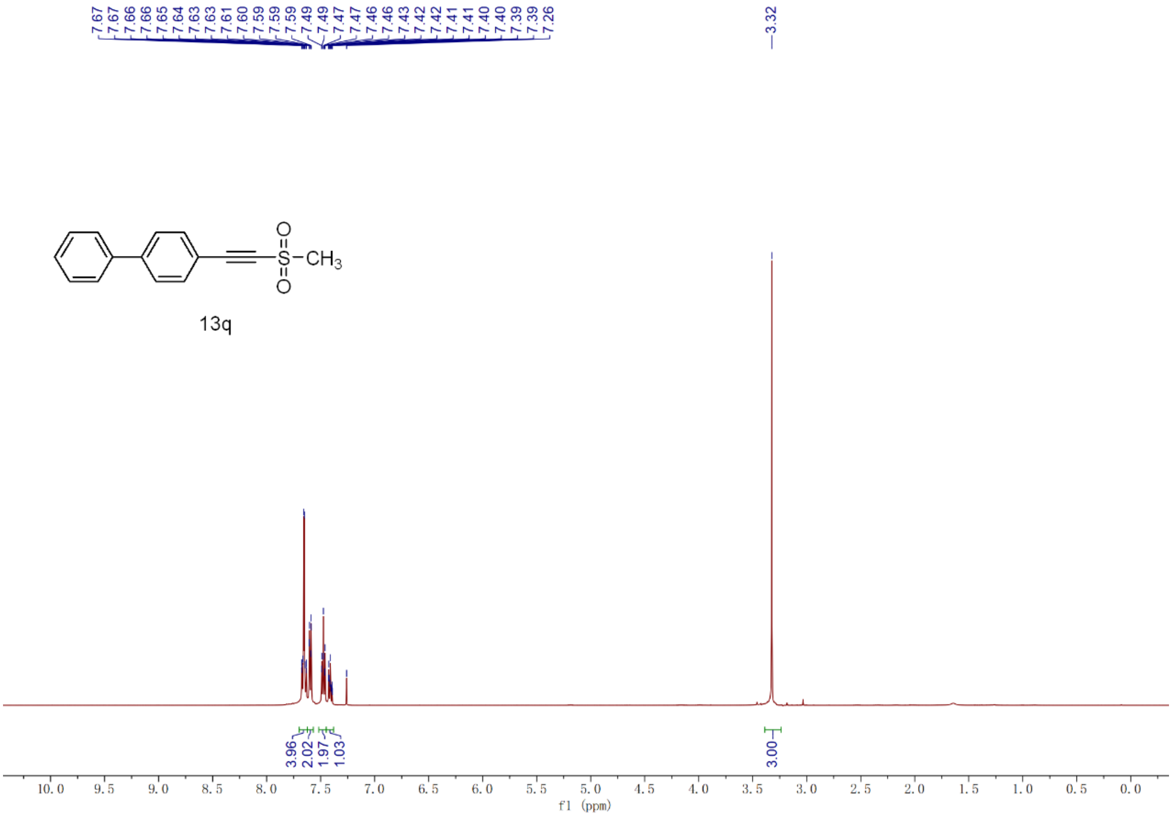

Supplementary Figure 27b | ^{13}C NMR (126 MHz, $CDCl_3$) of 4-((Methylsulfonyl)ethynyl)-1,1'-biphenyl (**13q**)

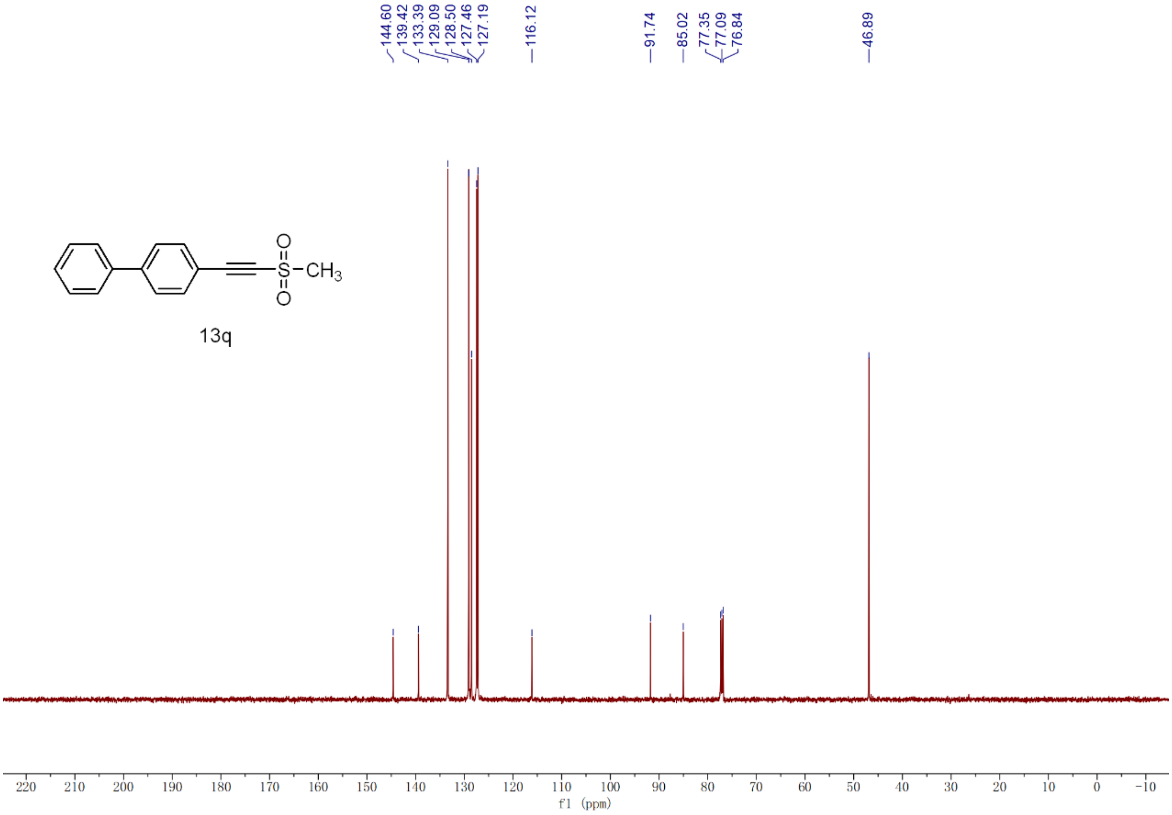

Supplementary Figure 28a | ^{1}H NMR (400 MHz, $CDCl_3$) of 1-Chloro-4-((methylsulfonyl)ethynyl)benzene (**13r**)

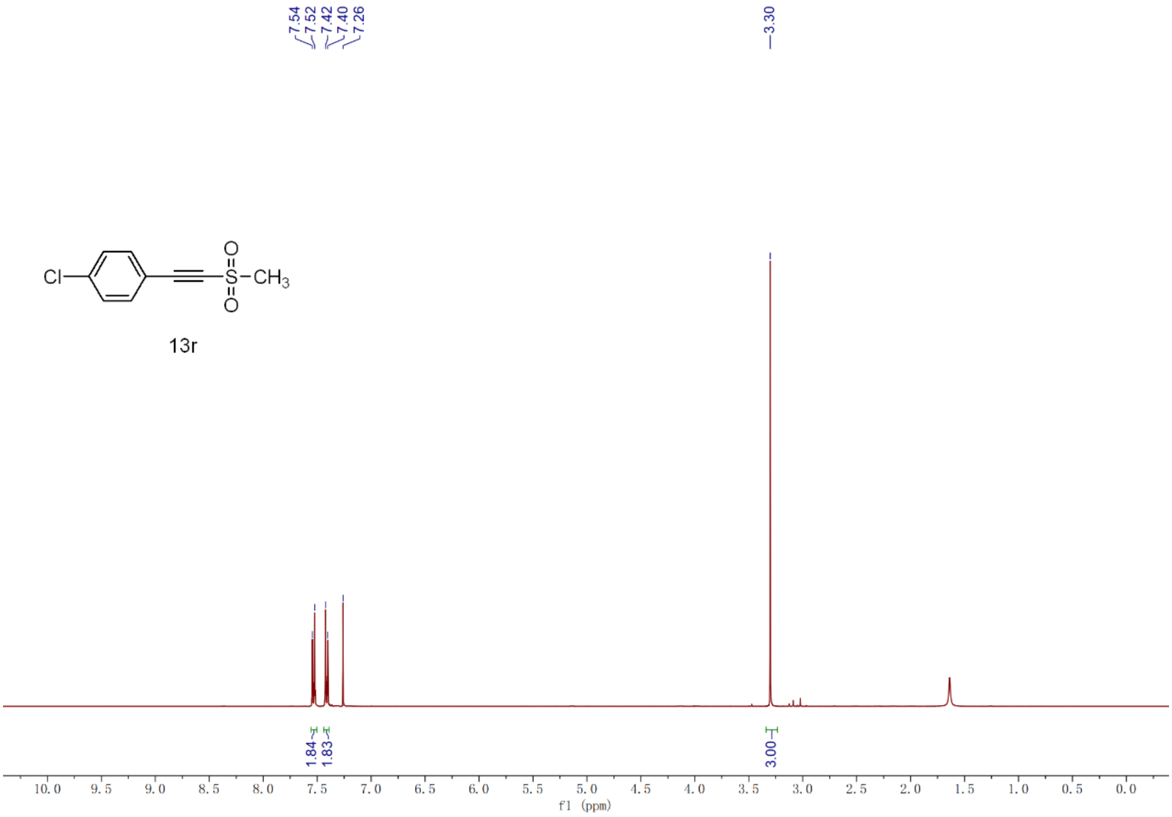

Supplementary Figure 28b | ^{13}C NMR (126 MHz, $CDCl_3$) of 1-Chloro-4-((methylsulfonyl)ethynyl)benzene (**13r**)

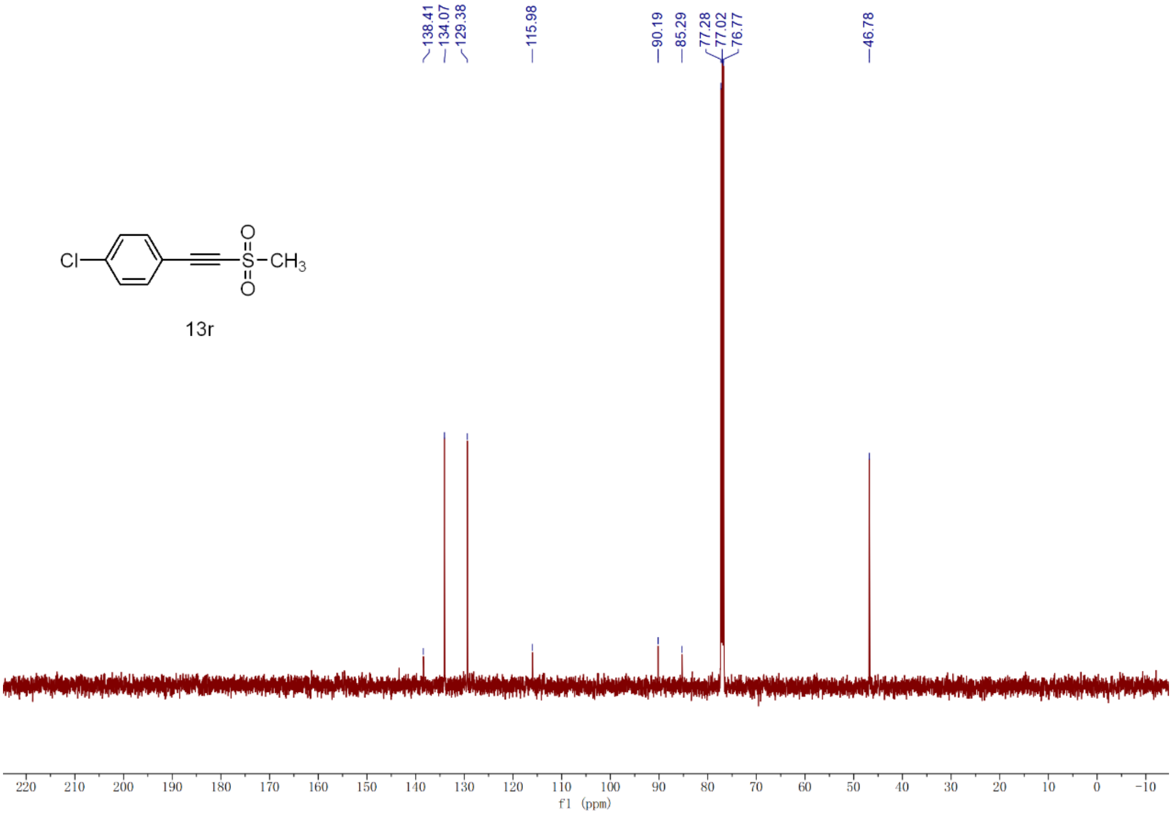

Supplementary Figure 29a | ^{1}H NMR (500 MHz, $CDCl_3$) of Methyl 4-((methylsulfonyl)ethynyl)benzoate (**13s**)

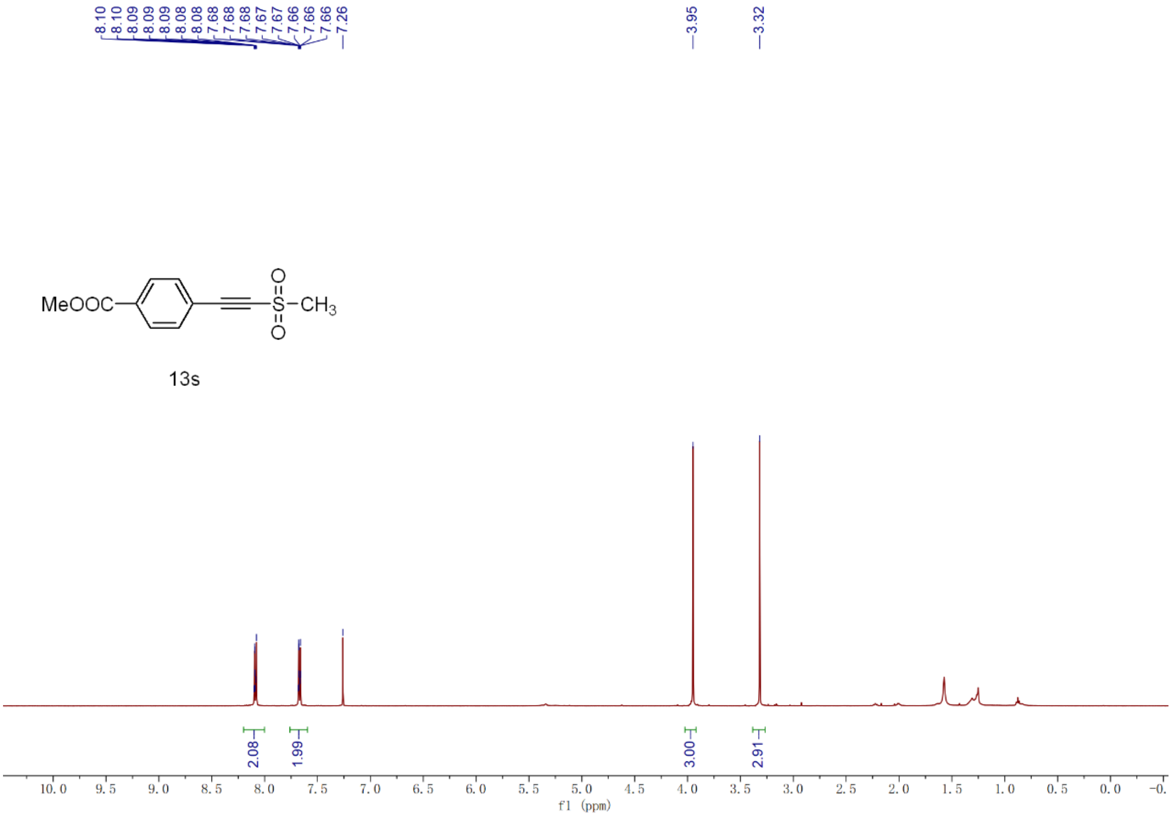

Supplementary Figure 29b | ^{13}C NMR (126 MHz, $CDCl_3$) of Methyl 4-((methylsulfonyl)ethynyl)benzoate (**13s**)

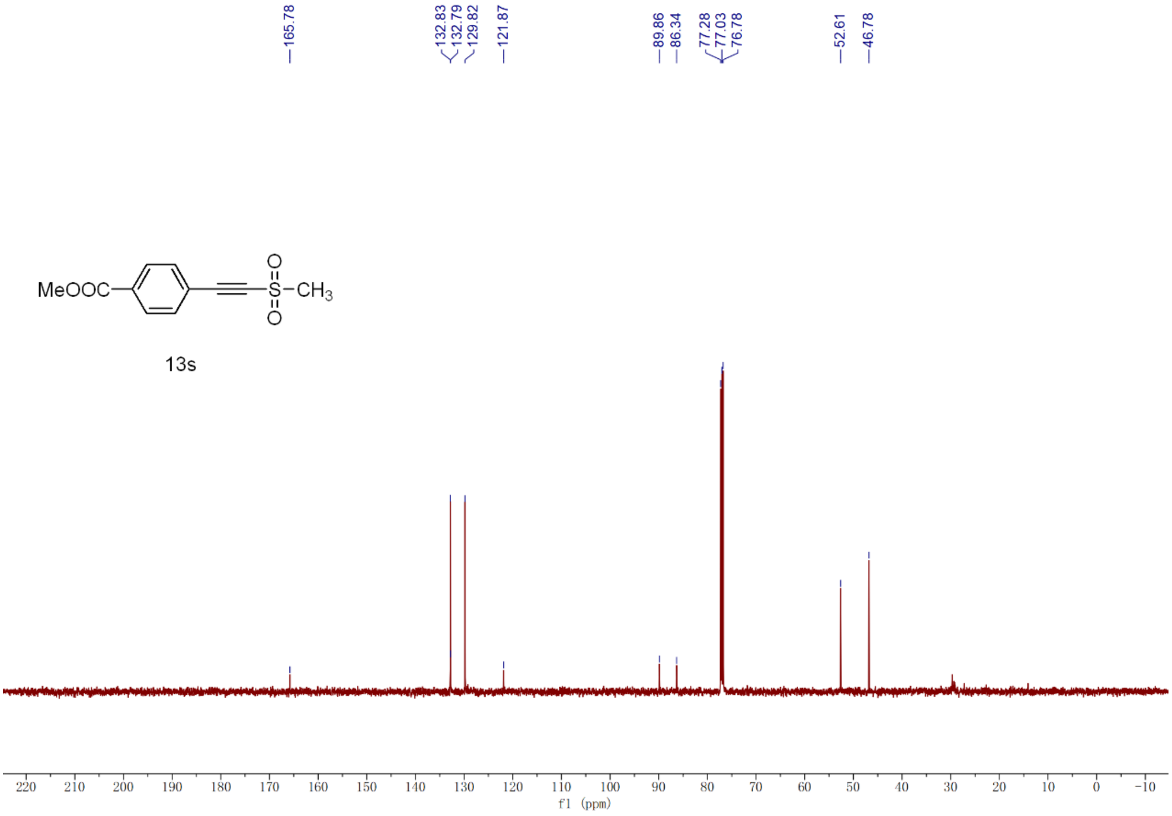

Supplementary Figure 30a | ^{1}H NMR (400 MHz, $CDCl_3$) of 1-Methyl-2-((methylsulfonyl)ethynyl)benzene (**13t**)

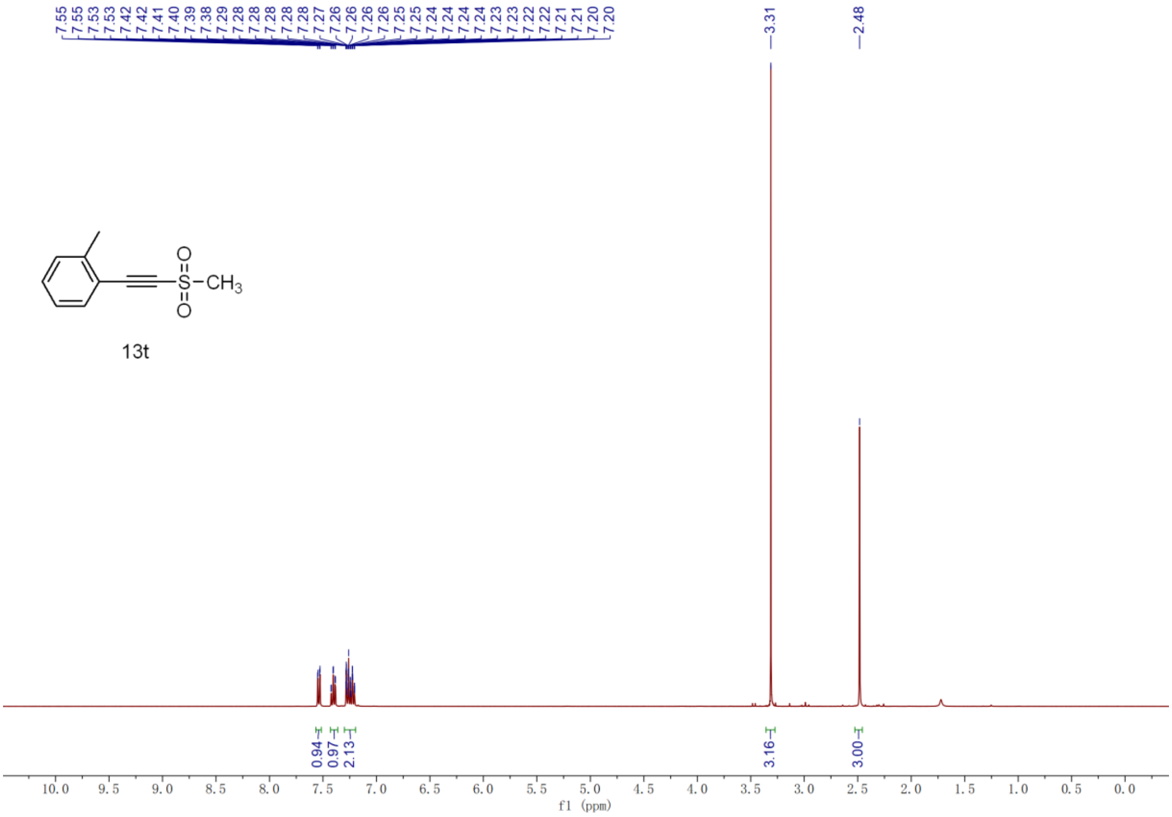

Supplementary Figure 30b | ^{13}C NMR (126 MHz, $CDCl_3$) of 1-Methyl-2-((methylsulfonyl)ethynyl)benzene (**13t**)

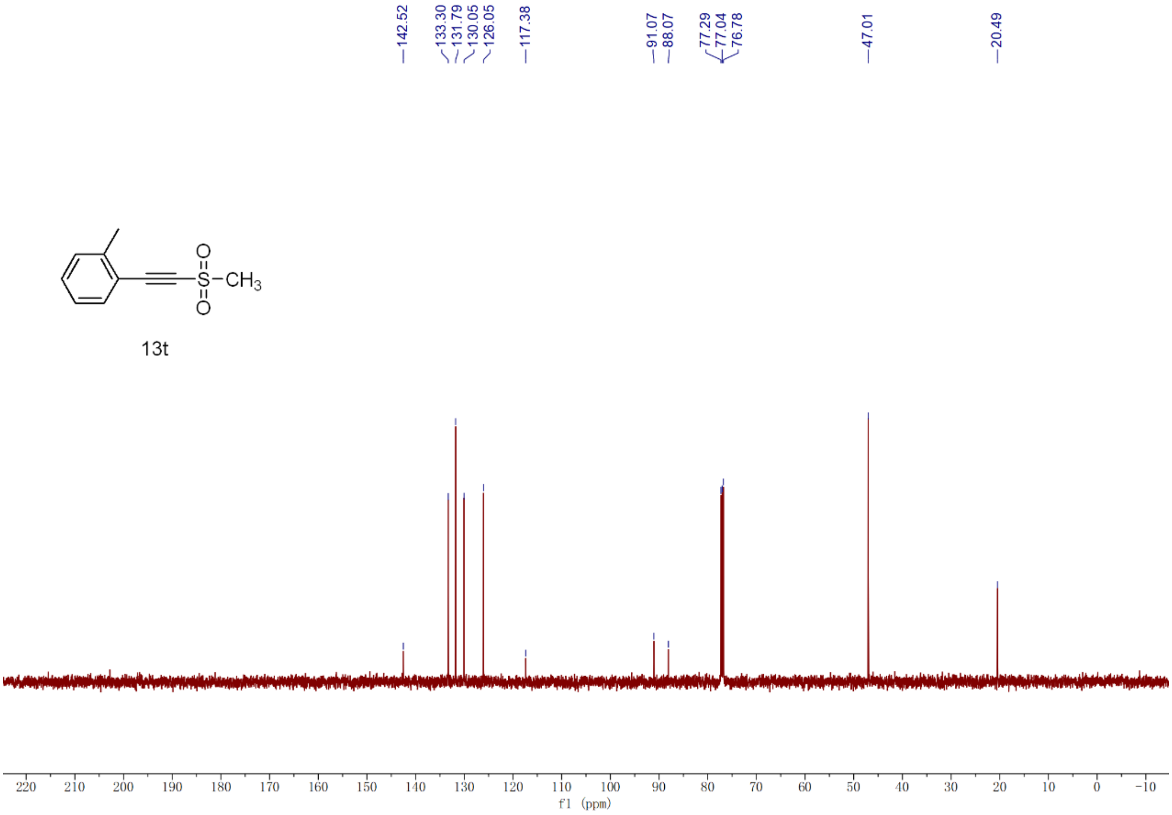

Supplementary Figure 31a | ^{1}H NMR (400 MHz, $CDCl_3$) of 1-Methoxy-2-((methylsulfonyl)ethynyl)benzene (**13u**)

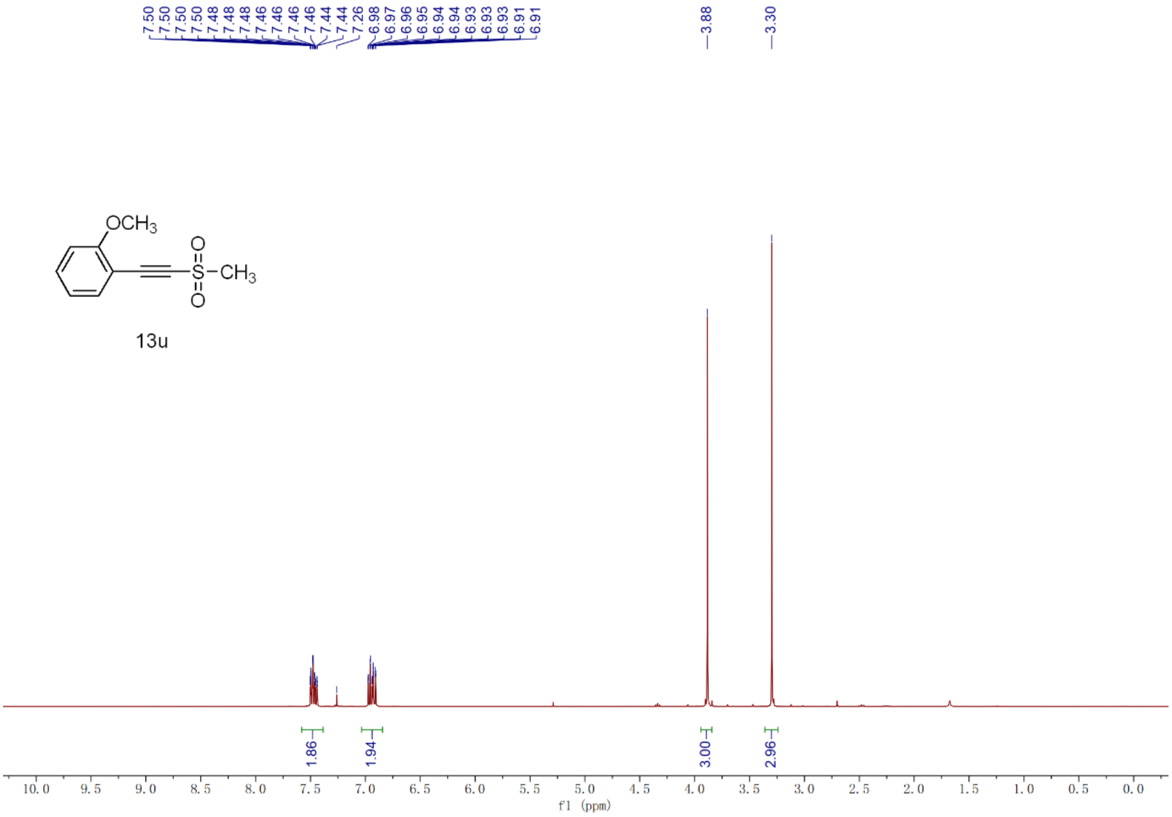

Supplementary Figure 31b | ^{13}C NMR (126 MHz, $CDCl_3$) of 1-Methoxy-2-((methylsulfonyl)ethynyl)benzene (**13u**)

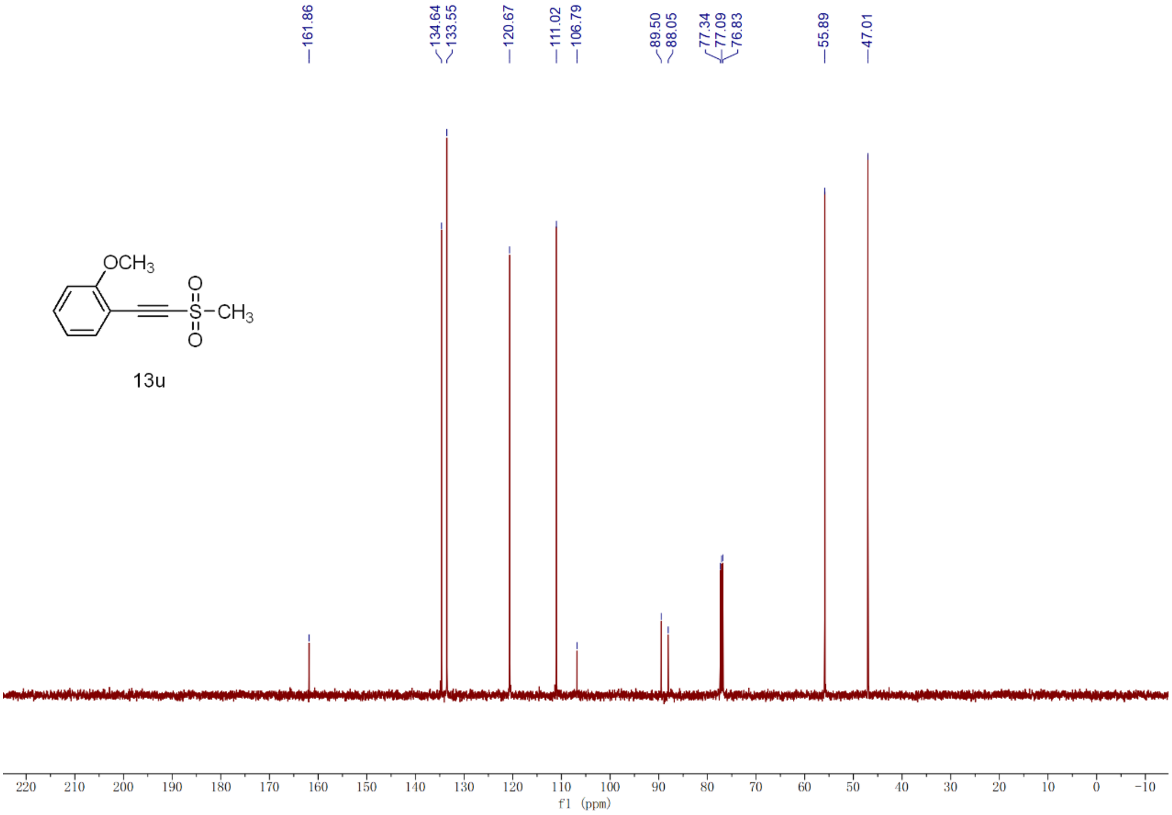

Supplementary Figure 32a | ^{1}H NMR (500 MHz, $CDCl_3$) of 2-((Methylsulfonyl)ethynyl)thiophene (**13v**)

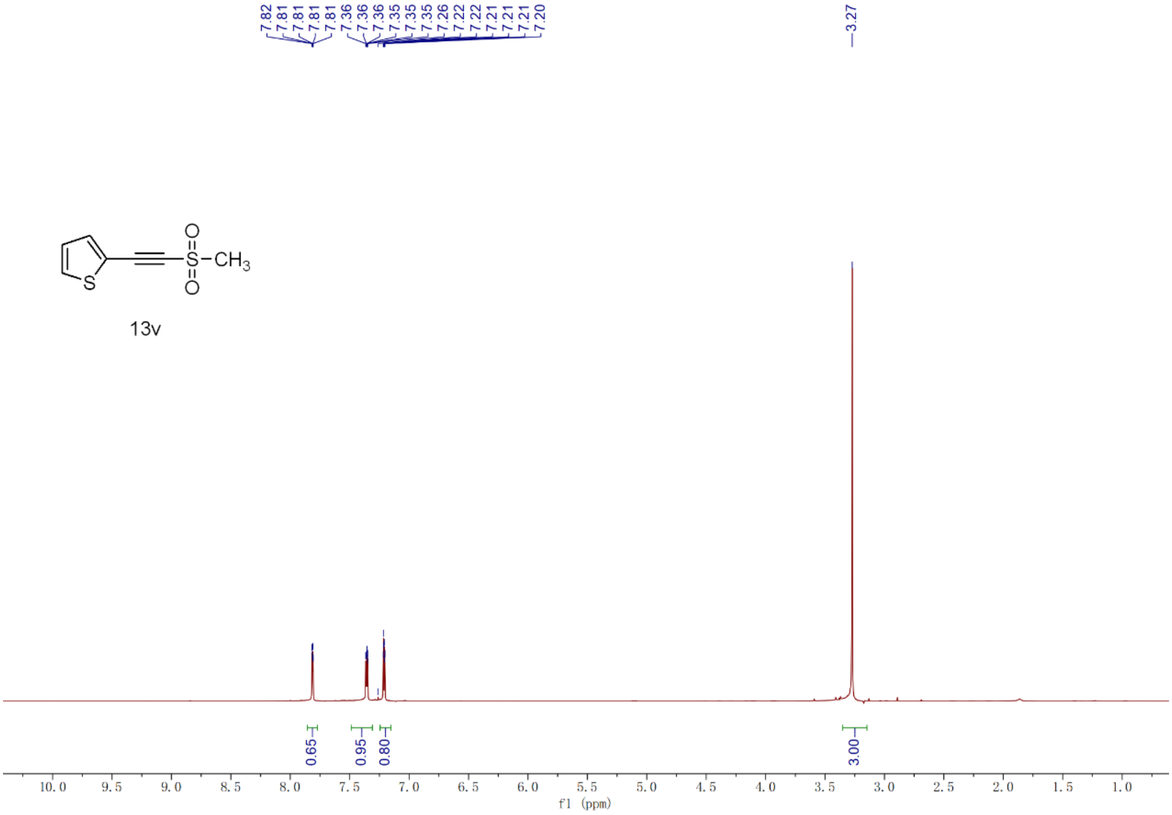

Supplementary Figure 32b | [13C]NMR (126 MHz, $CDCl_3$) of 2-((Methylsulfonyl)ethynyl)thiophene (**13v**)

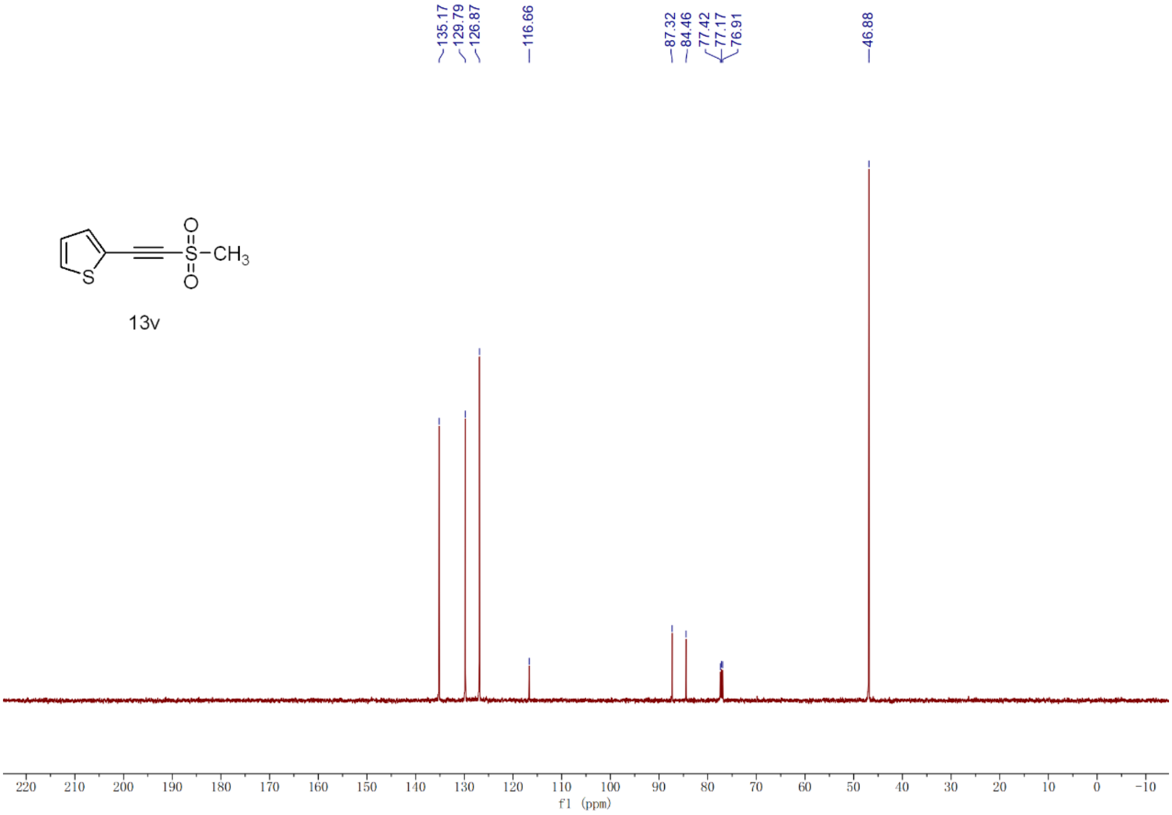

Supplementary Figure 33a | ^{1}H NMR (400 MHz, $CDCl_3$) of *S*-(fluoromethyl) benzothioate (**3a**)

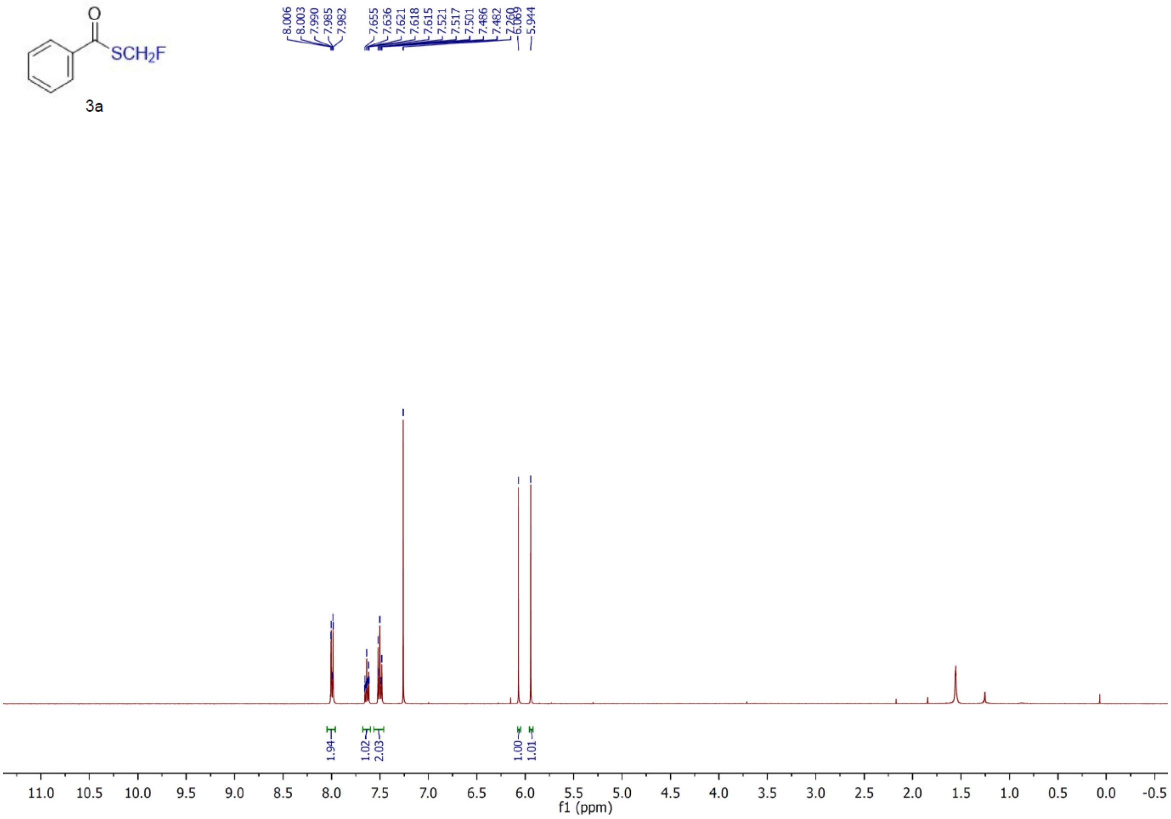

Supplementary Figure 33b | ^{13C} NMR (126 MHz, $CDCl_3$) of *S*-(fluoromethyl) benzothioate (**3a**)

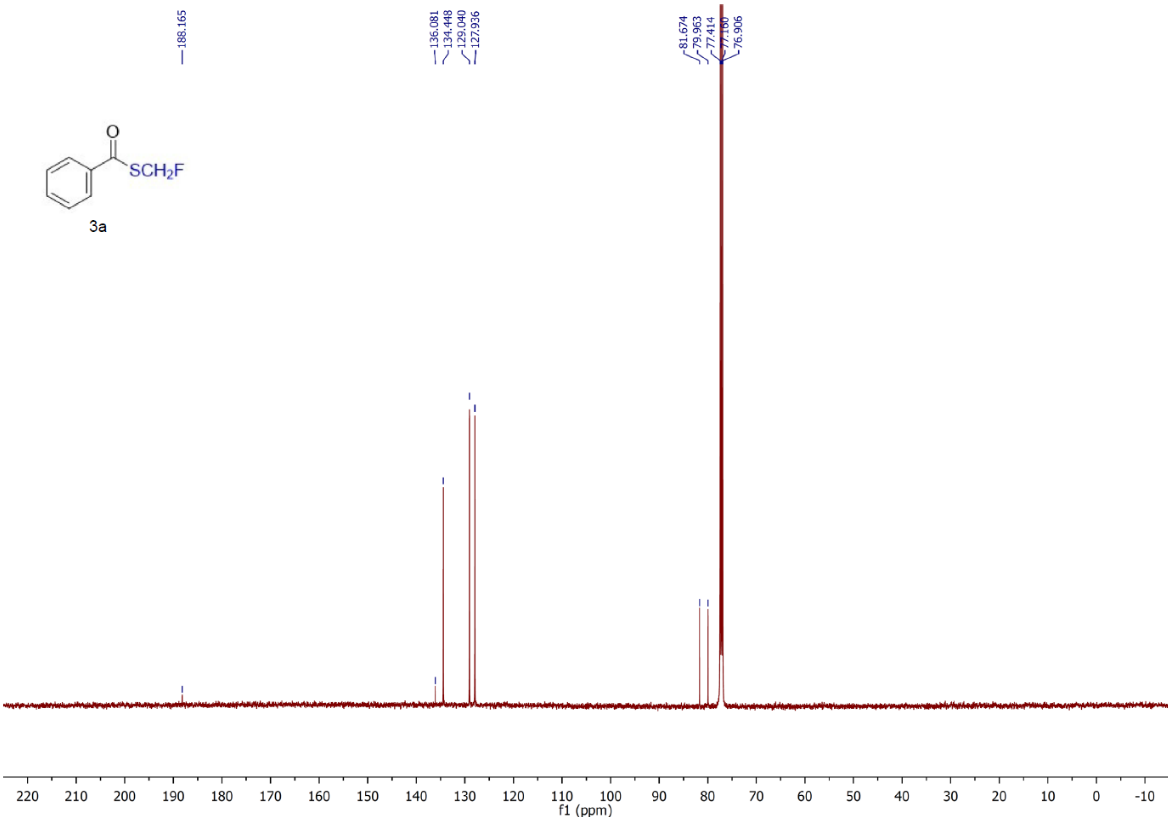

Supplementary Figure 33c | ^{19}F NMR (377 MHz, $CDCl_3$) of *S*-(fluoromethyl) benzothioate (**3a**)

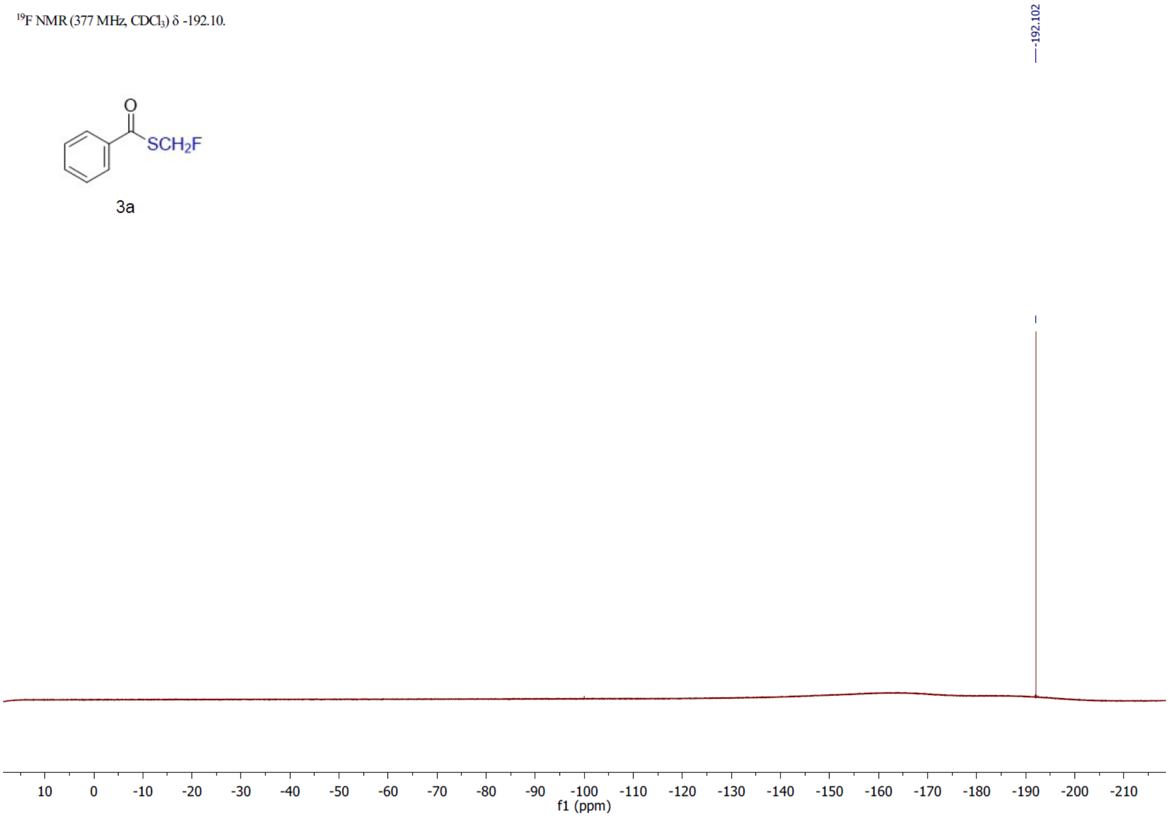

Supplementary Figure 34a | ^{1}H NMR (400 MHz, $CDCl_3$) of *S*-(fluoromethyl) 4-methoxybenzothioate (**3b**)

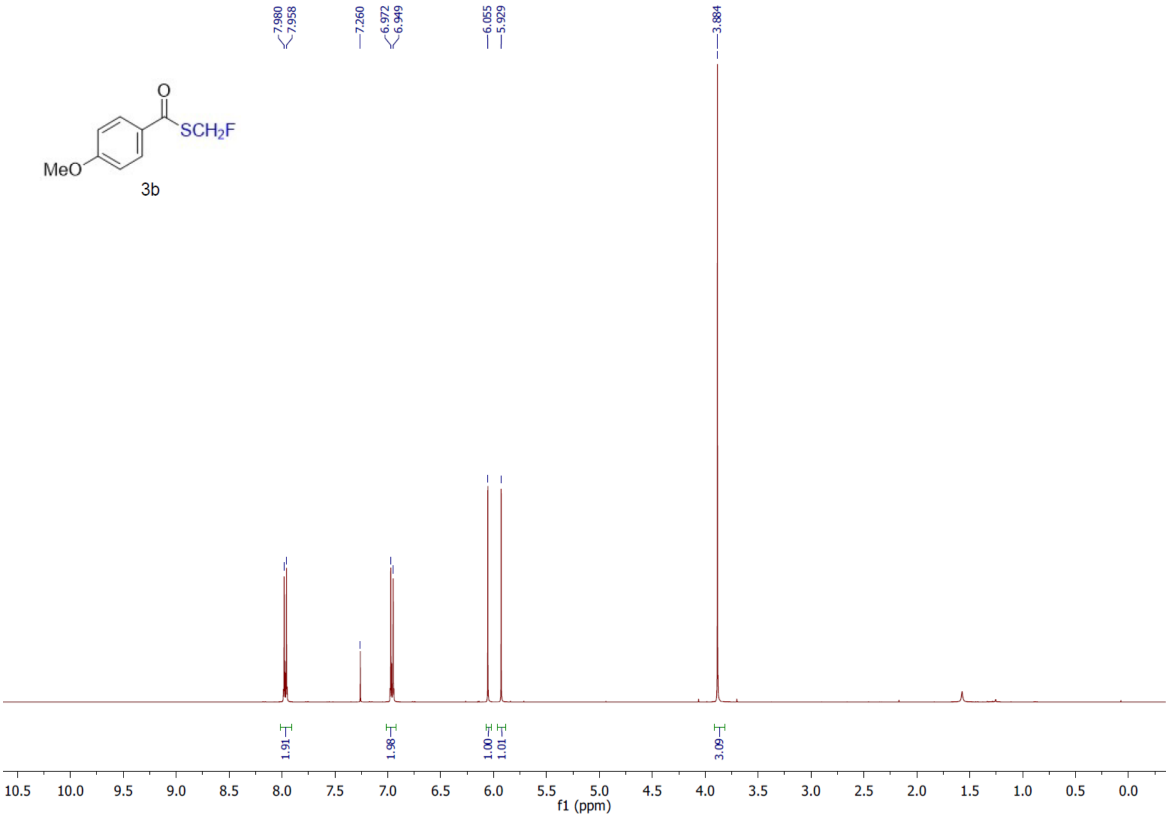

Supplementary Figure 34b | ^{13}C NMR (126 MHz, $CDCl_3$) of *S*-(fluoromethyl) 4-methoxybenzothioate (**3b**)

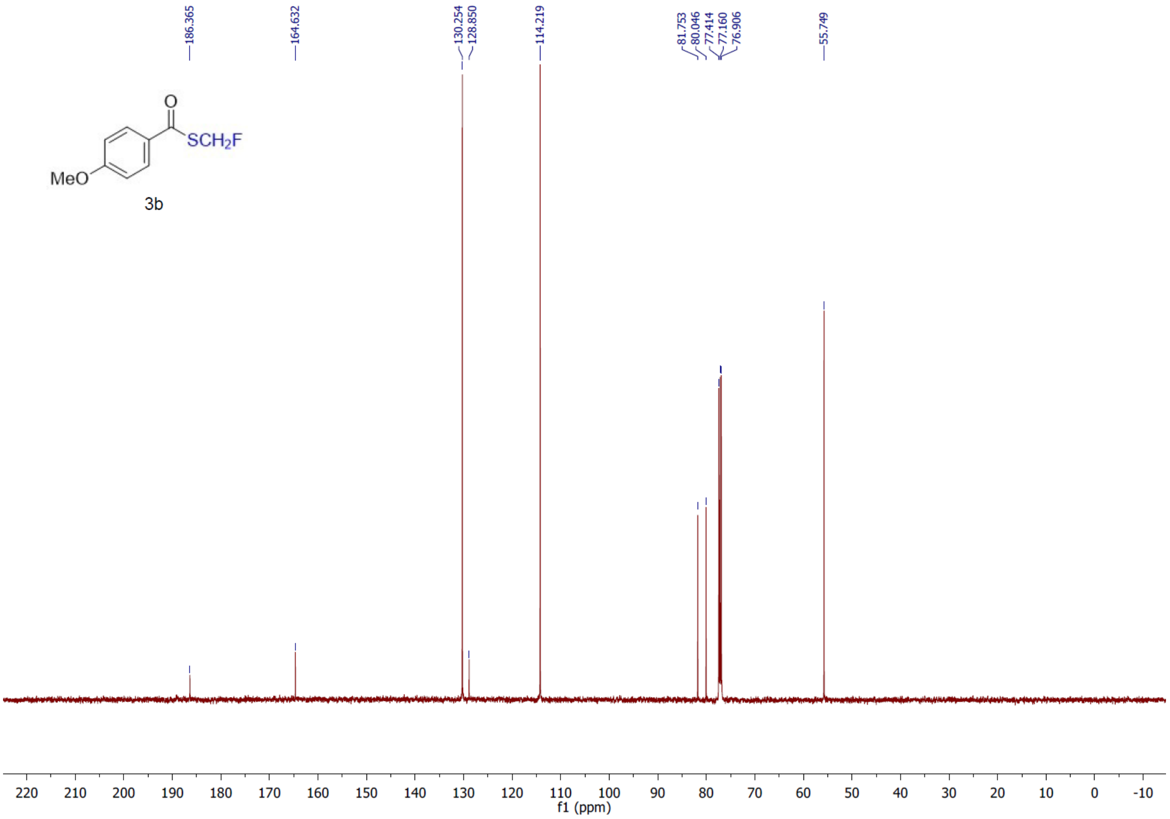

Supplementary Figure 34c | ^{19}F NMR (377 MHz, $CDCl_3$) of *S*-(fluoromethyl) 4-methoxybenzothioate (**3b**)

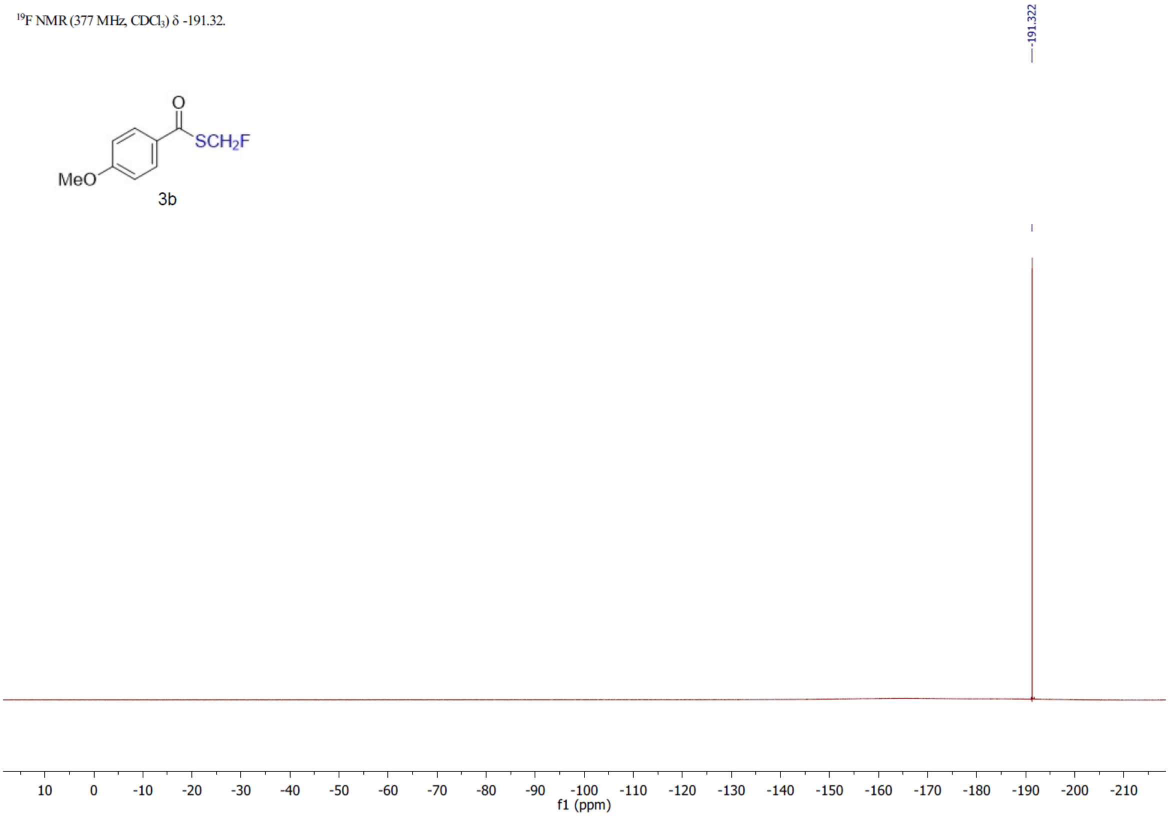

Supplementary Figure 35a | ^{1}H NMR (400 MHz, $CDCl_3$) of *S*-(fluoromethyl) 2-methoxybenzothioate (**3c**)

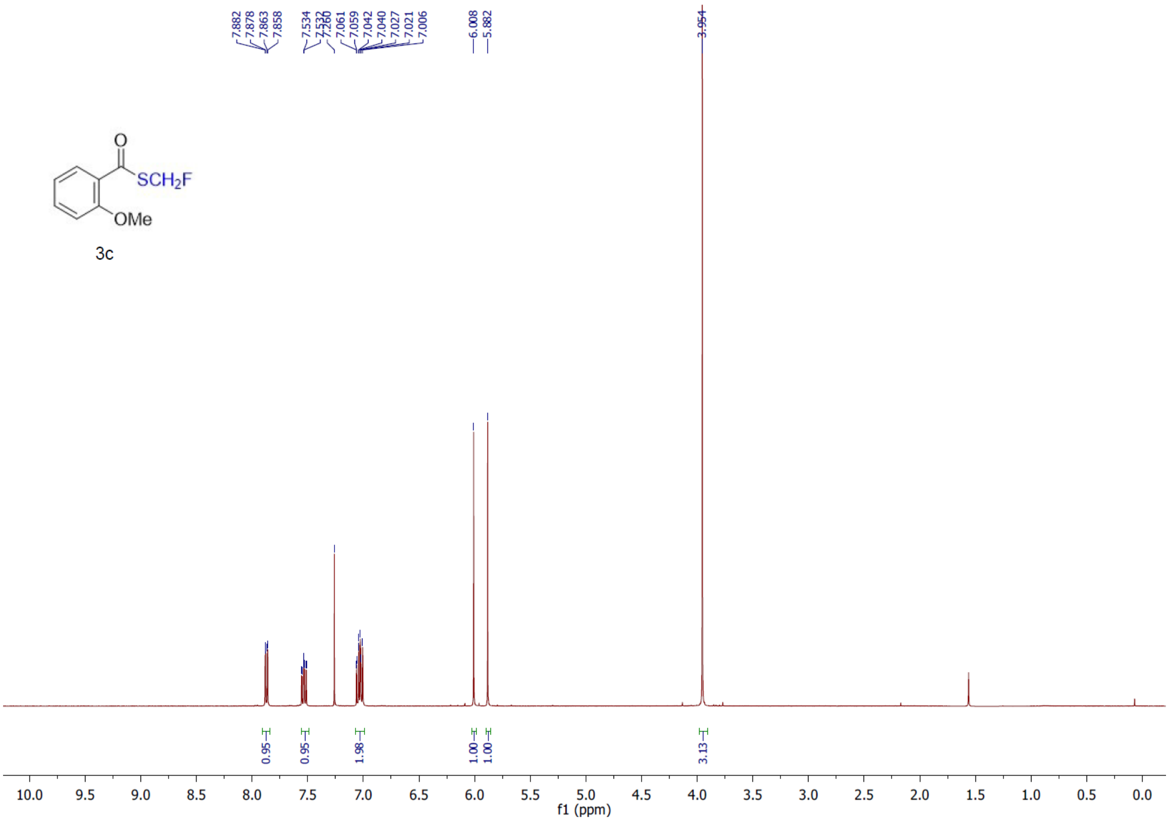

Supplementary Figure 35b | ^{13}C NMR (126 MHz, $CDCl_3$) of *S*-(fluoromethyl) 2-methoxybenzothioate (**3c**)

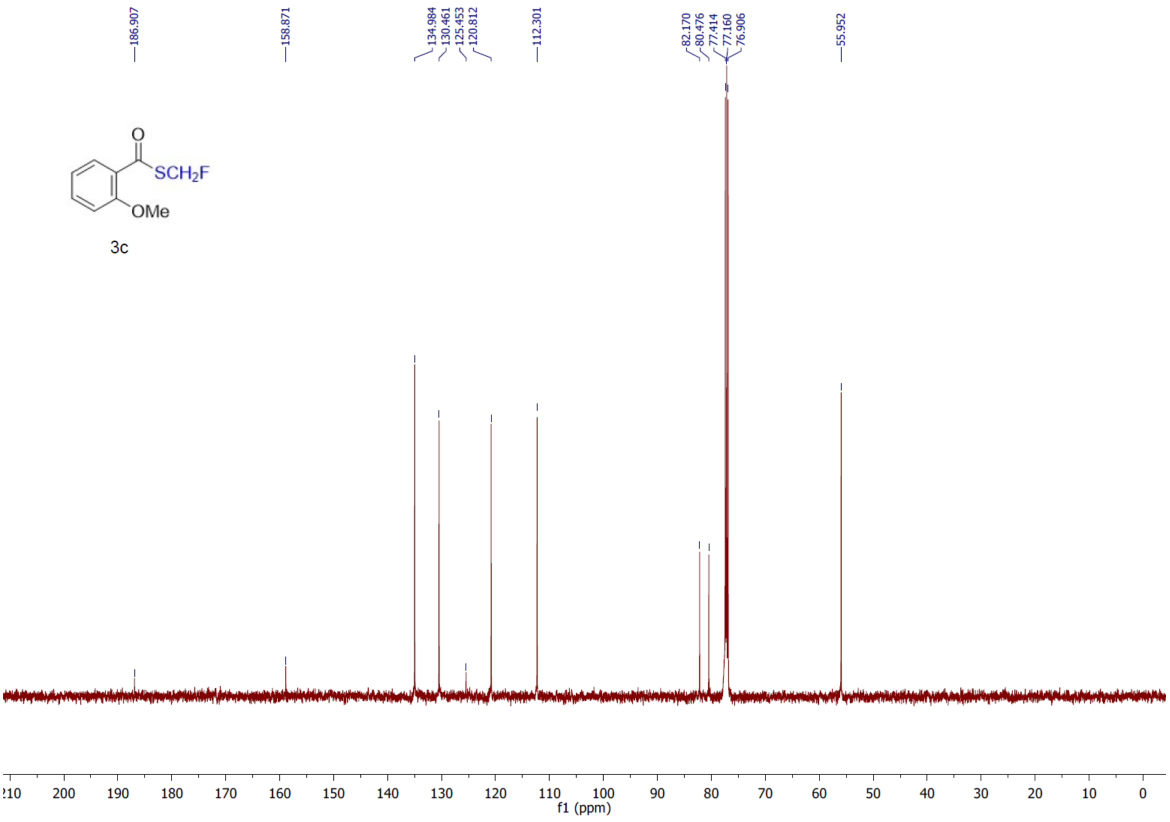

Supplementary Figure 35c | ^{19}F NMR (377 MHz, $CDCl_3$) of *S*-(fluoromethyl) 2-methoxybenzothioate (**3c**)

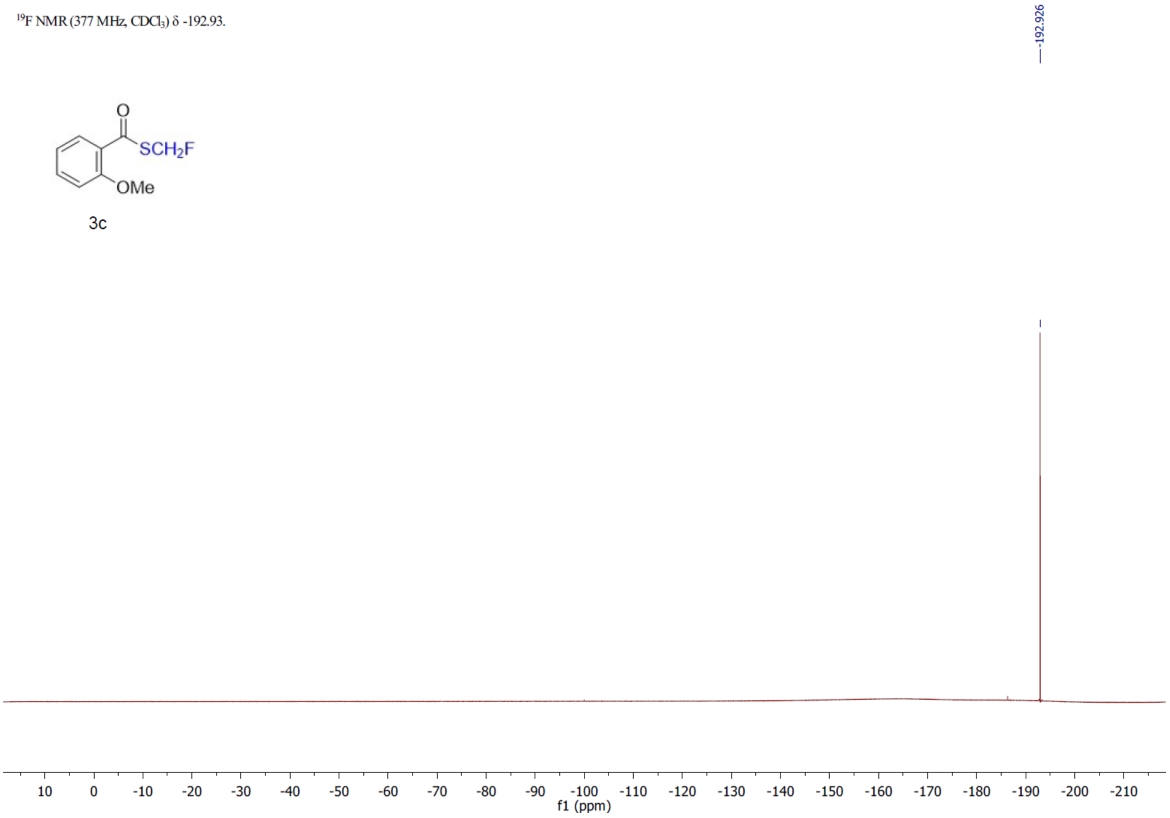

Supplementary Figure 36a | ^{1}H NMR (400 MHz, $CDCl_3$) of *S*-(fluoromethyl) 4-hydroxy-3-methoxybenzothioate (**3d**)

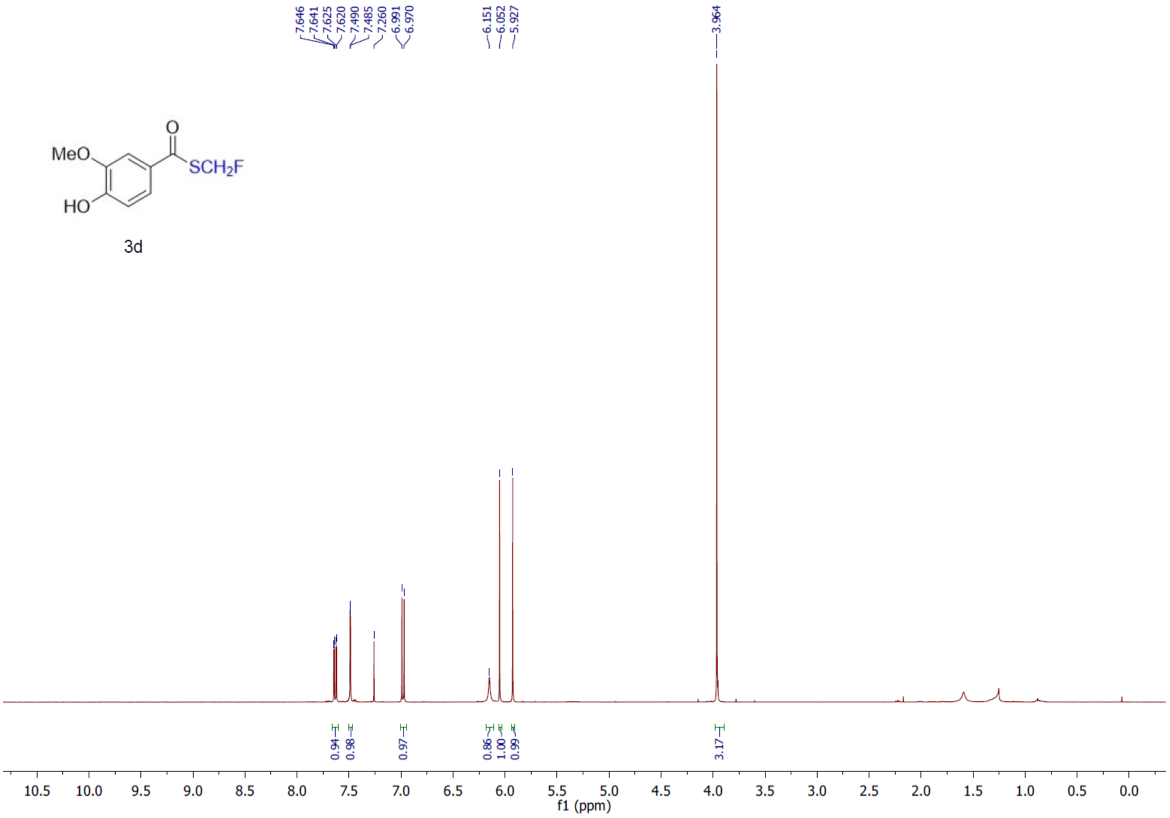

Supplementary Figure 36b | ^{13}C NMR (126 MHz, $CDCl_3$) of *S*-(fluoromethyl) 4-hydroxy-3-methoxybenzothioate (**3d**)

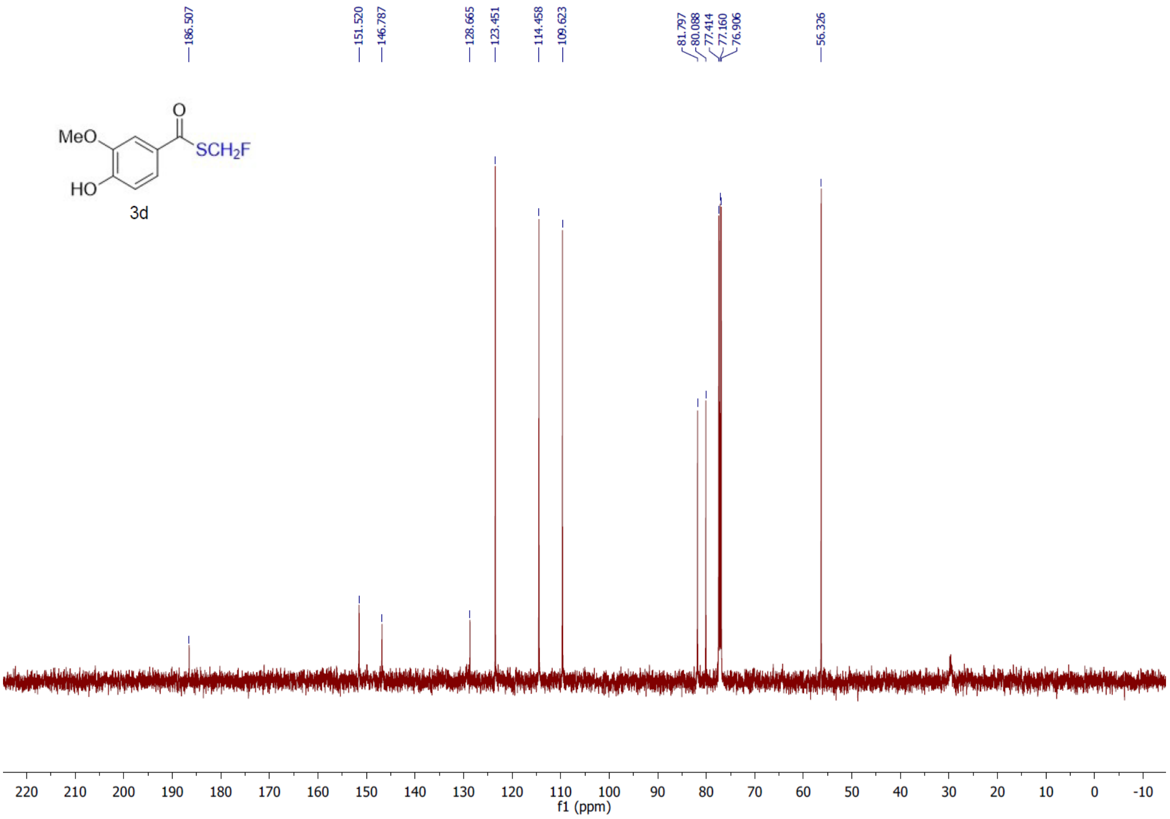

Supplementary Figure 36c | ^{19}F NMR (377 MHz, $CDCl_3$) of *S*-(fluoromethyl) 4-hydroxy-3-methoxybenzothioate (**3d**)

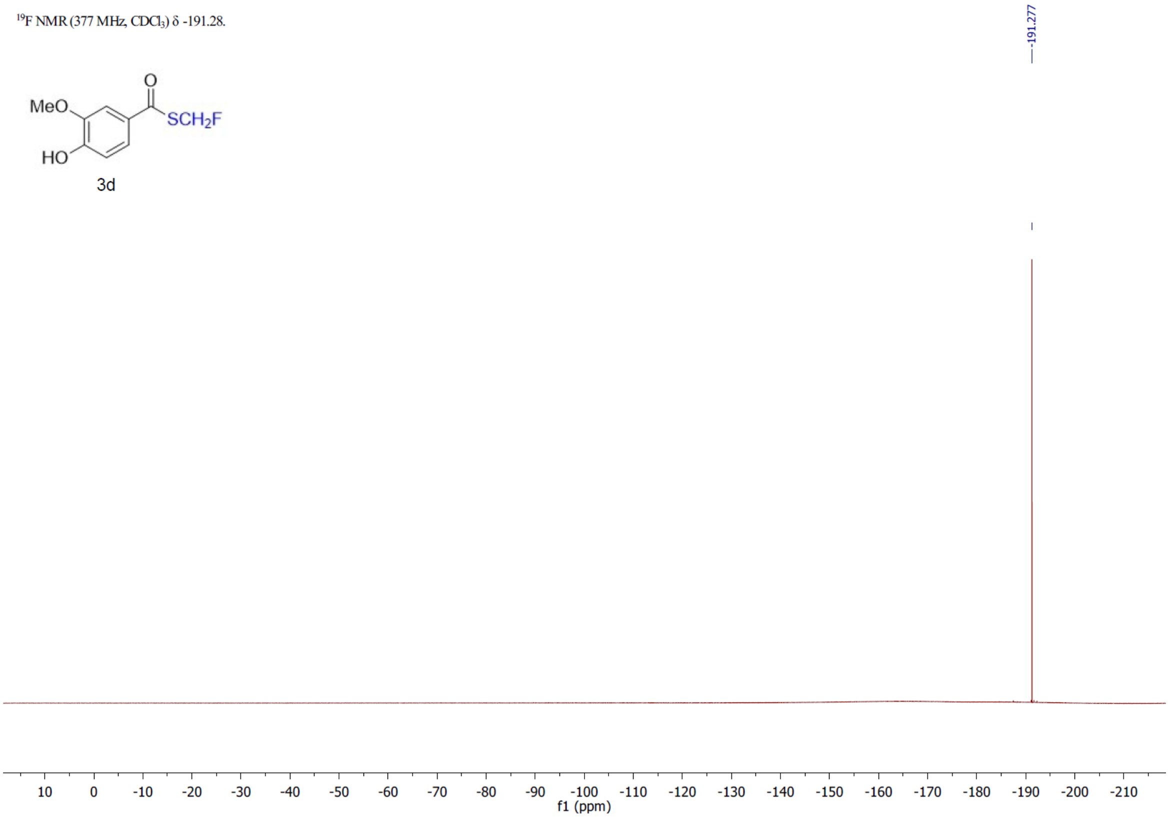

Supplementary Figure 37a | ^{1}H NMR (400 MHz, $CDCl_3$) of *S*-(fluoromethyl) 4-(benzyloxy)benzothioate (**3e**)

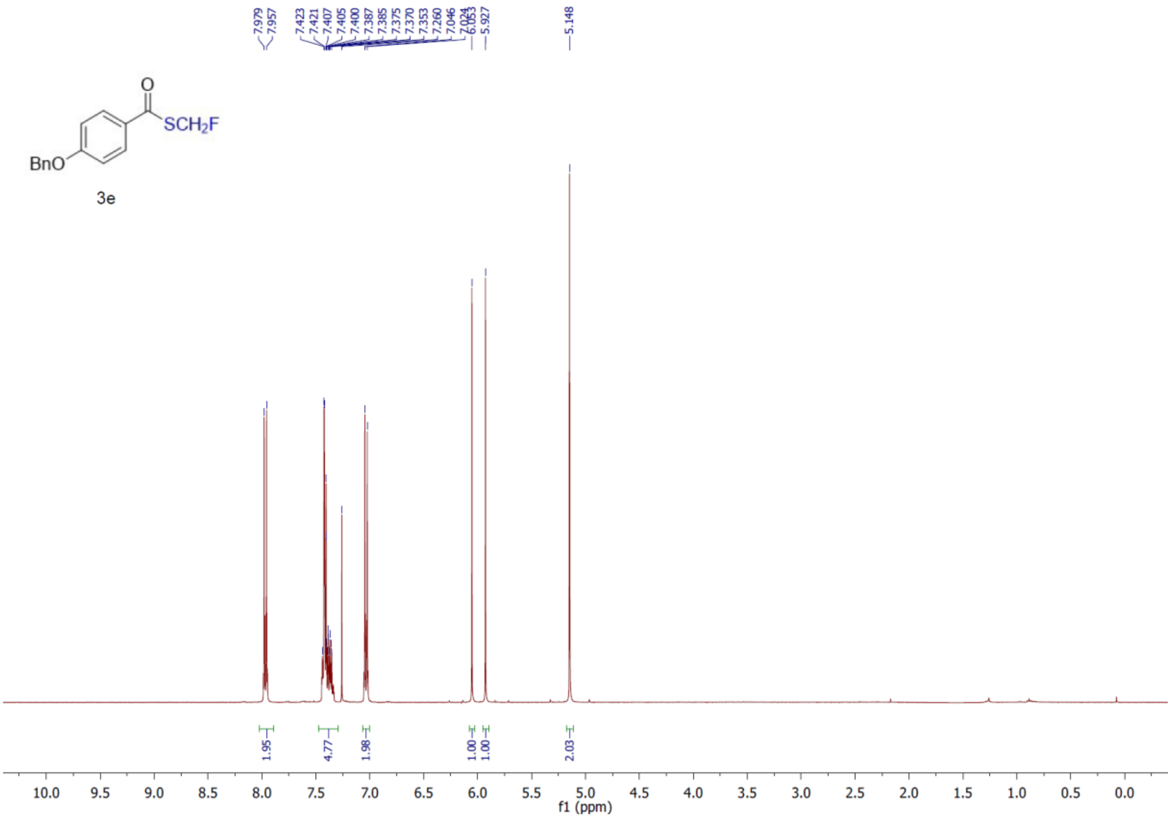

Supplementary Figure 37b | ^{13}C NMR (126 MHz, $CDCl_3$) of *S*-(fluoromethyl) 4-(benzyloxy)benzothioate (**3e**)

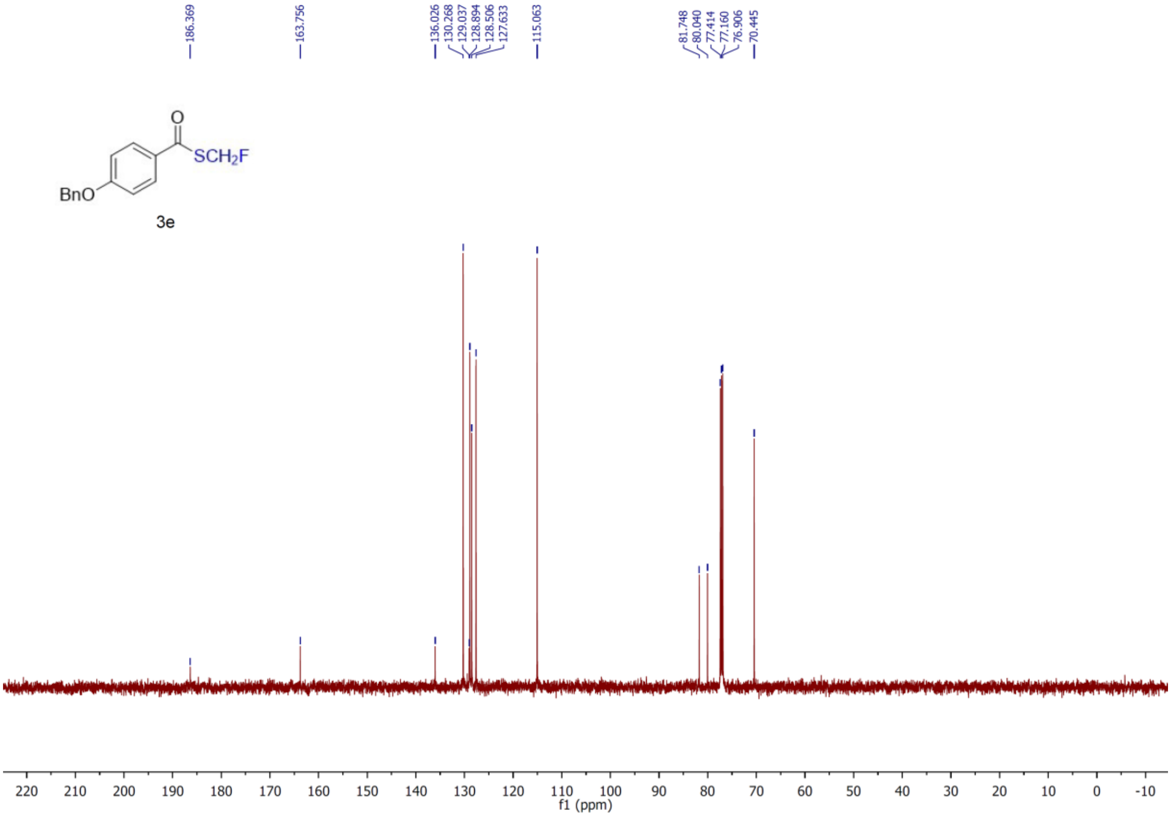

Supplementary Figure 37c | ^{19}F NMR (377 MHz, $CDCl_3$) of *S*-(fluoromethyl) 4-(benzyloxy)benzothioate (**3e**)

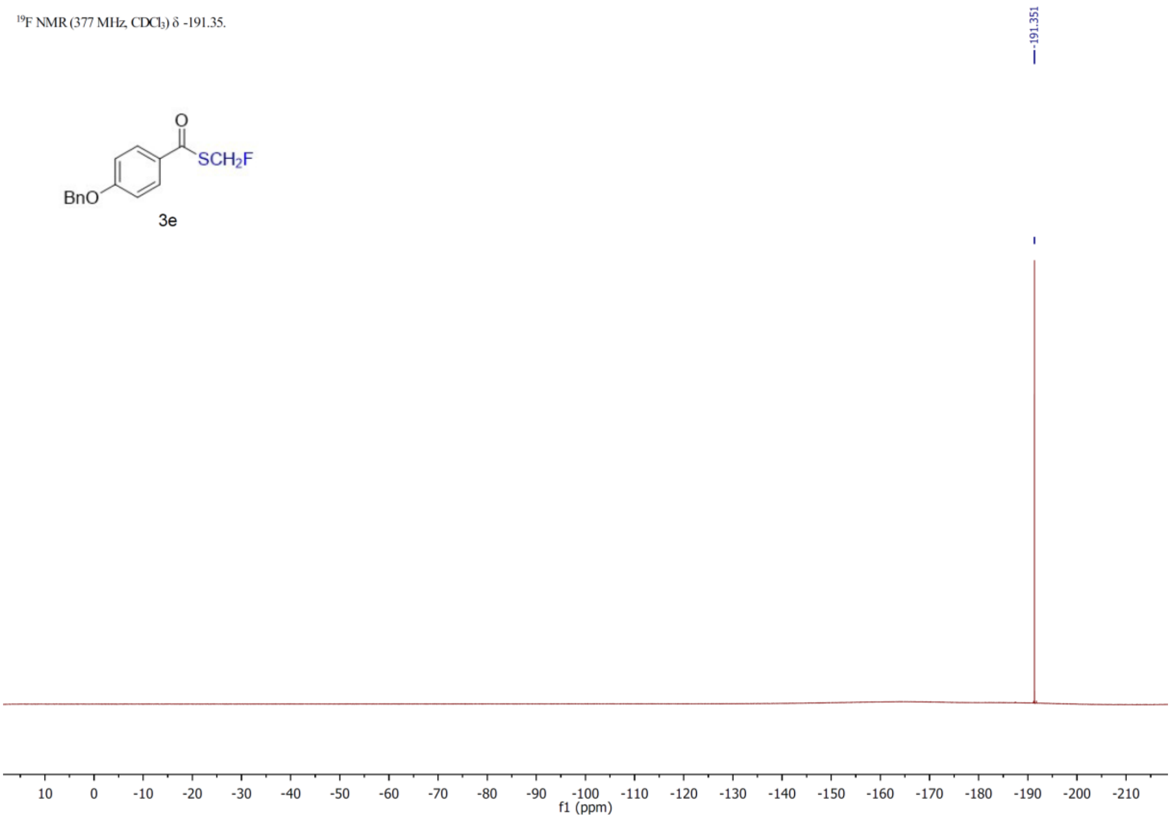

Supplementary Figure 38a | ^{1}H NMR (400 MHz, $CDCl_3$) of *S*-(fluoromethyl) 4-(methylthio)benzothioate (**3f**)

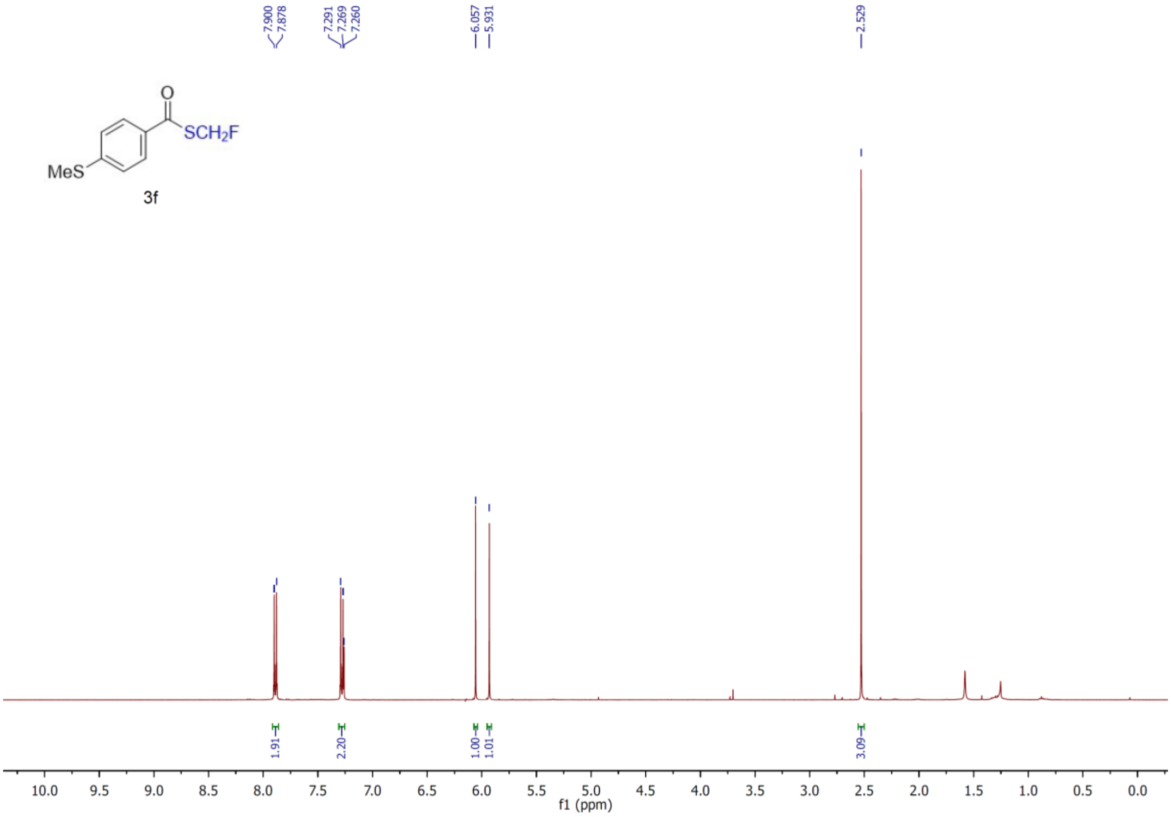

Supplementary Figure 38b | ^{13}C NMR (126 MHz, $CDCl_3$) of *S*-(fluoromethyl) 4-(methylthio)benzothioate **(3f)**

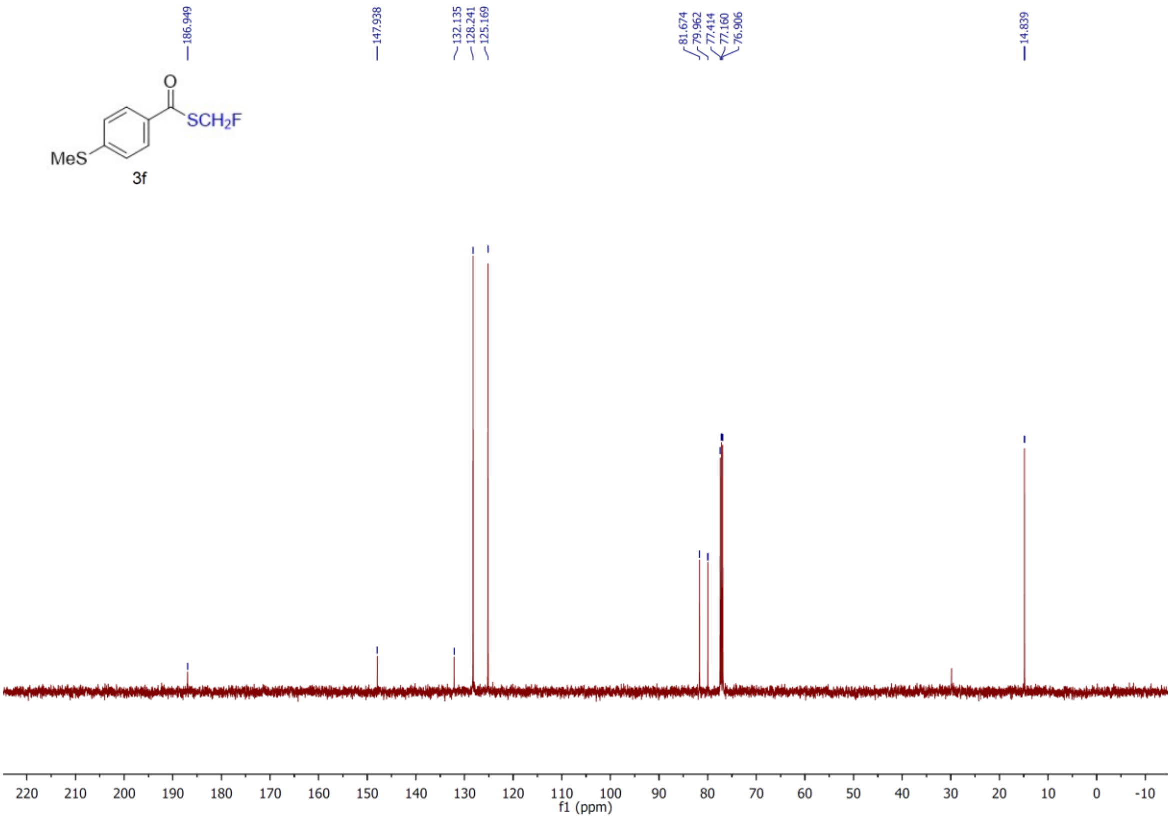

Supplementary Figure 38c | ^{19}F NMR (377 MHz, $CDCl_3$) of *S*-(fluoromethyl) 4-(methylthio)benzothioate **(3f)**

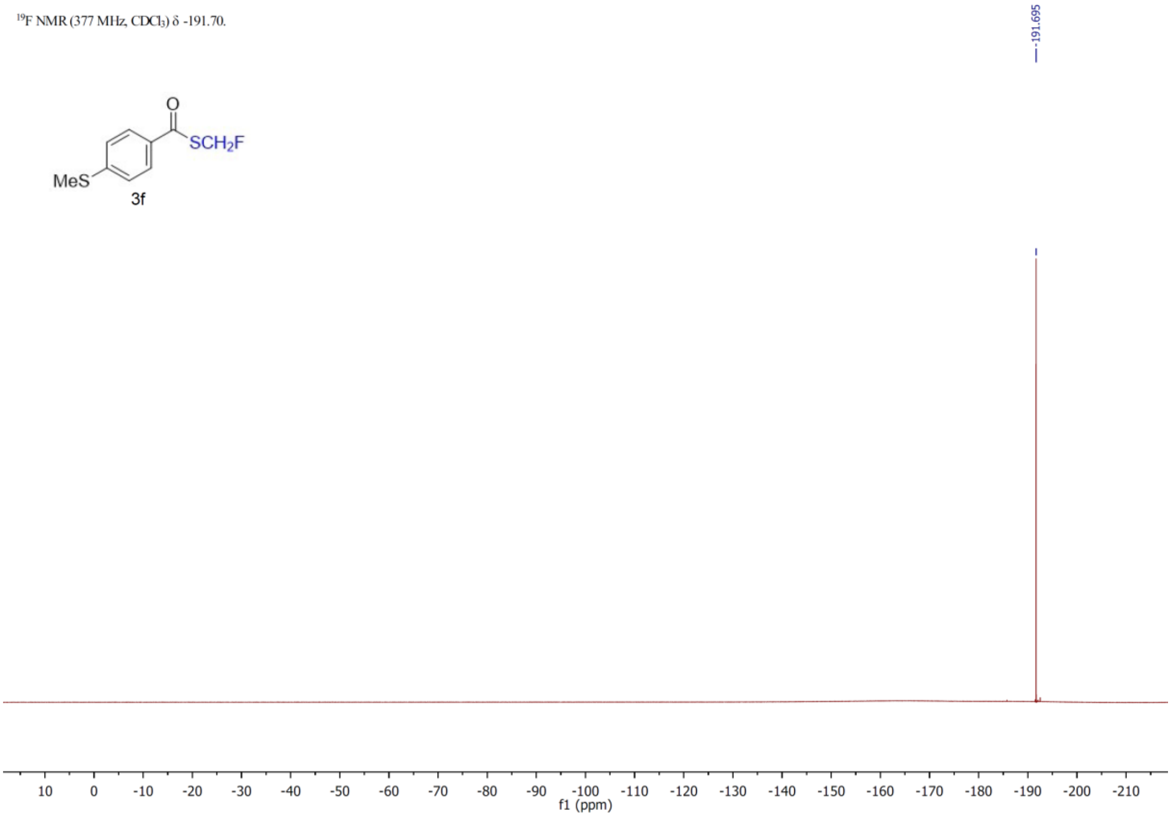

Supplementary Figure 39a | ^{1}H NMR (400 MHz, $CDCl_3$) of *S*-(fluoromethyl) [1,1'-biphenyl]-4-carbothioate (**3g**)

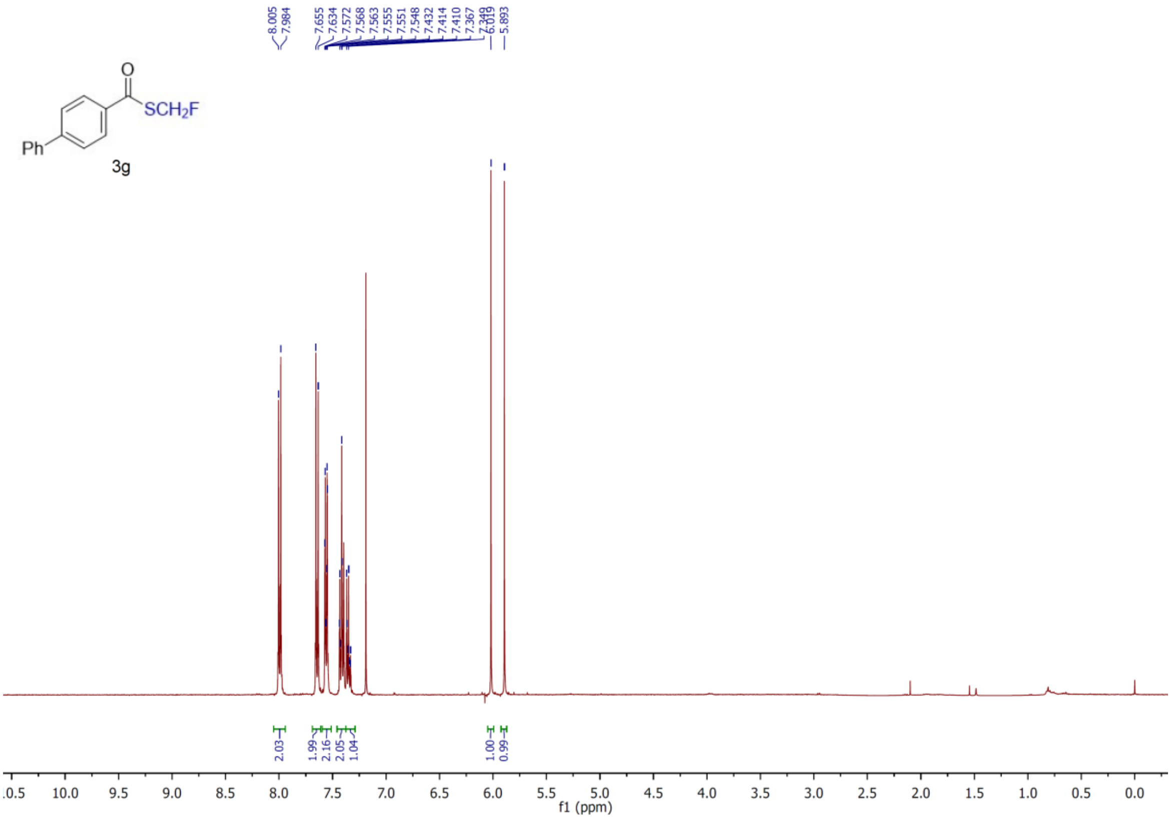

Supplementary Figure 39b | ^{13}C NMR (126 MHz, $CDCl_3$) of *S*-(fluoromethyl) [1,1'-biphenyl]-4-carbothioate (**3g**)

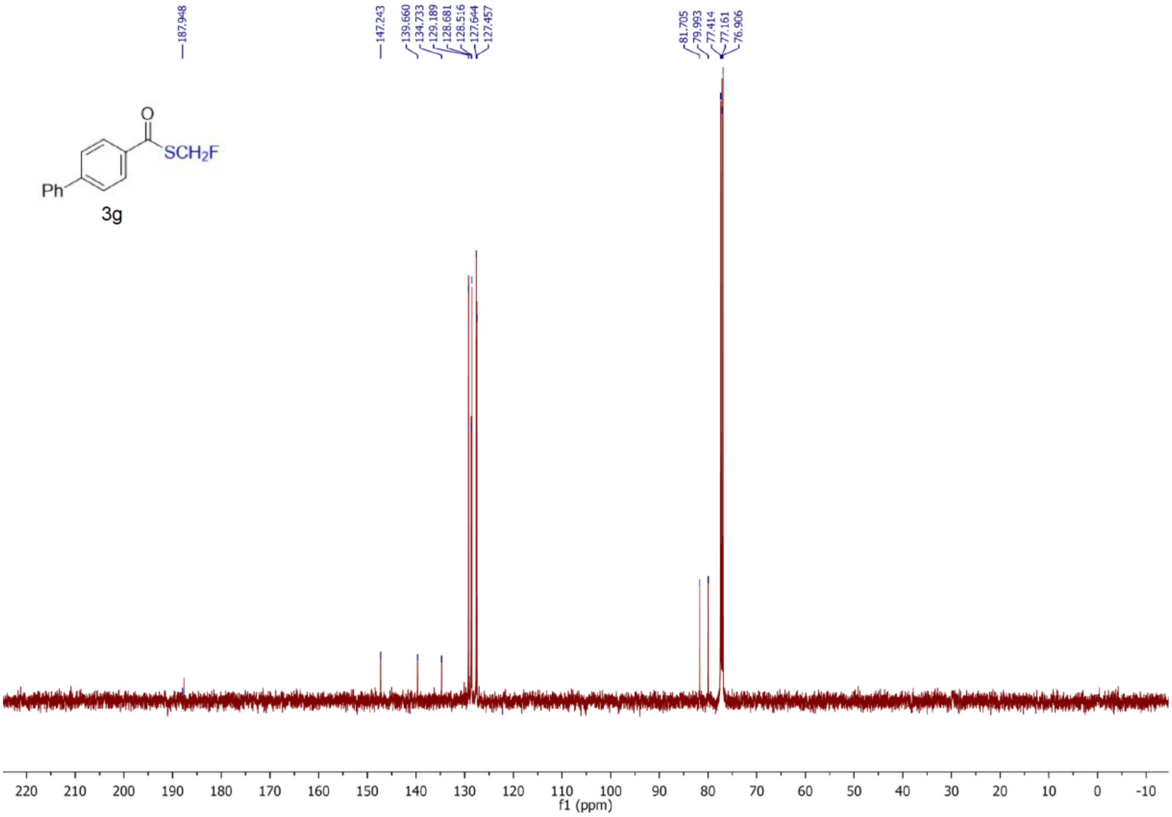

Supplementary Figure 39c | ^{19}F NMR (377 MHz, $CDCl_3$) of *S*-(fluoromethyl) [1,1'-biphenyl]-4-carbothioate (**3g**)

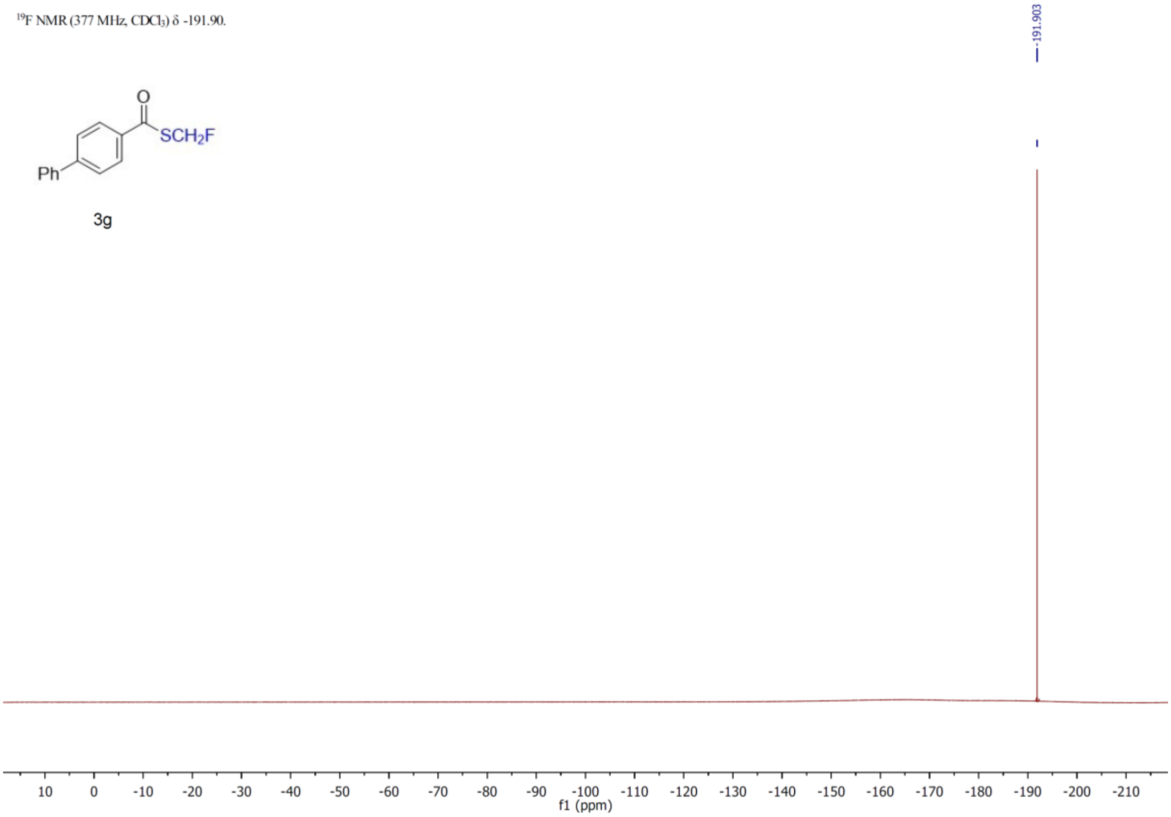

Supplementary Figure 40a | ^{1}H NMR (400 MHz, $CDCl_3$) of *S*-(fluoromethyl) 4-chlorobenzothioate (**3h**)

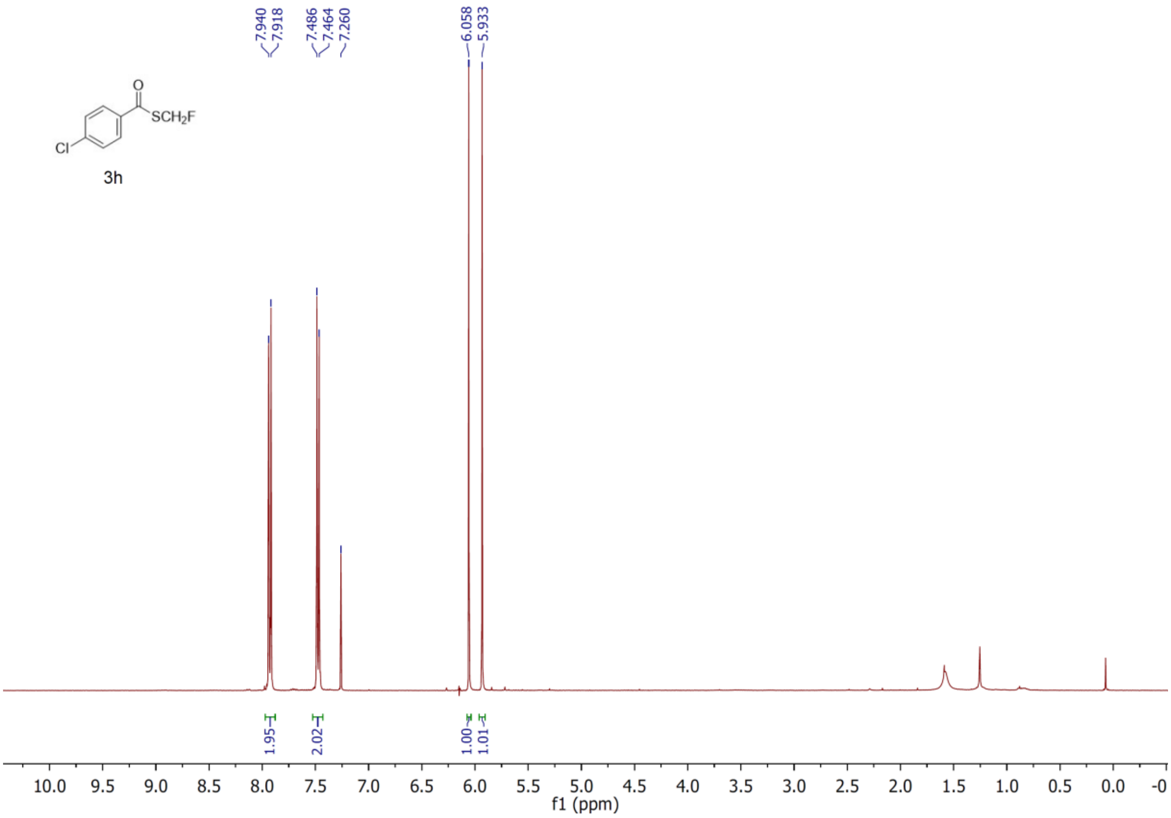

Supplementary Figure 40b | ^{13}C NMR (126 MHz, $CDCl_3$) of *S*-(fluoromethyl) 4-chlorobenzothioate (**3h**)

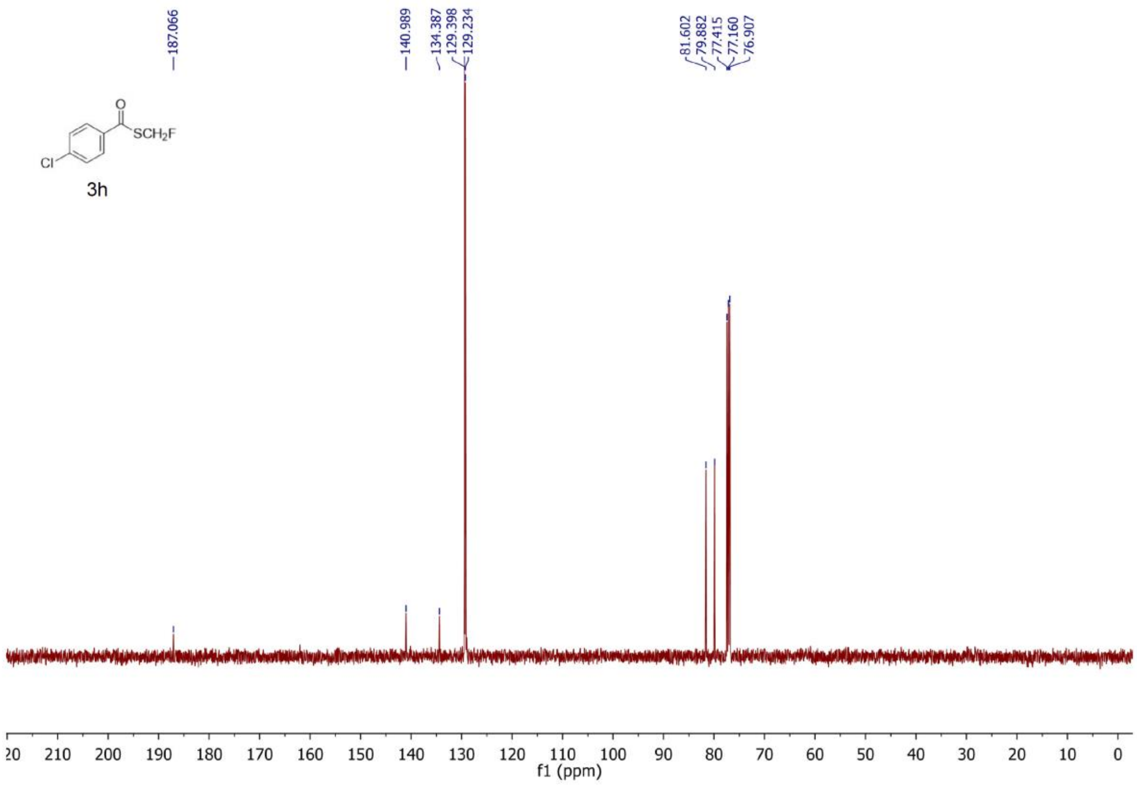

Supplementary Figure 40c | ^{19}F NMR (377 MHz, $CDCl_3$) of *S*-(fluoromethyl) 4-chlorobenzothioate (**3h**)

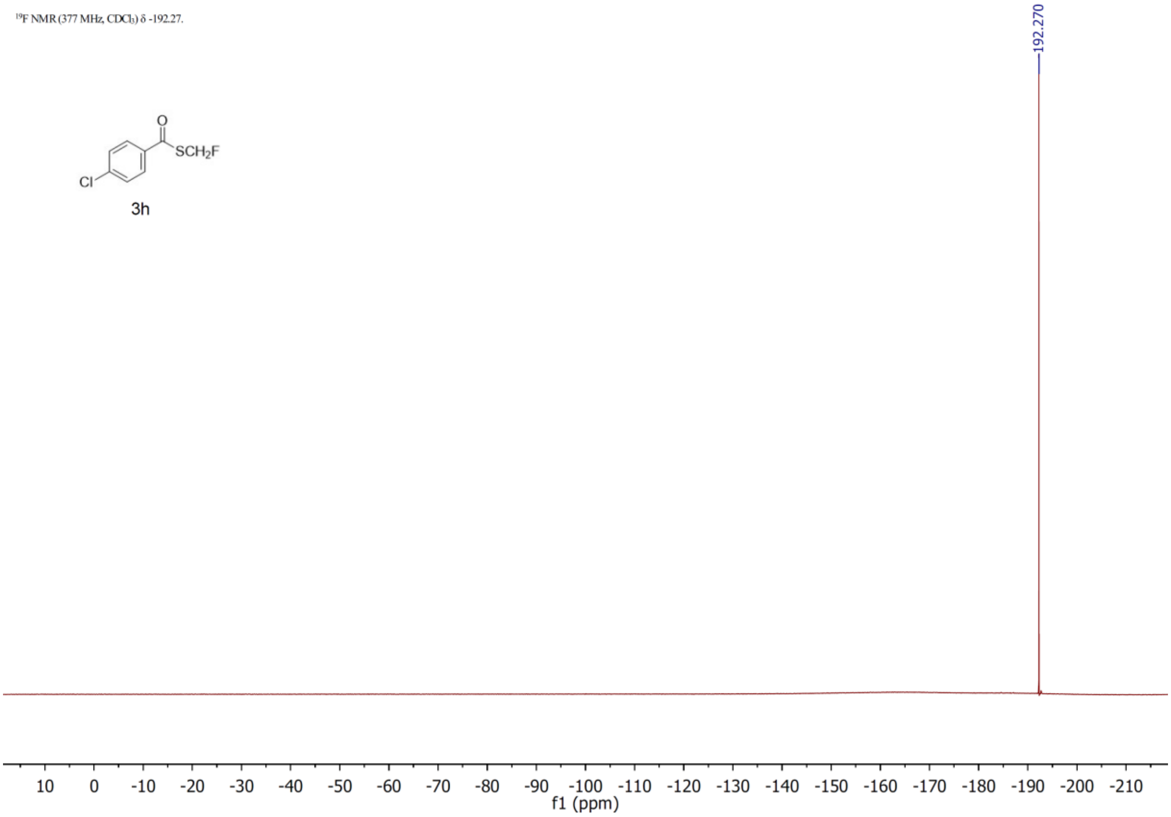

Supplementary Figure 41a | ^{1}H NMR (400 MHz, $CDCl_3$) of *S*-(fluoromethyl) 4-bromobenzothioate (**3i**)

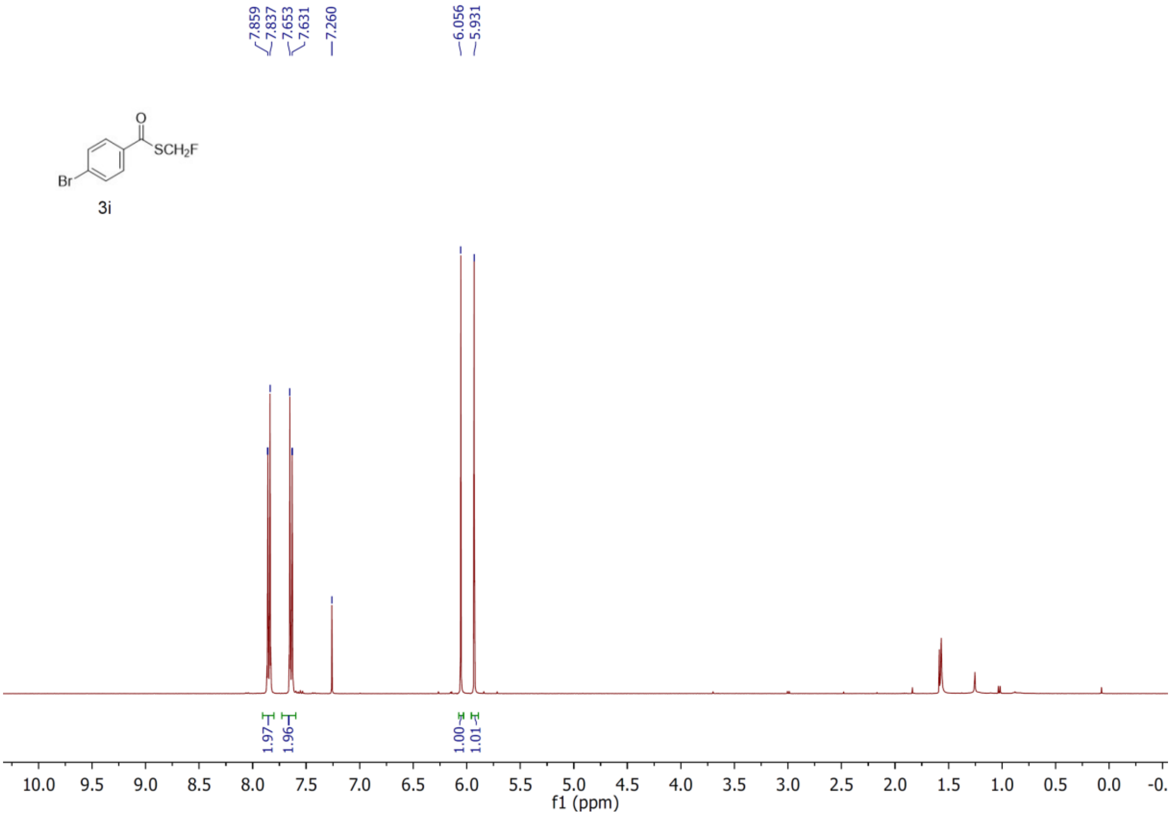

Supplementary Figure 41b | ^{13}C NMR (126 MHz, $CDCl_3$) of *S*-(fluoromethyl) 4-bromobenzothioate (**3i**)

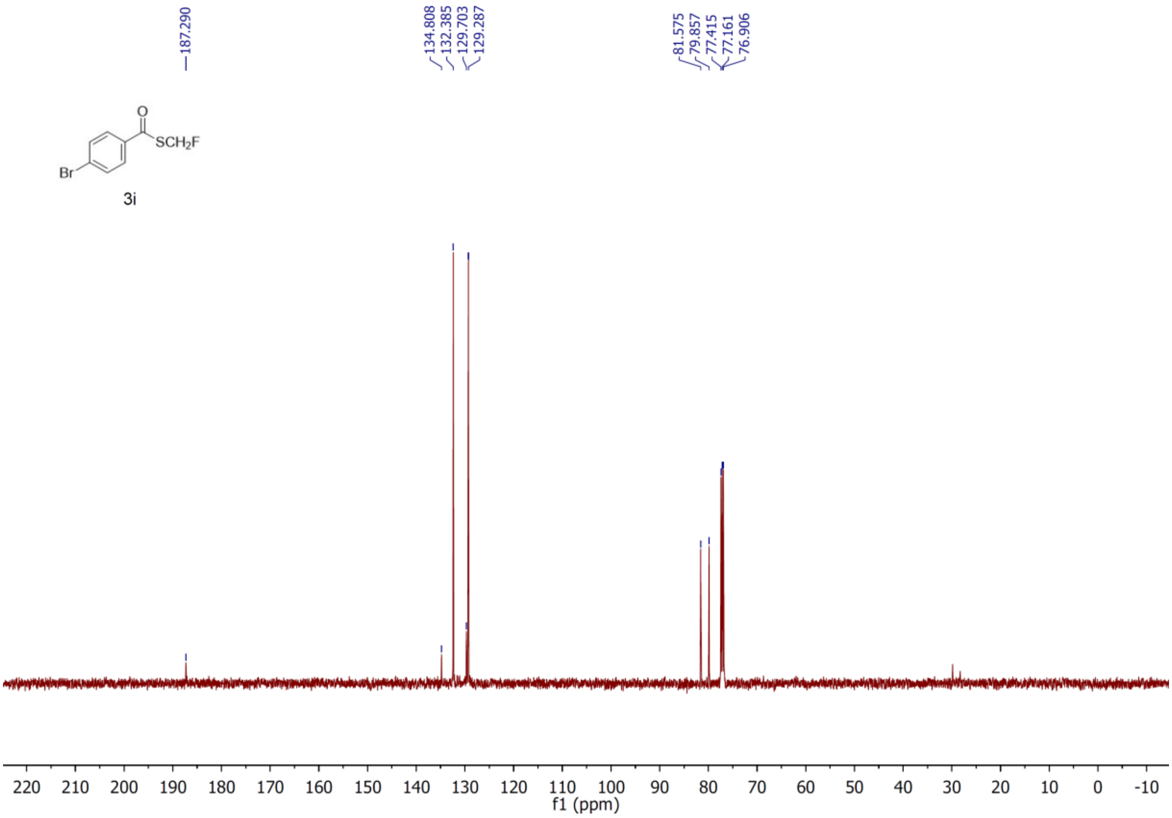

Supplementary Figure 41c | ^{19}F NMR (377 MHz, $CDCl_3$) of *S*-(fluoromethyl) 4-bromobenzothioate (3i)

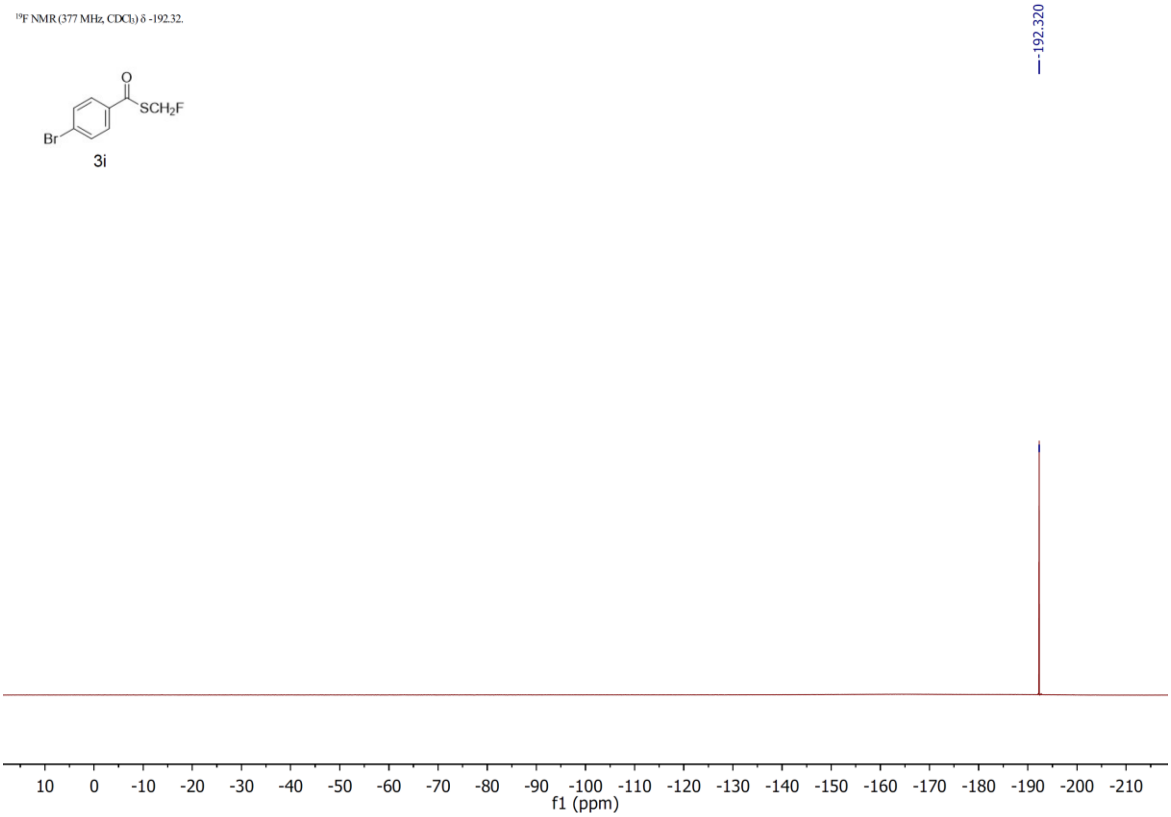

Supplementary Figure 42a | ^{1}H NMR (400 MHz, $CDCl_3$) of *S*-(fluoromethyl) 4-cyanobenzothioate (**3j**)

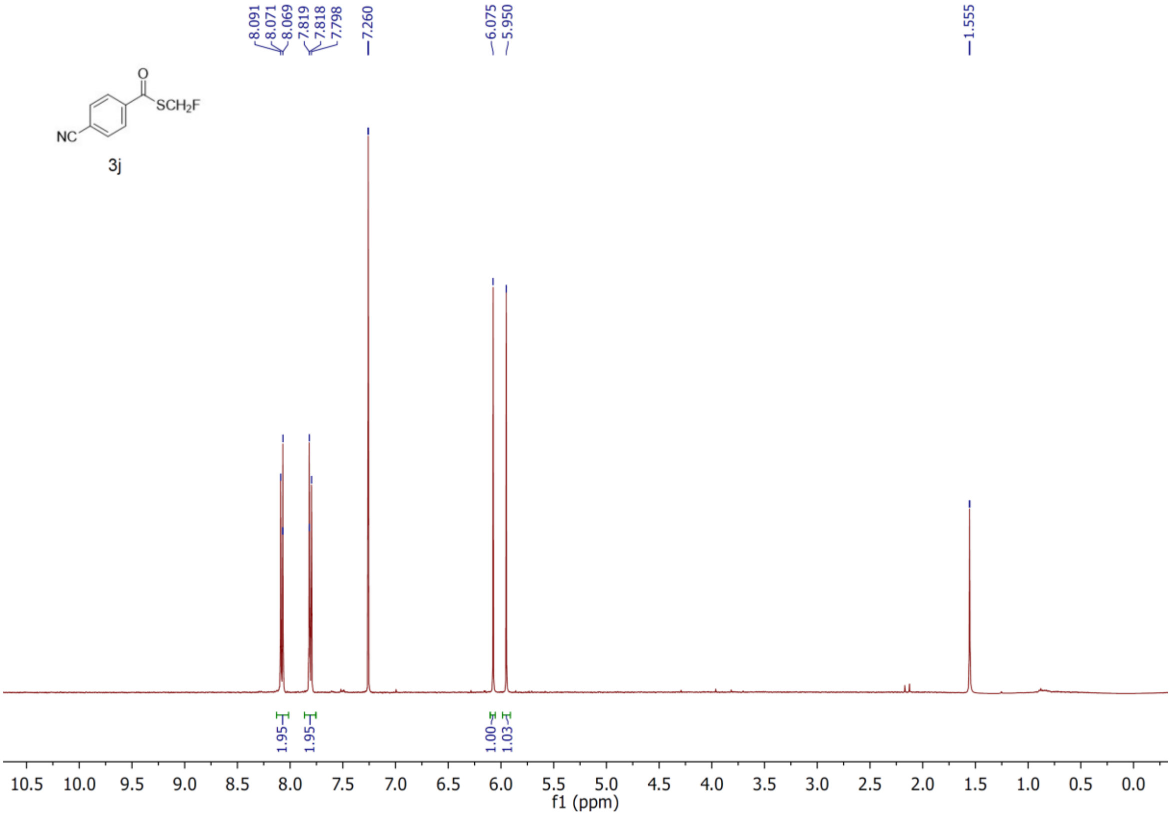

Supplementary Figure 42b | ^{13}C NMR (126 MHz, $CDCl_3$) of *S*-(fluoromethyl) 4-cyanobenzothioate (**3j**)

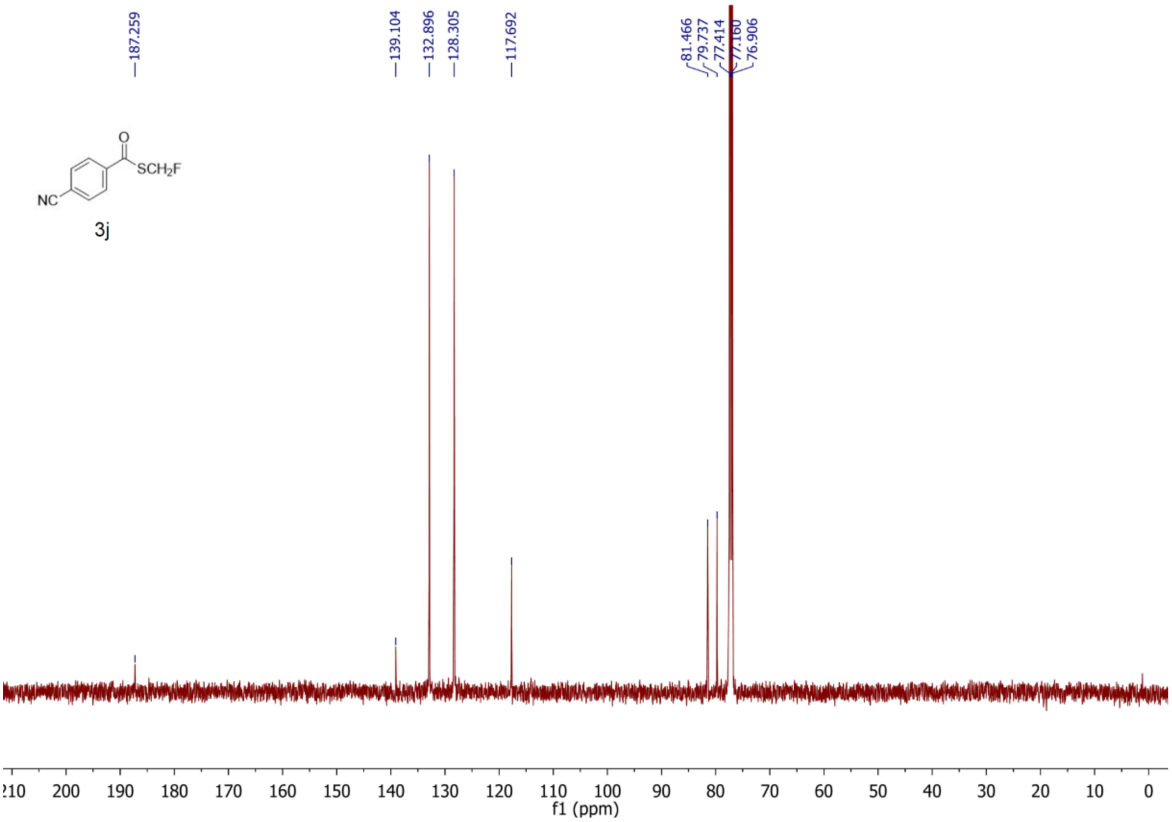

Supplementary Figure 42c | ^{19}F NMR (377 MHz, $CDCl_3$) of *S*-(fluoromethyl) 4-cyanobenzothioate (**3j**)

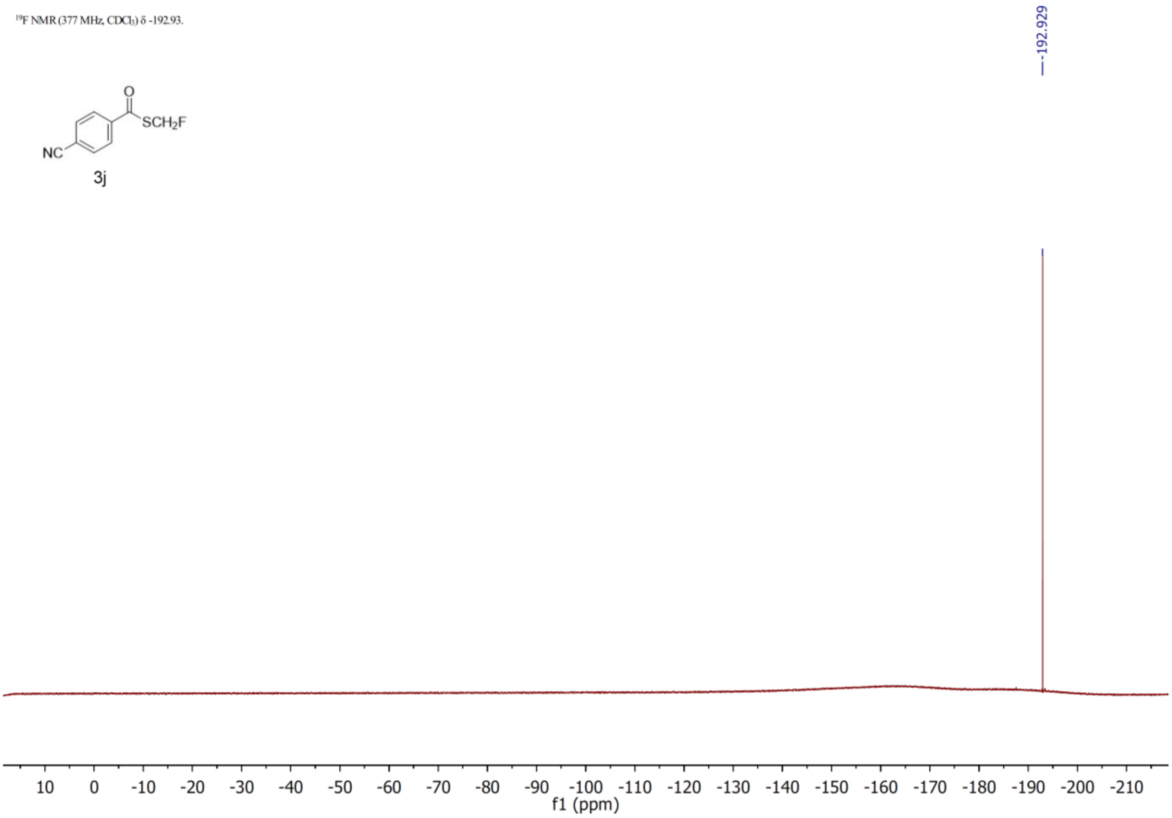

Supplementary Figure 43a | ^{1}H NMR (400 MHz, $CDCl_3$) of *S*-(fluoromethyl) 3-cyanobenzothioate (**3k**)

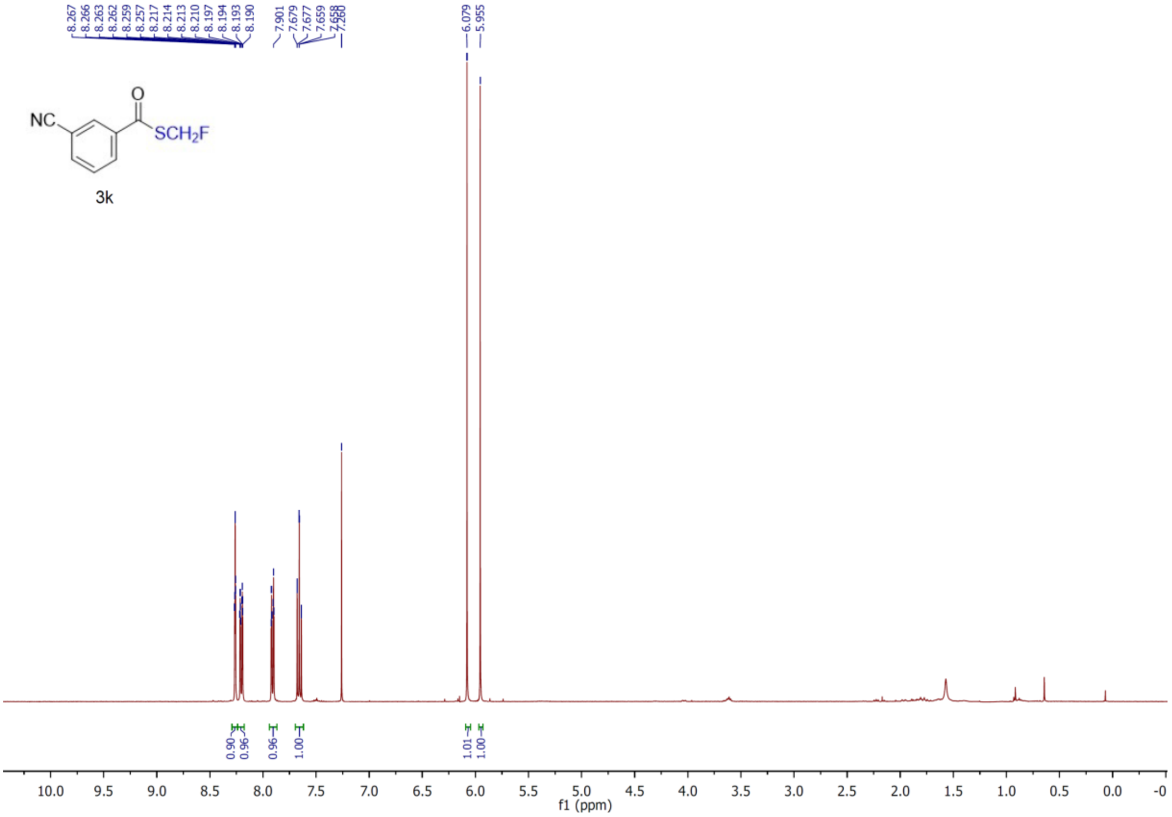

Supplementary Figure 43b | ^{13}C NMR (126 MHz, $CDCl_3$) of *S*-(fluoromethyl) 3-cyanobenzothioate (**3k**)

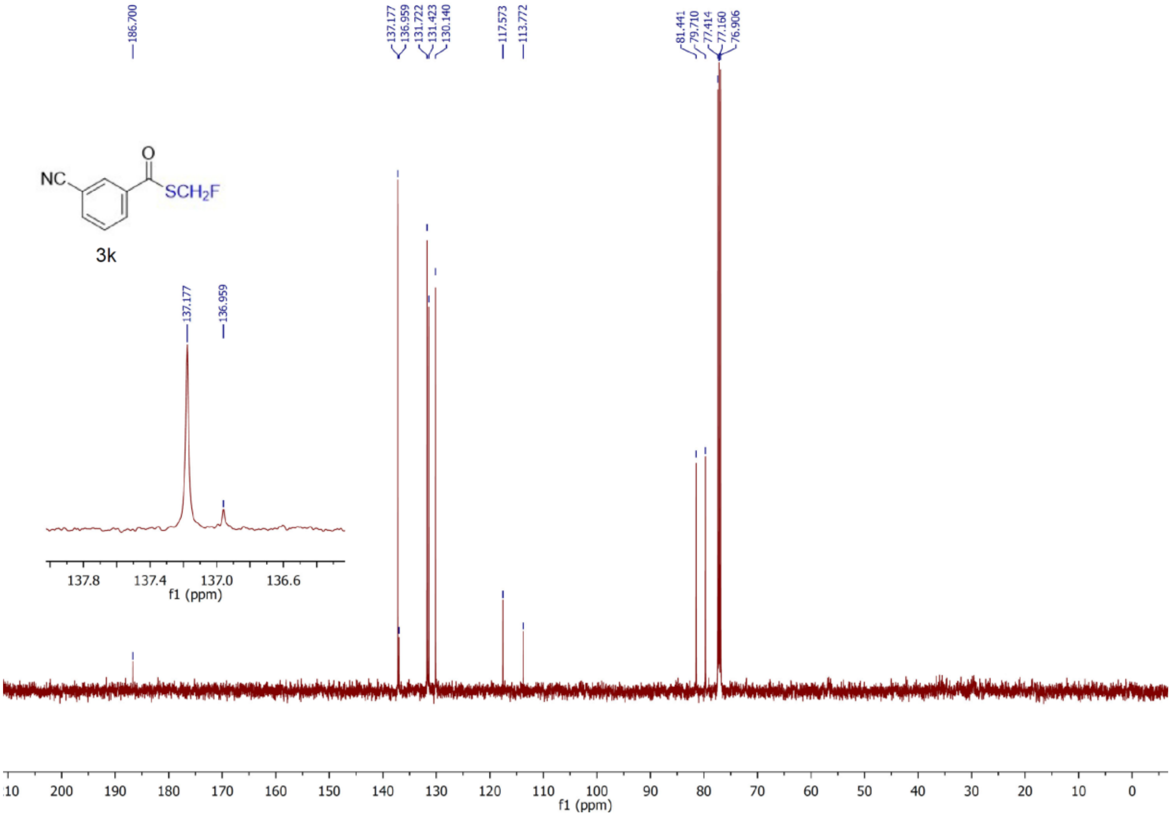

Supplementary Figure 43c | ^{19}F NMR (377 MHz, $CDCl_3$) of *S*-(fluoromethyl) 3-cyanobenzothioate (**3k**)

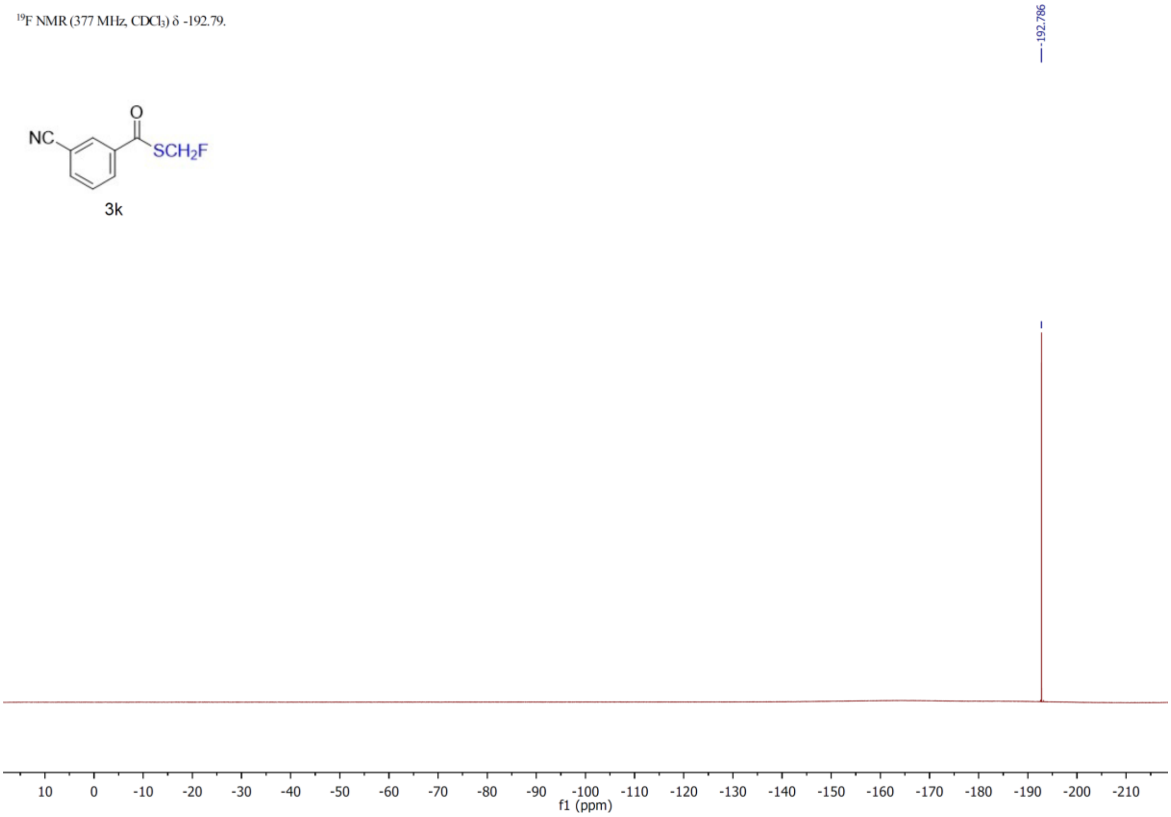

Supplementary Figure 44a | ^{1}H NMR (400 MHz, $CDCl_3$) of *S*-(fluoromethyl) naphthalene-2-carbothioate (**3l**)

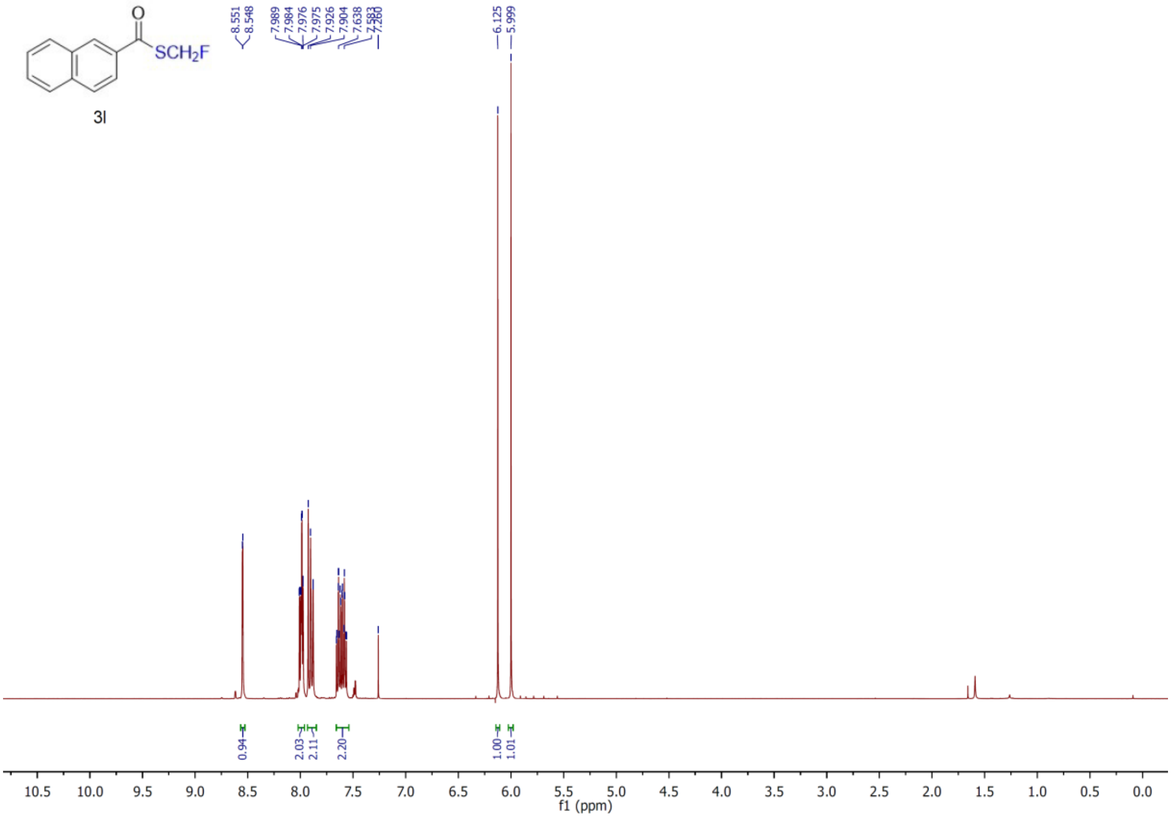

Supplementary Figure 44b | ^{13}C NMR (126 MHz, $CDCl_3$) of *S*-(fluoromethyl) naphthalene-2-carbothioate (**3l**)

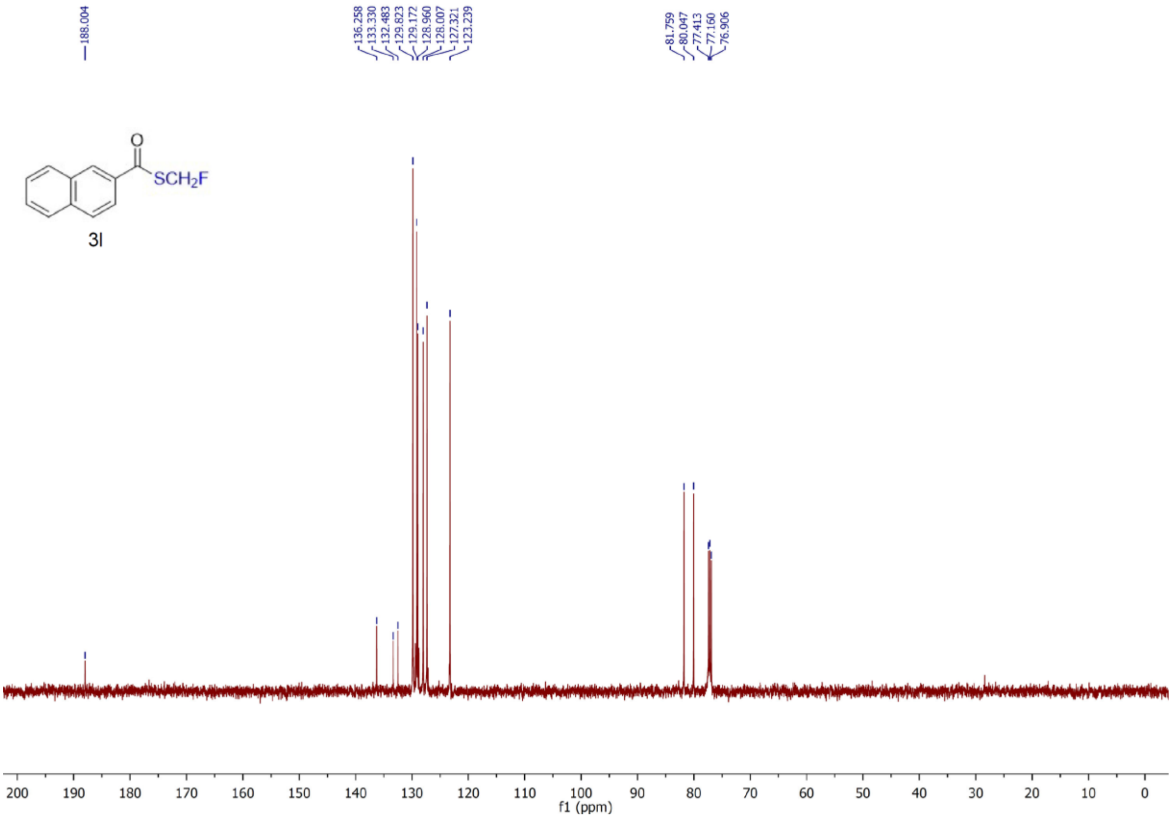

Supplementary Figure 44c | ^{19}F NMR (377 MHz, $CDCl_3$) of *S*-(fluoromethyl) naphthalene-2-carbothioate (**3l**)

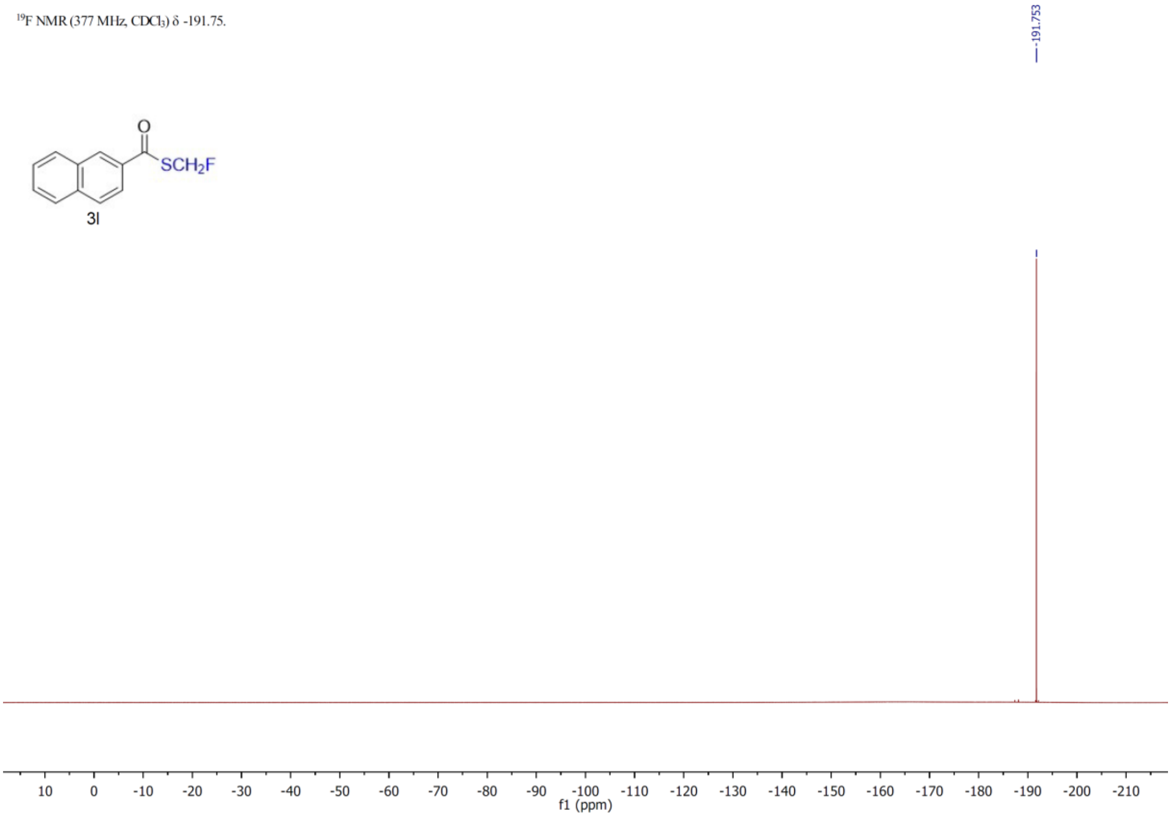

Supplementary Figure 45a | ^{1}H NMR (400 MHz, $CDCl_3$) of *S*-(fluoromethyl) 2,2-difluorobenzo[d][1,3]dioxole-5-carbothioate (**3m**)

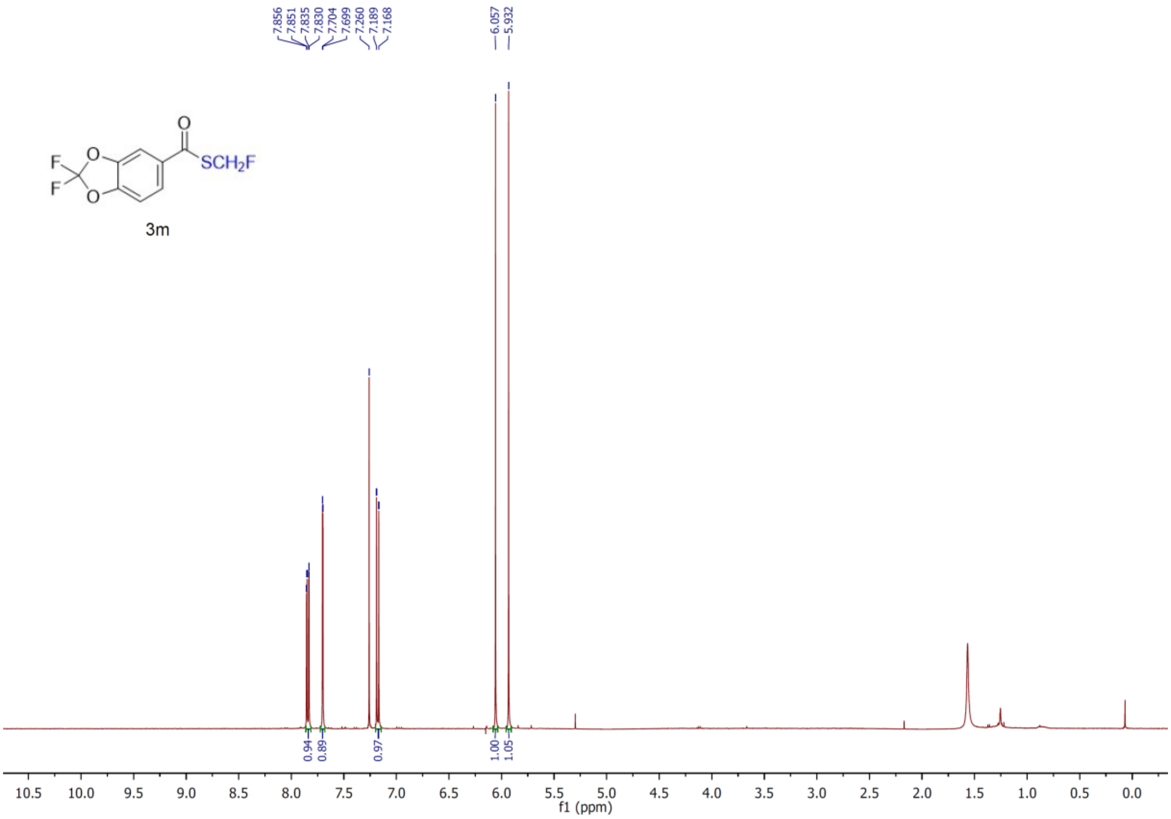

Supplementary Figure 45b | ^{13}C NMR (126 MHz, $CDCl_3$) of *S*-(fluoromethyl) 2,2-difluorobenzo[d][1,3]dioxole-5-carbothioate (**3m**)

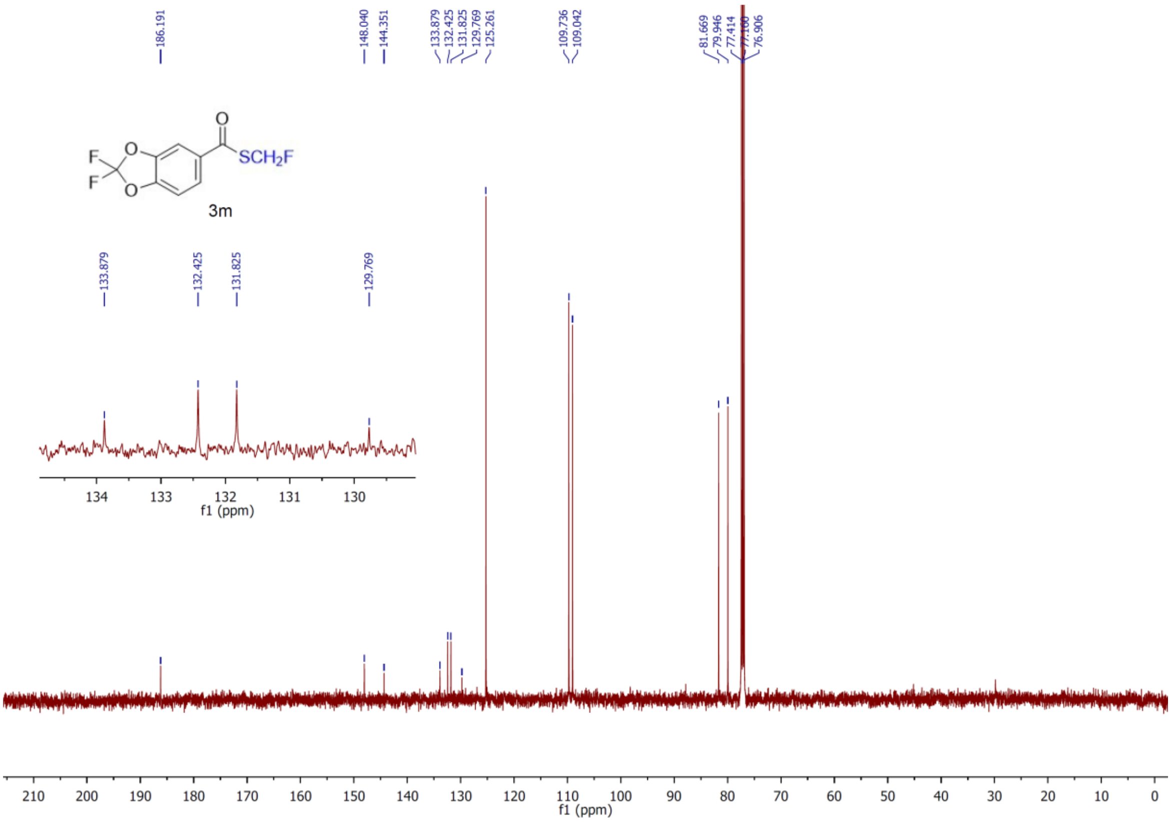

Supplementary Figure 45c | ^{19}F NMR (377 MHz, $CDCl_3$) of *S*-(fluoromethyl) 2,2-difluorobenzo[d][1,3]dioxole-5-carbothioate (**3m**)

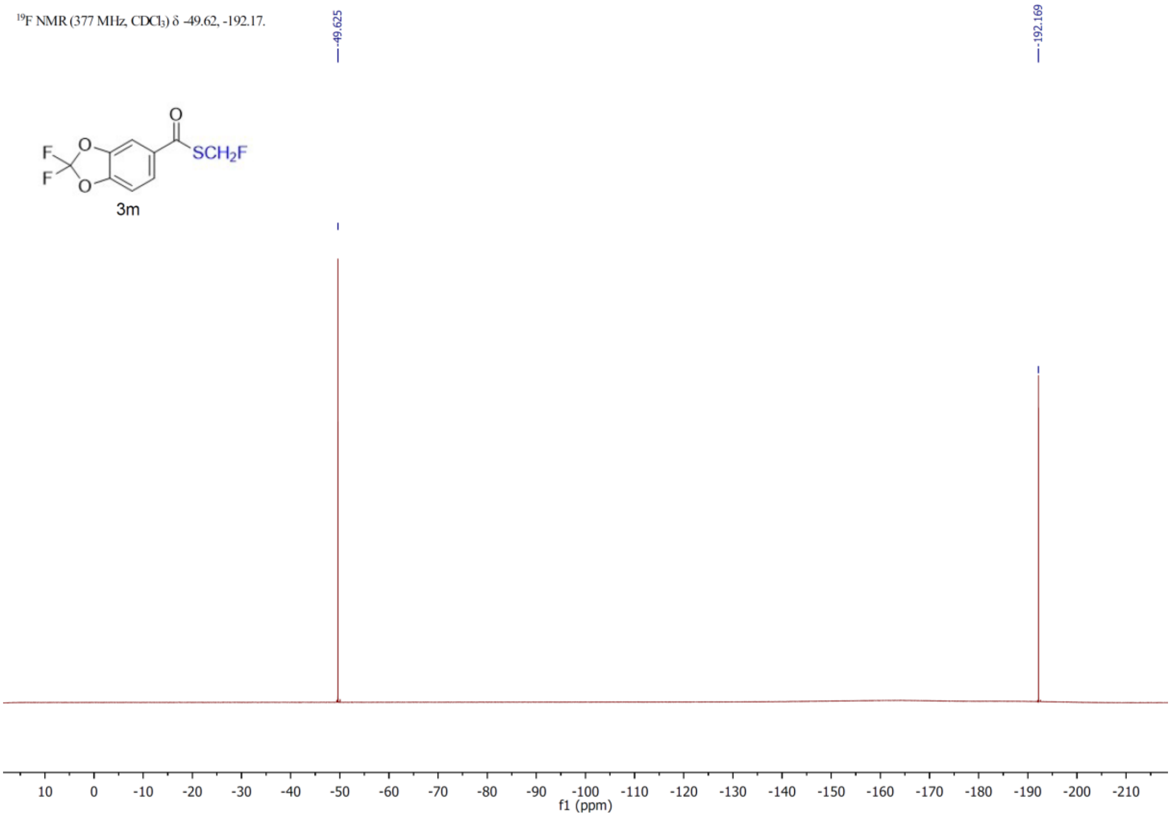

Supplementary Figure 46a | ^{1}H NMR (400 MHz, $CDCl_3$) of *S*-(fluoromethyl) benzo[b]thiophene-2-carbothioate (**3n**)

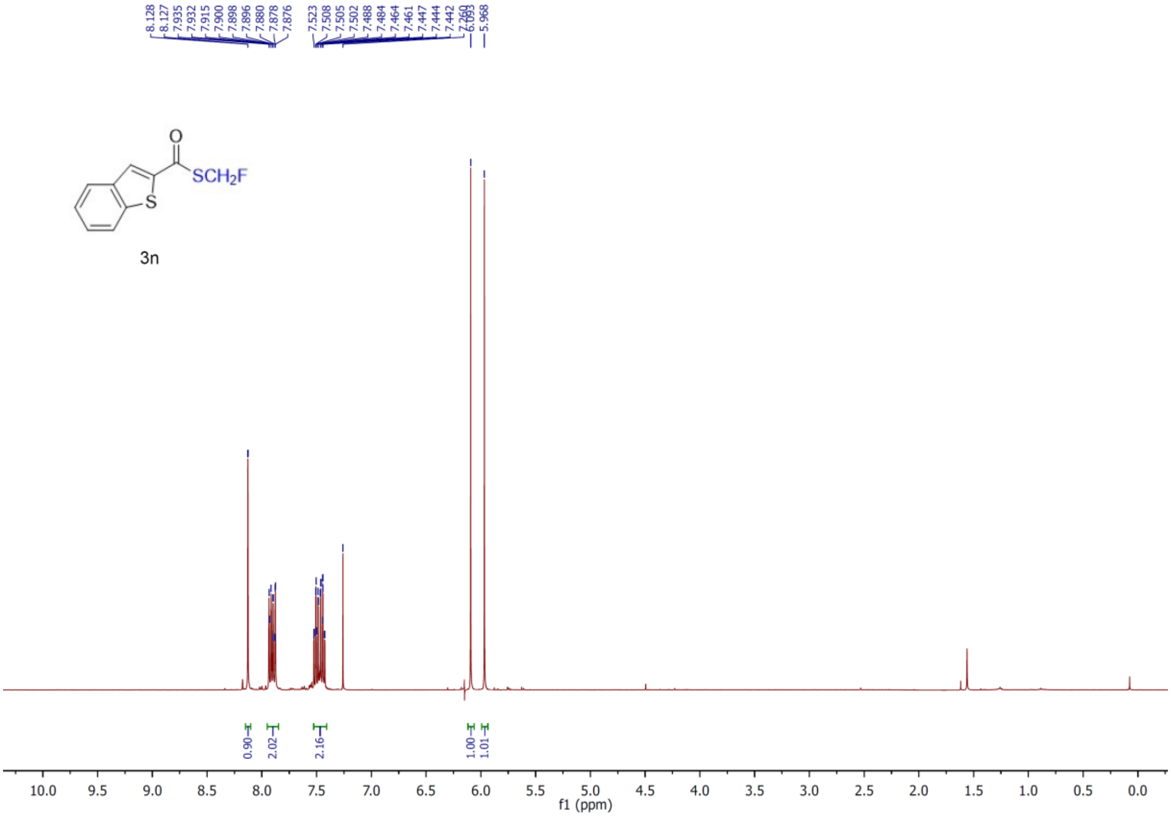

Supplementary Figure 46b | ^{13}C NMR (126 MHz, $CDCl_3$) of *S*-(fluoromethyl) benzo[b]thiophene-2-carbothioate (**3n**)

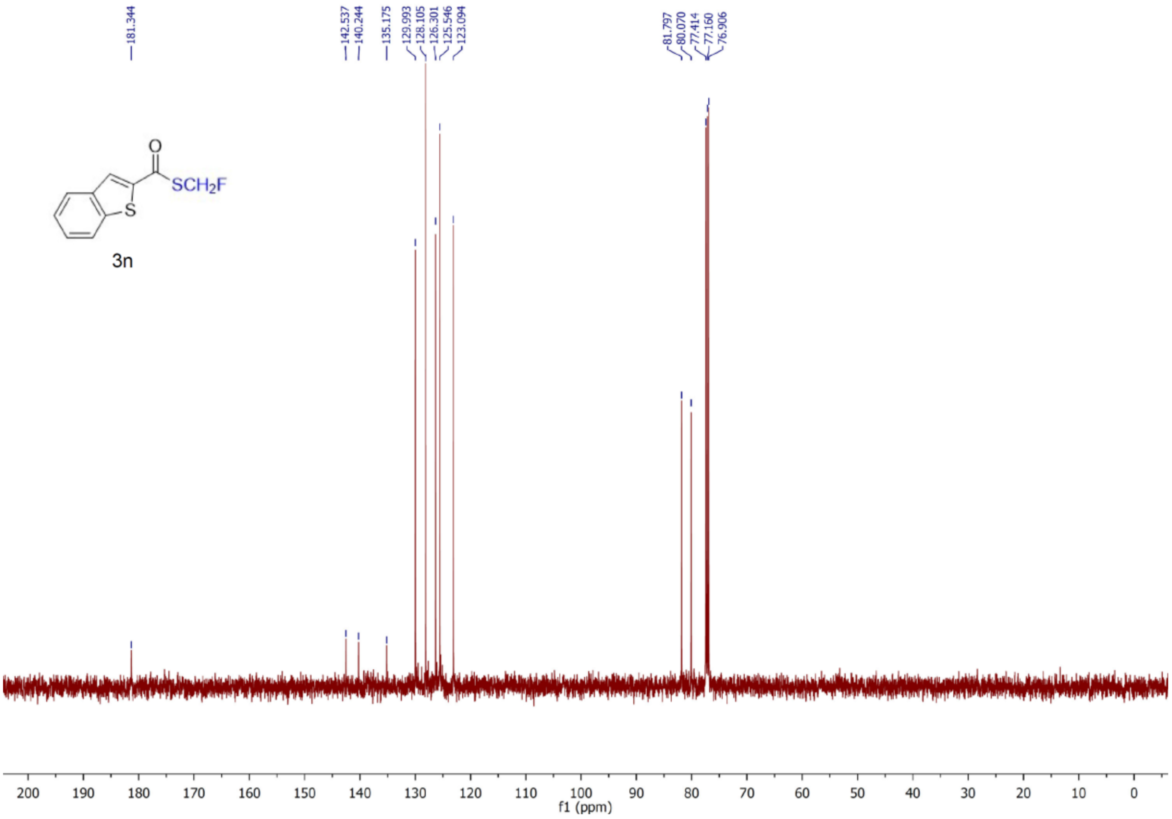

Supplementary Figure 46c | ^{19}F NMR (377 MHz, $CDCl_3$) of *S*-(fluoromethyl) benzo[b]thiophene-2-carbothioate (**3n**)

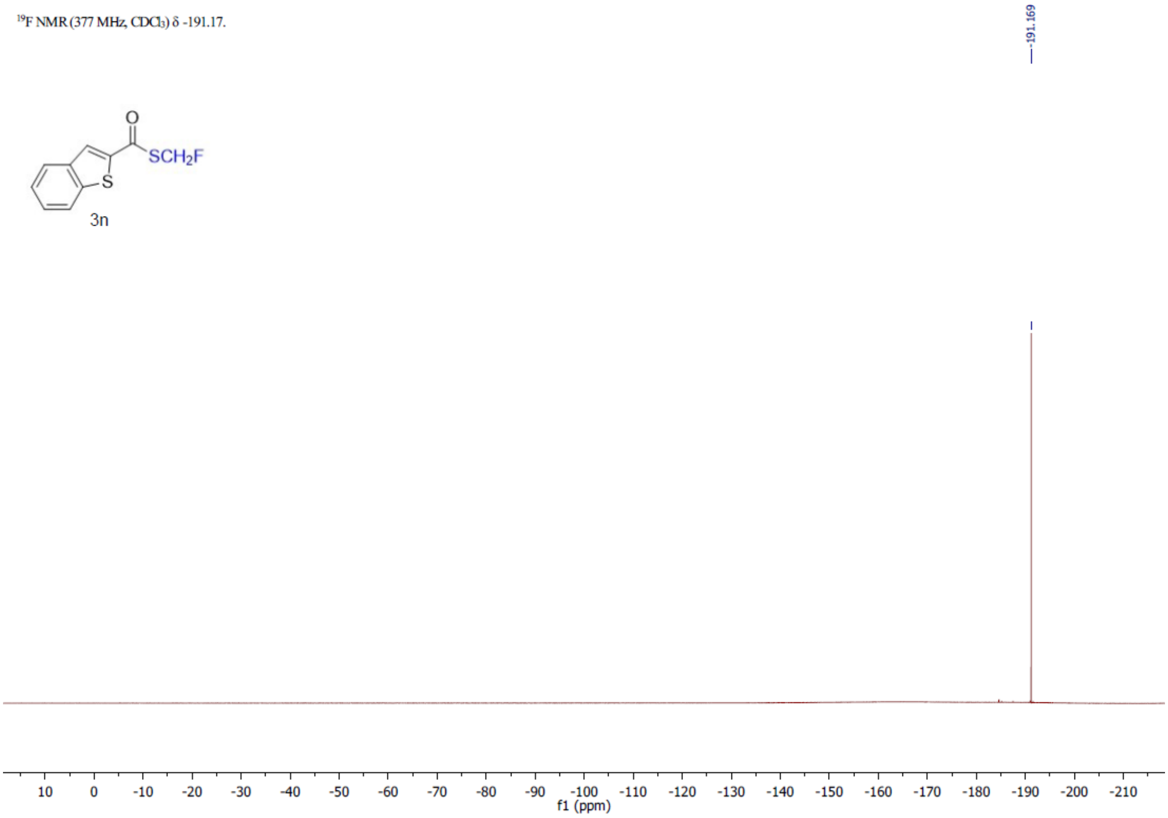

Supplementary Figure 47a | ^{1}H NMR (400 MHz, $CDCl_3$) of *S*-(fluoromethyl) dodecanethioate (**3o**)

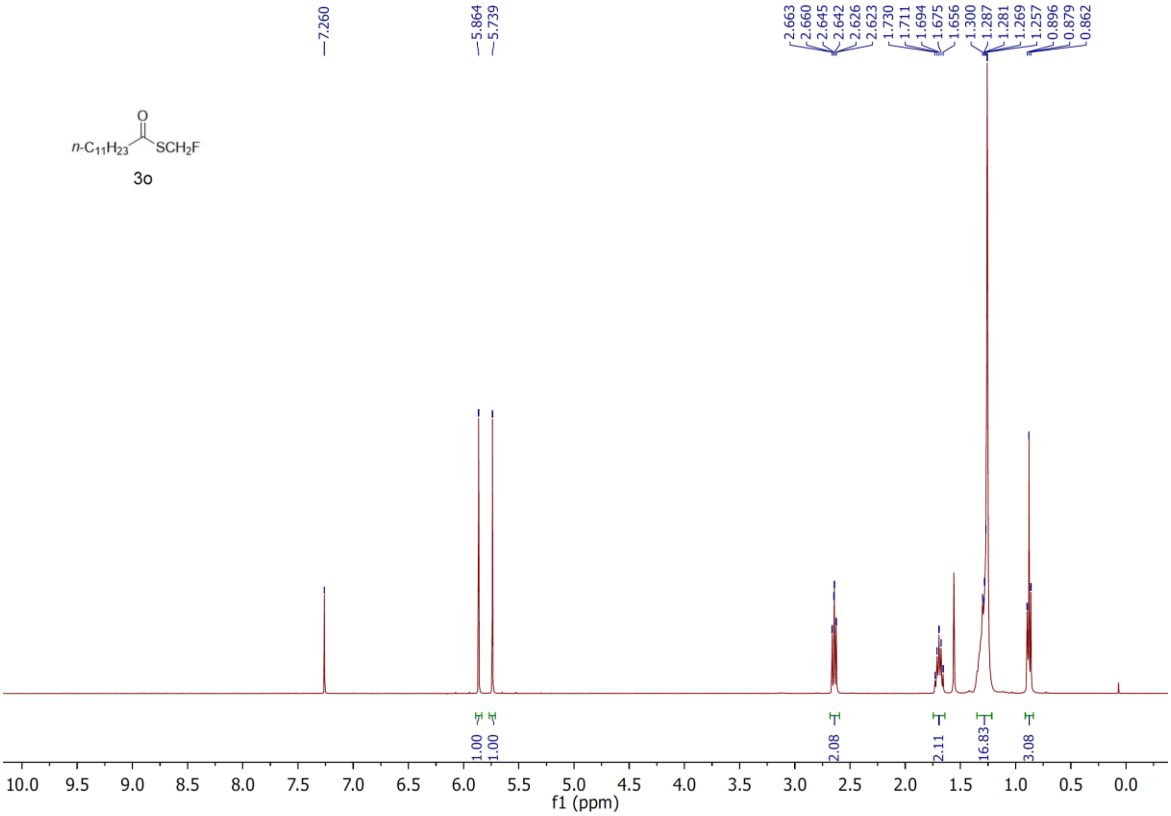

Supplementary Figure 47b | ^{13}C NMR (126 MHz, $CDCl_3$) of *S*-(fluoromethyl) dodecanethioate (**3o**)

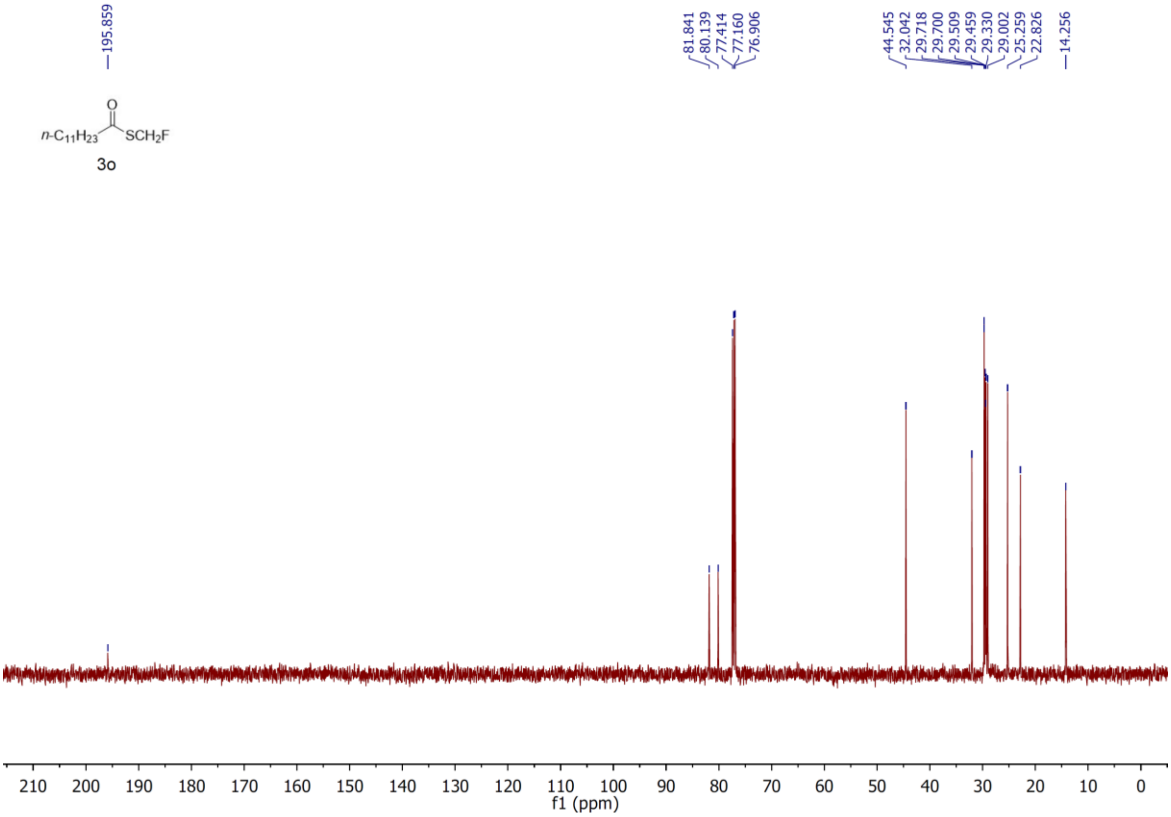

Supplementary Figure 47c | ^{19}F NMR (377 MHz, $CDCl_3$) of *S*-(fluoromethyl) dodecanethioate (**3o**)

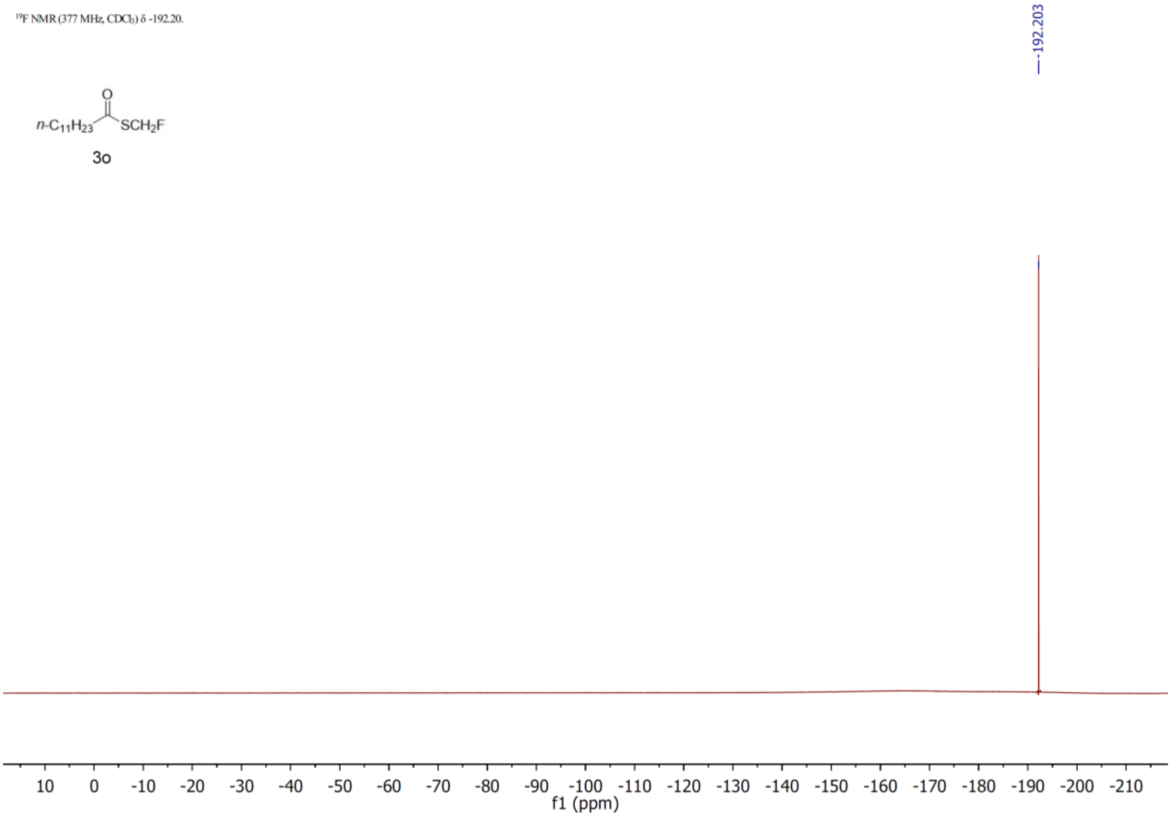

Supplementary Figure 48a | ^{1}H NMR (400 MHz, $CDCl_3$) of *S*-(fluoromethyl) 3-phenylpropanethioate (**3p**)

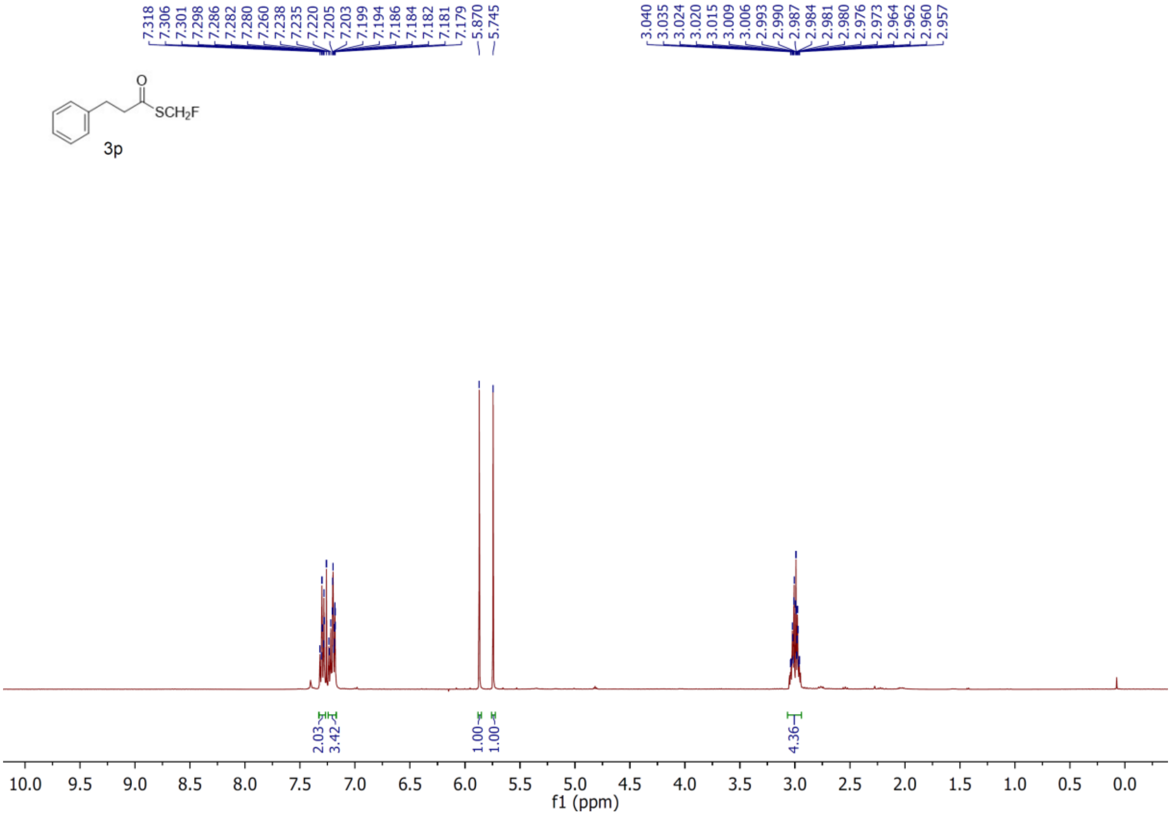

Supplementary Figure 48b | ^{13}C NMR (126 MHz, $CDCl_3$) of *S*-(fluoromethyl) 3-phenylpropanethioate (**3p**)

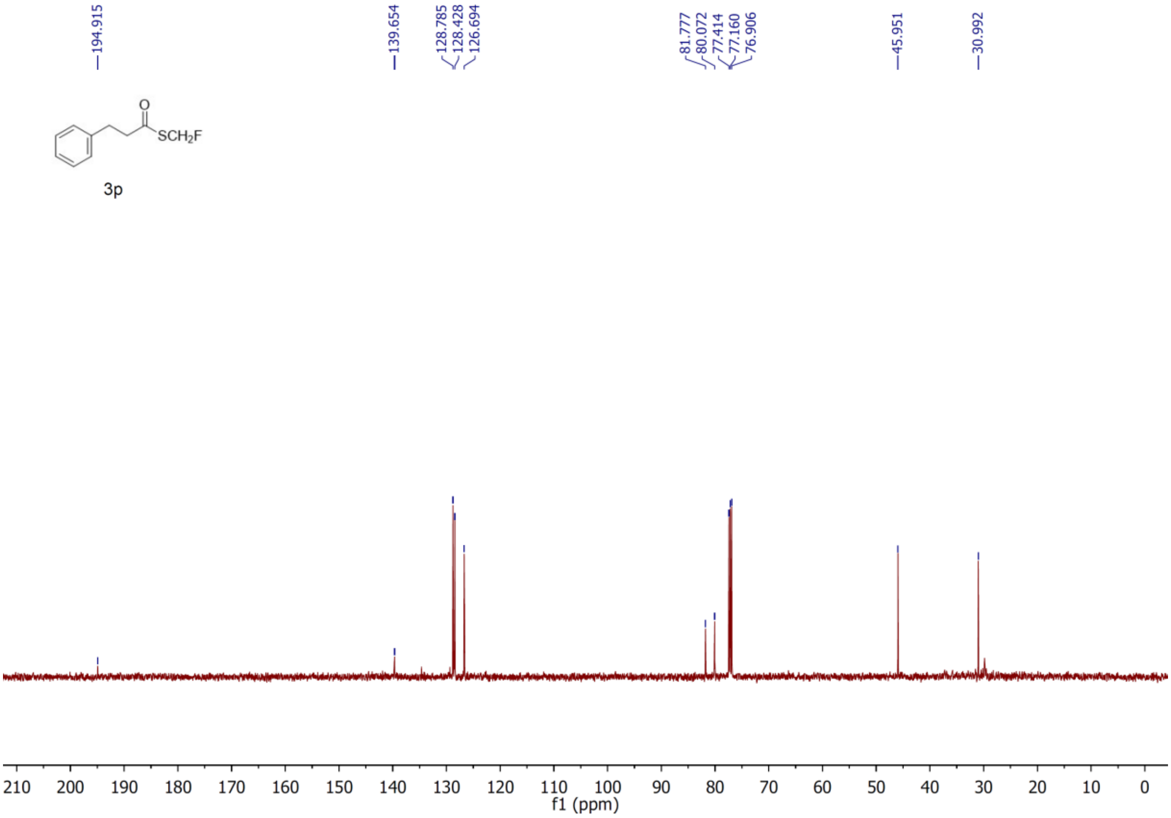

Supplementary Figure 48c | ^{19}F NMR (377 MHz, $CDCl_3$) of *S*-(fluoromethyl) 3-phenylpropanethioate (**3p**)

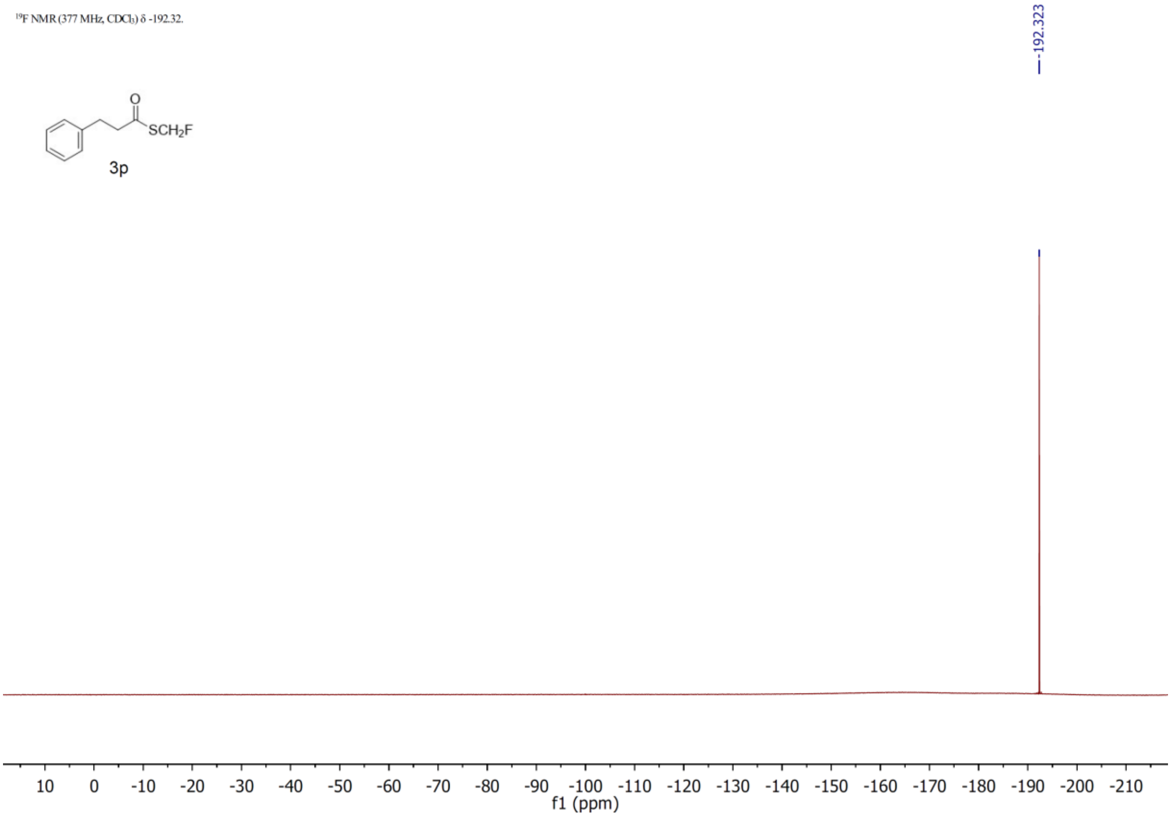

Supplementary Figure 49a | ^{1}H NMR (400 MHz, $CDCl_3$) of *S*-(fluoromethyl) 4-(1,3-dioxoisoindolin-2-yl)butanethioate (**3q**)

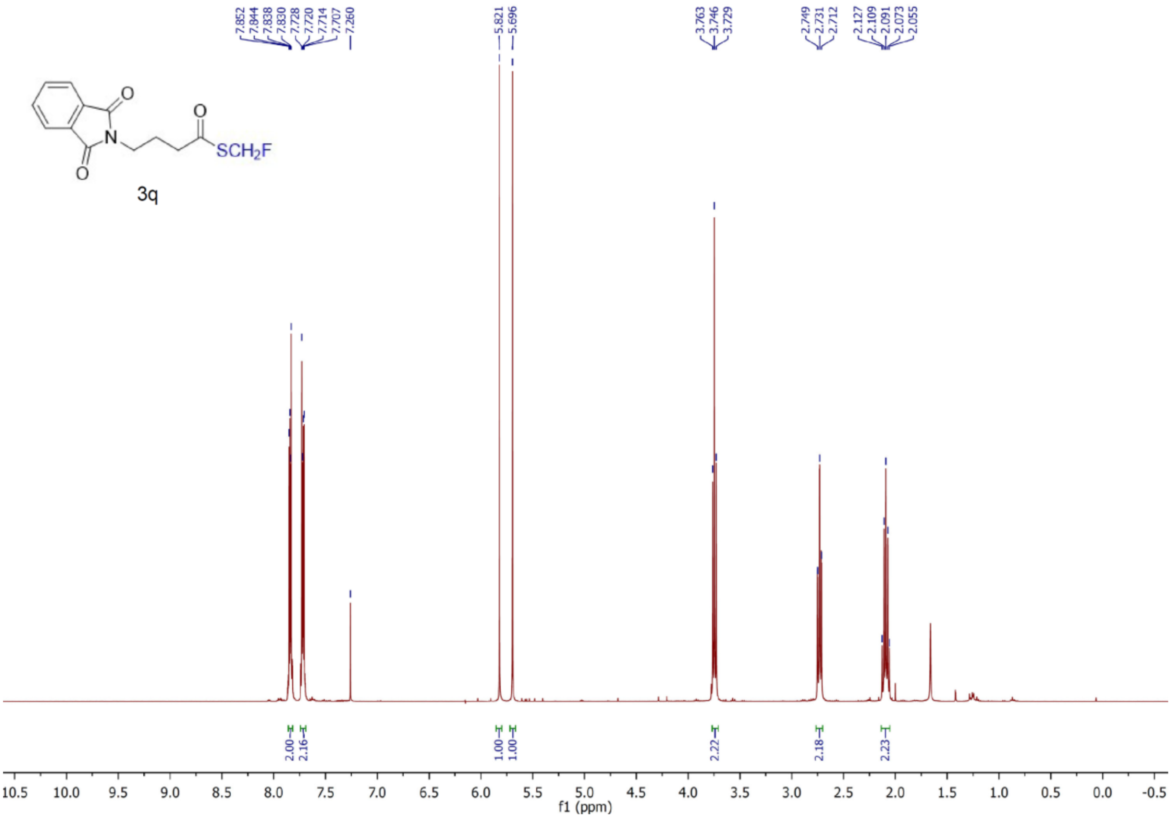

Supplementary Figure 49b | ^{13}C NMR (126 MHz, $CDCl_3$) of *S*-(fluoromethyl) 4-(1,3-dioxoisoindolin-2-yl)butanethioate (**3q**)

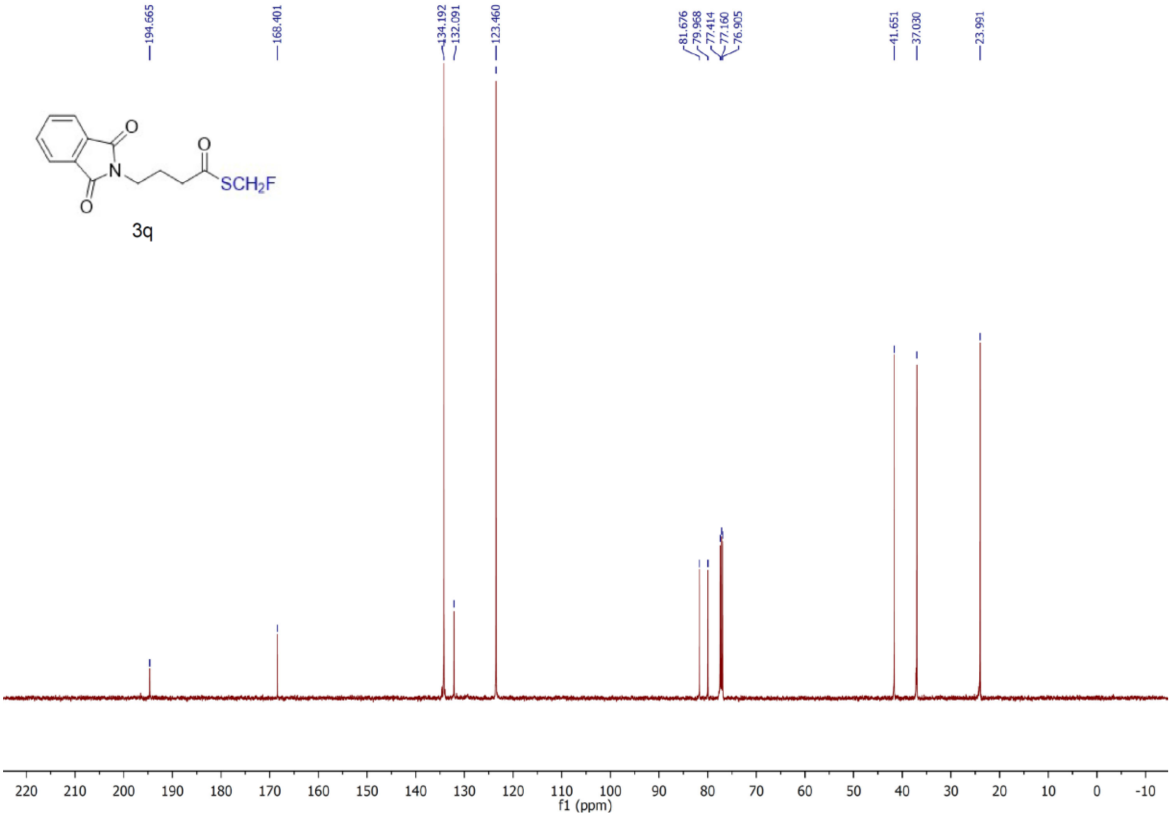

Supplementary Figure 49c | ^{19}F NMR (377 MHz, $CDCl_3$) of *S*-(fluoromethyl) 4-(1,3-dioxoisoindolin-2-yl)butanethioate (**3q**)

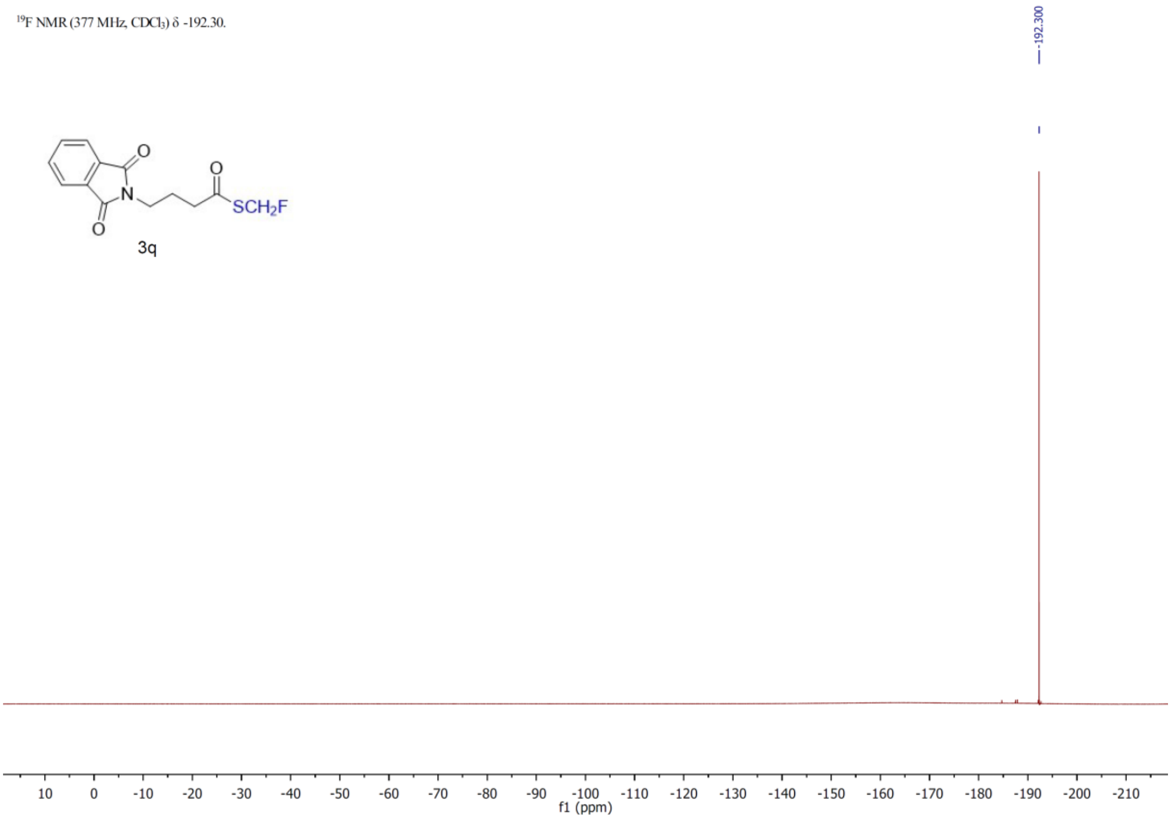

Supplementary Figure 50a | ^{1}H NMR (400 MHz, $CDCl_3$) of *S*-(fluoromethyl) undec-10-enethioate (**3r**)

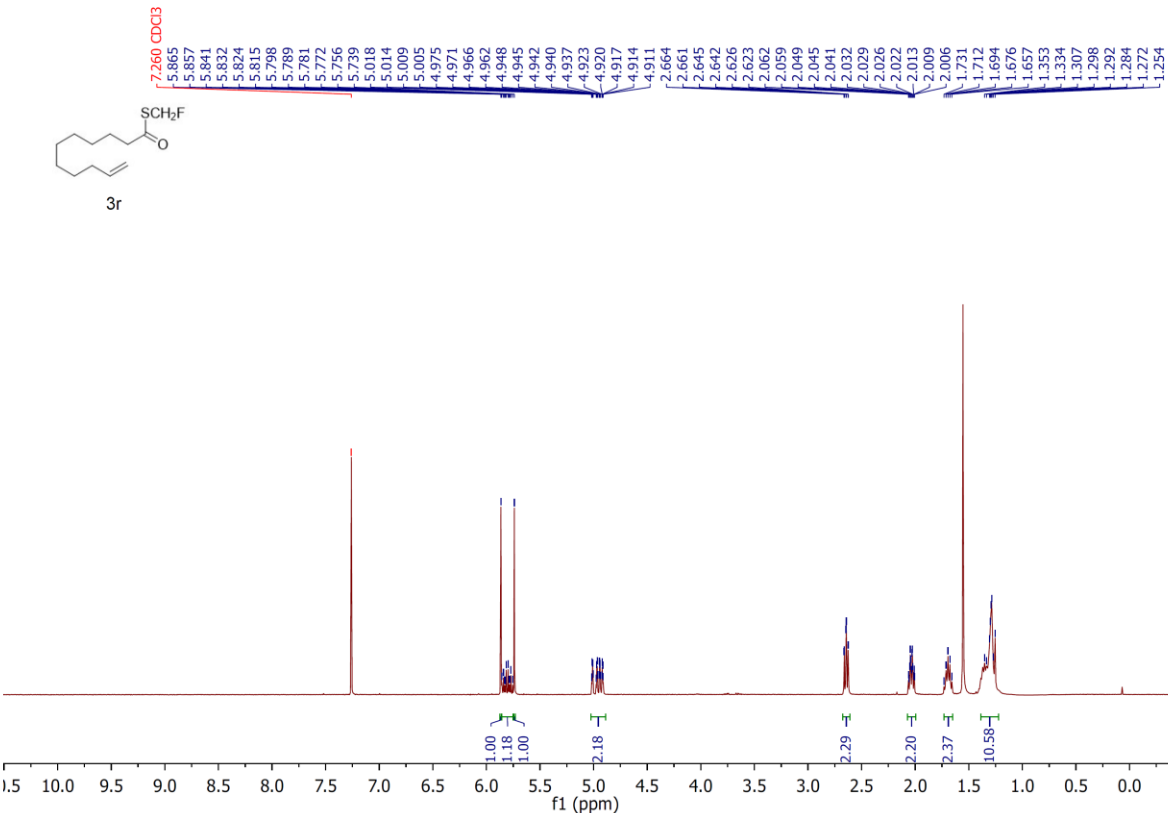

Supplementary Figure 50b | ^{13}C NMR (126 MHz, $CDCl_3$) of *S*-(fluoromethyl) undec-10-enethioate (**3r**)

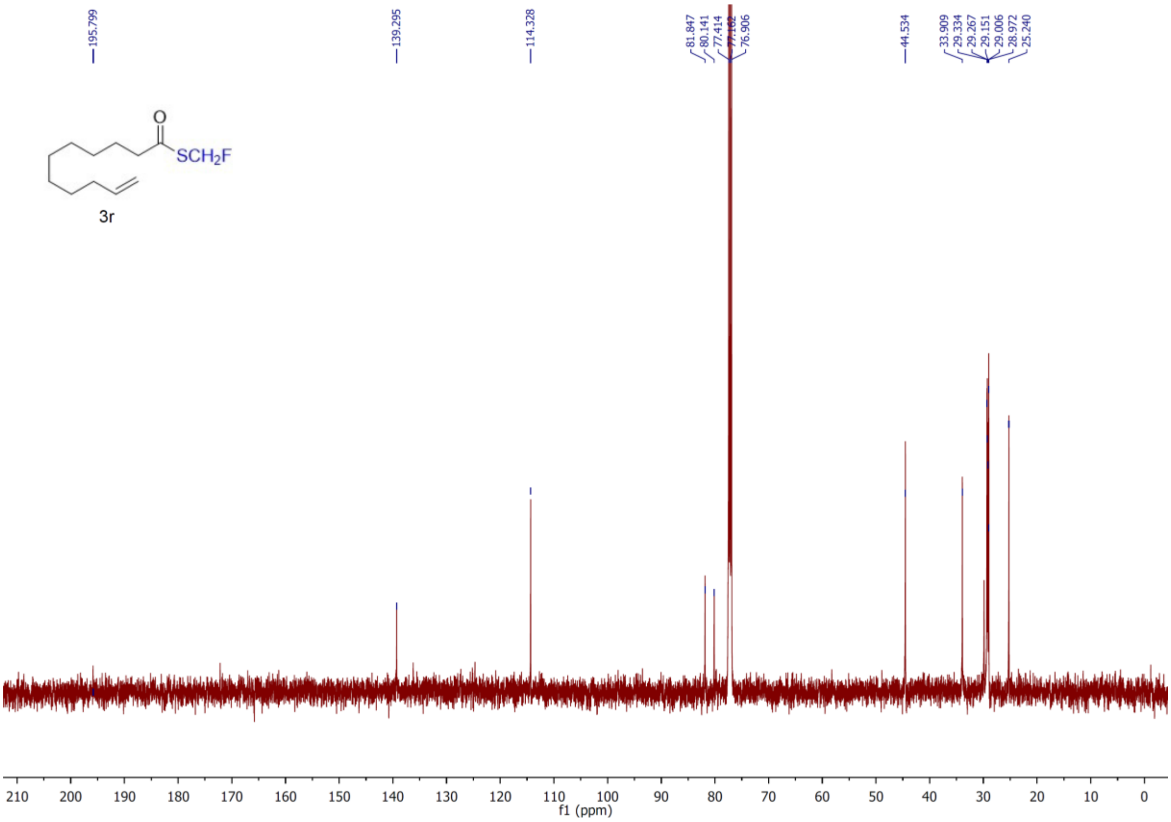

Supplementary Figure 50c | ^{19}F NMR (377 MHz, $CDCl_3$) of *S*-(fluoromethyl) undec-10-enethioate (**3r**)

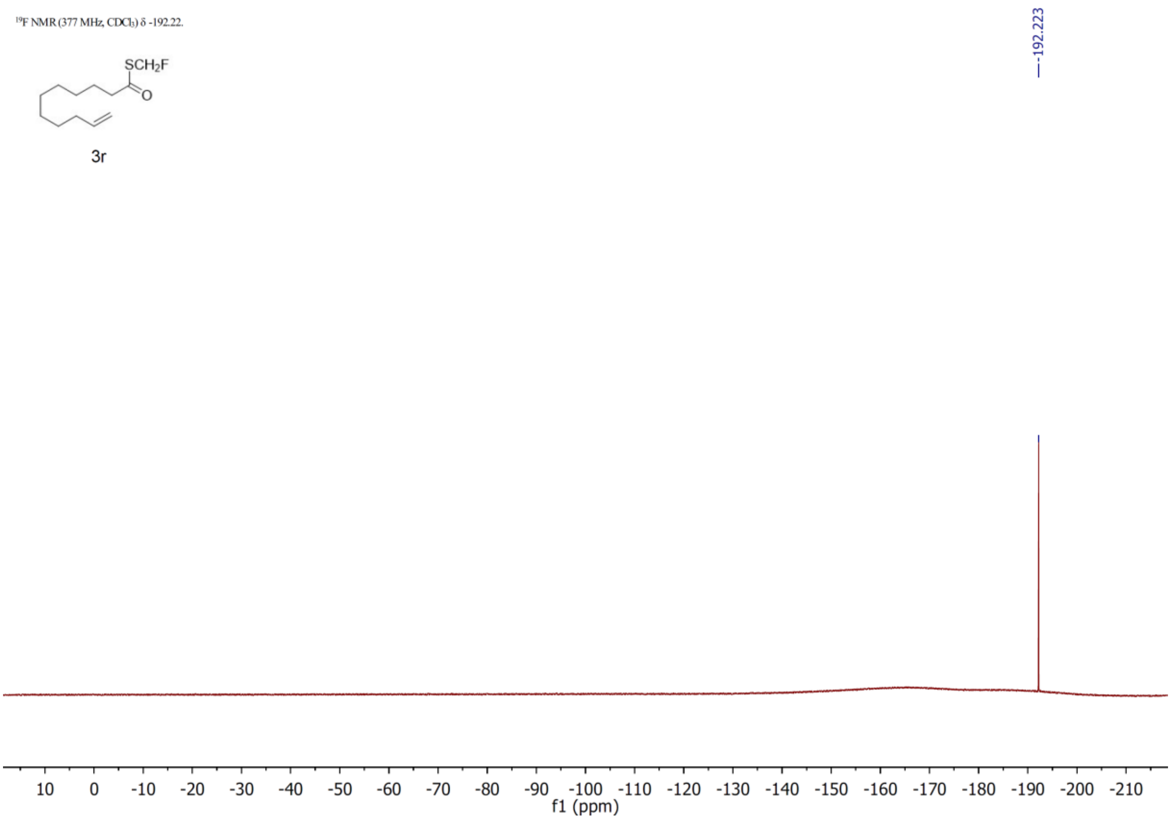

Supplementary Figure 51a | ^{1}H NMR (400 MHz, $CDCl_3$) of *S*-(fluoromethyl) 3-(4-(tert-butyl)phenyl)-2-methylpropanethioate (**3s**)

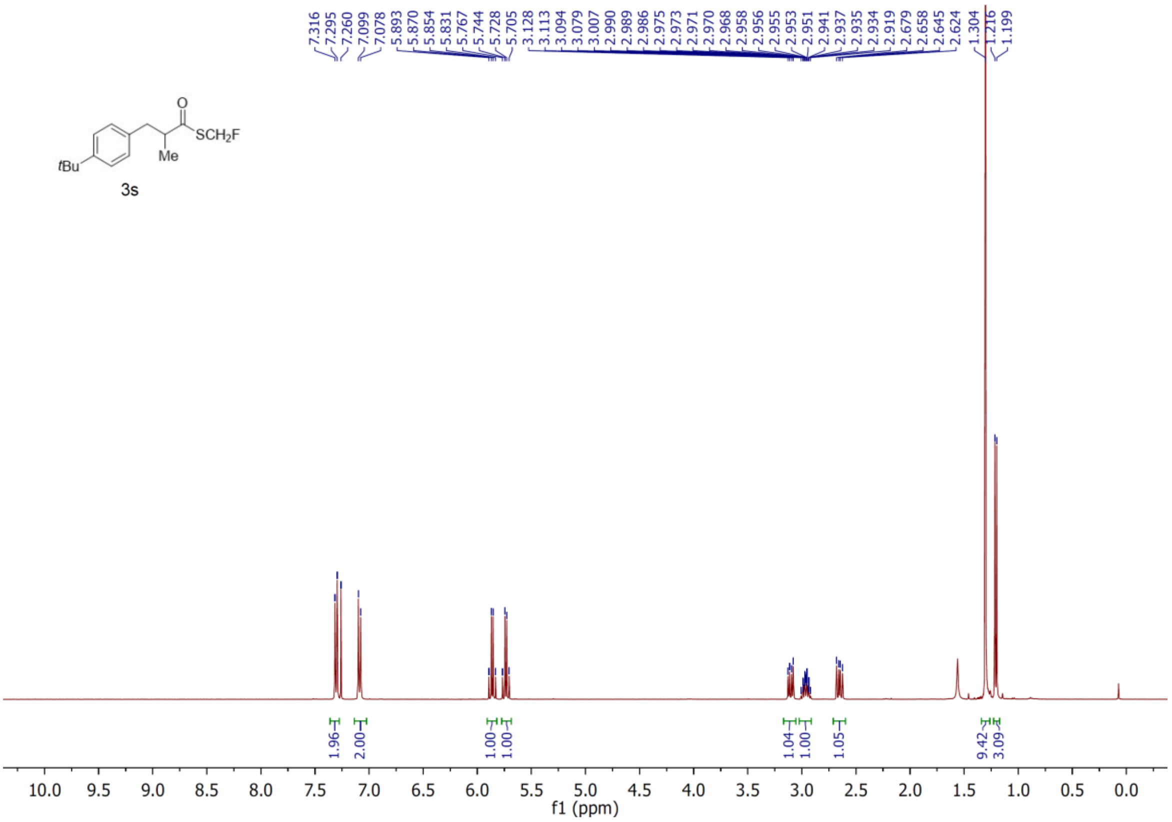

Supplementary Figure 51b | ^{13}C NMR (126 MHz, $CDCl_3$) of *S*-(fluoromethyl) 3-(4-(tert-butyl)phenyl)-2-methylpropanethioate (**3s**)

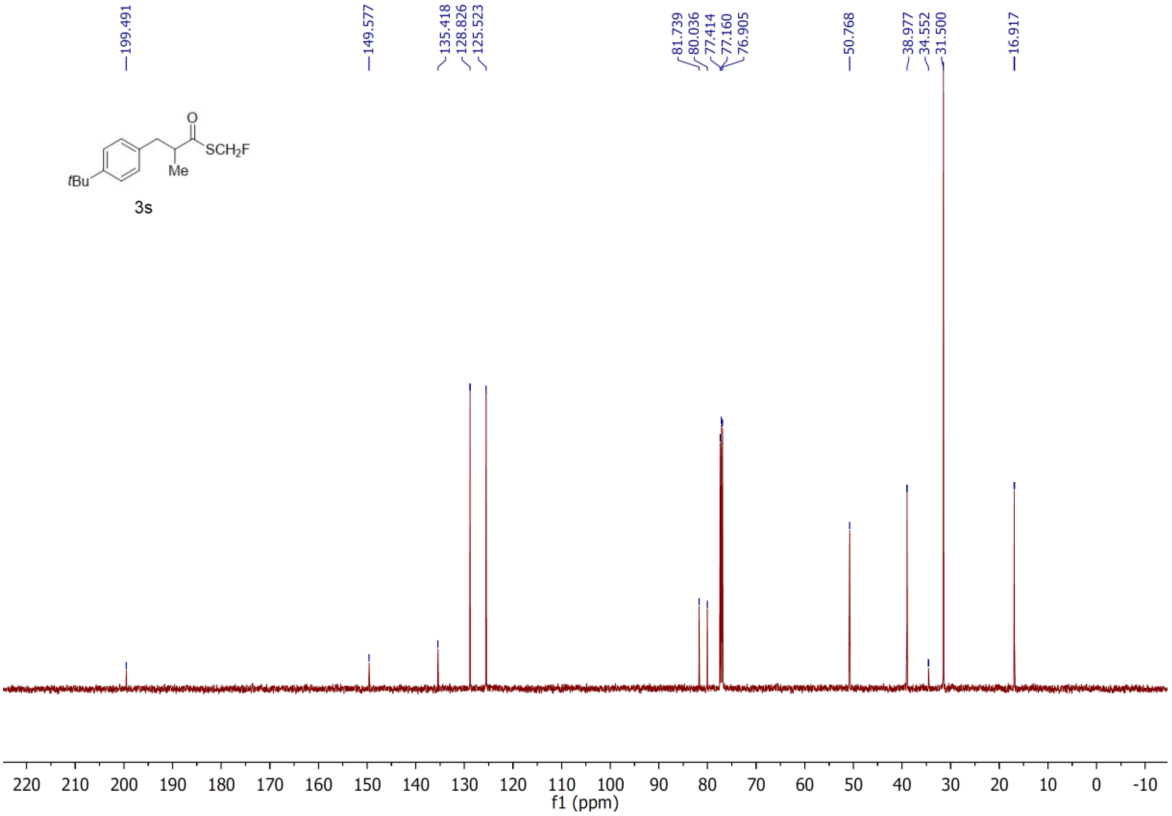

Supplementary Figure 51c | ^{19}F NMR (377 MHz, $CDCl_3$) of *S*-(fluoromethyl) 3-(4-(tert-butyl)phenyl)-2-methylpropanethioate (**3s**)

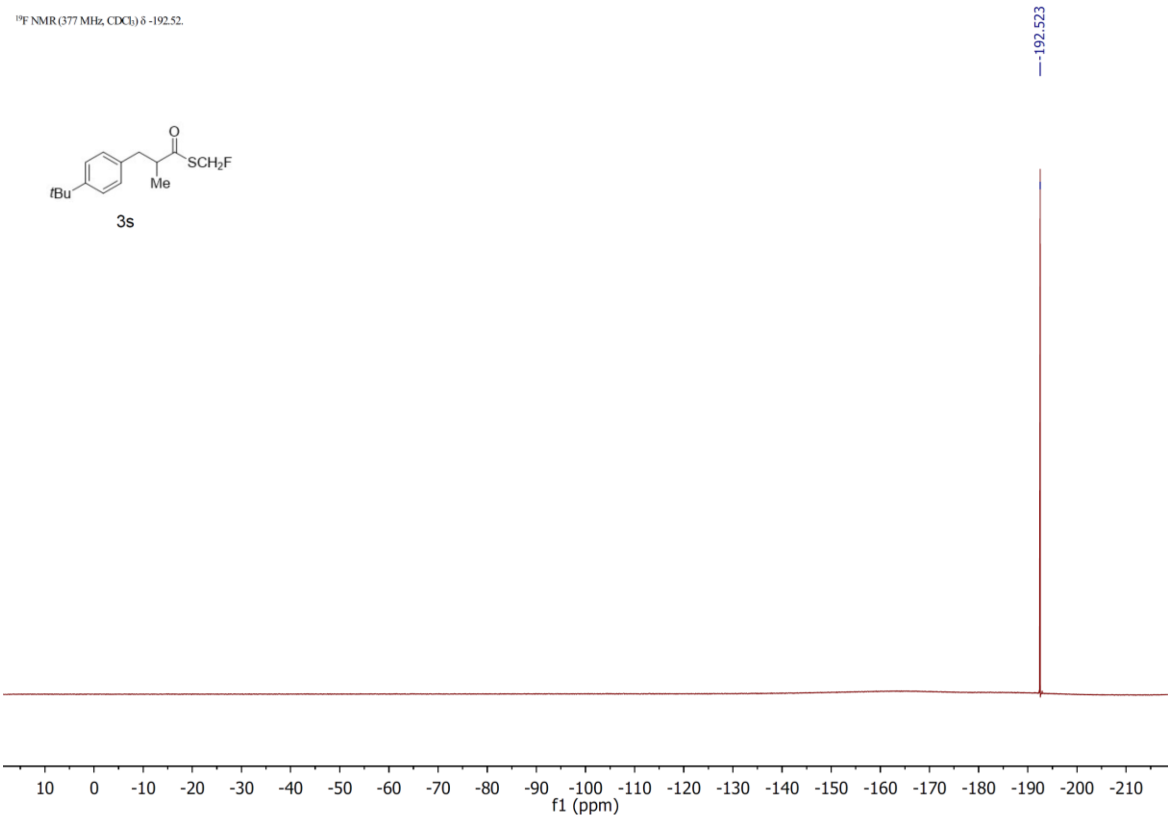

Supplementary Figure 52a | ^{1}H NMR (400 MHz, $CDCl_3$) of *tert*-Butyl 4-(((fluoromethyl)thio)carbonyl)piperidine-1-carboxylate (**3t**)

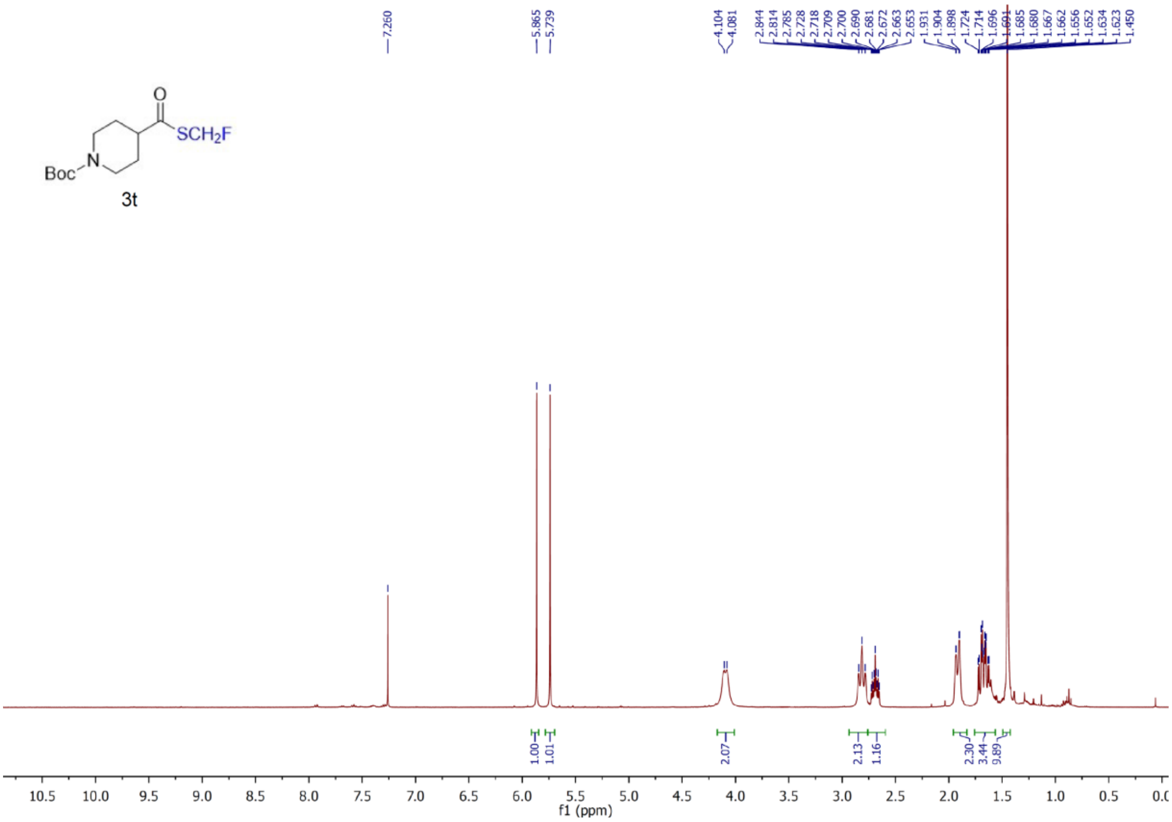

Supplementary Figure 52b | ^{13}C NMR (126 MHz, $CDCl_3$) of *tert*-Butyl 4-(((fluoromethyl)thio)carbonyl)piperidine-1-carboxylate (**3t**)

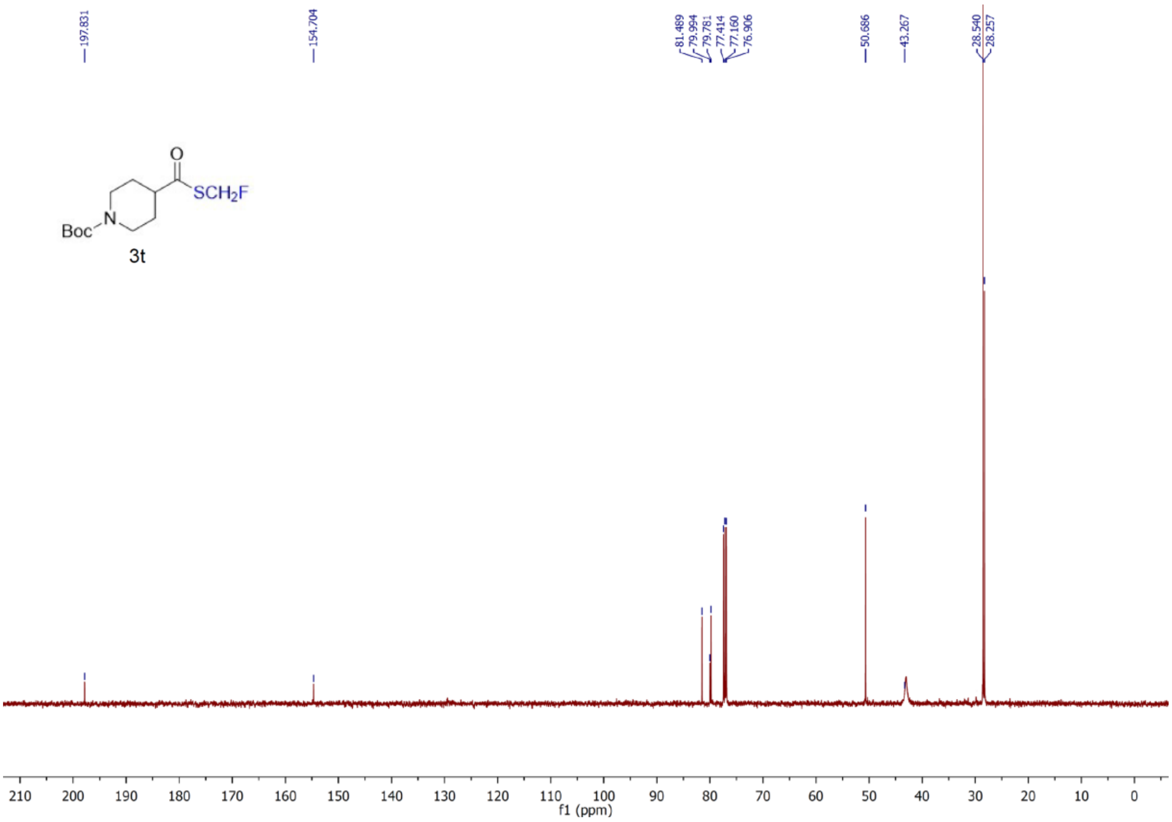

Supplementary Figure 52c | ^{19}F NMR (377 MHz, $CDCl_3$) of *tert*-Butyl 4-(((fluoromethyl)thio)carbonyl)piperidine-1-carboxylate (**3t**)

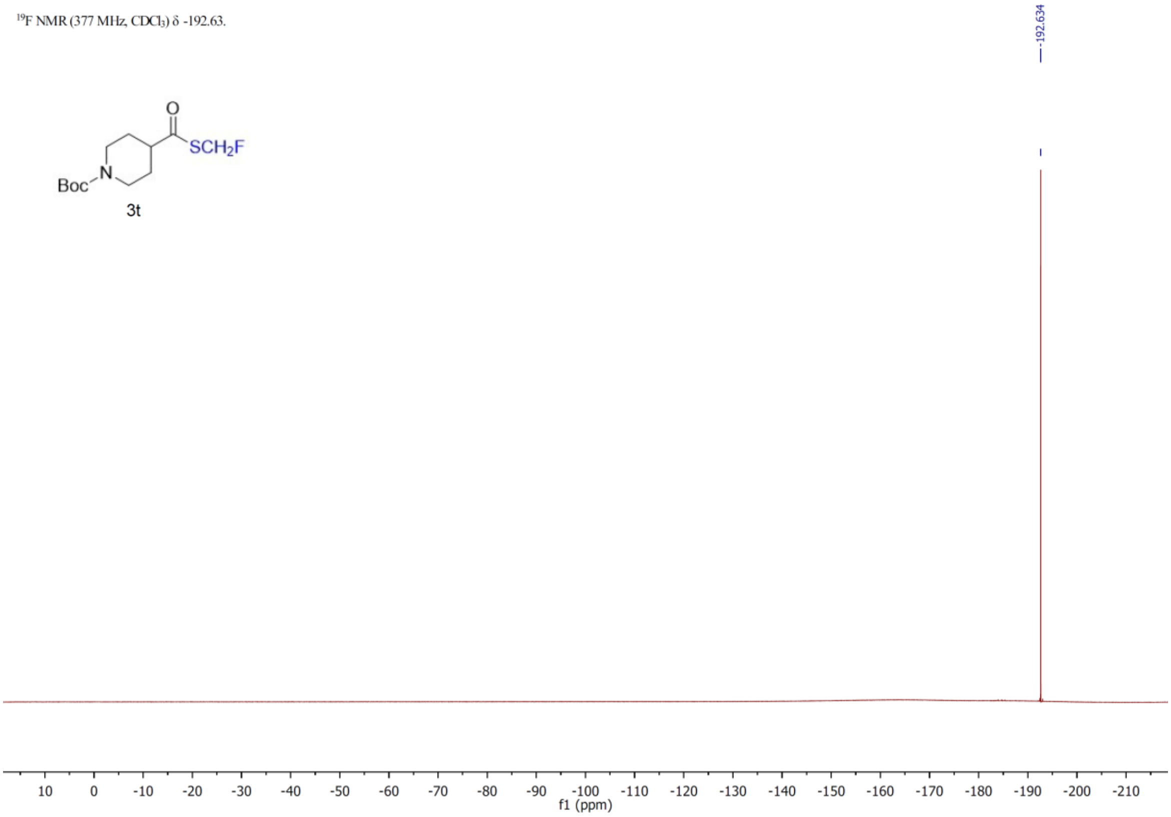

Supplementary Figure 53a | ^{1}H NMR (400 MHz, $CDCl_3$) of (*1R*,*2S*,*5R*)-2-Isopropyl-5-methylcyclohexyl 4-(((fluoromethyl)thio)carbonyl)benzoate (**3u**)

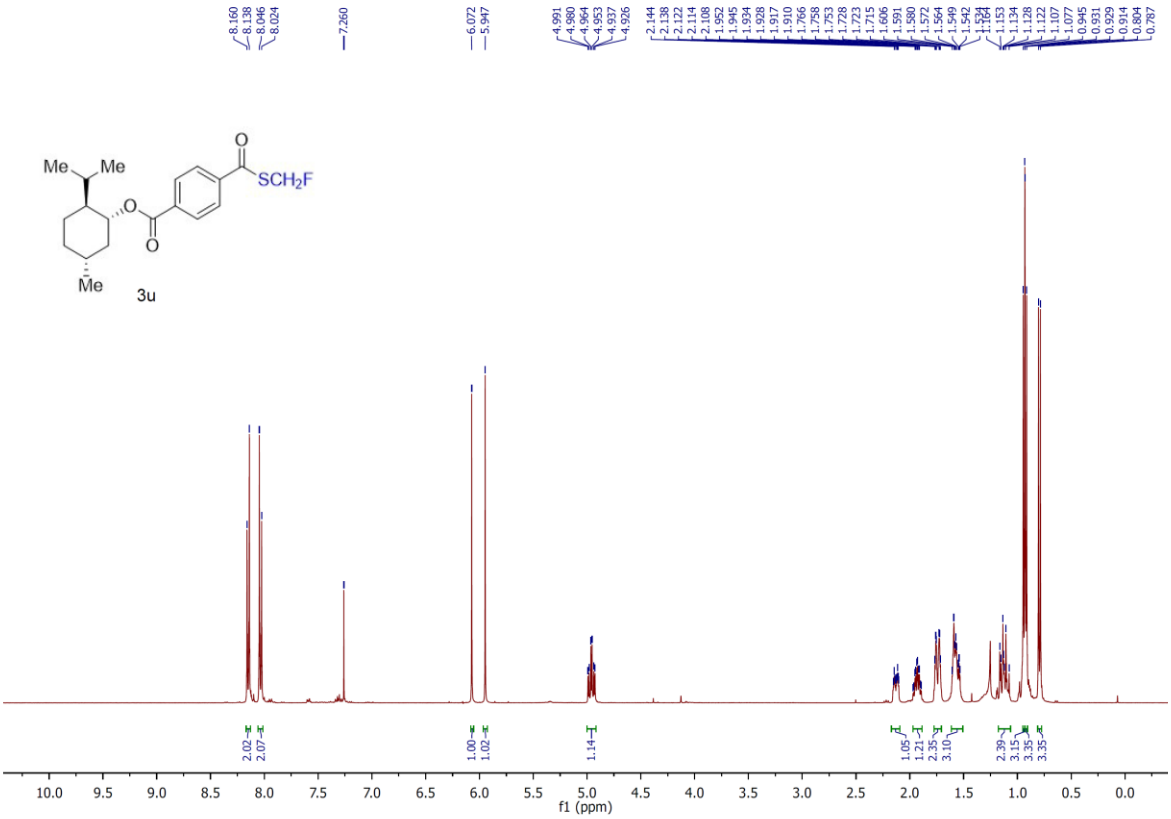

Supplementary Figure 53b | ^{13}C NMR (126 MHz, $CDCl_3$) of (*1R*,*2S*,*5R*)-2-Isopropyl-5-methylcyclohexyl 4-(((fluoromethyl)thio)carbonyl)benzoate (**3u**)

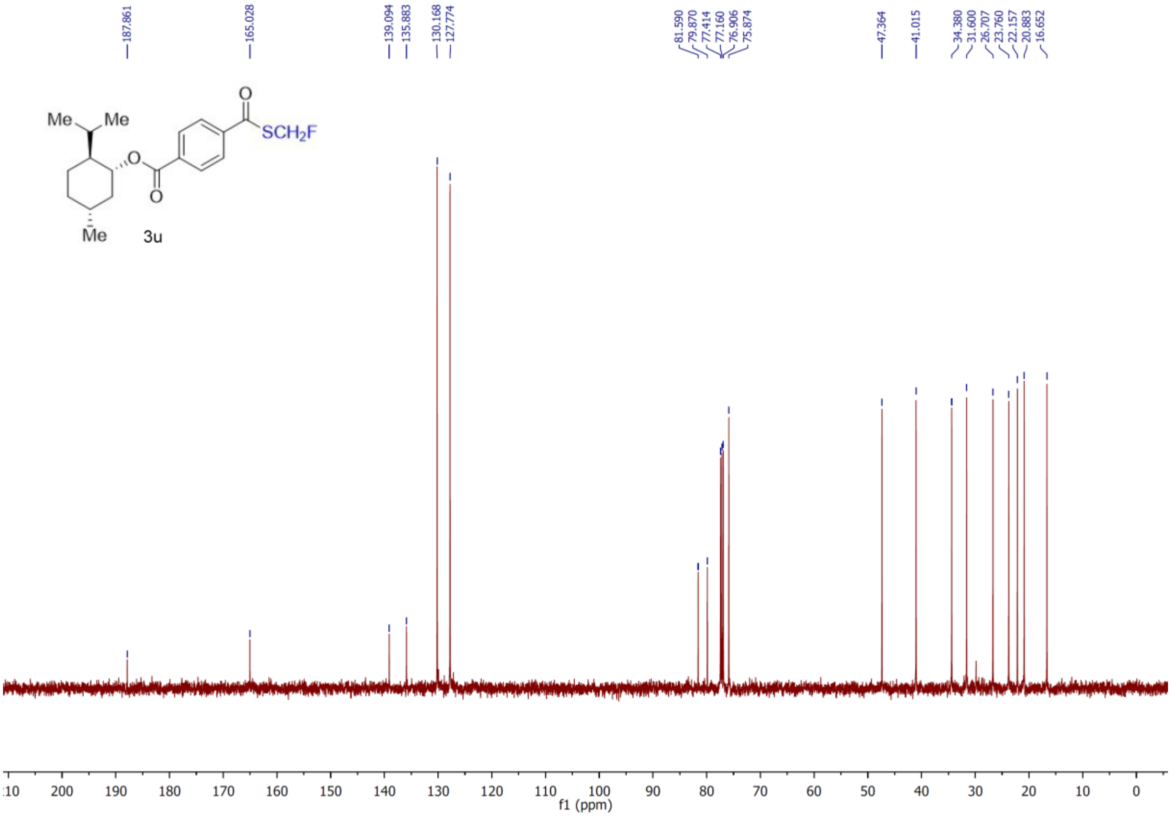

Supplementary Figure 53c | ^{19}F NMR (377 MHz, $CDCl_3$) of (*1R,2S,5R*)-2-Isopropyl-5-methylcyclohexyl 4-(((fluoromethyl)thio)carbonyl)benzoate (**3u**)

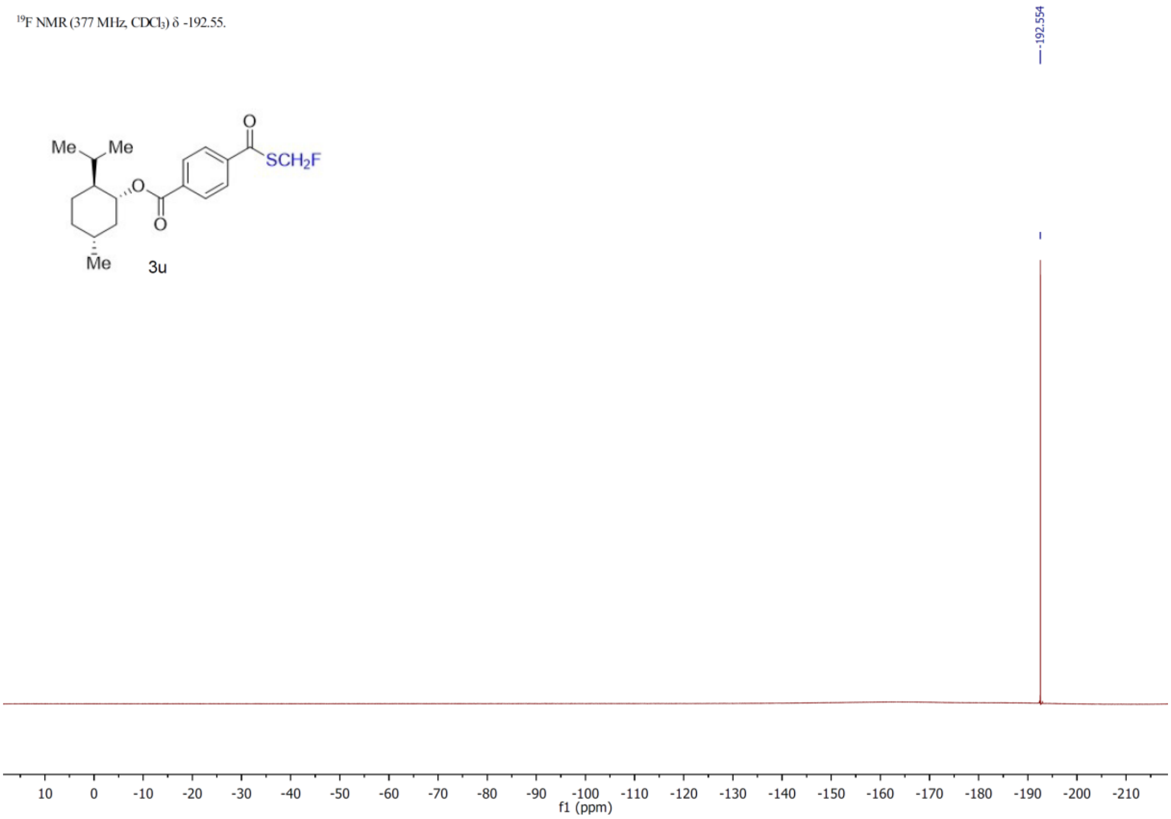

Supplementary Figure 54a | ^{1}H NMR (400 MHz, $CDCl_3$) of (*1R,2R,4S*)-1,3,3-Trimethylbicyclo[2.2.1]heptan-2-yl 4-(((fluoromethyl)thio)carbonyl)benzoate (**3v**)

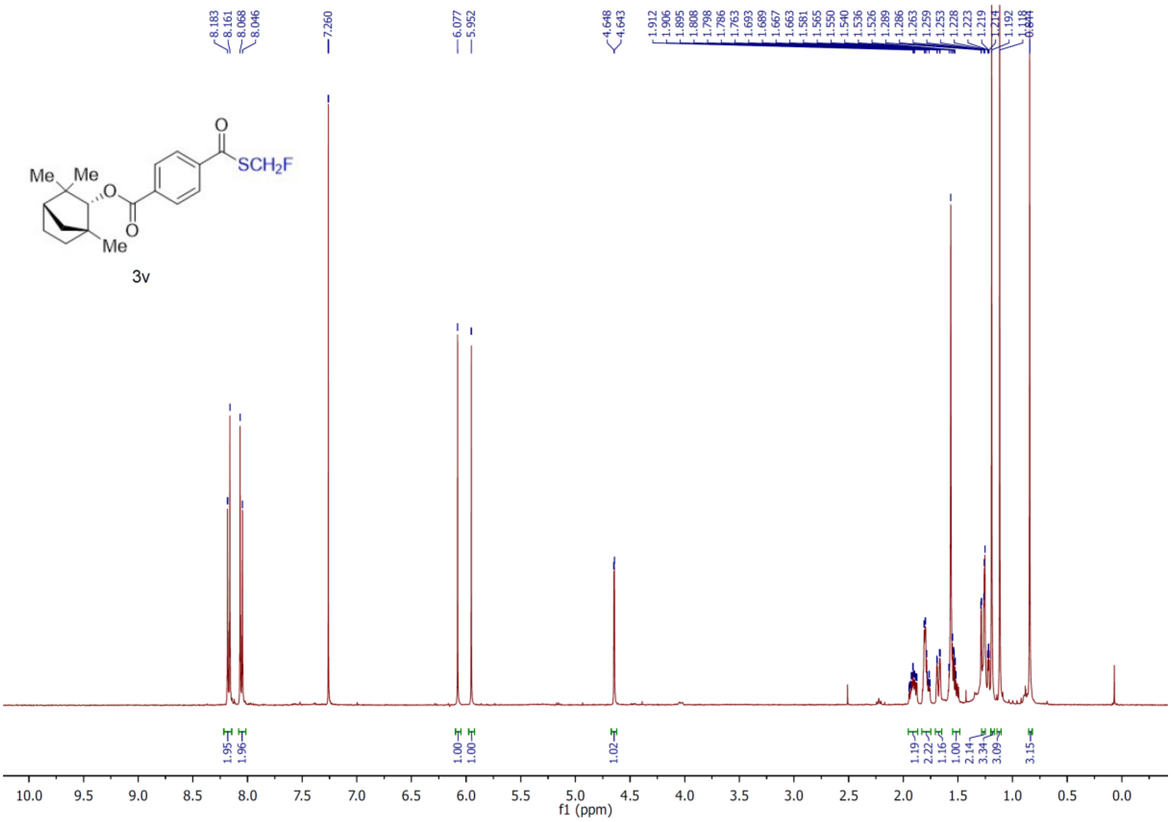

Supplementary Figure 54b | ^{13}C NMR (126 MHz, $CDCl_3$) of (*1R,2R,4S*)-1,3,3-Trimethylbicyclo[2.2.1]heptan-2-yl 4-(((fluoromethyl)thio)carbonyl)benzoate (**3v**)

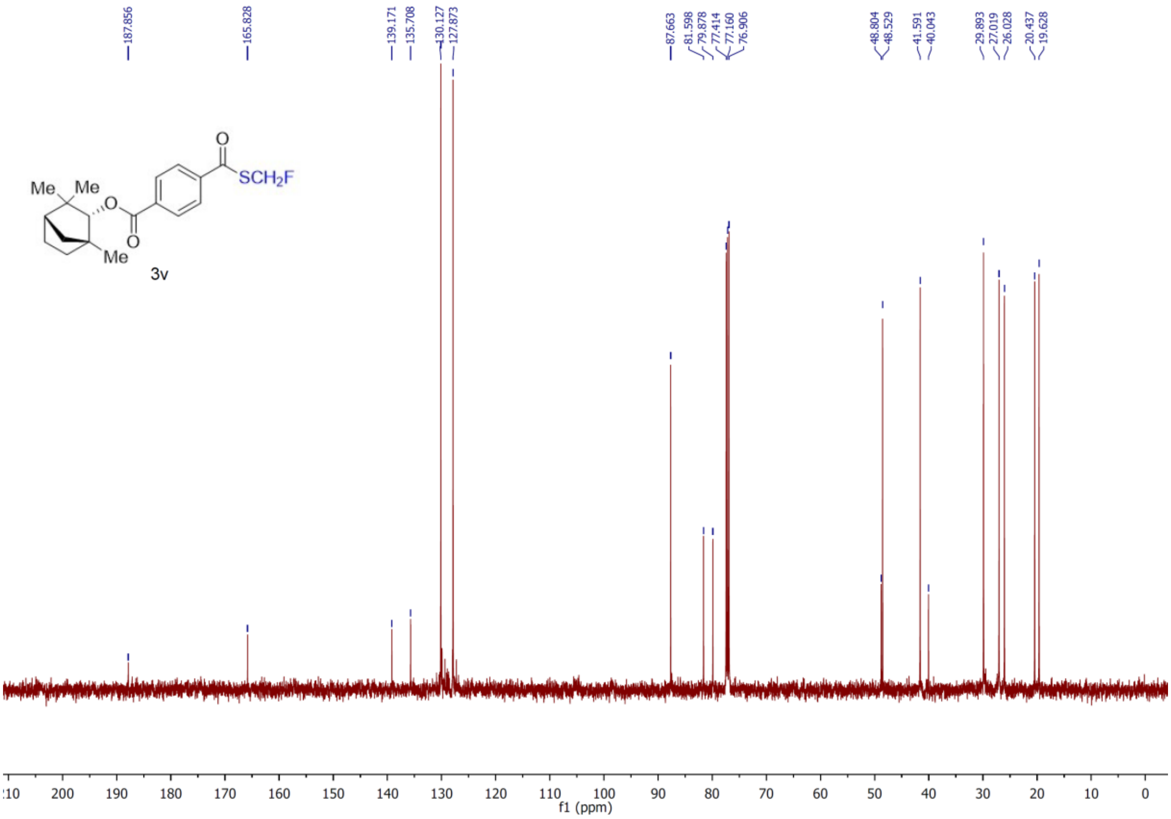

Supplementary Figure 54c | ^{19}F NMR (377 MHz, $CDCl_3$) of (*1R,2R,4S*)-1,3,3-Trimethylbicyclo[2.2.1]heptan-2-yl 4-(((fluoromethyl)thio)carbonyl)benzoate (**3v**)

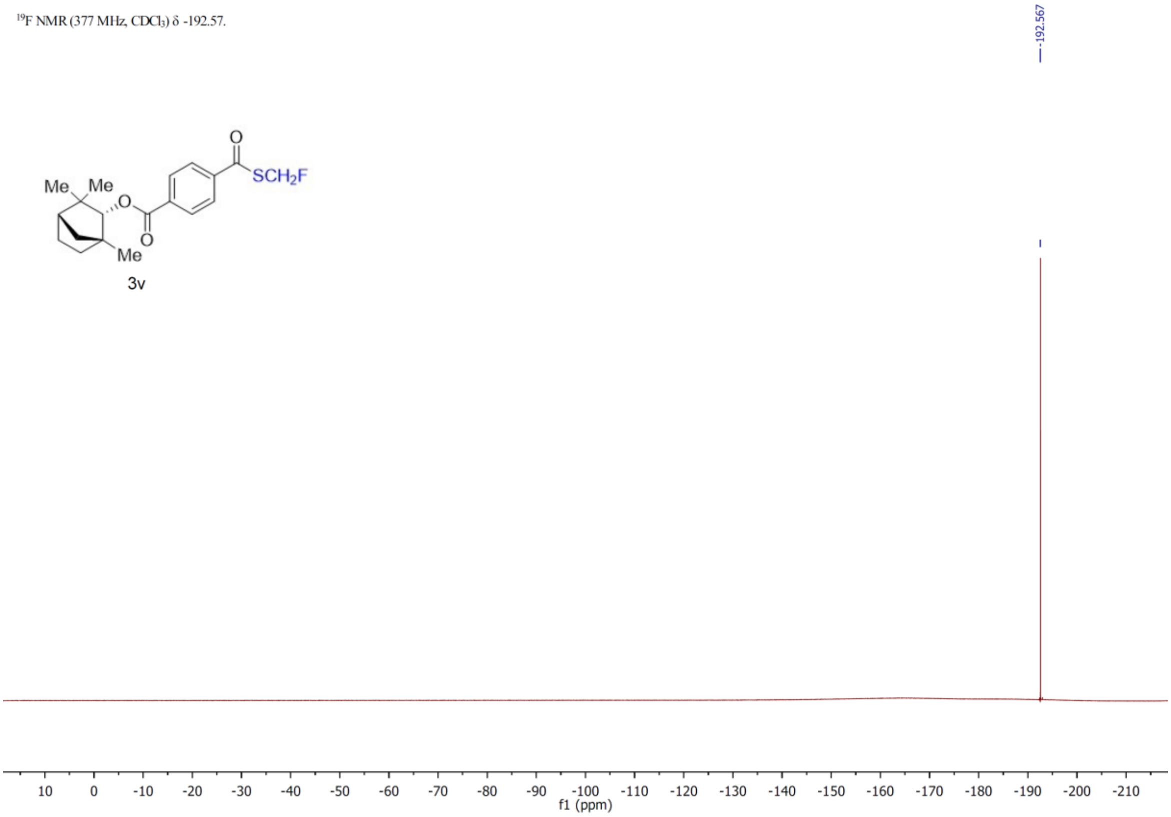

Supplementary Figure 55a | ^{1}H NMR (400 MHz, $CDCl_3$) of *S*-(fluoromethyl) (R)-4-((5R,8R,9S,10S,13R,14S,17R)-10,13-dimethyl-3-oxohexadecahydro-1H-cyclopenta[a]phenanthren-17-yl)pentanethioate (**3w**)

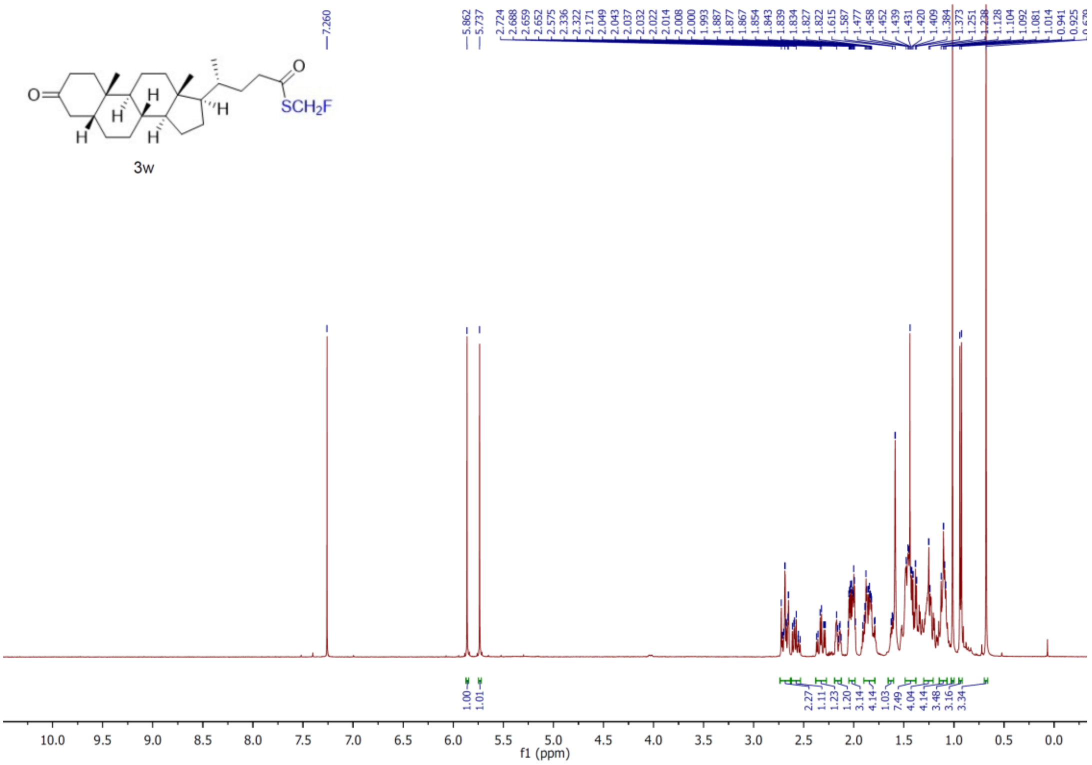

Supplementary Figure 55b | ^{13}C NMR (126 MHz, $CDCl_3$) of *S*-(fluoromethyl) (R)-4-((5R,8R,9S,10S,13R,14S,17R)-10,13-dimethyl-3-oxohexadecahydro-1H-cyclopenta[a]phenanthren-17-yl)pentanethioate (**3w**)

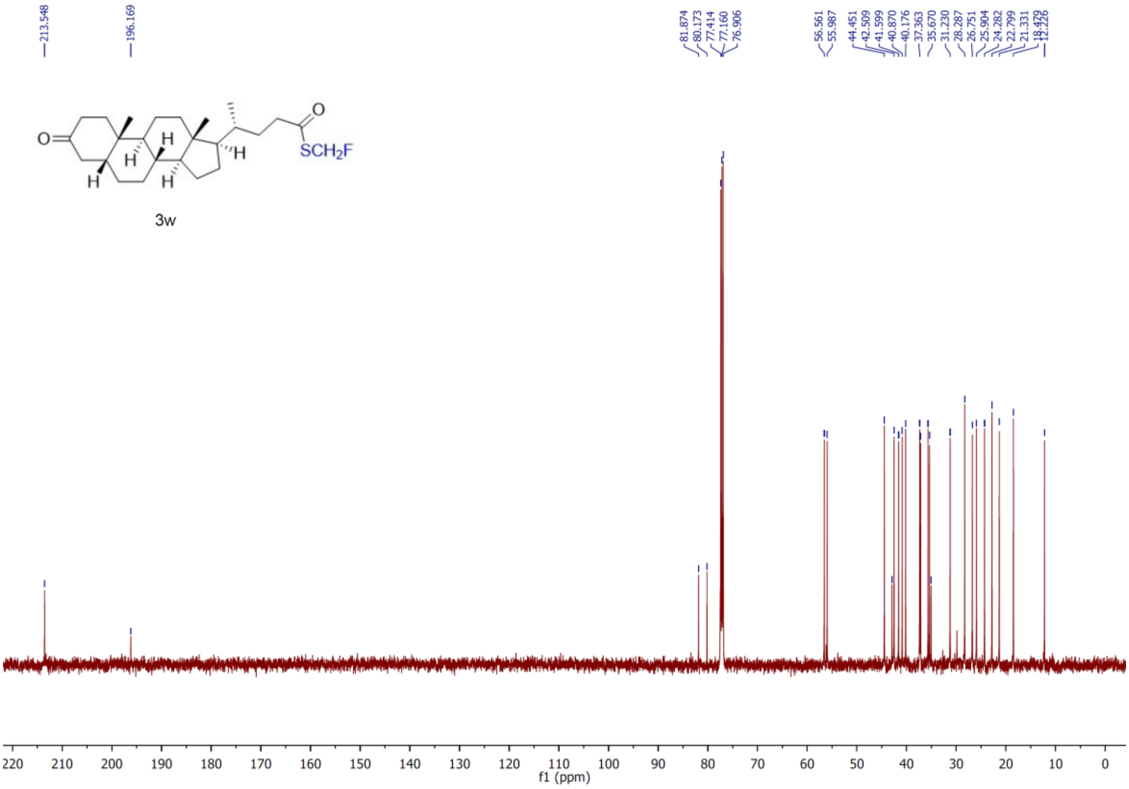

Supplementary Figure 55c | ^{19}F NMR (377 MHz, $CDCl_3$) of *S*-(fluoromethyl) (R)-4-((5R,8R,9S,10S,13R,14S,17R)-10,13-dimethyl-3-oxohexadecahydro-1H-cyclopenta[a]phenanthren-17-yl)pentanethioate (**3w**)

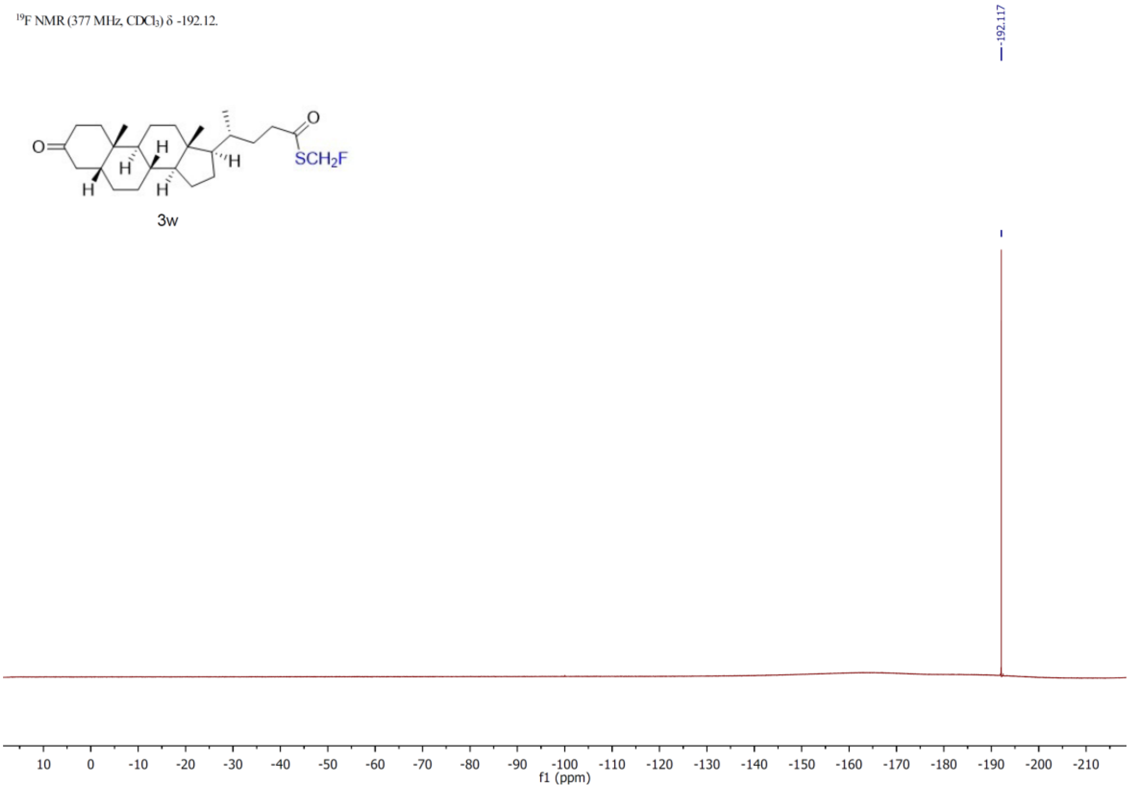

Supplementary Figure 56a | ^{1}H NMR (400 MHz, $CDCl_3$) of *S*-(difluoromethyl) 4-fluorobenzothioate (**3x**)

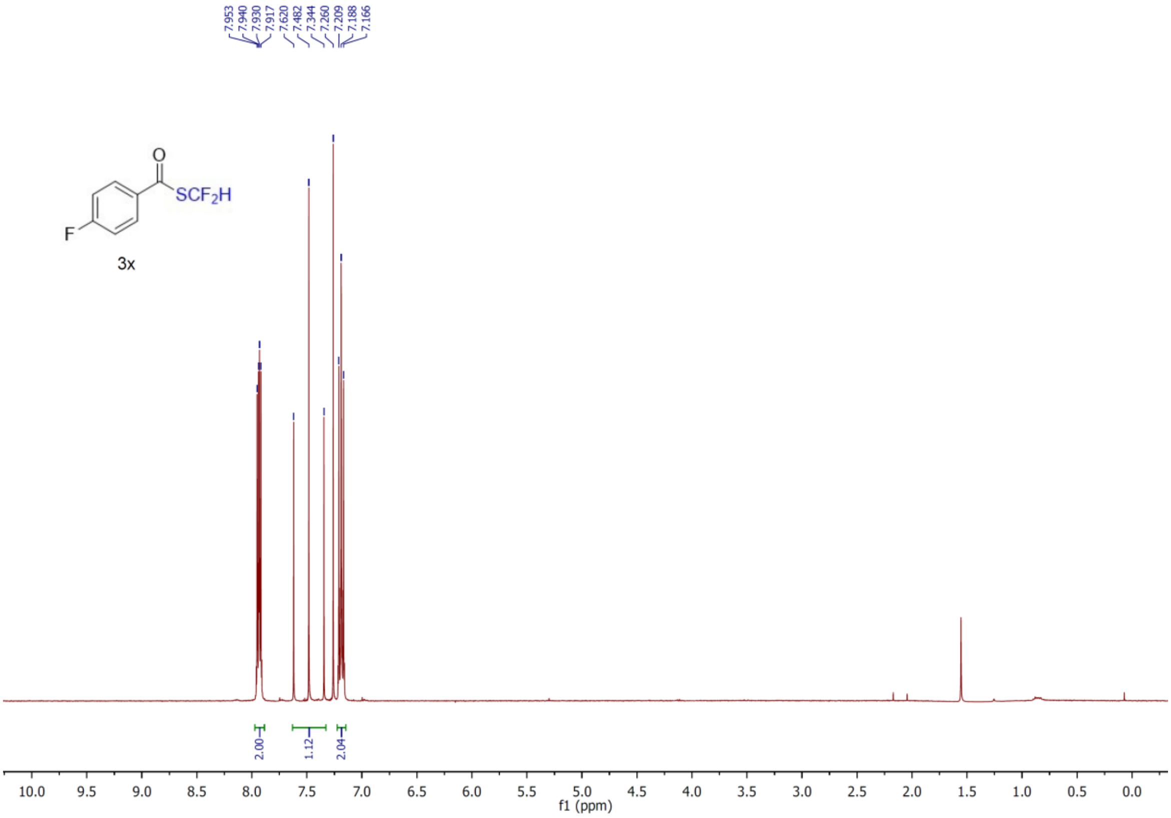

Supplementary Figure 56b | ^{13}C NMR (126 MHz, $CDCl_3$) of *S*-(difluoromethyl) 4-fluorobenzothioate (**3x**)

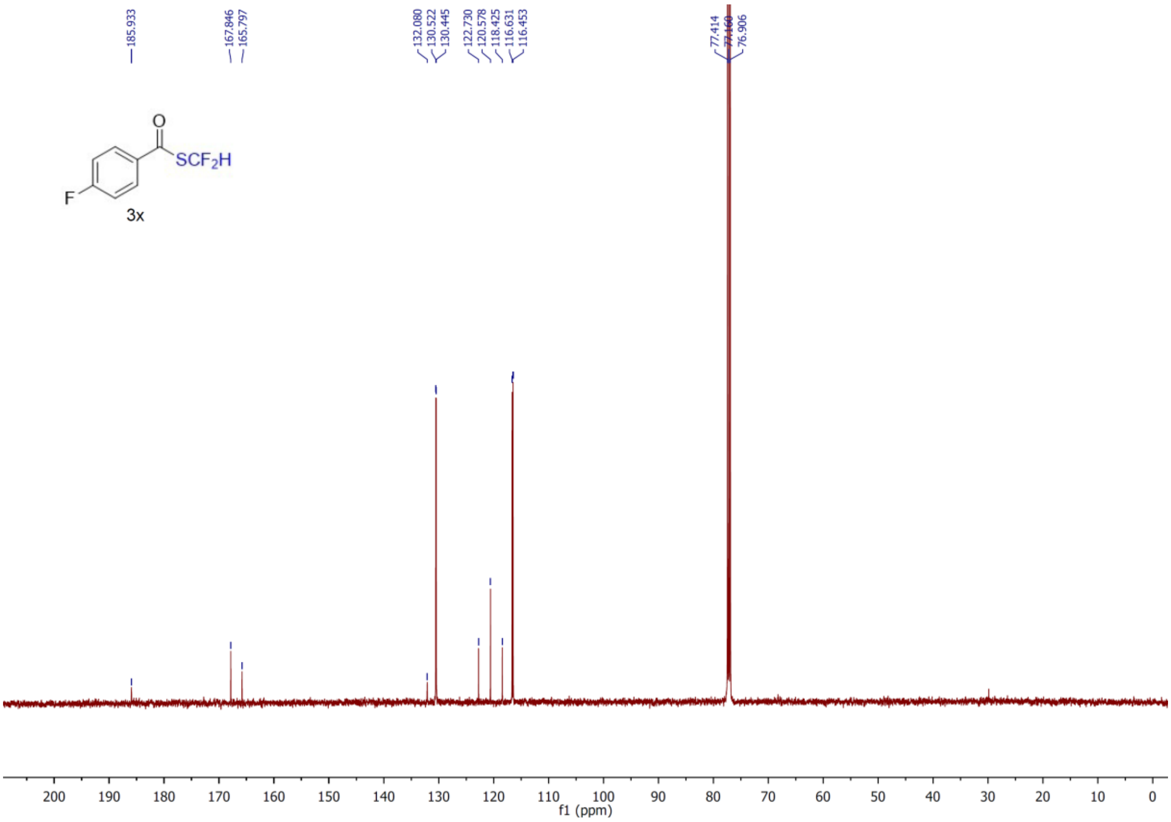

Supplementary Figure 56c | ^{19}F NMR (377 MHz, $CDCl_3$) of *S*-(difluoromethyl) 4-fluorobenzothioate (**3x**)

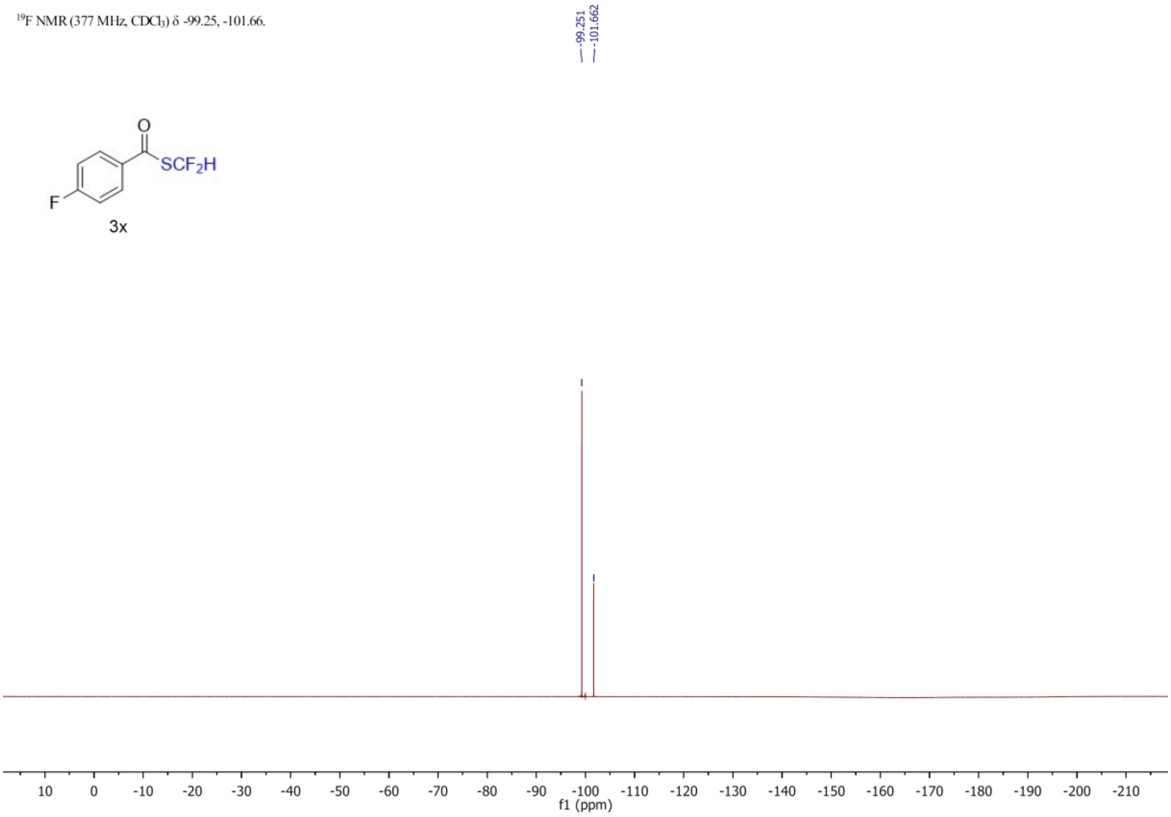

Supplementary Figure 57a | ^{1}H NMR (400 MHz, $CDCl_3$) of *S*-(trifluoromethyl) naphthalene-2-carbothioate (**3y**)

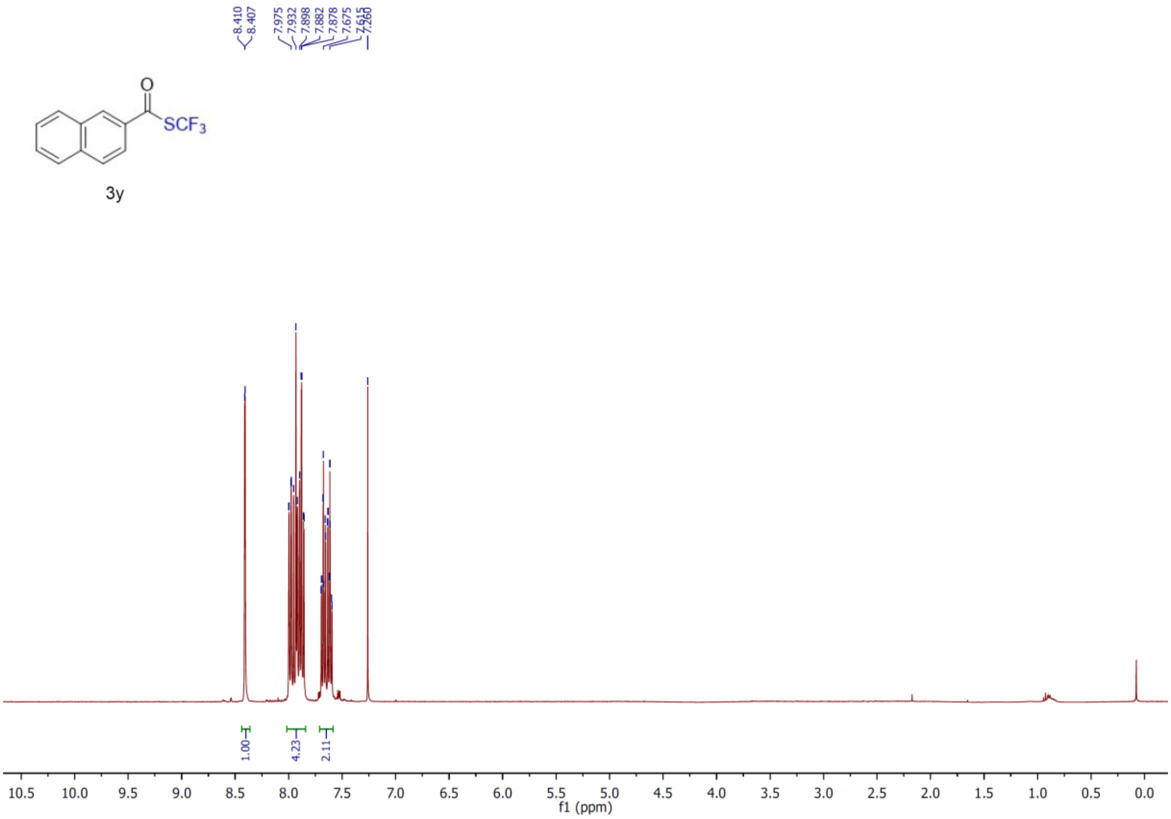

Supplementary Figure 57b | ^{13}C NMR (126 MHz, $CDCl_3$) of *S*-(trifluoromethyl) naphthalene-2-carbothioate (**3y**)

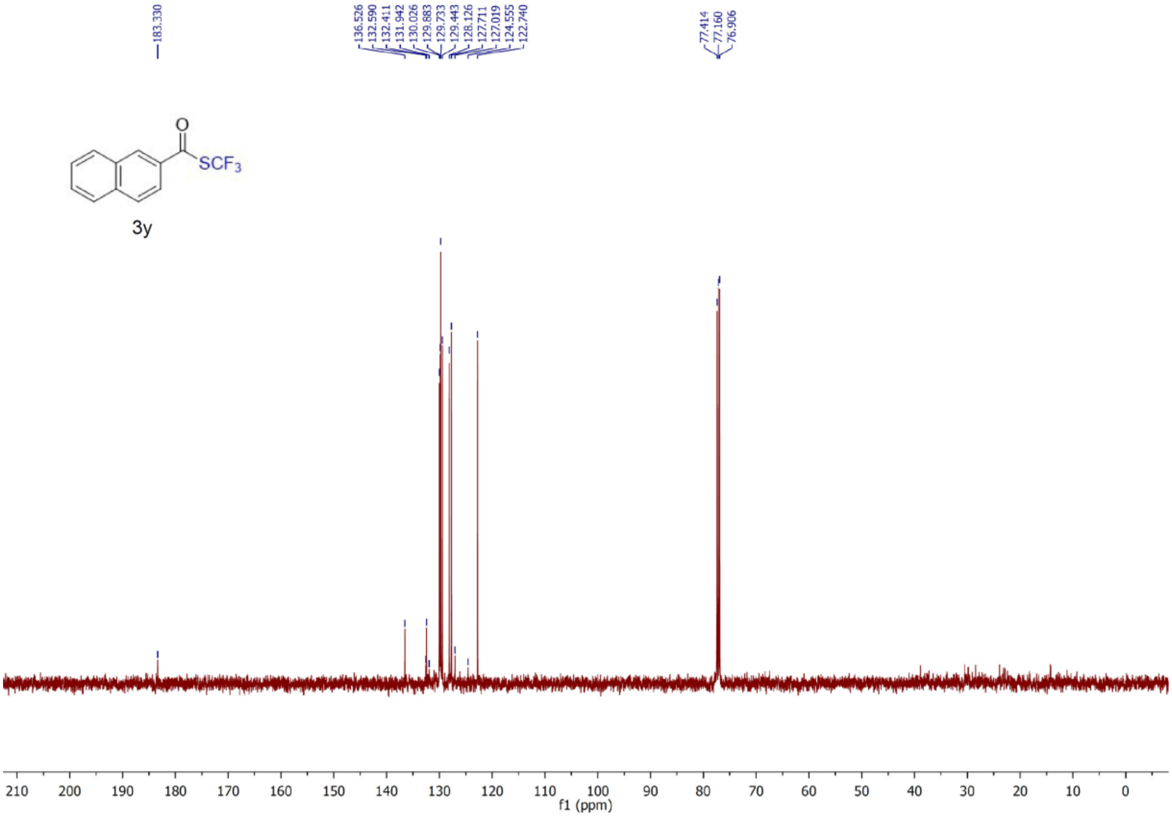

Supplementary Figure 57c | ^{19}F NMR (377 MHz, $CDCl_3$) of *S*-(trifluoromethyl) naphthalene-2-carbothioate (**3y**)

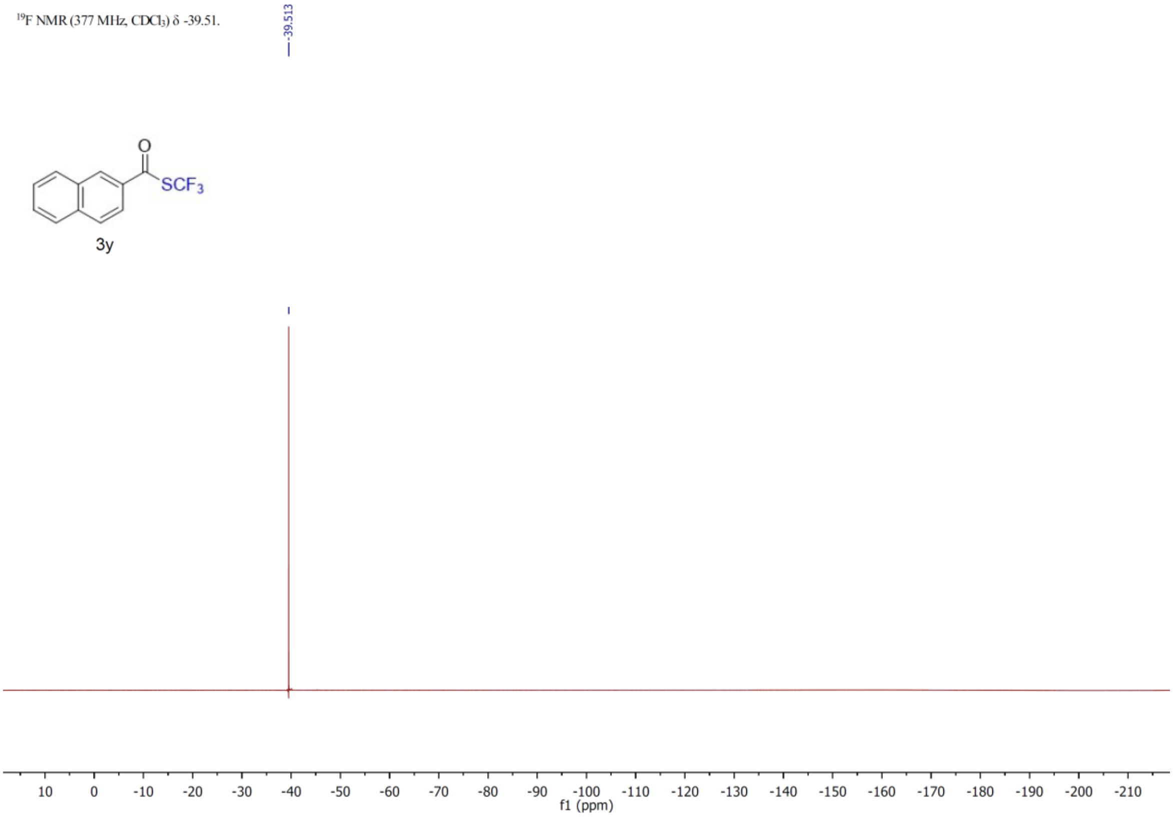

Supplementary Figure 58a | ^{1}H NMR (400 MHz, $CDCl_3$) of *S*-(trifluoromethyl) [1,1'-biphenyl]-4-carbothioate (**3z**)

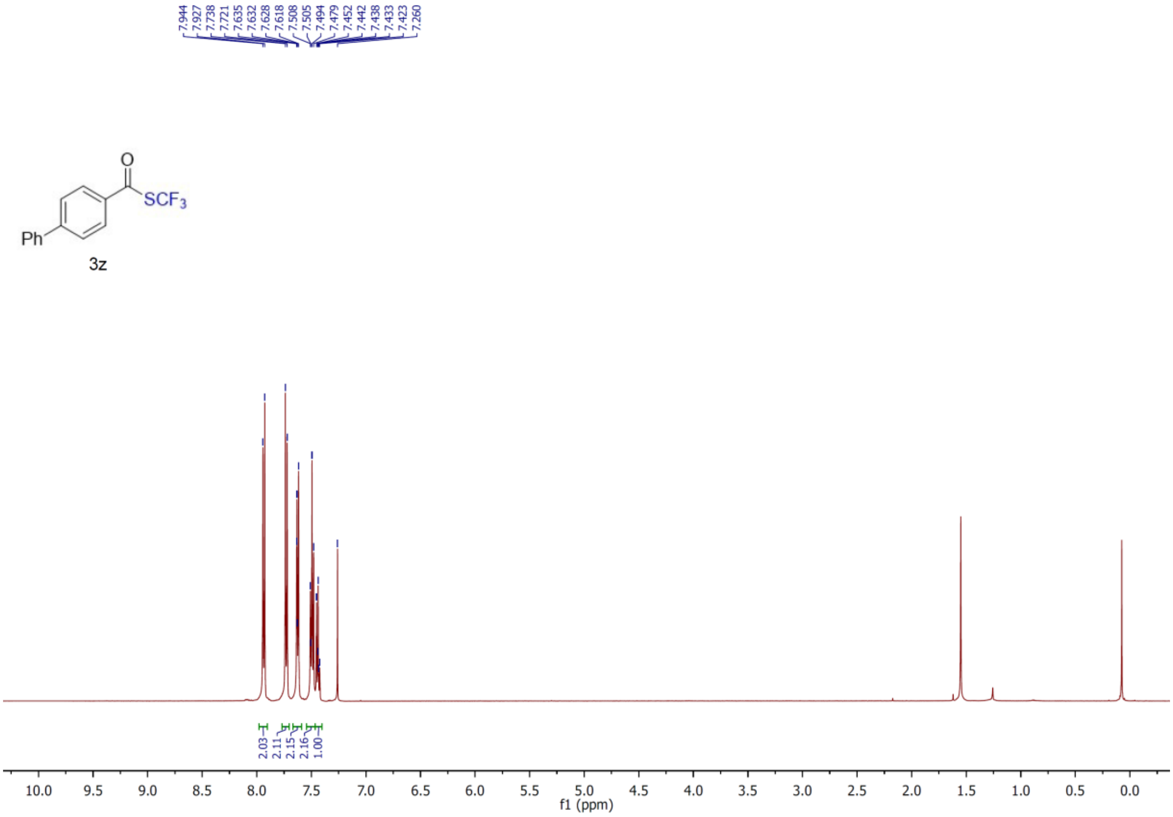

Supplementary Figure 58b | ^{13}C NMR (126 MHz, $CDCl_3$) of *S*-(trifluoromethyl) [1,1'-biphenyl]-4-carbothioate (**3z**)

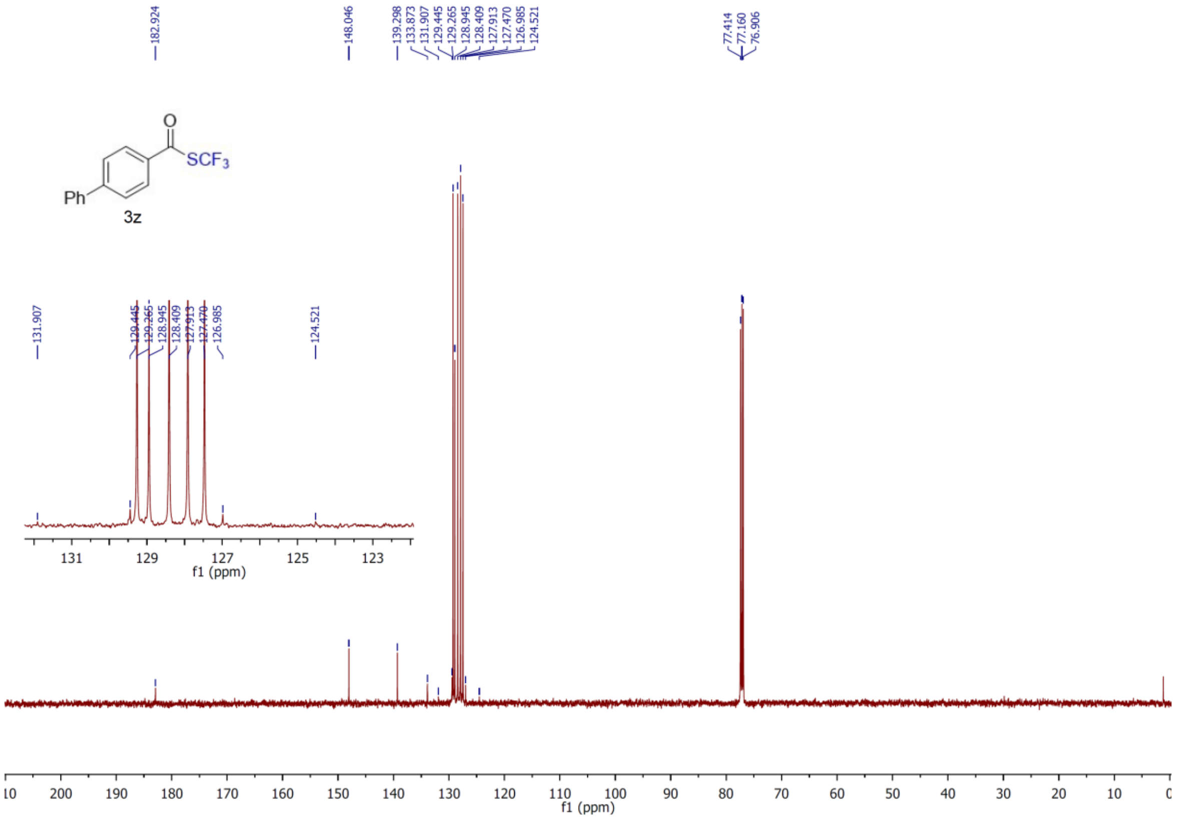

Supplementary Figure 58c | ^{19}F NMR (377 MHz, $CDCl_3$) of *S*-(trifluoromethyl) [1,1'-biphenyl]-4-carbothioate (**3z**)

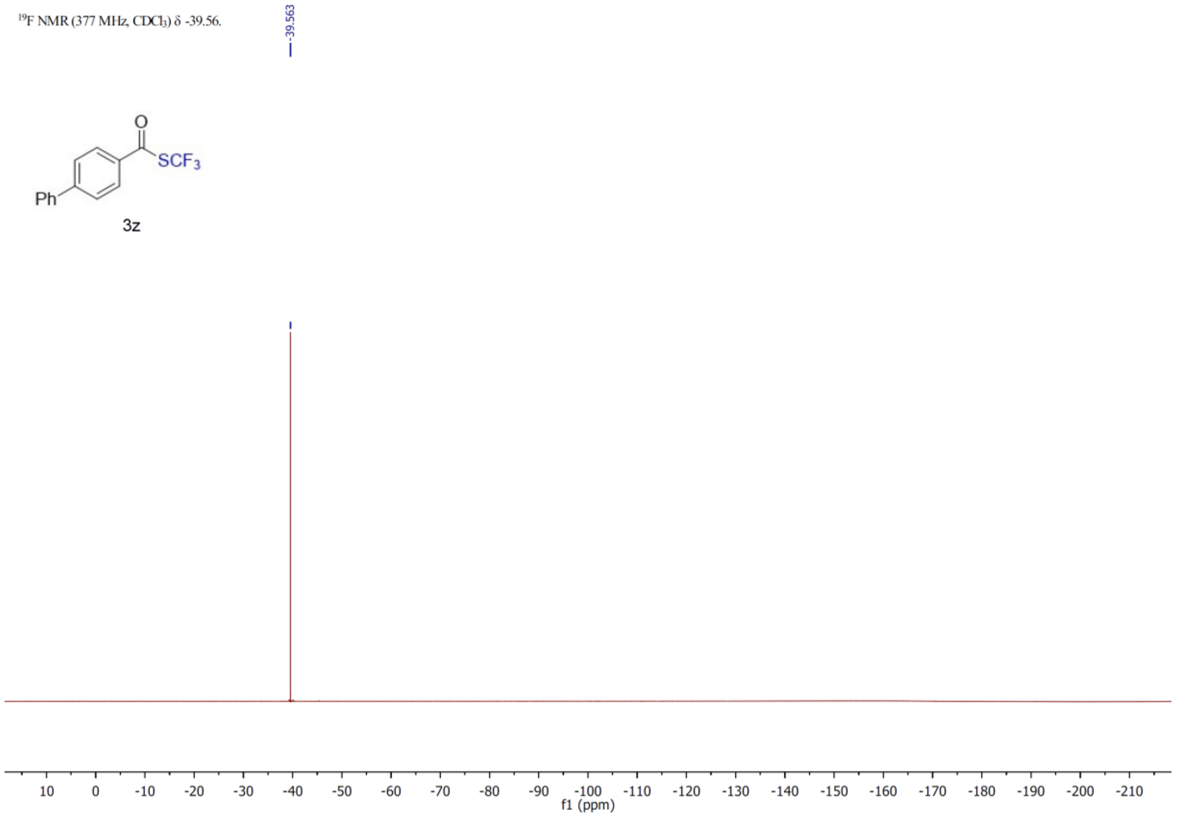

Supplementary Figure 59a | ^{1}H NMR (400 MHz, $CDCl_3$) of *S*-(fluoromethyl) 4-(3-((fluoromethyl)thio)-3-oxopropyl)benzothioate (**S2**)

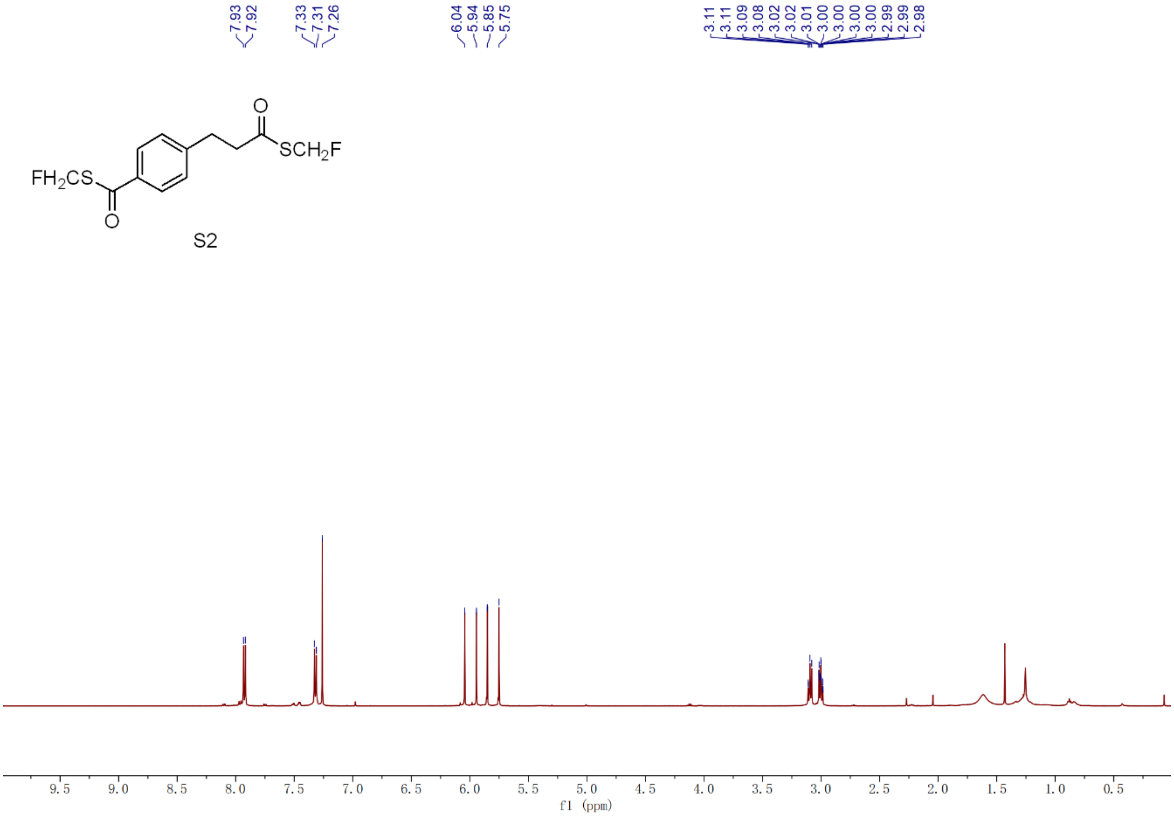

Supplementary Figure 59b | ^{13}C NMR (126 MHz, $CDCl_3$) of *S*-(fluoromethyl) 4-(3-((fluoromethyl)thio)-3-oxopropyl)benzothioate (**S2**)

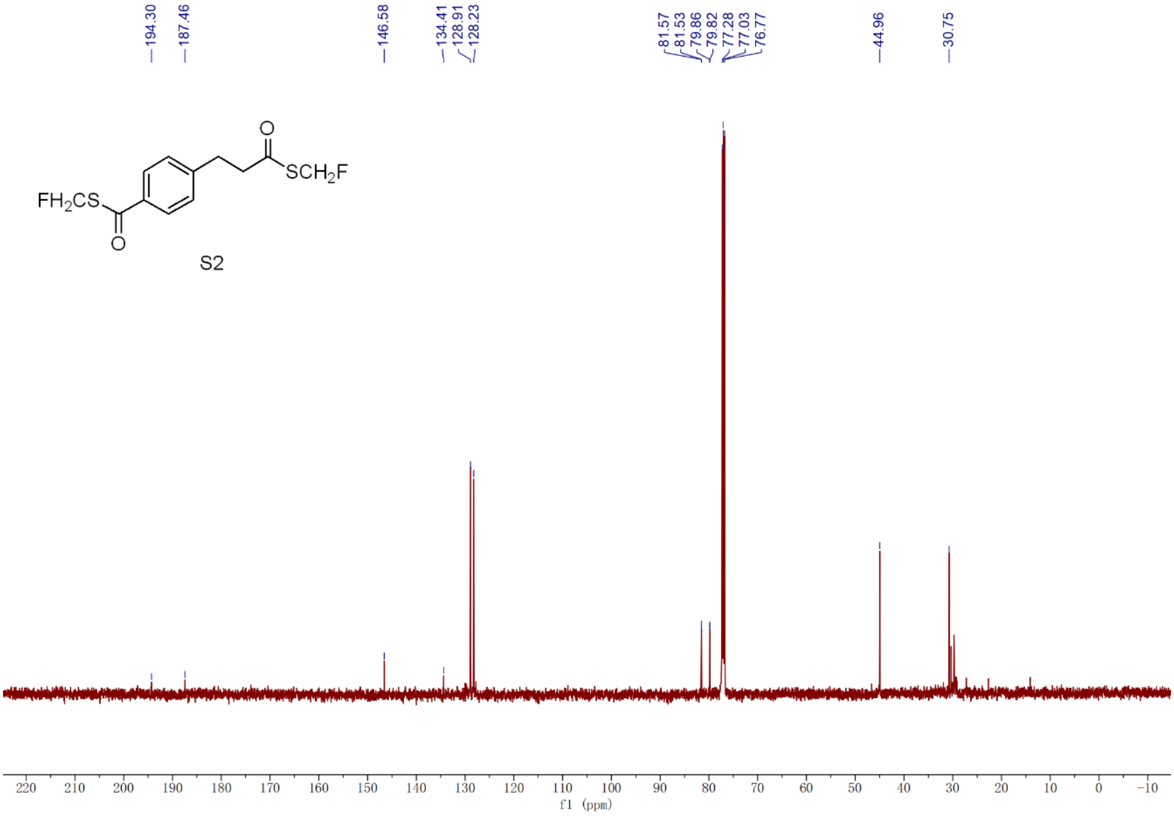

Supplementary Figure 59c | ^{19}F NMR (377 MHz, $CDCl_3$) of *S*-(fluoromethyl) 4-(3-((fluoromethyl)thio)-3-oxopropyl)benzothioate (**S2**)

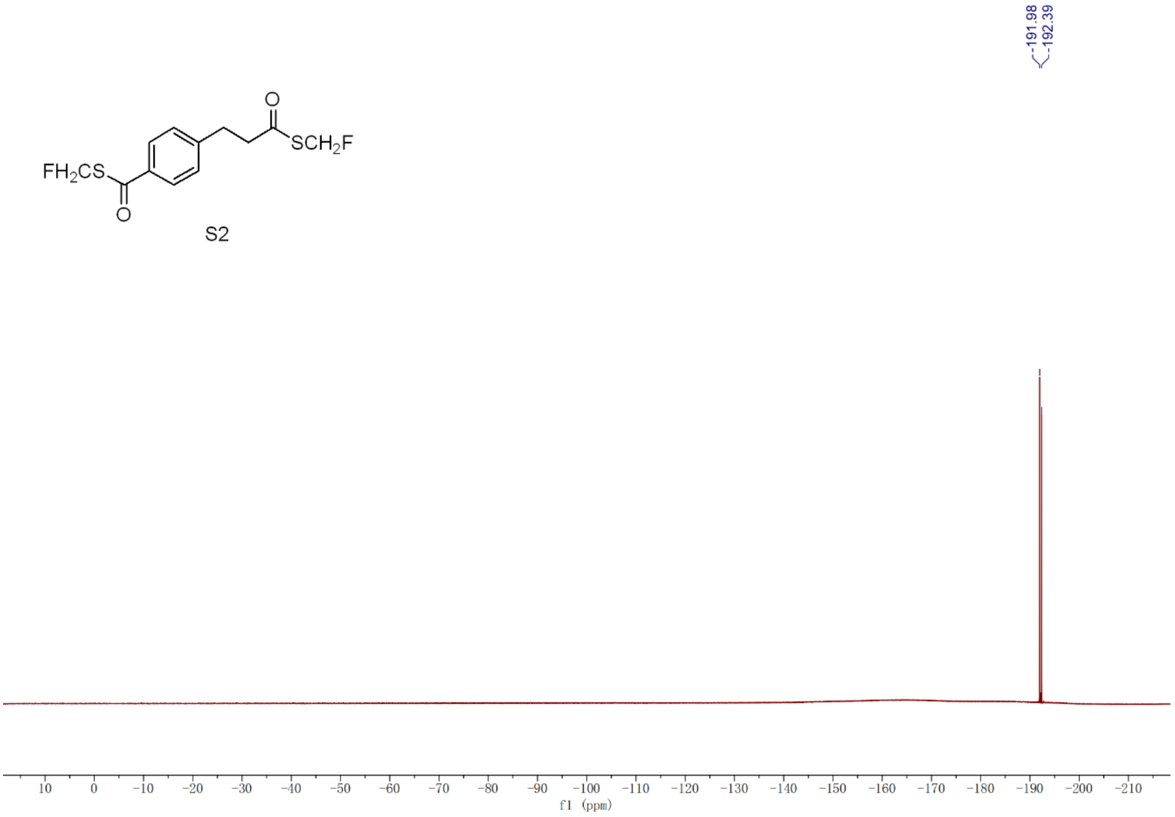

Supplementary Figure 60a | ^{1}H NMR (500 MHz, $CDCl_3$) of *S*-(fluoromethyl) 3-(4-formylphenyl)propanethioate (**S3**)

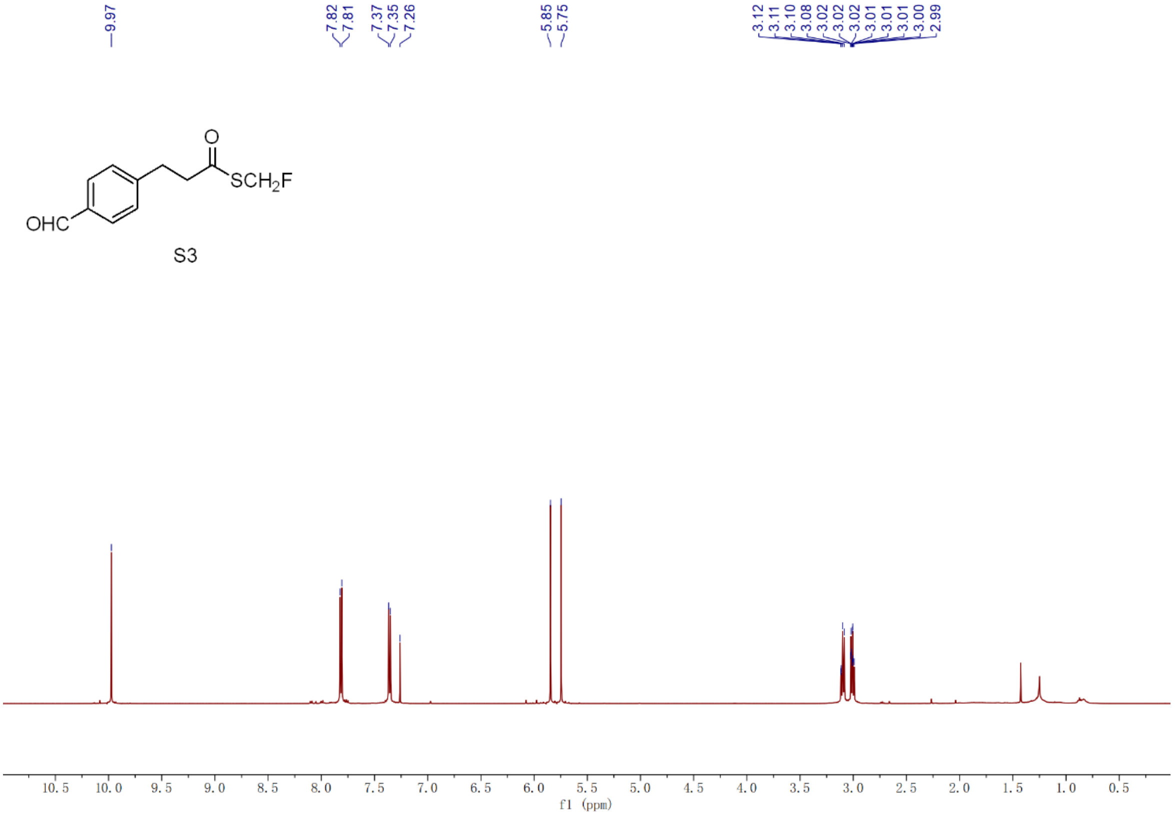

Supplementary Figure 60b | ^{13}C NMR (126 MHz, $CDCl_3$) of *S*-(fluoromethyl) 3-(4-formylphenyl)propanethioate (**S3**)

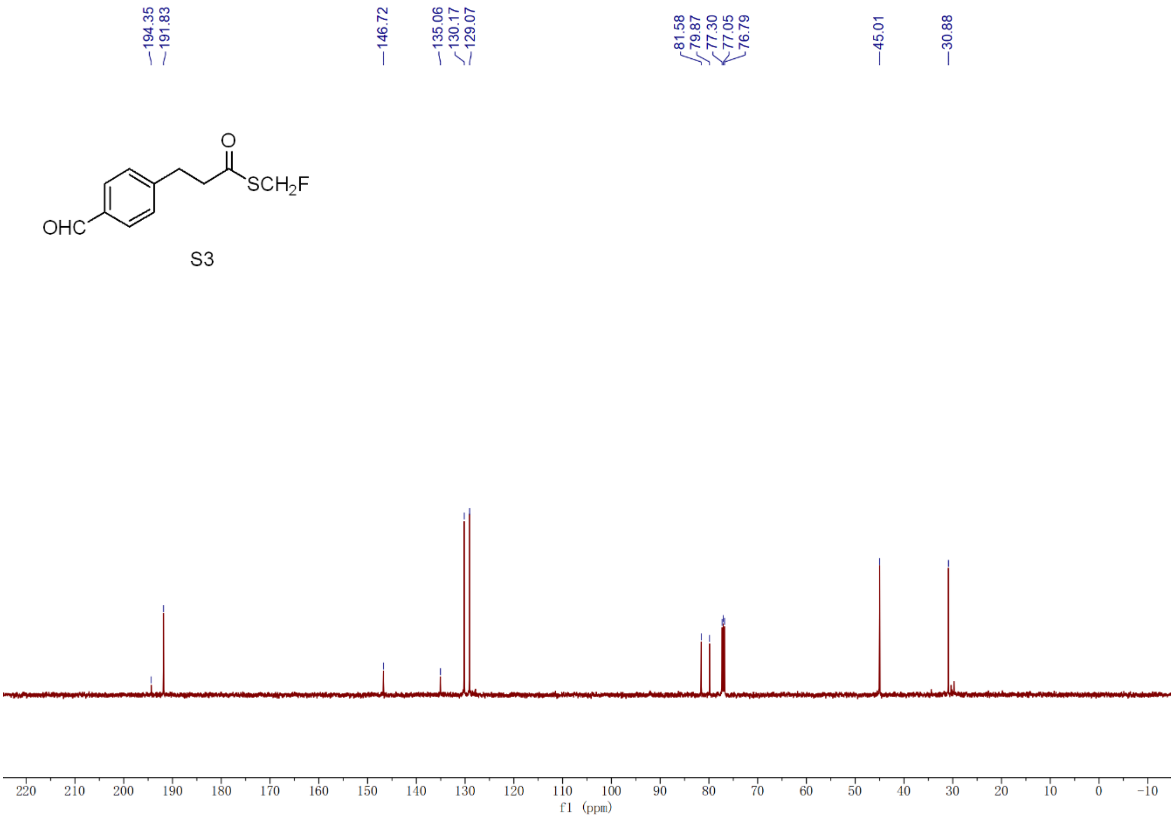

Supplementary Figure 60c | ^{19}F NMR (377 MHz, $CDCl_3$) of *S*-(fluoromethyl) 3-(4-formylphenyl)propanethioate (**S3**)

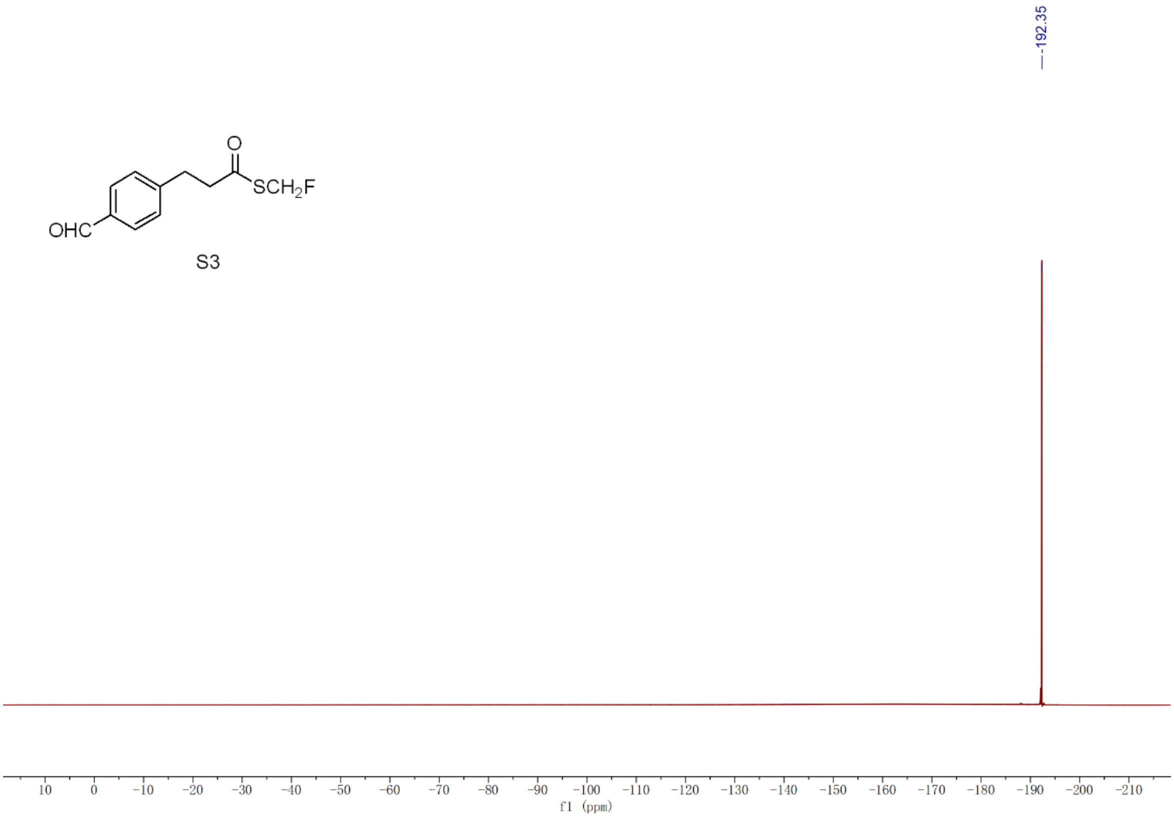

Supplementary Figure 61a | ^{1}H NMR (500 MHz, $CDCl_3$) of *S*-(p-tolyl) benzothioate (**4a**)

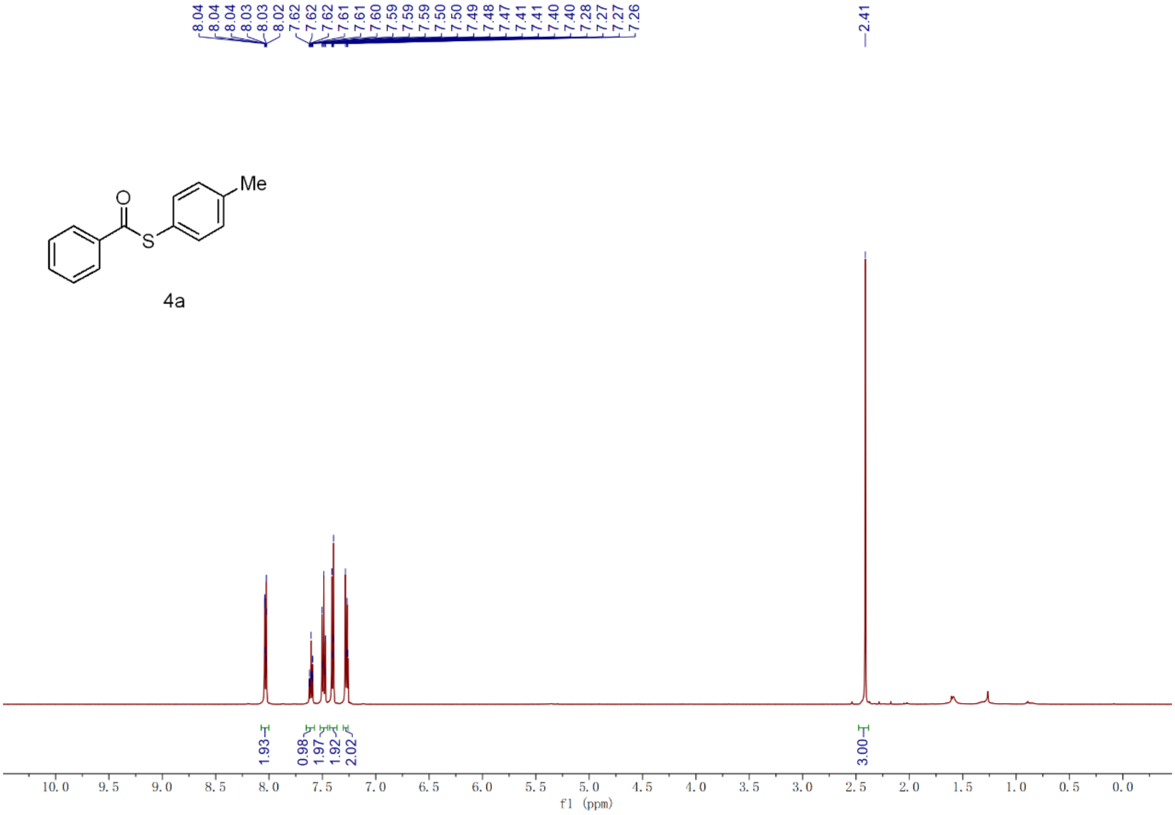

Supplementary Figure 61b | ^{13}C NMR (126 MHz, $CDCl_3$) of *S*-(p-tolyl) benzothioate (**4a**)

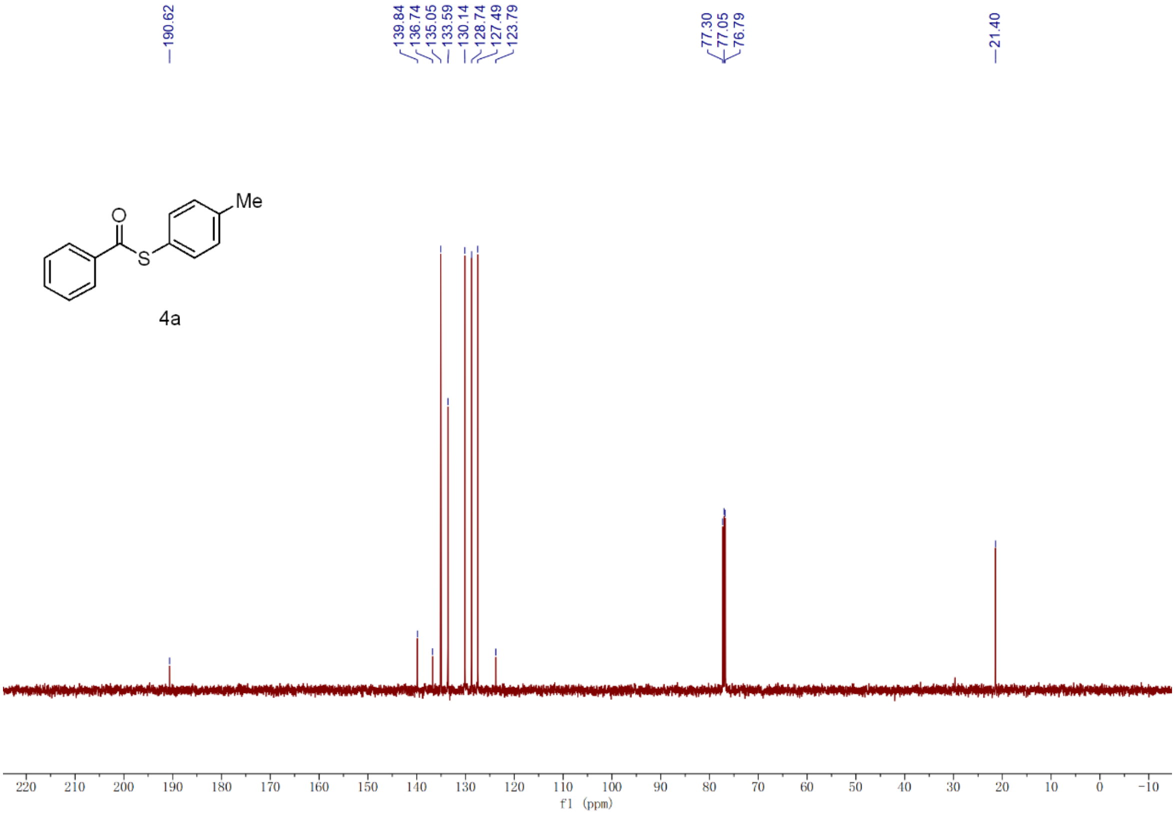

Supplementary Figure 62a | ^{1}H NMR (500 MHz, $CDCl_3$) of *S*-phenyl 4-methoxybenzothioate (**4b**)

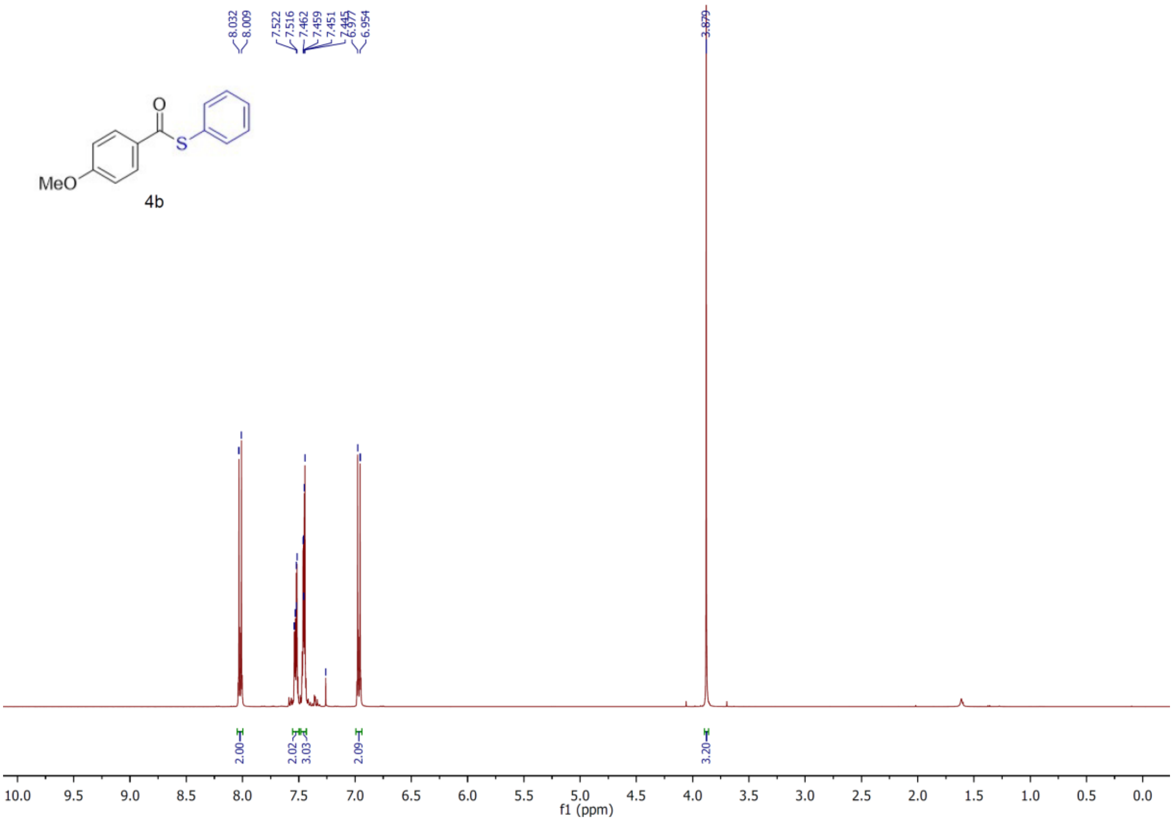

Supplementary Figure 62b | ^{13}C NMR (126 MHz, $CDCl_3$) of *S*-phenyl 4-methoxybenzothioate (**4b**)

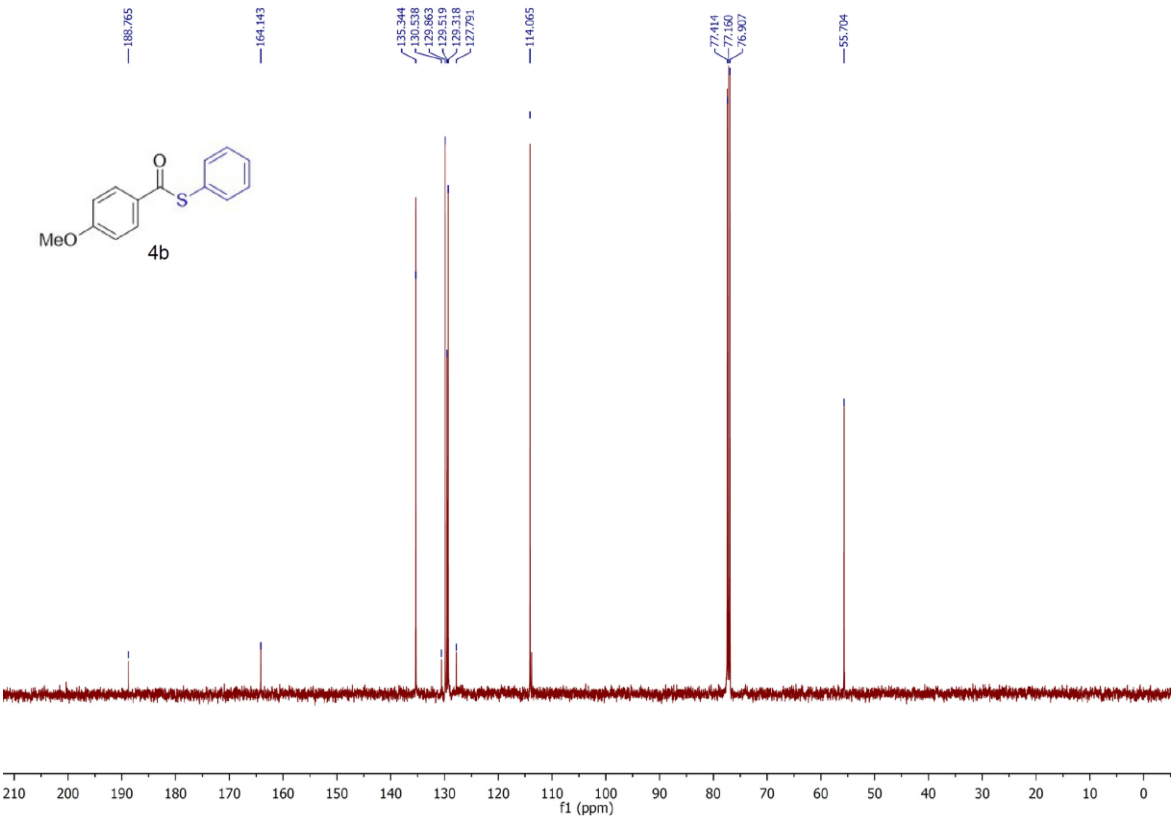

Supplementary Figure 63a | ^{1}H NMR (500 MHz, $CDCl_3$) of *S*-(p-tolyl) 4-methoxybenzothioate (**4c**)

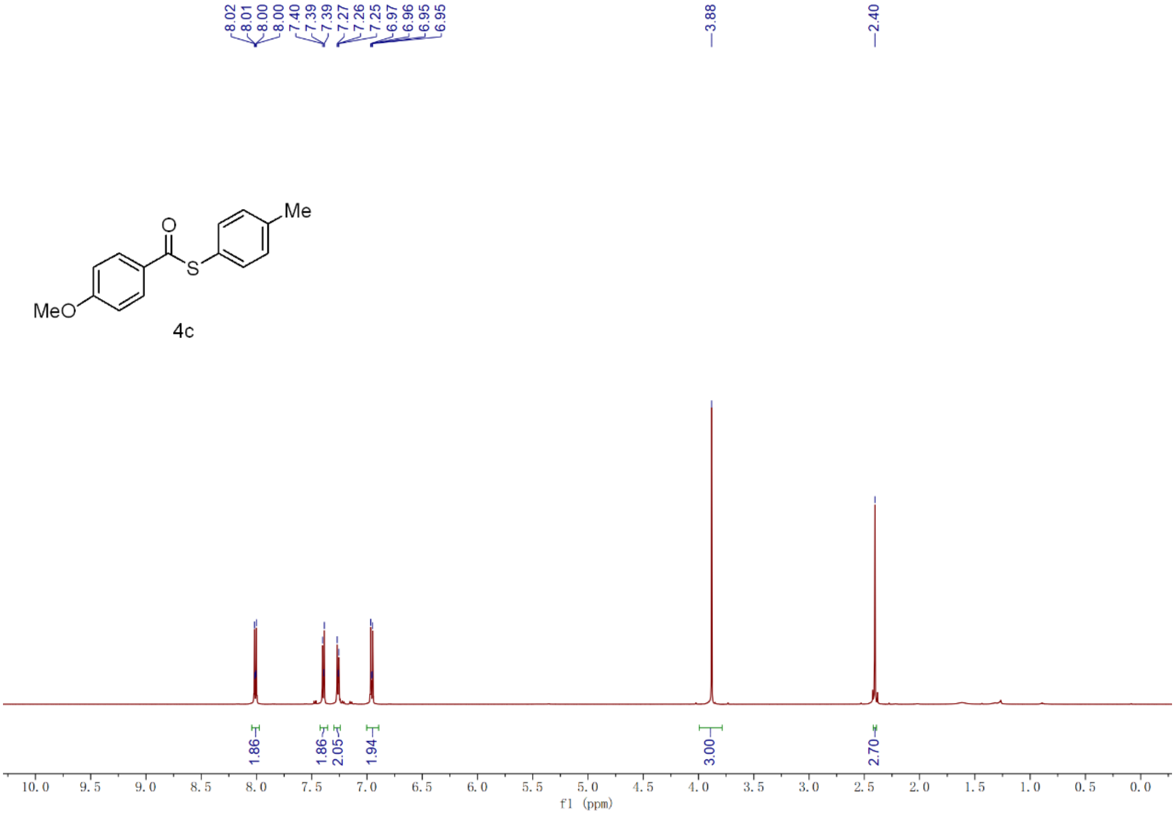

Supplementary Figure 63b | ^{13}C NMR (126 MHz, $CDCl_3$) of *S*-(p-tolyl) 4-methoxybenzothioate (**4c**)

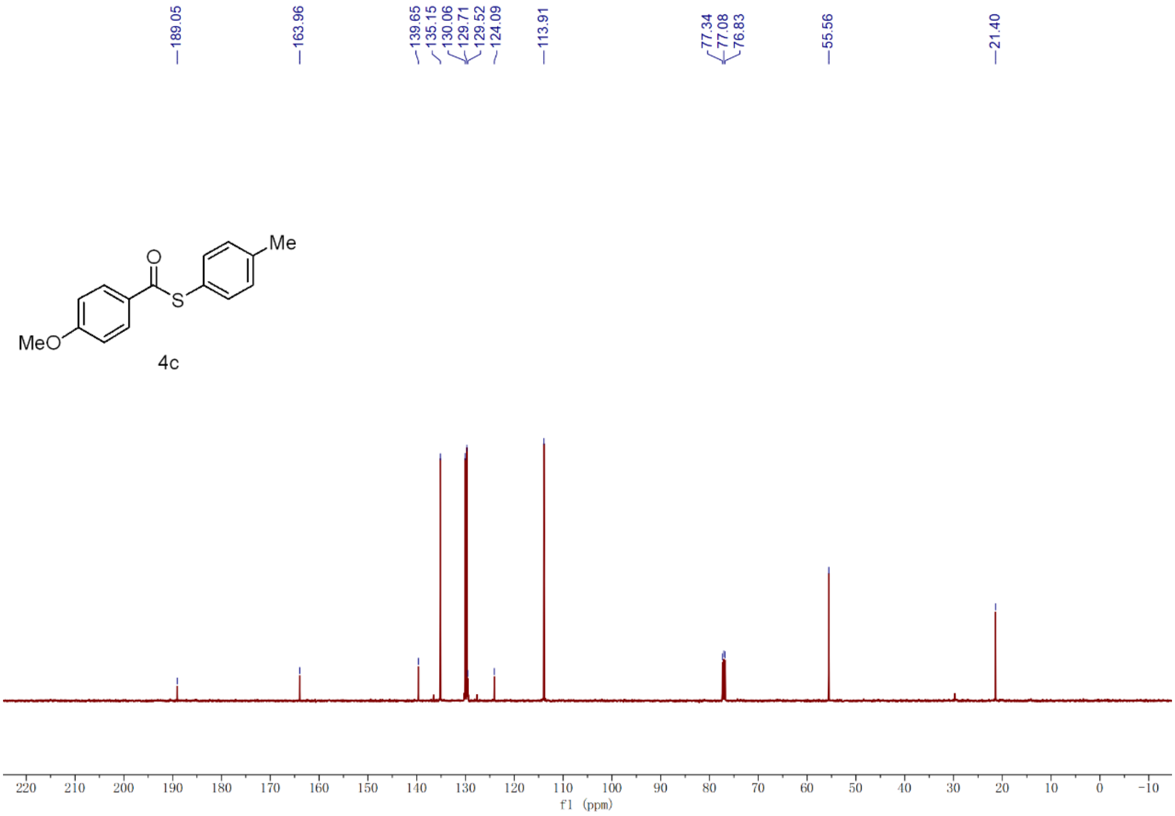

Supplementary Figure 64a | ^{1}H NMR (400 MHz, $CDCl_3$) of *S*-(p-tolyl) 4-acetamidobenzothioate (**4d**)

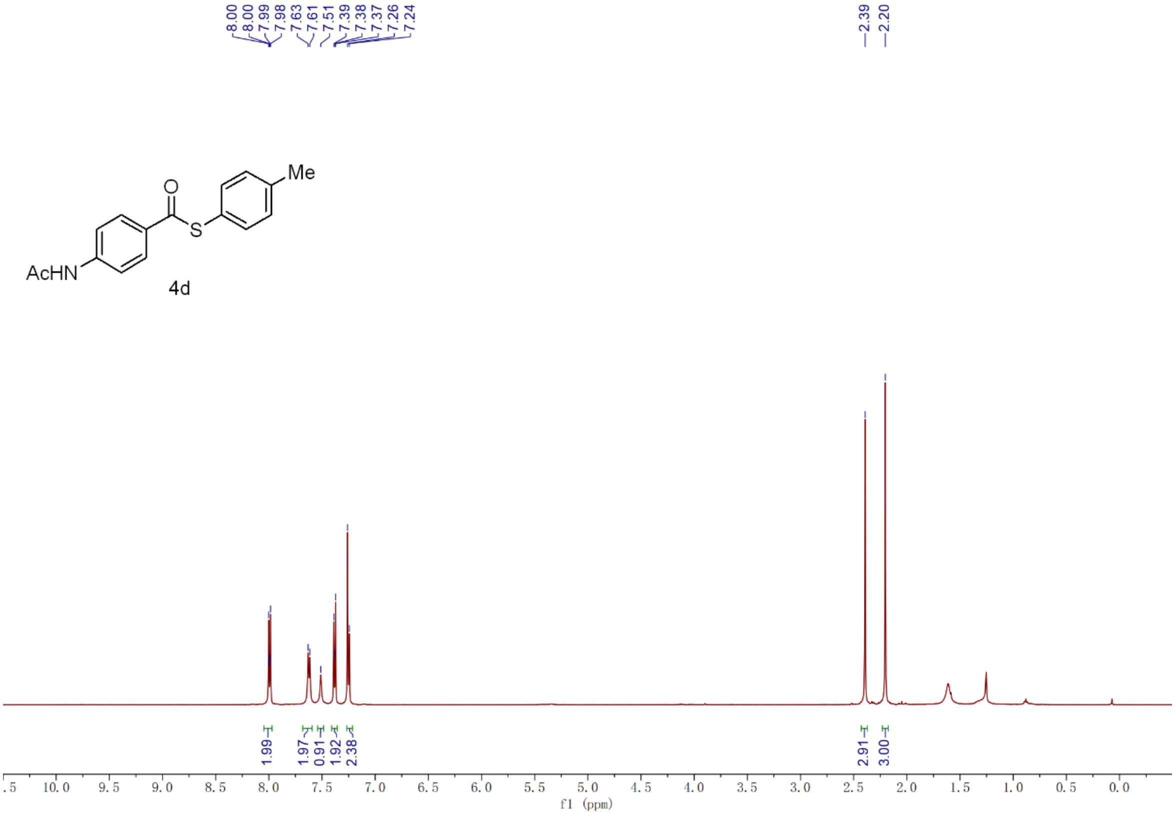

Supplementary Figure 64b | ^{13}C NMR (126 MHz, $CDCl_3$) of *S*-(p-tolyl) 4-acetamidobenzothioate (**4d**)

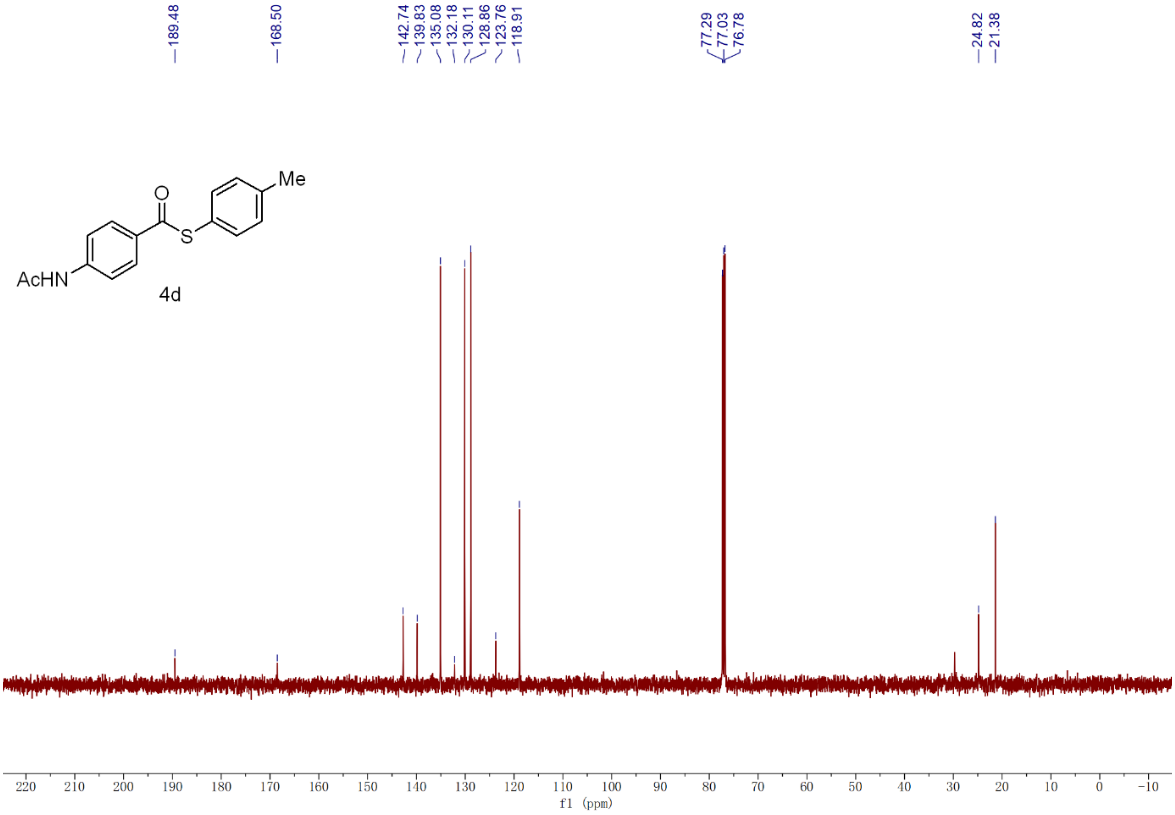

Supplementary Figure 65a | ^{1}H NMR (500 MHz, $CDCl_3$) of 4-((p-tolylthio)carbonyl)phenyl acetate (**4e**)

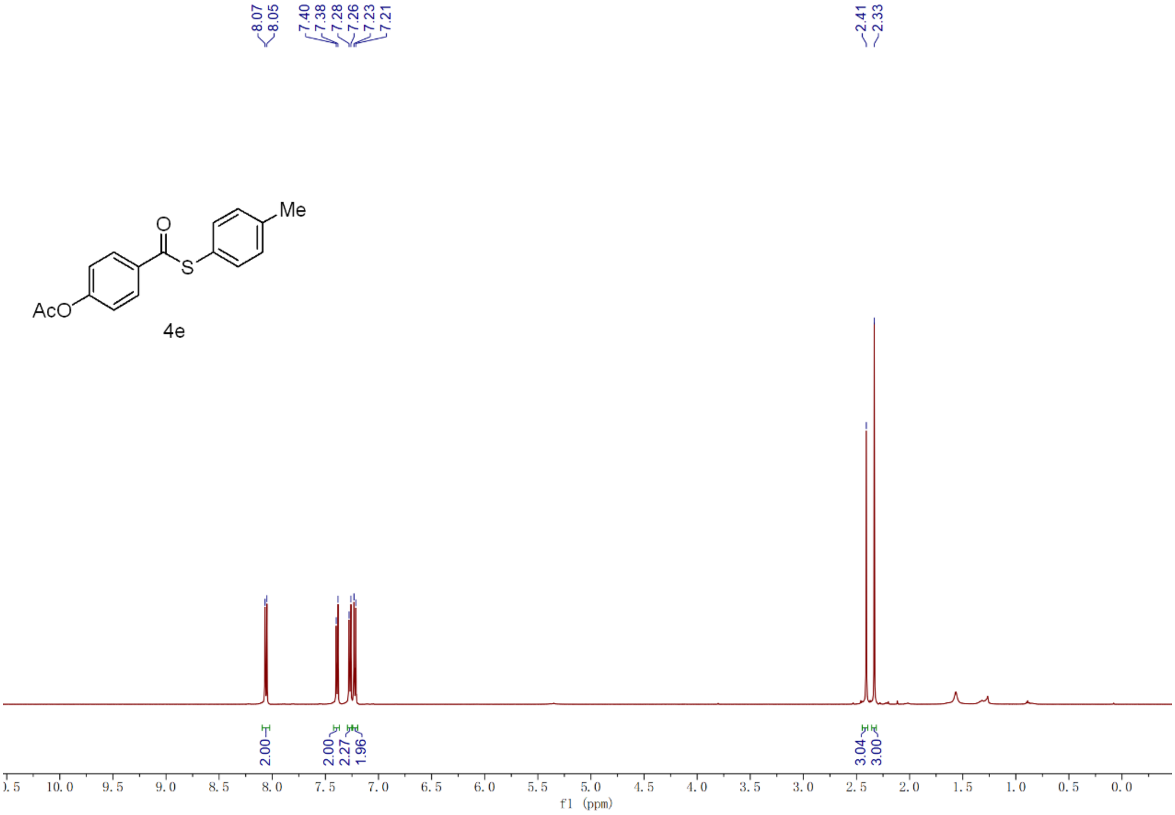

Supplementary Figure 65b | ^{13}C NMR (126 MHz, $CDCl_3$) of 4-((p-tolylthio)carbonyl)phenyl acetate (**4e**)

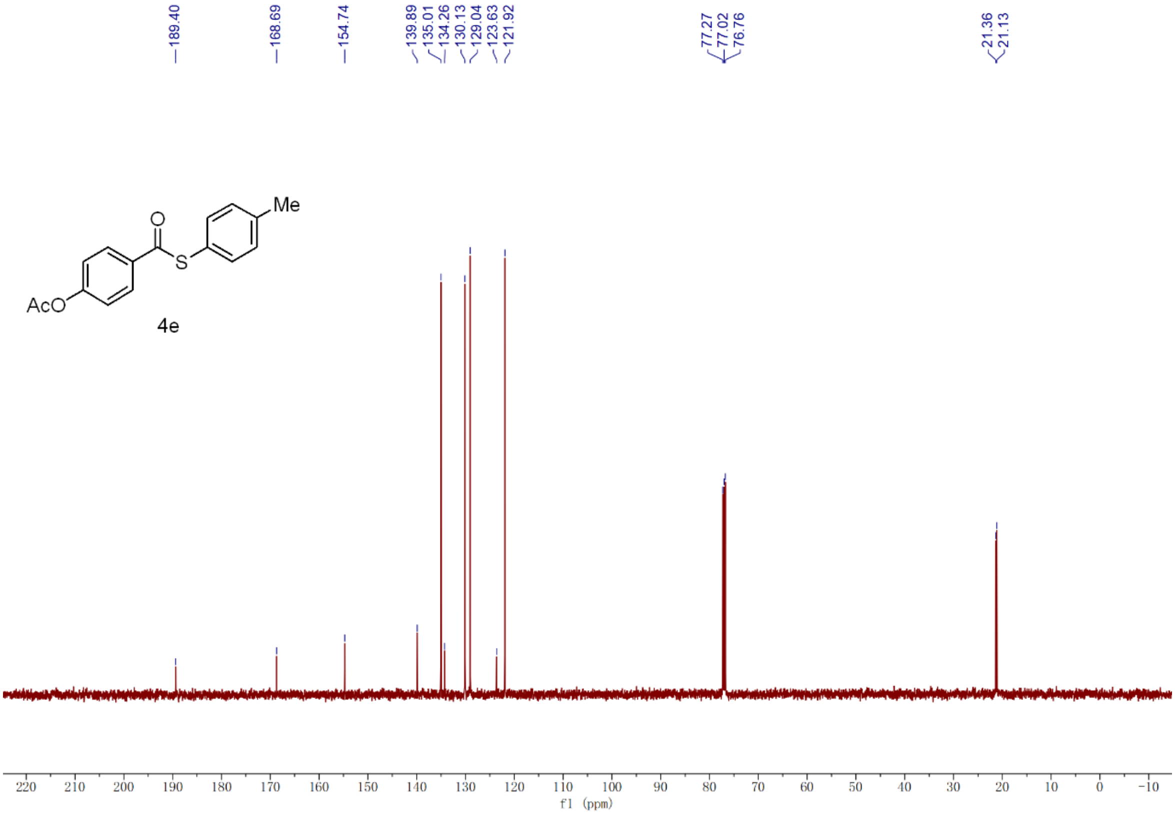

Supplementary Figure 66a | ^{1}H NMR (500 MHz, $CDCl_3$) of *S*-(p-tolyl) 2-methylbenzothioate (**4f**)

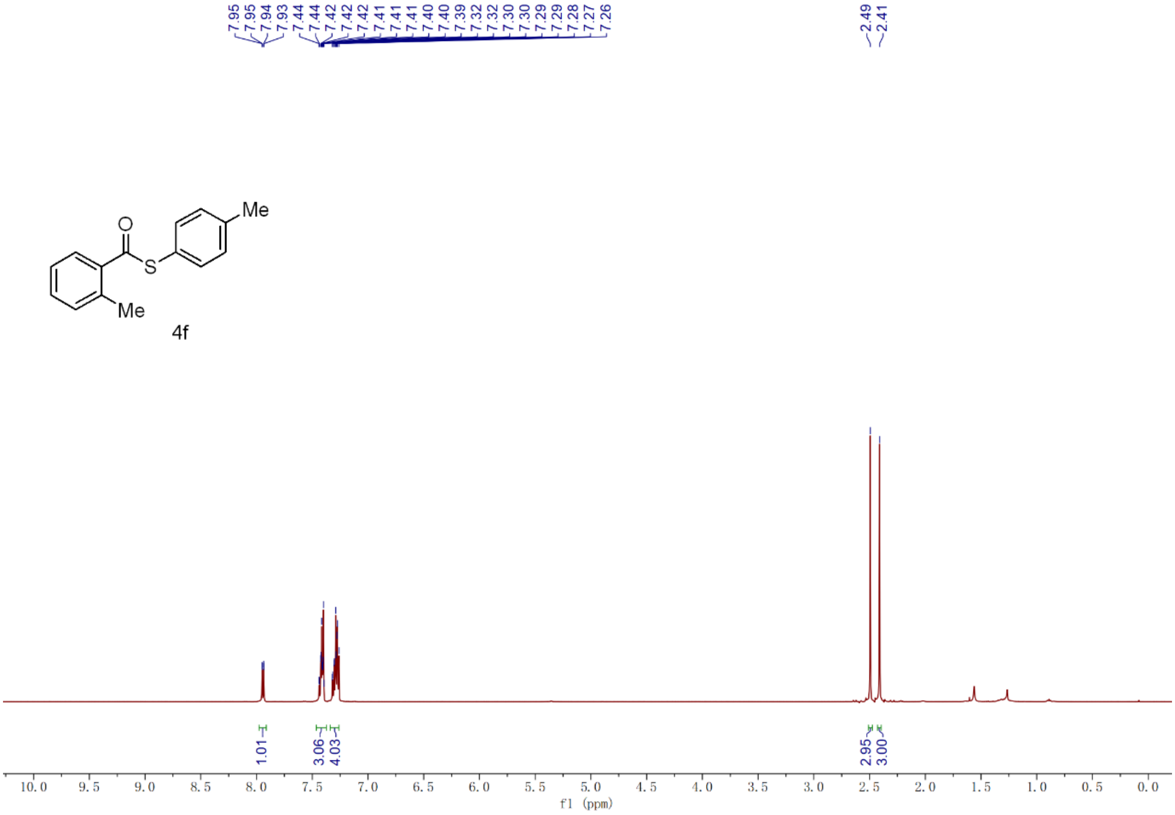

Supplementary Figure 66b | ^{13}C NMR (126 MHz, $CDCl_3$) of *S*-(p-tolyl) 2-methylbenzothioate (**4f**)

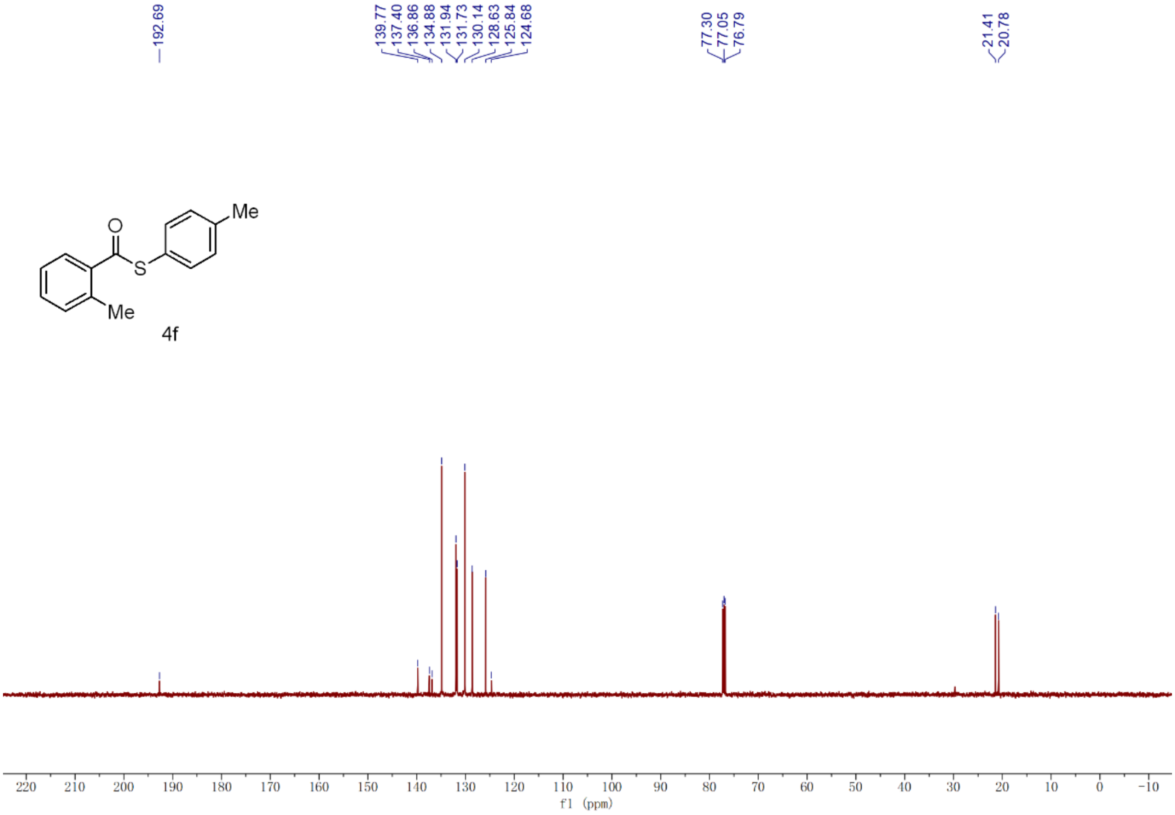

Supplementary Figure 67a | ^{1}H NMR (500 MHz, $CDCl_3$) of *S*-(p-tolyl) 3-methylbenzothioate (**4g**)

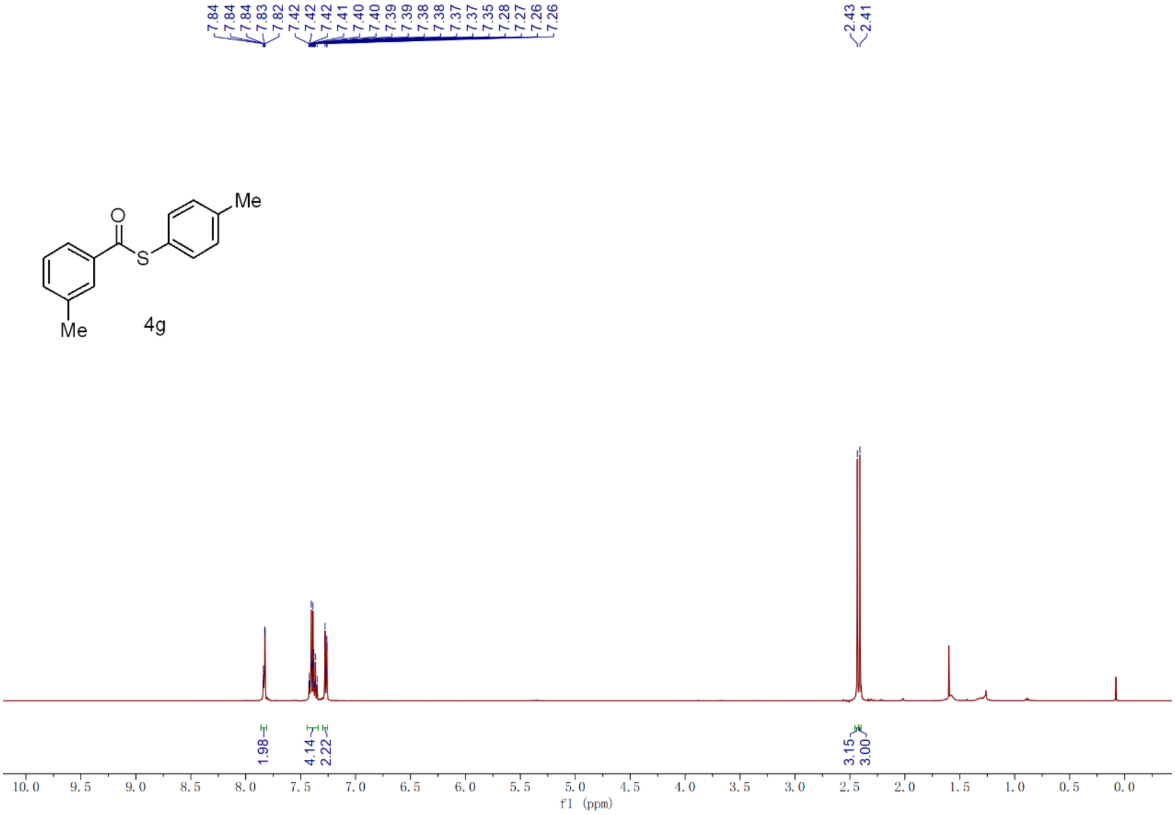

Supplementary Figure 67b | ^{13}C NMR (126 MHz, $CDCl_3$) of *S*-(p-tolyl) 3-methylbenzothioate (**4g**)

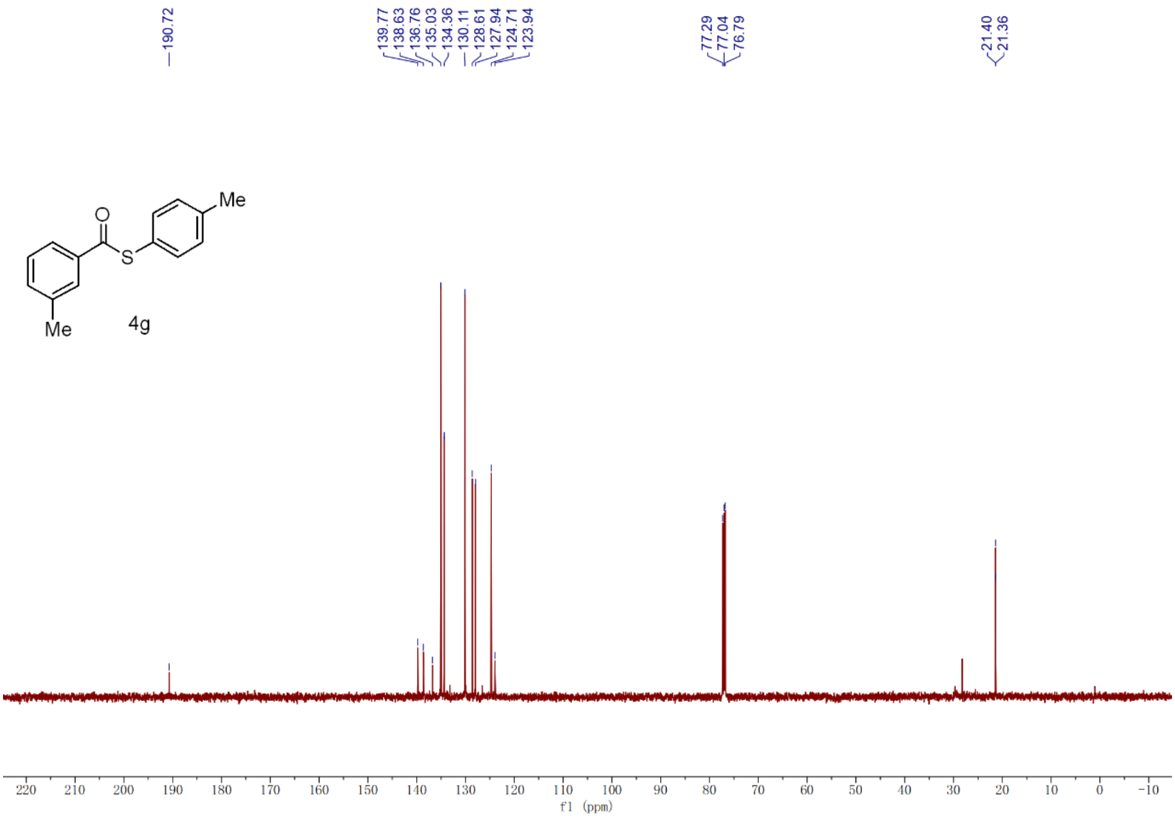

Supplementary Figure 68a | ^{1}H NMR (500 MHz, $CDCl_3$) of *S*-(p-tolyl) 4-fluorobenzothioate (**4h**)

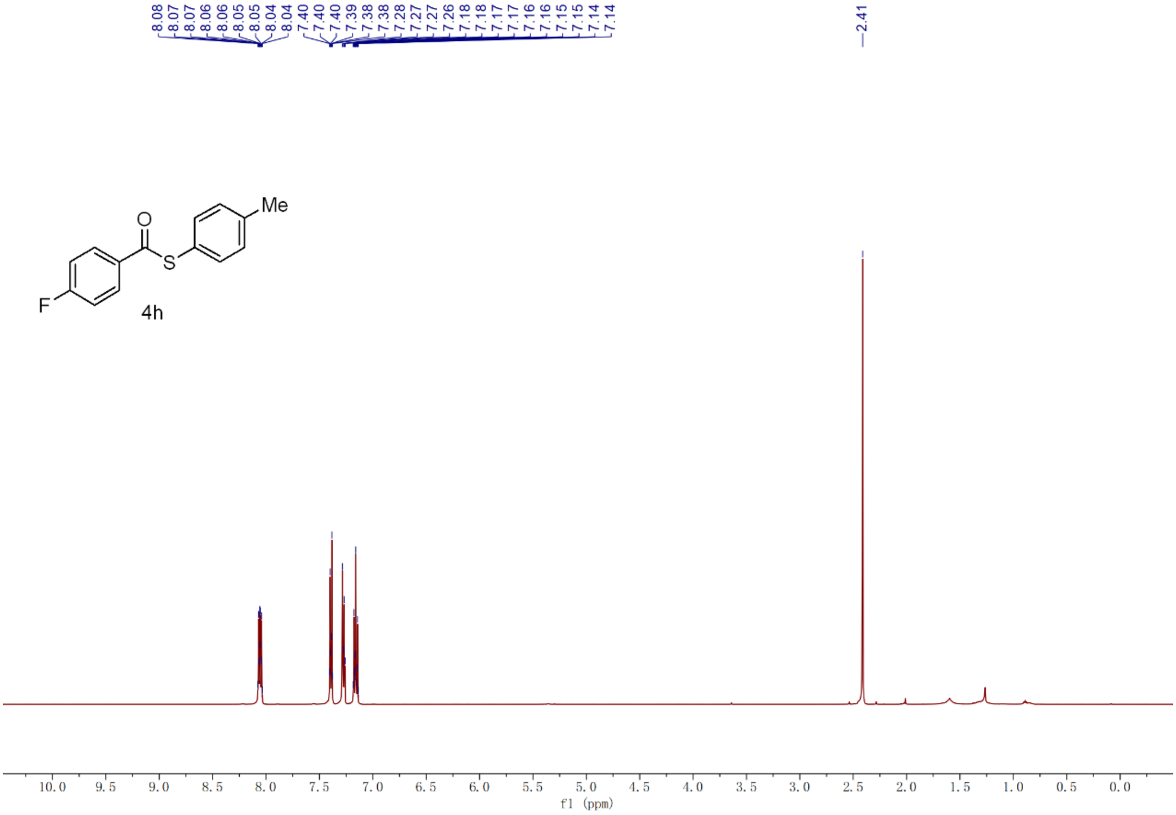

Supplementary Figure 68b | ^{13}C NMR (126 MHz, $CDCl_3$) of *S*-(p-tolyl) 4-fluorobenzothioate (**4h**)

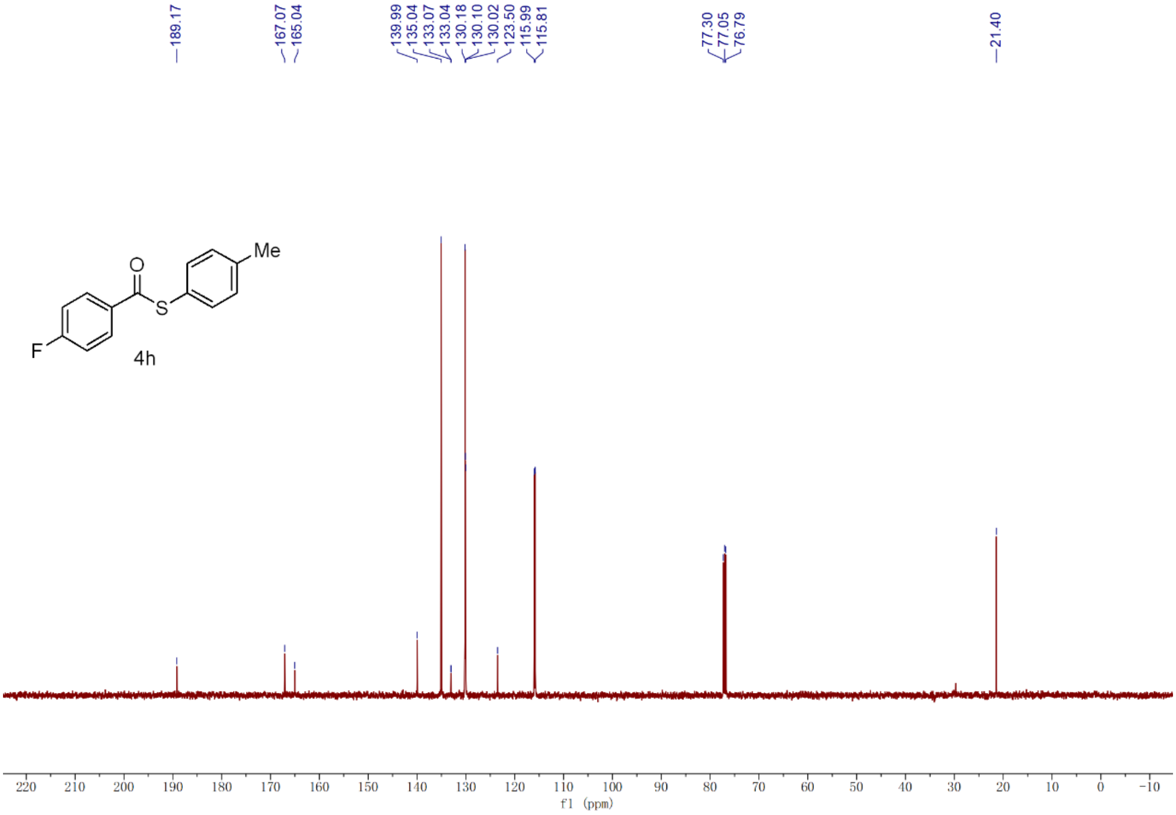

Supplementary Figure 68c | ^{19}F NMR (377 MHz, $CDCl_3$) of *S*-(p-tolyl) 4-fluorobenzothioate (**4h**)

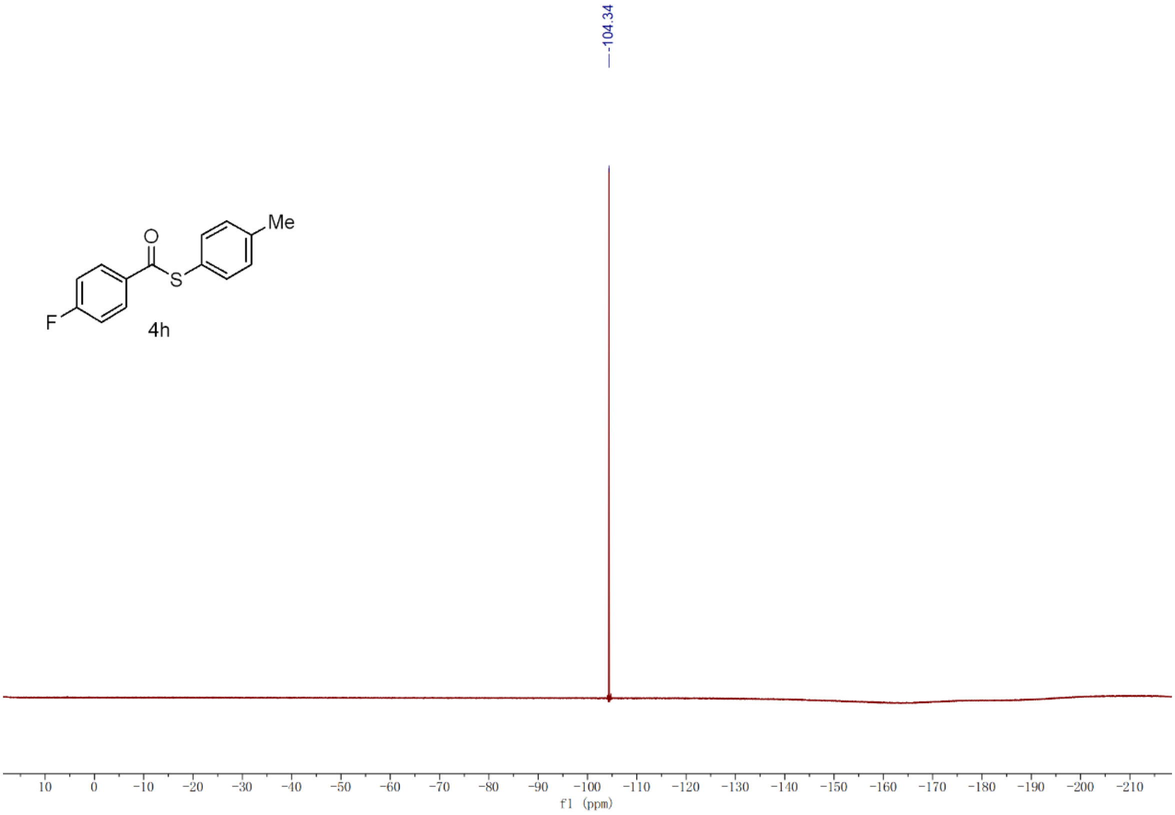

Supplementary Figure 69a | ^{1}H NMR (500 MHz, $CDCl_3$) of *S*-(p-tolyl) 4-(trifluoromethyl)benzothioate (**4i**)

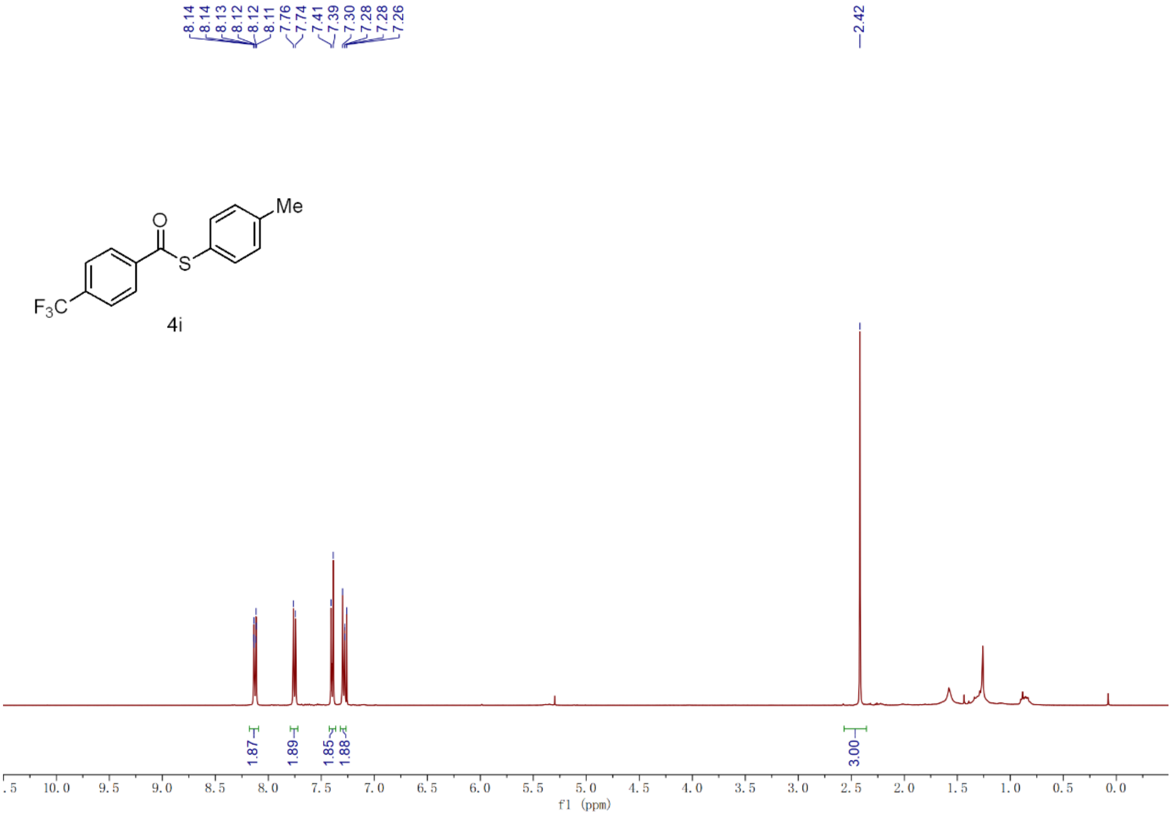

Supplementary Figure 69b | ^{13}C NMR (126 MHz, $CDCl_3$) of *S*-(p-tolyl) 4-(trifluoromethyl)benzothioate **(4i)**

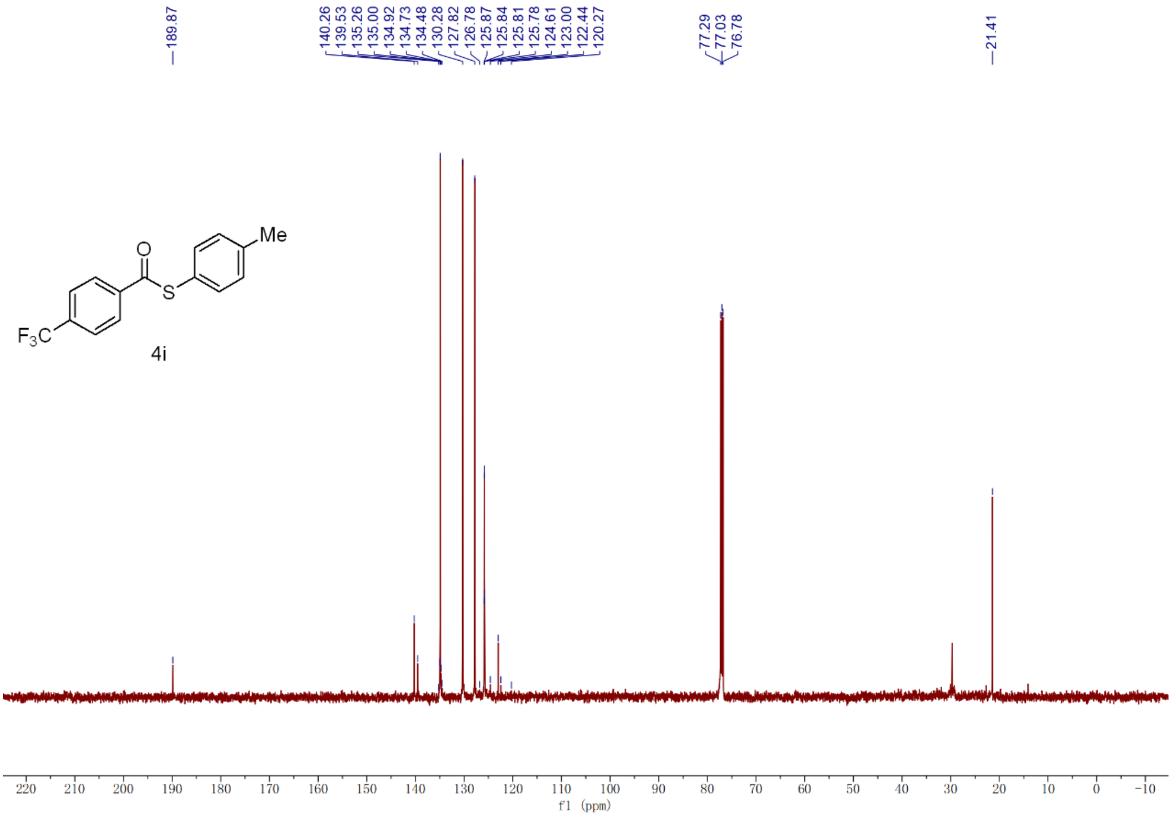

Supplementary Figure 69c | ^{19}F NMR (377 MHz, $CDCl_3$) of *S*-(p-tolyl) 4-(trifluoromethyl)benzothioate **(4i)**

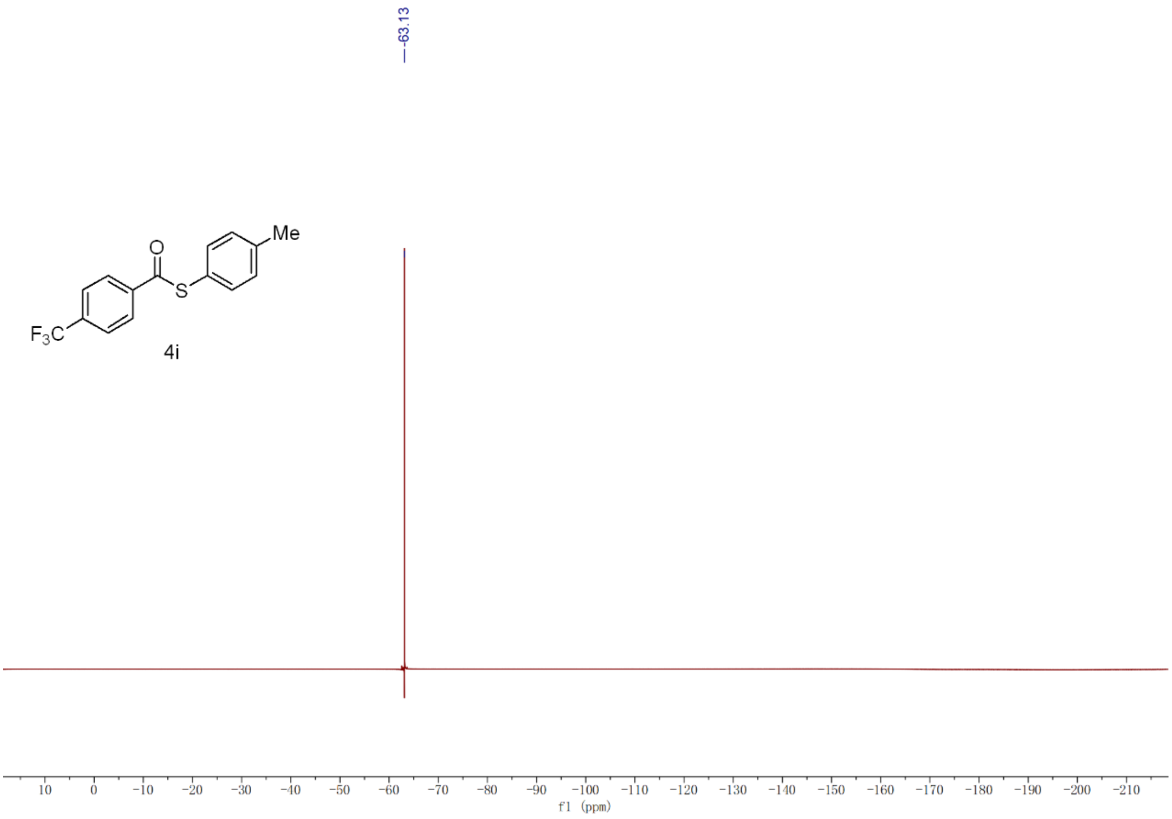

Supplementary Figure 70a | ^{1}H NMR (400 MHz, $CDCl_3$) of Methyl 3-((p-tolylthio)carbonyl)benzoate (**4j**)

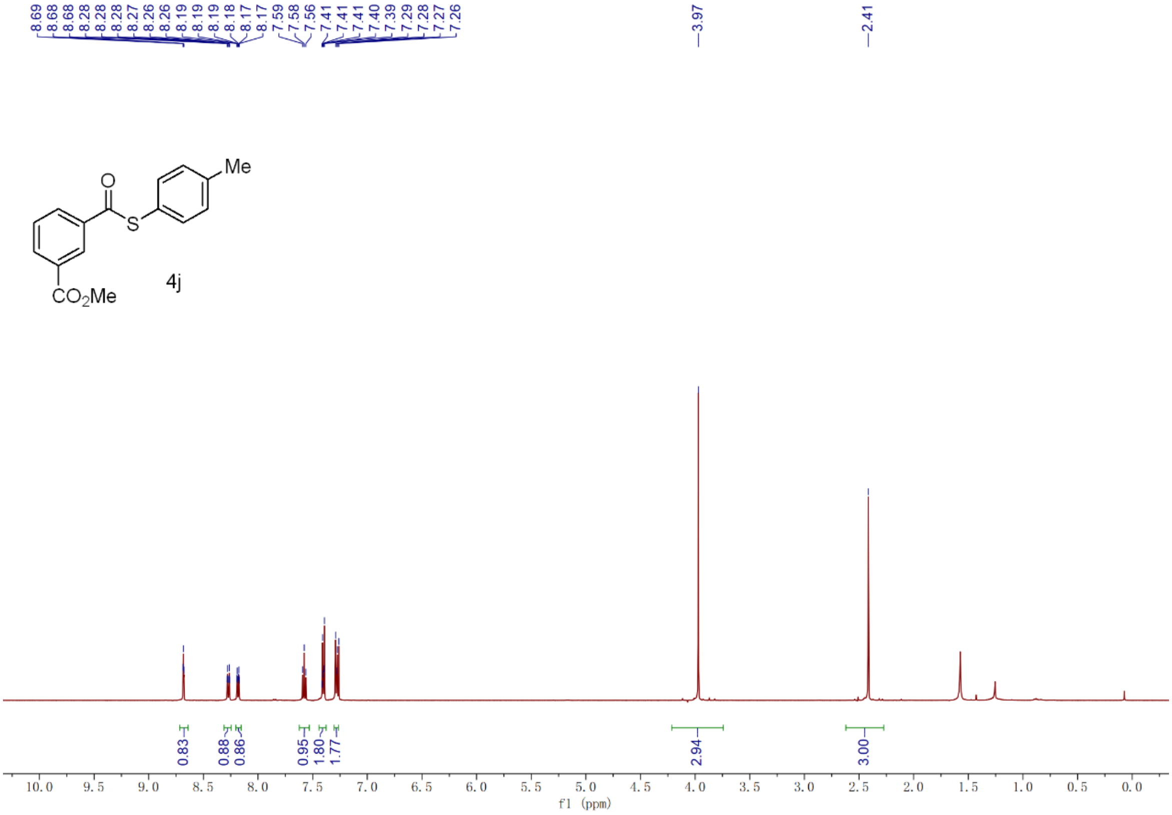

Supplementary Figure 70b | ^{13}C NMR (126 MHz, $CDCl_3$) of Methyl 3-((p-tolylthio)carbonyl)benzoate (**4j**)

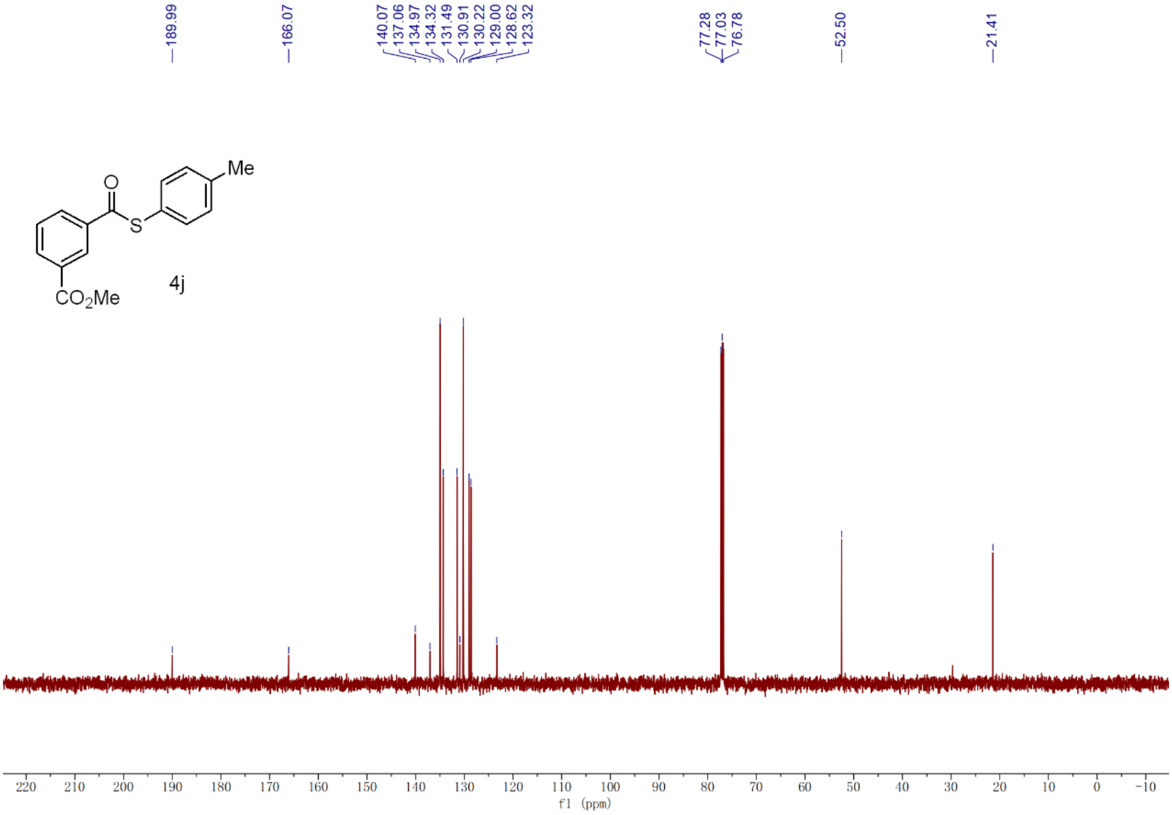

Supplementary Figure 71a | ^{1}H NMR (400 MHz, $CDCl_3$) of *S*-(p-tolyl) 2,2-difluorobenzo[d][1,3]dioxole-5-carbothioate (**4k**)

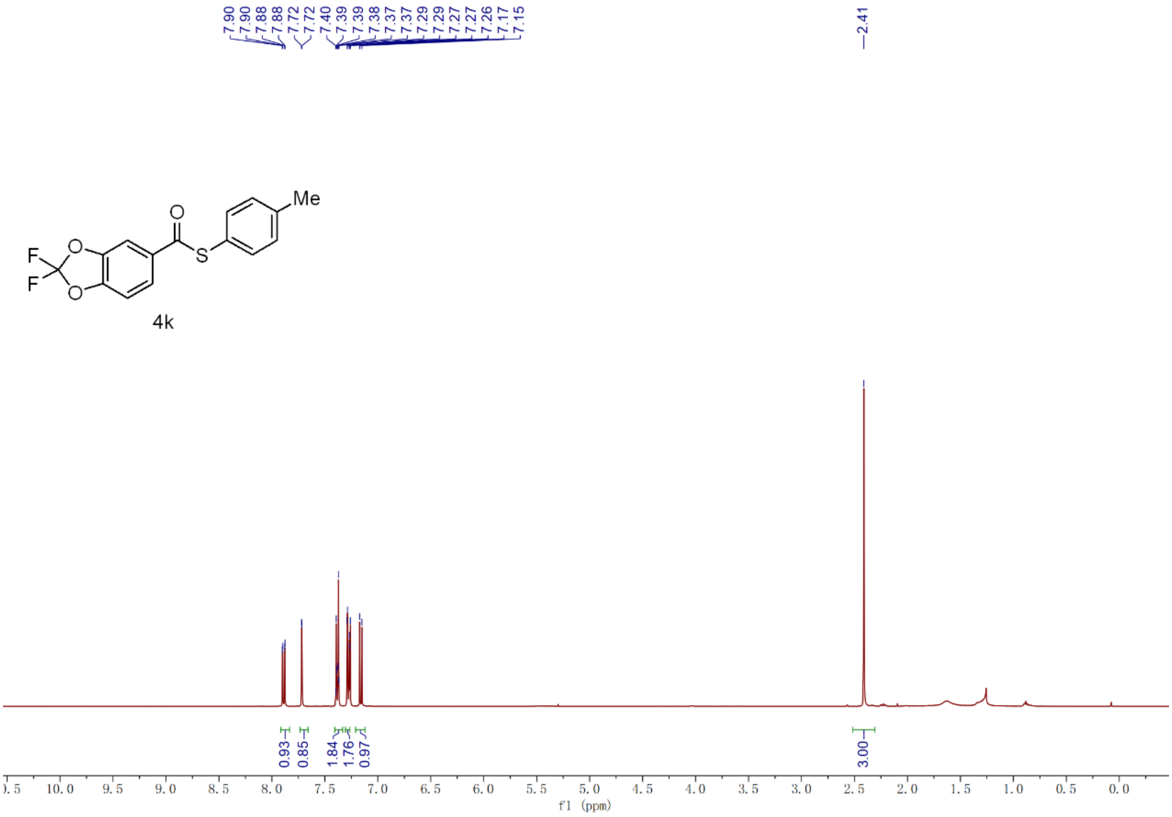

Supplementary Figure 71b | ^{13}C NMR (126 MHz, $CDCl_3$) of *S*-(p-tolyl) 2,2-difluorobenzo[d][1,3]dioxole-5-carbothioate (**4k**)

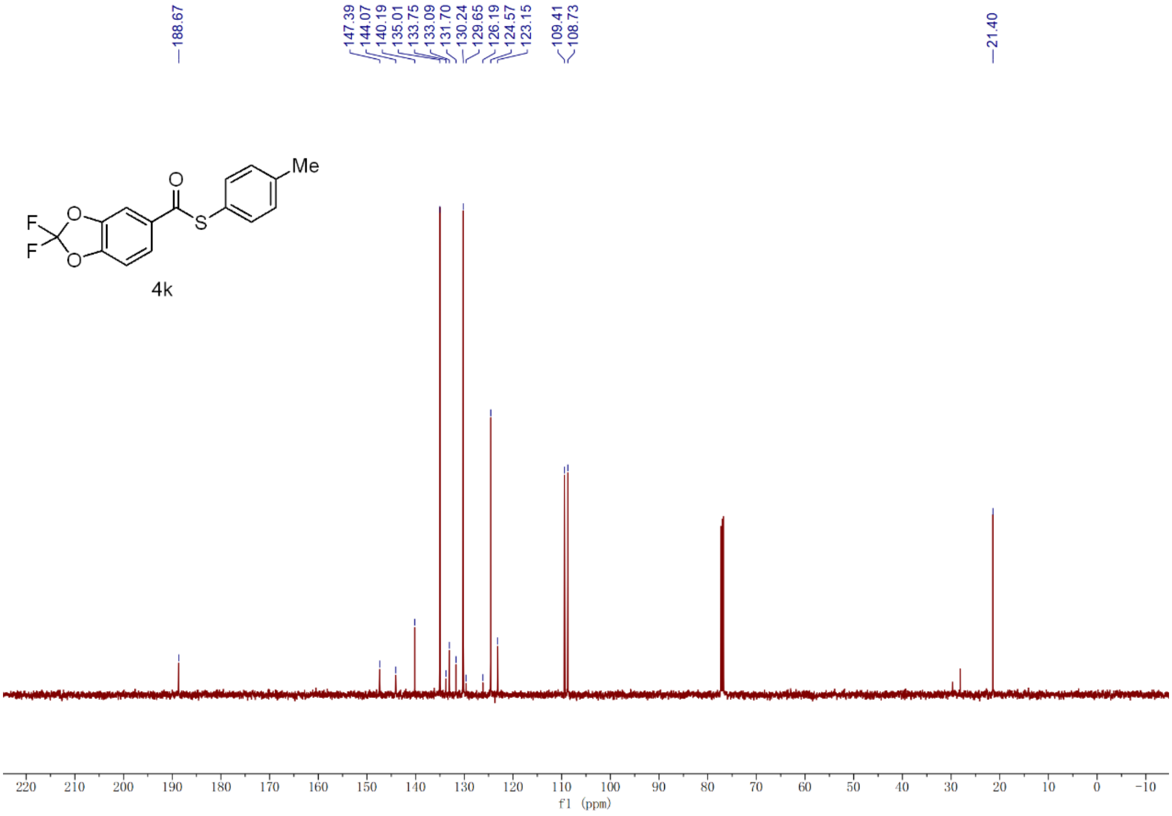

Supplementary Figure 71c | ^{19}F NMR (377 MHz, $CDCl_3$) of *S*-(p-tolyl) 2,2-difluorobenzo[d][1,3]dioxole-5-carbothioate (**4k**)

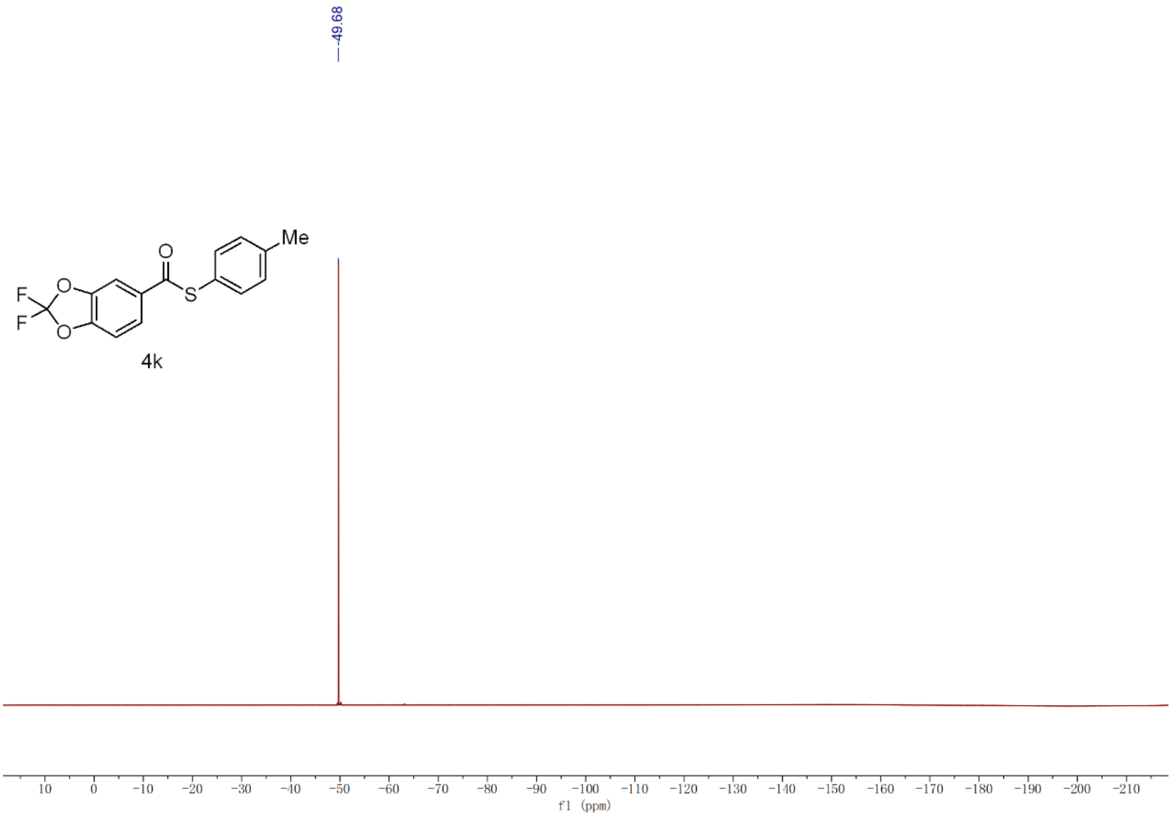

Supplementary Figure 72a | ^{1}H NMR (500 MHz, $CDCl_3$) of *S*-(p-tolyl) benzo[b]thiophene-2-carbothioate (**4l**)

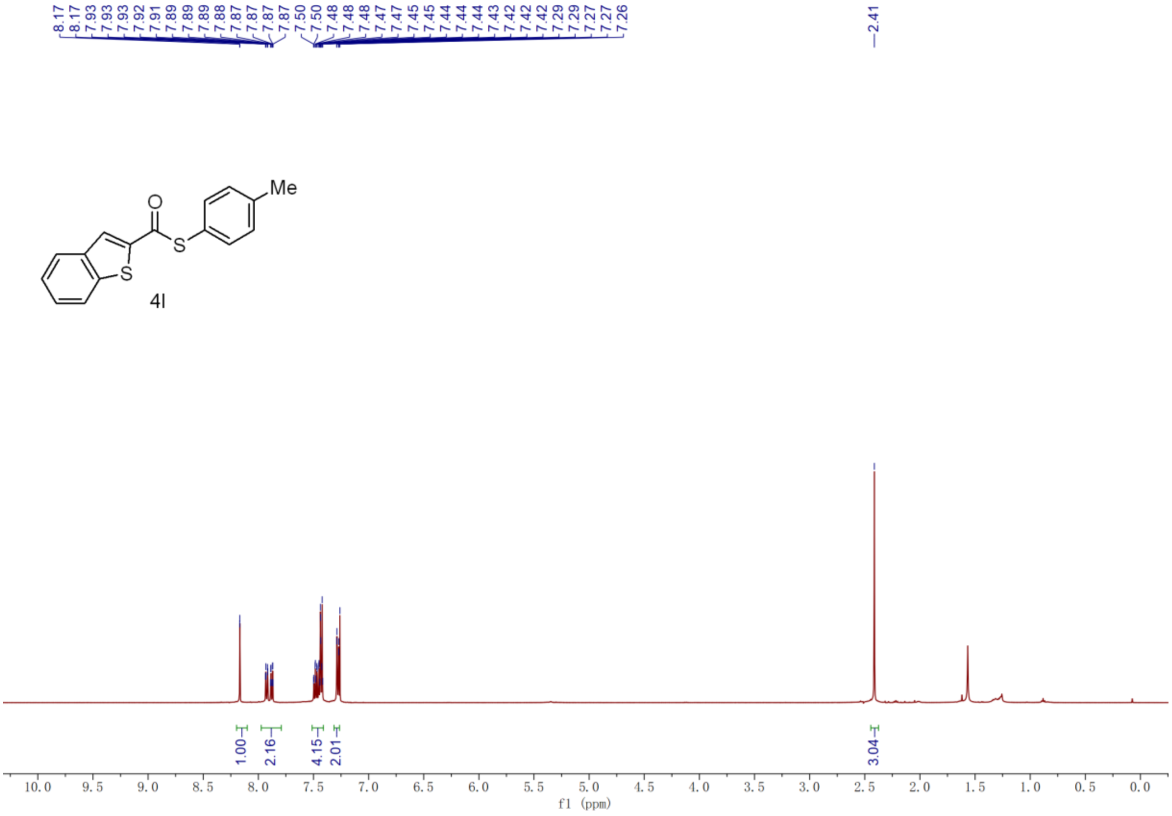

Supplementary Figure 72b | ^{13}C NMR (126 MHz, $CDCl_3$) of *S*-(p-tolyl) benzo[b]thiophene-2-carbothioate (**4l**)

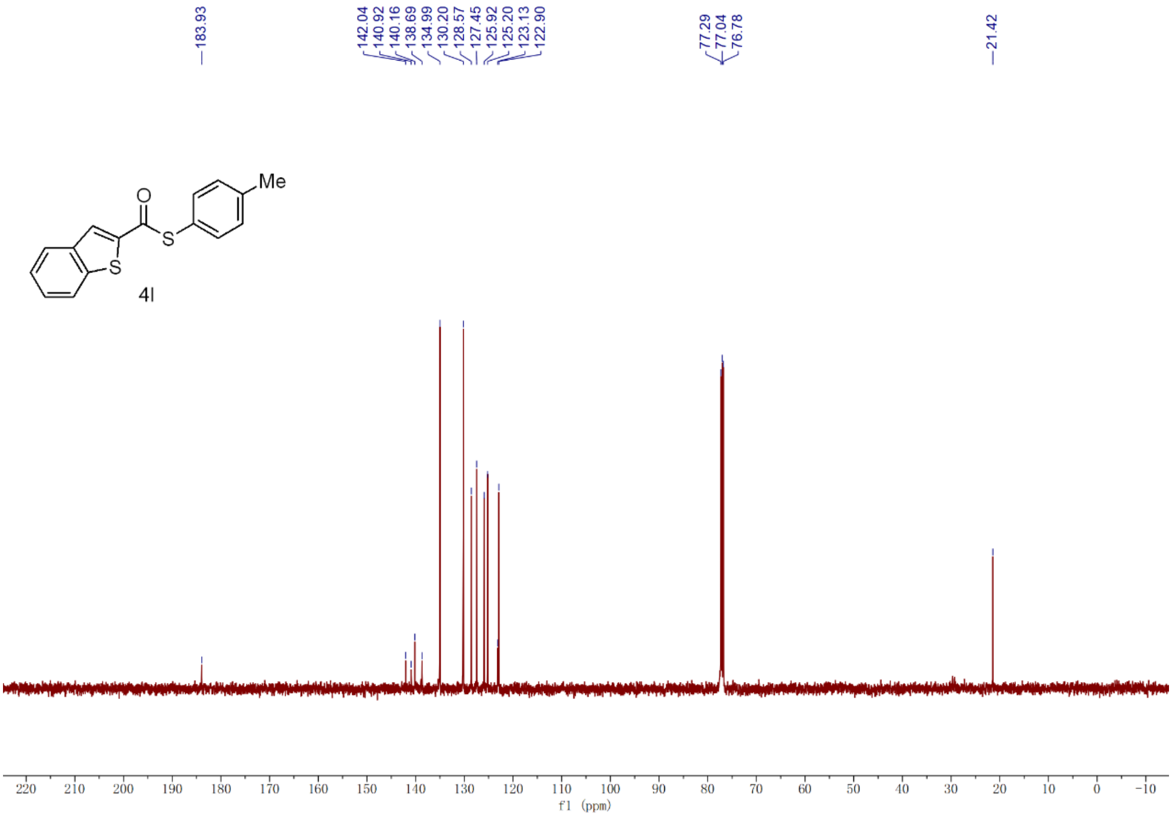

Supplementary Figure 73a | ^{1}H NMR (500 MHz, $CDCl_3$) of *S*-(p-tolyl) furan-2-carbothioate (**4m**)

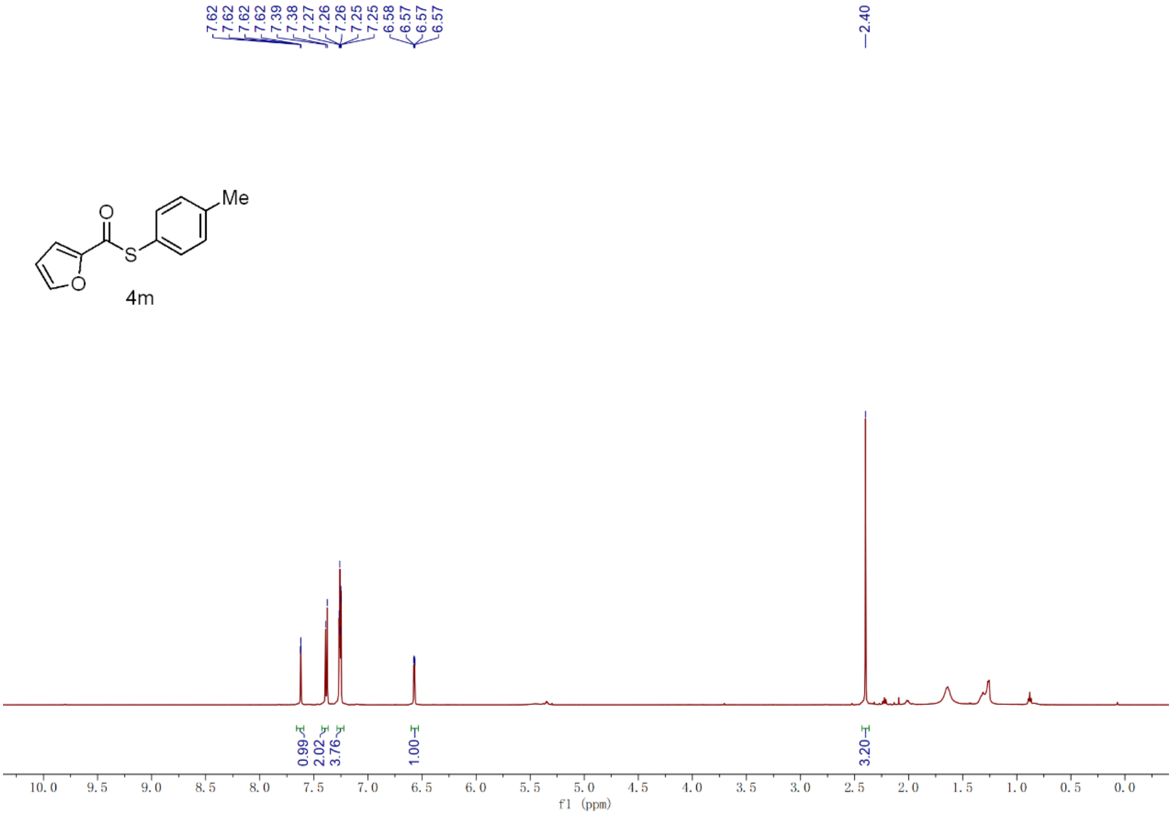

Supplementary Figure 73b | ^{13}C NMR (126 MHz, $CDCl_3$) of *S*-(p-tolyl) furan-2-carbothioate (**4m**)

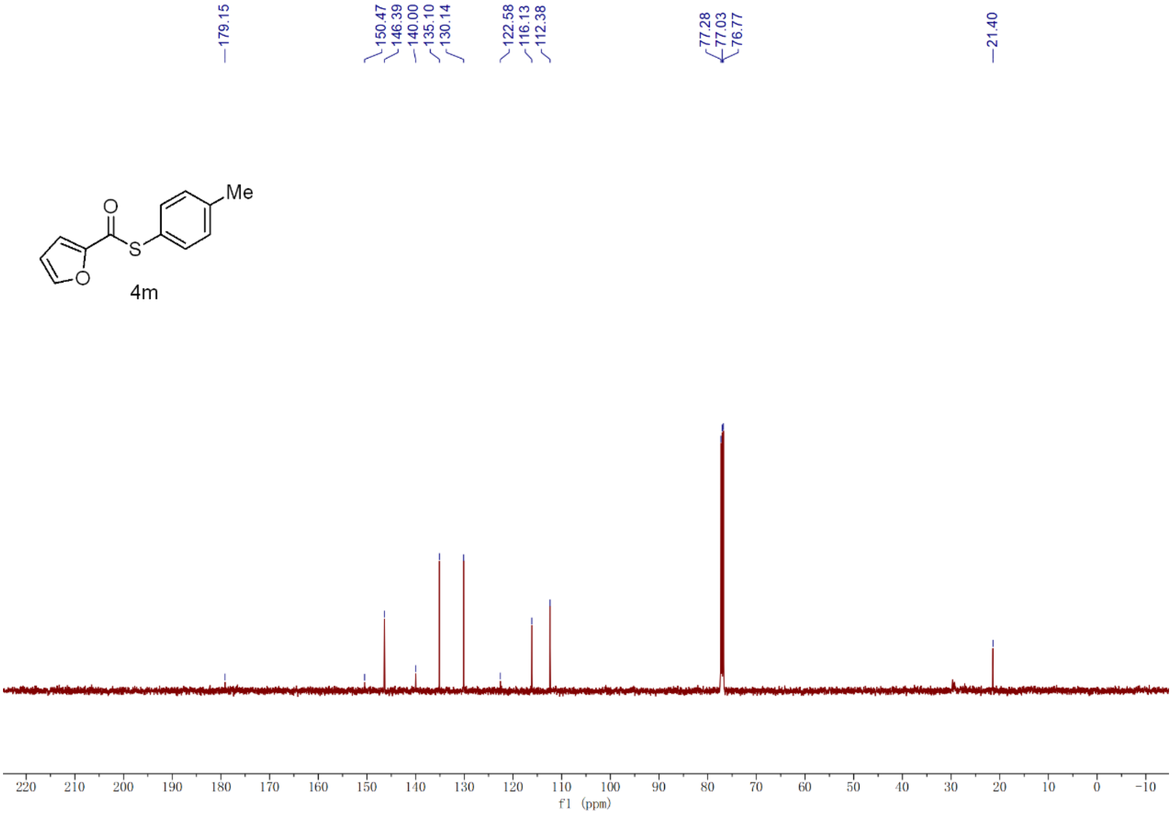

Supplementary Figure 74a | ^{1}H NMR (500 MHz, $CDCl_3$) of *S*-(p-tolyl) hexanethioate (**4n**)

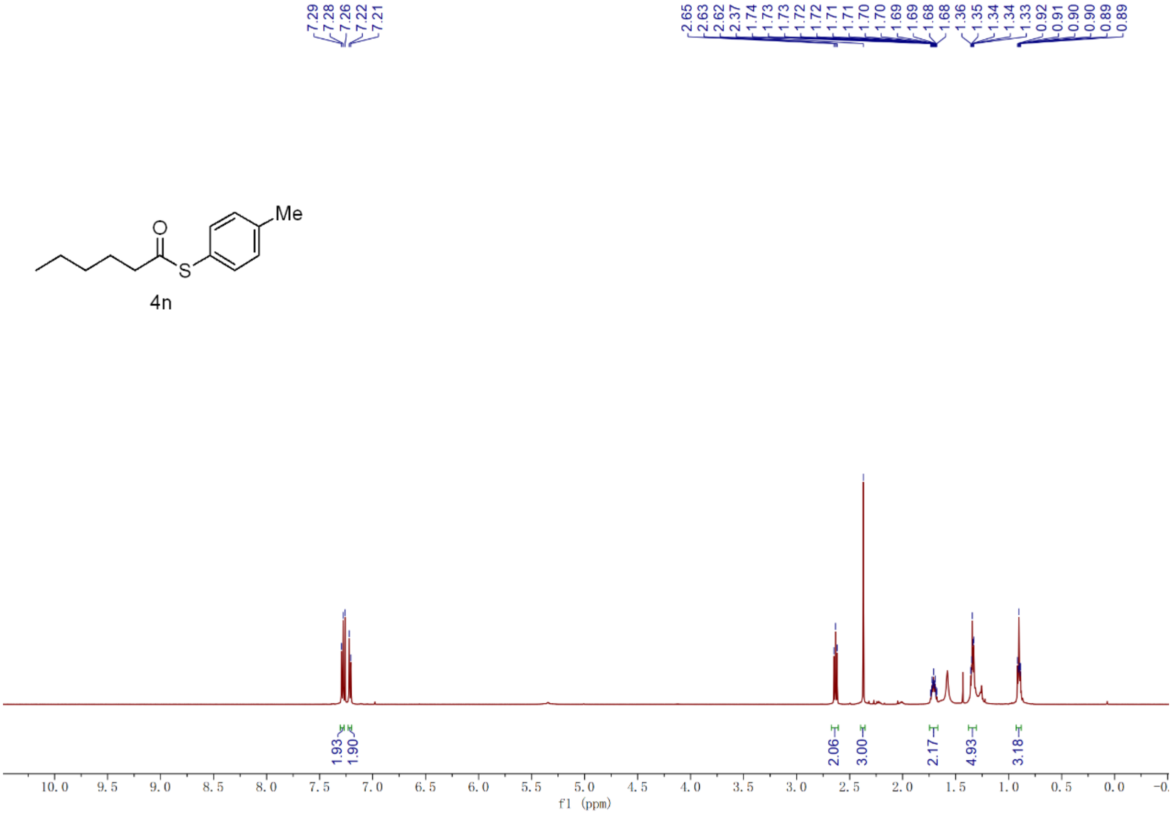

Supplementary Figure 74b | ^{13}C NMR (126 MHz, $CDCl_3$) of *S*-(p-tolyl) hexanethioate (**4n**)

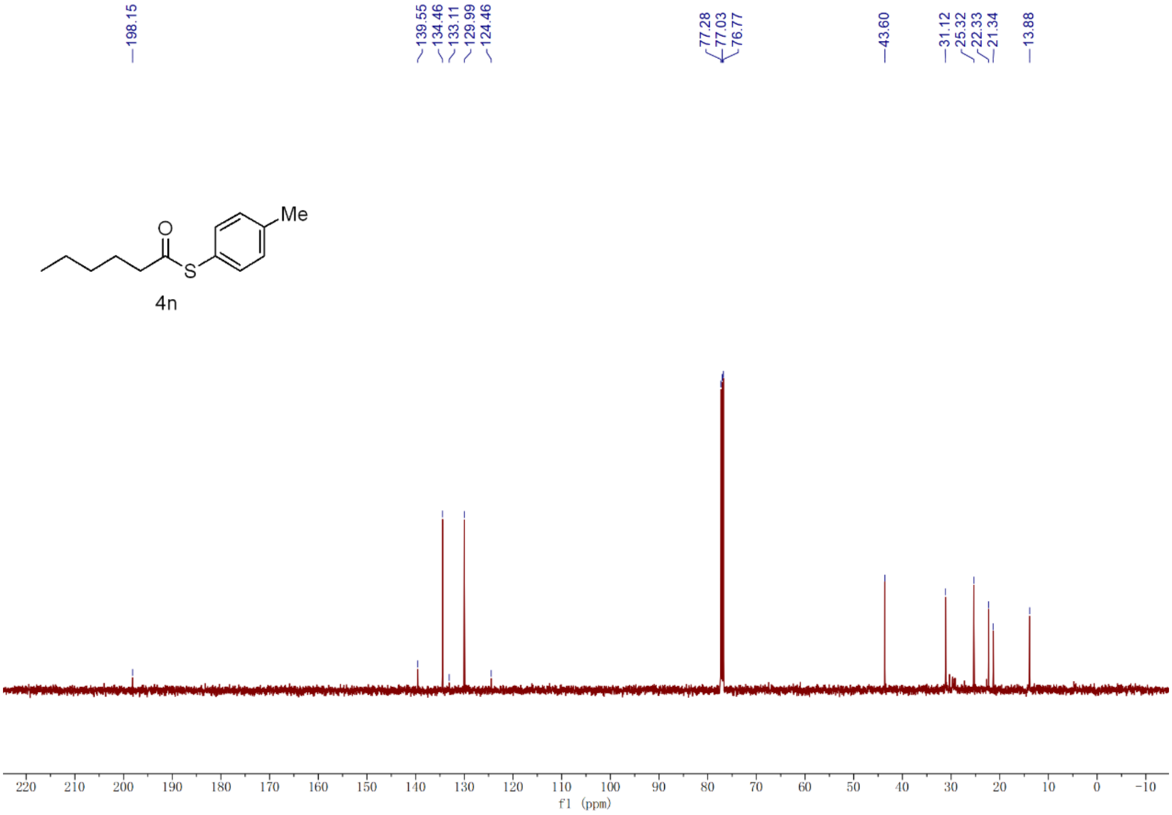

Supplementary Figure 75a | ^{1}H NMR (400 MHz, $CDCl_3$) of *S*-(p-tolyl) cyclopropanecarbothioate (**4o**)

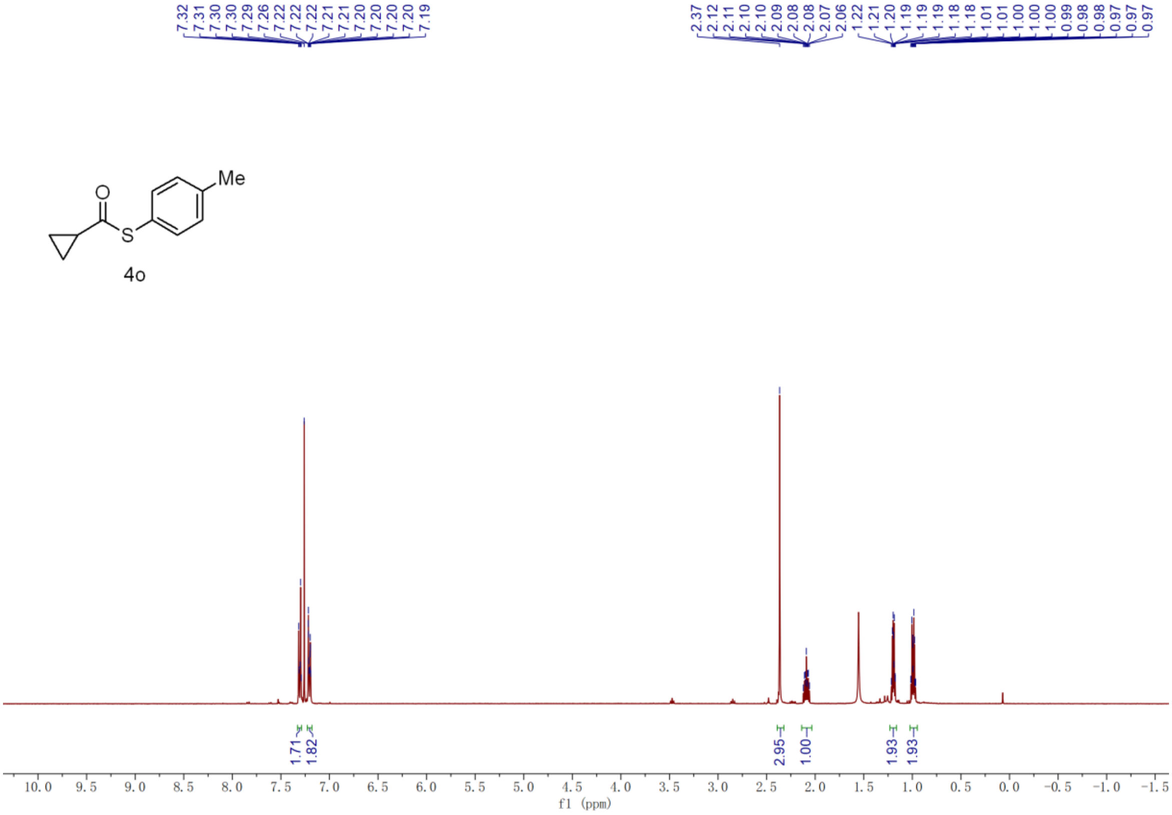

Supplementary Figure 75b | ^{13}C NMR (126 MHz, $CDCl_3$) of *S*-(p-tolyl) cyclopropanecarbothioate (**4o**)

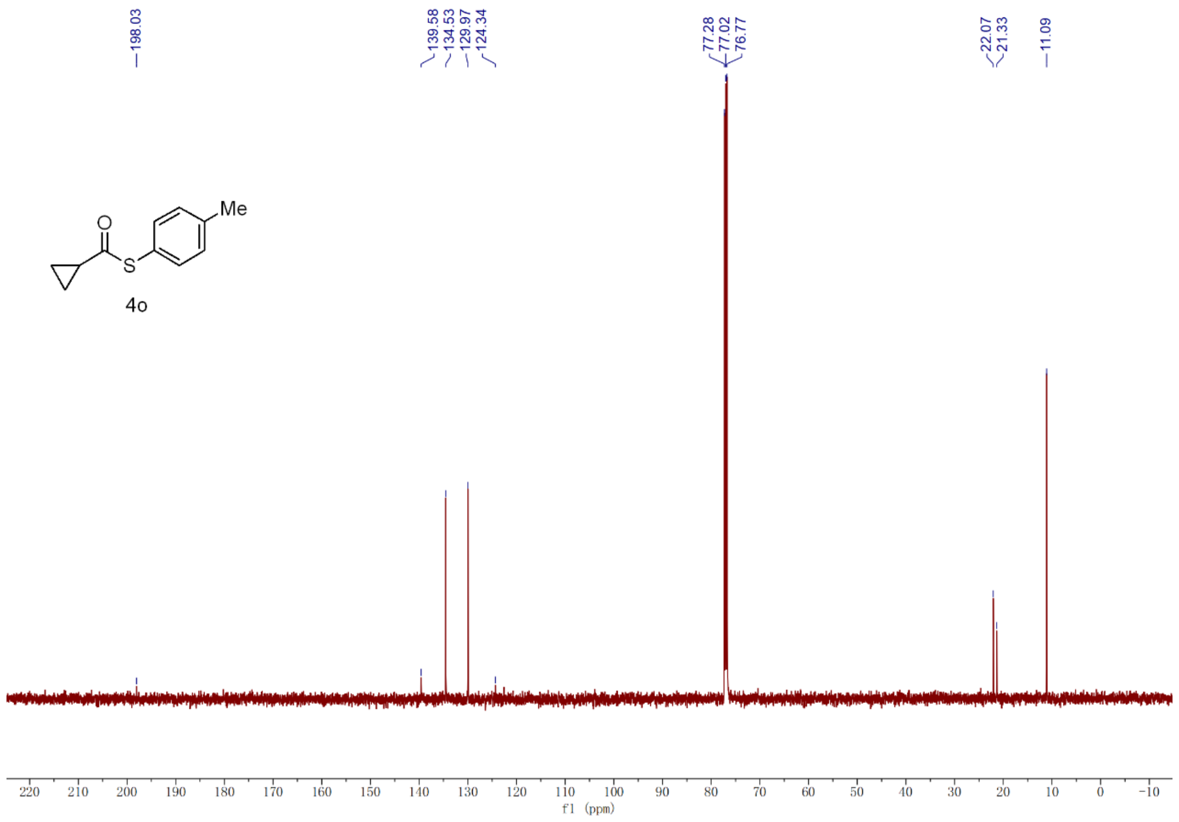

Supplementary Figure 76a | ^{1}H NMR (500 MHz, $CDCl_3$) of *S*-phenyl 2-methylbenzothioate (**4p**)

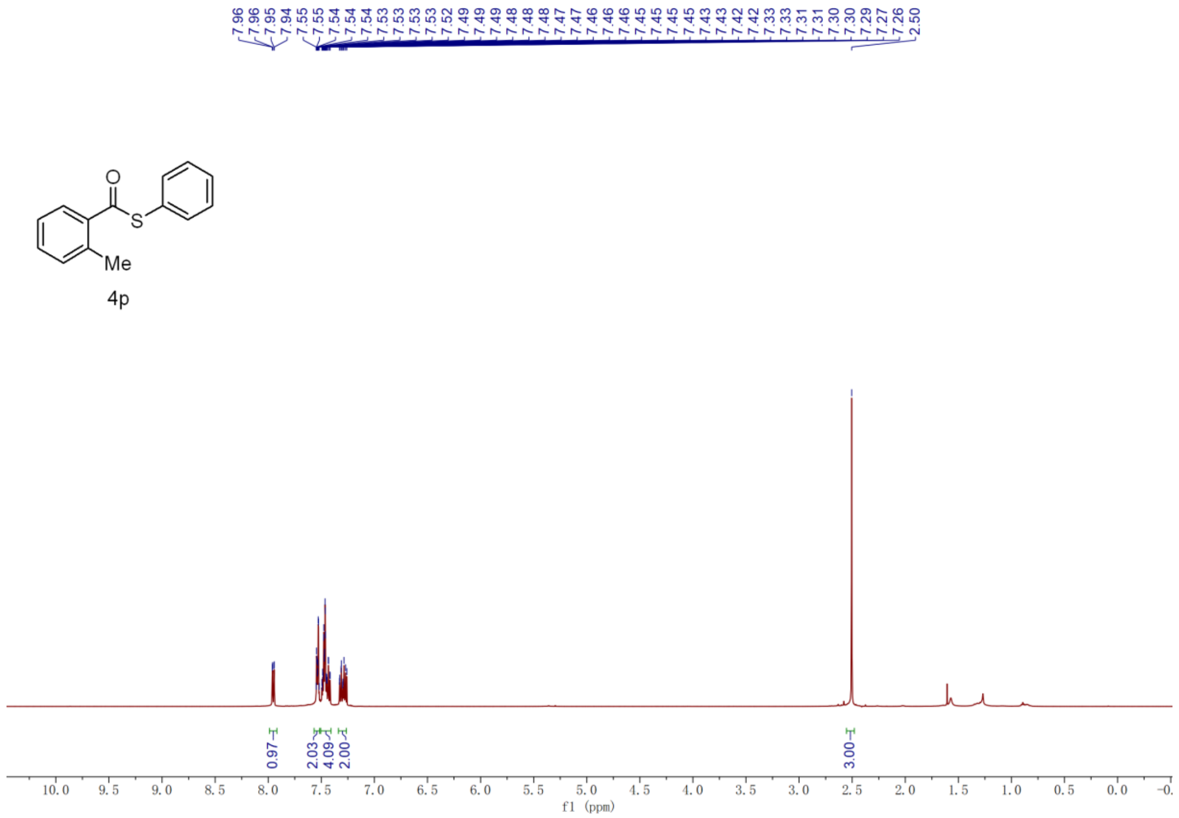

Supplementary Figure 76b | ^{13}C NMR (126 MHz, $CDCl_3$) of *S*-phenyl 2-methylbenzothioate (**4p**)

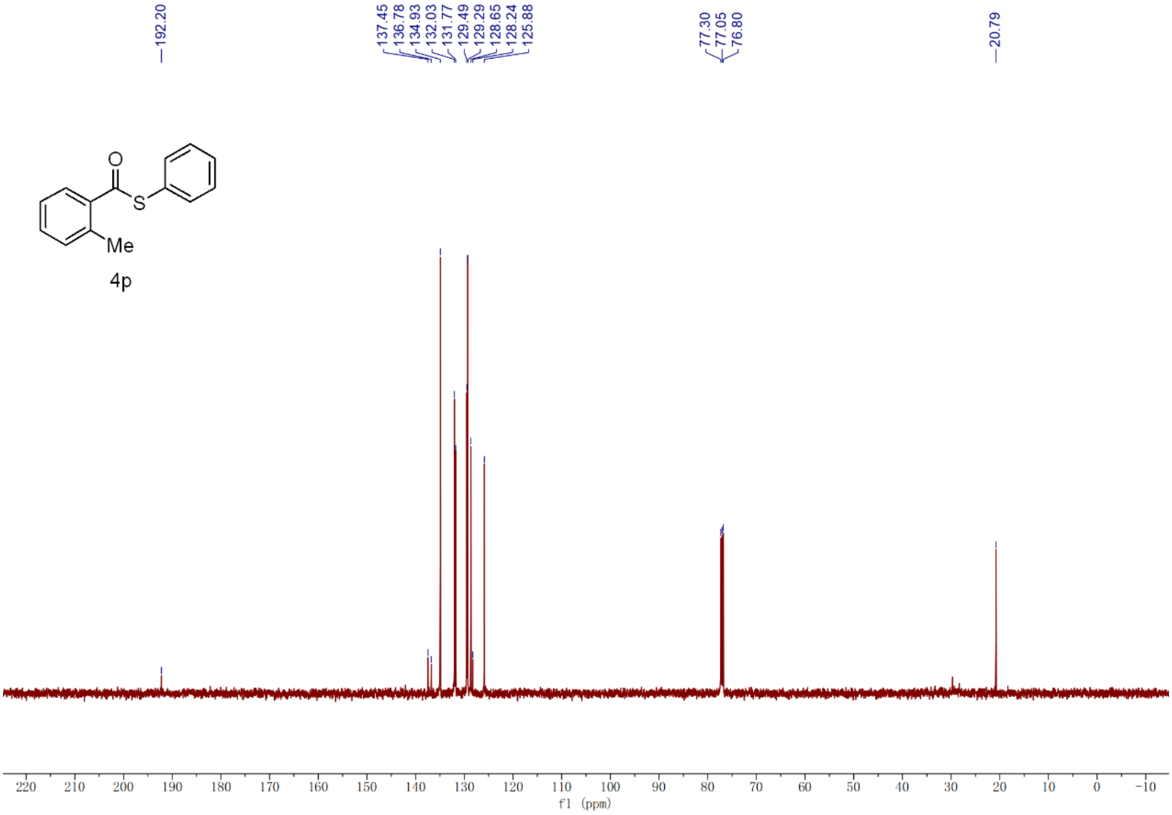

Supplementary Figure 77a | ^{1}H NMR (500 MHz, $CDCl_3$) of *S*-(2-methoxyphenyl) 2-methylbenzothioate (**4q**)

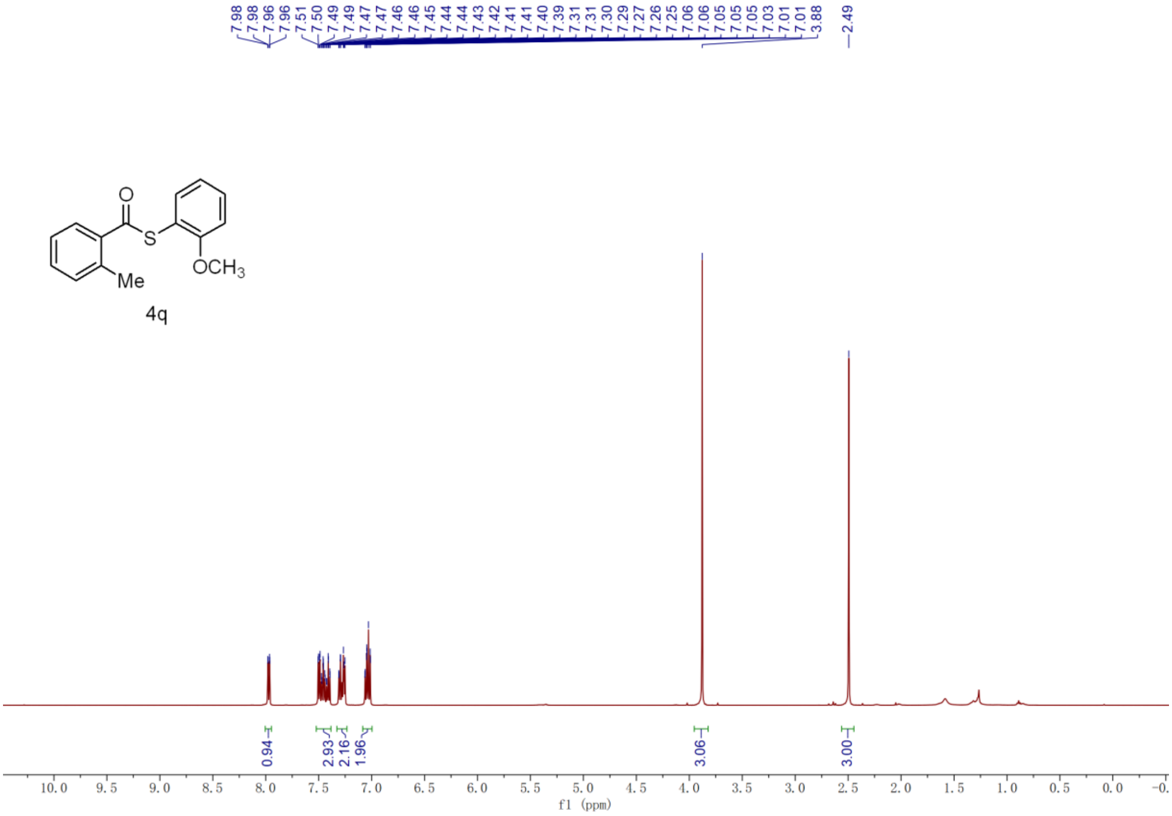

Supplementary Figure 77b | ^{13}C NMR (126 MHz, $CDCl_3$) of *S*-(2-methoxyphenyl) 2-methylbenzothioate (**4q**)

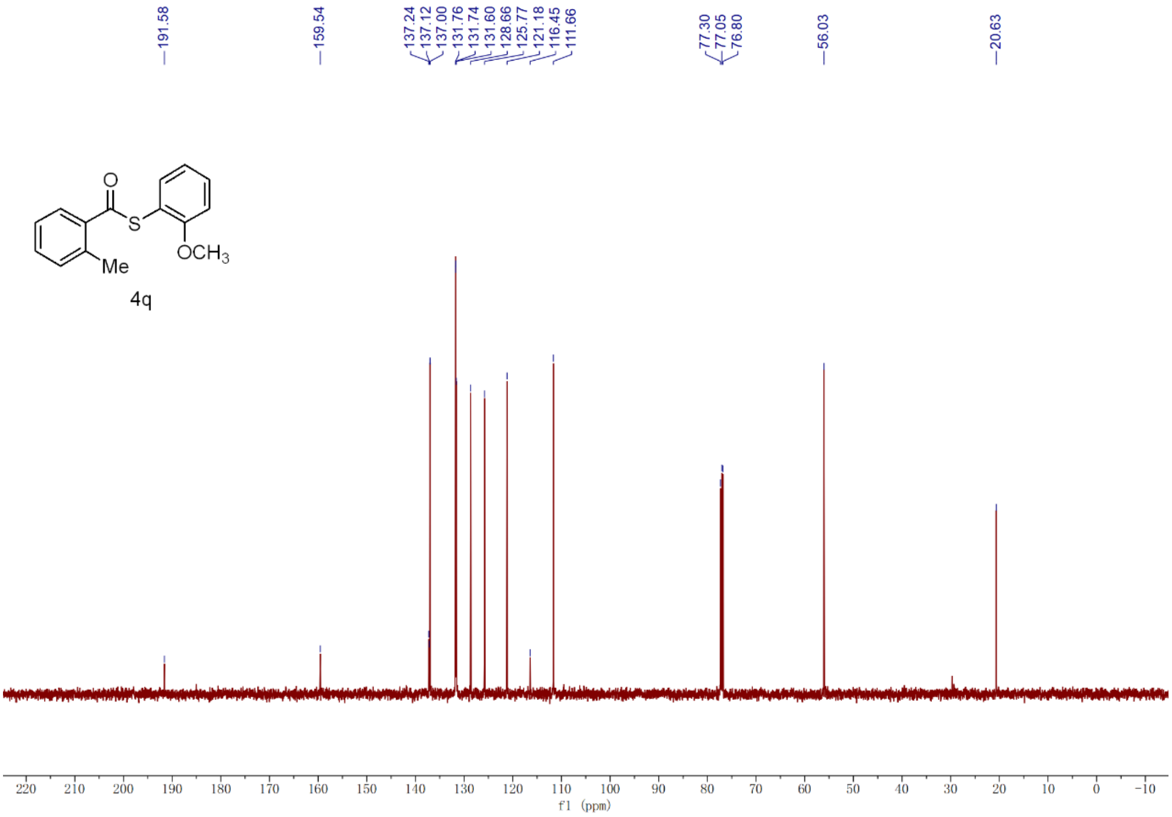

Supplementary Figure 78a | ^{1}H NMR (500 MHz, $CDCl_3$) of *S*-(4-chlorophenyl) 2-methylbenzothioate (**4r**)

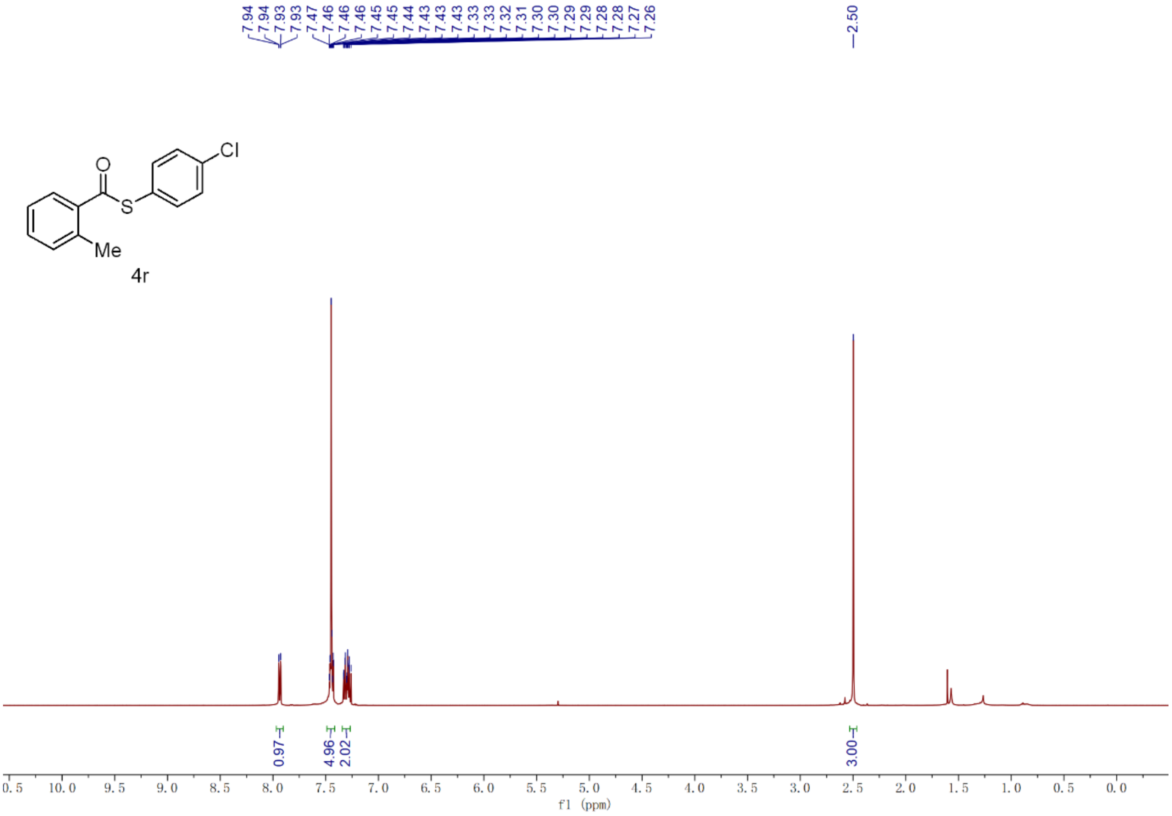

Supplementary Figure 78b | ^{13}C NMR (126 MHz, $CDCl_3$) of *S*-(4-chlorophenyl) 2-methylbenzothioate (**4r**)

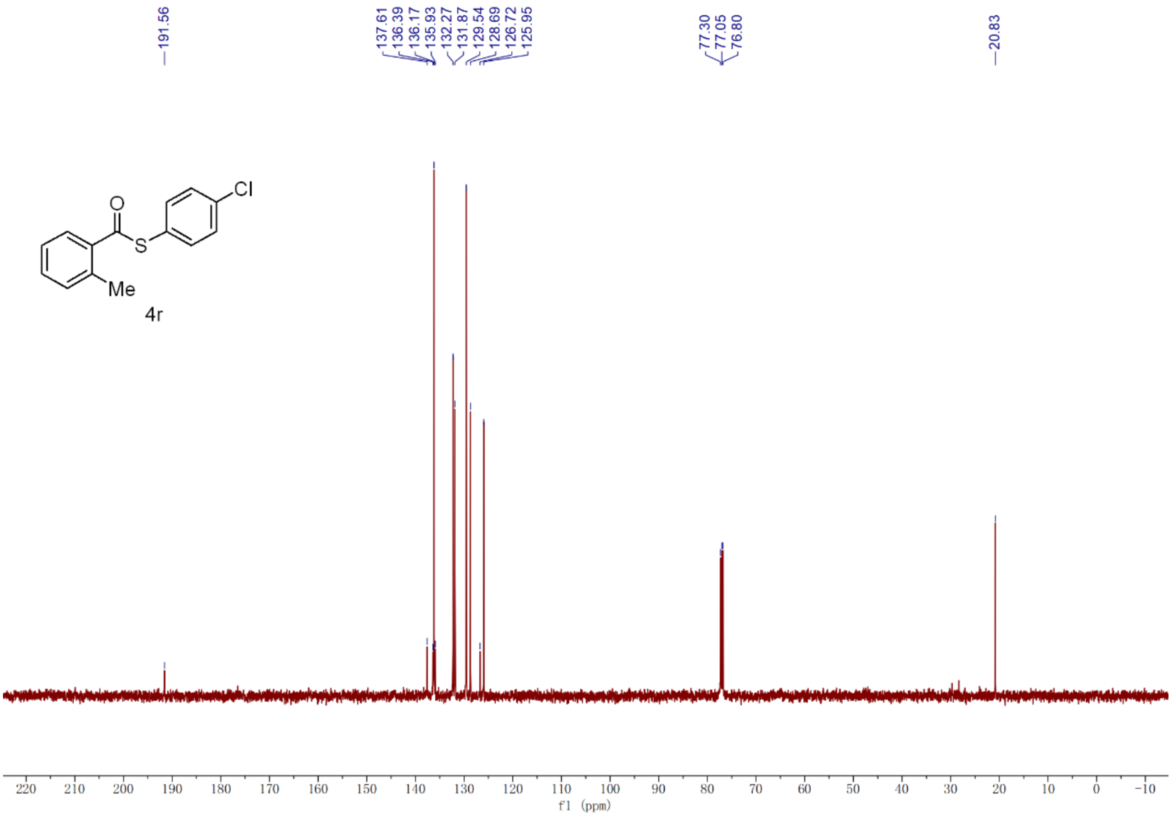

Supplementary Figure 79a | ^{1}H NMR (500 MHz, $CDCl_3$) of *S*-(4-(trifluoromethyl)phenyl) 2-methylbenzothioate (**4s**)

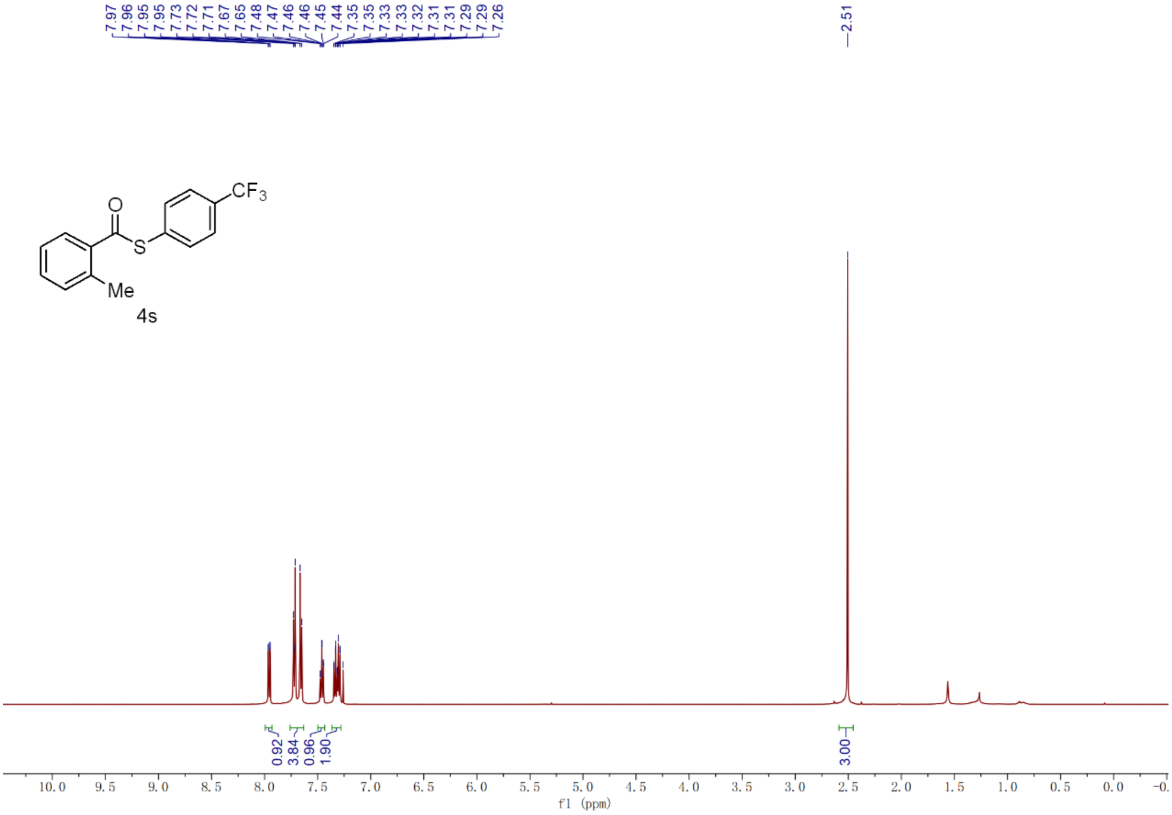

Supplementary Figure 79b | ^{13}C NMR (126 MHz, $CDCl_3$) of *S*-(4-(trifluoromethyl)phenyl) 2-methylbenzothioate (**4s**)

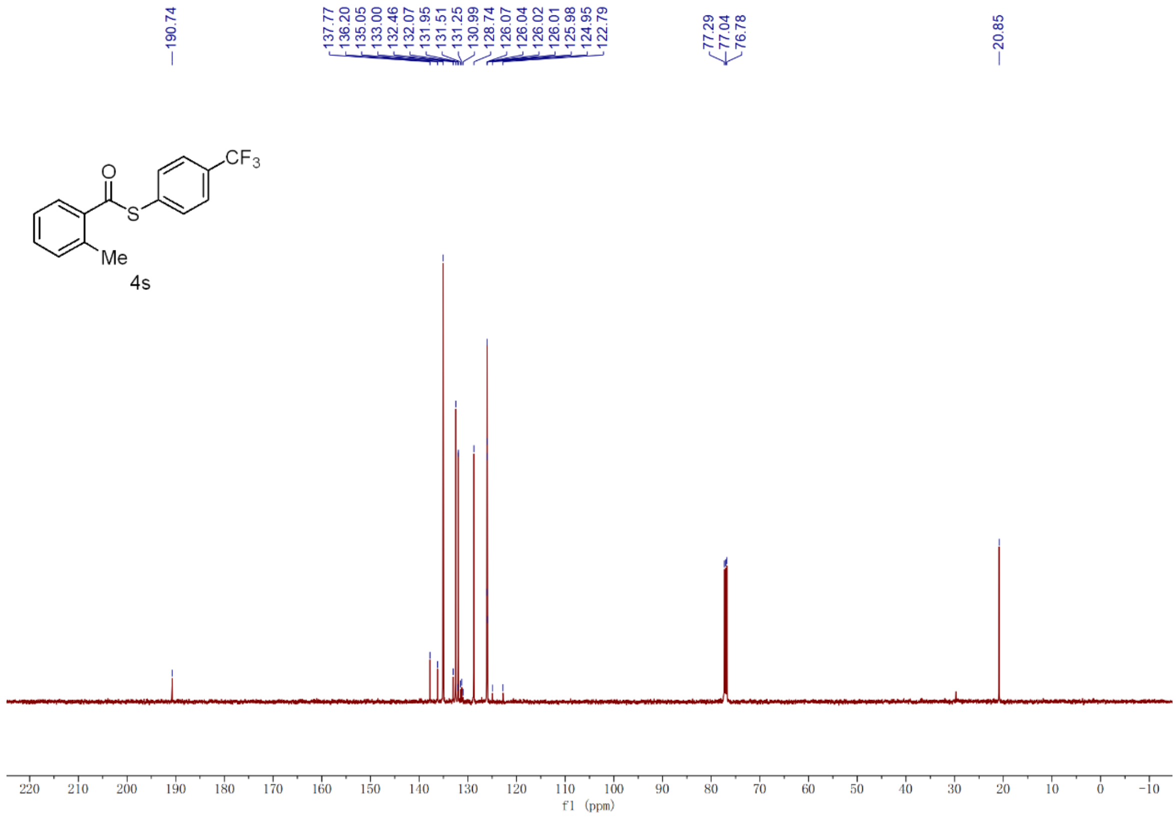

Supplementary Figure 79c | ^{19}F NMR (377 MHz, $CDCl_3$) of *S*-(4-(trifluoromethyl)phenyl) 2-methylbenzothioate (**4s**)

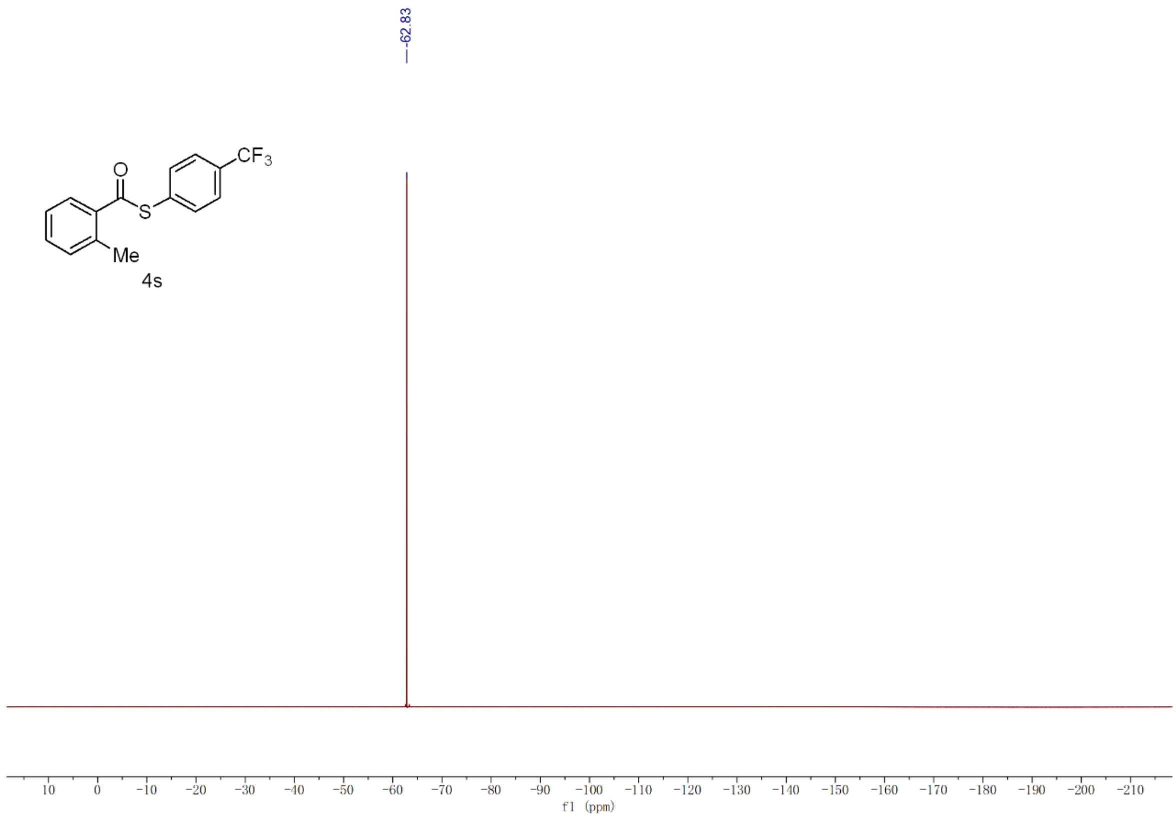

Supplementary Figure 80a | ^{1}H NMR (500 MHz, $CDCl_3$) of *S*-(naphthalen-2-yl) 2-methylbenzothioate (**4t**)

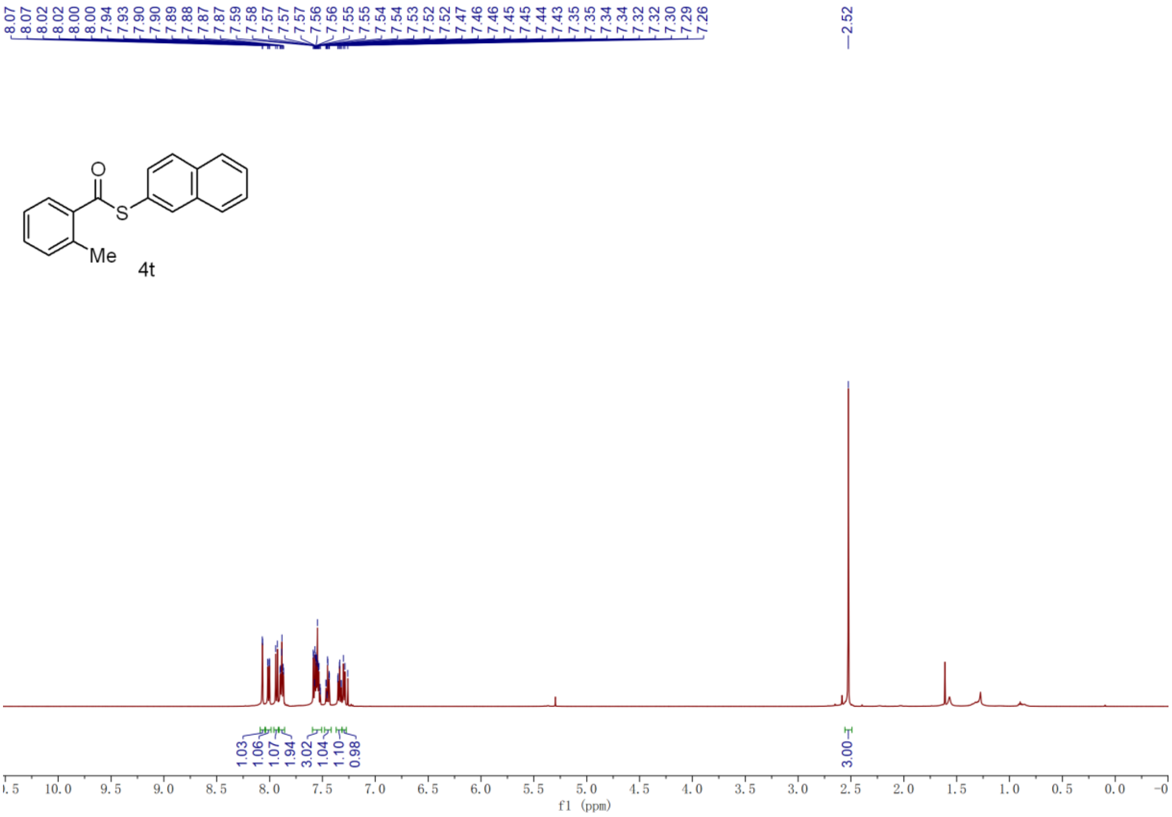

Supplementary Figure 80b | ^{13}C NMR (126 MHz, $CDCl_3$) of *S*-(naphthalen-2-yl) 2-methylbenzothioate (**4t**)

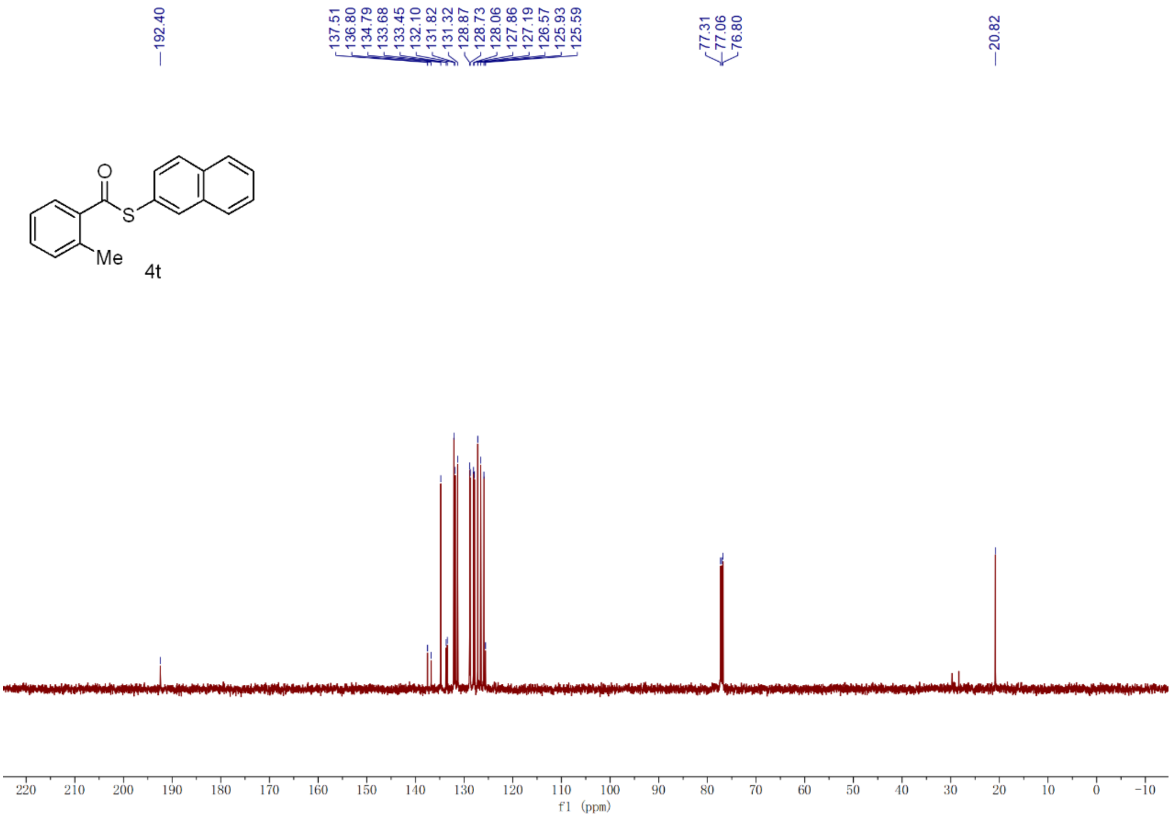

Supplementary Figure 81a | ^{1}H NMR (400 MHz, $CDCl_3$) of Methyl 3-((2-methylbenzoyl)thio)thiophene-2-carboxylate (**4u**)

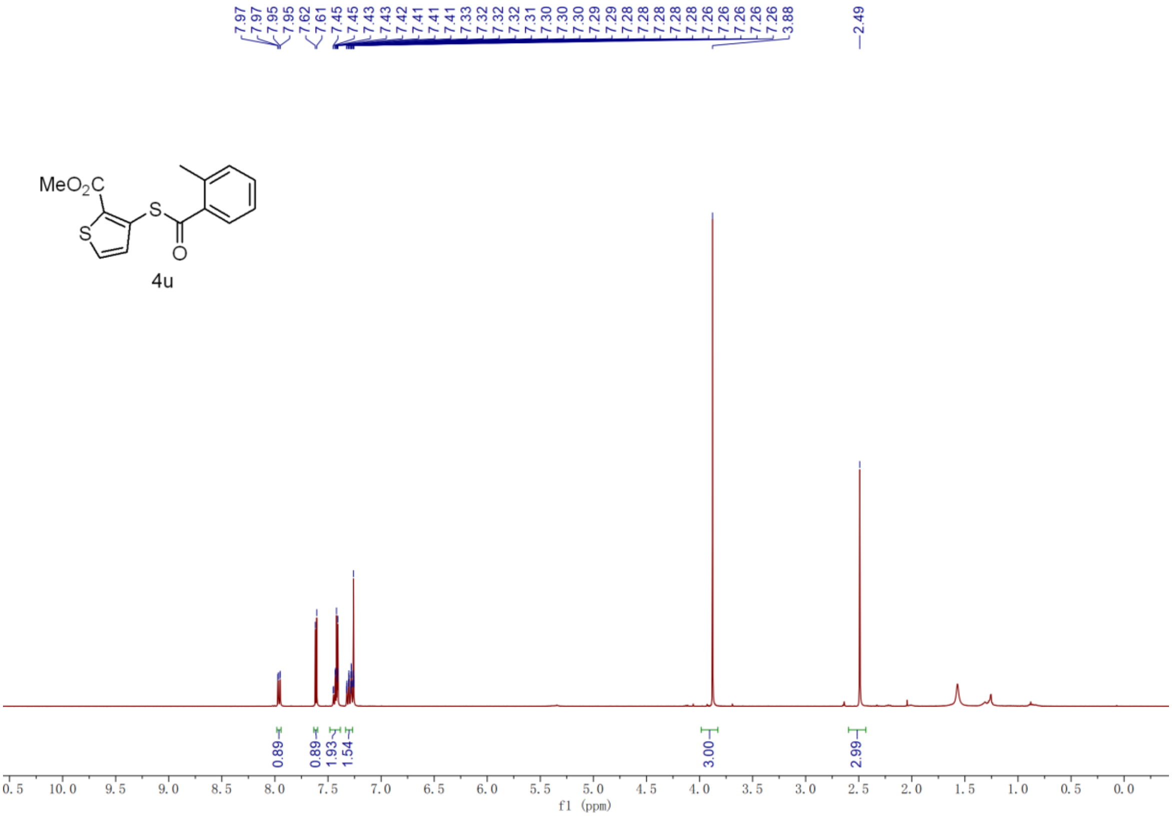

Supplementary Figure 81b | ^{13}C NMR (126 MHz, $CDCl_3$) of Methyl 3-((2-methylbenzoyl)thio)thiophene-2-carboxylate (**4u**)

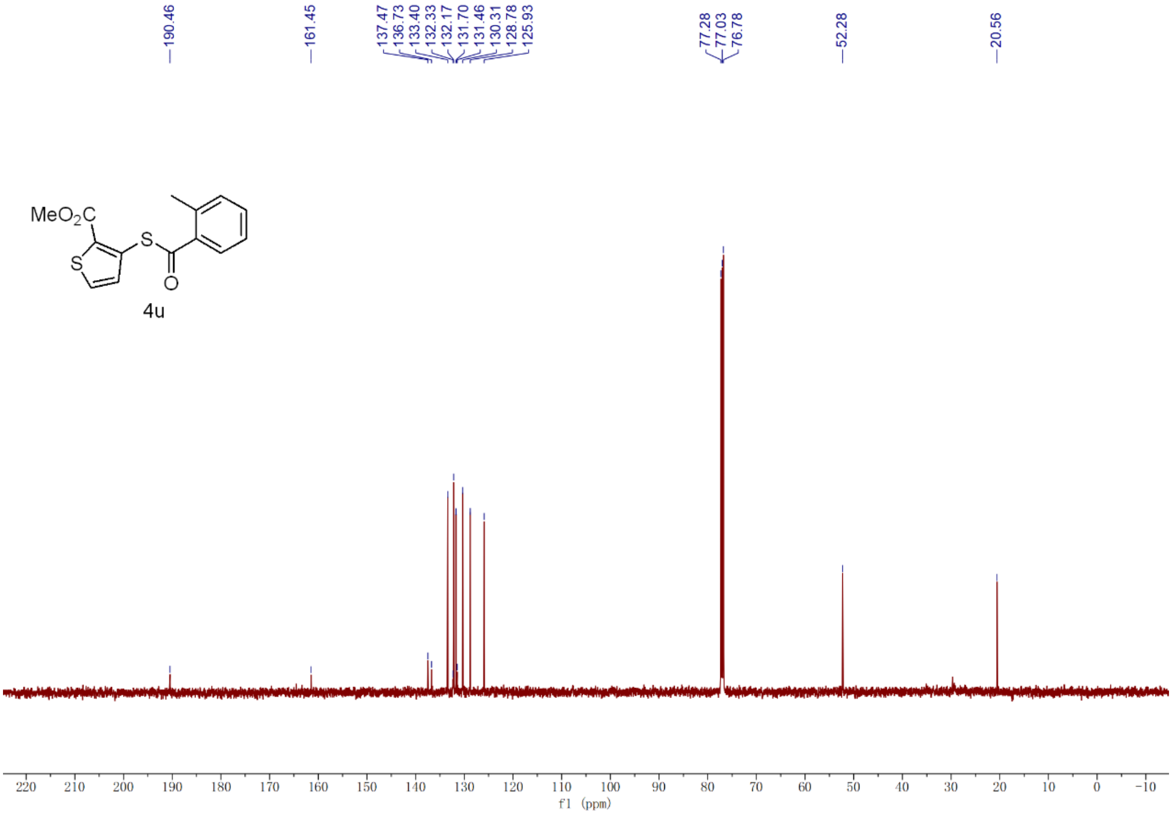

Supplementary Figure 82a | ^{1}H NMR (500 MHz, $CDCl_3$) of *S*-Phenyl benzenesulfonothioate (**9a**)

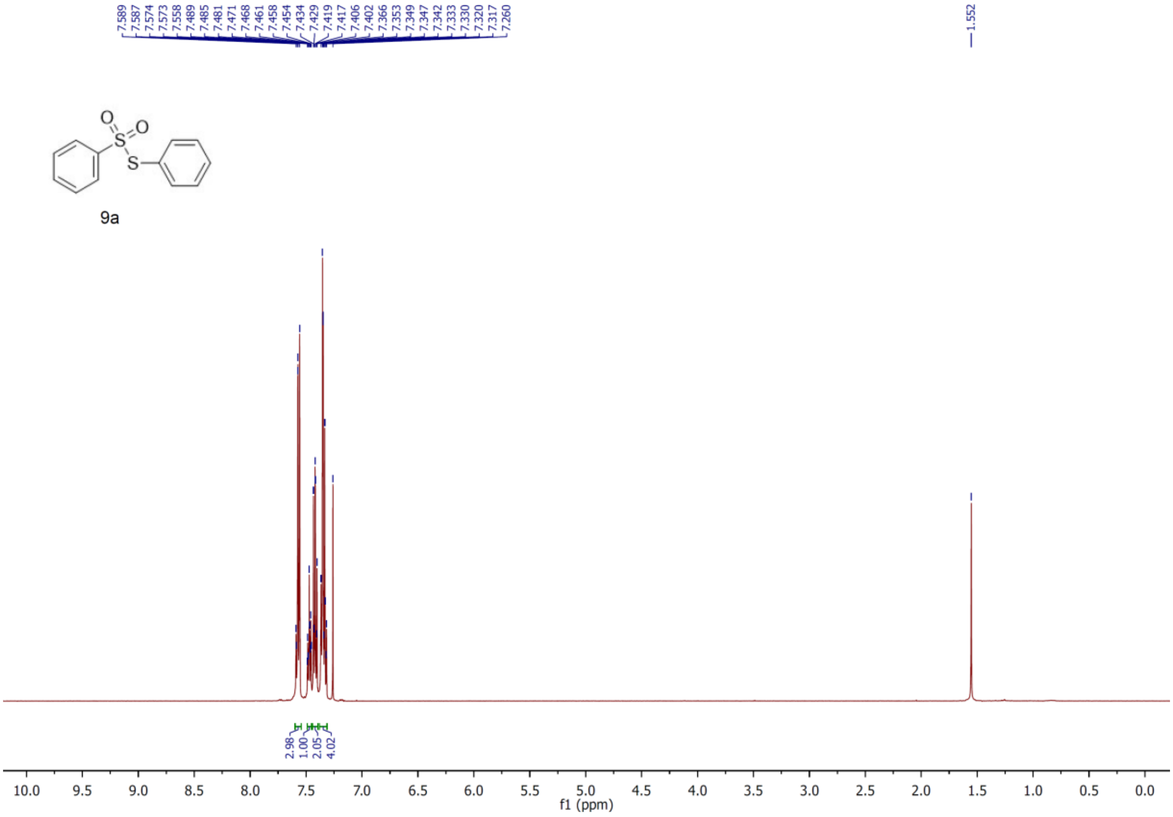

Supplementary Figure 82b | ^{13}C NMR (126 MHz, $CDCl_3$) of *S*-Phenyl benzenesulfonothioate (**9a**)

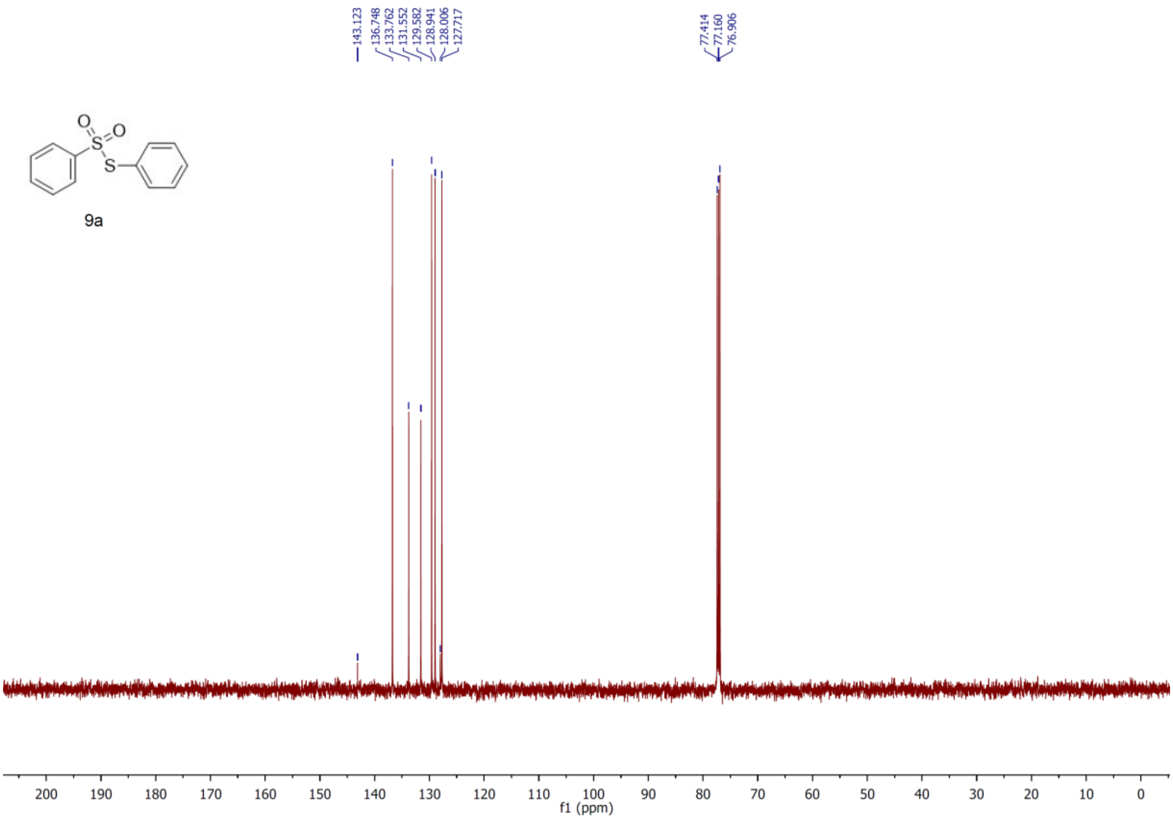

Supplementary Figure 83a | ^{1}H NMR (400 MHz, $CDCl_3$) of *S*-(*p*-tolyl) 4-methylbenzenesulfonothioate (**9b**)

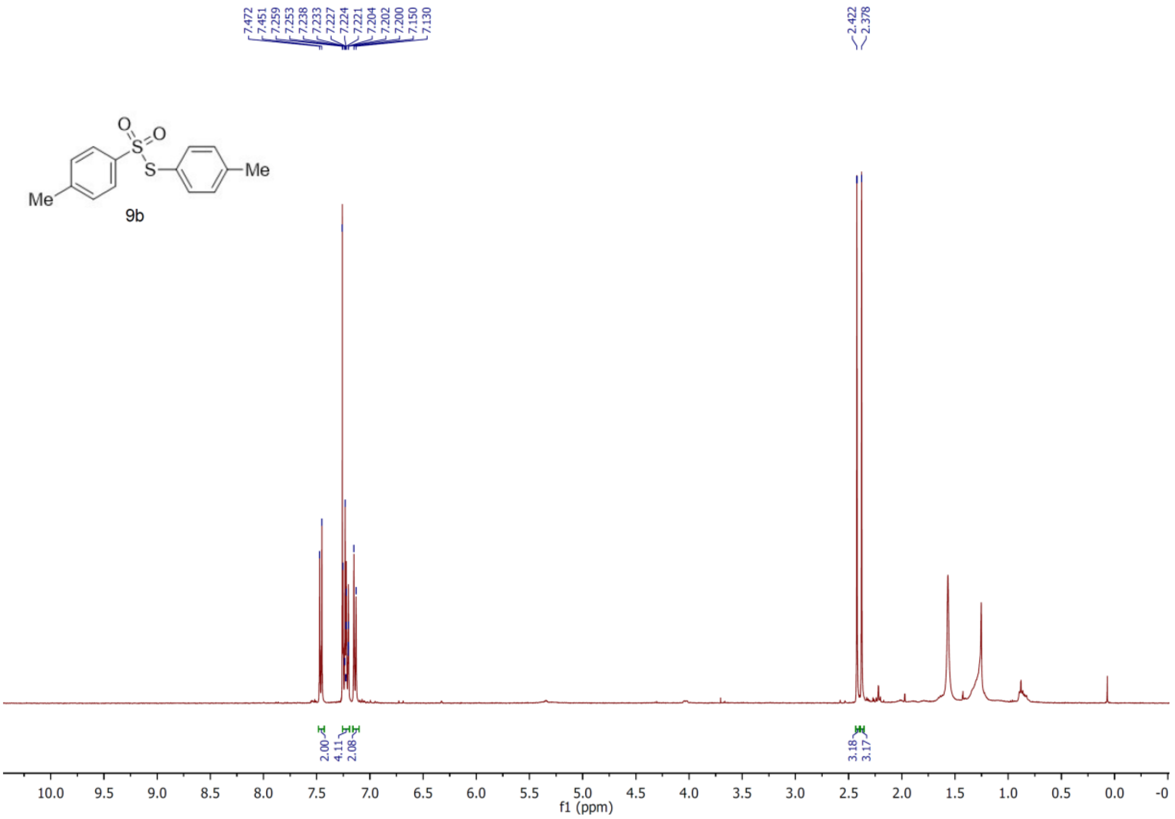

Supplementary Figure 83b | ^{13}C NMR (126 MHz, $CDCl_3$) of *S*-(*p*-tolyl) 4-methylbenzenesulfonothioate (**9b**)

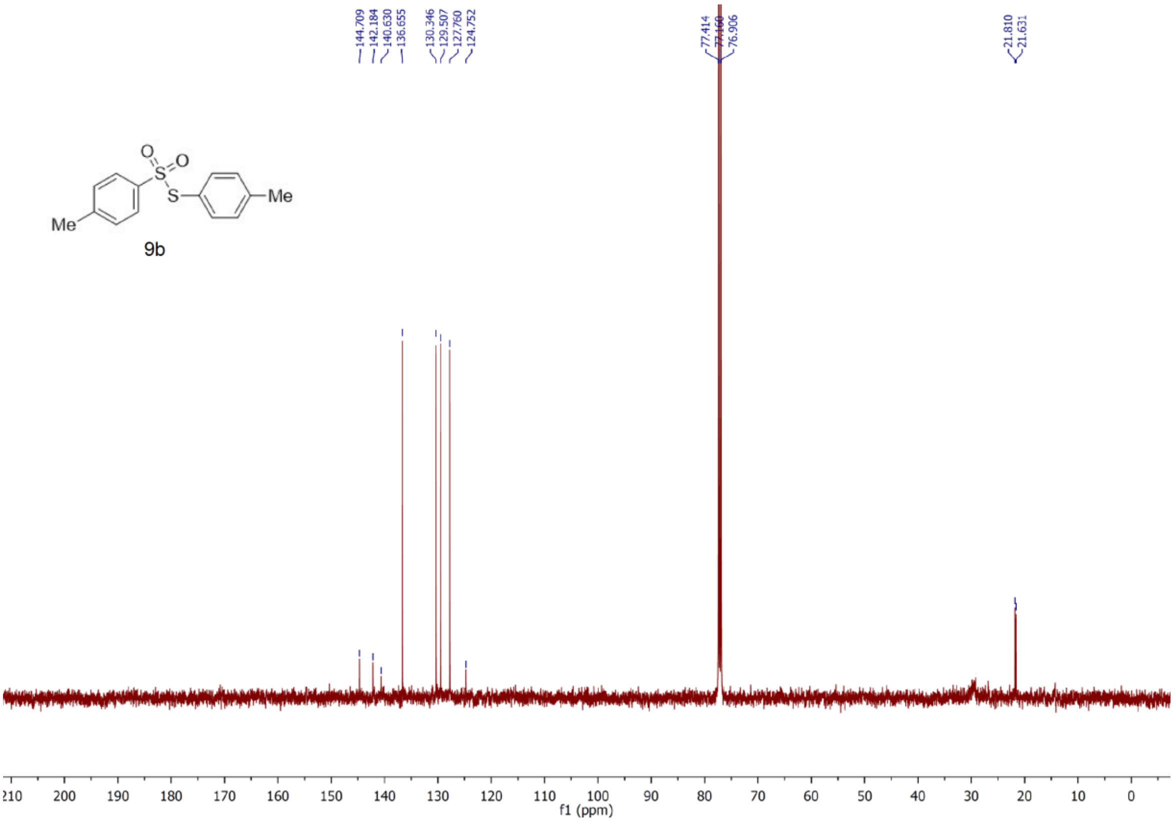

Supplementary Figure 84a | ^{1}H NMR (500 MHz, $CDCl_3$) of 1,3-Diphenylprop-2-yn-1-one (**14a**)

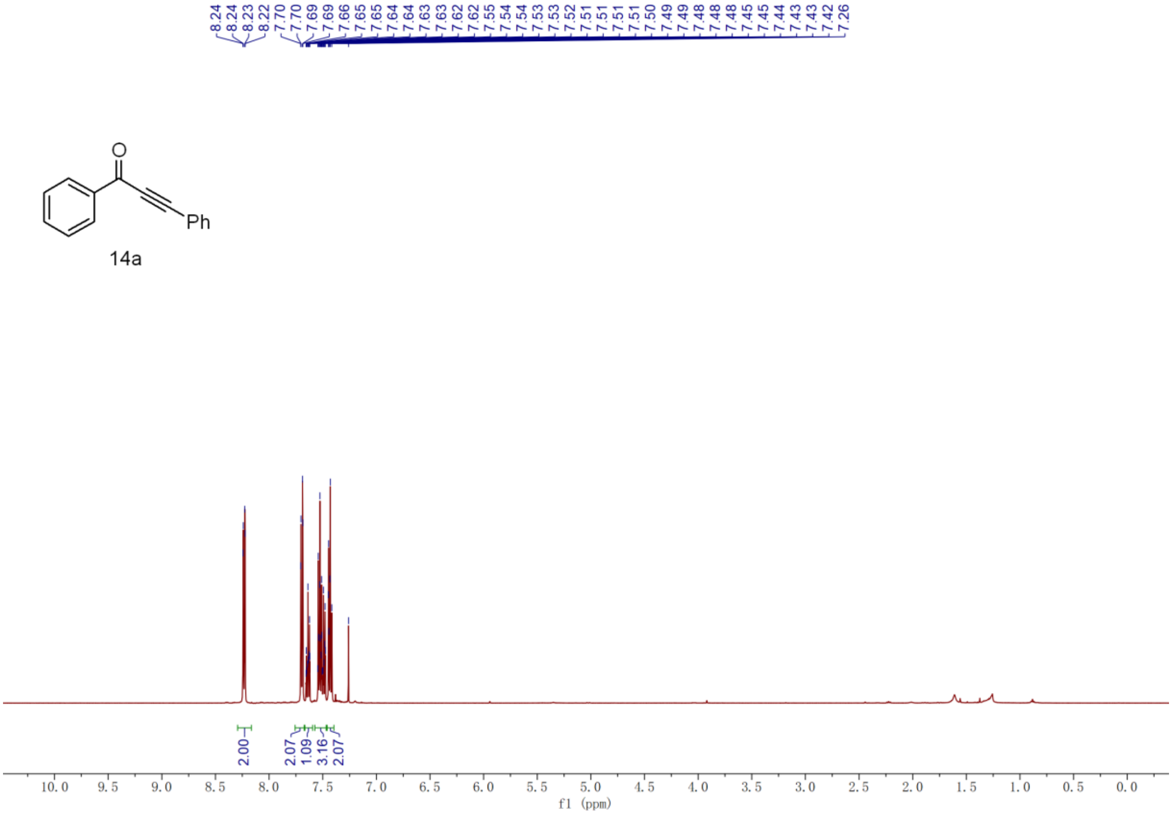

Supplementary Figure 84b | ^{13}C NMR (126 MHz, $CDCl_3$) of 1,3-Diphenylprop-2-yn-1-one (**14a**)

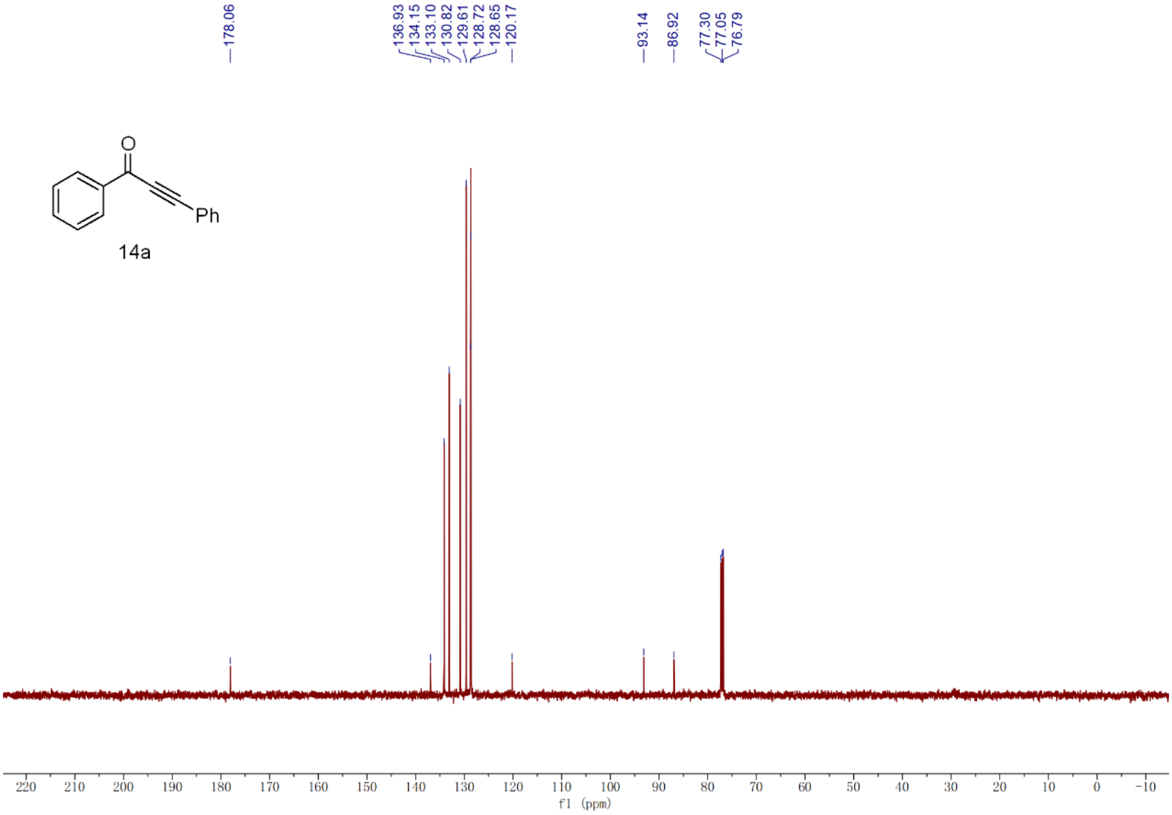

Supplementary Figure 85a | ^{1}H NMR (500 MHz, $CDCl_3$) of 1-(4-fluorophenyl)-3-phenylprop-2-yn-1-one (**14b**)

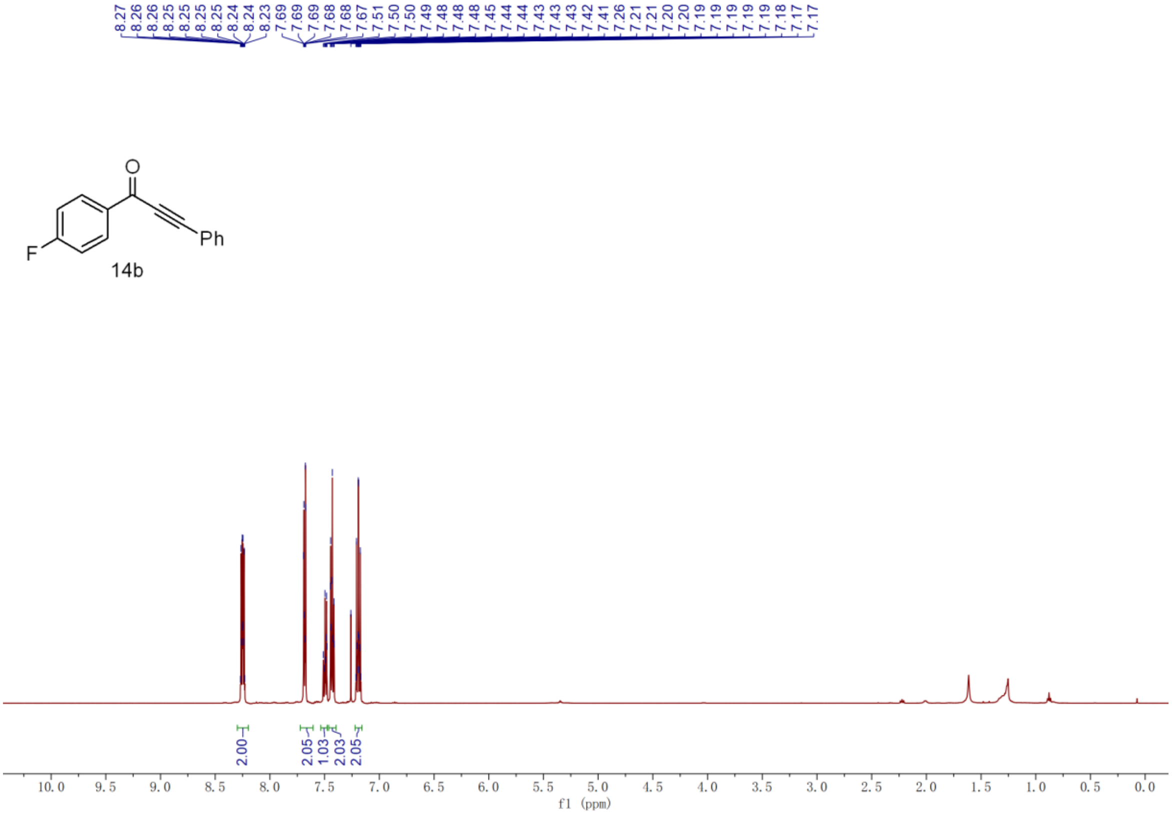

Supplementary Figure 85b | ^{13}C NMR (126 MHz, $CDCl_3$) of 1-(4-fluorophenyl)-3-phenylprop-2-yn-1-one (**14b**)

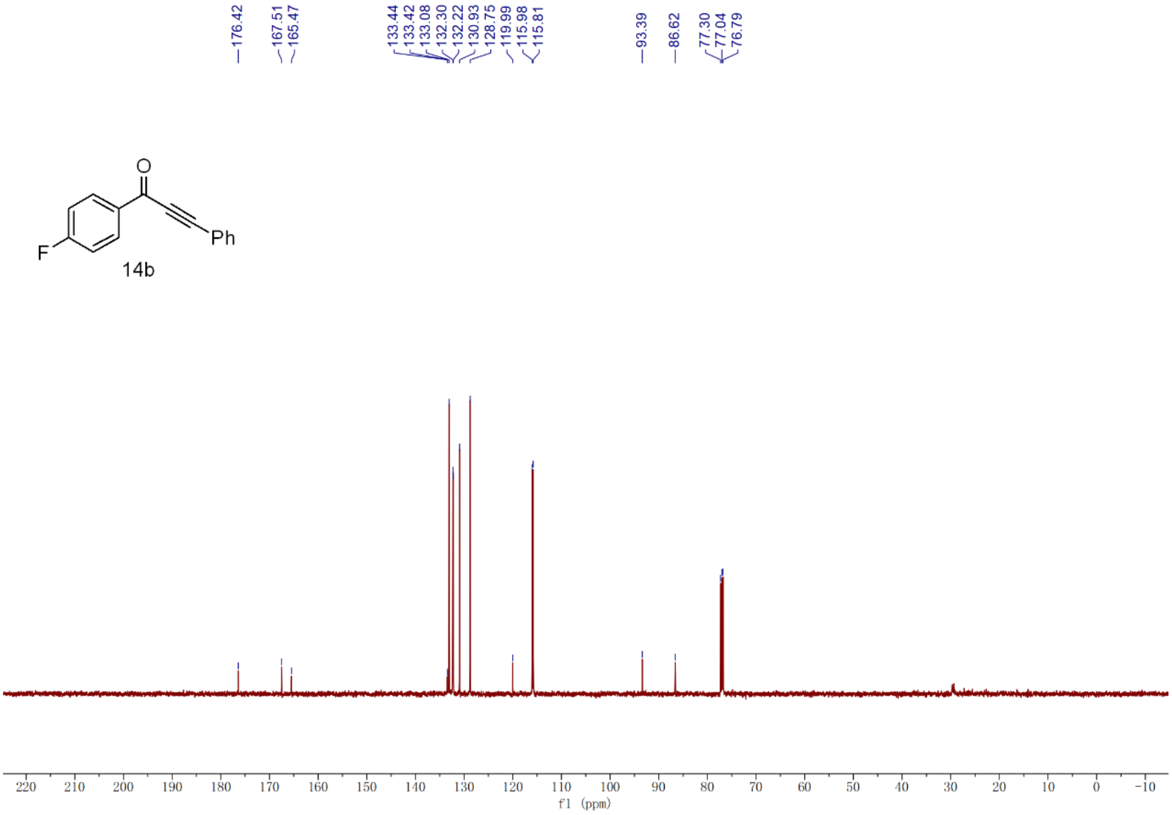

Supplementary Figure 85c | ^{19}F NMR (377 MHz, $CDCl_3$) of 1-(4-fluorophenyl)-3-phenylprop-2-yn-1-one (**14b**)

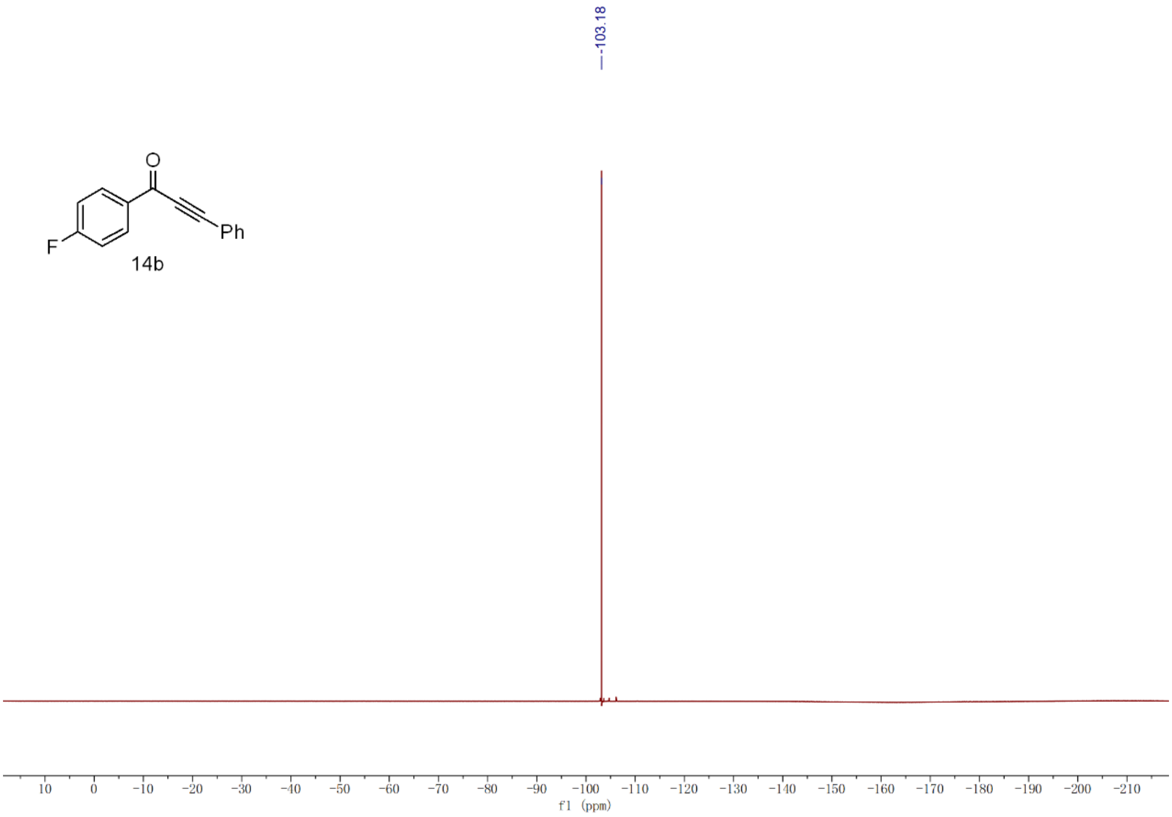

Supplementary Figure 86a | ^{1}H NMR (500 MHz, $CDCl_3$) of 1-(4-Chlorophenyl)-3-phenylprop-2-yn-1-one (**14c**)

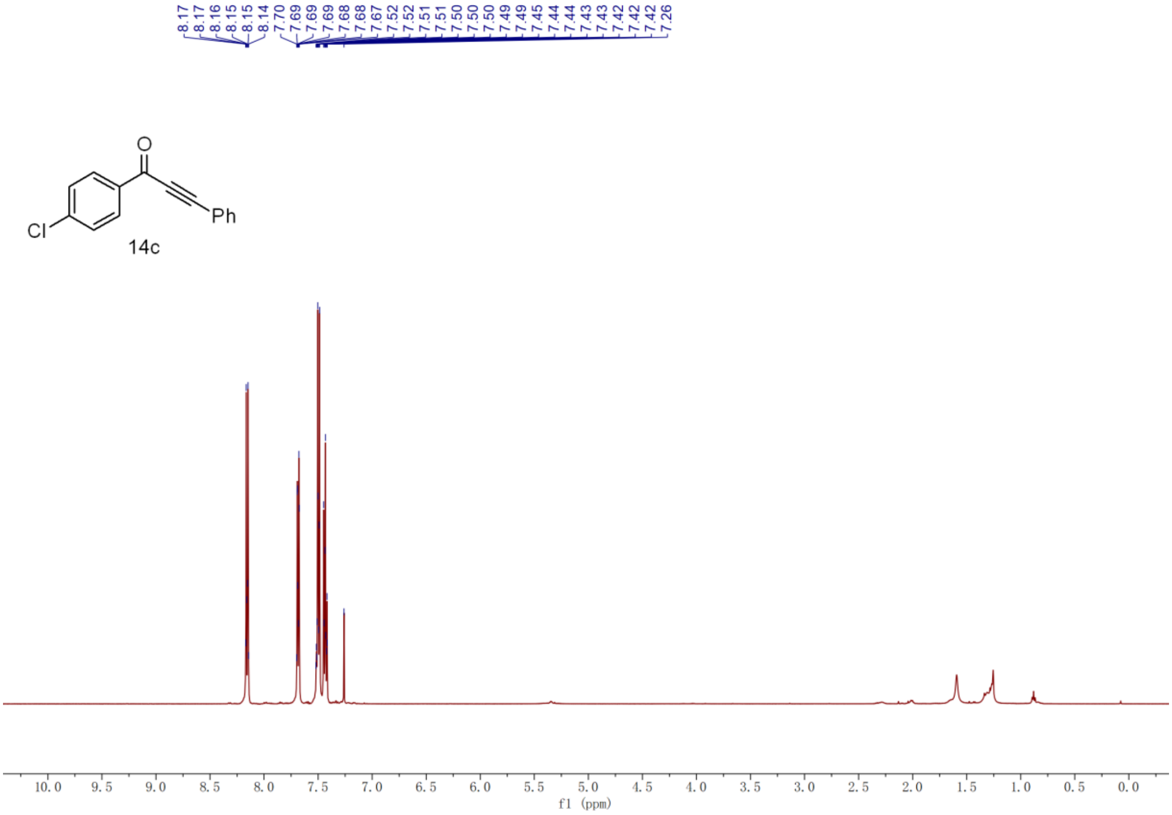

Supplementary Figure 86b | ^{13}C NMR (126 MHz, $CDCl_3$) of 1-(4-Chlorophenyl)-3-phenylprop-2-yn-1-one (**14c**)

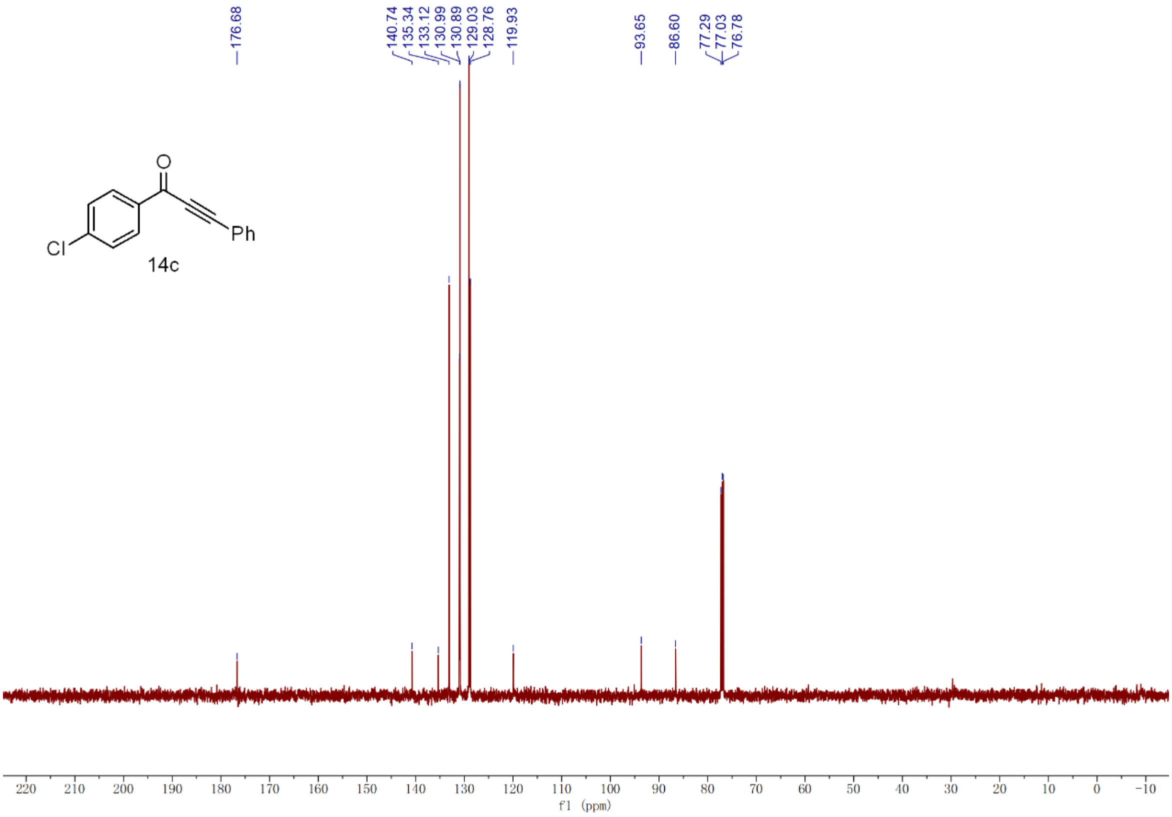

Supplementary Figure 87a | ^{1}H NMR (400 MHz, $CDCl_3$) of 1-(4-Bromophenyl)-3-phenylprop-2-yn-1-one (**14d**)

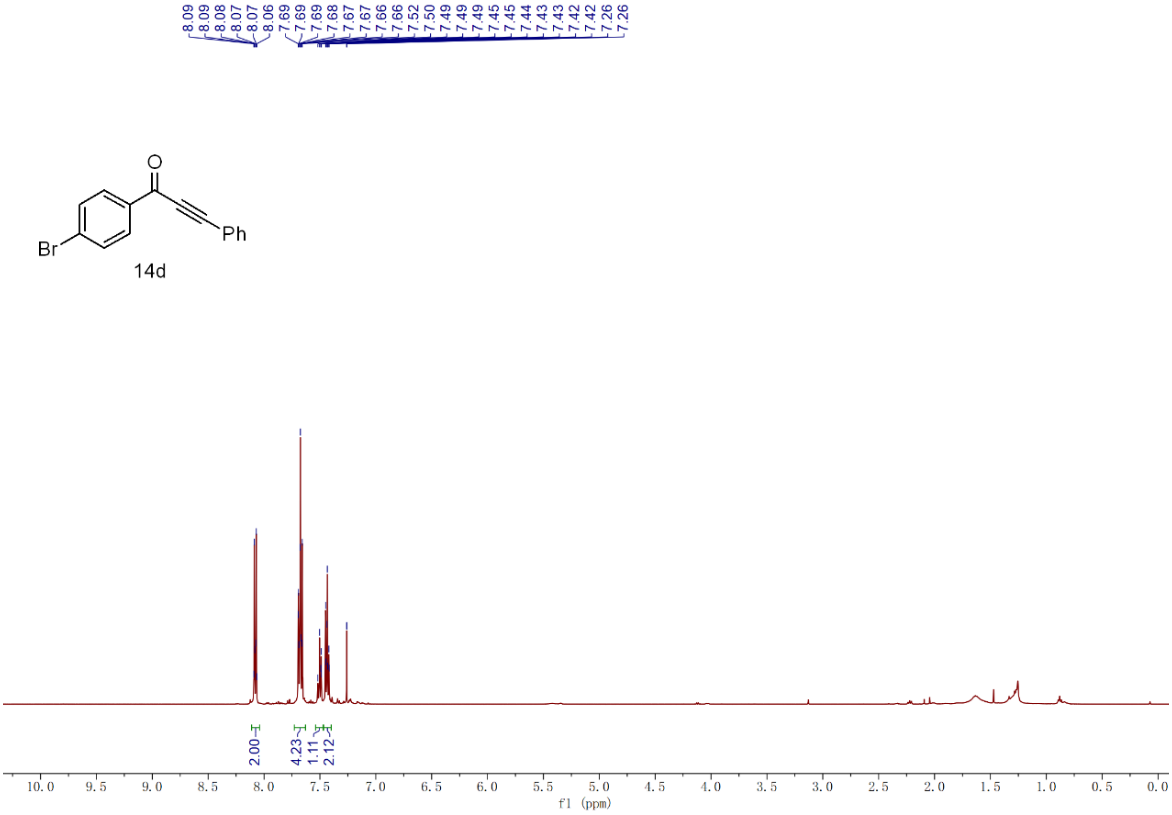

Supplementary Figure 87b | ^{13}C NMR (126 MHz, $CDCl_3$) of 1-(4-Bromophenyl)-3-phenylprop-2-yn-1-one (**14d**)

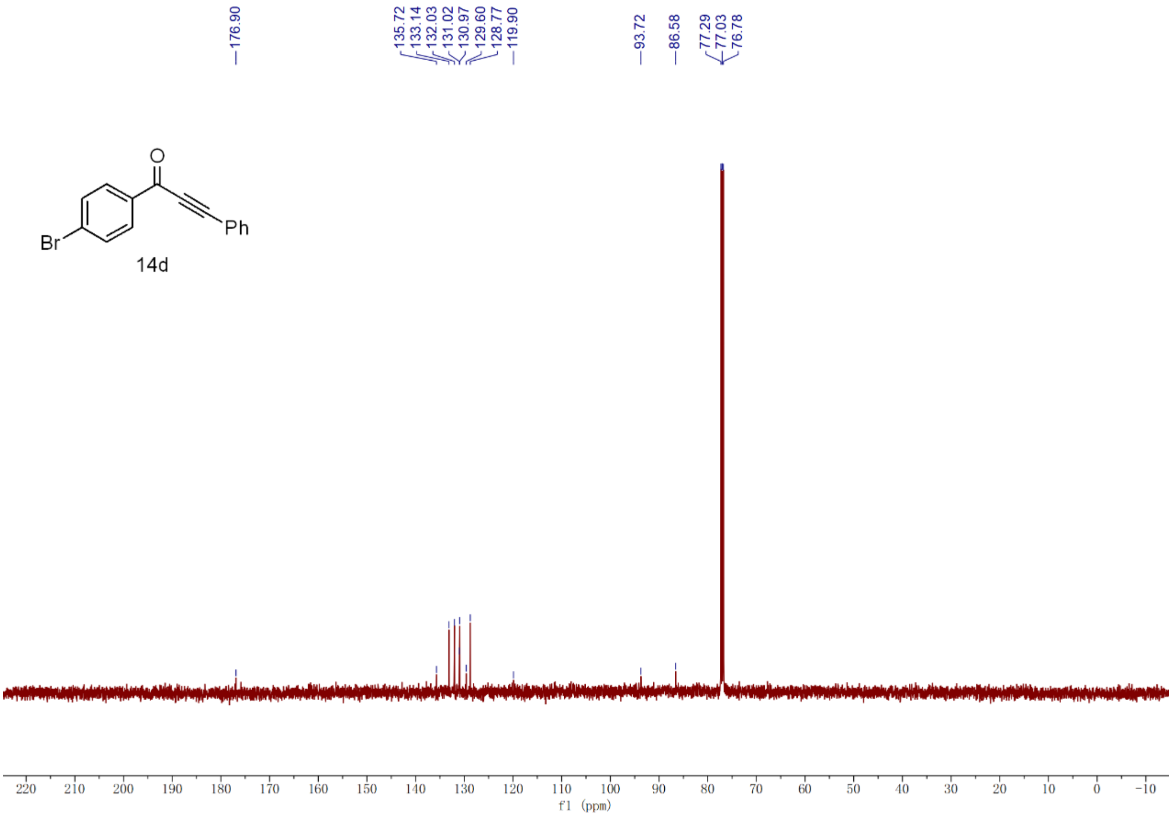

Supplementary Figure 88a | ^{1}H NMR (500 MHz, $CDCl_3$) of 3-Phenyl-1-(4-(trifluoromethyl)phenyl)prop-2-yn-1-one (**14e**)

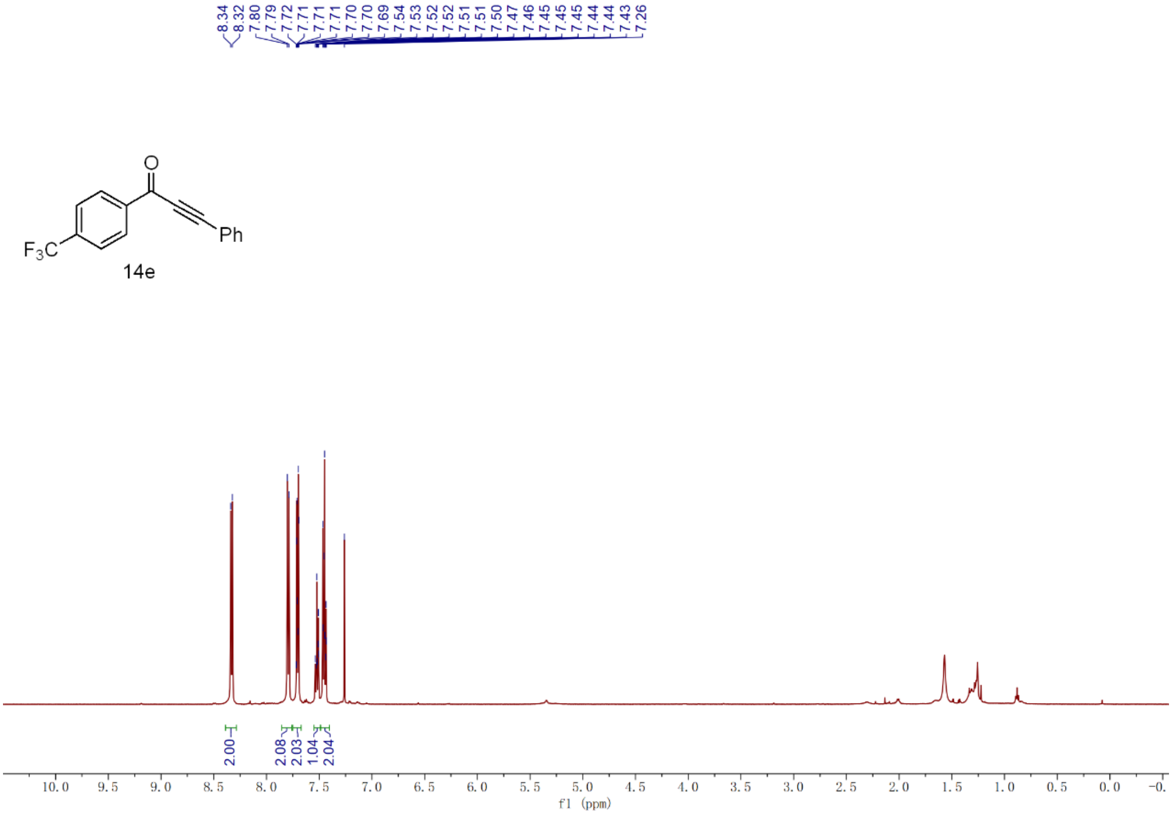

Supplementary Figure 88b | ^{13}C NMR (126 MHz, $CDCl_3$) of 3-Phenyl-1-(4-(trifluoromethyl)phenyl)prop-2-yn-1-one (**14e**)

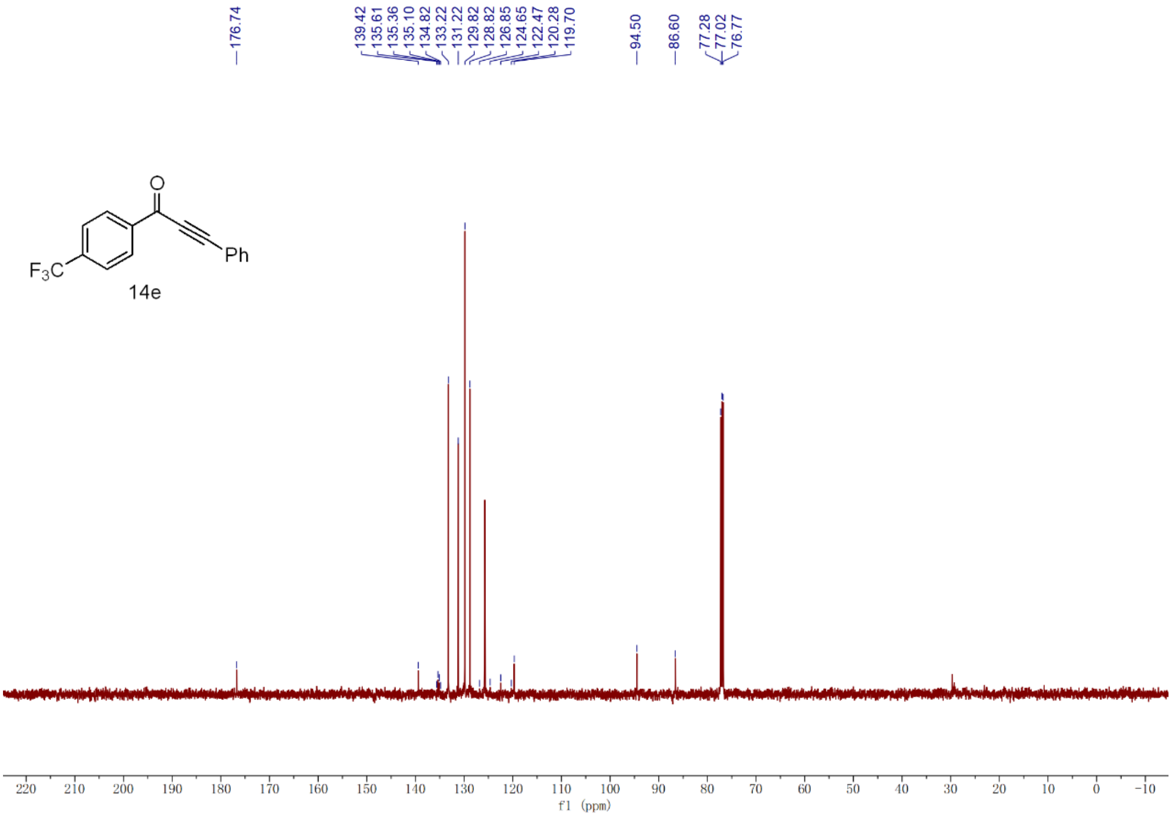

Supplementary Figure 88c | ^{19}F NMR (377 MHz, $CDCl_3$) of 3-Phenyl-1-(4-(trifluoromethyl)phenyl)prop-2-yn-1-one (**14e**)

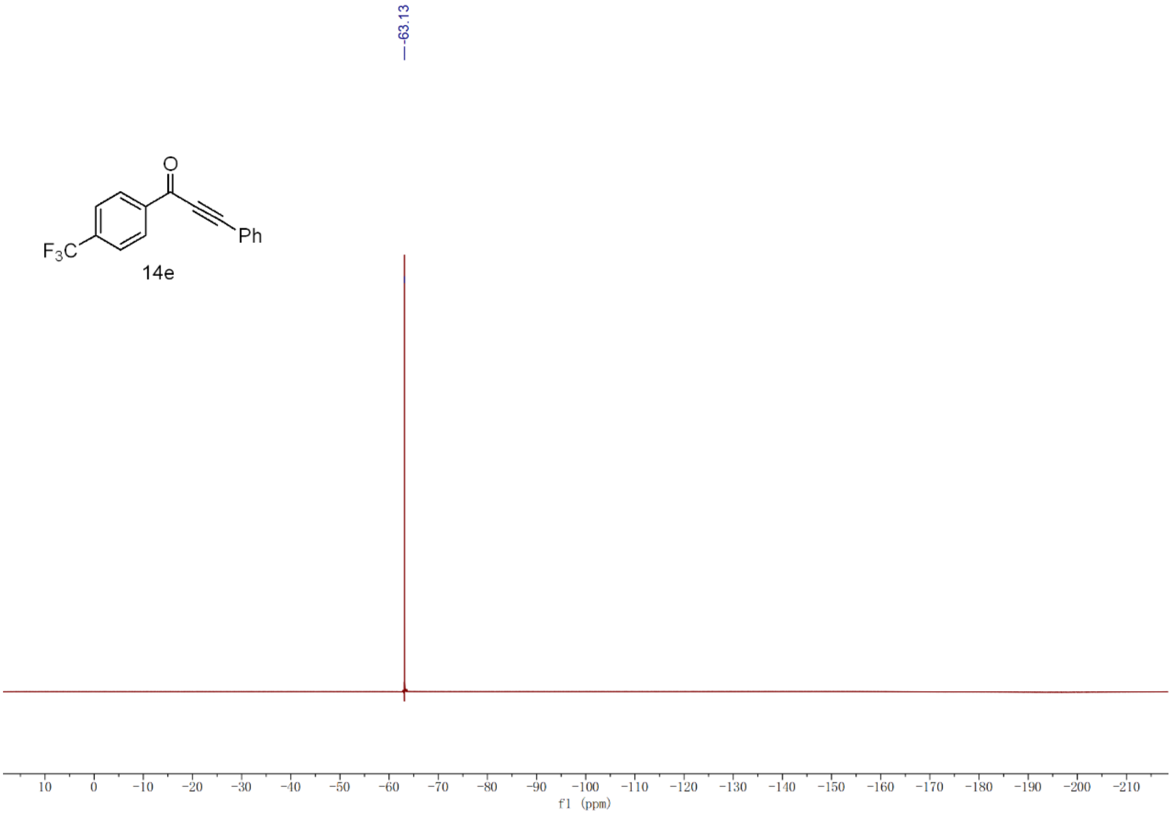

Supplementary Figure 89a | ^{1}H NMR (400 MHz, $CDCl_3$) of 3-Phenyl-1-(m-tolyl)prop-2-yn-1-one (**14f**)

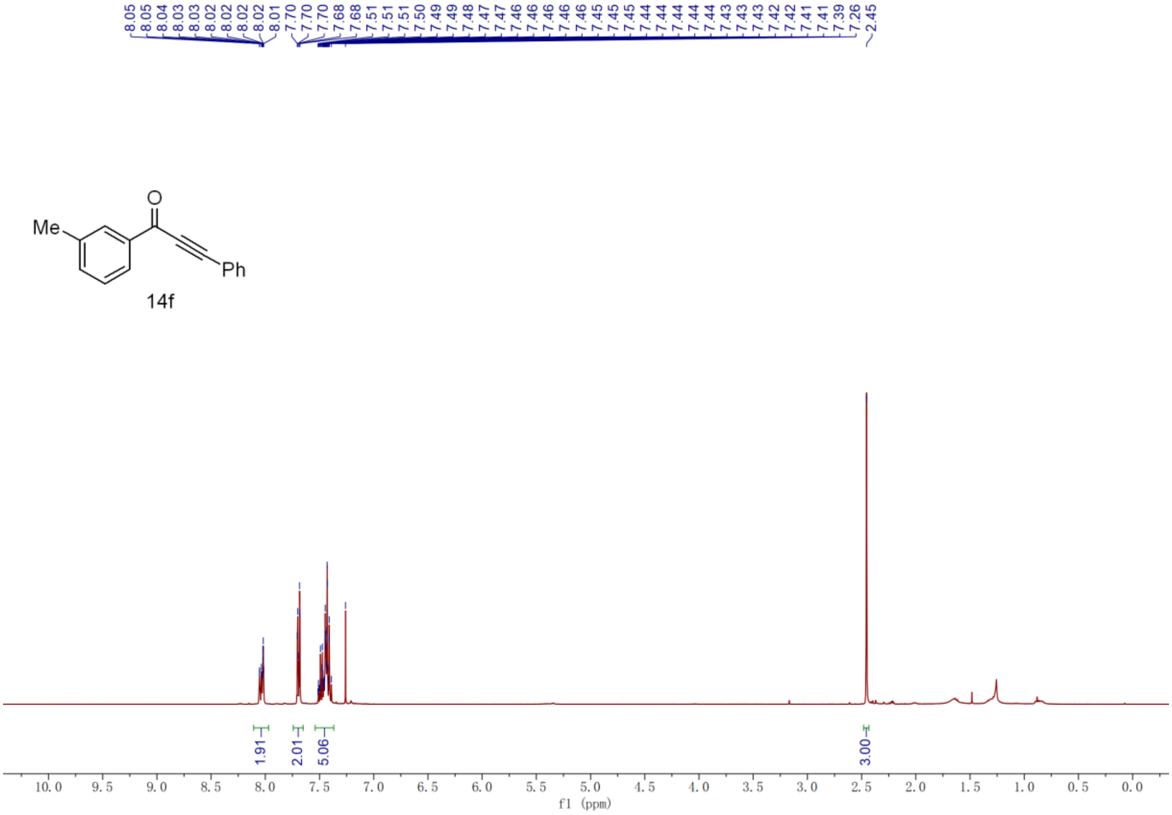

Supplementary Figure 89b | ^{13}C NMR (126 MHz, $CDCl_3$) of 3-Phenyl-1-(m-tolyl)prop-2-yn-1-one (**14f**)

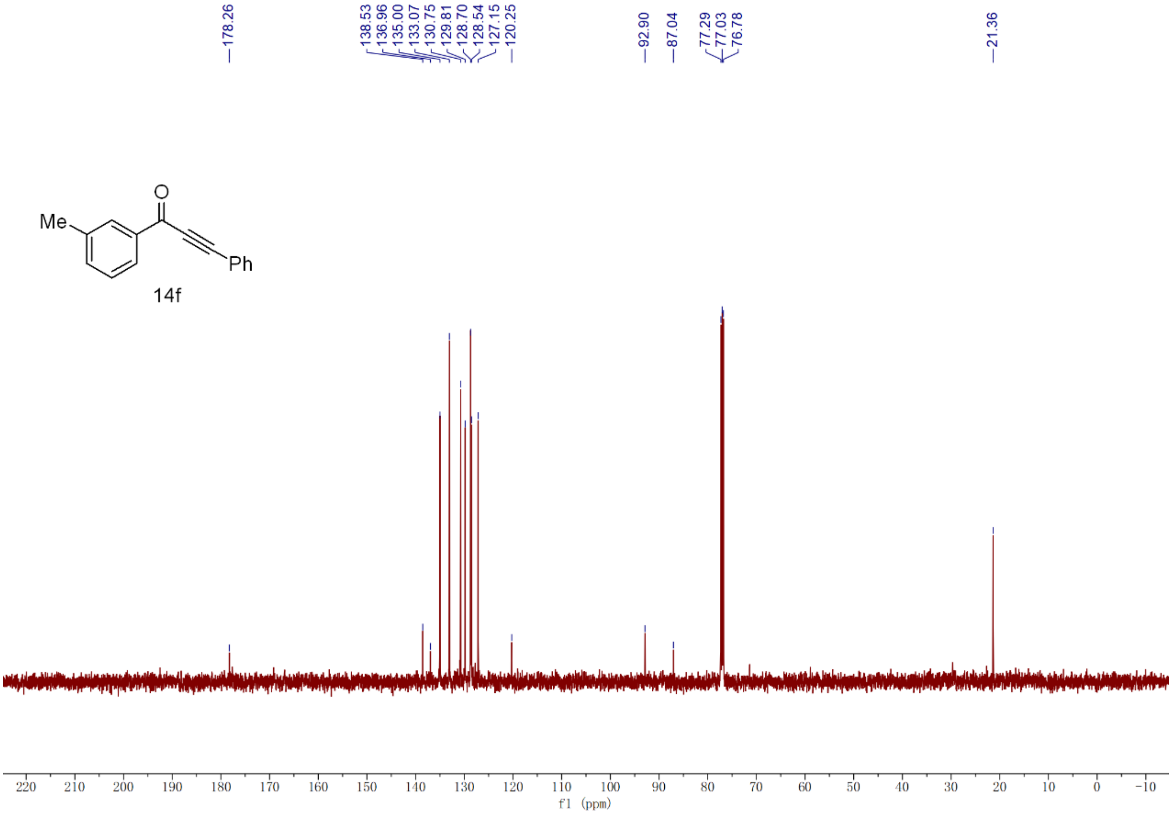

Supplementary Figure 90a | ^{1}H NMR (500 MHz, $CDCl_3$) of 1-(2,2-Difluorobenzo[d][1,3]dioxol-5-yl)-3-phenylprop-2-yn-1-one (**14g**)

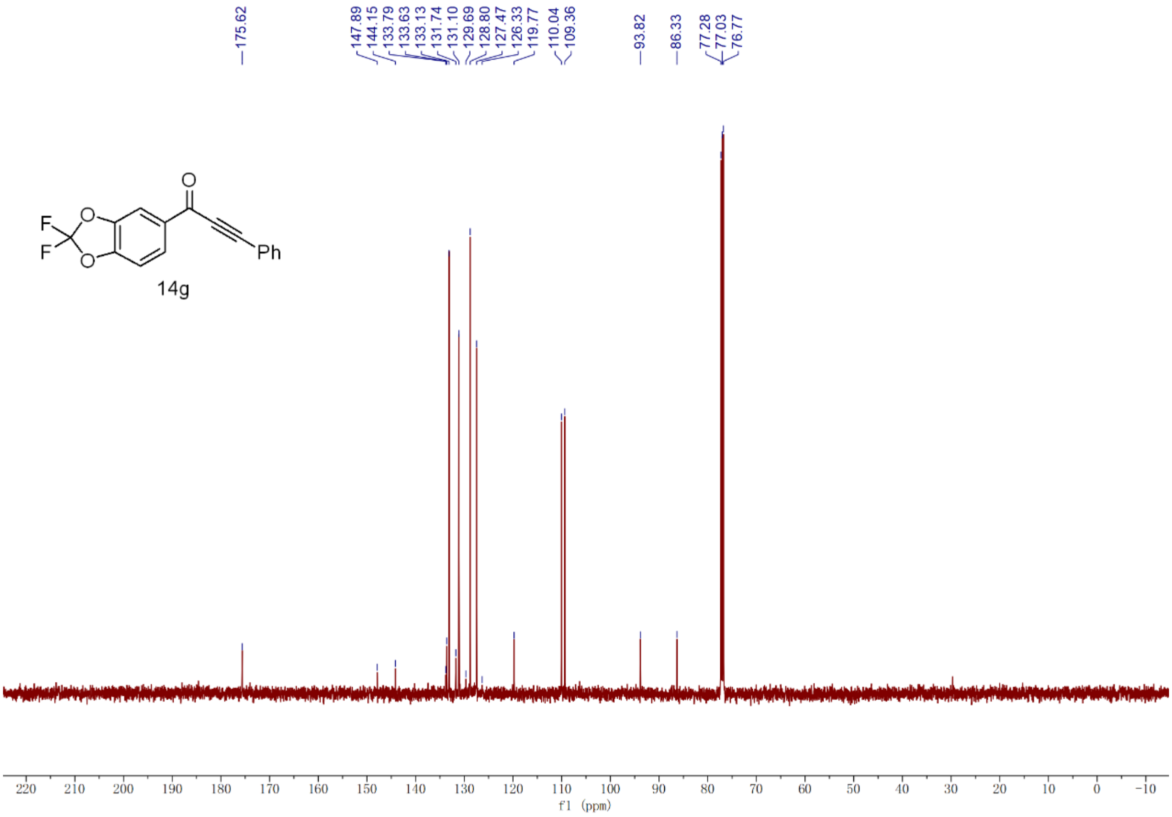

Supplementary Figure 90b | ^{13}C NMR (126 MHz, $CDCl_3$) of 1-(2,2-Difluorobenzo[d][1,3]dioxol-5-yl)-3-phenylprop-2-yn-1-one (**14g**)

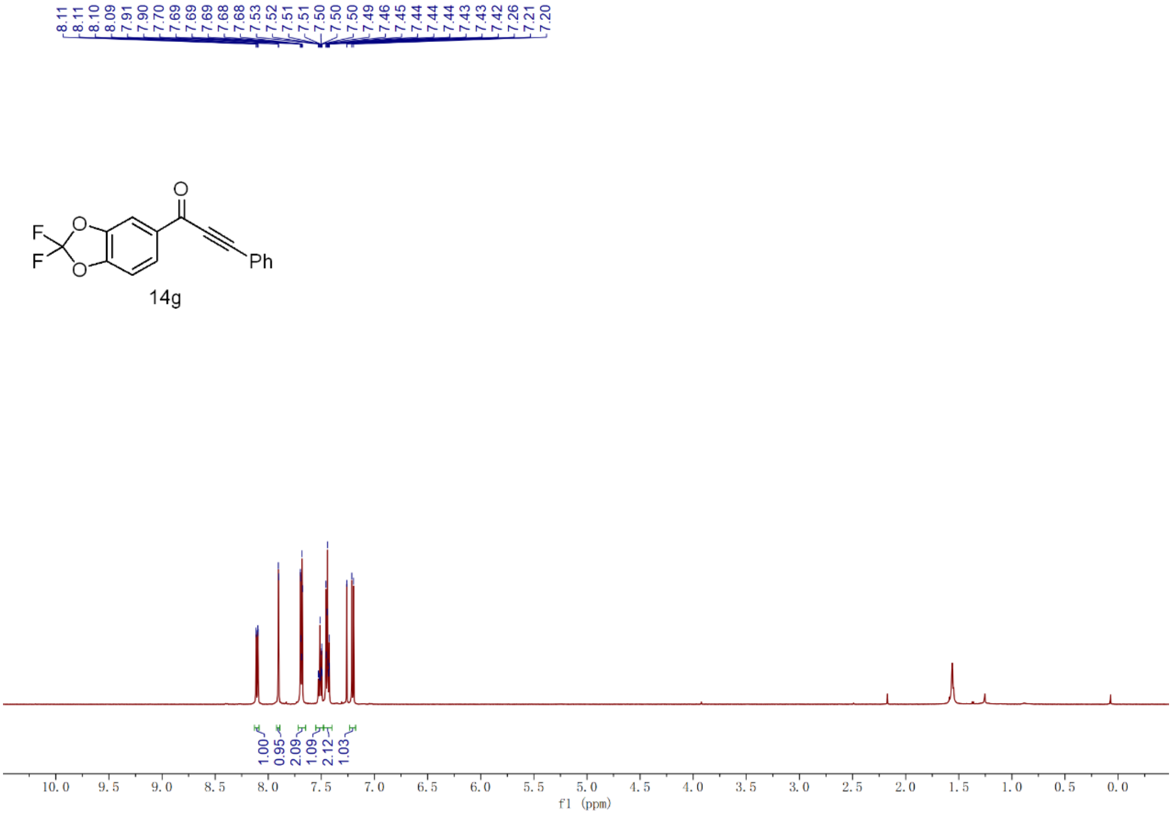

Supplementary Figure 90c | ^{19}F NMR (377 MHz, $CDCl_3$) of 1-(2,2-Difluorobenzo[d][1,3]dioxol-5-yl)-3-phenylprop-2-yn-1-one (**14g**)

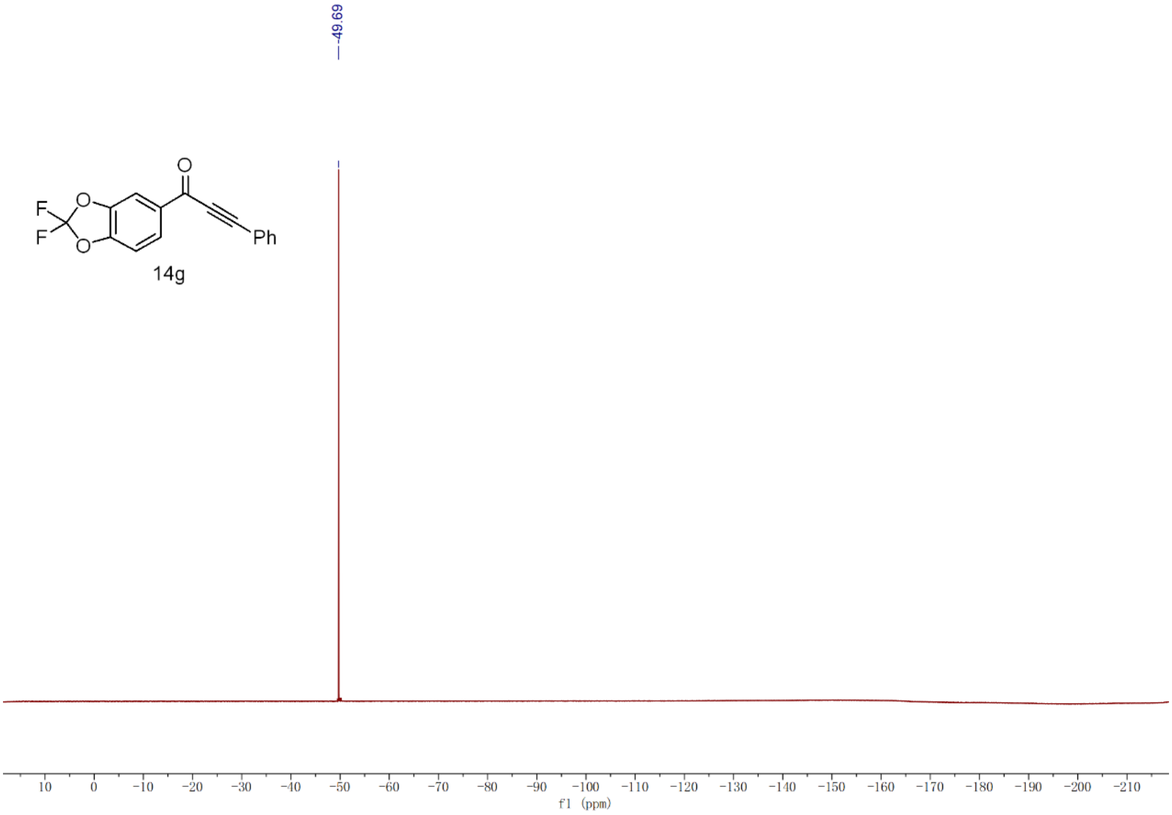

Supplementary Figure 91a | ^{1}H NMR (500 MHz, $CDCl_3$) of 3-Phenyl-1-(thiophen-2-yl)prop-2-yn-1-one (**14h**)

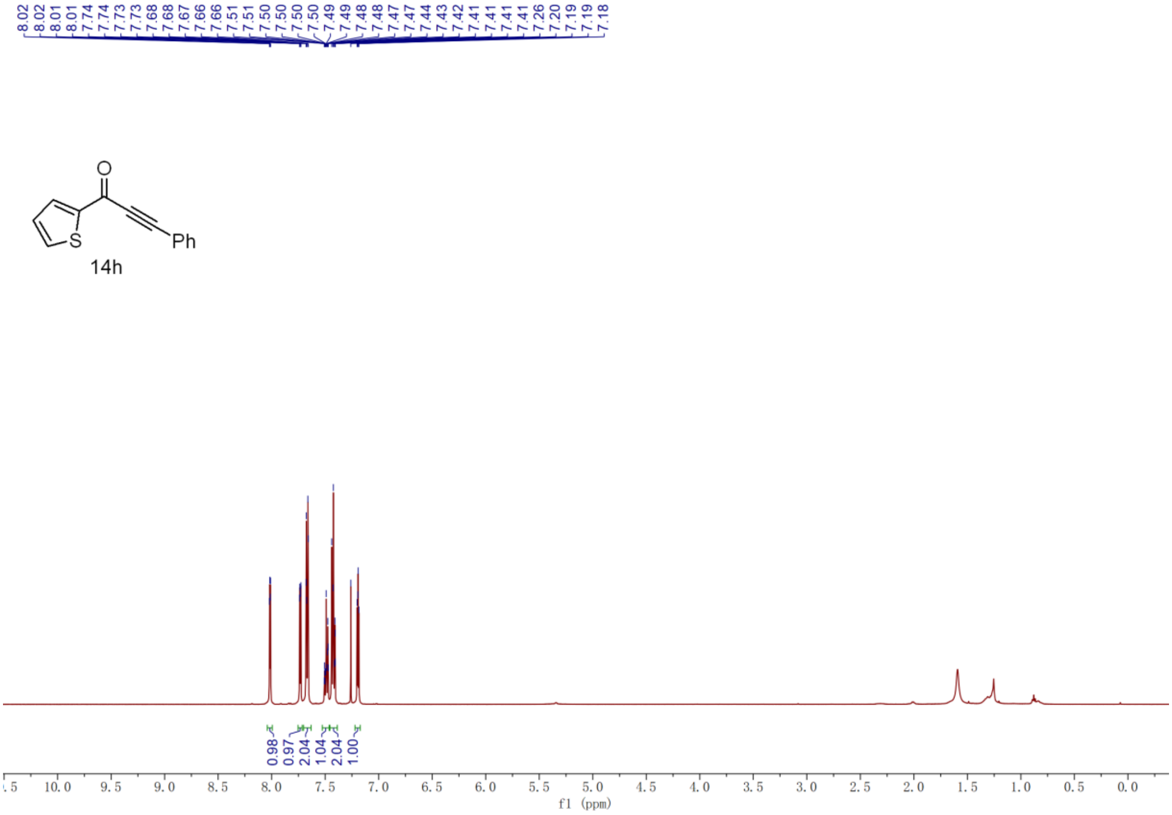

Supplementary Figure 91b | ^{13}C NMR (126 MHz, $CDCl_3$) of 3-Phenyl-1-(thiophen-2-yl)prop-2-yn-1-one (**14h**)

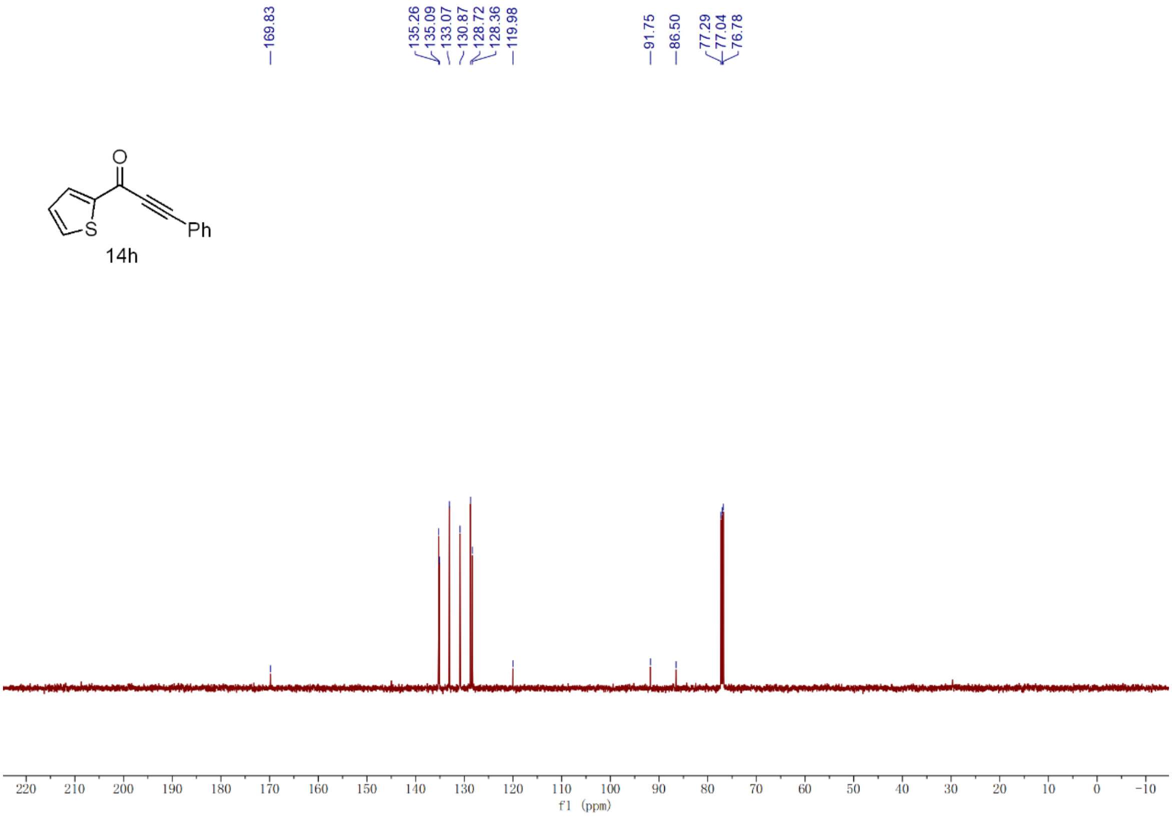

Supplementary Figure 92a | ^{1}H NMR (500 MHz, $CDCl_3$) of 1-Phenyloct-1-yn-3-one (**14i**)

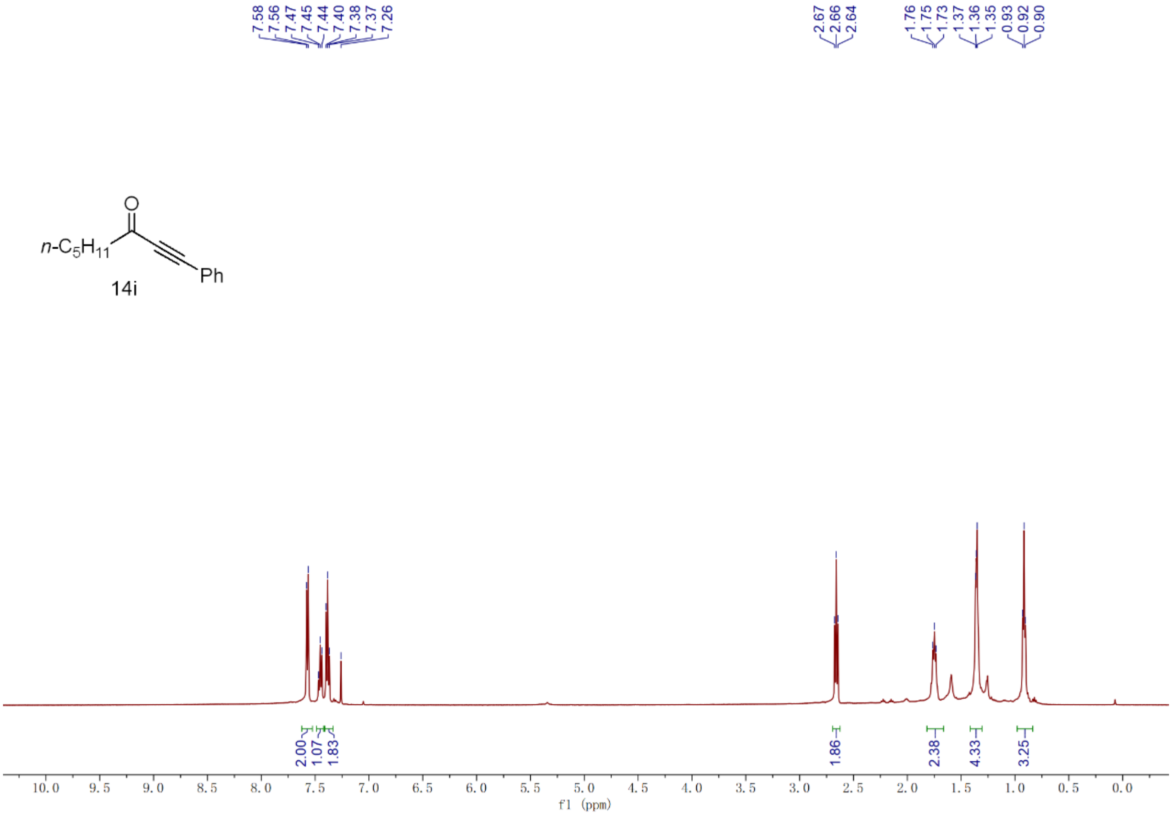

Supplementary Figure 92b | 13C NMR (126 MHz, $CDCl_3$) of 1-Phenyloct-1-yn-3-one (**14i**)

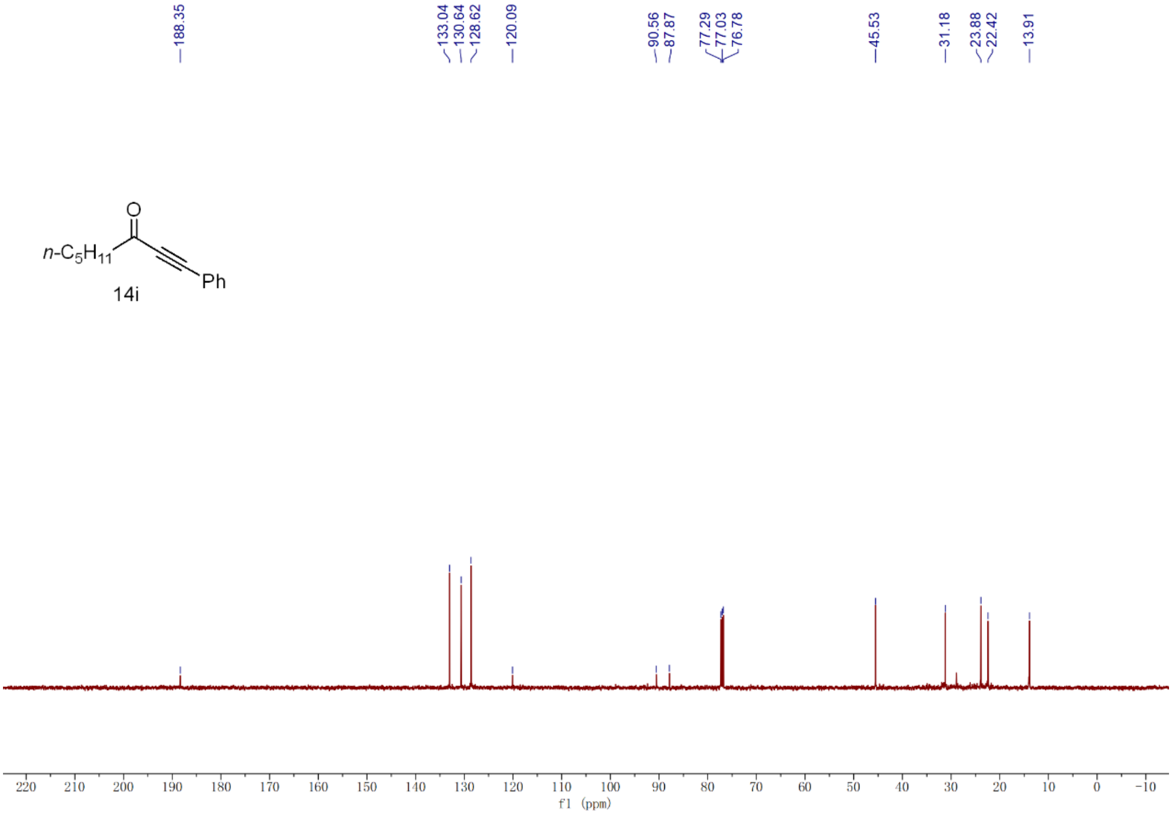

Supplementary Figure 93a | 1H NMR (500 MHz, $CDCl_3$) of 6-Oxo-8-phenyloct-7-yn-1-yl 4-methylbenzenesulfonate (**14j**)

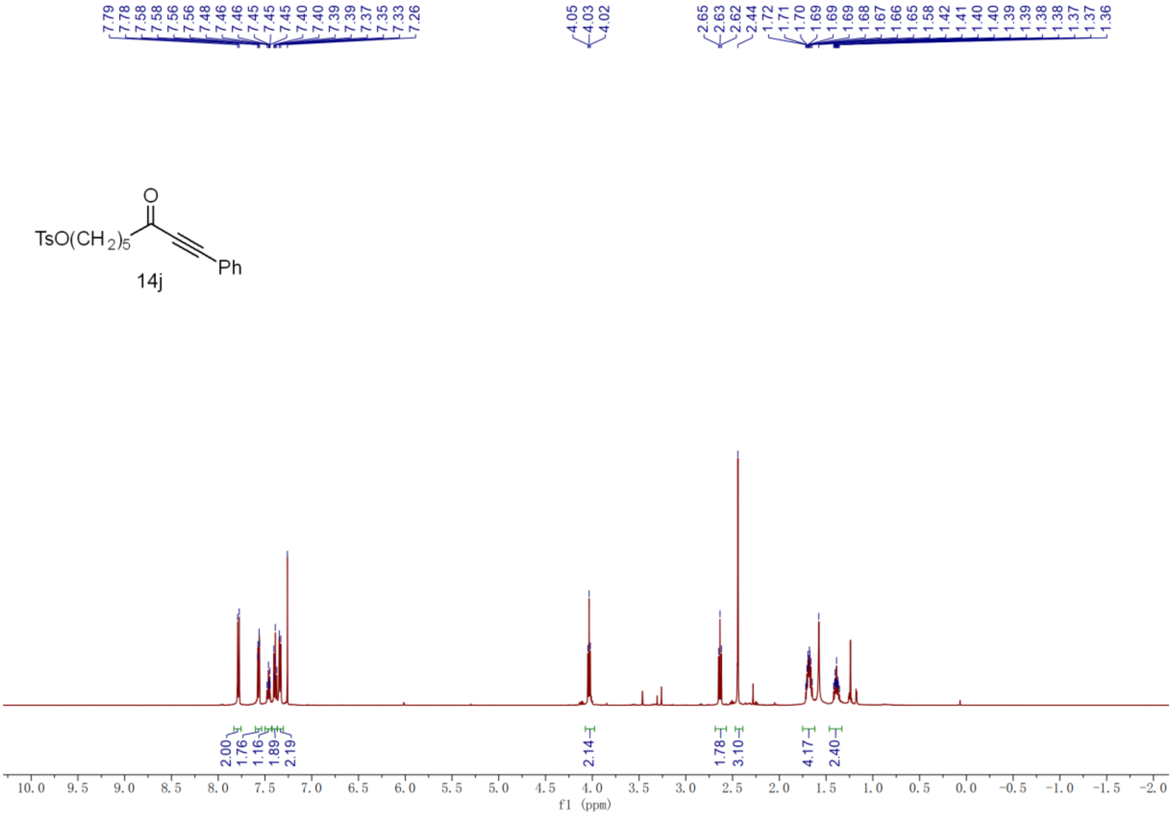

Supplementary Figure 93b | ^{13}C NMR (126 MHz, $CDCl_3$) of 6-Oxo-8-phenyloct-7-yn-1-yl 4-methylbenzenesulfonate (**14j**)

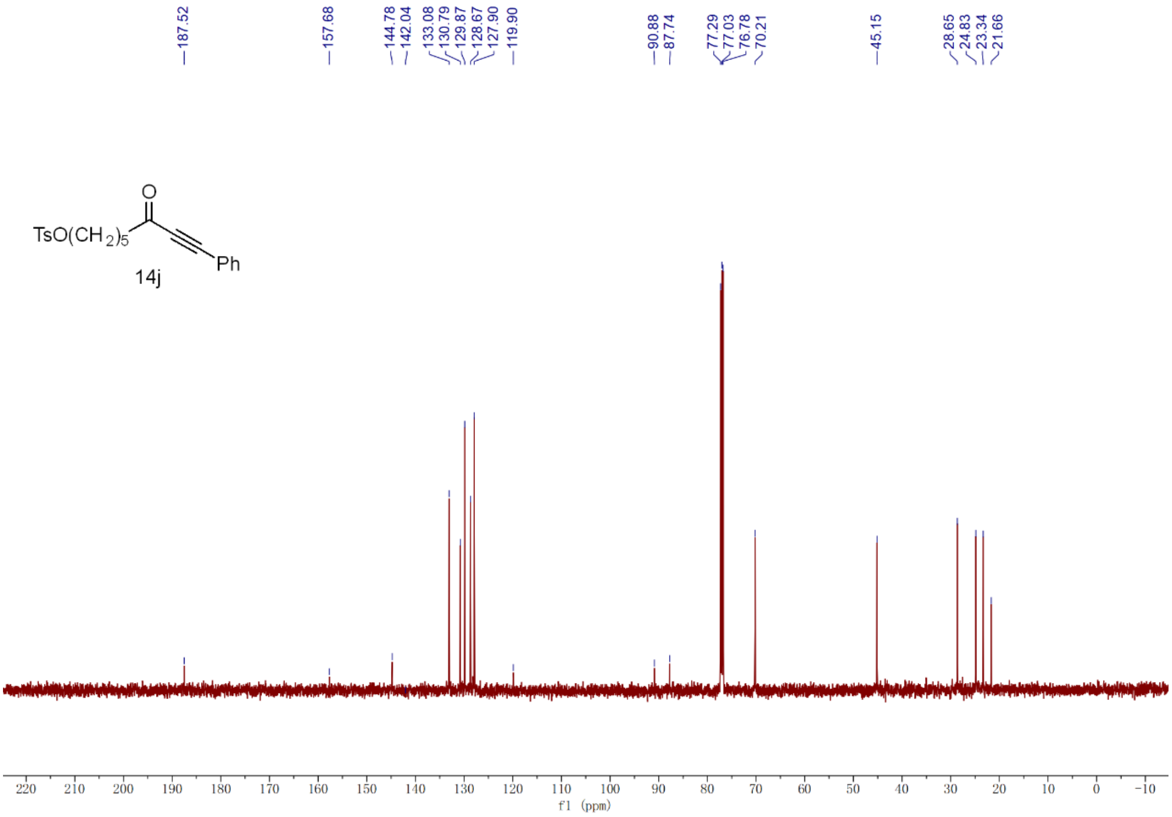

Supplementary Figure 94a | ^{1}H NMR (500 MHz, $CDCl_3$) of 4-Ethyl-1-phenyloct-1-yn-3-one (**14k**)

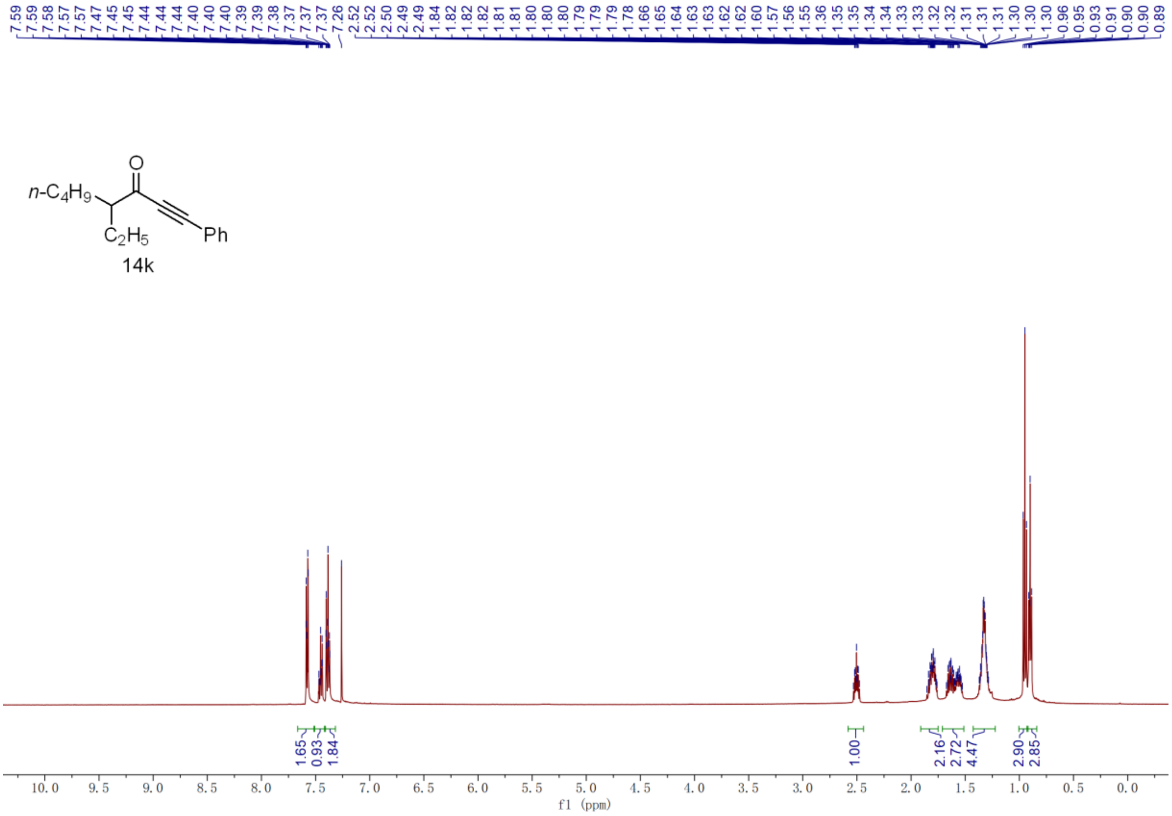

Supplementary Figure 94b | ^{13}C NMR (126 MHz, $CDCl_3$) of 4-Ethyl-1-phenyloct-1-yn-3-one (**14k**)

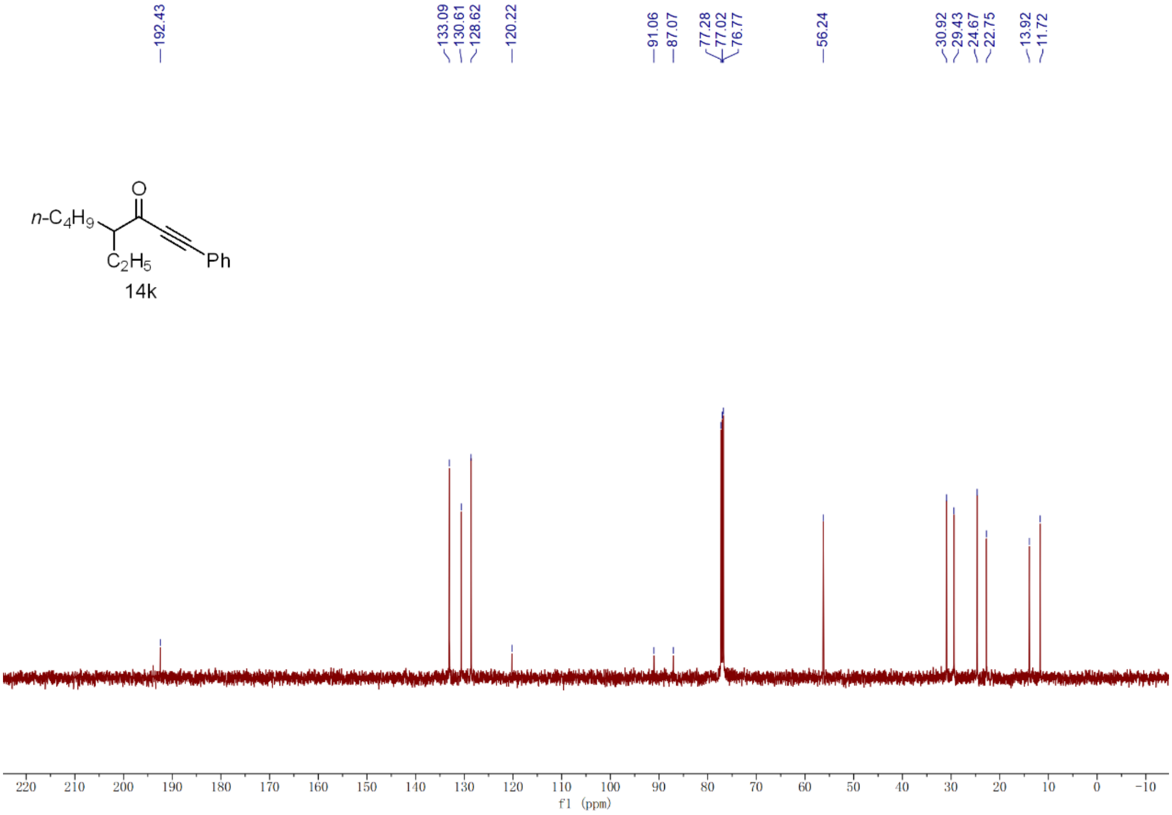

Supplementary Figure 95a | ^{1}H NMR (400 MHz, $CDCl_3$) of 1-Cyclopropyl-3-phenylprop-2-yn-1-one (**14l**)

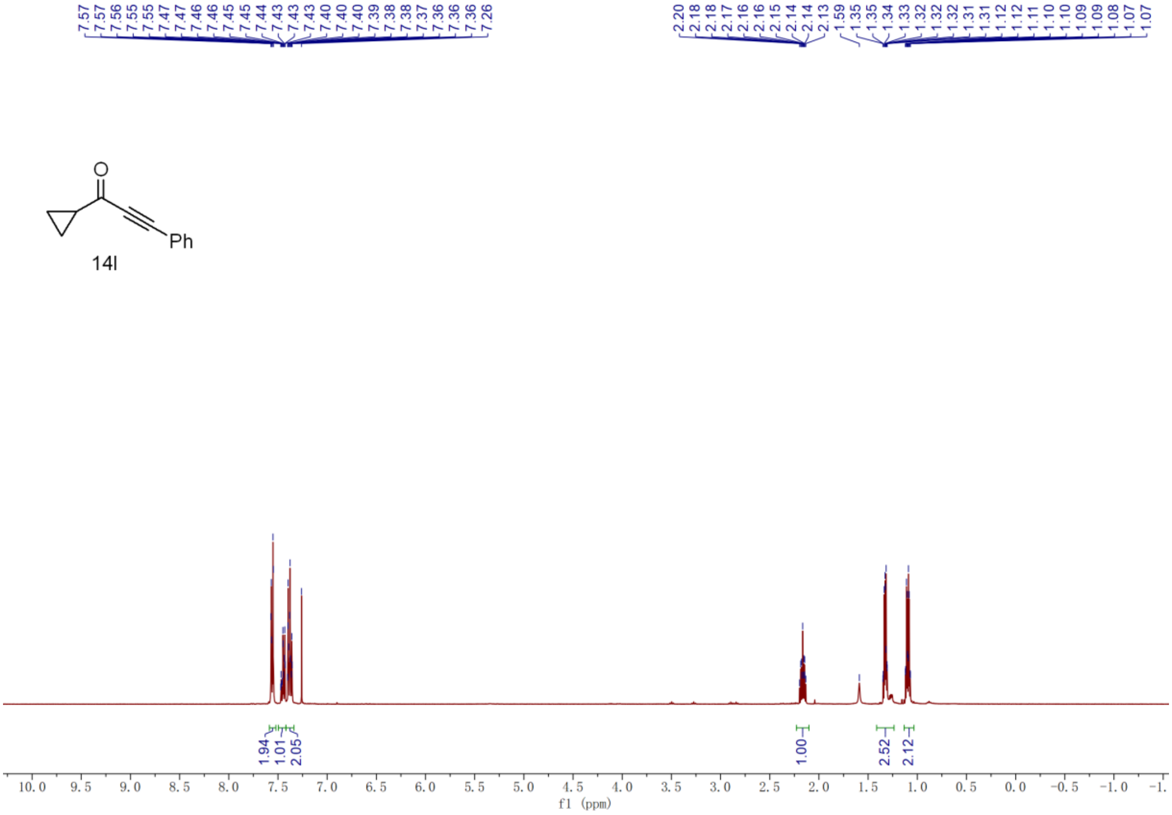

Supplementary Figure 95b | ^{13}C NMR (126 MHz, $CDCl_3$) of 1-Cyclopropyl-3-phenylprop-2-yn-1-one (**14l**)

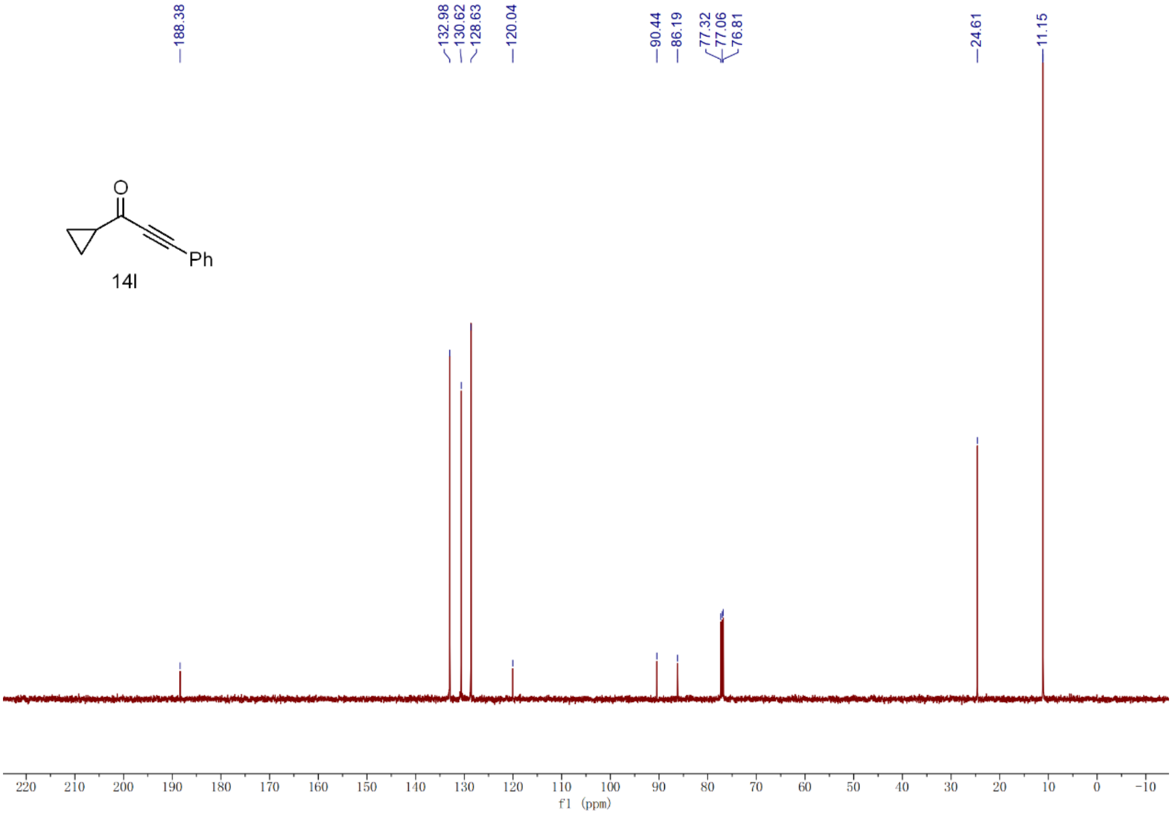

Supplementary Figure 96a | ^{1}H NMR (500 MHz, $CDCl_3$) of 1-Cyclohexyl-3-phenylprop-2-yn-1-one (**14m**)

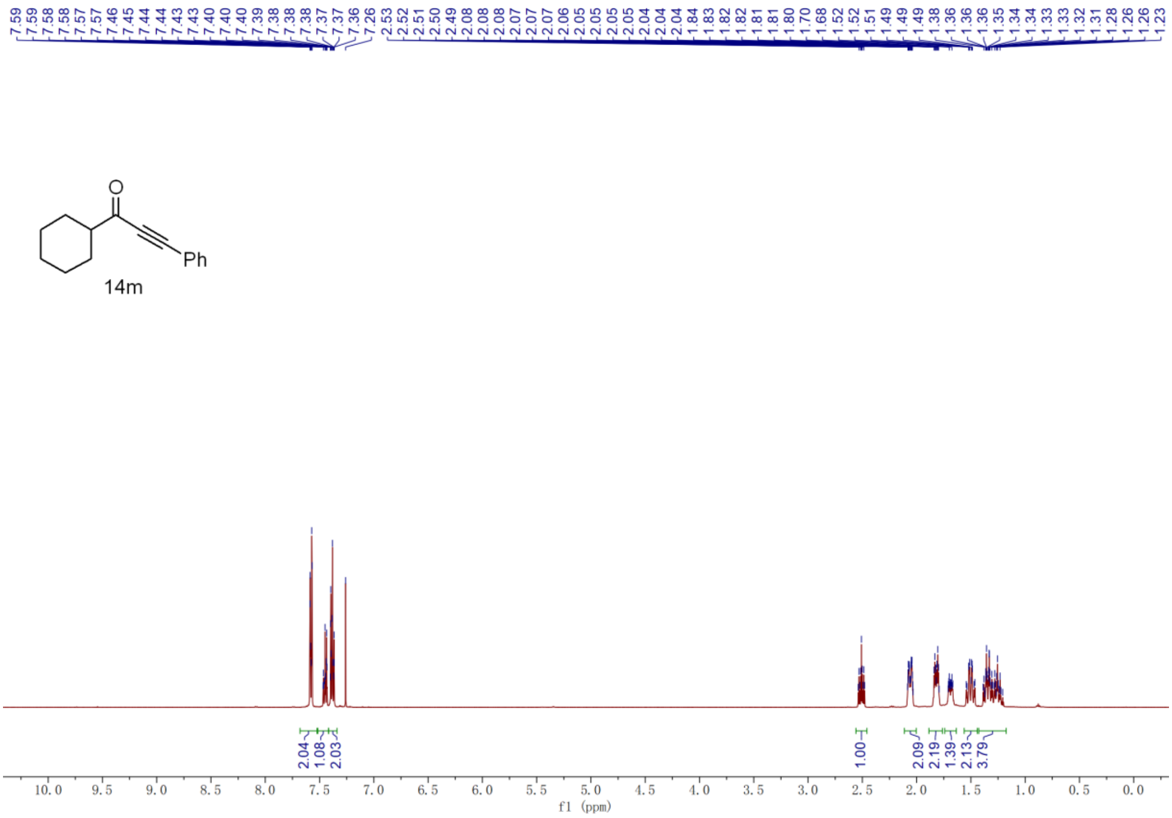

Supplementary Figure 96b | ^{13}C NMR (126 MHz, $CDCl_3$) of 1-Cyclohexyl-3-phenylprop-2-yn-1-one (**14m**)

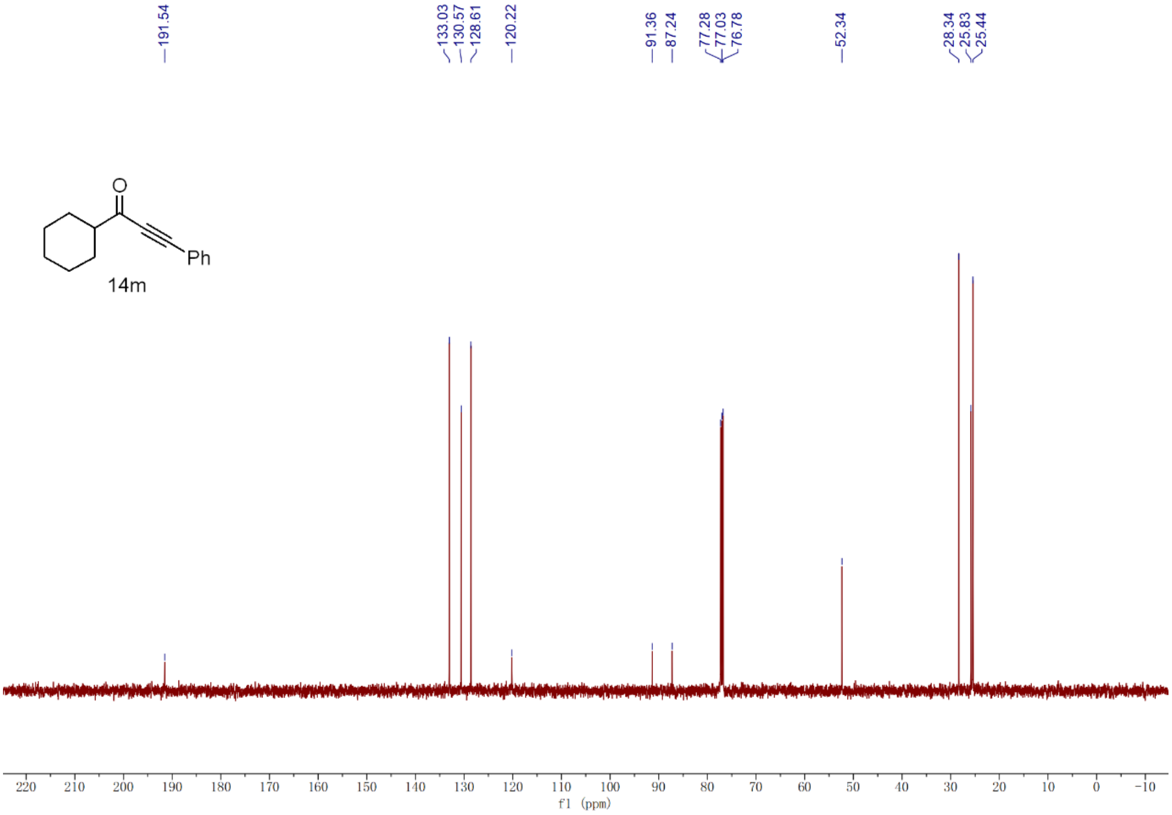

Supplementary Figure 97a | ^{1}H NMR (500 MHz, $CDCl_3$) of (5*R*,8*R*,9*S*,10*S*,13*R*,14*S*,17*R*)-10,13-Dimethyl-17-((*R*)-5-oxo-7-phenylhept-6-yn-2-yl)hexadecahydro-3H-cyclopenta[a]phenanthren-3-one (**14n**)

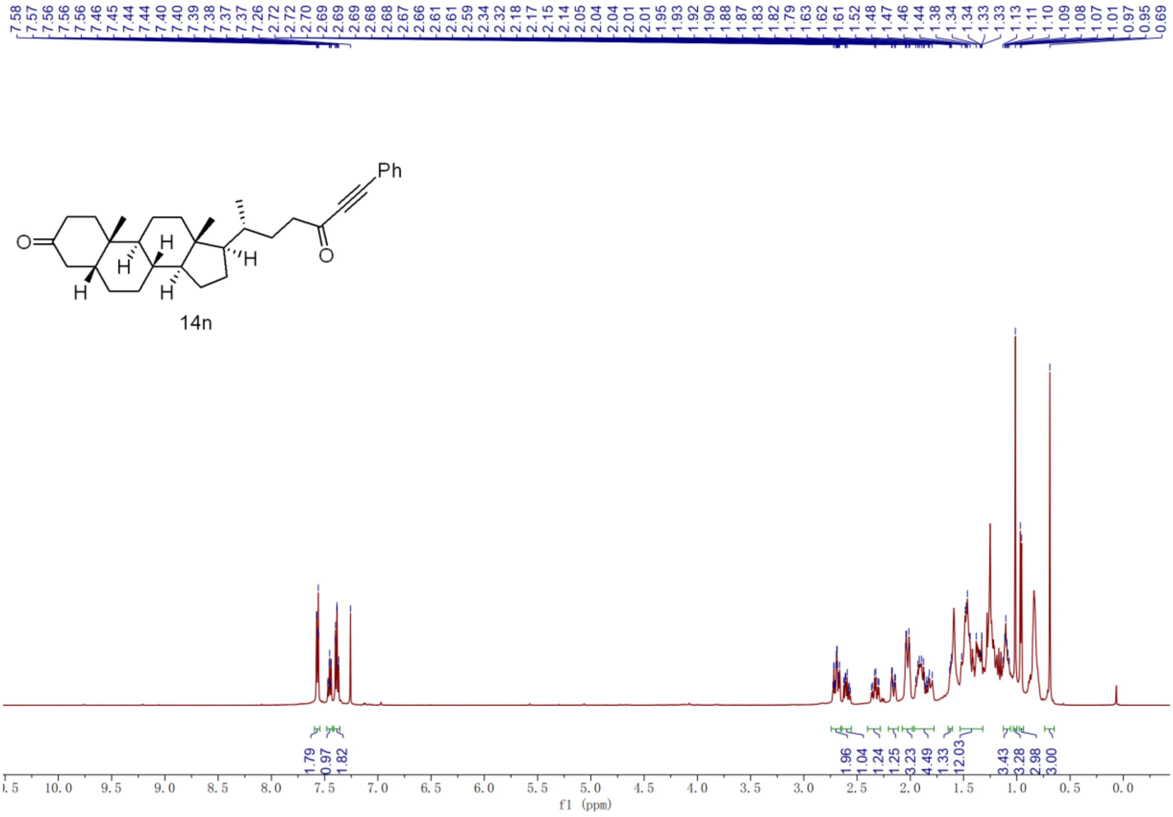

Supplementary Figure 97b | ^{13}C NMR (126 MHz, $CDCl_3$) of (5*R*,8*R*,9*S*,10*S*,13*R*,14*S*,17*R*)-10,13-Dimethyl-17-((*R*)-5-oxo-7-phenylhept-6-yn-2-yl)hexadecahydro-3H-cyclopenta[a]phenanthren-3-one (**14n**)

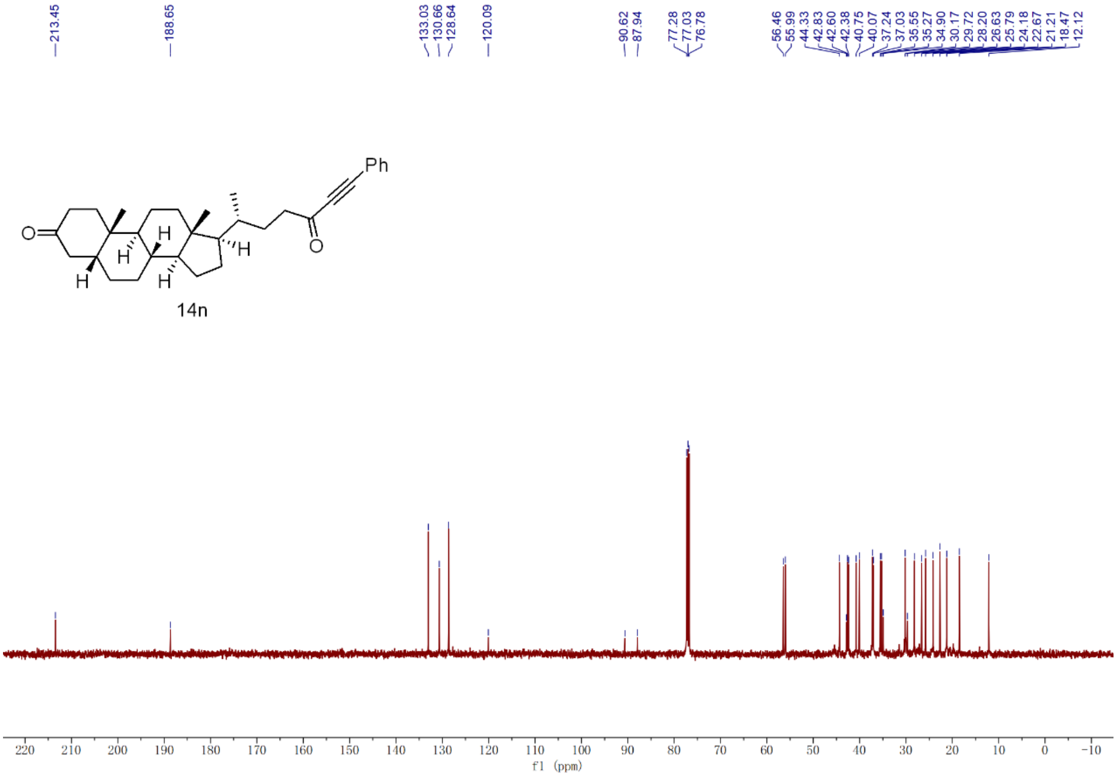

Supplementary Figure 98a | ^{1}H NMR (400 MHz, $CDCl_3$) of (3*S*,8*S*,9*S*,10*S*,13*R*,14*S*)-10,13-Dimethyl-16-((*S*)-6-methylheptan-2-yl)hexadecahydro-1H-cyclopenta[a]phenanthren-3-yl 4-(3-phenylpropioloyl)benzoate (**14o**)

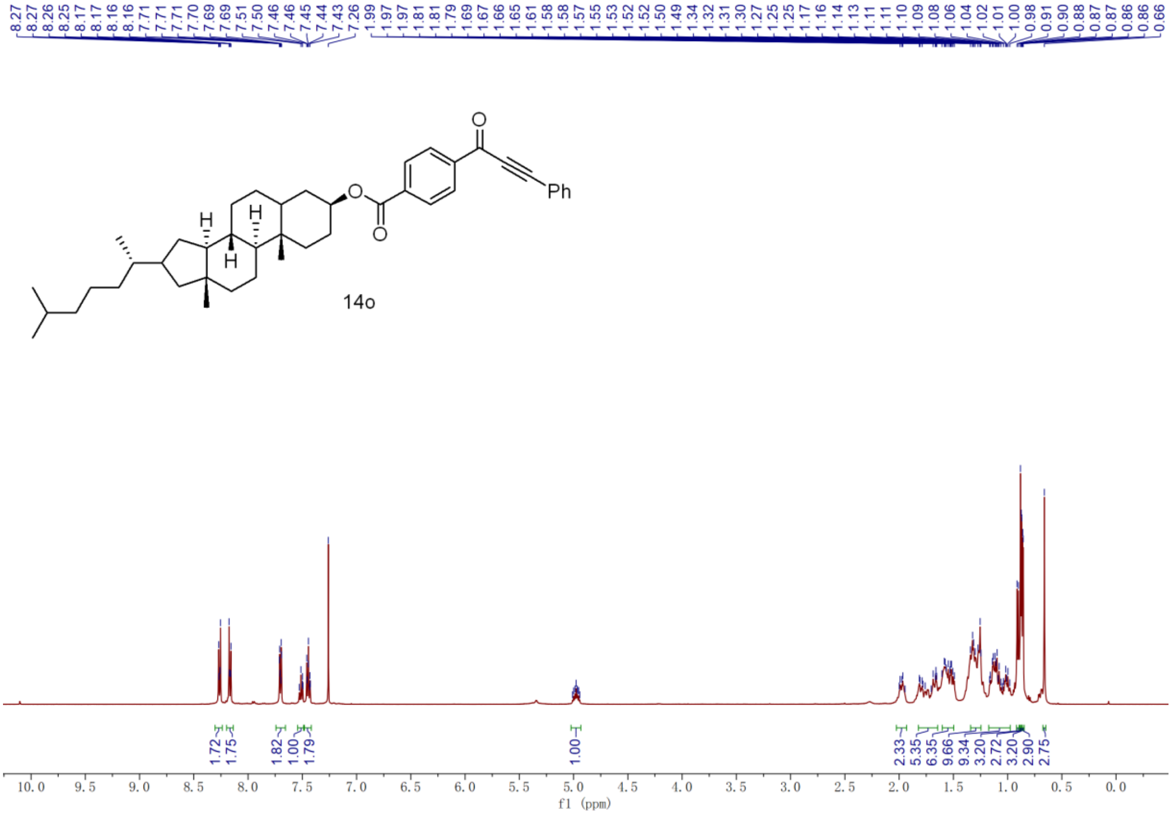

Supplementary Figure 98b | ^{13}C NMR (126 MHz, $CDCl_3$) of (3*S*,8*S*,9*S*,10*S*,13*R*,14*S*)-10,13-Dimethyl-16-((*S*)-6-methylheptan-2-yl)hexadecahydro-1H-cyclopenta[a]phenanthren-3-yl 4-(3-phenylpropioloyl)benzoate (**14o**)

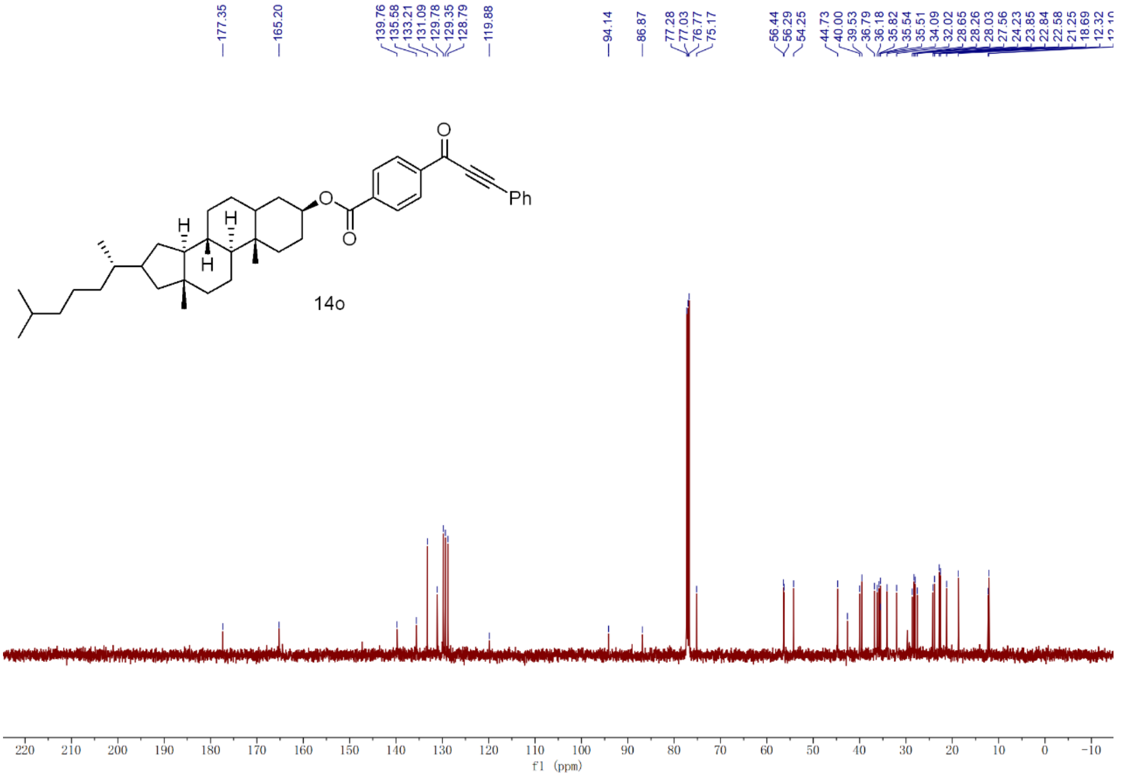

Supplementary Figure 99a | ^{1}H NMR (500 MHz, $CDCl_3$) of 1-Cyclopropyl-3-(p-tolyl)prop-2-yn-1-one (14p)

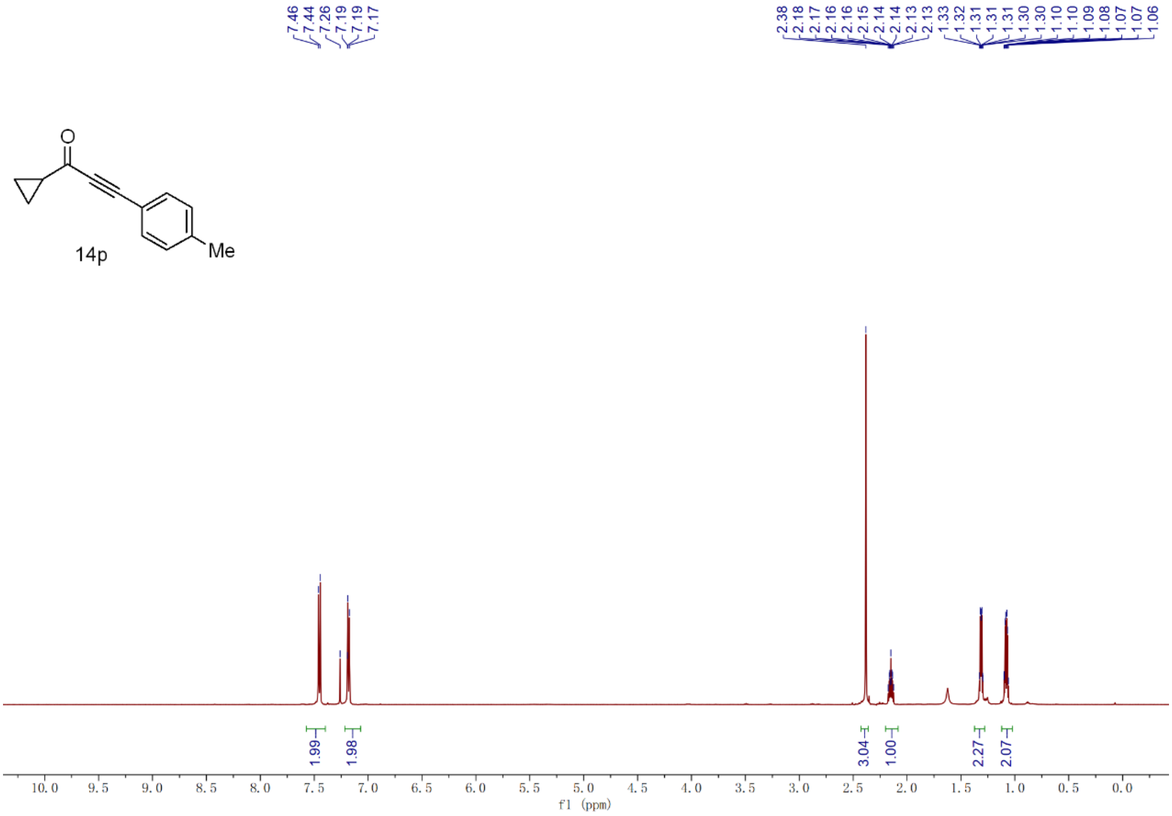

Supplementary Figure 99b | ^{13}C NMR (126 MHz, $CDCl_3$) of 1-Cyclopropyl-3-(p-tolyl)prop-2-yn-1-one (**14p**)

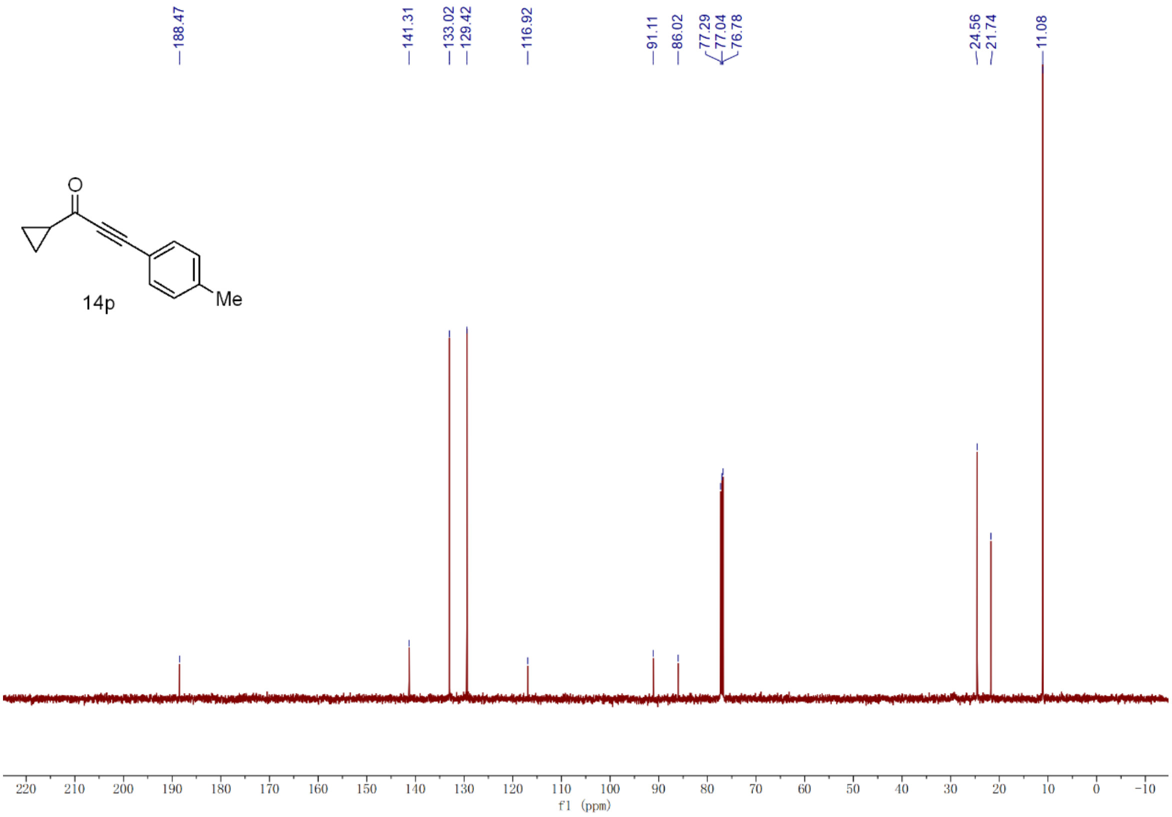

Supplementary Figure 100a | ^{1}H NMR (500 MHz, $CDCl_3$) of 3-([1,1'-Biphenyl]-4-yl)-1-cyclopropylprop-2-yn-1-one (**14q**)

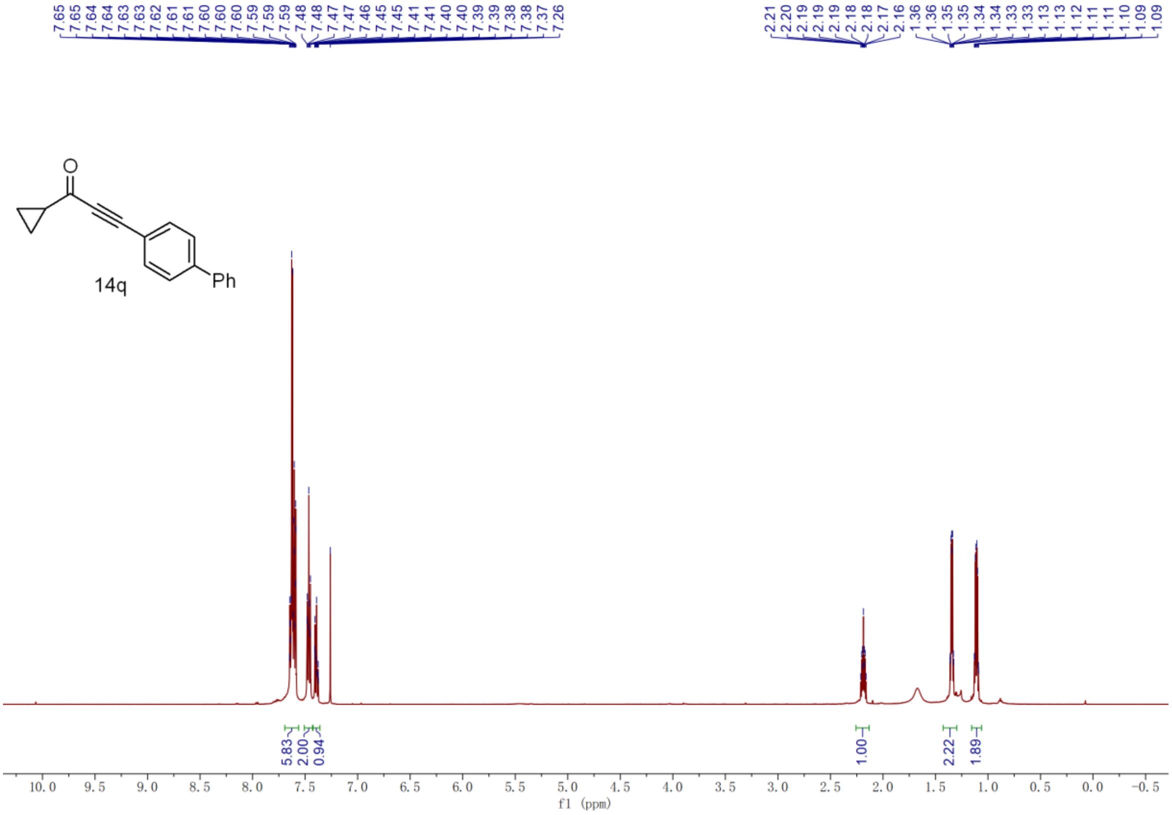

Supplementary Figure 100b | ^{13}C NMR (126 MHz, $CDCl_3$) of 3-([1,1'-Biphenyl]-4-yl)-1-cyclopropylprop-2-yn-1-one (**14q**)

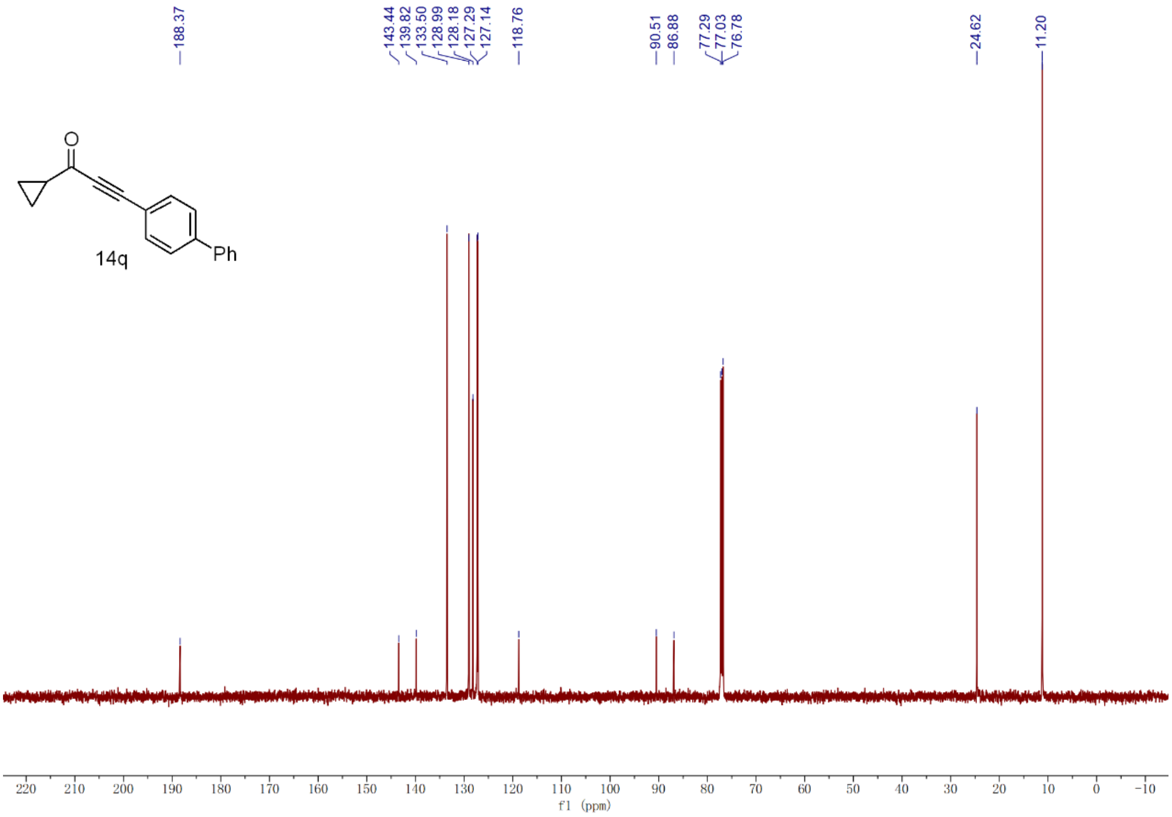

Supplementary Figure 101a | ^{1}H NMR (500 MHz, $CDCl_3$) of 3-(4-Chlorophenyl)-1-cyclopropylprop-2-yn-1-one (**14r**)

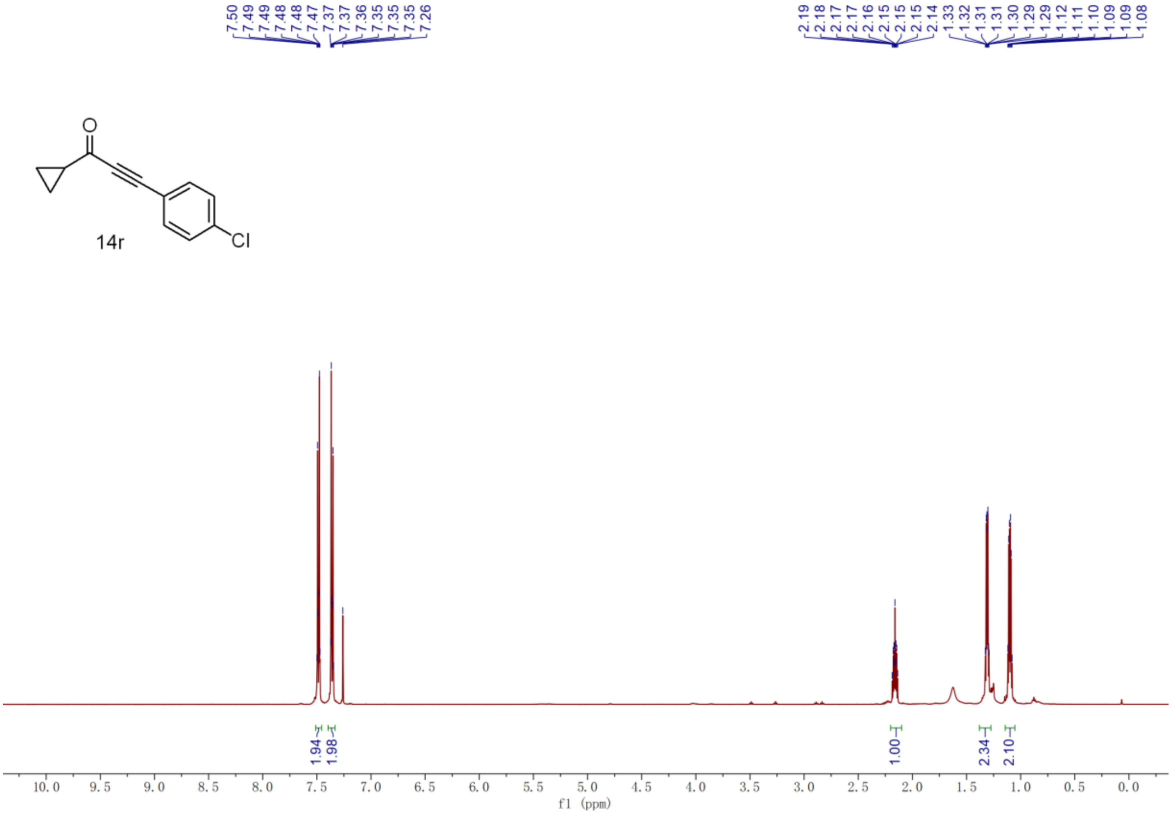

Supplementary Figure 101b | ^{13}C NMR (126 MHz, $CDCl_3$) of 3-(4-Chlorophenyl)-1-cyclopropylprop-2-yn-1-one (**14r**)

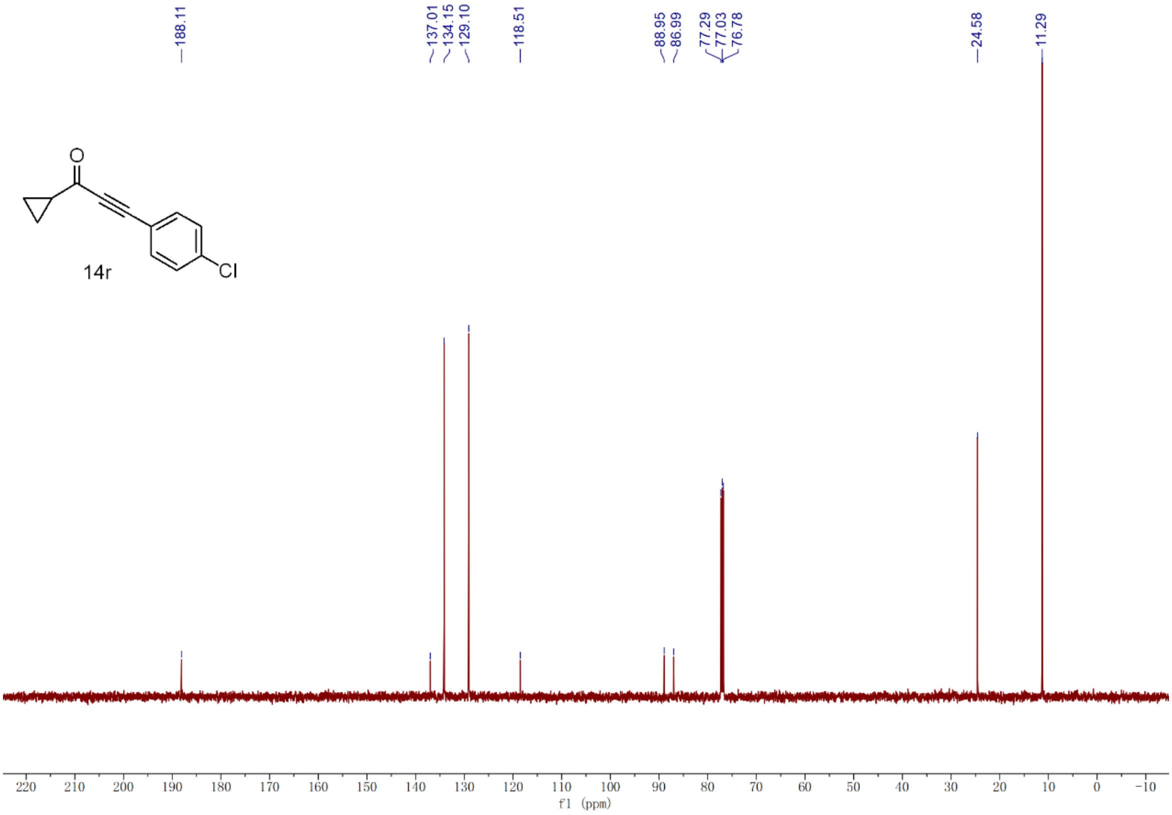

Supplementary Figure 102a | ^{1}H NMR (500 MHz, $CDCl_3$) of Methyl 4-(3-cyclopropyl-3-oxoprop-1-yn-1-yl)benzoate (**14s**)

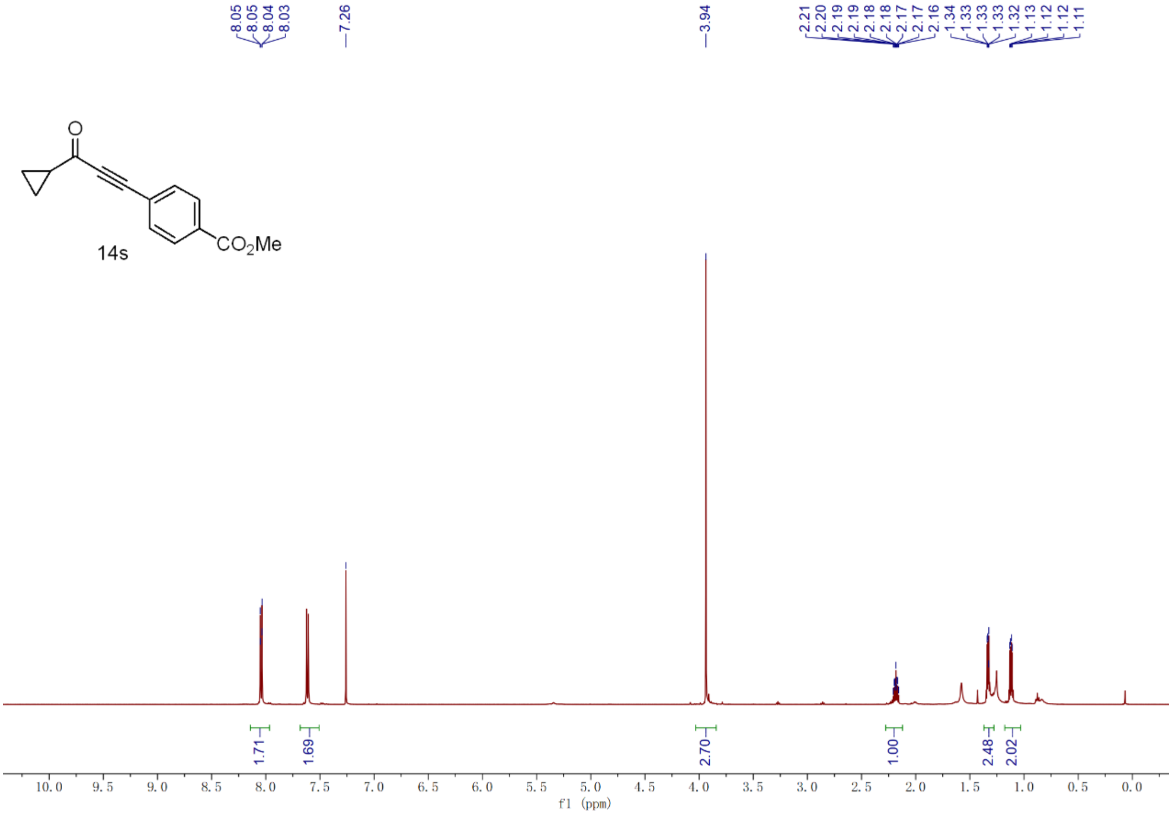

Supplementary Figure 102b | ^{13}C NMR (126 MHz, $CDCl_3$) of Methyl 4-(3-cyclopropyl-3-oxoprop-1-yn-1-yl)benzoate (**14s**)

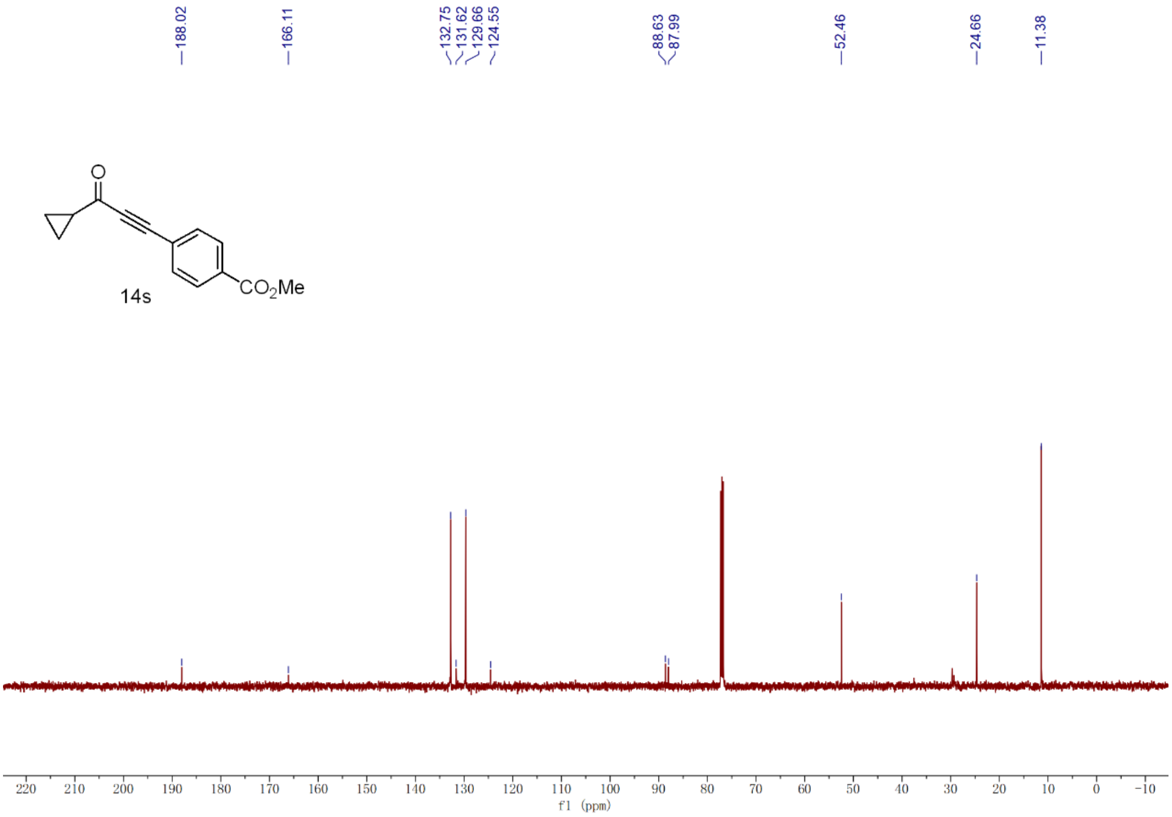

Supplementary Figure 103a | ^{1}H NMR (500 MHz, $CDCl_3$) of 1-Cyclopropyl-3-(o-tolyl)prop-2-yn-1-one (**14t**)

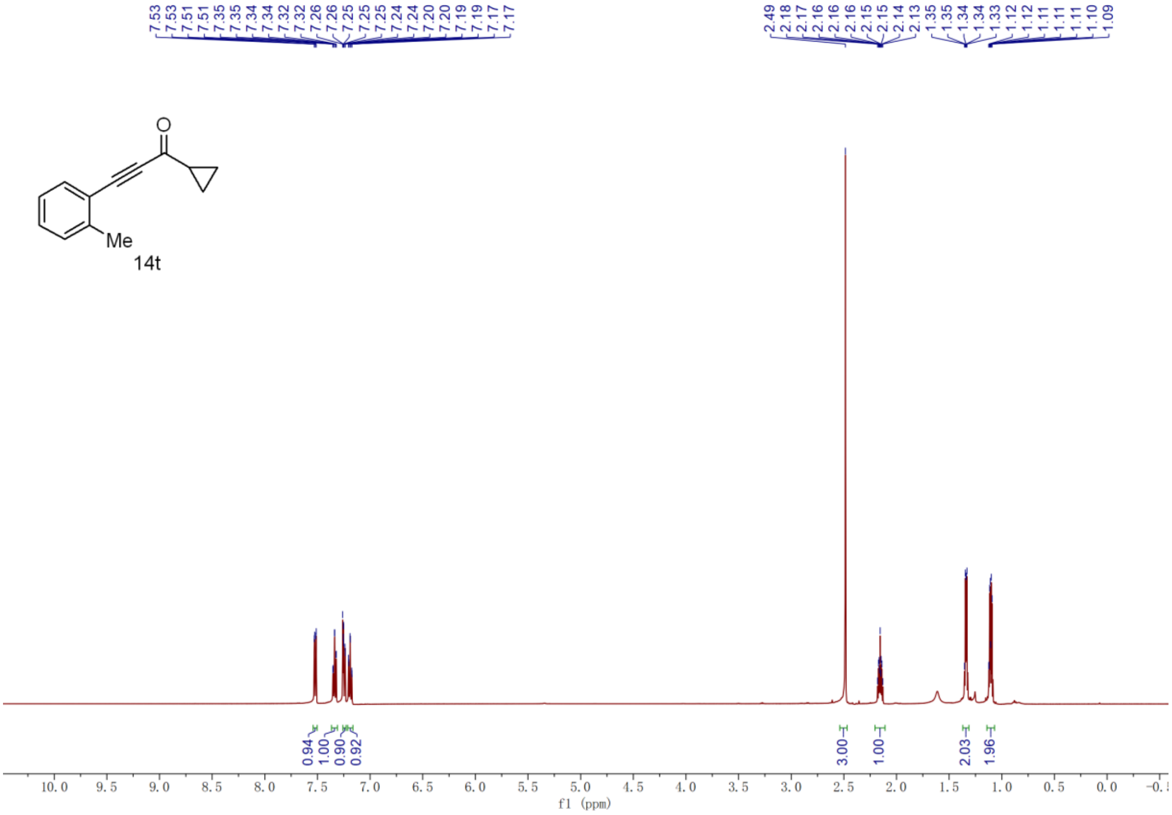

Supplementary Figure 103b | ^{13}C NMR (126 MHz, $CDCl_3$) of 1-Cyclopropyl-3-(o-tolyl)prop-2-yn-1-one (**14t**)

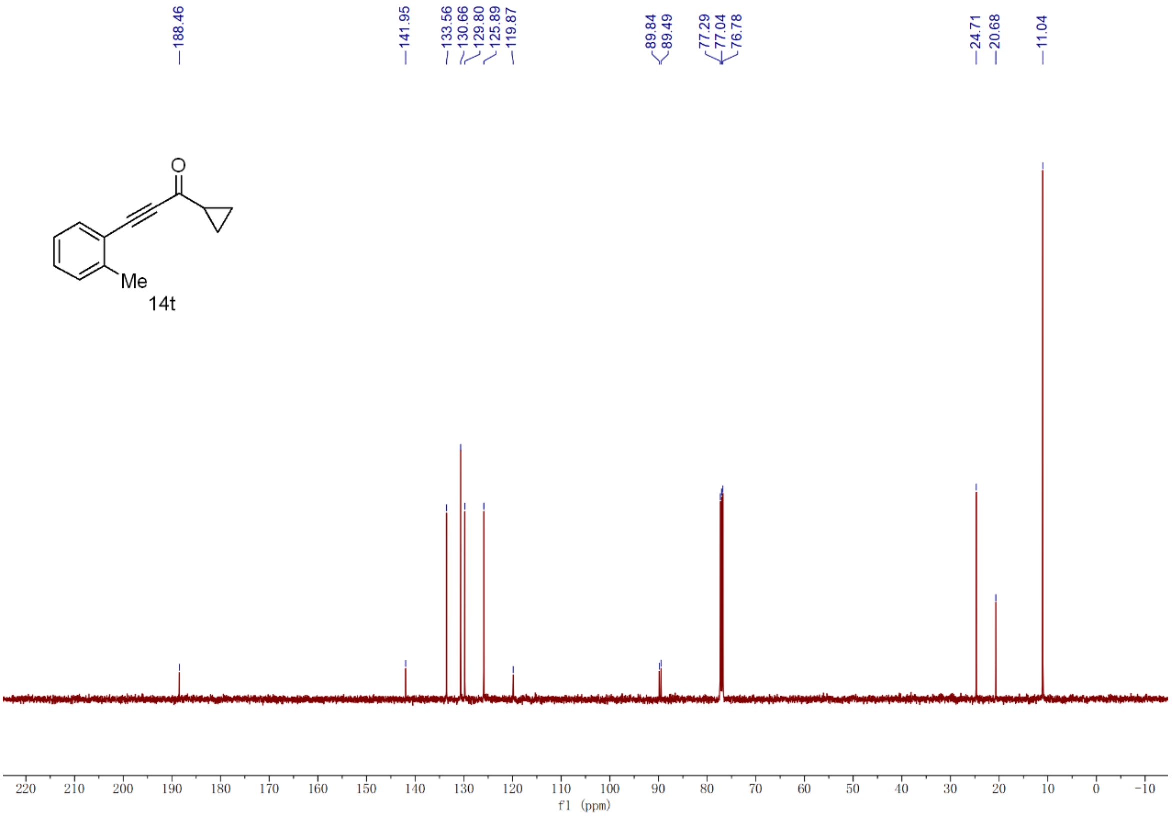

Supplementary Figure 104a | ^{1}H NMR (500 MHz, $CDCl_3$) of 1-Cyclopropyl-3-(2-methoxyphenyl)prop-2-yn-1-one (**14u**)

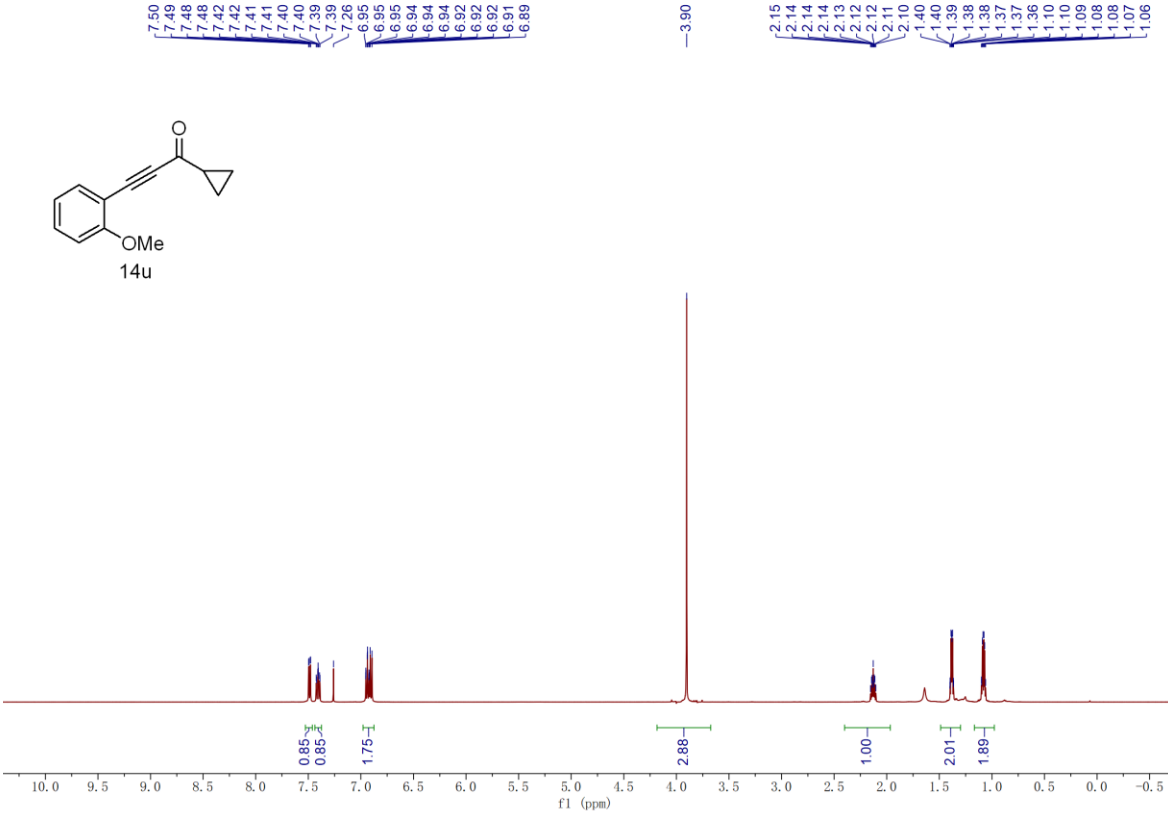

Supplementary Figure 104b | ^{13}C NMR (126 MHz, $CDCl_3$) of 1-Cyclopropyl-3-(2-methoxyphenyl)prop-2-yn-1-one (**14u**)

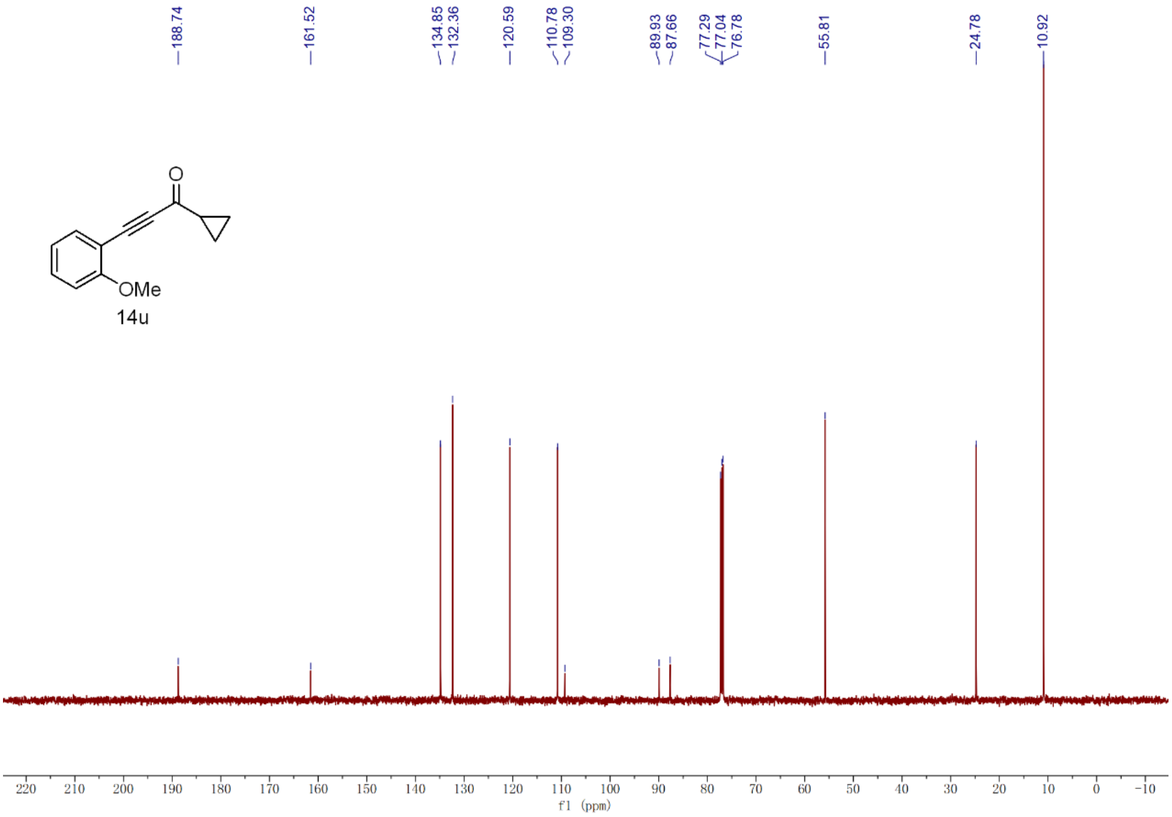

Supplementary Figure 105a | ^{1}H NMR (500 MHz, $CDCl_3$) of 1-Cyclopropyl-3-(thiophen-3-yl)prop-2-yn-1-one (**14v**)

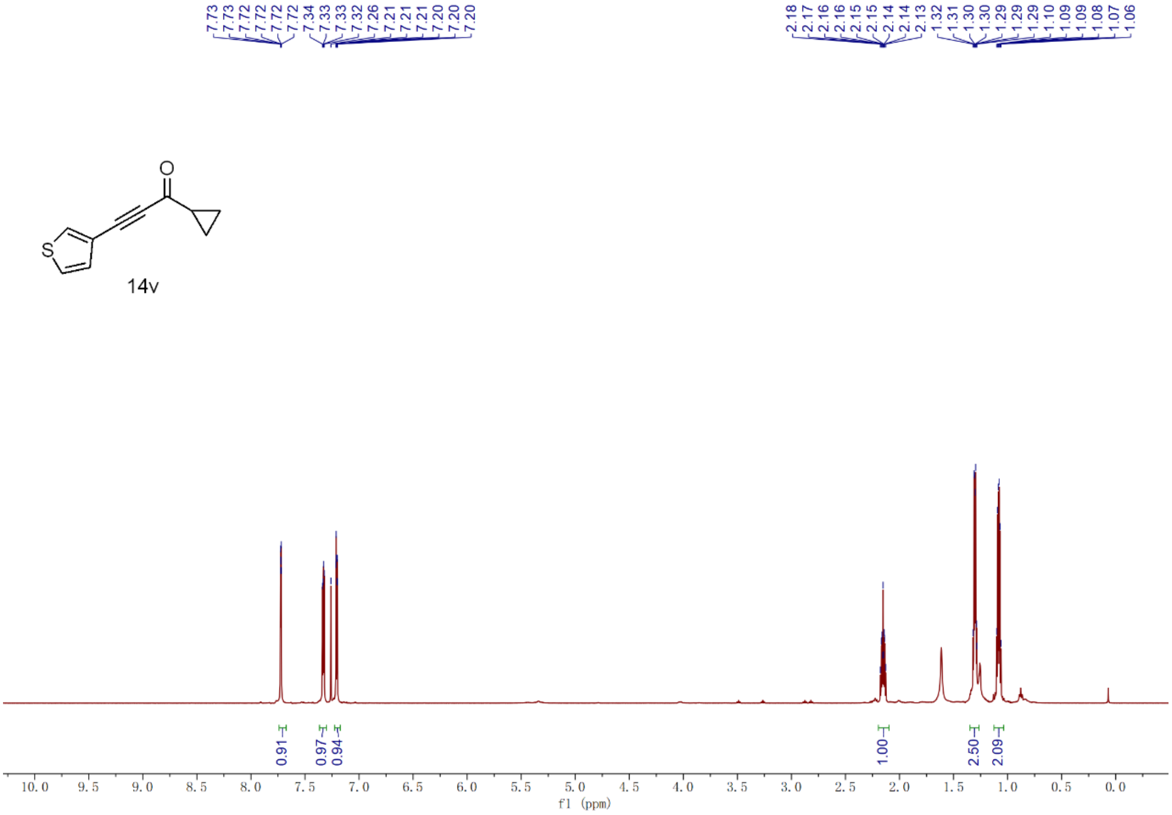

Supplementary Figure 105b | ^{1}H NMR (500 MHz, $CDCl_3$) of 1-Cyclopropyl-3-(thiophen-3-yl)prop-2-yn-1-one (**14v**)

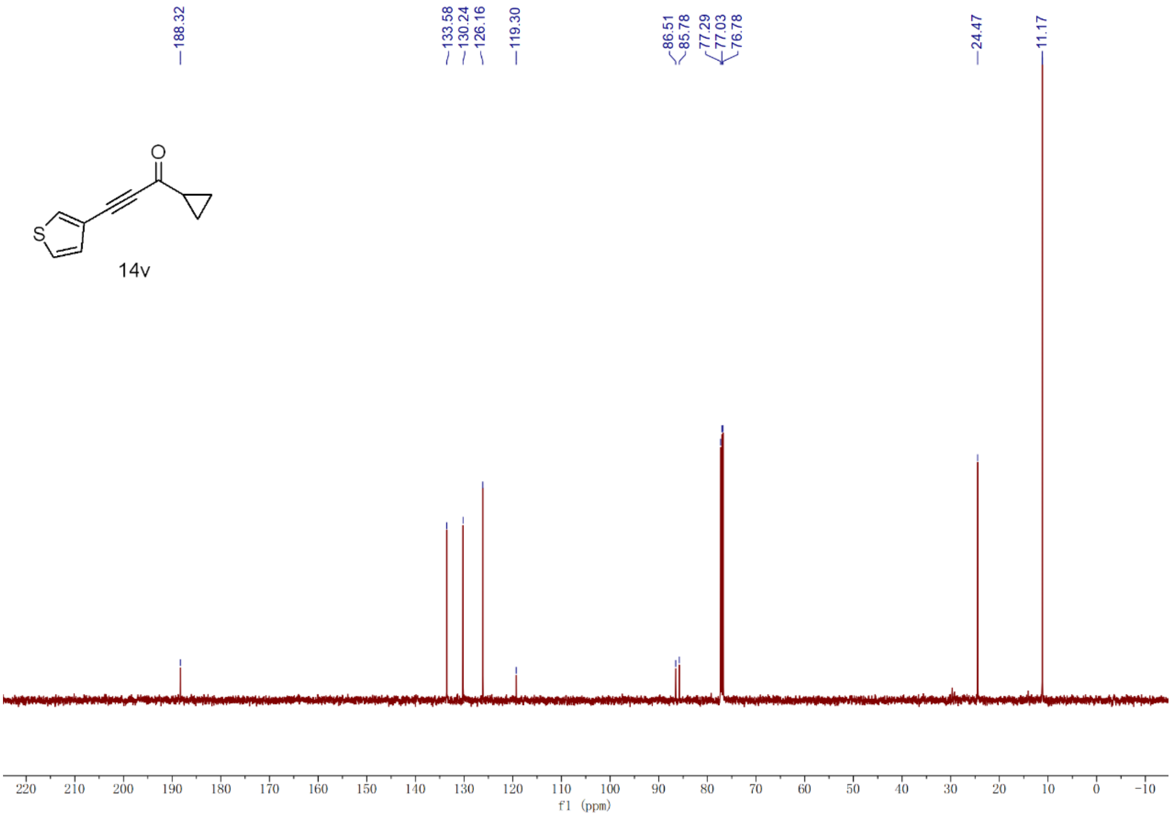

Supplementary Figure 106a | ^{13}C NMR (126 MHz, $CDCl_3$) of (3,3-dimethylbut-1-yn-1-yl)benzene (**14w**)

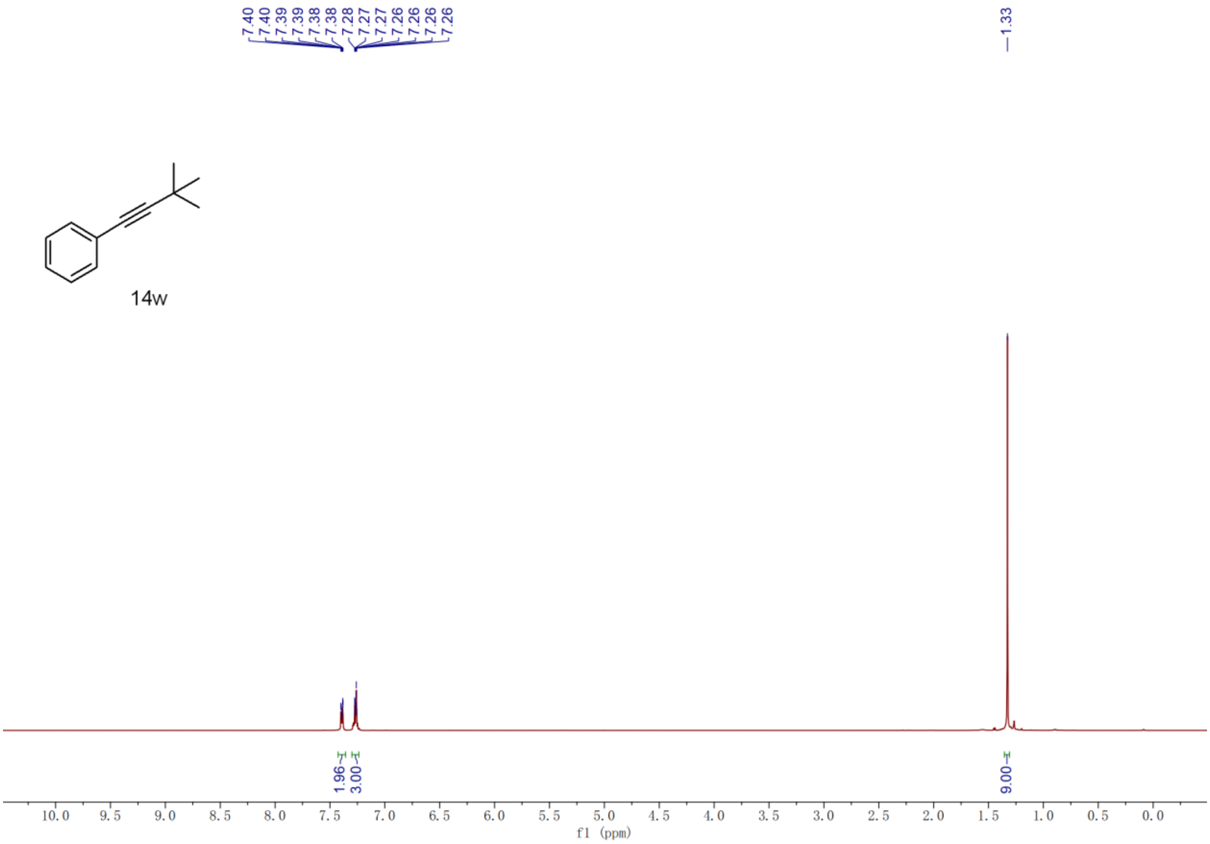

Supplementary Figure 106b | ^{13}C NMR (126 MHz, $CDCl_3$) of (3,3-dimethylbut-1-yn-1-yl)benzene (**14w**)

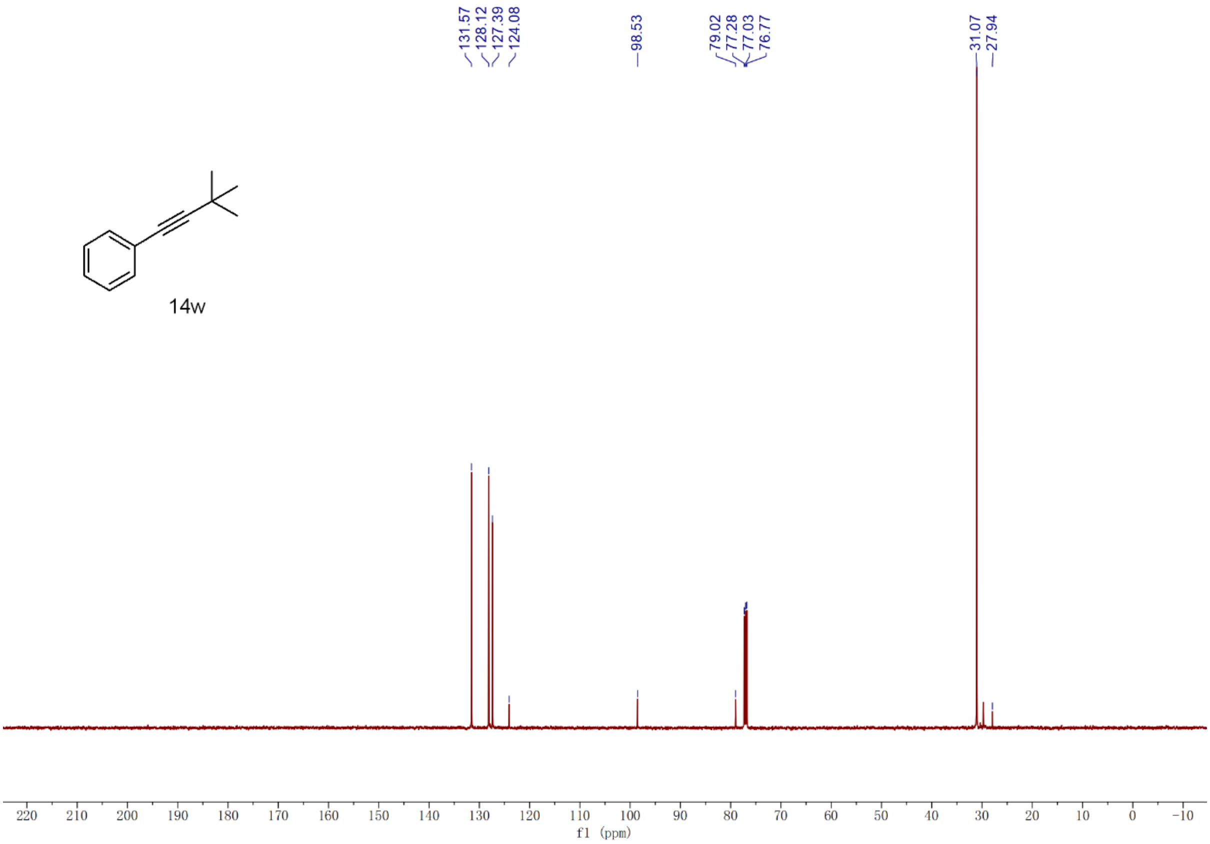

Supplementary Figure 107a | ^{1}H NMR (400 MHz, $CDCl_3$) of [1,1'-Biphenyl]-4-carbonyl azide (**16a**)

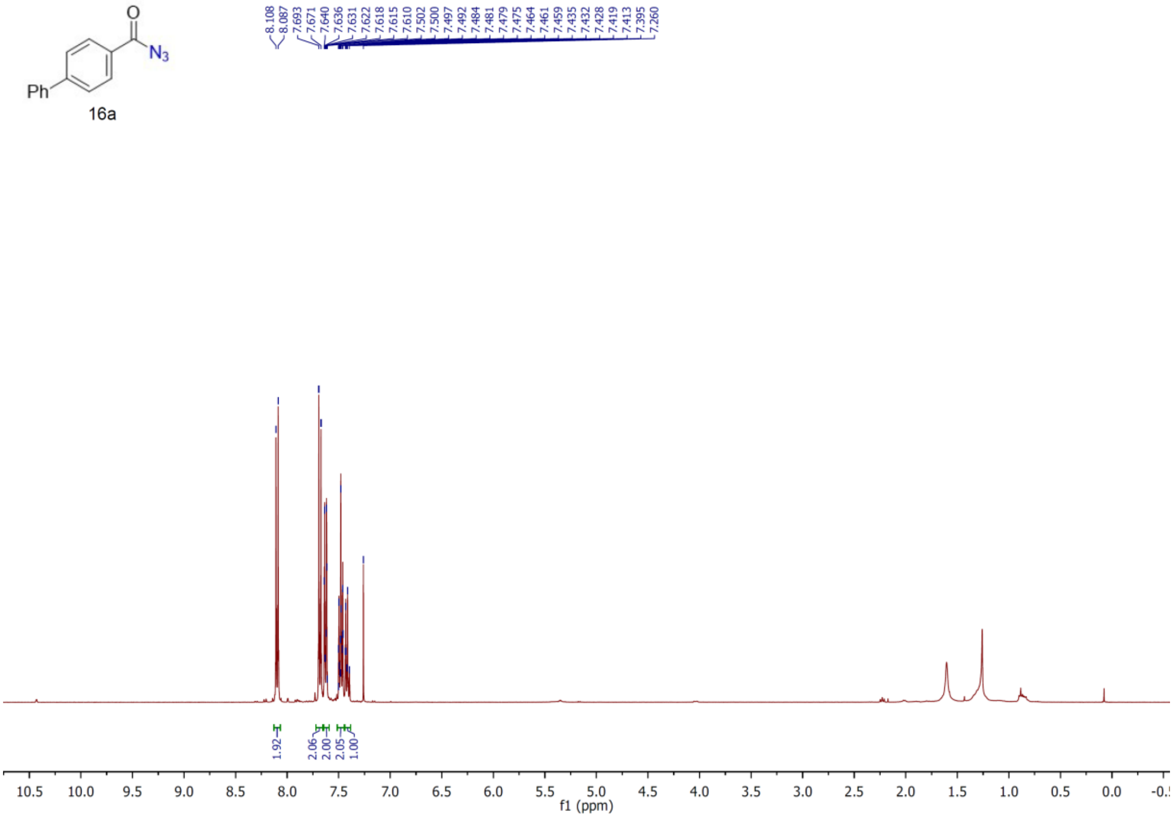

Supplementary Figure 107b | ^{13}C NMR (126 MHz, $CDCl_3$) of [1,1'-Biphenyl]-4-carbonyl azide (**16a**)

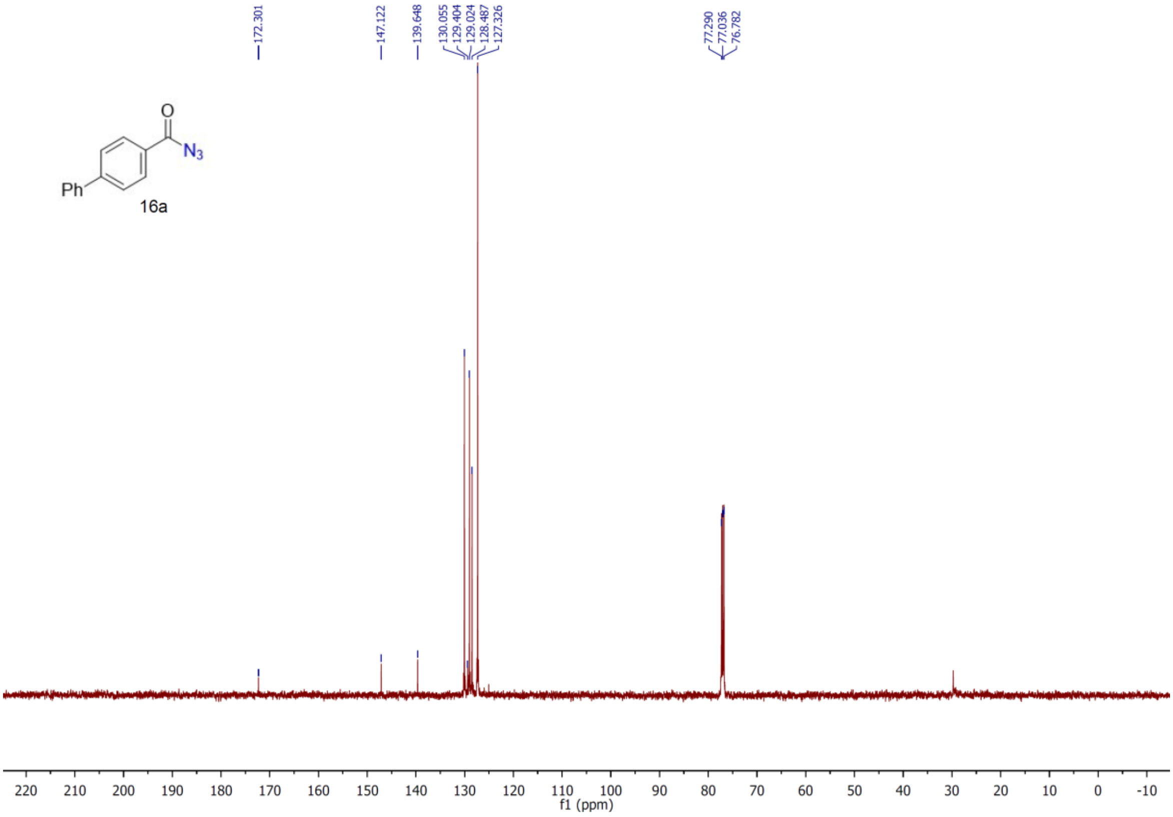

Supplementary Figure 108a | ^{1}H NMR (400 MHz, $CDCl_3$) of 4-Methoxybenzoyl azide (**16b**)

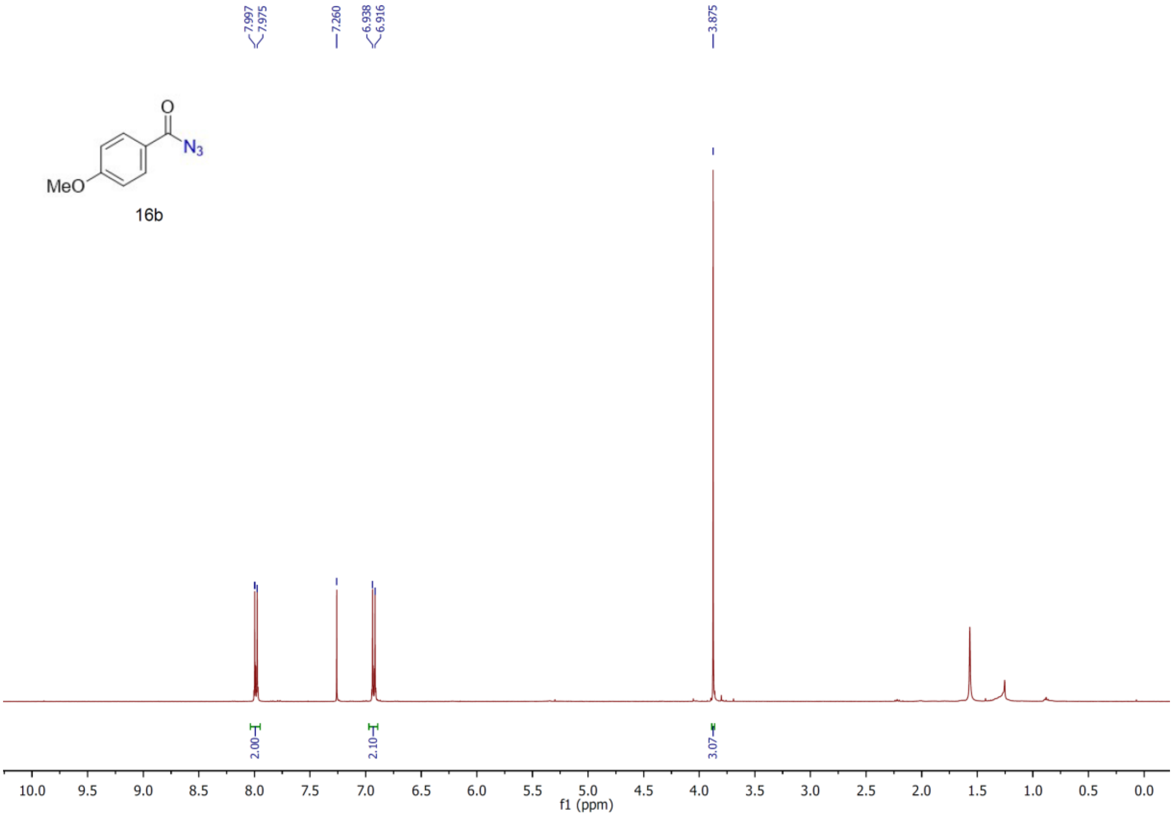

Supplementary Figure 108b | ^{13}C NMR (126 MHz, $CDCl_3$) of 4-Methoxybenzoyl azide (**16b**)

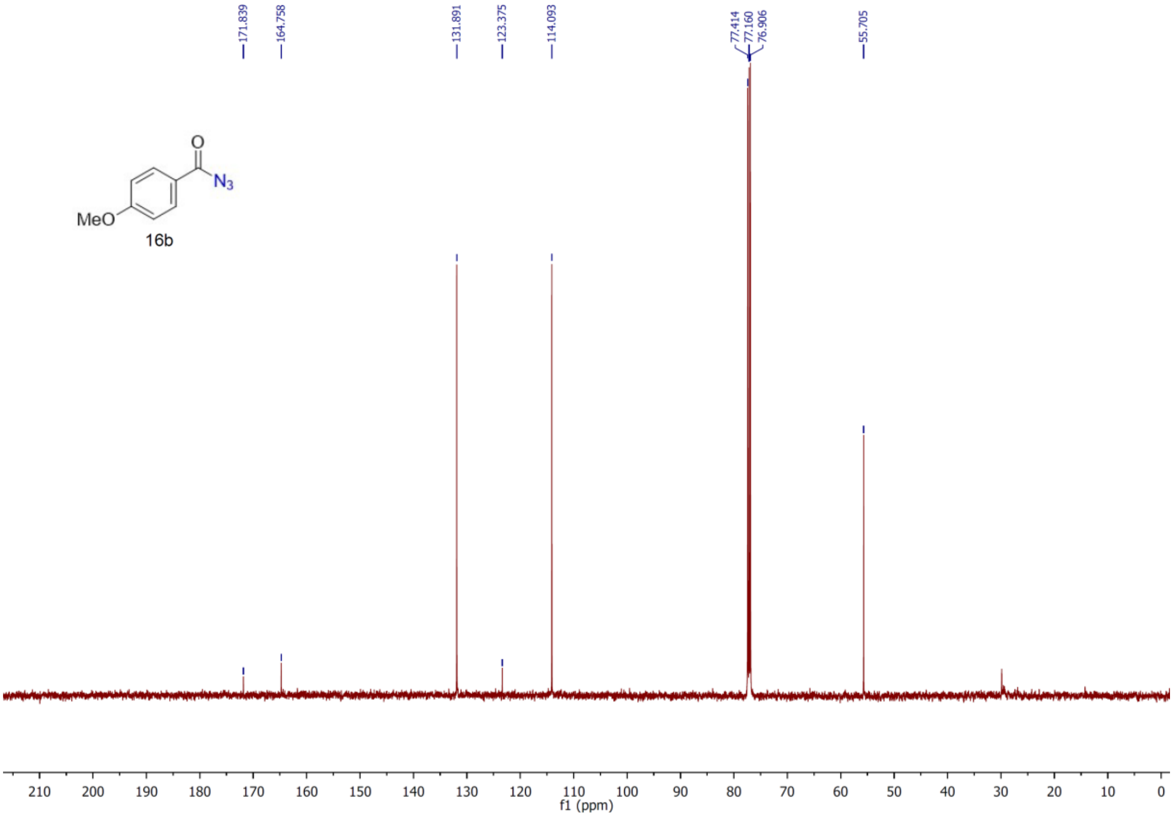

Supplementary Figure 109a | ^{1}H NMR (400 MHz, $CDCl_3$) of (*E*)-1-(4-Methoxyphenyl)-3-(phenylsulfonyl)prop-2-en-1-one (**18**)

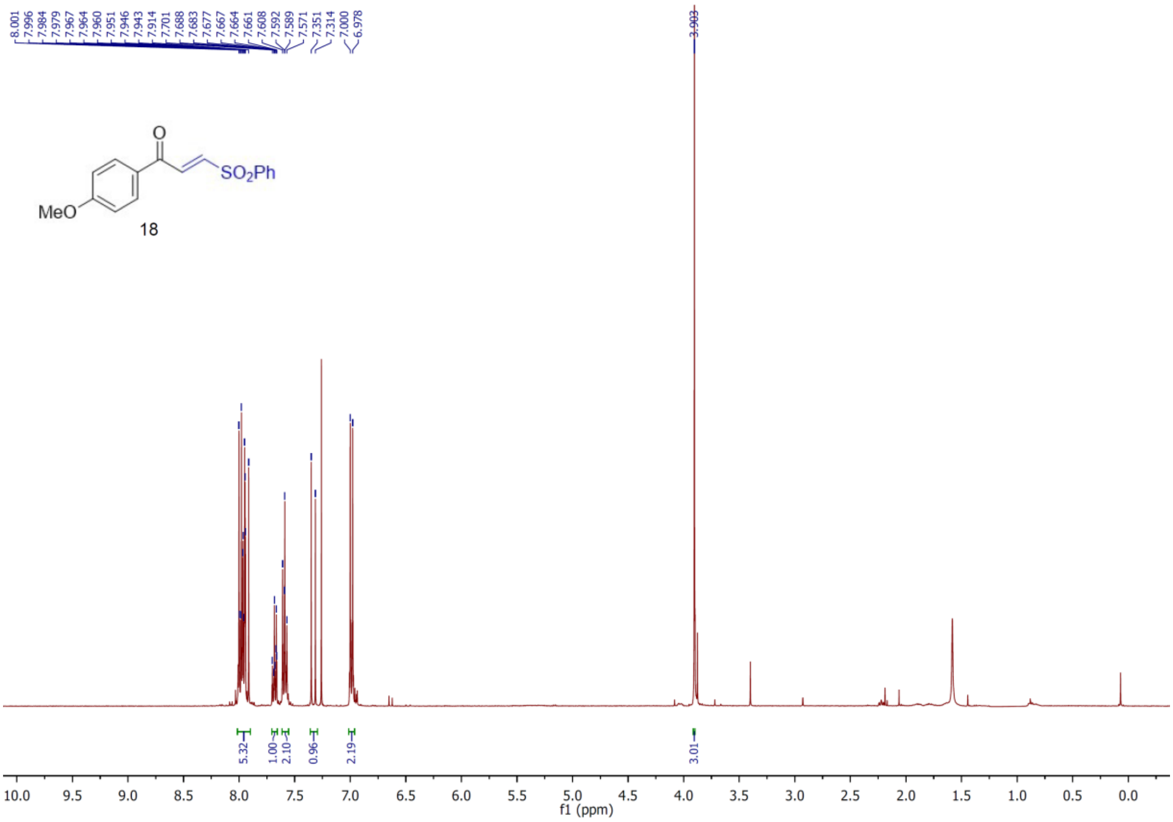

Supplementary Figure 109b | ^{13}C NMR (126 MHz, $CDCl_3$) of (*E*)-1-(4-Methoxyphenyl)-3-(phenylsulfonyl)prop-2-en-1-one (**18**)

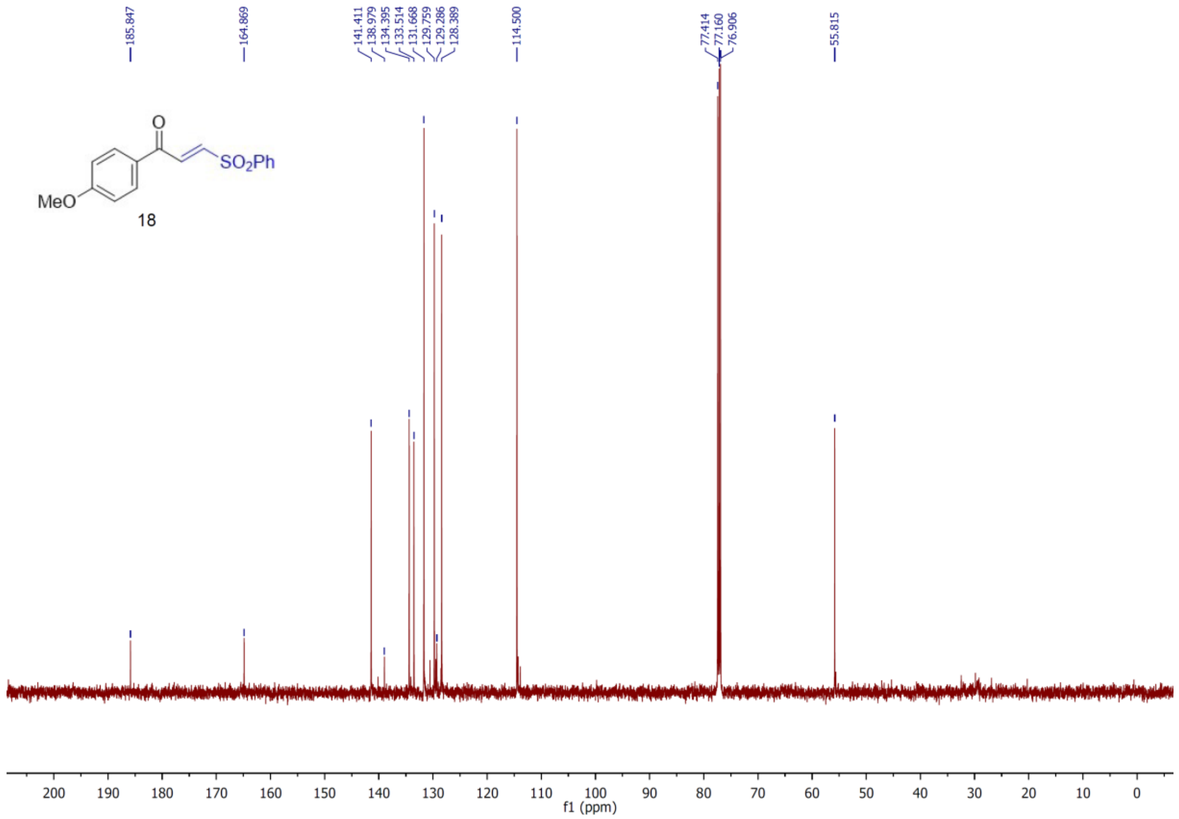

Supplementary References

1. Schmidt, A. M., Eilbracht, P. Total synthesis of the Fusarium toxin equisetin. *Org. Biomol. Chem*. **3**, 2333−2343 (2005).
2. Yan, J., Cheo, H. W., Teo, W. K., Shi, X., Wu, H., Idres, S. B., Deng, L. W., Wu, J. A radical smiles rearrangement promoted by neutral eosin Y as a direct hydrogen atom transfer photocatalyst. *J. Am. Chem. Soc*. **142**, 11357−11362 (2020).
3. Cao, H., Kuang, Y., Shi, X., Wong, K. L., Tan, B. B., Kwan, J. M. C., Wu, J. Photoinduced site-selective alkenylation of alkanes and aldehydes with aryl alkenes. *Nat. Commun*. **11**, 1956 (2020).
4. Zlotorzynska, M., Zhai, H., Sammis, G. M. Chemoselective oxygen-centered radical cyclizations onto silyl enol ethers. *Org. Lett*. **10**, 5083−5086 (2008).
5. Qian, P., Deng, Y., Mei, H., Han, J., Zhou, J., Pan, Y. Visible-light photoredox catalyzed oxidative/reductive cyclization reaction of N-cyanamide alkenes for the synthesis of sulfonated quinazolinones. *Org. Lett.* **19**, 4798–4801 (2017).
6. Liu, Y., Xie, P., Sun. Z., Wo. X., Gao. C., Fu, W., Loh, T.-P. Direct substitution of secondary and tertiary alcohols to generate sulfones under catalyst- and additive-free conditions. *Org. Lett.*, **20**, 5353–5356 (2018).
7. Li, H., Shan, C., Tung, C. H., Xu, Z. Dual gold and photoredox catalysis: visible light-mediated intermolecular atom transfer thiosulfonylation of alkenes. *Chem. Sci*. **8**, 2610−2615 (2017).
8. Zheng, Y., Qing, F. L., Huang, Y., Xu, X. H. Tunable and practical synthesis of thiosulfonates and disulfides from sulfonyl chlorides in the presence of tetrabutylammonium iodide. *Adv. Synth. Catal*. **358**, 3477−3481(2016).
9. Xia, Y., Wang, L., Studer, A. Site-selective remote radical C−H functionalization of unactivated C−H bonds in amides using sulfone reagents. *Angew. Chem. Int. Ed*. **57**, 12940 –12944 (2018).
10. Meesin, J., Katrun, P., Pareseecharoen, C., Reutrakul,M. V., Soorukram, D., Kuhakarn, C. Iodine-catalyzed sulfonylation of arylacetylenic acids and arylacetylenes with sodium sulfinates: synthesis of arylacetylenic sulfones. *J. Org. Chem.* **81**, 2744–2752 (2016).
11. Chen, M., Wang, J., Chai, Z., You, C., Lei, A. C-X (X=Br, I) Bond-tolerant aerobic oxidative cross- coupling: A strategy to selectively construct β-aryl ketones and aldehydes. *Adv. Synth. Catal.* **354**, 341–346 (2012).
12. Guo, S.-H., Wang, M.-Y., Pan, G.-F., Zhu, X.-Q., Gao, Y.-R. Wang, Y.-Q. Synthesis of monofluoromethylthioesters from aldehydes. *Adv. Synth. Catal.* **360**, 1861–1869 (2018).
13. Guo, S.-H., Zhang, X.-L. Pan, G.-F., Zhu, X.-Q. Gao, Y.-R. Wang, Y.-Q. Synthesis of difluoromethylthioesters from aldehydes. A*ngew. Chem., Int. Ed.* **57**, 1663–1667 (2017).
14. Xu, B., Li, D., Lu, L., Wang, D., Hu, Y., Shen, Q. Radical fluoroalkylthiolation of aldehydes with $PhSO_2SRf$ (Rf = CF_3, C_2F_5, CF_2H or CH_2F): a general protocol for the preparation of fluoroalkylthioesters. *Org. Chem. Front.* **5**, 2163−2166 (2018).
15. De Sarkar, S., Studer, A. Oxidative amidation and azidation of aldehydes by NHC catalysis. O*rg. Lett*. **12**, 1992 −1995 (2010).
16. Arisawa, M., Yamada, T., Yamaguchi, M. Rhodium-catalyzed interconversion between acid fluorides and thioesters controlled using heteroatom acceptors. *Tetrahedron Lett*. **51**, 6090−6092 (2010).
17. Xu, Y., Peng, Y., Sun, J., Chen, J., Ding, J., Wu, H. TCCA-promoted solvent-free chemoselective synthesis of thiosulfonates on grinding. *J. Chem. Res.* **6**, 358−360 (2010).
18. Burhardt, M. N., Taaning, R. H., Skrydstrup, T. Pd-catalyzed thiocarbonylation with stoichiometric carbon monoxide: scope and applications. *Org. Lett*. **15**, 948−951 (2013).
19. Zhao, X., Liu, T. X., Zhang, G. Synthesis of thiosulfonates via CuI-catalyzed reductive coupling of arenesulfonyl chlorides using Na_2SO_3 or $NaHSO_3$ as reductants. *Asian J. Org. Chem*. **6**, 677−681 (2017).

20. Reilly,S. W., Bennett, F. P., Fier, S., Ren, S. M., Strotman, N. A. Late-stage ^{18}O labeling of primary sulfonamides via a degradation-reconstruction pathway. *Chem. Eur. J.* **26**, 4251–4255 (2020).

21. Griesser, M., Chauvin, J. P. R., Pratt, D. A. The hydrogen atom transfer reactivity of sulfinic acids. *Chem. Sci.* **9**, 7218−7229 (2018).

22. Kice, J. L. & Bowers, W. K. The mechanism of the disproportionation of sulfinic acids. *J. Am. Chem. Soc.* **84**, 605–610 (1962).

23. Cao, L., Luo, S. H. Jiang, K., Hao, Z. F., Wang, B. W., Pang, C. M., Wang, Z. Y. Disproportionate coupling reaction of sodium sulfinates mediated by $BF_3{\cdot}OEt_2$: an approach to symmetrical/unsymmetrical thiosulfonates. *Org. Lett.* **20**, 4754−4758 (2018).

24. Hari, D. P. & König, B. Synthetic applications of eosin Y in photoredox catalysis. *Chem. Commun.* **50**, 6688−6699 (2014).

25. Banerjee, A., Lei, Z. & Ngai, M.-Y. Acyl radical chemistry via visible-light photoredox catalysis. *Synthesis* **51**, 303–333 (2019).

26. Persson, B. A*cta Chem. Scand.* **31B**, 88–89 (1997).

27. Roth, H. G., Romero, N. A., Nicewicz, D. A. Experimental and calculated electrochemical potentials of common organic molecules for applications to single-electron redox chemistry. *Synlett*, **27**: 714-723. (2016)

28. Gaussian 16, Revision A.03, Frisch, M. J., Trucks, G. W., Schlegel, H. B., Scuseria, G. E., Robb, M. A., Cheeseman, Scalmani, J. R., Barone, G. V., Petersson, G. A., Nakatsuji, H., Li, X., Caricato, M., Marenich, A. V., Bloino, J. B., Janesko, G., Gomperts, R., Mennucci, B. H., Hratchian, P., Ortiz, J. V., Izmaylov, A. F., Sonnenberg, J. L., Williams-Young, D., Ding, F., Lipparini, F., Egidi, F., Peng, J. B., Petrone, A., Henderson, T., Zakrzewski, D. V. G., Gao, J., Rega, N., Zheng, G., Liang, W., Hada, M., Ehara, M., Toyota, K., Fukuda, R., Hasegawa, J., Ishida, M., Nakajima, T., Honda, Y., Kitao, O., Nakai, H., Vreven, T., Throssell, K., Montgomery, J. A. Jr., Peralta, J. E., Ogliaro, F., Bearpark, M. J., Heyd, J. J., Brothers, E. N., Kudin, K. N., Staroverov, V. N., Keith, T. A., Normand, R. J., Raghavachari, K. A., Rendell,P., Burant, J. C., Iyengar, S. S., Tomasi, J., Cossi, M., Millam, J. M., Klene, M., Adamo, C., Cammi, R., Ochterski, J. W., Martin, R. L., Morokuma, K., Farkas, O., Foresman, J. B. & Fox, D. J. Gaussian, Inc., Wallingford CT, (2006).

29. Cismesia, M. A., Yoon, T. P. Characterizing chain processes in visible light photoredox catalysis. *Chem. Sci.* **6**, 5426−5434 (2015).

30. Hatchard, C. G., Parker, C. A., A new sensitive chemical actinometer. II. Potassium ferrioxalate as a standard chemical actinometer. *Proc. Roy. Soc. (London)* **A235**, 518−536 (1956).
