# Supplementary material for: Divergent functionalization of aldehydes photocatalyzed by neutral eosin Y with sulfone reagents
Source: Nat Commun. 2021 Dec 10;12:7214. doi: 10.1038/s41467-021-27550-8 (PMC8664905; doi:10.1038/s41467-021-27550-8)
Supplement: Supplementary file 2 — Source Data [file 41467_2021_27550_MOESM2_ESM.pdf]

Supplementary Data

Divergent Functionalization of Aldehydes Photocatalyzed by Neutral Eosin Y with Sulfone Reagents

Jianming Yan[1,2,*], Haidi Tang[1,3,*], Eugene Jun Rong Kuek[1], Xiangcheng Shi[1], Chenguang Liu[1], Muliang Zhang[1], Jared L. Piper[4], Shengquan Duan[4] & Jie Wu[1,3]

[1]Department of Chemistry, National University of Singapore, 3 Science Drive 3, Singapore 117543, Republic of Singapore

[2]Department of Medicinal Chemistry, College of Pharmacy, Chongqing Medical University, Chongqing 400016, China

[3]National University of Singapore (Suzhou) Research Institute, 377 Lin Quan Street, Suzhou Industrial Park, Suzhou, Jiangsu, 215123, China

[4]Pfizer Worldwide Research and Development, Eastern Point Rd, Groton, CT 06340, USA

*These authors contributed equally to this work.

Correspondence and requests for materials should be addressed to J.W. (email: chmjie@nus.edu.sg),

M.Z. (email: muliang0206@foxmail.com), S.D. (email: shengquan.duan@pfizer.com).

Table of Contents

Calculation of electrochemical potential of eosin Y-H species 2

DFT calculation on SET and RHAT pathways for eosin Y regeneration 5

Calculation of electrochemical potential of eosin Y-H species

Density functional theory (DFT) calculations were performed to study the electrochemical potential of eosin Y-H. The calculation method was adapted from Reference.[1] The redox potential $E_{1/2}^{o,calc}$ (vs SCE) was calculated according to the following equation:

$$E_{1/2}^{o,calc}(\text{vs SCE}) = -\frac{(G_{298}[reduced] - G_{298}[oxidized])}{n_e\mathcal{F}} - E_{1/2}^{o,SHE} - E_{1/2}^{o,SCE}$$

$$E_{1/2}^{ox}\text{(eosin Y-H)} = -0.11\text{ V vs SCE}$$

Where n_e is the number of electrons transferred (n_e=1 in our calculation), $\mathcal{F}$ is the Faraday constant (23.061 kcal mol^{-1} V^{-1}). $E_{1/2}^{o,SHE}$ is the absolute value for the standard hydrogen electrode (SHE, value = 4.281 V), $E_{1/2}^{o,SCE}$ is the potential of the saturated calomel electrode (SCE) relative to SHE in acetonitrile (value = 0.141 V). $G_{298}[reduced]$ and $G_{298}[oxidized]$ (1 Hartrees = 630 kcal/mol) are the calculated Gibbs free energies with the solvation model.

Computational Details

The geometries optimization in this study was performed at the uB3LYP/Def2SVP level of theory. The free energies of the optimized geometries were calculated at the same level of theory, considering the solvent effect using the Solvation Model Density (SMD) solvation model. All calculations were performed using the Gaussian 16 Rev. A.03 software suite. [2]

Calculated Cartesian Coordinates

Structure 1. eosin Y-H

Sum of electronic and zero-point Energies= -11438.095727

Sum of electronic and thermal Energies= -11438.069559

Sum of electronic and thermal Enthalpies= -11438.068615

Sum of electronic and thermal Free Energies= -11438.158042

C	-0.17520	3.19224	-1.71564
C	0.06052	2.66549	-0.43202
C	0.37347	3.57132	0.61440
C	0.46124	4.94799	0.34035
C	0.22124	5.44770	-0.93731
C	-0.10251	4.56328	-1.97006
H	-0.41092	2.50428	-2.53116
H	0.71513	5.62171	1.16053
H	0.28638	6.52186	-1.12685
H	-0.29173	4.93779	-2.97945
C	0.63191	3.18143	2.03815

O	1.16511	3.91048	2.84724
O	0.19219	1.95776	2.36579
H	0.41178	1.81158	3.30617
C	0.00263	1.18358	-0.27356
C	1.20310	0.39772	-0.22981
C	2.50542	0.94540	-0.30257
C	1.10216	-1.01617	-0.11732
C	3.62440	0.12672	-0.25603
H	2.62801	2.02370	-0.39937
C	2.23370	-1.83263	-0.07033
C	3.52938	-1.27507	-0.13721
C	-1.25709	0.50103	-0.21031
C	-2.51367	1.15398	-0.23106
C	-1.26650	-0.91422	-0.09950
C	-3.70222	0.44144	-0.15808
H	-2.54496	2.24063	-0.30423
C	-2.46575	-1.62350	-0.02788
C	-3.71495	-0.96532	-0.05595
Br	2.05022	-3.71462	0.08327
O	4.59109	-2.09122	-0.08871
H	5.40785	-1.56455	-0.14561
Br	5.37519	0.89594	-0.35748
Br	-5.36399	1.37303	-0.19150
O	-4.88424	-1.61550	0.01194
H	-4.72145	-2.57312	0.08041
O	-0.11162	-1.62929	-0.05569
Br	-2.45023	-3.52395	0.11386

Structure 2. eosin Y^{+}-H

Sum of electronic and zero-point Energies= -11437.939649
Sum of electronic and thermal Energies= -11437.913967
Sum of electronic and thermal Enthalpies= -11437.913023
Sum of electronic and thermal Free Energies= -11438.000243

C	-0.01505	3.07454	-1.78299
C	0.02978	2.63590	-0.44946
C	0.11569	3.59078	0.59151
C	0.15837	4.95562	0.26379
C	0.11367	5.38145	-1.06193
C	0.02571	4.43712	-2.08746
H	-0.07928	2.33946	-2.58827
H	0.22462	5.67807	1.07906
H	0.14615	6.44829	-1.29428
H	-0.01106	4.75590	-3.13190
C	0.15685	3.25762	2.05036

O	0.29167	4.07816	2.92857
O	0.01988	1.94584	2.30316
H	0.06012	1.81490	3.27066
C	0.00260	1.15690	-0.24067
C	1.21161	0.41940	-0.19118
C	2.49474	1.03111	-0.24424
C	1.14999	-1.00030	-0.08790
C	3.62785	0.25926	-0.19709
H	2.56292	2.11606	-0.32239
C	2.30484	-1.78676	-0.04105
C	3.56669	-1.16788	-0.09504
C	-1.22687	0.45647	-0.19253
C	-2.49634	1.10081	-0.23553
C	-1.19903	-0.96327	-0.08810
C	-3.65671	0.36973	-0.17935
H	-2.53341	2.18728	-0.31242
C	-2.37987	-1.70844	-0.03768
C	-3.62662	-1.05887	-0.07942
Br	2.18979	-3.66668	0.08996
O	4.64592	-1.92780	-0.05099
H	5.45691	-1.38540	-0.09609
Br	5.35351	1.06112	-0.26600
Br	-5.34231	1.23309	-0.22858
O	-4.77745	-1.70301	-0.03066
H	-4.63816	-2.66748	0.03637
O	-0.03630	-1.62995	-0.04092
Br	-2.32916	-3.60010	0.08467

DFT calculation on SET and RHAT pathways for eosin Y regeneration

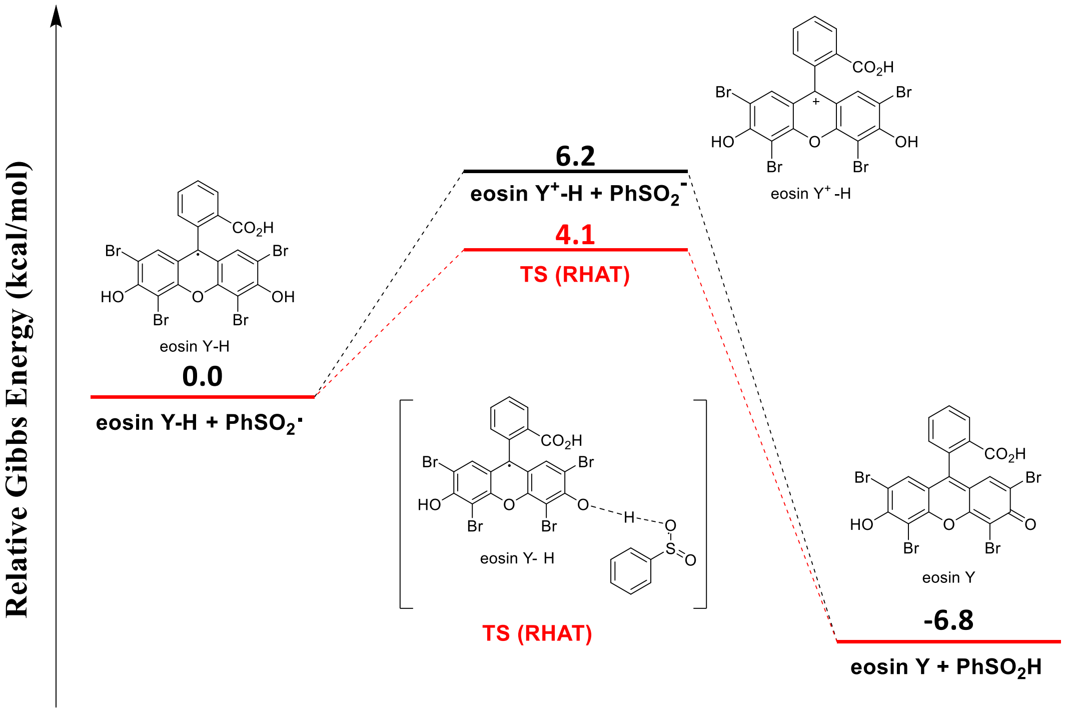

Supplementary Figure 17. Density functional theory (DFT) calculations on eosin Y regeneration.

Computational details

Density functional theory (DFT) calculations were performed to shed light on the mechanism of eosin Y regeneration (Supplementary Figure 17). Reverse hydrogen atom transfer (RHAT, red line) is the favored pathway, which features a barrier 2.1 kcal/mol lower than an alternative single electron transfer (SET, black line). The geometries optimization in this study was performed at the uB3LYP density functional with a standard def2-SVP basis set. The nature of the stationary points (minima with no imaginary frequency or transition states with one imaginary frequency) were confirmed. The free energies of the optimized geometries were calculated at the same level of theory, considering the solvent effect of acetone using an SMD continuum solvation model. Unless specified otherwise, the Gibbs free energy was used throughout. For transition state, intrinsic reaction coordinate (IRC) calculations were performed to verify whether it connected with correct reactants and products or intermediates. All calculations were performed using the Gaussian 16 Rev. A.03 software suite.[2]

Calculated Cartesian Coordinates

$PhSO_2^{\bullet}$

Sum of electronic and zero-point Energies= -779.715038
Sum of electronic and thermal Energies= -779.707423

Sum of electronic and thermal Enthalpies= -779.706479

Sum of electronic and thermal Free Energies= -779.748770

——

C	2.16539	1.21528	0.03415
C	0.77114	1.22604	-0.05578
C	0.10036	0.00001	-0.08355
C	0.77119	-1.22602	-0.05617
C	2.16543	-1.21525	0.03372
C	2.85902	0.00003	0.07770
H	2.71022	2.16177	0.07080
H	0.21597	2.16501	-0.08934
H	0.21608	-2.16502	-0.08994
H	2.71032	-2.16172	0.06999
H	3.94986	0.00004	0.14462
S	-1.70522	-0.00011	-0.25892
O	-2.21959	-1.29615	0.27148
O	-2.21968	1.29628	0.27054

Eosin Y-H

Sum of electronic and zero-point Energies= -11438.097330

Sum of electronic and thermal Energies= -11438.071167

Sum of electronic and thermal Enthalpies= -11438.070223

Sum of electronic and thermal Free Energies= -11438.159590

——

C	-0.17520	3.19224	-1.71564
C	0.06052	2.66549	-0.43202
C	0.37347	3.57132	0.61440
C	0.46124	4.94799	0.34035
C	0.22124	5.44770	-0.93731
C	-0.10251	4.56328	-1.97006
H	-0.41092	2.50428	-2.53116
H	0.71513	5.62171	1.16053
H	0.28638	6.52186	-1.12685
H	-0.29173	4.93779	-2.97945
C	0.63191	3.18143	2.03815
O	1.16511	3.91048	2.84724
O	0.19219	1.95776	2.36579
H	0.41178	1.81158	3.30617
C	0.00263	1.18358	-0.27356
C	1.20310	0.39772	-0.22981
C	2.50542	0.94540	-0.30257
C	1.10216	-1.01617	-0.11732
C	3.62440	0.12672	-0.25603
H	2.62801	2.02370	-0.39937

C	2.23370	-1.83263	-0.07033
C	3.52938	-1.27507	-0.13721
C	-1.25709	0.50103	-0.21031
C	-2.51367	1.15398	-0.23106
C	-1.26650	-0.91422	-0.09950
C	-3.70222	0.44144	-0.15808
H	-2.54496	2.24063	-0.30423
C	-2.46575	-1.62350	-0.02788
C	-3.71495	-0.96532	-0.05595
Br	2.05022	-3.71462	0.08327
O	4.59109	-2.09122	-0.08871
H	5.40785	-1.56455	-0.14561
Br	5.37519	0.89594	-0.35748
Br	-5.36399	1.37303	-0.19150
O	-4.88424	-1.61550	0.01194
H	-4.72145	-2.57312	0.08041
O	-0.11162	-1.62929	-0.05569
Br	-2.45023	-3.52395	0.11386

$PhSO_2^-$

Sum of electronic and zero-point Energies= -779.865145
Sum of electronic and thermal Energies= -779.857451
Sum of electronic and thermal Enthalpies= -779.856507
Sum of electronic and thermal Free Energies= -779.898401

——

C	2.17417	1.21752	0.05878
C	0.77782	1.20715	-0.04940
C	0.08890	-0.00636	-0.12330
C	0.79424	-1.21283	-0.08550
C	2.19044	-1.20747	0.02048
C	2.88268	0.00913	0.08983
H	2.71333	2.16794	0.12492
H	0.19844	2.13544	-0.05789
H	0.23186	-2.15095	-0.12006
H	2.74244	-2.15211	0.05588
H	3.97335	0.01505	0.17309
S	-1.78175	-0.01580	-0.35702
O	-2.17439	-1.26948	0.42854
O	-2.17574	1.29382	0.33033

Eosin Y^+-H

Sum of electronic and zero-point Energies= -11437.939381
Sum of electronic and thermal Energies= -11437.913746
Sum of electronic and thermal Enthalpies= -11437.912801

Sum of electronic and thermal Free Energies= -11438.000015

——

C	-0.01505	3.07454	-1.78299
C	0.02978	2.63590	-0.44946
C	0.11569	3.59078	0.59151
C	0.15837	4.95562	0.26379
C	0.11367	5.38145	-1.06193
C	0.02571	4.43712	-2.08746
H	-0.07928	2.33946	-2.58827
H	0.22462	5.67807	1.07906
H	0.14615	6.44829	-1.29428
H	-0.01106	4.75590	-3.13190
C	0.15685	3.25762	2.05036
O	0.29167	4.07816	2.92857
O	0.01988	1.94584	2.30316
H	0.06012	1.81490	3.27066
C	0.00260	1.15690	-0.24067
C	1.21161	0.41940	-0.19118
C	2.49474	1.03111	-0.24424
C	1.14999	-1.00030	-0.08790
C	3.62785	0.25926	-0.19709
H	2.56292	2.11606	-0.32239
C	2.30484	-1.78676	-0.04105
C	3.56669	-1.16788	-0.09504
C	-1.22687	0.45647	-0.19253
C	-2.49634	1.10081	-0.23553
C	-1.19903	-0.96327	-0.08810
C	-3.65671	0.36973	-0.17935
H	-2.53341	2.18728	-0.31242
C	-2.37987	-1.70844	-0.03768
C	-3.62662	-1.05887	-0.07942
Br	2.18979	-3.66668	0.08996
O	4.64592	-1.92780	-0.05099
H	5.45691	-1.38540	-0.09609
Br	5.35351	1.06112	-0.26600
Br	-5.34231	1.23309	-0.22858
O	-4.77745	-1.70301	-0.03066
H	-4.63816	-2.66748	0.03637
O	-0.03630	-1.62995	-0.04092
Br	-2.32916	-3.60010	0.08467

TS (HAT)

Sum of electronic and zero-point Energies= -12217.824902

Sum of electronic and thermal Energies= -12217.790042

Sum of electronic and thermal Enthalpies= -12217.789098

Sum of electronic and thermal Free Energies= -12217.901894

——

C	2.86194	3.16522	-1.63468
C	2.46652	2.68693	-0.37482
C	2.57160	3.54137	0.74945
C	3.06011	4.84678	0.57342
C	3.44894	5.31026	-0.68131
C	3.35008	4.46467	-1.78888
H	2.78152	2.50910	-2.50429
H	3.12884	5.49160	1.45106
H	3.82797	6.32854	-0.79460
H	3.65067	4.81297	-2.78009
C	2.19507	3.16380	2.14881
O	2.20261	3.93543	3.08123
O	1.85433	1.87330	2.28544
H	1.61886	1.71847	3.22090
C	1.94470	1.28622	-0.33110
C	2.84869	0.19245	-0.18289
C	4.24219	0.36006	0.01866
C	2.33363	-1.13006	-0.23256
C	5.05963	-0.73769	0.15433
H	4.65432	1.36772	0.06577
C	3.16790	-2.24682	-0.10172
C	4.54849	-2.06864	0.09433
C	0.58064	1.01974	-0.52626
C	-0.40258	2.04220	-0.70418
C	0.13423	-0.34230	-0.54737
C	-1.72071	1.72007	-0.86919
H	-0.08353	3.08401	-0.69859
C	-1.20470	-0.66934	-0.72388
C	-2.19156	0.34808	-0.88305
Br	2.45601	-3.99550	-0.18247
O	5.31792	-3.14170	0.21523
H	6.24888	-2.88173	0.34796
Br	6.93495	-0.52258	0.43158
Br	-3.01702	3.08881	-1.08720
O	-3.44200	0.10766	-1.06164
O	1.01857	-1.34905	-0.40727
Br	-1.73871	-2.48401	-0.80917
H	-3.95987	-0.66663	-0.38595
C	-6.54701	-0.69749	0.74723
C	-7.54118	-1.31003	-0.02075
C	-6.79058	0.51151	1.40518

C	-8.79762	-0.70405	-0.12910
H	-7.32737	-2.26117	-0.51421
C	-8.04829	1.11389	1.29326
H	-5.99954	0.96367	2.00820
C	-9.05035	0.50759	0.52563
H	-9.58316	-1.18017	-0.72272
H	-8.24910	2.05686	1.80966
H	-10.03267	0.98001	0.43924
O	-4.33637	-1.03405	2.17975
O	-5.11507	-2.92691	0.63179
S	-4.87671	-1.45765	0.84026

$PhSO_2H$

Sum of electronic and zero-point Energies= -780.311851
Sum of electronic and thermal Energies= -780.304312
Sum of electronic and thermal Enthalpies= -780.303368
Sum of electronic and thermal Free Energies= -780.345025

——

C	-2.18685	1.21526	-0.03283
C	-0.79181	1.22316	0.04602
C	-0.11461	-0.00001	0.08600
C	-0.79185	-1.22316	0.04616
C	-2.18689	-1.21523	-0.03269
C	-2.88063	0.00002	-0.07051
H	-2.73293	2.16114	-0.06818
H	-0.23506	2.16196	0.06816
H	-0.23514	-2.16199	0.06837
H	-2.73299	-2.16111	-0.06792
H	-3.97196	0.00003	-0.13271
O	2.18528	-1.27852	-0.31195
O	2.18530	1.27859	-0.31159
S	1.67458	-0.00003	0.20576
H	1.86595	-0.00023	1.57547

Eosin Y

Sum of electronic and zero-point Energies= -11437.512694
Sum of electronic and thermal Energies= -11437.487032
Sum of electronic and thermal Enthalpies= -11437.486087
Sum of electronic and thermal Free Energies= -11437.574106

——

C	-0.15096	3.08714	-1.75740
C	-0.04019	2.62192	-0.43605
C	0.10157	3.56726	0.60990

C	0.12350	4.93792	0.29800
C	0.01133	5.38390	-1.01659
C	-0.12630	4.45262	-2.04887
H	-0.25701	2.36265	-2.56806
H	0.22964	5.64897	1.11890
H	0.03030	6.45423	-1.23482
H	-0.21584	4.78493	-3.08613
C	0.22920	3.23103	2.06440
O	0.38395	4.05919	2.93489
O	0.15490	1.91913	2.33209
H	0.25172	1.80599	3.29745
C	-0.06080	1.13688	-0.24820
C	1.18478	0.40925	-0.20673
C	2.45044	1.03093	-0.27103
C	1.13868	-1.00082	-0.09900
C	3.60401	0.27263	-0.22456
H	2.51070	2.11551	-0.35888
C	2.31022	-1.76817	-0.05328
C	3.56811	-1.14185	-0.11422
C	-1.25485	0.43521	-0.19928
C	-2.54773	1.07161	-0.23793
C	-1.23025	-1.00885	-0.08849
C	-3.69045	0.34215	-0.17865
H	-2.58636	2.15814	-0.31493
C	-2.38376	-1.75020	-0.03106
C	-3.71791	-1.14018	-0.07135
Br	2.21711	-3.65307	0.08894
O	4.66459	-1.89690	-0.06861
H	5.45925	-1.33493	-0.12012
Br	5.31790	1.11472	-0.30888
Br	-5.38453	1.20148	-0.22917
O	-4.75967	-1.78304	-0.02097
O	-0.03819	-1.65531	-0.04116
Br	-2.31559	-3.63919	0.10738

References:

1. Roth, H. G., Romero, N. A., Nicewicz, D. A. Experimental and calculated electrochemical potentials of common organic molecules for applications to single-electron redox chemistry. *Synlett*, **27**: 714-723. (2016)
2. Gaussian 16, Revision A.03, Frisch, M. J., Trucks, G. W., Schlegel, H. B., Scuseria, G. E., Robb, M. A., Cheeseman, Scalmani, J. R., Barone, G. V., Petersson, G. A., Nakatsuji, H., Li, X., Caricato, M., Marenich, A. V., Bloino, J. B., Janesko, G., Gomperts, R., Mennucci, B. H., Hratchian, P., Ortiz, J. V., Izmaylov, A. F., Sonnenberg, J. L., Williams-Young, D., Ding, F., Lipparini, F., Egidi, F., Peng, J. B., Petrone, A., Henderson, T., Zakrzewski, D. V. G., Gao, J., Rega, N., Zheng, G., Liang, W., Hada, M., Ehara, M., Toyota, K., Fukuda, R., Hasegawa, J., Ishida, M., Nakajima, T., Honda, Y., Kitao, O., Nakai, H., Vreven, T., Throssell, K., Montgomery, J. A. Jr., Peralta, J. E., Ogliaro, F., Bearpark, M. J., Heyd, J. J., Brothers, E. N., Kudin, K. N., Staroverov, V. N., Keith, T. A., Normand, R. J., Raghavachari, K. A., Rendell,P., Burant, J. C., Iyengar, S. S., Tomasi, J., Cossi, M., Millam, J. M., Klene, M., Adamo, C., Cammi, R., Ochterski, J. W., Martin, R. L., Morokuma, K., Farkas, O., Foresman, J. B. & Fox, D. J. Gaussian, Inc., Wallingford CT, (2006).
